# Enantioselective Allylation of Stereogenic Nitrogen Centers

**DOI:** 10.1021/acs.orglett.3c00195

**Published:** 2023-03-07

**Authors:** Snizhana Zaitseva, Alessandro Prescimone, Valentin Köhler

**Affiliations:** Department of Chemistry, University of Basel, Mattenstrasse 22, 4058 Basel, Switzerland

## Abstract

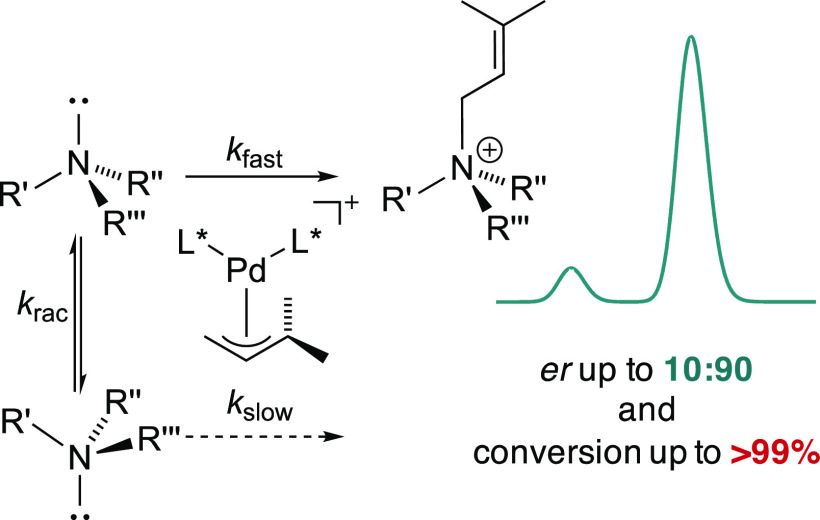

Most tertiary amines with a stereogenic nitrogen center undergo rapid racemization at room temperature. Consequently, the quaternization of amines under dynamic kinetic resolution seems feasible. *N*-Methyl tetrahydroisoquinolines are converted into configurationally stable ammonium ions by Pd-catalyzed allylic alkylation. The optimization of conditions and the evaluation of the substrate scope enabled high conversions and an enantiomeric ratio of up to 10:90. We report here the first examples for the enantioselective catalytic synthesis of chiral ammonium ions.

The latent chirality of tertiary amines has attracted the attention of chemists for well over a hundred years.^1^ sp^3^-hybridized nitrogen atoms with three different substituents constitute stereogenic centers that typically racemize rapidly at room temperature, with exceptions being nitrogen centers at bridgehead positions. Steric hindrance, electronegative substituents, and ring strain increase the activation barrier for pyramidal inversion at nitrogen.^2^

Although rarely explored as a structural element in synthesis, stereogenic quaternary nitrogen can be found in natural products^3^ and in a few instances in pharmaceutical compounds.^4^ Chiral ammonium ions are successfully applied in phase transfer catalysis and as chiral cations for anionic transition metal (TM) catalysts to control the stereochemical reaction outcome.^5^ Likewise, ammonium-ion-linked phosphine ligands have served in ion-pair-controlled stereoselective reactions.^6^ The ability of alkylammonium ions to form strong hydrogen bonds via their C–H acidic α substituents and their substantially altered solubility compared to quaternary carbon analogues renders them intriguing motifs for medicinal chemistry.^7^ Hofmann elimination and substitution of the tertiary amine represent viable degradation pathways and have been exploited in the pro-drug atracurium and for dynamic resolution approaches.^1d,4a,8^

Despite this potential, no enantioselective catalytic method for the synthesis of chiral ammonium ions with stereogenic nitrogen centers has been published to date. Current methods rely on substrate control in diastereoselective quaternization reactions or resolution by diastereomeric salt or adduct formation. The latter two approaches require stoichiometric amounts of an enantiomerically enriched additive.^8,9^ Remarkably, tertiary amines have been converted stereoselectively with various oxidants to chiral *N*-oxides in the presence of bovine serum albumin as a dirigent protein [up to 66.8% enantiomeric excess (ee)] or enzymatically with cyclohexanone monooxygenase from *Acinetobacter calcoaceticus* (up to 32% ee).^10^

Allylation is one of the few electrophilic alkylation reactions that can be efficiently catalyzed by TM complexes.^11^ While only a handful of reports have appeared for the TM-mediated allylation of tertiary amines,^12^ the reverse reaction, namely, the catalytic deallylation of quaternary ammonium ions, was utilized by Hirao and co-workers already in 1982.^13^ Stereoselective deallylation offers, in principle, the possibility to resolve chiral allyl ammonium ions in a kinetic resolution (Figure 1A). Kinetic resolution is, however, limited to a maximum yield of 50% for one enantiomer. We reasoned that the rapid interconversion of the enantiomeric forms of the amine should enable a dynamic kinetic resolution approach for electrophilic quaternization, thus raising the maximum theoretical yield to 100% (Figure 1B).

**Figure 1:**
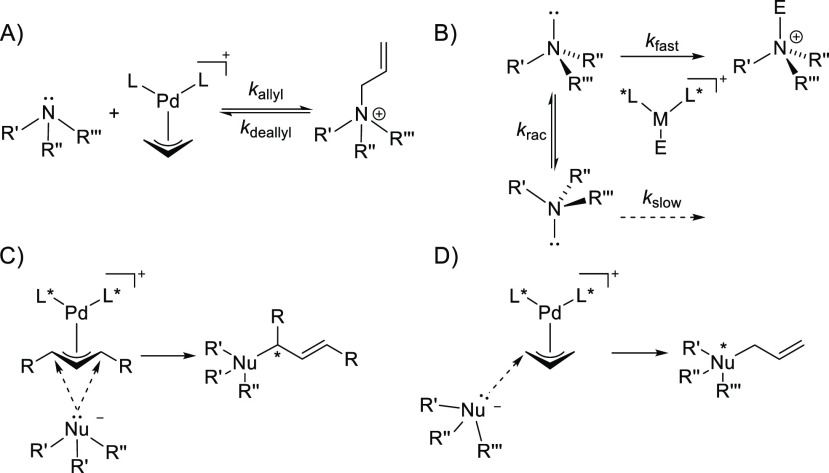
(A) Reversible allylation of tertiary amines. (B) Dynamic kinetic resolution of chiral amines via electrophilic quaternization. (C) Selective attack of the nucleophile results in a new sterogenic center derived from the allyl moiety. (D) The stereogenic center is derived from the nucleophilic moiety.

Stereoselective allylic alkylation usually aims to install a stereogenic center at a terminal carbon atom of the electrophilic allyl fragment (Figure 1C). The more challenging reaction with stereogenic or prostereogenic nucleophiles can also proceed with high enantioselectivity (Figure 1D).^14^

The key for the development of the process was the identification of suitable reaction conditions for high conversion and high stereochemical induction, i.e., reaction medium, electrophile, and chiral ligand. Furthermore, the racemization of the product had to be prevented.

*N*-Methyl tetrahydroisoquinoline (N-Me THIQ, **1a**; Figure 2) was selected as a model substrate based on the pharmaceutical relevance of the THIQ core,^4e^ an expected high nucleophilicity for a tertiary amine,^15^ and ease of access. We also considered the challenging enantiomer discrimination of a substrate with little difference in the steric demand of the substituents in immediate proximity to nitrogen.

**Figure 2:**
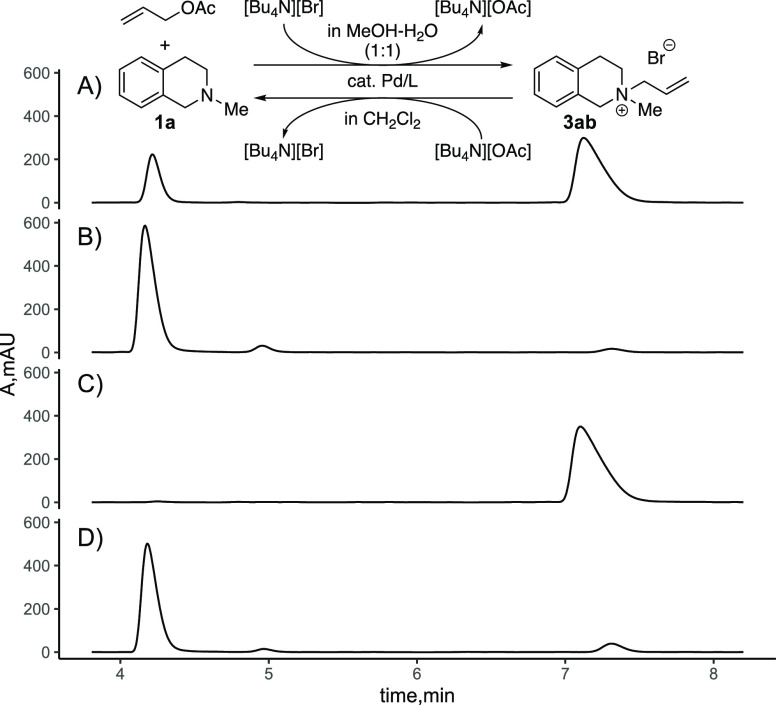
Equilibrium shift in high versus low polarity solvents. HPLC traces for allylation and deallylation reactions after 21 h with 0.50 mol % [PdCl(C_3_H_5_)]_2_, 1.03 mol % (*S*,*S*)-DACH-phenyl-Trost ligand, (A) 10 mM amine **1a** + 1 equiv of allyl acetate + 1 equiv of [Bu_4_N]Br in MeOH/water (1:1), (B) 10 mM amine **1a** + 1 equiv of allyl acetate + 1 equiv of [Bu_4_N]Br in CH_2_Cl_2_, (C) 10 mM salt [**3ab**]Br + 1 equiv of [Bu_4_N][OAc] in MeOH/water (1:1), and (D) 10 mM salt [**3ab**]Br + 1 equiv of [Bu_4_N][OAc] in CH_2_Cl_2_. The signal at 4.95 min results from the uncatalyzed reaction of amine **1a** with dichloromethane, i.e., the corresponding chloromethylated ammonium ion. See the Supporting Information for HPLC conditions.

Commonly employed reaction conditions for AAA, e.g., CH_2_Cl_2_ as a solvent, [PdCl(C_3_H_5_)]_2_ as a catalyst precursor, chiral diphosphines as ligands, and allyl acetate as the electrophile,^15^ showed only negligible conversion to the desired product (entries 1 and 2 in Table 1). Initial tests with more reactive electrophiles, such as allyl bromides, chlorides, mesylates, or tosylates, resulted in high uncatalyzed allylation rates. Crucial for the realization of high conversions without concomitant background reaction was the employment of electrophiles of lower inherent reactivity, e.g., methyl carbonates in combination with polar protic solvents. The preference for a polar reaction medium is believed to reside in a change in the thermodynamic driving force: while highly polar solvents can readily stabilize the ionic products formed, charge separation is disfavored in less polar solvents. Experimental support for this hypothesis was obtained by comparing the conversion of the deallylation reaction of the allyl ammonium ion *rac*-**3ab** in different solvents. The reaction of *rac*-**3ab** in the presence of 1 equiv of [Bu_4_N][OAc] as a source of nucleophilic acetate, 0.50 mol % [PdCl(C_3_H_5_)]_2_, and 1.03 mol % (*S*,*S*)-DACH-phenyl-Trost ligand led to 73% conversion to compound **1a** in 15 min in CH_2_Cl_2_, whereas no conversion was observed by high-performance liquid chromatography (HPLC) in MeOH/H_2_O (1:1) after 21 h. The reversed trend was observed for the “forward reaction” of compound **1a** to compound **3ab** with allyl acetate as the electrophile (Figure 2 and entries 2 and 3 in Table 1).

**Table 1:**
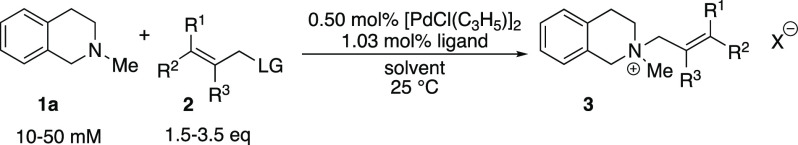

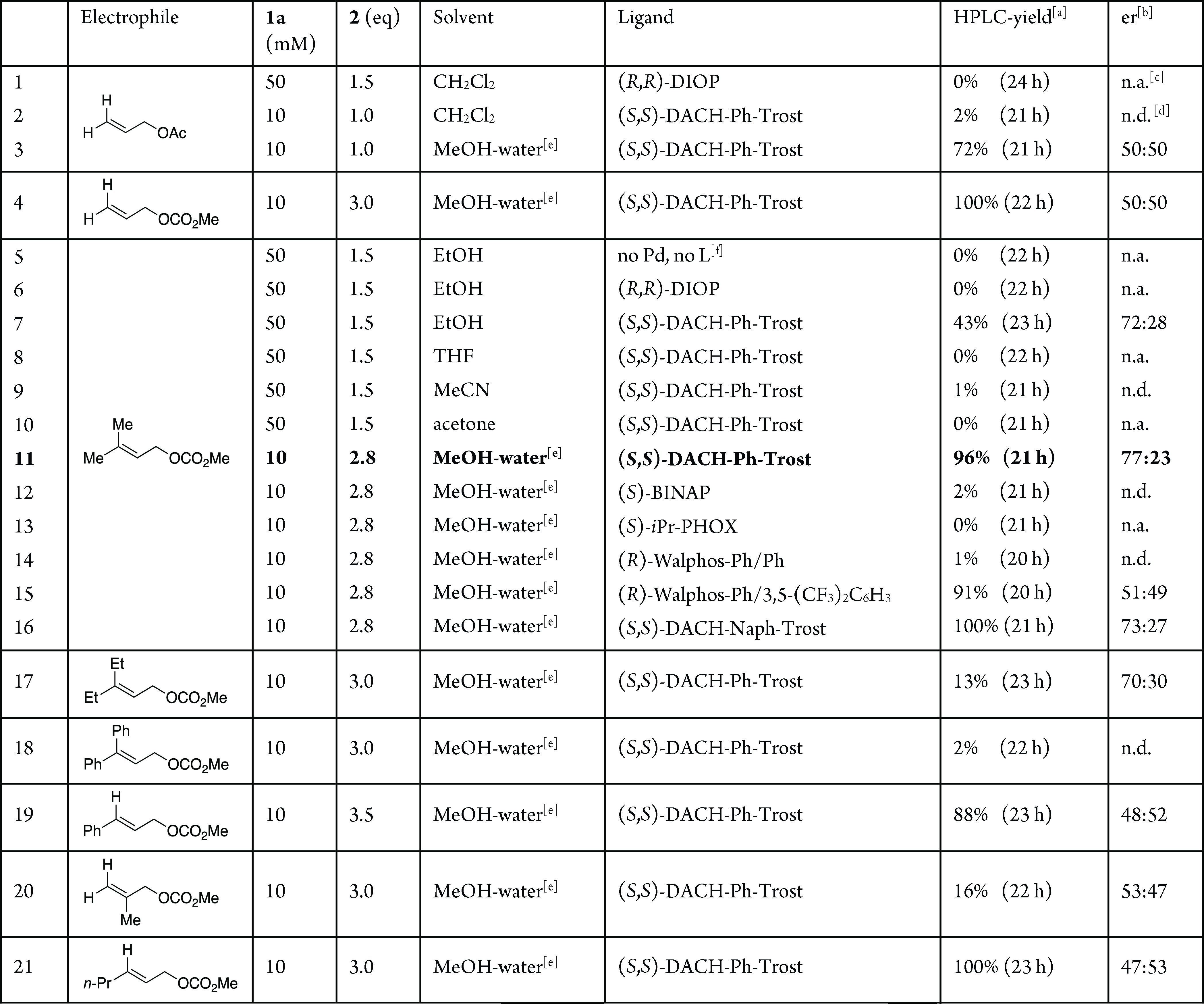
Selected Screening Results for the Catalytic Formation of Quaternary Ammonium Ions^g^

a The HPLC yield was determined by reversed-phase HPLC (210 nm), considering the experimentally determined response factor.

b The enantiomeric ratio (er) of compound **3** was determined by chiral-phase HPLC. Enantiomeric ratios are listed in the order of elution from chiral-phase HPLC.

c n.a. = not applicable.

d n.d. = not determined.

e A 1:1 mixture.

f No [PdCl(C_3_H_5_]_2_ and ligand were added. Further alternative conditions can be found in the Supporting Information.

g Reactions were run on a 1.0 mL scale. Catalyst loading for entries 1 and 6, Pd = 1.0 mol % and L_2_ = 2.0 mol %; catalyst loading for entries 8–10, Pd = 0.20 mol % and L_2_ = 0.41 mol %; and catalyst loading for all other entries, Pd = 0.50 mol % and L_2_ = 1.03 mol %.

Reactions with unsubstituted allyl electrophiles yielded exclusively the racemic product, possibly as a result of racemization of the ammonium ions under the reaction conditions (Tables S2 and S8 of the Supporting Information). Enantioenriched material **3ab**, obtained by fractional crystallization of a diastereomeric salt, showed indeed rapid racemization in the presence of the catalyst when the amine nucleophile **1a** was added (Scheme 1 and Figure S4 of the Supporting Information). In the absence of an additional nucleophile, the ammonium ion does not racemize notably for extended periods of time.

**Scheme 1:**
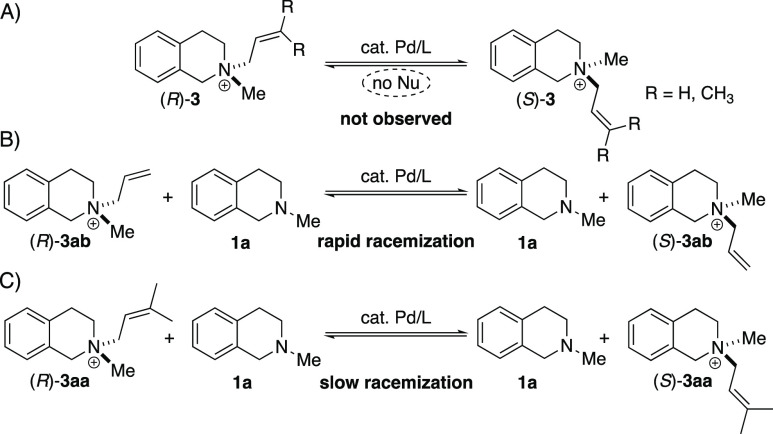
Racemization of the Allyl Ammonium Salts **3aa** and **3ab** Conditions: 0.50 mol % [PdCl(C_3_H_5_)]_2_, 1.03 mol % (*S*,*S*)-DACH-phenyl-Trost, MeOH/water = 1:1, (A) 10 mM [**3ab**][CF_3_CO_2_] (er of 93:7) or (*R*)-[**3aa**][CF_3_CO_2_] (er of 90.5:9.5), with no amine nucleophile added, (B) 10 mM [**3ab**][CF_3_CO_2_] + 1.0 equiv of compound **1a**, and (C) [**3aa**][CF_3_CO_2_] + 1.0 equiv of compound **1a**. Racemization was monitored by chiral-phase HPLC.

The introduction of geminal methyl groups at the terminus of the allyl moiety (**3aa**; Scheme 1) resulted in a strong reduction of the racemization rate (Scheme 1C; see also the Supporting Information). Consequently, prenyl methyl carbonate (**2a**) was examined as an electrophile for the allylation reaction.

Gratifyingly, for the first time, the enantioselective formation of compound **3aa** was observed by chiral-phase HPLC (entry 7 in Table 1) when compound **2a** was employed with (*S*,*S*)-DACH-phenyl-Trost as a chiral ligand.

A structurally diverse set of 40 chiral ligands, mainly diphosphines, was screened with respect to conversion and enantioselectivity (see Table 1 and the Supporting Information). This screen revealed that (i) larger bite angles and a flexible ligand scaffold are advantageous with respect to the reaction rate, (ii) Walphos-type ligands with electron-poor phosphines lead to higher conversion than their electron-rich counterparts, and (iii) Trost-type ligands enable high conversion and significant enantioselectivity. It needs to be noted that the leaving group, ligand, and solvent affect rates in a sensitive interplay.

Further reaction optimization (see the Supporting Information) resulted in the following conditions: 10 mM amine nucleophile, 28 mM methyl prenyl carbonate **2a** as the electrophile, 0.50 mol % [PdCl(C_3_H_5_)]_2_ as the catalyst precursor, 1.03 mol % (*S*,*S*)-DACH-phenyl-Trost ligand, and MeOH/water in a ratio of 1:1 as the solvent system. Observed side reactions were the Pd-catalyzed formation of isoprene and the formation of 2-methylpent-4-en-2-ol and the corresponding methyl ether from the reaction of the nucleophilic solvents H_2_O and MeOH with prenyl methyl carbonate (see the Supporting Information). Hofmann elimination was only observed when the catalytic reaction was concentrated and lyophilized for product isolation (around 5% side product, on a 1 mmol scale). Workup in the presence of a volatile buffer (basic ammonium acetate at pH 9.85) removed this problem reliably. A simple workup consisting of the concentration step, addition of ammonium acetate buffer, washing with *tert*-butyl methyl ether (TBME), and removal of volatiles allowed for isolation of the ammonium ion in good purity and high yield (82%) without any chromatographic steps [see the Supporting Information for representative nuclear magnetic resonance (NMR) spectra]. No Hofmann elimination was observed when ammonium ion **3aa** (10 mM) was treated with 1 equiv of NaOD in CD_3_OD/D_2_O (1:1) at room temperature for 24 h.

We determined the absolute configuration of compound **3aa** in three independent crystals of a diastereomerically enriched dibenzoyltartrate salt by X-ray diffraction. A comparison of the chiral-phase HPLC data to the catalytic reaction showed that the major product in the enantioselective allylation of compound **1a** with compound **2a** had the *R* configuration when (*S*,*S*)-DACH-phenyl-Trost ligand was employed.

A set of related amines (Scheme 2) was then subjected to allylation. As expected, the substrate structure has significant influence on the catalysis outcome. Increasing the exocyclic substituent at nitrogen from methyl to ethyl in substrate **1r** led to lower conversion and a lower enantiomeric ratio. *N*-Methyl tetrahydro-1*H*-benzoazepine **1j** showed good conversion but only formed the racemic product. Aromatic amine *N*-methyl tetrahydroquinoline **1t** and linear substrate **1s** did not form the allyl ammonium ion under the conditions investigated. A low conversion and racemic product were also observed in the reaction of linear substrate **1p**.

**Scheme 2:**
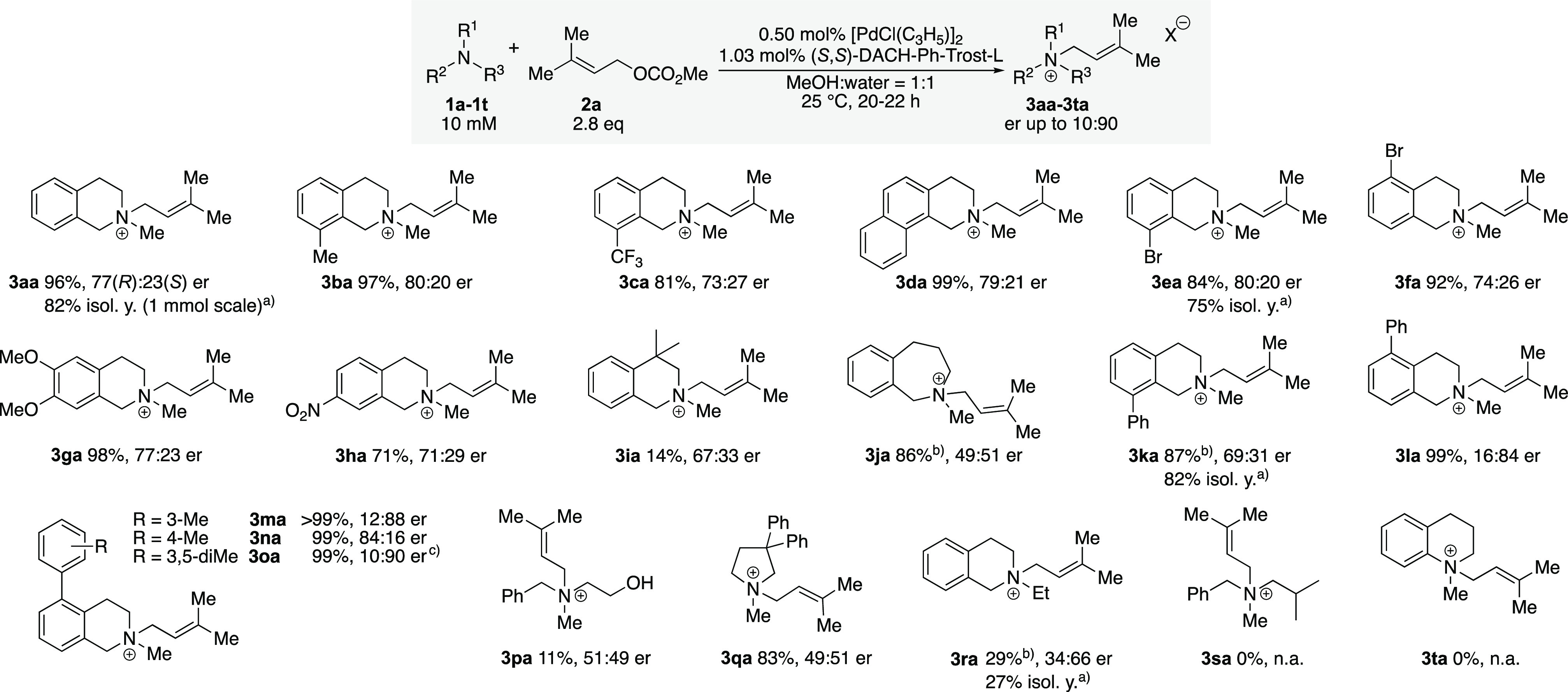
HPLC Yields and Enantiomeric Ratios of Various Ammonium Ions Prepared by AAA The enantiomeric ratios of the isolated salts were the same as the enantiomeric ratios observed in the analytical samples. Scale for the preparative reactions: **3aa**, 1.0 mmol; **3ea**, 0.22 mmol; **3ka**, 78 μmol; and **3ra**, 0.10 mmol. Conversion was determined by ^1^H NMR. Reaction was performed at 20 °C. HPLC yields were determined by reversed-phase HPLC under ultraviolet (UV) detection (210 nm) under the consideration of the experimentally determined response factors. The enantiomeric ratio was determined by chiral-phase HPLC. Enantiomeric ratios are listed in the order of elution in chiral-phase HPLC and do not indicate absolute configuration unless noted. X = HCO_3_ or OAc.^19^ n.a. = not applicable.

Alternative substitution patterns of the allylcarbonate, i.e., larger groups in the 3 position, substitution at the 2 position, or monosubstitution at the 3 position, led all to either low rates or nearly the racemic product (entries 17–21 in Table 1).

Substituents at the aromatic ring of the N-Me THIQ substrates had mostly a moderate effect but, remarkably, enabled the realization of an er of 10:90 for compound **3oa** when the reaction was performed at 20 °C.

In conclusion, we have demonstrated for the first time that configurationally labile stereogenic nitrogen centers can be converted in an enantioselective catalytic transformation to chiral ammonium ions. The reaction is efficient (high conversion at 1 mol % catalyst loading, after <1 day at room temperature; er up to 10:90) and convenient to carry out. There is clearly room to improve the enantiomeric excess and to widen the substrate scope. However, we believe that the results are significant and provide an important stepping stone for further exploration.

## Data Availability

The data underlying this study are available in the published article and its Supporting Information.

## Supporting Information Available

Experimental procedures, optimization of conditions, racemization studies, NMR, HPLC, and crystallographic data (PDF)
