# Supplementary material for: Enantioselective Allylation of Stereogenic Nitrogen Centers
Source: Org Lett. 2023 Mar 7;25(10):1649–54. doi: 10.1021/acs.orglett.3c00195 (PMC10028698; doi:10.1021/acs.orglett.3c00195)
Supplement: Supplementary file 1 — ol3c00195_si_001.pdf [file ol3c00195_si_001.pdf]

Enantioselective Allylation of Stereogenic Nitrogen Centers

Snizhana Zaitseva, Alessandro Prescimone, and Valentin Köhler*

Department of Chemistry, University of Basel, Mattenstrasse 22, 4058 Basel

Corresponding Author: valentin.koehler@unibas.ch

Supporting Information

Table of Contents

General information **3**

Synthesis of substrates and standards **4**

Methylation of secondary amines *4*
General method [1] 4

Cross coupling reactions with bromo-substituted N-methyl tetrahydroisoquinolines [6] *7*
General Procedure A 7
General Procedure B 7
Synthesis of 5-(3,5-dimethylphenyl)-2-methyl-1,2,3,4-tetrahydroisoquinoline (**1o**) 9

Synthesis of 2-ethyl-1,2,3,4-tetrahydroisoquinoline (**1r**) *9*

Synthesis of N-benzyl-N,2-dimethylpropan-1-amine (**1s**) *10*

Preparation of allyl methyl carbonates [7] *10*
General method 10

Preparation of racemic allyl ammonium bromides *13*
General method 13

Preparation of enantioenriched ammonium salts *20*

Catalysis **23**

Analytical scale *24*

Preparative scale *25*
Work-up under addition of water 25
Formation of the Hofmann-elimination product 26
Work-up under addition of volatile basic buffer 27

Optimization of conditions *31*
Leaving group 31
Screening of solvents 32
with unsubstituted allyl acetate as the electrophile 32
with **2a** as the electrophile 33
Screening of various nucleophile and electrophile concentrations and ratios 35
Ligand screening 36

Substrate screening *41*
Electrophiles 41
Nucleophiles 42

Racemization studies *44*

Further experiments to investigate side product formation *46*
Isoprene formation 46
Effect of the leaving group on side product formation 48
Effect of water addition on conversion and isoprene formation 51
Solvolysis of the electrophile 53
Spontaneous formation of the chloromethylated ammonium ion in CH_2Cl_2 56

Equilibrium position of tertiary amine allylation in different solvents *57*

X-ray crystallographic data and determination of absolute configuration **59**

HPLC methods **61**

Calibration curves for HPLC-yield determination *63*

Response factors *64*

HPLC chromatograms **73**

Copies of ^{1}H and ^{13}C NMR spectra **98**

NMR spectra of the isolated from preparative reactions ammonium salts *206*

References **222**

General information

Chemicals were purchased from Sigma-Aldrich, Acros, Fluorochem, Strem, Enamine, Supelco, Combiblocks, Synthonix, BLDpharm and used as received.
NMR experiments were performed at 25°C if not indicated otherwise (MeOH calibration) on Bruker Avance III (400 MHz), Avance III HD (600 MHz), or Neo (500 MHz) NMR spectrometers. The 400 and 500 MHz instruments were equipped with direct dual channel, broadband probe-heads (BBFO SP), the 600 MHz instrument with a four channel inverse cryogenic He-cooled probe (QCI-F); all probes included self-shielded z-gradients. Chemical shifts were referenced to residual proton solvent peaks (^{1}H NMR: 3.31 ppm for CHD_2OD, 7.26 ppm for $CHCl_3$, 2.51 ppm for DMSO-d_5, 6.00 ppm for TCE-d_1;^{13}C NMR: 49.9 ppm for CD_3OD, 77.2 ppm for $CDCl_3$, 39.5 ppm for DMSO-d_6). Accepted abbreviations for multiplicities and descriptors are s, d, t, q, quint, m, dd, dt, td, br, other abbreviations for multiplicities used are: sext – sextet; non – nonet; qd – quartet of doublets; tsept – triplet of septets; ddt – doublet of doublets of triplets; dtt – doublet of triplets of triplets; br.s – broad singlet; br.t – broad triplet. Abbreviations for multiplicities in quotation marks refer to pseudo-splitting patterns, i.e., as they appear in the ^{1}H-NMR spectra.
Analytical HPLC measurements were performed on Agilent (or hp) machines equipped with modules from the 1100 and 1200 series and diode array detectors, if not indicated otherwise. Preparative HPLC-separations were performed on a Waters 4000 System equipped with a Waters 2487: Dual λ Absorbance Detector. HPLC-columns and conditions are indicated in a separate section. High resolution mass spectra (HRMS) spectra were recorded on a Bruker maxis 4G ESI-Q-TOF. LC-MS data were recorded on a Shimadzu prominence HPLC-system with an LCMS2020 detector.
Optical rotation was measured on Anton Paar MCP 100 and Jasco P-2000 instruments.

Synthesis of substrates and standards

Methylation of secondary amines

General method [1]

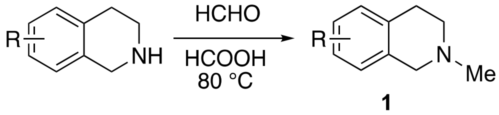

1

Formic acid (0.25 mL, 6.6 mmol) and formaldehyde (0.45 mL, 37% in H_2O, 6.0 mmol) were added to the corresponding amine (2.00 mmol) and the mixture was stirred in an oil bath at 80 °C for 2-3 hours. Completion of the reaction was monitored by TLC. After consumption of the starting material, the solution was diluted with 1M HCl (5-10 mL) and washed with TBME (2×5 mL). Subsequently, the pH was adjusted to 11 with solid or aqueous NaOH (2M), and the aqueous phase extracted with TBME (2×10 mL). The organic phase was washed with brine, dried over Na_2SO_4 and low boiling volatiles were removed at the rotary evaporator. The product was purified by distillation or column chromatography over silica.

*2-methyl-1,2,3,4-tetrahydroisoquinoline (**1a**)*

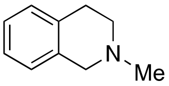

Light yellow liquid (12.6 g, 86% with respect to 10.0 mmol of the secondary amine), purified by vacuum distillation (45-47 °C, 0.18-0.20 mbar); ^{1}H NMR (500 MHz, $CDCl_3$) δ 7.15 – 7.07 (m, 3H), 7.05 – 6.97 (m, 1H), 3.59 (s, 2H), 2.93 (br.t, *J* = 6.0 Hz, 2H), 2.69 (t, *J* = 6.0 Hz, 2H), 2.46 (s, 3H). The NMR data were in accordance with literature reported values.[2]

*2,8-dimethyl-1,2,3,4-tetrahydroisoquinoline (**1b**)*

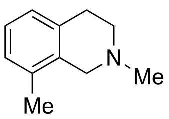

Yellow liquid (100 mg, 61%, with respect to 1.02 mmol of the secondary amine), purified by column chromatography over silica (CH_2Cl_2 : MeOH from 99:1 to 24:1); ^{1}H NMR (500 MHz, $CDCl_3$) δ 7.05 (m, 1H), 6.96 (m, 2H), 3.50 (s, 2H), 2.94 (br.t, *J* = 5.9 Hz, 2H), 2.68 (t, *J* = 5.9 Hz, 2H), 2.51 (s, 3H), 2.19 (s, 3H); ^{13}C NMR (126 MHz, $CDCl_3$) δ 134.5, 133.9, 133.4, 127.3, 126.5, 125.9, 56.2, 52.5, 46.6, 30.0, 18.7; HRMS (ESI) m/z: $[M + H]^+$ Calcd for $C_{11}H_{16}N$ 162.1278; Found 162.1279.

*2-methyl-8-(trifluoromethyl)-1,2,3,4-tetrahydroisoquinoline (**1c**)*

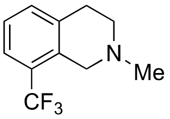

Yellow liquid (220 mg, 83%, with respect to 1.24 mmol of the secondary amine), the compound was not further purified; ^{1}H NMR (500 MHz, $CDCl_3$) δ 7.45 ('d', *J* = 7.6 Hz, 1H), 7.29 ('d', *J* = 7.6 Hz, 1H), 7.22 ('t', *J* = 7.6 Hz, 1H), 3.74 (s, 2H), 3.01 (t, *J* = 6.0 Hz, 2H), 2.72 (t, *J* = 6.0 Hz, 2H), 2.50 (s, 3H); ^{13}C NMR (126 MHz, $CDCl_3$) δ 135.8, 133.4 (q, *J* = 1.5 Hz), 132.8 (q, *J* = 1.2 Hz), 127.1 (q, *J* = 30.0 Hz), 126.0, 124.6 (q, *J* = 273.4 Hz), 123.6 (q, *J* = 5.9 Hz), 54.9 (q, *J* =

3.0 Hz), 52.2, 46.3, 29.7; ^{19}F NMR (470 MHz, $CDCl_3$) δ -61.11; HRMS (ESI) m/z: $[M + H]^+$ Calcd for $C_{11}H_{13}F_3N$ 216.0995; Found 216.0997.

2-methyl-1,2,3,4-tetrahydrobenzo[h]isoquinoline (1d)

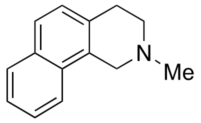

Yellow liquid (125 mg, 63%, with respect to 1.05 mmol of the secondary amine in the form of the hydrochloride salt). The hydrochloride salt of the amine was directly treated under Eschweiler-Clarke conditions. The crude product was purified by column chromatography over silica (CH_2Cl_2 : MeOH = 33:1); ^{1}H NMR (500 MHz, $CDCl_3$) δ 7.80 (m, 2H), 7.66 (d, *J* = 8.0 Hz, 1H), 7.50 (m, 1H), 7.44 (m, 1H), 7.24 (d, *J* = 8.4 Hz, 1H), 4.05 (s, 2H), 3.09 (t, *J* = 5.8 Hz, 2H), 2.84 (t, *J* = 5.8 Hz, 2H), 2.64 (s, 3H); ^{13}C NMR (126 MHz, $CDCl_3$) δ 132.1, 131.5, 130.7, 129.2, 128.7, 127.5, 126.5, 126.2, 125.0, 122.0, 55.3, 52.4, 46.5, 30.3; HRMS (ESI) m/z: $[M + H]^+$ Calcd for $C_{14}H_{16}N$ 198.1278; Found 198.1280.

8-bromo-2-methyl-1,2,3,4-tetrahydroisoquinoline (1e)

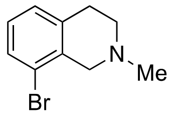

Pale yellow liquid (388 mg, 74%), the compound was not further purified; ^{1}H NMR (500 MHz, $CDCl_3$) δ 7.36 (dd, *J* = 7.7, 1.2 Hz, 1H), 7.07 (dd, *J* = 7.7, 1.2 Hz, 1H), 7.01 (t, *J* = 7.7 Hz, 1H), 3.56 (s, 2H), 2.93 (t, *J* = 5.9 Hz, 2H), 2.67 (t, *J* = 5.9 Hz, 2H), 2.51 (s, 3H); ^{13}C NMR (126 MHz, $CDCl_3$) δ 136.9, 134.4, 129.9, 127.9, 127.4, 122.8, 58.6, 52.3, 46.2, 29.9; HRMS (ESI) m/z: $[M + H]^+$ Calcd for $C_{10}H_{13}BrN$ 226.0226; Found 226.0227.

5-bromo-2-methyl-1,2,3,4-tetrahydroisoquinoline (1f)

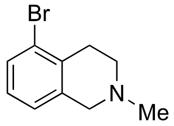

Pale yellow liquid (3.70 g, 71%, with respect to 20.0 mmol of the secondary amine), the compound was not further purified; ^{1}H NMR (500 MHz, $CDCl_3$) δ 7.41 – 7.37 (m, 1H), 7.02 – 6.95 (m, 2H), 3.56 (br.s, 2H), 2.87 (t, *J* = 6.1 Hz, 2H), 2.70 (t, *J* = 6.1 Hz, 2H), 2.45 (br.s, 3H); ^{13}C NMR (126 MHz, $CDCl_3$) δ 137.4, 133.9, 130.3, 127.1, 125.7, 125.4, 58.2, 53.1, 45.9, 30.5; HRMS (ESI) m/z: $[M + H]^+$ Calcd for $C_{10}H_{13}BrN$ 226.0226; Found 226.0224.

6,7-dimethoxy-2-methyl-1,2,3,4-tetrahydroisoquinoline (1g)

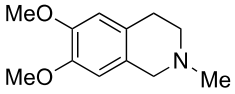

White solid (1.70 g, 86%, with respect to 9.60 mmol of the secondary amine), the compound was not further purified; ^{1}H NMR (500 MHz, $CDCl_3$) δ 6.60 (s, 1H), 6.51 (s, 1H), 3.84 (s, 3H), 3.83 (s, 3H), 3.52 (s, 2H), 2.85 (t, *J* = 6.0 Hz, 2H), 2.68 (t, *J* = 6.0 Hz, 2H), 2.46 (s, 3H). ^{1}H NMR data were in accordance with reported values.[3]

*2-methyl-7-nitro-1,2,3,4-tetrahydroisoquinoline (**1h**)*

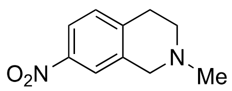

Red solid (290 mg, 75%), the compound was not further purified; ^{1}H NMR (500 MHz, $CDCl_3$) δ 7.98 (dd, *J* = 8.4, 2.4 Hz, 1H), 7.91 (d, *J* = 2.4 Hz, 1H), 7.26 (d, *J* = 8.4 Hz, 1H), 3.66 (s, 2H), 3.02 (t, *J* = 6.0 Hz, 2H), 2.74 (t, *J* = 6.0 Hz, 2H), 2.50 (s, 3H); ^{13}C NMR (126 MHz, $CDCl_3$) δ 146.2, 142.1, 136.4, 129.7, 121.8, 121.3, 57.7, 52.3, 46.0, 29.6; HRMS (ESI) m/z: $[M + H]^+$ Calcd for $C_{10}H_{13}N_2O_2$ 193.0972; Found 193.0973.

*2,4,4-trimethyl-1,2,3,4-tetrahydroisoquinoline (**1i**)*

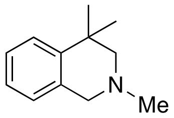

Light yellow liquid (400 mg, 58%, with respect to 4.30 mmol of the secondary amine in the form of the hydrochloride salt). The hydrochloride salt of the amine was directly treated with formaldehyde and formic acid as described in the general procedure. The crude material was purified by column chromatography over silica (CH_2Cl_2 : MeOH = 97 : 3); ^{1}H NMR (500 MHz, $CDCl_3$) δ 7.31 (d, *J* = 7.6 Hz, 1H), 7.17 (t, *J* = 7.5 Hz, 1H), 7.09 (td, *J* = 7.5 Hz, *J* = 1.3 Hz, 1H), 6.99 (d, *J* = 7.6 Hz, 1H), 3.53 (s, 2H), 2.41 (s, 3H), 2.39 (s, 2H), 1.31 (s, 6H); ^{13}C NMR (126 MHz, $CDCl_3$) δ 143.5, 134.1, 126.5, 126.3, 126.1, 125.5, 67.8, 59.5, 46.7, 35.4, 30.0; HRMS (ESI) m/z: $[M + H]^+$ Calcd for $C_{12}H_{18}N$ 176.1434; Found 176.1436.

*2-methyl-2,3,4,5-tetrahydro-1H-benzo[c]azepine (**1j**)*

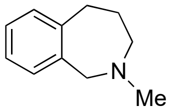

Light yellow liquid (203 mg, 63%), purified by column chromatography over silica (CH_2Cl_2 : MeOH = 9:1 + 0.5% Et_3N); ^{1}H NMR (500 MHz, $CDCl_3$) δ 7.19 – 7.08 (m, 4H), 3.81 (s, 2H), 3.09 – 2.94 (m, 2H), 2.93 – 2.81 (m, 2H), 2.31 (s, 3H), 1.83 – 1.69 (m, 2H). ^{1}H NMR data were in accordance with reported values.[4]

*2-(benzyl(methyl)amino)ethan-1-ol (**1p**)*

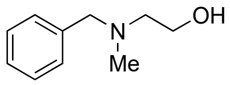

Yellow liquid (700 mg, 64%, with respect to 6.61 mmol of the secondary amine), purified by column chromatography over silica (CH_2Cl_2 : MeOH = 2:1); ^{1}H NMR (500 MHz, $CDCl_3$) δ 7.34 – 7.24 (m, 5H), 3.62 (t, *J* = 5.3 Hz, 2H), 3.57 (s, 2H), 2.60 (t, *J* = 5.3 Hz, 2H), 2.23 (s, 3H); ^{13}C NMR (126 MHz, $CDCl_3$) δ 138.6, 129.1 (2C), 128.5 (2C), 127.3, 62.4, 58.5, 58.5, 41.7; HRMS (ESI) m/z: $[M + H]^+$ Calcd for $C_{10}H_{16}NO$ 166.1226; Found 166.1226.

*1-methyl-3,3-diphenylpyrrolidine (**1q**)*

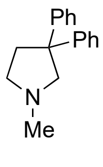

Yellow liquid (423 mg, 68%, with respect to 2.61 mmol of the secondary amine[5]; purified by column chromatography over silica (CH_2Cl_2 : MeOH = 10:1); ^{1}H NMR (500 MHz, $CDCl_3$) δ 7.30 – 7.25 (m, 8H), 7.19 – 7.14 (m, 2H), 3.24 (br.s, 2H), 2.83 (t, J = 7.2 Hz, 2H), 2.60 (t, J = 7.2 Hz, 2H), 2.43 (s, 3H); ^{13}C NMR (126 MHz, $CDCl_3$) δ 149.1, 128.3 (4C), 127.3 (4C), 125.9, 68.8, 55.6, 54.8, 43.1, 39.2; HRMS (ESI) m/z: $[M + H]^+$ Calcd for $C_{17}H_{20}N$ 238.1590; Found 238.1594.

Cross coupling reactions with bromo-substituted N-methyl tetrahydroisoquinolines [6]

General Procedure A

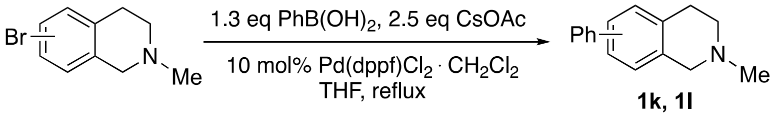

Phenylboronic acid (210 mg, 1.73 mmol), cesium acetate (640 mg, 3.33 mmol) and $Pd(dppf)Cl_2 \cdot CH_2Cl_2$ (108 mg, 132 µmol, 10 mol%) were added to a solution of the bromo-2-methyl-1,2,3,4-tetrahydroisoquinoline (300 mg, 1.33 mmol) in THF (9 mL) and the reaction mixture was heated to reflux in an oil bath overnight. Subsequently, volatiles were evaporated and the crude material was dissolved in 2M HCl (100 mL) and washed with TBME (2×30 mL). The aqueous layer was adjusted to pH 11 with solid NaOH and extracted with TBME (2×75 mL). The combined organic phases were washed with a saturated aqueous solution of the disodium salt of EDTA (1×50 mL), brine (1×50 mL), dried over Na_2SO_4 and volatiles were removed under reduced pressure on a rotary evaporator. The product was purified by column chromatography over silica (CH_2Cl_2 : MeOH = 30:1).

General Procedure B

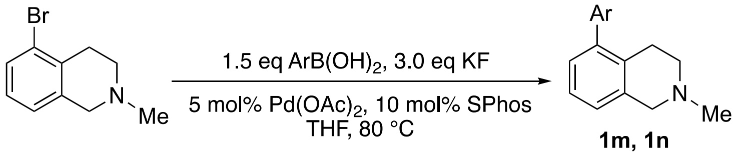

The corresponding tolylboronic acid (306 mg, 2.25 mmol), potassium fluoride (260 mg, 4.48 mmol), $Pd(OAc)_2$ (17 mg, 76 µmol, 5.0 mol%) and S-Phos ligand (62 mg, 0.15 mmol, 10 mol%) were weighed into a Schlenk flask and transferred to the glovebox. THF (2.0 mL) was added; the suspension was stirred for 5 min and 8-bromo-2-methyl-1,2,3,4-tetrahydroisoquinoline (340 mg, 1.50 mmol) was added. The flask was tightly closed, removed from the glovebox and the mixture was heated in an oil bath at 80 °C for 18 hours. The reaction mixture was diluted with TBME (50 mL) and 1M NaOH (10 mL) and filtered through 1-2 cm of celite. The organic layer was separated and the aqueous layer was extracted with TBME (1×30 mL). The combined organic layers were washed with brine (1×20 mL), dried over Na_2SO_4 and volatiles removed under reduced pressure on a rotary evaporator. The product was purified by column chromatography over silica (CH_2Cl_2 : MeOH = 30:1).

2-methyl-8-phenyl-1,2,3,4-tetrahydroisoquinoline (1k)

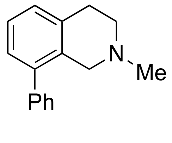

Prepared according to **procedure A**. Pale yellow viscous oil (190 mg, 64%); ^{1}H NMR (500 MHz, $CDCl_3$) δ 7.43 – 7.38 (m, 2H), 7.37 – 7.33 (m, 1H), 7.30 – 7.27 (m, 2H), 7.20 (t, *J* = 7.6 Hz, 1H), 7.13 (d, *J* = 7.5 Hz, 1H), 7.03 (d, *J* = 7.5 Hz, 1H), 3.43 (s, 2H), 3.03 (t, *J* = 6.1 Hz, 2H), 2.72 (t, *J* = 6.1 Hz, 2H), 2.38 (s, 3H); ^{13}C NMR (126 MHz, $CDCl_3$) δ 141.2, 140.5, 134.2, 132.3, 129.1, 128.3, 128.1, 127.4, 127.1, 126.1, 57.2, 52.9, 46.4, 29.8; HRMS (ESI) m/z: $[M + H]^+$ Calcd for $C_{16}H_{18}N$ 224.1434; Found 224.1437.

2-methyl-5-phenyl-1,2,3,4-tetrahydroisoquinoline (1l)

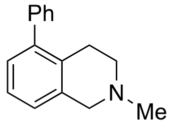

Prepared according to **procedure A**. Brown solid (920 mg, 94%, with respect to 4.42 mmol of the starting amine); ^{1}H NMR (500 MHz, $CDCl_3$) δ 7.43 – 7.38 (m, 2H), 7.36 – 7.32 (m, 1H), 7.32 – 7.28 (m, 2H), 7.20 (t, *J* = 7.5 Hz, 1H), 7.08 (dd, *J* = 7.5, 1.4 Hz, 1H), 7.05 (dd, *J* = 7.6, 1.4 Hz, 1H), 3.69 (br.s, 2H), 2.78 (t, *J* = 5.9 Hz, 2H), 2.63 (t, *J* = 5.9 Hz, 2H), 2.47 (s, 3H); ^{13}C NMR (126 MHz, $CDCl_3$) δ 142.1, 141.6, 134.9, 131.5, 129.2, 128.2, 127.8, 127.0, 125.8, 125.7, 58.4, 53.1, 46.0, 28.5; HRMS (ESI) m/z: $[M + H]^+$ Calcd for $C_{16}H_{18}N$ 224.1434; Found 224.1437.

2-methyl-5-(m-tolyl)-1,2,3,4-tetrahydroisoquinoline (1m)

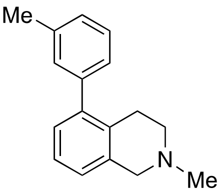

Prepared according to **procedure B**. Pale yellow oil (316 mg, 89%, with respect to 2.00 mmol of the starting amine); ^{1}H NMR (500 MHz, $CDCl_3$) δ 7.29 (t, *J* = 7.5 Hz, 1H), 7.20 – 7.14 (m, 2H), 7.11 – 7.02 (m, 4H), 3.66 (br.s, 2H), 2.77 (t, *J* = 5.9 Hz, 2H), 2.60 (t, *J* = 5.9 Hz, 2H), 2.46 (s, 3H), 2.38 (s, 3H); ^{13}C NMR (126 MHz, $CDCl_3$) δ 142.2, 141.6, 137.8, 135.2, 131.6, 130.0, 128.1, 127.7, 127.7, 126.2, 125.7, 125.6, 58.7, 53.3, 46.2, 28.7, 21.6; HRMS (ESI) m/z: $[M + H]^+$ Calcd for $C_{17}H_{20}N$ 238.1590; Found 238.1592.

2-methyl-5-(p-tolyl)-1,2,3,4-tetrahydroisoquinoline (1n)

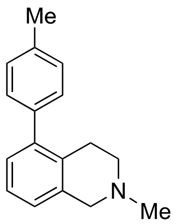

Prepared according to **procedure B**. Pale yellow liquid (106 mg, 81%, with respect to 54.9 μmol of the starting amine); ^{1}H NMR (500 MHz, $CDCl_3$) δ 7.23 – 7.15 (m, 5H), 7.06 (dd, *J* = 7.5, 1.4 Hz, 1H), 7.03 (dd, *J* = 7.6, 1.3 Hz, 1H), 3.65 (s, 1H), 2.77 (t, *J* = 5.9 Hz, 2H), 2.59 (t, *J* = 5.9 Hz, 2H), 2.45 (s, 3H), 2.40 (s, 3H); ^{13}C NMR (126 MHz, $CDCl_3$) δ 142.0, 138.7, 136.6, 135.2, 131.7, 129.1 (2C), 128.9 (2C), 127.8, 125.7, 125.7, 58.7, 53.3, 46.2, 28.8, 21.3; HRMS (ESI) m/z: $[M + H]^+$ Calcd for $C_{17}H_{20}N$ 238.1590; Found 238.1594.

Synthesis of 5-(3,5-dimethylphenyl)-2-methyl-1,2,3,4-tetrahydroisoquinoline (1o)

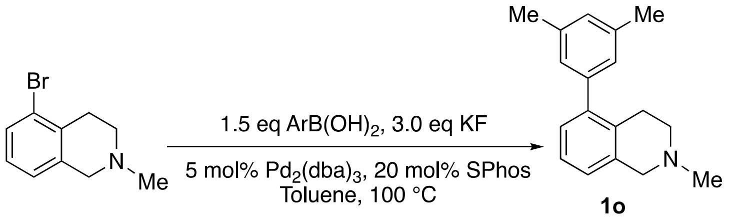

3,5-Dimethylphenylboronic acid (133 mg, 0.887 mmol) and potassium fluoride (103 mg, 1.77 mmol) were weighed into a Schlenk flask and transferred to the glovebox. $Pd_2(dba)_3$ (27 mg, 29 μmol, 4.9 mol%) and SPhos ligand (48 mg, 0.12 mmol, 20 mol%) were weighed into a separate vial in the glovebox, toluene (1.2 mL) was added and the mixture agitated for 15 minutes before it was transferred to the Schlenk flask with arylboronic acid and KF. 8-bromo-2-methyl-1,2,3,4-tetrahydroisoquinoline (134 mg, 0.593 mmol) was added. The flask was tightly closed, removed from the glovebox and the mixture was heated in an oil bath at 100 °C for 18 hours. The reaction mixture was diluted with TBME (50 mL) and 1M NaOH (10 mL) and filtered through a thin plug of celite (1-2 cm). The organic layer was separated and the aqueous layer was extracted with TBME (1×30 mL). The combined organic layers were washed with brine (1×20 mL), dried over Na_2SO_4 and volatiles removed under reduced pressure on a rotary evaporator. The product was purified by column chromatography over silica (CH_2Cl_2 : MeOH = 30:1).

Colorless oil (80 mg, 54%); ^{1}H NMR (500 MHz, $CDCl_3$) δ 7.17 (t, J = 7.6 Hz, 1H), 7.07 – 7.00 (m, 2H), 6.99 – 6.97 (m, 1H), 6.92 – 6.90 (m, 2H), 3.67 (br.s, 2H), 2.78 (t, J = 5.9 Hz, 2H), 2.61 (t, J = 5.9 Hz, 2H), 2.46 (s, 3H), 2.35 (d, J = 0.7 Hz, 6H); ^{13}C NMR (126 MHz, $CDCl_3$) δ 142.3, 141.6, 137.7, 135.0, 131.6, 128.6, 127.7, 127.0, 125.6, 125.6, 58.6, 53.3, 46.2, 28.7, 21.5; HRMS (ESI) m/z: $[M + H]^+$ Calcd for $C_{18}H_{22}N$ 252.1747; Found 252. 1750.

Synthesis of 2-ethyl-1,2,3,4-tetrahydroisoquinoline (1r)

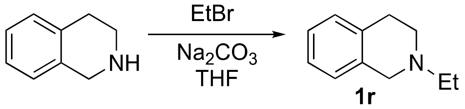

Bromoethane (1.13 mL, 15.0 mmol) was added dropwise to a precooled (ice-bath) solution of 1,2,3,4-tetrahydroisoquinoline (1.35 g, 10.0 mmol) in THF (50 mL) containing sodium carbonate (2.12 g, 20.0 mmol). The reaction mixture was left stirring at room temperature for 2 days before volatiles were removed under reduced pressure at the rotary evaporator (150 rpm, 39 °C water bath). The crude material was dissolved in 200 mL of TBME, washed with water (2×25 mL), brine (1×25 mL), dried over Na_2SO_4 and volatiles removed under reduced pressure on a rotary evaporator. The product was purified by column chromatography over silica in CH_2Cl_2 : MeOH (99:1, 0.5% Et_3N).

Yellow liquid (0.98 g, 61%); ^{1}H NMR (500 MHz, $CDCl_3$) δ 7.16 – 7.07 (m, 3H), 7.06 – 6.99 (m, 1H), 3.64 (br.s, 2H), 2.93 (t, J = 6.0 Hz, 2H), 2.75 (t, J = 6.0 Hz, 2H), 2.60 (q, J = 7.2 Hz, 2H), 1.20 (t, J = 7.2 Hz, 3H); ^{13}C NMR (126 MHz, $CDCl_3$) δ 135.0, 134.5, 128.8, 126.7, 126.2, 125.7, 55.9, 52.3, 50.8, 29.3, 12.5; HRMS (ESI) m/z: $[M + H]^+$ Calcd for $C_{11}H_{16}N$ 162.1278; Found 162.1280.

Synthesis of *N*-benzyl-*N*,2-dimethylpropan-1-amine (1s)

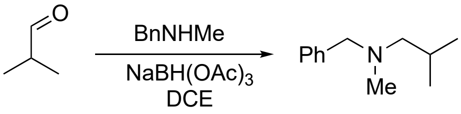

1s

Isobutyraldehyde (1.00 mL, 11.0 mmol), DCE (30 mL) and *N*-methyl-1-phenylmethanamine (1.50 mL, 12.0 mmol) were placed in the flask under a nitrogen atmosphere and sodium triacetoxyborohydride (3.26 g, 15.4 mmol) was added to the solution under a stream of nitrogen. The mixture was left stirring at room temperature overnight. 100 mL of saturated sodium bicarbonate solution were added and the reaction mixture was extracted with ethyl acetate (3×100 mL). The combined organic phases were washed with brine (1×70 mL), dried over Na_2SO_4 and volatiles removed under reduced pressure on a rotary evaporator. The product was purified by column chromatography over silica (CH_2Cl_2 : MeOH = 30:1).

Yellow liquid (517 mg, 27%); ^{1}H NMR (500 MHz, $CDCl_3$) δ 7.35 – 7.27 (m, 4H), 7.26 – 7.21 (m, 1H), 3.46 (s, 2H), 2.16 (s, 3H), 2.11 (d, *J* = 7.3 Hz, 2H), 1.82 ('non', *J* = 6.8 Hz, 1H), 0.92 (s, 3H), 0.90 (s, 3H); ^{13}C NMR (126 MHz, $CDCl_3$) δ 139.8, 129.1 (2C), 128.2 (2C), 126.9, 66.2, 62.8, 42.8, 26.3, 21.0 (2C); HRMS (ESI) m/z: $[M + H]^+$ Calcd for $C_{12}H_{20}N$ 178.1590; Found 178.1591.

Preparation of allyl methyl carbonates [7]

General method

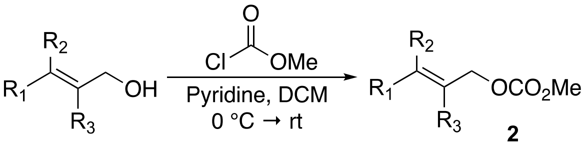

2

Pyridine (2.40 mL, 30.0 mmol) was added to a solution of the corresponding allyl alcohol (10.0 mmol) in 25 mL of CH_2Cl_2 and the mixture cooled in an ice bath. Methyl chloroformate (1.55 mL, 20.0 mmol) was added dropwise and the resulting mixture was stirred at room temperature overnight. 50 mL of brine was added and the mixture was extracted with TBME (2×75 mL). The organic layers were combined, washed with 1M HCl (1×50 mL), brine (1×50 mL), dried over Na_2SO_4 and concentrated at the rotary evaporator. The crude product was purified by column chromatography over silica (cyclohexane : ethyl acetate from 80:1 to 9:1).

Methyl (3-methylbut-2-en-1-yl) carbonate (2a)

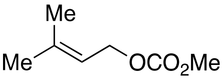

Colorless liquid (4.06 g, 94%, with respect to 30.0 mmol of the allyl alcohol), purified by column chromatography over silica (cyclohexane : ethyl acetate 20:1); ^{1}H NMR (500 MHz, $CDCl_3$) δ 5.37 ('tsept', *J* = 7.3, 1.4 Hz, 1H), 4.63 (d, *J* = 7.3 Hz, 2H), 3.77 (d, *J* = 1.4 Hz, 3H), 1.76 (s, 3H), 1.72 (s, 3H). NMR data were in accordance with reported values.[8]

Allyl methyl carbonate (2b)

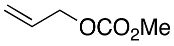

Colorless liquid (2.34 g, 67%, with respect to 30.1 mmol of the allyl alcohol), filtered through silica with CH_2Cl_2; ^1H NMR (500 MHz, $CDCl_3$) δ 5.94 (ddt, *J* = 17.1, 10.4, 5.8 Hz, 1H), 5.36 (dd, *J* = 17.1, 1.4 Hz, 1H), 5.27 (m, 1H), 4.63 (dt, *J* = 5.8, 1.3 Hz, 2H), 3.80 (s, 3H). The ^1H NMR data were in accordance with reported values.[9]

*3-ethylpent-2-en-1-yl methyl carbonate (**2c**)*

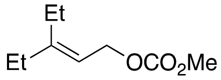

Colorless liquid (1.46 g, 85%), purified by column chromatography over silica (cyclohexane : ethyl acetate = 20:1); ^1H NMR (500 MHz, $CDCl_3$) δ 5.31 (t, *J* = 7.2 Hz, 1H), 4.67 (d, *J* = 7.2 Hz, 2H), 3.78 (s, 3H), 2.12 (q, *J* = 7.7 Hz, 2H), 2.08 (q, *J* = 7.4 Hz, 2H), 1.01 (t, *J* = 7.4 Hz, 3H), 0.99 (t, *J* = 7.7 Hz, 3H); ^{13}C NMR (126 MHz, $CDCl_3$) δ 156.0, 150.8, 116.0, 64.7, 54.8, 29.2, 23.9, 13.7, 12.4; HRMS (ESI) m/z: $[M + Na]^+$ Calcd for $C_9H_{16}O_3Na$ 195.0992; Found 195.0988.

*3,3-diphenylallyl methyl carbonate (**2d**)*

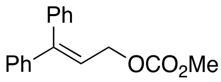

White solid (1.62 g, 60%), purified by column chromatography over silica (cyclohexane : ethyl acetate = 9:1); ^1H NMR (500 MHz, $CDCl_3$) δ 7.42 – 7.32 (m, 3H), 7.30 – 7.24 (m, 5H), 7.22 – 7.16 (m, 2H), 6.21 (t, *J* = 7.1 Hz, 1H), 4.70 (d, *J* = 7.1 Hz, 2H), 3.79 (s, 3H); ^{13}C NMR (126 MHz, $CDCl_3$) δ 155.9, 147.2, 141.5, 138.6, 129.8, 128.5, 128.4, 128.1, 128.0, 127.9, 121.7, 66.1, 54.9; HRMS (ESI) m/z: $[M + Na]^+$ Calcd for $C_{17}H_{16}O_3Na$ 291.0992; Found 291.0989.

*Cinnamyl methyl carbonate (**2e**)*

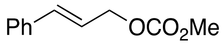

Colorless liquid (0.71 g, 37%), purified by column chromatography over silica (cyclohexane : ethyl acetate = 20:1); ^1H NMR (500 MHz, $CDCl_3$) δ 7.39 (d, *J* = 7.5 Hz, 2H), 7.33 (t, *J* = 7.5 Hz, 2H), 7.27 (t, *J* = 7.4 Hz, 1H), 6.69 (dd, *J* = 15.9, 1.4 Hz, 1H), 6.30 (dt, *J* = 15.9, 6.5 Hz, 1H), 4.80 (dd, *J* = 6.5, 1.4 Hz, 2H), 3.81 (s, 3H). ^1H NMR data were in accordance with reported values.[10]

*Methyl (2-methylallyl) carbonate (**2f**)*

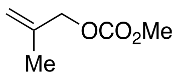

Colorless liquid (2.02 g, 52%, with respect to 29.9 mmol of the allyl alcohol); ^1H NMR (600 MHz, $CDCl_3$) δ 5.02 (br.s, 1H), 4.95 (br.s, 1H), 4.55 (s, 2H), 3.80 (s, 3H), 1.77 (br.s, 3H). ^1H NMR data were in accordance with reported values.[11]

*(E)-hex-2-en-1-yl methyl carbonate (**2g**)*

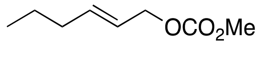

Colorless liquid (1.67 g, 53%, with respect to 20.0 mmol of the allyl alcohol), purified by column chromatography over silica (cyclohexane : ethyl acetate = 80:1); ^1H NMR (500 MHz, $CDCl_3$)

δ 5.81 (dtt, J = 15.4, 6.6, 1.1 Hz, 1H), 5.58 (dtt, J = 15.4, 6.6, 1.4 Hz, 1H), 4.57 (dd, J = 6.6, 1.0 Hz, 2H), 3.78 (s, 3H), 2.04 (q, J = 7.2 Hz, 2H), 1.41 ('sext', J = 7.3 Hz, 2H), 0.90 (t, J = 7.4 Hz, 3H). ^{1}H NMR data were in accordance with reported values.[12]

Allyl electrophiles with leaving groups other than methyl carbonates were synthesized from the corresponding allyl alcohols according to the previously reported procedures.[13]

Allyl alcohols 3,3-diphenylprop-2-en-1-ol[14] and 3-ethylpent-2-en-1-ol[15] and allyl bromides 3-bromoprop-1-ene-1,1-diyl)dibenzene[16]; 1-bromo-3-ethylpent-2-ene[17] and (*E*)-1-bromohex-2-ene[18] were synthesized according to previously reported procedures.

Preparation of racemic allyl ammonium bromides

General method

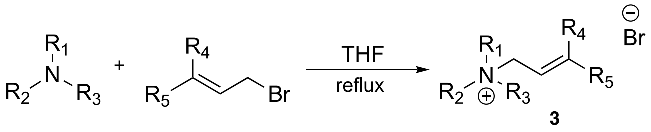

The corresponding allyl bromide (1.1 eq) was added dropwise to a solution of the corresponding tertiary amine (0.2 mmol) in THF (1 mL). If after 1 hour of stirring at room temperature no precipitation was observed, the reaction mixture was heated to reflux in an oil bath for 1-5 h; in case of early precipitate formation, the reaction was stirred for 2 hours at room temperature before filtration. The formed precipitate was filtered off, washed with THF and diethyl ether and dried under vacuum. In the cases where no solid was formed even after prolonged heating, volatiles were evaporated and the product recrystallized from CH_2Cl_2-THF.

*rac-2-methyl-2-(3-methylbut-2-en-1-yl)-1,2,3,4-tetrahydroisoquinolin-2-ium bromide ([**3aa**]Br)*

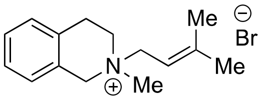

White solid (2.39 g, 81%, 10.0 mmol scale); ^{1}H NMR (500 MHz, $CDCl_3$) δ 7.32 (t, *J* = 7.4 Hz, 1H), 7.27 (d, *J* = 7.6 Hz, 1H), 7.24 (t, *J* = 7.6 Hz, 1H), 7.14 (d, *J* = 7.6 Hz, 1H), 5.41 ('br.t', *J* = 8.2 Hz, 1H), 4.79 (AB spin system, $δ_A$ = 4.82, $δ_B$ = 4.76, J_{AB} = 15.1 Hz, 2H), 4.51 (dd, *J* = 13.4, 8.1 Hz, 1H), 4.43 (dd, *J* = 13.4, 8.2 Hz, 1H), 4.21 – 4.14 (m, 1H), 4.05 – 3.97 (m, 1H), 3.39 (s, 3H), 3.29 – 3.17 (m, 2H), 1.89 (s, 3H), 1.86 (s, 3H); ^{1}H NMR (500 MHz, DMSO-d_6) δ 7.37 – 7.27 (m, 3H), 7.21 (d, *J* = 7.3 Hz, 1H), 5.49 (m, 1H), 4.54 (AB spin system, $δ_A$ = 4.59, $δ_A$ = 4.50, *J* = 15.4 Hz, 2H), 4.06 – 3.95 (m, 2H), 3.71 – 3.64 (m, 2H), 3.18 (t, *J* = 6.6 Hz, 2H), 2.97 (s, 3H), 1.86 (br.s, 2H), 1.72 (d, *J* = 1.3 Hz, 3H); ^{13}C NMR (101 MHz, $CDCl_3$) δ 149.5, 129.0, 128.9, 128.9, 127.7, 127.6, 126.5, 110.4, 60.8, 60.6, 56.9, 47.2, 26.6, 24.0, 19.6.; HRMS (ESI) m/z: $[M]^+$ Calcd for $C_{15}H_{22}N$ 216.1747; Found 216.1750; HPLC (OX-H, ethanol/n-heptane/Et_3N/CF_3CO_2H = 20/80/0.5/0.3, flow rate = 1.0 mL/min, l = 220 nm) tR = 26.0 min, 27.4 min.

*rac-2-allyl-2-methyl-1,2,3,4-tetrahydroisoquinolin-2-ium bromide ([**3ab**]Br)*

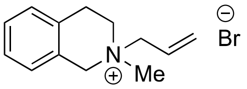

White solid (2.52 g, 94%, 10.0 mmol scale); ^{1}H NMR (500 MHz, $CDCl_3$) δ 7.33 (td, *J* = 7.5, 1.4 Hz, 1H), 7.28 (dd, *J* = 7.5, 1.4 Hz, 1H), 7.24 (d, *J* = 7.6 Hz, 1H), 7.15 (d, *J* = 7.6 Hz, 1H), 6.09 ('ddt', *J* = 17.0, 10.0, 7.4 Hz, 1H), 5.87 (m,1H), 5.78 (m, *J* = 10.0, 0.8 Hz, 1H), 4.85 (AB spin system, $δ_A$ = 4.89, $δ_B$ = 4.81, J_{AB} = 15.2 Hz, 2H), 4.59 (d, *J* = 7.4 Hz, 2H), 4.24 – 4.16 (m, 1H), 4.06 – 3.99 (m, 1H), 3.45 (s, 3H), 3.30 – 3.17 (m, 2H); HRMS (ESI) m/z: $[M]^+$ Calcd for $C_{13}H_{18}N$ 188.1434; Found 188.1434; HPLC (OX-H, ethanol/n-heptane/Et_3N/CF_3CO_2H = 20/80/0.5/0.3, flow rate = 1.0 mL/min, l = 220 nm) tR = 24.2 min, 26.4 min. ^{1}H NMR data were in accordance with reported values.[19]

rac-2-(3-ethylpent-2-en-1-yl)-2-methyl-1,2,3,4-tetrahydroisoquinolin-2-ium bromide ([3ac]Br)

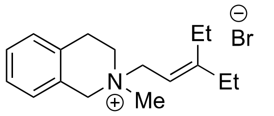

White solid (43 mg, 67%); ^{1}H NMR (500 MHz, $CDCl_3$) δ 7.33 (t, *J* = 7.4 Hz, 1H), 7.28 (d, *J* = 7.4 Hz, 1H), 7.24 (d, *J* = 7.6 Hz, 1H), 7.14 (d, *J* = 7.6 Hz, 1H), 5.34 (t, *J* = 8.1 Hz, 1H), 4.82 – 4.75 (m, 2H), 4.49 (dd, *J* = 13.5, 8.1 Hz, 1H), 4.43 (dd, *J* = 13.5, 8.2 Hz, 1H), 4.23 – 4.16 (m, 1H), 4.04 – 3.97 (m, 1H), 3.41 (s, 3H), 3.30 – 3.18 (m, 2H), 2.31 – 2.16 (m, 4H), 1.07 (t, *J* = 7.4 Hz, 3H), 1.00 (t, *J* = 7.5 Hz, 3H); ^{13}C NMR (126 MHz, $CDCl_3$) δ 159.7, 129.0, 129.0 (2C), 127.8, 127.6, 126.4, 108.0, 60.9, 59.9, 57.0, 47.5, 29.6, 24.0 (2C), 13.1, 12.6; HRMS (ESI) m/z: $[M]^+$ Calcd for $C_{17}H_{26}N$ 244.2060; Found 244.2059; HPLC (OX-H, ethanol/n-heptane/Et_3N/CF_3CO_2H = 20/80/0.5/0.3, flow rate = 1.0 mL/min, l = 220 nm) tR = 22.9 min, 24.7 min.

rac-2-(3,3-diphenylallyl)-2-methyl-1,2,3,4-tetrahydroisoquinolin-2-ium bromide ([3ad]Br)

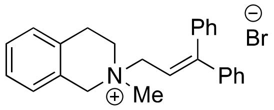

White solid (66 mg, 78%); ^{1}H NMR (500 MHz, $CDCl_3$) δ 7.38 – 7.27 (m, 9H), 7.23 (t, *J* = 7.5 Hz, 1H), 7.11 (d, *J* = 7.5 Hz, 1H), 7.09 – 7.03 (m, 3H), 6.36 (t, *J* = 7.7 Hz, 1H), 4.72 (AB spin system, $δ_A$ = 4.78, $δ_B$ = 4.65, J_{AB} = 15.2 Hz, 2H), 4.56 (dd, *J* = 13.4, 7.8 Hz, 1H), 4.50 (dd, *J* = 13.4, 7.7 Hz, 1H), 4.21 – 4.14 (m, 1H), 3.92 – 3.84 (m, 1H), 3.45 (s, 3H), 3.16 (dt, *J* = 18.4, 6.8 Hz, 1H), 3.02 (dt, *J* = 18.4, 6.4 Hz, 1H); ^{13}C NMR (126 MHz, $CDCl_3$) δ 153.9, 140.0, 137.2, 129.3, 129.2, 128.9 (2C), 128.8, 128.8, 128.6 (2C), 127.9, 127.7, 127.5, 125.9, 112.7, 61.8, 60.7, 57.7, 48.6, 23.8; HRMS (ESI) m/z: $[M]^+$ Calcd for $C_{25}H_{26}N$ 340.2060; Found 340.2059.

rac-2-cinnamyl-2-methyl-1,2,3,4-tetrahydroisoquinolin-2-ium bromide ([3ae]Br)

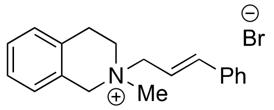

Light brown solid (59 mg, 86%); refluxed (oil bath) for 2 h, volatiles removed under reduced pressure and oil triturated with Et_2O; ^{1}H NMR (600 MHz, $CDCl_3$) δ 7.48 – 7.44 (m, 2H), 7.35 – 7.31 (m, 3H), 7.29 (t, *J* = 7.4 Hz, 1H), 7.25 – 7.20 (m, 2H), 7.13 (br.s, 1H), 7.11 (t, *J* = 6.8 Hz, 1H), 6.37 (dt, *J* = 15.4, 7.7 Hz, 1H), 4.96 (d, *J* = 15.2 Hz, 1H), 4.85 (d, *J* = 7.7 Hz, 2H), 4.79 (d, *J* = 15.2 Hz, 1H), 4.23 – 4.17 (m, 1H), 4.09 – 4.03 (m, 1H), 3.43 (s, 3H), 3.28 – 3.17 (m, 2H); ^{13}C NMR (126 MHz, $CDCl_3$) δ 144.4, 134.8, 129.6, 129.0, 129.0, 128.9, 128.9, 127.9, 127.6, 127.4, 126.2, 113.9, 65.3, 61.3, 57.2, 47.0, 24.0; HRMS (ESI) m/z: $[M]^+$ Calcd for $C_{19}H_{22}N$ 264.1747; Found 264.1750; HPLC (OX-H, ethanol/n-heptane/Et_3N/CF_3CO_2H = 20/80/0.5/0.3, flow rate = 1.0 mL/min, l = 220 nm) tR = 28.3 min, 31.1 min.

rac-2-methyl-2-(2-methylallyl)-1,2,3,4-tetrahydroisoquinolin-2-ium bromide ([3af]Br)

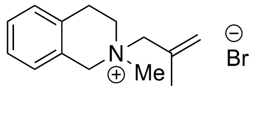

White solid (45 mg, 80%); ^{1}H NMR (500 MHz, $CDCl_3$) δ 7.34 (t, *J* = 7.5 Hz, 1H), 7.29 (t, *J* = 7.5 Hz, 1H), 7.26 – 7.25 (m, 1H), 7.14 (d, *J* = 7.6 Hz, 1H), 5.58 (br.s, 1H), 5.54 (s, 1H), 4.81 (AB spin system, $δ_A$ = 4.86, $δ_B$ = 4.77, J_{AB} = 15.1 Hz, 2H), 4.54 (AB spin system, $δ_A$ = 4.61, $δ_B$

= 4.48, J_{AB} = 12.7 Hz, 2H), 4.27 – 4.10 (m, 2H), 3.45 (s, 3H), 3.30 – 3.24 (m, 2H), 2.09 (s, 3H); ^{13}C NMR (126 MHz, $CDCl_3$) δ 133.0, 129.1, 129.0, 128.9, 128.6, 127.8, 127.6, 126.3, 68.0, 61.6, 58.1, 47.7, 24.5, 24.0; HRMS (ESI) m/z: $[M]^+$ Calcd for $C_{14}H_{20}N$ 202.1591; Found 202.1593; HPLC (OX-H, ethanol/n-heptane/Et_3N/CF_3CO_2H = 20/80/0.5/0.3, flow rate = 1.0 mL/min, l = 220 nm) tR = 27.0 min, 30.0 min.

*rac-(E)-2-(hex-2-en-1-yl)-2-methyl-1,2,3,4-tetrahydroisoquinolin-2-ium bromide ([**3ag**]Br)*

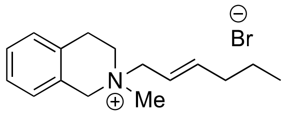

Orange solid (45 mg, 72%); refluxed (oil bath) for 2 h, volatiles removed under reduced pressure and oil recrystallized from CH_2Cl_2-THF; ^{1}H NMR (500 MHz, $CDCl_3$) δ 7.32 (t, J = 7.4 Hz, 1H), 7.27 (t, J = 7.5 Hz, 1H), 7.24 (d, J = 7.6 Hz, 1H), 7.14 (d, J = 7.6 Hz, 1H), 6.27 (dt, J = 15.2, 6.8 Hz, 1H), 5.64 (dt, J = 15.2, 7.4 Hz, 1H), 4.79 (AB spin system, $δ_A$ = 4.82, $δ_B$ = 4.76, J_{AB} = 15.2 Hz, 2H), 4.55 – 4.44 (m, 2H), 4.15 (dt, J = 12.6, 6.2 Hz, 1H), 3.96 (dt, J = 12.6, 6.9 Hz, 1H), 3.40 (s, 3H), 3.29 – 3.16 (m, 2H), 2.14 (q, J = 7.2 Hz, 2H), 1.45 ('sext', J = 7.4 Hz, 2H), 0.91 (t, J = 7.4 Hz, 3H); ^{13}C NMR (126 MHz, $CDCl_3$) δ 148.0, 129.0, 129.0, 129.0, 127.8, 127.6, 126.3, 115.6, 64.7, 61.0, 57.0, 47.4, 34.9, 23.9, 21.7, 13.8; HRMS (ESI) m/z: $[M]^+$ Calcd for $C_{16}H_{24}N$ 230.1908; Found 230.1910; HPLC (OX-H, ethanol/n-heptane/Et_3N/CF_3CO_2H = 20/80/0.5/0.3, flow rate = 1.0 mL/min, l = 220 nm) tR = 22.0 min, 23.8 min.

*rac-2,8-dimethyl-2-(3-methylbut-2-en-1-yl)-1,2,3,4-tetrahydroisoquinolin-2-ium bromide ([**3ba**]Br)*

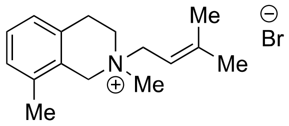

Grey solid (40 mg, 65%); ^{1}H NMR (500 MHz, $CDCl_3$) δ 7.17 (t, J = 7.6 Hz, 1H), 7.06 (d, J = 7.5 Hz, 1H), 7.04 (d, J = 7.5 Hz, 1H), 5.41 (t, J = 7.8 Hz, 1H), 4.78 (AB spin system, $δ_A$ = 4.81, $δ_B$ = 4.75, J_{AB} = 15.6 Hz, 2H), 4.52 – 4.38 (m, 2H), 4.05 – 4.01 (m, 1H), 3.91 – 3.88 (m, 1H), 3.42 (s, 3H), 3.23 – 3.14 (m, 2H), 2.26 (s, 3H), 1.86 (br.s, 3H), 1.81 (br.s, 3H); ^{13}C NMR (126 MHz, $CDCl_3$) δ 149.6, 136.1, 129.4, 128.7, 128.4, 126.6, 125.2, 110.5, 60.6, 59.1, 56.3, 48.0, 26.7, 24.2, 19.5, 19.4; HRMS (ESI) m/z: $[M]^+$ Calcd for $C_{16}H_{24}N$ 230.1904; Found 230.1907; HPLC (OX-H, ethanol/n-heptane/Et_3N/CF_3CO_2H = 20/80/0.5/0.3, flow rate = 1.0 mL/min, l = 220 nm) tR = 23.9 min, 28.0 min.

*rac-2-methyl-2-(3-methylbut-2-en-1-yl)-8-(trifluoromethyl)-1,2,3,4-tetrahydroisoquinolin-2-ium bromide ([**3ca**]Br)*

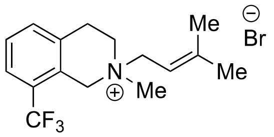

White solid (58 mg, 80%); ^{1}H NMR (500 MHz, $CDCl_3$) δ 7.58 (d, J = 7.6 Hz, 1H), 7.50 (d, J = 7.7 Hz, 1H), 7.43 (t, J = 7.7 Hz, 1H), 5.36 (t, J = 8.1 Hz, 1H), 4.69 (AB spin system, $δ_A$ = 4.73, $δ_B$ = 4.65, J_{AB} = 15.7 Hz, 2H), 4.53 (dd, J = 13.3, 7.9 Hz, 1H), 4.45 – 4.29 (m, 2H), 4.26 (dd, J = 13.3, 8.4 Hz, 1H), 3.44 – 3.33 (m, 2H), 3.40 (s, 3H), 1.85 (d, J = 1.3 Hz, 3H), 1.70 (d, J = 1.3 Hz, 3H); ^{13}C NMR (126 MHz, $CDCl_3$) δ 150.3, 133.4, 131.9, 128.8, 128.0 (q, J = 30.5 Hz), 125.4 (q, J = 5.7 Hz), 124.6 (q, J = 1.4 Hz), 123.7 (q, J = 274.2 Hz), 109.8, 60.3, 57.0, 56.5, 48.2, 26.6, 24.3, 19.2; ^{19}F NMR (470 MHz, $CDCl_3$) δ -60.34; HRMS (ESI) m/z: $[M]^+$ Calcd for

$C_{16}H_{21}F_3N$ 284.1621; Found 284.1626; HPLC (OX-H, ethanol/n-heptane/Et_3N/CF_3CO_2H = 20/80/0.5/0.3, flow rate = 1.0 mL/min, l = 220 nm) tR = 19.5 min, 24.7 min.

rac-2-methyl-2-(3-methylbut-2-en-1-yl)-1,2,3,4-tetrahydrobenzo[h]isoquinolin-2-ium bromide ([3da]Br)

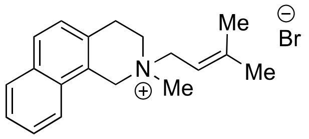

White solid (46 mg, 67%); ^{1}H NMR (500 MHz, $CDCl_3$) δ 7.85 – 7.77 (m, 2H), 7.75 (d, *J* = 8.5 Hz, 1H), 7.54 – 7.46 (m, 2H), 7.22 (d, *J* = 8.5 Hz, 1H), 5.42 (t, *J* = 8.1 Hz, 1H), 5.22 (AB spin system, δ_A = 5.25, δ_B = 5.20, J_{AB} = 16.0 Hz, 2H), 4.56 – 4.44 (m, 2H), 4.16 – 4.10 (m, 1H), 4.00 – 3.94 (m, 1H), 3.42 (s, 3H), 3.36 – 3.18 (m, 2H), 1.83 (br.s, 3H), 1.73 (br.s, 3H); ^{13}C NMR (126 MHz, $CDCl_3$) δ 149.8, 132.5, 130.1, 129.1, 128.9, 127.7, 127.1, 126.6, 126.1, 121.9, 121.6, 110.4, 60.9, 58.6, 56.1, 48.0, 26.6, 24.7, 19.5; HRMS (ESI) m/z: [M]$^+$ Calcd for $C_{19}H_{24}N$ 266.1904; Found 266.1907; HPLC (OX-H, ethanol/n-heptane/Et_3N/CF_3CO_2H = 20/80/0.5/0.3, flow rate = 1.0 mL/min, l = 220 nm) tR = 29.6 min, 33.6 min.

rac-8-bromo-2-methyl-2-(3-methylbut-2-en-1-yl)-1,2,3,4-tetrahydroisoquinolin-2-ium bromide ([3ea]Br)

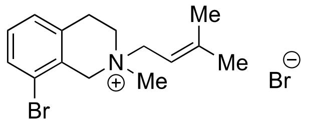

White solid (60 mg, 60%, 0.27 mmol scale), refluxed (oil bath) for 5h; ^{1}H NMR (500 MHz, $CDCl_3$) δ 7.51 (dd, *J* = 7.5, 1.7 Hz, 1H), 7.25 – 7.17 (m, 2H), 5.42 (m, 1H), 4.63 (d, *J* = 15.8 Hz, 1H), 4.61 – 4.56 (m, 1H), 4.54 (d, *J* = 15.8 Hz, 1H), 4.45 – 4.38 (m, 1H), 4.31 (dd, *J* = 13.2, 8.4 Hz, 1H), 4.24 – 4.17 (m, 1H), 3.47 (s, 3H), 3.35 – 3.26 (m, 2H), 1.90 (br.s, 3H), 1.78 (d, *J* = 1.3 Hz, 3H); ^{1}H NMR (500 MHz, DMSO-d_6) δ 7.62 (dd, *J* = 7.8, 1.2 Hz, 1H), 7.37 (dd, *J* = 7.8, 1.2 Hz, 1H), 7.32 (t, *J* = 7.8 Hz, 1H), 5.49 (m, 1H), 4.58 – 4.50 (m, 2H), 4.12 (dd, *J* = 13.4, 8.7 Hz, 1H), 3.98 (dd, *J* = 13.4, 7.4 Hz, 1H), 3.67 – 3.57 (m, 2H), 3.22 (t, *J* = 6.6 Hz, 2H), 3.04 (s, 3H), 1.86 (br.s, 3H), 1.70 (d, *J* = 1.3 Hz, 3H); ^{13}C NMR (126 MHz, $CDCl_3$) δ 150.0, 132.3, 131.8, 130.0, 128.4, 126.1, 122.9, 110.2, 60.4, 60.3, 56.7, 48.1, 26.6, 24.3, 19.2; HRMS (ESI) m/z: [M]$^+$ Calcd for $C_{15}H_{21}BrN$ 294.0852; Found 294.0853; HPLC (OX-H, ethanol/n-heptane/Et_3N/CF_3CO_2H = 20/80/0.5/0.3, flow rate = 1.0 mL/min, l = 220 nm) tR = 30.1 min, 39.8 min.

rac-5-bromo-2-methyl-2-(3-methylbut-2-en-1-yl)-1,2,3,4-tetrahydroisoquinolin-2-ium bromide ([3fa]Br)

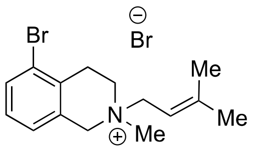

White solid (38 mg, 50%), refluxed (oil bath) for 1h; ^{1}H NMR (500 MHz, $CDCl_3$) δ 7.56 – 7.53 (m, 1H), 7.17 – 7.14 (m, 2H), 5.40 (t, *J* = 8.1 Hz, 1H), 5.01 (AB spin system, δ_A = 5.03, δ_B = 4.98, J_{AB} = 16.0 Hz, 2H), 4.53 – 4.41 (m, 2H), 4.25 – 4.18 (m, 1H), 4.10 – 4.03 (m, 1H), 3.37 (s, 3H), 3.15 – 3.04 (m, 2H), 1.88 (br.s, 3H), 1.85 (br.s, 3H); ^{13}C NMR (126 MHz, $CDCl_3$) δ 145.0, 132.9, 129.3, 129.2, 129.0, 1267.0, 125.1, 110.2, 60.7, 60.6, 56.5, 47.0, 26.7, 25.7, 19.7; HRMS (ESI) m/z: [M]$^+$ Calcd for $C_{15}H_{21}BrN$ 294.0852; Found 294.0855; HPLC (IB-N5,

ethanol/n-heptane/Et_3N/CF_3CO_2H = 10/90/0.1/0.3, flow rate = 1.0 mL/min, l = 220 nm) tR = 14.4 min, 15.8 min.

rac-6,7-dimethoxy-2-methyl-2-(3-methylbut-2-en-1-yl)-1,2,3,4-tetrahydroisoquinolin-2-ium bromide ([3ga]Br)

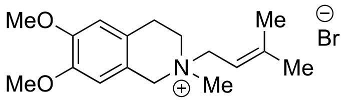

White solid (58 mg, 81%); ^{1}H NMR (500 MHz, $CDCl_3$) δ 6.67 (s, 1H), 6.61 (s, 1H), 5.41 (t, *J* = 8.1 Hz, 1H), 4.72 (AB spin system, δ_A = 4.75, δ_B = 4.69, J_{AB} = 15.0 Hz, 2H), 4.46 ('qd', *J* = 13.3, 8.1 Hz, 2H), 4.10 – 4.03 (m, 1H), 3.95 – 3.88 (m, 1H), 3.87 (s, 3H), 3.84 (s, 3H), 3.38 (s, 3H), 3.19 – 3.08 (m, 2H), 1.88 (br.s, 3H), 1.89 (br.s, 3H); ^{13}C NMR (126 MHz, $CDCl_3$) δ 149.5, 149.4, 148.8, 121.0, 118.2, 111.1, 110.5, 109.8, 60.7, 60.5, 57.0, 56.3, 56.1, 47.1, 26.6, 23.7, 19.6; HRMS (ESI) m/z: $[M]^+$ Calcd for $C_{17}H_{26}NO_2$ 276.1959; Found 276.1958; HPLC (OX-H, ethanol/n-heptane/Et_3N/CF_3CO_2H = 20/80/0.5/0.3, flow rate = 1.0 mL/min, l = 220 nm) tR = 48.5 min, 54.7 min.

rac-2-methyl-2-(3-methylbut-2-en-1-yl)-7-nitro-1,2,3,4-tetrahydroisoquinolin-2-ium bromide ([3ha]Br)

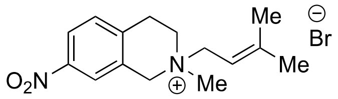

Orange solid (31 mg, 45%); ^{1}H NMR (500 MHz, $CDCl_3$) δ 8.15 (dd, *J* = 8.5, 2.3 Hz, 1H), 8.10 (d, *J* = 2.3 Hz, 1H), 7.47 (d, *J* = 8.5 Hz, 1H), 5.41 (m, 1H), 5.00 (AB spin system, δ_A = 5.01, δ_B = 4.97, J_{AB} = 15.6 Hz, 2H), 4.37 (d, *J* = 8.2 Hz, 2H), 4.22 – 4.15 (m, 1H), 4.09 – 4.01 (m, 1H), 3.32 – 3.40 (m, 2H), 3.30 (s, 3H), 1.90 (d, *J* = 1.3 Hz, 3H), 1.85 (d, *J* = 1.3 Hz, 3H); ^{13}C NMR (126 MHz, $CDCl_3$) δ 150.3, 147.0, 137.0, 130.4, 128.6, 123.5, 122.9, 109.9, 61.4, 60.3, 56.0, 47.0, 26.6, 24.3, 19.4; HRMS (ESI) m/z: $[M]^+$ Calcd for $C_{15}H_{21}N_2O_2$ 261.1598; Found 261.1600; HPLC (OX-H, ethanol/n-heptane/Et_3N/CF_3CO_2H = 20/80/0.5/0.3, flow rate = 1.0 mL/min, l = 220 nm) tR = 28.6 min, 34.2 min.

rac-2,4,4-trimethyl-2-(3-methylbut-2-en-1-yl)-1,2,3,4-tetrahydroisoquinolin-2-ium bromide ([3ia]Br)

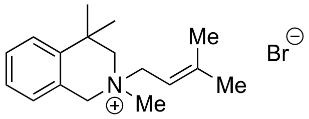

White solid (32 mg, 50%); ^{1}H NMR (500 MHz, $CDCl_3$) δ 7.43 – 7.40 (m, 2H), 7.30 – 7.27 (m, 1H), 7.17 (d, *J* = 7.6 Hz, 1H), 5.38 (t, *J* = 8.0 Hz, 1H), 4.78 (AB spin system, δ_A = 4.80, δ_B = 4.75, J_{AB} = 14.6 Hz, 2H), 4.51 (dd, *J* = 13.2, 8.0 Hz, 1H), 4.44 (dd, *J* = 13.2, 8.3 Hz, 1H), 3.96 – 3.88 (m, 2H), 3.32 (s, 3H), 1.91 (s, 3H), 1.88 (s, 3H), 1.58 (s, 3H), 1.53 (s, 3H); ^{13}C NMR (126 MHz, $CDCl_3$) δ 150.1, 139.7, 129.9, 127.9, 127.6, 126.3, 125.2, 110.5, 68.8, 62.2, 61.3, 48.1, 34.2, 32.5, 32.3, 26.7, 19.7; HRMS (ESI) m/z: $[M]^+$ Calcd for $C_{17}H_{26}N$ 244.2060; Found 244.2060; HPLC (OX-H, ethanol/n-heptane/Et_3N/CF_3CO_2H = 20/80/0.5/0.3, flow rate = 1.0 mL/min, l = 220 nm) tR = 23.6 min, 25.5 min.

rac-2-methyl-2-(3-methylbut-2-en-1-yl)-2,3,4,5-tetrahydro-1H-benzo[c]azepin-2-ium bromide ([3ja]Br)

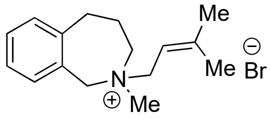

White solid (35 mg, 56%), refluxed (oil bath) for 1h; ^{1}H NMR (600 MHz, 358 K, TCE-d_2) δ 7.61 (d, *J* = 7.4 Hz, 1H), 7.42 (td, *J* = 7.5, 1.4 Hz, 1H), 7.34 (td, *J* = 7.5, 1.4 Hz, 1H), 7.26 (d, *J* = 7.4 Hz, 1H), 5.40 (t, *J* = 8.1 Hz, 1H), 5.11 (d, *J* = 13.7 Hz, 1H), 4.98 (d, *J* = 13.7 Hz, 1H), 4.43 (dd, *J* = 13.5, 8.4 Hz, 1H), 4.22 – 4.15 (m, 1H), 3.99 – 3.93 (m, 1H), 3.87 – 3.81 (m, 1H), 3.26 – 3.17 (m, 1H), 3.16 – 3.05 (m, 4H), 2.18 – 2.03 (m, 2H), 1.96 (br.s, 3H), 1.94 (br.s, 3H); ^{13}C NMR (126 MHz, $CDCl_3$) δ 149.1, 142.8, 133.3, 130.6, 129.3, 128.9, 127.6, 110.7, 68.1, 66.3, 64.9, 33.3, 26.7, 25.7, 23.8, 19.7; HRMS (ESI) m/z: [M]$^+$ Calcd for $C_{16}H_{24}N$ 230.1904; Found 230.1905; HPLC (OX-H, ethanol/n-heptane/Et_3N/CF_3CO_2H = 20/80/0.5/0.3, flow rate = 1.0 mL/min, l = 220 nm) tR = 24.2 min, 25.9 min.

rac-2-methyl-2-(3-methylbut-2-en-1-yl)-5-phenyl-1,2,3,4-tetrahydroisoquinolin-2-ium bromide ([3la]Br)

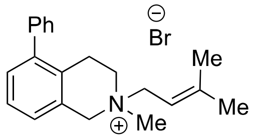

Pale brown solid (46 mg, 62%), refluxed (oil bath) for 1h; ^{1}H NMR (500 MHz, $CDCl_3$) δ 7.45 – 7.40 (m, 2H), 7.39 – 7.35 (m, 1H), 7.32 (t, *J* = 7.6 Hz, 1H), 7.25 – 7.20 (m, 3H), 7.15 (d, *J* = 7.6 Hz, 1H), 5.40 (t, *J* = 8.0 Hz, 1H), 4.89 (AB spin system, δ_A = 4.92, δ_B = 4.86, J_{AB} = 15.2 Hz, 2H), 4.53 (dd, *J* = 13.3, 8.1 Hz, 1H), 4.47 (dd, *J* = 13.3, 8.1 Hz, 1H), 4.14 – 4.06 (m, 1H), 3.95 – 3.85 (m, 1H), 3.43 (br.s, 3H), 3.02 (dt, *J* = 18.7, 6.4 Hz, 1H), 2.94 (dt, *J* = 18.7, 6.4 Hz, 1H), 1.87 (br.s, 6H); ^{13}C NMR (126 MHz, $CDCl_3$) δ 149.8, 142.6, 139.7, 130.3, 128.9, 128.7, 127.9, 127.9, 127.0, 126.9, 126.8, 110.3, 61.2, 60.7, 57.1, 47.3, 26.7, 23.4, 19.7; HRMS (ESI) m/z: [M]$^+$ Calcd for $C_{21}H_{26}N$ 292.2060; Found 292.2063; HPLC (OZ-H, ethanol/n-heptane/Et_3N/CF_3CO_2H = 10/90/0.5/0.3, flow rate = 1.0 mL/min, l = 220 nm) tR = 40.7 min, 44.1 min.

rac-2-methyl-2-(3-methylbut-2-en-1-yl)-5-(m-tolyl)-1,2,3,4-tetrahydroisoquinolin-2-ium bromide ([3ma]Br)

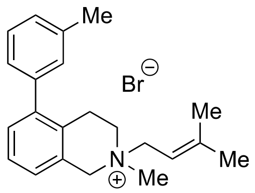

White solid (43 mg, 75% with respect to 0.15 mmol of the starting amine); ^{1}H NMR (500 MHz, $CDCl_3$) δ 7.29 (td, *J* = 7.6, 4.2 Hz, 2H), 7.20 – 7.15 (m, 2H), 7.13 (d, *J* = 7.6 Hz, 1H), 7.03 (br.s, 1H), 7.01 (d, *J* = 7.7 Hz, 1H), 5.40 (m, 1H), 4.88 (AB spin system, δ_A = 4.90, δ_B = 4.85, J_{AB} = 15.2 Hz, 2H), 4.53 (dd, *J* = 13.4, 8.2 Hz, 1H), 4.46 (dd, *J* = 13.4, 8.1 Hz, 1H), 4.14 – 4.07 (m, 1H), 3.95 – 3.87 (m, 1H), 3.41 (s, 3H), 3.00 (dt, *J* = 18.7, 6.9 Hz, 1H), 2.92 (dt, *J* = 18.7, 6.2 Hz, 1H), 2.38 (s, 3H), 1.88 – 1.82 (m, 6H); ^{13}C NMR (126 MHz, $CDCl_3$) δ 149.5, 142.7, 139.6, 138.4, 130.2, 129.5, 128.6, 128.5, 127.7, 127.0, 126.7, 126.7, 125.9, 110.4, 61.1, 60.6, 57.0, 47.2, 26.6, 23.3, 21.5, 19.6; HRMS (ESI) m/z: [M]$^+$ Calcd for $C_{22}H_{28}N$ 306.2216; Found 306.2219; Found 292.2063; HPLC (OZ-H, ethanol/n-heptane/Et_3N/CF_3CO_2H = 10/90/0.5/0.3, flow rate = 1.0 mL/min, l = 220 nm) tR = 34.2 min, 37.6 min.

*rac-2-methyl-2-(3-methylbut-2-en-1-yl)-5-(p-tolyl)-1,2,3,4-tetrahydroisoquinolin-2-ium bromide ([**3na**]Br)*

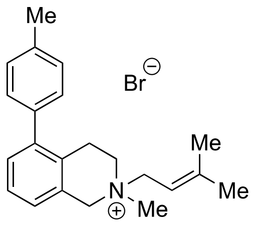

White solid (24 mg, 63%, 0.10 mmol scale); ^{1}H NMR (500 MHz, $CDCl_3$) δ 7.33 (t, *J* = 7.6 Hz, 1H), 7.26 – 7.22 (m, 3H), 7.16 – 7.11 (m, 3H), 5.39 (m, 1H), 4.85 (AB spin system, $δ_A$ = 4.89, $δ_B$ = 4.80, J_{AB} = 15.2 Hz, 2H), 4.52 (dd, *J* = 13.4, 8.2 Hz, 1H), 4.44 (dd, *J* = 13.4, 8.2 Hz, 1H), 4.12 – 4.04 (m, 1H), 3.92 – 3.83 (m, 1H), 3.42 (s, 3H), 3.04 (dt, *J* = 18.7, 6.8 Hz, 1H), 2.97 (dt, *J* = 18.7, 6.2 Hz, 1H), 2.41 (s, 3H), 1.89 (d, *J* = 1.4 Hz, 6H); ^{13}C NMR (126 MHz, $CDCl_3$) δ 149.4, 142.4, 137.5, 136.7, 130.3, 129.3 (2C), 128.7 (2C), 127.7, 127.1, 126.8, 126.6, 110.4, 61.0, 60.5, 57.0, 47.2, 26.6, 23.3, 21.3, 19.6; HRMS (ESI) m/z: [M]$^+$ Calcd for $C_{22}H_{28}N$ 306.2216; Found 306.2218; Found 292.2063; HPLC (OX-H, ethanol/n-heptane/Et_3N/CF_3CO_2H = 20/80/0.5/0.3, flow rate = 1.0 mL/min, l = 220 nm) tR = 22.4 min, 23.9 min.

*rac-5-(3,5-dimethylphenyl)-2-methyl-2-(3-methylbut-2-en-1-yl)-1,2,3,4-tetrahydroisoquinolin-2-ium bromide ([**3oa**]Br)*

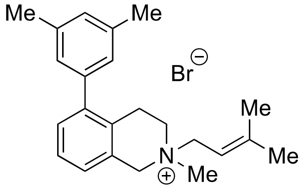

White solid (26 mg, 88% with respect to 75 µmol of the starting amine); ^{1}H NMR (500 MHz, $CDCl_3$) δ 7.28 (t, *J* = 7.6 Hz, 1H), 7.18 (dd, *J* = 7.6, 1.3 Hz, 1H), 7.12 (dd, *J* = 7.7, 1.3 Hz, 1H), 7.01 – 6.97 (m, 1H), 6.84 – 6.80 (m, 2H), 5.40 (m, 1H), 4.87 (AB spin system, $δ_A$ = 4.88, $δ_B$ = 4.84, J_{AB} = 15.2 Hz, 2H), 4.53 (dd, *J* = 13.3, 8.1 Hz, 1H), 4.46 (dd, *J* = 13.3, 8.1 Hz, 1H), 4.17 – 4.05 (m, 1H), 3.97 – 3.86 (m, 1H), 3.42 (s, 3H), 3.00 (dt, *J* = 18.7, 6.9 Hz, 1H), 2.93 (dt, *J* = 18.7, 6.2 Hz, 1H), 2.34 (s, 6H), 1.85 (dd, *J* = 3.5, 1.3 Hz, 6H); ^{13}C NMR (126 MHz, $CDCl_3$) δ 149.5, 142.8, 139.6, 138.3 (2C), 130.2, 129.4, 127.7, 127.0, 126.6, 126.6, 126.6 (2C), 110.4, 61.1, 60.6, 57.1, 47.3, 26.6, 23.3, 21.4 (2C), 19.6; HRMS (ESI) m/z: [M]$^+$ Calcd for $C_{23}H_{30}N$ 320.2373; Found 320.2372; Found 292.2063; HPLC (OZ-H, ethanol/n-heptane/Et_3N/CF_3CO_2H = 10/90/0.5/0.3, flow rate = 1.0 mL/min, l = 220 nm) tR = 28.0 min, 31.3 min.

*rac-N-benzyl-N-(2-hydroxyethyl)-N,3-dimethylbut-2-en-1-aminium bromide ([**3pa**]Br)*

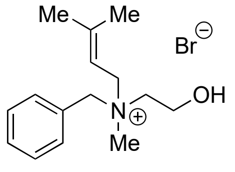

White solid (33 mg, 52%); ^{1}H NMR (500 MHz, $CDCl_3$) δ 7.63 (d, *J* = 7.3 Hz, 2H), 7.50 – 7.43 (m, 3H), 5.41 (t, *J* = 8.0 Hz, 1H), 5.21 (t, *J* = 5.8 Hz, 1H), 4.90 (AB spin system, $δ_A$ = 4.96, $δ_B$ = 4.84, J_{AB} = 12.9 Hz, 2H), 4.34 (dd, *J* = 13.6, 7.8 Hz, 1H), 4.30 – 4.24 (m, 2H), 4.13 (dd, *J* = 13.6, 8.0 Hz, 1H), 3.59 – 3.52 (m, 1H), 3.51 – 3.45 (m, 1H), 3.11 (br.s, 3H), 1.88 (s, 3H), 1.80

(s, 3H); ^{13}C NMR (126 MHz, $CDCl_3$) δ 148.4, 133.6 (2C), 130.6, 129.2 (2C), 127.6, 111.0, 65.8, 61.3, 60.3, 55.8, 47.4, 26.7, 19.5; HRMS (ESI) m/z: $[M]^+$ Calcd for $C_{15}H_{24}NO$ 234.1852; Found 234.1854; Found 292.2063; HPLC (OZ-H, ethanol/n-heptane/Et_3N/CF_3CO_2H = 10/90/0.5/0.3, flow rate = 1.0 mL/min, l = 220 nm) tR = 51.7 min, 56.5 min.

*rac-1-methyl-1-(3-methylbut-2-en-1-yl)-3,3-diphenylpyrrolidin-1-ium bromide ([**3qa**]Br)*

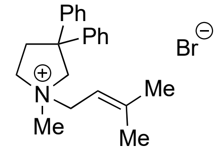

White solid (70 mg, 89%); ^1H NMR (500 MHz, $CDCl_3$) δ 7.38 – 7.28 (m, 8H), 7.21 (td, *J* = 6.7, 1.8 Hz, 2H), 5.30 (t, *J* = 8.0 Hz, 1H), 4.65 – 4.55 (m, 2H), 4.42 (dd, *J* = 13.5, 7.6 Hz, 1H), 4.28 (dd, *J* = 13.5, 8.3 Hz, 1H), 4.00 – 3.90 (m 1H), 3.89 – 3.80 (m, 1H), 3.34 (s, 3H), 3.08 (dt, *J* = 14.3, 7.1 Hz, 1H), 3.02 (dt, *J* = 14.3, 7.1 Hz, 1H), 1.78 (s, 3H), 1.75 (s, 3H); ^{13}C NMR (126 MHz, $CDCl_3$) δ 148.8, 143.4, 143.2, 129.5 (4C), 127.7, 127.6, 126.3 (2C), 126.2 (2C) 111.4, 73.8, 64.6, 63.6, 55.0, 53.2, 36.4, 26.5, 19.4; HRMS (ESI) m/z: $[M]^+$ Calcd for $C_{22}H_{28}N$ 306.2216; Found 306.2220; Found 292.2063; HPLC (OZ-H, ethanol/n-heptane/Et_3N/CF_3CO_2H = 10/90/0.5/0.3, flow rate = 1.0 mL/min, l = 220 nm) tR = 40.2 min, 43.1 min.

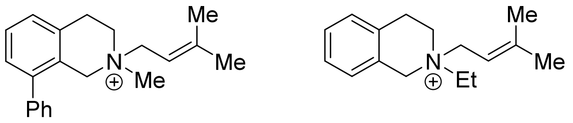

3ka **3ra**

The bromide salts **3ka** and **3ra** were not obtained in the required purity. To obtain racemic materials as standards, the acetate salts were prepared by catalytic means with racemic ligands (see section *Catalysis*, *Preparative scale*).

Preparation of enantioenriched ammonium salts by diastereomeric salt formation and fractional crystallization

silver(I) (2R,3R)-2,3-bis(benzoyloxy)-3-carboxypropanoate (S1)

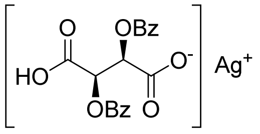

Sodium hydroxide (1.12 g, 28.0 mmol) was added to a suspension of (2*R*,3*R*)-2,3-bis(benzoyloxy)succinic acid (10.0 g, 27.9 mmol) in water (200 mL) and the mixture heated with a heat gun until a clear solution formed. Subsequently, a freshly prepared solution of silver nitrate (4.76 g, 28.0 mol) in water (5 mL) was added to the reaction mixture under vigorous stirring. Stirring was continued for 2 hours before the precipitate was collected by filtration, washed with water and dried under reduced pressure.

Light grey solid (10.1 g, 78%); ^1H NMR (500 MHz, DMSO-d_6) δ 8.02 – 7.96 (m, 2H), 7.71 – 7.64 (m, 1H), 7.59 – 7.52 (m, 2H), 5.76 (s, 1H); ^{13}C NMR (126 MHz, DMSO-d_6) δ 168.8, 165.4, 133.9, 129.8, 129.6, 129.1, 73.8.

2-allyl-2-methyl-1,2,3,4-tetrahydroisoquinolin-2-ium (2R,3R)-2,3-bis(benzoyloxy)-3-carboxypropanoate (S2)

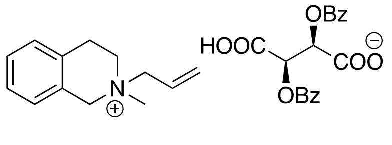

rac-2-allyl-2-methyl-1,2,3,4-tetrahydroisoquinolin-2-ium bromide [**3ab**]Br (2.33 g, 8.69 mmol) was dissolved in methanol (30 mL) and the mono silver(I) salt of (L)-dibenzoyl tartaric acid **S1** (4.04 g, 8.68 mmol) was added. The mixture was vigorously stirred for 15 min before silver bromide was filtered off and volatiles were removed under reduced pressure. The obtained diastereomeric salt was recrystallized in 4-5 rounds from methanol to yield a *dr* of 6.5:93.5.

Pale brown solid (2.42 g, 51% crude material before recrystallization); ^{1}H NMR (500 MHz, DMSO-d$_6$) δ 7.88 (dd, *J* = 7.7, 1.2 Hz, 4H), 7.59 (m, 2H), 7.45 (t, *J* = 7.7 Hz 4H), 7.38 – 7.27 (m, 3H), 7.20 (d, *J* = 7.4 Hz, 1H), 6.15 ('ddt', *J* = 17.4, 10.2, 7.3 Hz, 1H), 5.70 – 5.61 (m, 2H), 5.60 (s, 2H), 4.57 (AB spin system, δ_A = 4.61, δ_B = 4.53, J_{AB} = 15.5 Hz, 2H), 4.07 – 3.99 (m, 2H), 3.73 – 3.58 (m, 2H), 3.21 – 3.14 (m, 2H), 3.03 (s, 3H); ^{13}C NMR (126 MHz, CDCl$_3$) δ 170.5, 166.2, 133.0, 130.1, 130.0, 129.0, 128.9, 128.9, 128.3, 127.7, 127.5, 126.1, 124.0, 73.5, 64.9, 61.4, 57.6, 48.0, 23.7; HRMS (ESI) m/z: [M]$^+$ Calcd for $C_{13}H_{18}N$ 188.1434; Found 188.1436; [M]$^-$ Calcd for $C_{18}H_{13}O_8$ 357.0616; Found 357.0613.

After diastereomeric enrichment, the chiral anion was exchanged via preparative HPLC (see Section *HPLC methods*) to obtain the trifluoroacetate salt.

2-allyl-2-methyl-1,2,3,4-tetrahydroisoquinolin-2-ium trifluoroacetate (er 7:93)

Yellow oil (48 mg, 97%); ^{1}H NMR (500 MHz, DMSO-d$_6$) δ 7.39 – 7.27 (m, 3H), 7.21 (d, *J* = 7.4 Hz, 1H), 6.16 ('ddt', *J* = 17.3, 10.2, 7.3 Hz, 1H), 5.71 – 5.60 (m, 2H), 4.58 (AB spin system, δ_A = 4.61, δ_B = 4.54, J_{AB} = 15.4 Hz, 2H), 4.07 – 4.00 (m, 2H), 3.72 – 3.61 (m, 2H), 3.18 (t, *J* = 6.5 Hz, 2H), 3.03 (s, 3H).

2-methyl-2-(3-methylbut-2-en-1-yl)-1,2,3,4-tetrahydroisoquinolin-2-ium (2R,3R)-2,3-bis(benzoyloxy)-3-carboxypropanoate (S3)

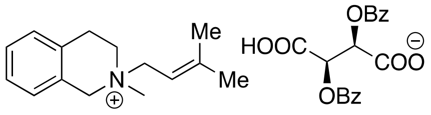

rac-2-methyl-2-(3-methylbut-2-en-1-yl)-1,2,3,4-tetrahydroisoquinolin-2-ium bromide [**3aa**]Br (1.14 g, 3.85 mmol) was dissolved in methanol (15 mL) and the mono silver (I) salt of (L)-dibenzoyl tartaric acid **S1** (1.77 g, 3.60 mmol) was added. The mixture was stirred vigorously for 15 min before filtration and removal of volatiles from the filtrate. The obtained diastereomeric salt was recrystallized 5 to 6 times from methanol to yield a dr of 97:3. In a second experiment a dr of 95:5 was obtained.

White solid (2.28 g, 96% crude product before recrystallization); ^{1}H NMR (500 MHz, DMSO-d$_6$) δ 7.91 – 7.85 (m, 4H), 7.62 – 7.56 (m, 2H), 7.48 – 7.41 (m, 4H), 7.38 – 7.27 (m, 3H), 7.21 (d, *J* = 7.4 Hz, 1H), 5.60 (s, 2H), 5.49 (m, 1H), 4.53 (AB spin system, δ_A = 4.57, δ_B = 4.48, J_{AB} = 15.4 Hz, 2H), 4.03 – 3.94 (m, 2H), 3.65 (t, *J* = 6.6 Hz, 2H), 3.18 (t, *J* = 6.6 Hz, 2H), 2.96 (s, 3H), 1.85 (br.s, 3H), 1.71 (d, *J* = 1.3 Hz, 3H); ^{13}C NMR (126 MHz, DMSO-d$_6$) δ 168.2, 165.3, 147.3, 133.7, 130.0, 129.9, 129.5, 129.1, 128.9, 128.5, 127.4, 127.3, 127.2, 111.1, 72.7, 60.5, 60.1, 56.5, 46.5, 26.2, 23.4, 18.6; HRMS (ESI) m/z: [M]$^+$ Calcd for $C_{15}H_{22}N$ 216.1747; Found

216.1749; $[M]^-$ Calcd for $C_{18}H_{13}O_8$ 357.0616; Found 357.0618. The salt was diastereomerically enriched in 5-6 rounds of recrystallization from methanol (dr 97:3). Crystals for X-ray structural analysis were grown in an NMR tube by slow diffusion of ethyl acetate into a chloroform-methanol solution of salt **S3**.

After diastereomeric enrichment, the chiral anion was exchanged via preparative HPLC (see Section *HPLC methods*) to obtain the trifluoroacetate salt.

2-methyl-2-(3-methylbut-2-en-1-yl)-1,2,3,4-tetrahydroisoquinolin-2-ium trifluoroacetate (er 90.5:9.5)

Yellow viscous oil (57 mg, 98%); ^{1}H NMR (500 MHz, $CDCl_3$) δ 7.34 (td, *J* = 7.5, 1.4 Hz, 1H), 7.29 (td, *J* = 7.5, 1.4 Hz, 1H), 7.24 (d, *J* = 7.5 Hz, 1H), 7.13 (d, *J* = 7.5 Hz, 1H), 5.39 (t, *J* = 8.1 Hz, 1H), 4.54 (AB spin system, $δ_A$ = 4.57, $δ_B$ = 4.49, J_{AB} = 15.3 Hz, 2H), 4.19 – 4.06 (m, 2H), 3.87 – 3.80 (m, 1H), 3.80 – 3.73 (m, 1H), 3.23 (t, *J* = 6.2 Hz, 2H), 3.17 (s, 3H), 1.89 (br.s, 3H), 1.74 (d, *J* = 1.3 Hz, 3H); $[\alpha]_D^{22.4}$ = -2.97° (*er* 90.5:9.5, c 3.0, CH_3CN).

Catalysis

General: All catalysis reactions were carried out in a glovebox under a nitrogen atmosphere. Solvents for catalysis were degassed by at least three freeze-pump-thaw cycles. $[PdCl(C_3H_5)]_2$ and ligands were weighed in the glovebox and dissolved in dichloromethane to obtain stock solutions (5-30 mM for $[PdCl(C_3H_5)]_2$ and 10-30 mM for ligands). Nucleophile and electrophile were either employed as stock solutions or as neat materials. Analytical scale reactions were carried out in 2.0 mL HPLC vials with PTFE lined screw caps and agitated in a thermoshaker (25°C, 700 rpm, Eppendorf ThermoMixer C, SmartBlock cryo thaw, Eppendorf ThermoTop).

For the determination of HPLC-yields by reversed phase HPLC, aliquots of the reaction mixture were diluted in methanol outside of the glovebox and either internal standard stock solution (300 mM benzyl alcohol in DMSO) was added or the respective experimentally determined response factors for the amine/allyl ammonium ion were considered (see section for HPLC methods).

For the determination of the enantiomeric ratio, aliquots (100 µL) of reactions *without* water were diluted in EtOH/heptane (20:80, 200 µL) outside of the glovebox before analysis.
Aliquots (100 µL) of partly *aqueous* samples were removed from the glovebox, exposed to air and volatiles removed with a speedvac concentrator (45 °C, 1.5 h). The residue was then dissolved in EtOH/heptane (20:80, 200 µL) for HPLC-analysis.

The following densities were used in the calculations:

Table S1. Density values

**Structure**	**Density**
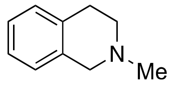	0.9896 g/cm^3 [20]
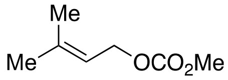	0.978±0.06 g/cm^3 [21]
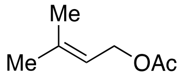	0.9169 g/cm^3 [22]
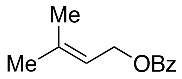	1.018±0.06 g/cm^3 [21]
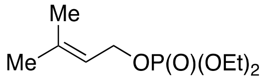	1.039±0.06 g/cm^3 [21]
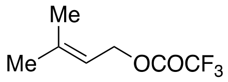	1.157±0.06 g/cm^3 [21]
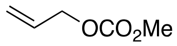	1.0261 g/cm^3 [23]
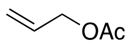	0.9272 g/cm^3 [24]

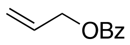	1.056 g/cm^3 [25]
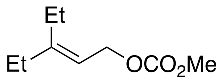	0.946±0.06 g/cm^3 [21]
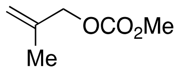	0.983±0.06 g/cm^3 [21]
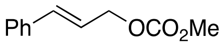	1.109±0.06 g/cm^3 [21]
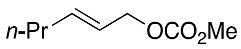	0.964±0.06 g/cm^3 [21]

Analytical scale

Optimized conditions, representative procedure: $[PdCl(C_3H_5)]_2$ (stock solution in CH_2Cl_2, 6.3 mM; 8.0 µL or 8.3 mM; 6.0 µL, 50 nmol) and (*S*,*S*)-DACH-Ph-Trost ligand (stock solution in CH_2Cl_2, 10.3 mM, 10.0 µL, 103 nmol) were added to methanol (389 µL or 390 µL, respectively) in an HPLC-vial and the mixture agitated for 10 minutes (25 °C, 700 rpm). Subsequently, the electrophile methyl (3-methylbut-2-en-1-yl) carbonate (4.12 µL, 27.9 µmol, density 0.978±0.06 g/cm^3 predicted, ACD/Labs V11.02) was added, followed by water (489 or 490 µL, respectively). The tertiary amine nucleophile (stock solution in MeOH, 100 mM, 100 µL, 10 µmol) was added immediately after the addition of water and the mixture was agitated in the thermoshaker. The total volume added up to 1000 µL and contained less then 2 vol% CH_2Cl_2. The HPLC-yield was determined by reversed phase HPLC of aliquots (250 µL) that were diluted with MeOH (250 µL) outside of the glovebox as described above and to which standard stock solution (296 mM benzyl alcohol in DMSO, 10 µL) was added if applicable. The enantiomeric ratio was determined as described above. Conditions for HPLC-analysis of the individual products are listed in the section for HPLC methods.

Deviating conditions are indicated in the respective tables and schemes.

Preparative scale

Note: Chemical shifts in ^{1}H NMR spectra in $CDCl_3$ for ammonium ions show high mobility. A varying D^+ or H^+ concentration likely contributes to this effect, as might concentration and counteranion content and nature. When measured in DMSO-d_6, the shifts of the racemic ammonium bromide signals are identical to those observed for the enantioenriched material prepared in preparative catalysis runs (confirmed for **3aa** and **3ea**).

Work-up under addition of water

*2-methyl-2-(3-methylbut-2-en-1-yl)-1,2,3,4-tetrahydroisoquinolin-2-ium bicarbonate ([**3aa**][HCO_3])*

To check the scalability of the developed system, a reaction was set up on a 1.0 mmol scale (analytical scale × 100).

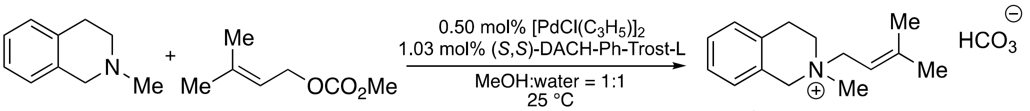

1a 1.0 mmol; **2a** 3.0 eq; **3aa**

Allylpalladium chloride dimer (800 µL of 6.26 mM solution in CH_2Cl_2, 5.01 µmol) and (*S*,*S*)-DACH-phenyl Trost ligand (500 µL of a 20.6 mM solution in CH_2Cl_2, 10.3 µmol) were stirred with a magnetic stir bar for 10 minutes in methanol (49 mL) inside the glovebox under a nitrogen atmosphere. Methyl (3-methylbut-2-en-1-yl) carbonate (433 mg, 3.00 mmol) was added and stirring continued for 5 minutes before water (49 mL) was added, followed by 2-methyl-1,2,3,4-tetrahydroisoquinoline (147 mg, 1.00 mmol). The reaction mixture was left stirring vigorously at room temperature in the glovebox.

After 23 hours, samples for HPLC-yield (250 µL) and *er* determination (100 µL) by HPLC were taken, and after RP-HPLC analysis indicated high conversion, the flask was removed from the glovebox (after 24h), the mixture exposed to air and the solvents were evaporated under reduced pressure at the rotary evaporator (water bath 37 °C). The crude material was dissolved in water (50 mL) and the aqueous phase was washed with TBME (30 mL). The aqueous fraction was partially evaporated (water bath 38 °C) at the rotary evaporator under reduced pressure, before being frozen in liquid nitrogen and lyophilized overnight. The product was obtained as the bicarbonate salt according to NMR analysis, 267 mg (96%), *er* 76.5:24.5. HPLC analysis before the work-up showed an HPLC-yield of 95% and an *er* of 77:23.

^{1}H NMR (500 MHz, $CDCl_3$) δ 7.23 – 7.11 (m, 4H), 5.36 (t, *J* = 8.0 Hz, 1H), 4.54 (AB spin system, δ_A = 4.58, δ_B = 4.50, J_{AB} = 15.5 Hz, 2H), 4.14 – 4.02 (m, 2H), 3.87 (dt, *J* = 13.0, 6.6 Hz, 1H), 3.76 (dt, *J* = 13.0, 6.6 Hz, 1H), 3.21 – 3.08 (m, 5H), 1.79 (br.s, 3H), 1.63 (br.s, 3H); ^{13}C NMR (126 MHz, $CDCl_3$) δ 160.5, 148.7, 129.6, 128.9, 128.6, 127.8, 127.5, 127.0, 110.8, 60.4, 59.8, 57.1, 47.9, 26.6, 23.9, 18.9; HRMS (ESI) m/z: $[M]^+$ Calcd for $C_{15}H_{22}N$ 216.1747; Found 216.1750; HPLC (OX-H, ethanol/n-heptane/Et_3N/CF_3CO_2H = 20/80/0.5/0.3, flow rate = 1.0 mL/min, l = 220 nm) tR = 26.1 min (major), 27.7 min (minor).

Identification of the counteranion as bicarbonate is based on i) the observation of a signal at 160.5 ppm by ^{13}C NMR and ii) the lack of unidentified signals in the ^{1}H-NMR spectrum.

A sample of NH_4HCO_3 in D_2O (100 mM) was recorded for comparison.

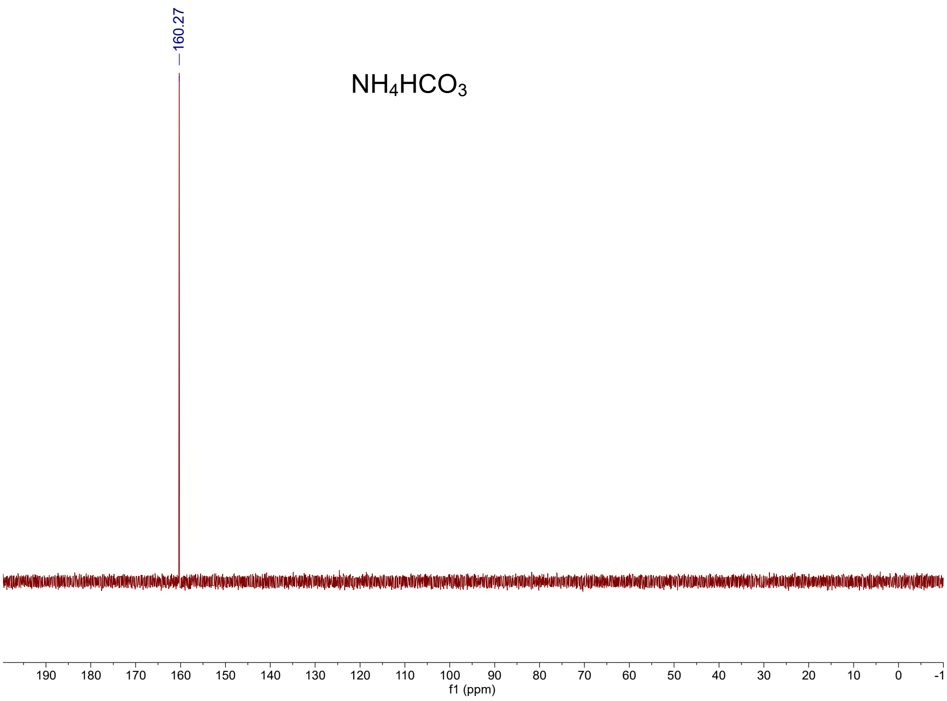

Fig. S1. ^{13}C spectrum of ammonium bicarbonate in D_2O (151 MHz, D_2O, D1=5 sec; NS=1024, 100 mM) δ 160.27.

Formation of the Hofmann-elimination product

^{1}H and ^{13}C NMR spectra of the isolated material revealed ~5% of an impurity that was separated by preparative HPLC. The impurity was identified as the Hofmann elimination product by 2D NMR analysis (for a structural depiction of the impurity, see Figure S2 below):

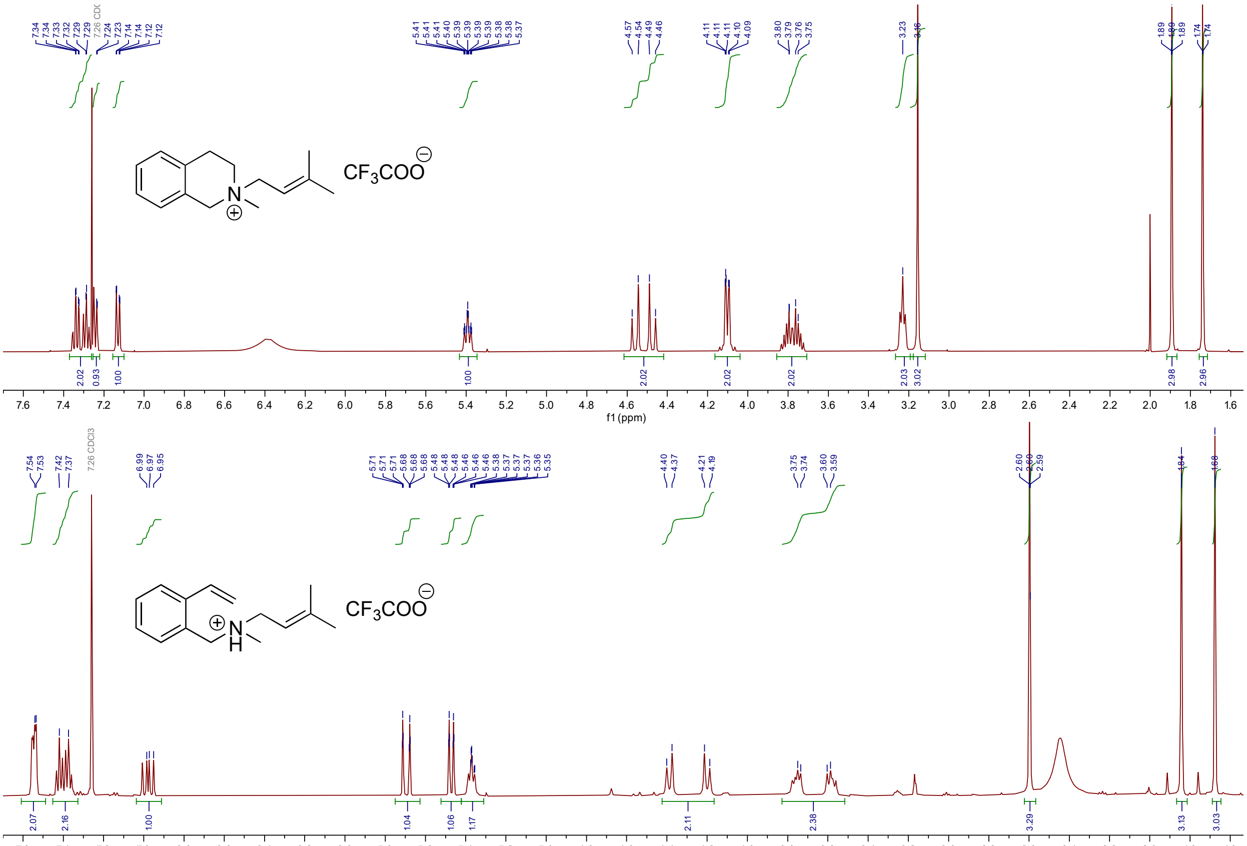

Fig. S2. Comparison of ^{1}H NMR spectra (500 MHz, $CDCl_3$) of the ammonium salt [**3aa**][CF_3CO_2] (top) and the Hofmann elimination product **S4** (bottom).

N,3-dimethyl-N-(2-vinylbenzyl)but-2-en-1-aminium trifluoroacetate (S4)

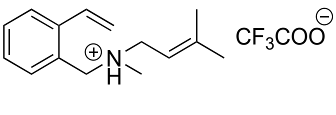

^{1}H NMR (500 MHz, $CDCl_3$) δ 7.51 – 7.44 (m, 2H), 7.35 (t, J = 7.4 Hz, 1H), 7.31 (t, J = 7.4 Hz, 1H), 6.91 (dd, J = 17.2, 10.9 Hz, 2H), 5.63 (d, J = 17.2 Hz, 1H), 5.40 (d, J = 10.9 Hz, 1H), 5.30 (t, J = 7.6 Hz, 1H), 4.23 (AB spin system, $δ_A$ = 4.32, $δ_B$ = 4.13, J_{AB} = 13.3 Hz, 2H), 3.74 – 3.63 (m, 1H), 3.56 – 3.45 (m, 1H), 2.53 (br.s, 3H), 1.78 (br.s, 3H), 1.61 (br.s, 3H); ^{13}C NMR (126 MHz, $CDCl_3$) δ 144.8, 139.0, 133.4, 131.9, 130.4, 128.9, 127.3, 126.4, 119.2, 112.1, 54.4, 53.0, 38.1, 26.1, 18.4; HRMS (ESI) m/z: [M + H]$^+$ Calcd for $C_{15}H_{22}N$ 216.1747; Found 216.1750.

Work-up under addition of volatile basic buffer

To isolate the pure ammonium salt from the reaction mixture, a volatile buffer (NH_4OAc/NH_3) was added for work-up.

2-methyl-2-(3-methylbut-2-en-1-yl)-1,2,3,4-tetrahydroisoquinolin-2-ium acetate ([3aa][OAc])

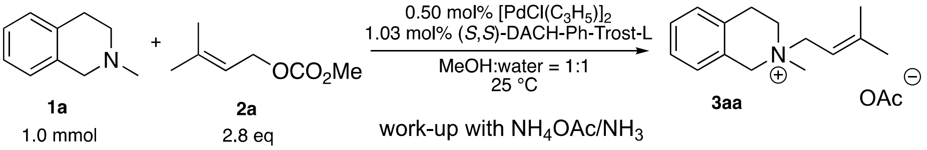

The preparative reaction on a 1.0 mmol scale was repeated. Allylpalladium chloride dimer (100 µL of 50.0 mM solution in CH_2Cl_2) and (*S*,*S*)-DACH-phenyl Trost ligand (200 µL of a 51.5 mM solution in CH_2Cl_2) were stirred for 10 minutes in methanol (46.5 mL) inside a glovebox under a nitrogen atmosphere. Methyl (3-methylbut-2-en-1-yl) carbonate (406 mg, 2.82 mmol) was added and stirring continued for 5 minutes before water (49.5 mL) was added, followed by 2-methyl-1,2,3,4-tetrahydroisoquinoline (148 mg, 1.01 mmol). The reaction mixture was left stirring vigorously at room temperature in the glovebox.

After 23 hours, the flask was removed from the glovebox, exposed to air and the solvents were evaporated under reduced pressure at a rotary evaporator (water bath 35 °C). HPLC-analysis showed 96% conversion and an er of 77:23. The crude material was dissolved in ammonium acetate buffer (100 mM, 120 mL; pH = 9.85; prepared by mixing 0.64 g of NH_4OAc and 0.70 mL of ~32% NH_3 solution in 200 mL of miliQ water) and the aqueous phase was washed with TBME (2 ×25 mL). Phase separation was supported by brief centrifugation. The aqueous fraction was frozen in liquid nitrogen and lyophilized. The obtained material was dissolved in acetonitrile (6 mL), the solution was filtered through a syringe filter; volatiles were removed under reduced pressure and the residue was dried at the Schlenk line overnight. Since the mass exceeded 100% yield, the product was redissolved in water (appr. 6 mL), frozen in liquid nitrogen and lyophilized overnight (repeated twice). The product was isolated as the acetate salt (237 mg, 82%, NMR purity 95%, *er* 77:23). Impurities: 4% [NH_4][OAc], <1% **1a**, traces of solvents.

^{1}H NMR (500 MHz, DMSO-d_6) δ 7.38 – 7.26 (m, 3H), 7.20 (d, J = 7.6 Hz, 1H), 5.49 (m, 1H), 4.58 (AB spin system, $δ_A$ = 4.62, $δ_B$ = 4.54, J_{AB} = 15.4 Hz, 2H), 4.09 – 3.97 (m, 2H), 3.74 – 3.64 (m, 2H), 3.17 (t, J = 6.6 Hz, 2H), 2.98 (s, 3H), 1.85 (br.s, 3H), 1.72 (d, J = 1.4 Hz, 3H), 1.64 (s, 3H); ^{13}C NMR (126 MHz, DMSO-d_6) δ 172.8, 146.6, 129.9, 128.8, 128.1, 127.2, 127.1, 127.0, 111.2, 60.1, 59.8, 56.0, 46.1, 26.0, 24.5, 23.1, 18.3; HRMS (ESI) m/z: [M]$^+$ Calcd for

$C_{15}H_{22}N$ 216.1747; Found 216.1750; HPLC (OX-H, ethanol/n-heptane/Et_3N/CF_3CO_2H = 20/80/0.5/0.3, flow rate = 1.0 mL/min, l = 220 nm) tR = 26.1 min (major), 27.7 min (minor).

Reaction monitoring of catalysis by NMR or reactions in D_2O/MeOD-d_4 showed no formation of the Hofmann-elimination product during the reaction (see Section *Further experiments to investigate side product formation* for details). Work-up with water (without NH_4OAc/NH_3 buffer) but no heating during the evaporation still showed the elimination product in the isolated material.

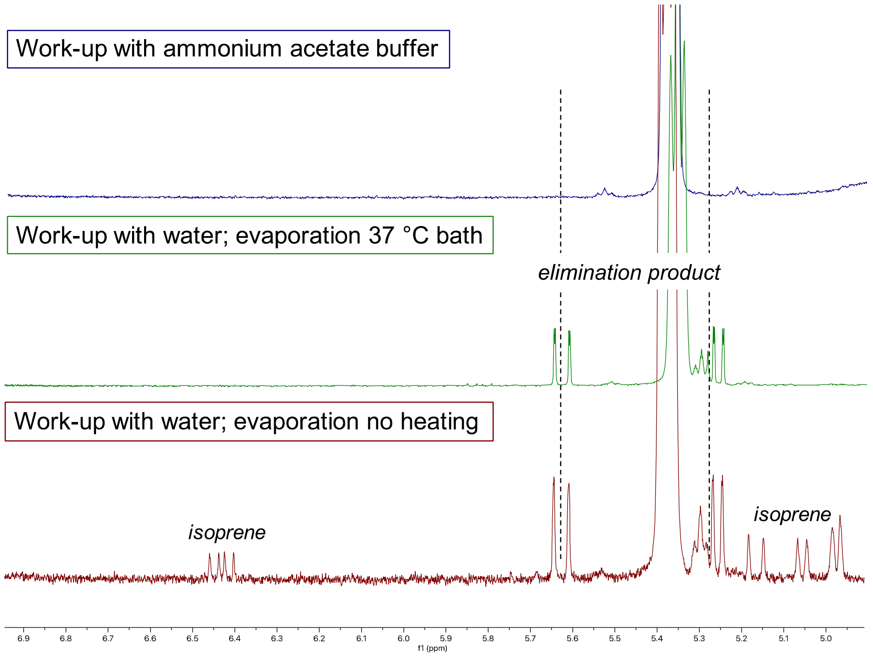

Fig. S3. Comparison of ^{1}H NMR (500 MHz, $CDCl_3$) spectra of ammonium salt **3aa**, isolated from catalytic reaction runs on a preparative scale obtained under three different work-up conditions (top – 0.26 mmol, work-up under addition of ammonium acetate buffer; middle – 1.00 mmol, work-up without additional buffer and evaporation of MeOH at 37 ° bath temperature of the rotary evaporator; bottom – 0.26 mmol scale, work-up without additional buffer and evaporation of MeOH without heating of the rotary evaporator bath, room temperature ~25 °C).

Following the same procedure (work-up under addition of volatile basic buffer), preparative scale reactions were run for:

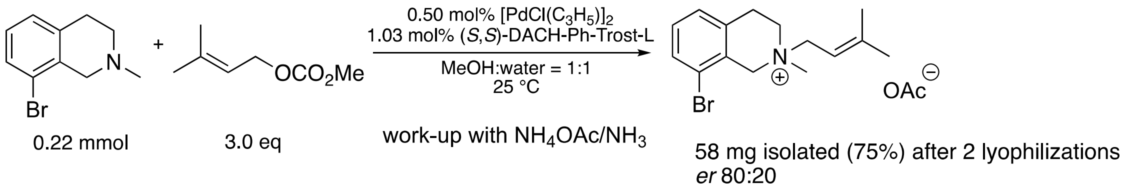

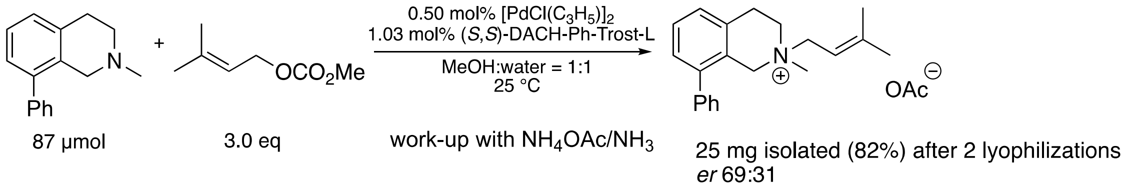

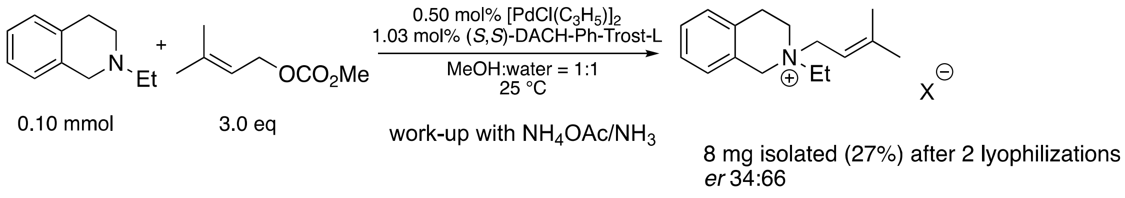

*8-bromo-2-methyl-2-(3-methylbut-2-en-yl)-1,2,3,4-tetrahydroisoquinolin-2-ium acetate ([**3ea**][OAc])*

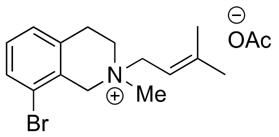

Viscous yellow oil (58 mg, 75% with respect to 0.22 mmol of the starting amine, *er* 80:20); ^{1}H NMR (500 MHz, $CDCl_3$) δ 7.51 (dd, *J* = 7.1, 2.0 Hz, 1H), 7.25 – 7.19 (m, 2H), 5.43 (m, 1H), 4.55 – 4.48 (m, 2H), 4.38 (d, *J* = 16.1 Hz, 1H), 4.35 – 4.30 (m, 1H), 4.19 (dd, *J* = 13.3, 8.5 Hz, 1H), 4.13 (dt, *J* = 12.4, 6.5 Hz, 1H), 3.40 (s, 3H), 3.37 – 3.26 (m, 2H), 1.95 (s, 3H), 1.89 (br.s, 3H), 1.72 (d, *J* = 1.3 Hz, 3H); ^{1}H NMR (500 MHz, DMSO-d_6) δ 7.62 (dd, *J* = 7.8, 1.3 Hz, 1H), 7.37 (dd, *J* = 7.8, 1.2 Hz, 1H), 7.31 (t, *J* = 7.8 Hz, 1H), 5.49 (m, 1H), 4.59 – 4.50 (m, 2H), 4.12 (dd, *J* = 13.4, 8.6 Hz, 1H), 4.00 (dd, *J* = 13.4, 7.5 Hz, 1H), 3.68 – 3.59 (m, 2H), 3.22 (t, *J* = 6.6 Hz, 2H), 3.04 (s, 3H), 1.86 (br.s, 3H), 1.70 (d, *J* = 0.8 Hz, 3H), 1.53 (s, 3H); ^{13}C NMR (126 MHz, $CDCl_3$) δ 176.4, 149.9, 132.6, 131.8, 130.2, 128.5, 126.0, 122.8, 110.3, 60.5, 60.3, 57.2, 48.5, 26.7, 24.3, 23.2, 18.6; HRMS (ESI) m/z: $[M]^+$ Calcd for $C_{15}H_{21}BrN$ 294.0852; Found 294.0857; HPLC (OX-H, ethanol/n-heptane/Et_3N/CF_3CO_2H = 20/80/0.5/0.3, flow rate = 1.0 mL/min, l = 220 nm) tR = 30.0 min (major), 39.5 min (minor).

*2-methyl-2-(3-methylbut-2-en-1-yl)-8-phenyl-1,2,3,4-tetrahydroisoquinolin-2-ium acetate ([**3ka**][OAc])*

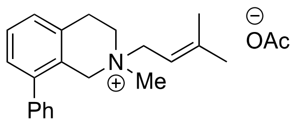

Viscous colorless oil (25 mg, 82% with respect to 87 µmol of the starting amine, *er* 69:31); ^{1}H NMR (500 MHz, $CDCl_3$) δ 7.46 – 7.41 (m, 2H), 7.41 – 7.38 (m, 1H), 7.35 (t, *J* = 7.7 Hz, 1H), 7.26 – 7.22 (m, 3H), 7.18 (dd, *J* = 7.6, 1.3 Hz, 1H), 5.16 (m, *J* = 8.1, 1.2 Hz, 1H), 4.36 (AB spin system, $δ_A$ = 4.38, $δ_B$ = 4.33, J_{AB} = 15.4 Hz, 2H), 4.31-4.25 (m, 2H), 4.15 (dt, *J* = 12.6, 6.5 Hz, 1H), 4.00 (dd, *J* = 13.3, 8.6 Hz, 1H), 3.33 (t, *J* = 6.7 Hz, 2H), 3.22 (s, 3H), 1.90 (s, 3H), 1.71 (d, *J* = 1.3 Hz, 3H), 1.56 (d, *J* = 1.3 Hz, 3H); ^{1}H NMR (500 MHz, DMSO-d_6) δ 7.53 – 7.48 (m, 2H), 7.47 – 7.41 (m, 2H), 7.36 (d, *J* = 7.7 Hz, 1H), 7.33 – 7.29 (m, 2H), 7.21 (dd, *J* = 7.5, 1.3 Hz, 1H), 5.35 (t, *J* = 8.0 Hz, 1H), 4.42 (AB spin system, $δ_A$ = 4.46, , $δ_B$ = 4.37, *J* = 15.5 Hz, 2H), 3.97 (dd, *J* = 13.4, 8.9 Hz, 1H), 3.86 (dd, *J* = 13.4, 7.2 Hz, 1H), 3.64 (t, *J* = 6.6 Hz, 2H), 2.94 (s, 3H), 1.75 (br.s, 3H), 1.58 (d, *J* = 1.3 Hz, 3H), 1.52 (s, 3H); ^{13}C NMR (126 MHz, $CDCl_3$) δ 176.4, 148.9, 141.5, 138.6, 130.0, 129.1, 128.9, 128.8, 128.7, 128.3, 128.2, 123.9, 110.3, 59.6, 59.2, 57.1, 48.1, 26.5, 24.3, 23.4, 18.6; HRMS (ESI) m/z: $[M]^+$ Calcd for $C_{21}H_{26}N$ 292.2060; Found 292.2065; HPLC (OX-H, ethanol/n-heptane/Et_3N/CF_3CO_2H = 20/80/0.5/0.3, flow rate = 1.0 mL/min, l = 220 nm) tR = 30.9 min (major), 38.6 min (minor).
Racemic standard to establish chiral phase HPLC analysis was prepared in an analytical scale catalysis run with an equimolar mixture of (*R*,*R*)- and (*S*,*S*)-DACH-phenyl Trost ligand.

*2-ethyl-2-(3-methylbut-2-en-1-yl)-1,2,3,4-tetrahydroisoquinolin-2-ium acetate (**3ra**)*

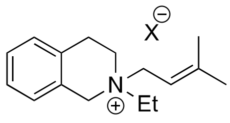

Viscous oil (8 mg, 27% with respect to 0.10 mmol of the starting amine, *er* 34:66; calculated for X = acetate); ^{1}H NMR (500 MHz, $CDCl_3$) δ 7.28 (t, *J* = 7.5 Hz, 1H), 7.23 (t, *J* = 7.5 Hz, 1H), 7.19 (d, *J* = 7.6 Hz, 1H), 7.13 (d, *J* = 7.6 Hz, 1H), 5.32 (t, *J* = 8.0 Hz, 1H), 4.73 (AB spin system, $δ_A$ = 4.76, $δ_B$ = 4.68, J_{AB} = 15.3 Hz, 2H), 4.29 – 4.17 (m, 2H), 4.11 – 4.03 (m, 1H), 3.98 – 3.90 (m, 1H), 3.61 (m, 2H), 3.24 – 3.12 (m, 2H), 1.88 (s, 3H), 1.85 (d, *J* = 1.4 Hz, 3H), 1.73 (d, *J* = 1.4 Hz, 3H), 1.42 (t, *J* = 7.3 Hz, 3H); ^{1}H NMR (500 MHz, DMSO-d_6) δ 7.36 – 7.29 (m, 3H),

7.25 – 7.19 (m, 1H), 5.44 (m, J = 7.7, 1.2 Hz, 1H), 4.52 (s, 2H), 4.02 – 3.88 (m, 2H), 3.72 – 3.63 (m, 2H), 3.32 – 3.24 (m, 2H), 3.17 – 3.12 (m, 1H), 1.84 (s, 3H), 1.69 (d, J = 1.3 Hz, 3H), 1.59 (s, 3H), 1.29 (t, J = 7.2 Hz, 3H); ^{13}C NMR (126 MHz, $CDCl_3$) δ 177.2, 148.7, 129.5, 128.9, 128.9, 127.8, 127.7, 126.6, 110.2, 58.9, 56.4, 54.4, 53.5, 26.7, 24.9, 23.8, 19.2, 8.2; The integral of the CH_3-protons of the acetate counter anion in ^{1}H NMR spectrum accounted only for ca. 58% of the required counterion equivalent. The remainder is likely bicarbonate; HRMS (ESI) m/z: $[M]^+$ Calcd for $C_{16}H_{24}N$ 230.1903; Found 230.1907; HPLC (OX-H, ethanol/n-heptane/Et_3N/CF_3CO_2H = 20/80/0.5/0.3, flow rate = 1.0 mL/min, l = 220 nm) tR = 30.5 min (minor), 32.3 min (major).

Racemic standard to establish chiral phase HPLC analysis was prepared in an analytical scale catalysis run with an equimolar mixture of (*R*,*R*)- and (*S*,*S*)-DACH-phenyl Trost ligand.

Optimization of conditions
Leaving group

Various parameters were optimized either independently or in combination.

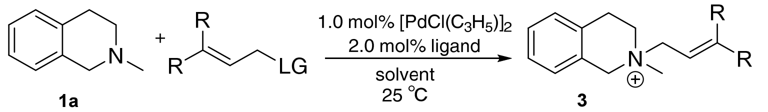

1a **3**

100 mM **1.5 eq**

L1 = (*R*,*R*)-DIOP; L2 = (*R*,*R*)-Chiraphos; L3 = (*S*,*S*)-DACH-Ph-Trost

Table S2. Leaving group optimization

	**R, LG**	**solvent**	**catalyst**[a]	**HPLC-yield**[b]	***er***[c]
1	R = H; LG = **OTs**	CH_2Cl_2	no Pd, no L	84% (4h)	50:50
			Pd, L1	86% (4h)	50:50
			Pd, L2	86% (4h)	50:50
2	R = H; LG = **OMs**	CH_2Cl_2	no Pd, no L	77% (4h)	50:50
			Pd, L1	90% (4h)	50:50
			Pd, L2	75% (4h)	50:50
3	R = H; LG = **OAc**	CH_2Cl_2	no Pd, no L	0% (24-48h)	
			Pd, L1	0% (24-48h)	n.a.[d]
			Pd, L2	0% (24-48h)	
4	R = H; LG = **OBz**	EtOH[e]	no Pd, no L	1% (72h)	n.a.
			Pd, L1	65% (72h)	50:50
			Pd, L2	8% (72h)	50:50
5	R = H; LG = **OCO₂Me**	MeCN	no Pd, no L	0% (17h)	n.a.
			Pd, L1	44% (17h)	50:50
			Pd, L2	1% (17h)	n.a.
6	R = Me; LG = **OAc**	MeOH	no Pd, no L	0% (6 days)	n.a.
			Pd, L1/L2	0% (6 days)	
7	R = Me; LG= **OBz**	EtOH	no Pd, no L	0% (6 days)	n.a.
			Pd, L1/L2	0% (6 days)	
8	R = Me; LG = **OAc**	EtOH	no Pd, no L	0% (5h)	n.a.
			Pd, L3	13% (5h)	58:42
9	R = Me; LG = **OBz**[e]	EtOH	no Pd, no L	0% (5h)	n.a.
			Pd, L3	18% (5h)	63:37
10	R = Me; LG = **OCO₂Me**	EtOH	no Pd, no L	0% (4 days)	n.a.
			Pd, L1/L2	0% (4 days)	n.a.
			Pd, L3	31% (5h)	73:24

[a] 'Pd' = allylpalladium(II) chloride dimer; 'L1' = (*R*,*R*)-DIOP; 'L2' = (*R*,*R*)-Chiraphos; 'L3' = (*S*,*S*)-DACH-Ph-Trost;
[b] The HPLC-yield was determined by reversed phase HPLC, integrated at 210 nm; an experimentally determined response factor for the amine/ammonium salt was used for entries 1-3 and 6-10; benzyl alcohol was used as an internal standard and product concentration was determined by the calibration curve for entries 4,5;
[c] *er* of **3** was determined by normal phase chiral HPLC, integrated at 220 nm;
[d] not applicable;
[e] reaction was set with 50 mM Nu.

Deviations from the procedure described for the analytical scale catalysis:

**entry**	**E and Nu used as**	**Pd and L stock, pipetting volume**
1, 2, 3	stock solutions in CH_2Cl_2	50+50 µL
4	neat material (1.6 eq of E)	50+50 µL (10% v/v CH_2Cl_2)
5	neat material (1.6 eq of E)	20+50 µL (7% v/v CH_2Cl_2)
6, 7	neat material (1.6 eq of E)	20+20 µL (4% v/v CH_2Cl_2)
8, 10	neat material	10+25 µL (3.5% v/v CH_2Cl_2)
9	neat material	40+40 µL(8% v/v CH_2Cl_2)

Screening of solvents
with unsubstituted allyl acetate as the electrophile

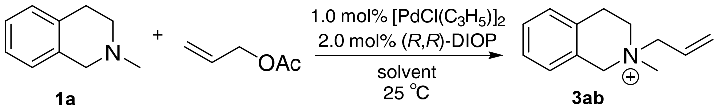

Table S3. Screening of solvents: unsubstituted allyl acetate

	solvent	Nu conc., mM	HPLC-yield (reaction time 1)[a]	HPLC-yield (reaction time 2)
1	Et_2O[b]	70	0% (20h)	0% (40h)
2	Acetone	50	0% (18h)	0% (45h)
3	CH_2Cl_2[b]	70	0% (20h)	0% (40h)
4	MeCN	50	2% (18h)	3% (45h)
5	MeCN:H_2O (1:1)	50	62% (18h)	76% (45h)
6	MeOH	50	27% (18h)	57% (45h)
7	EtOH	50	57% (18h)	72% (45h)
8	*i*PrOH	50	35% (18h)	39% (45h)
9	$(CF_3)_2CHOH$	50	0% (18h)	0% (45h)
10	EtOH:H_2O (1:1)	50	60% (18h)	63% (45h)
11	DMF[b]	70	4% (20h)	4% (40h)
12	DMA[b]	70	2% (20h)	4% (40h)
13	DMSO[b]	70	14% (20h)	26% (40h)

[a] The HPLC-yield was determined by reversed phase HPLC (210 nm) under consideration of the experimentally determined response factor; [b] reactions were run on a 1.4 mL scale.

Deviations from the procedure described for the analytical scale catalysis (E = allyl acetate, Nu = 2-methyl-1,2,3,4-tetrahydroisoquinoline):

entry	E and Nu added as	Pd and L stock, pipetting volume
1, 3, 11, 12, 13	stock solutions in CH_2Cl_2	50+50 µL (18% v/v CH_2Cl_2, E and Nu stocks)
2, 4, 5–10	neat material	20+20 µL (4% v/v CH_2Cl_2)

*with **2a** as the electrophile*

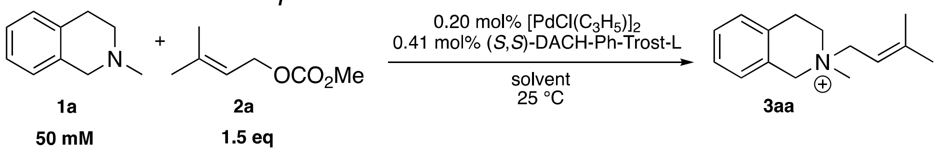

Table S4. Screening of solvents: **2a** as electrophile

	solvent	HPLC-yield[a] (reaction time)	*er*[b]
1	Et_2O	0% (21h)	n.a.[c]
2	Acetone	0% (21h)	n.a.
3	Dioxane	0% (21h)	n.a.
4	MeCN	1% (21h)<br>5% (5h, at 60 °C)	n.a.<br>64:36
5	THF	0% (21h)<br>0% (23h, at 60 °C)	n.a.
6	Toluene	0% (21h)<br>0% (23h, at 60 °C)	n.a.
7	DMF[d]	12% (23h, at 60 °C)	58:42
8	DMSO[d]	43% (23h, at 60 °C)	62:38
9	MeOH	7% (1h)<br>72% (25h)	75:25<br>73:27
10	EtOH	6% (1h)<br>40% (25h)	76:24<br>74:26
11	*i*PrOH	2% (25h)<br>3% (23h, at 60 °C)	75:25<br>60:40
12	$(CF_3)_2CHOH$	0% (25h)	n.a.
13	*n*-BuOH	3% (1h)<br>13% (23h)	75:25<br>73:24
14	*sec*-BuOH	0% (25h)	n.a.
15	Cyclohexanol	0% (25h)	n.a.
16	Et_2O : water = 1:1[d]	4% (23h)	53:47
17	Toluene : water = 1:1[d]	2% (23h)	60:40
18	CH_2Cl_2 : water = 1:1[d]	3% (23h)	45:55

[a] The HPLC-yield was determined by reversed phase HPLC (210 nm); benzyl alcohol was used as an internal standard and product concentration was determined by the calibration curve;
[b] the *er* of **3aa** was determined by normal phase chiral HPLC, integrated at 220 nm;
[c] not applicable;
[d] 0.5 mol% $[Pd(C_3H_5)Cl]_2$ and 1.03 mol% (*S*,*S*)-DACH-Ph-Trost-L used.

Deviations from the procedure described for analytical scale catalysis:
Allyl electrophile and tertiary amine nucleophile were added as neat materials;

entry	Pd and L stock, pipetting volume (final volume percent CH_2Cl_2 in reaction)
1 – 6, 9 – 15	8+10 µL (1.8% v/v CH_2Cl_2 total)
7, 8	20+20 µL (4% v/v CH_2Cl_2)
16 – 18	8+8 µL (4% v/v CH_2Cl_2, total volume is 0.4 mL)

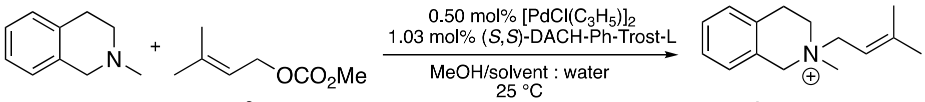

1a **10 mM** **2a** **2.8 eq** **3aa**

Table S5. Screening of solvents: **2a** as electrophile with 10 mM Nu

**Solvent**	**1h reaction time** HPLC-yield[a]	*er*[b]	**24h reaction time** HPLC-yield	*er*
methanol	3%	70:30	63%	75:25
methanol : water 9:1	9%	75:25	80%	76:24
methanol : water 4:1	17%	76:24	92%	76:24
methanol : water 3:1	20%	77:23	91%	77:23
methanol : water 2:1	25%	78:22	96%	78:22
methanol : water 1:1	23%	78:22	96%	78:22
methanol : water 1:2	26%	77:23	88%	77:23
methanol : water 1:3	6%	70:30	23%	67:33
THF : water 1:1	2%	n.a.[c]	10%	53:47
acetone : water 1:1	36%	74:26	95%	74:26
MeCN : water 1:1	19%	70:30	90%	69:31

Amine nucleophile and allyl electrophile were used as neat materials; 1% v/v CH_2Cl_2 final concentration in the reaction mixtures.
[a The HPLC-yield was determined by reversed phase HPLC (210 nm); benzyl alcohol was used as an internal standard and product concentration was determined by the calibration curve;
[b] *er* of **3aa** was determined by chiral phase HPLC, integrated at 220 nm;
[c] not applicable.

Screening of various nucleophile and electrophile concentrations and ratios

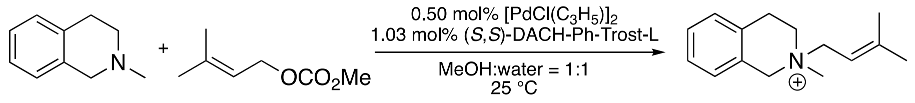

1a **10-25 mM**   **2a** **1.5-7.0 eq**   **3aa**

Table S6. Screening of nucleophile concentrations with different electrophile loadings

Nu conc, mM	E excess, eq	**reaction time 1h** HPLC-yield[a]	*er*[b]	**reaction time 22h** HPLC-yield	*er*	**reaction time 45h** HPLC-yield	*er*
10	1.5	30%	78.1:21.9	93%	77.8:22.2	96%	77.8:22.2
10	3.0	29%	78.2:21.2	96%	77.9:22.1	99%	78.0:22.0
10	5.0	31%	77.9:22.1	95%	77.8:22.2	99%	78.1:21.9
10	7.0	34%	77.8:22.2	93%	77.7:22.3	99%	77.9:22.1
15	1.5	30%	78.3:21.7	92%	77.7:22.3	96%	77.8:22.3
15	3.0	36%	77.7:23.3	94%	77.5:22.5	99%	77.5:22.5
15	5.0	36%	77.5:22.5	94%	77.3:22.7	97%	77.7:22.3
20	3.0	40%	77.1:22.9	94%	76.8:23.2	99%	77.1:22.9
25	1.5	35%	77.1:22.9	93%	76.7:23.3	99%	76.8:23.2
25	3.0	37%	76.5:23.5	93%	76.3:23.7	96%	76.5:23.6
25	5.0	33%	76.6:23.4	88%	76.5:23.5	92%	76.0:24.0

Amine nucleophile and allyl electrophile were used as 1M stock solutions in MeOH; The total volume percent (% v/v) of CH_2Cl_2 in the reaction mixture varied in accordance with the nucleophile concentration (variation between 0.8% v/v CH_2Cl_2 for 10 mM **1a** and 2.0% v/v CH_2Cl_2 for 25 mM **1a**)
[a] The HPLC-yield was determined by reversed phase HPLC (210 nm), considering the experimentally determined response factor;
[b] *er* of **3aa** was determined by normal phase chiral HPLC, integrated at 220 nm; The higher precision (3 significant digits) indicated here serves only to track trends.

Higher nucleophile concentrations:

Table S7. Screening of higher nucleophile concentrations

Nu conc, mM	E excess, eq	**5h** HPLC-yield[a]	*er*[b]
30	1.5	67%	75.4:24.6
30	5.3	61%	75.5:25.5
30	9.0	47%	75.8:24.3
50	5.3	40%	71.8:28.2

Amine nucleophile and allyl electrophile were used as 1M stock solutions in MeOH; The total volume percent (% v/v) of CH_2Cl_2 in the reaction mixture was 2.4% v/v CH_2Cl_2 for the reactions with 30 mM **1a** and 4.0% v/v for the reactions with 50 mM **1a**.
[a] The HPLC-yield was determined by reversed phase HPLC (210 nm), considering the experimentally determined response factor;
[b] *er* of **3aa** was determined by normal phase chiral HPLC, integrated at 220 nm; The higher precision (3 significant digits) indicated here serves only to track trends.

Ligand screening

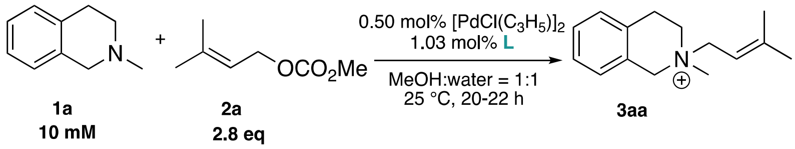

1a **10 mM** **2a** **2.8 eq** **3aa**

Table S8. Ligands screening

Name	Structure	Optimized conditions		Various conditions[a]		Various conditions[a]	
		**Electrophile** 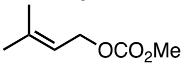		**Electrophile** 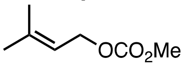		**Electrophile** 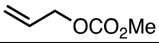	
		**HPLC-y.**[b]**, %**	***er***[b]	**HPLC-y., %**	***er***	**HPLC-y., %**	***er***
(1*S*,1'*S*,2*R*,2'*R*) DuanPhos CAS 795290-34-5	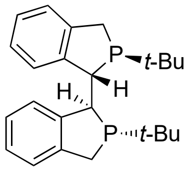	0	n.a.[c]	n.d.[c]	n.d.	0[d]	n.a.
(*R*)-SolPhos CAS 649559-68-2	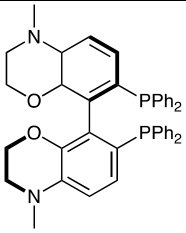	4	47:53	n.d.	n.d.	n.d.	n.d.
(*S*)-BINAP CAS 76189-56-5	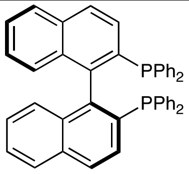	2	n.a.	n.d.	n.d.	10[d]	50:50
(*S*)-BINAPINE CAS 610304-81-9	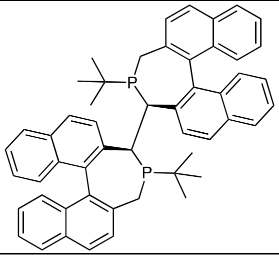	3	42:58	n.d.	n.d.	n.d.	n.d.
(*S*)-C3-TunePhos CAS 486429-99-6	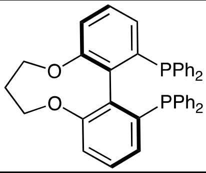	2	60:40	n.d.	n.d.	n.d.	n.d.
(*S*)-C4-TunePhos CAS 486429-94-1	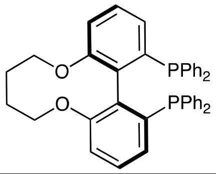	3	50:50	n.d.	n.d.	n.d.	n.d.
(*S*)-Me-f-KetalPhos CAS 488760-58-3	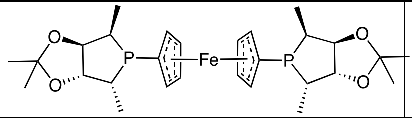	0	n.a.	0[e]	n.a.	8[d]	50:50

(*R*)-Ph-BPE<br>CAS 528565-79-9	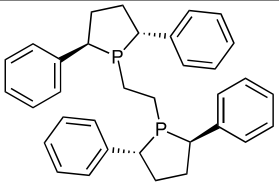	0	n.a.	n.d.	n.d.	n.d.	n.d.
(*R*,*R*)-DIOP<br>CAS 32305-98-9	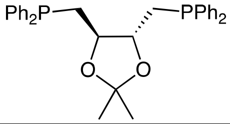	0	n.a.	0[f]	n.a.	65[g]	0
(*R*,*R*)-Skewphos<br>CAS 96183-46-9	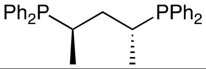	0	n.a.	n.d.	n.d.	7[d]	50:50
(*R*,*R*)-Chiraphos<br>CAS 74839-84-2	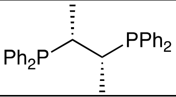	0	n.a.	0[f]	n.a.	2[g]	n.a.
(*S*,*S*)-DACH-Ph-Trost<br>CAS 169689-05-8	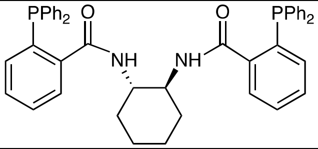	96	78:22	40[e]	74:26	100[h]	50:50
(*S*,*S*)-DACH-Naph Trost<br>CAS 205495-66-5	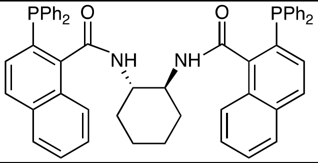	100	73:27	1[f]	47:53	n.d.	n.d.
(*S*,*S*)-ANDEN-Trost<br>CAS 152140-65-3	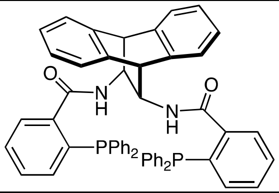	100	67:33	28[e]	55:45	91[e]	50:50
(*S*,*S*)-Stilbene-Trost<br>CAS 143668-57-9	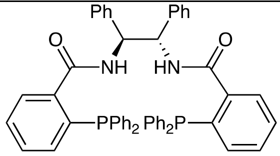	27	75:25	7[e]	72:28	70[e]	49:51
(*R*,*R*)-Trost-NH<br>CAS 1150113-65-7	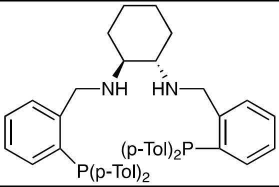	64	54:46	5[e]	n.a.	42[e]	49:51
(*S*)-iPr-PHOX<br>CAS 148461-14-7	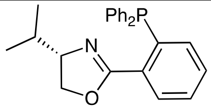	0	n.a.	0[e]	n.a.	4[d]	50:50
(*S*)-iPr-oxazoline<br>CAS 131833-92-6	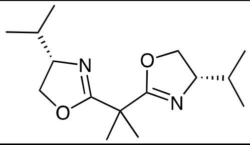	2	n.a.	0[e]	n.a.	2[d]	50:50
(*R*)-PHANEPHOS<br>CAS 364732-88-7	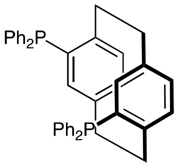	1	n.a.	0[e]	n.a.	11[e]	50:50

(*S*,*S*,*S*)-(-)-Ph-SKP<br>CAS 1439556-82-7	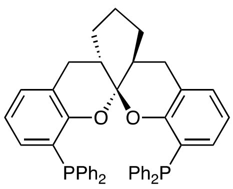	70	53:47	78[e]	50:50	89[e]	52:48
CAS 2119686-55-2	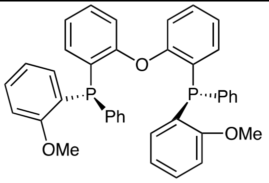	1	n.a.	0[e]	n.a.	n.d.	n.d.
(*R*)-SDP<br>CAS 917377-74-3	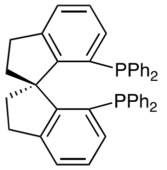	31	48:52	1[e]	49:51	n.d.	n.d.
(*S*,*S*)-BPPM<br>CAS 61478-28-2	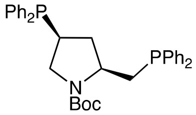	1	n.a.	0[e]	n.a.	n.d.	n.d.
(*R*)-SegPhos<br>CAS 244261-66-3	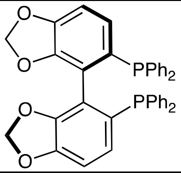	0	n.a.	n.d.	n.d.	n.d.	n.d.
(*S*)-DifluorPhos<br>CAS 503538-70-3	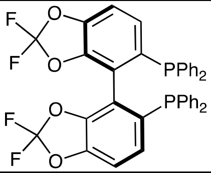	0	n.a.	n.d.	n.d.	n.d.	n.d.
Ph-JoyaPhos<br>**1.5 eq**<br>R = Ph	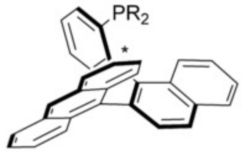	100	49:51	n.d.	n.d.	n.d.	n.d.
3,5-CF_3-JoyaPhos<br>**1.5 eq**<br>R= 3,5-$(CF_3)_2C_5H_3$	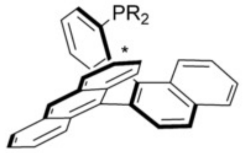	11	52:48	n.d.	n.d.	n.d.	n.d.
CF_3-PHOX<br>CAS 1006708-91-3	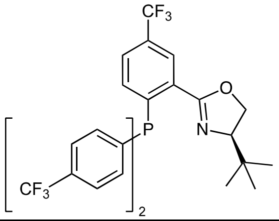	0	n.a.	n.d.	n.d.	n.d.	n.d.
CAS 565184-32-9	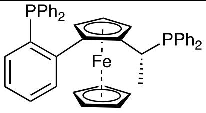	1	n.a.	n.d.	n.d.	n.d.	n.d.
CAS 565184-29-4	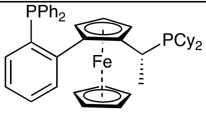	2	n.a.	n.d.	n.d.	n.d.	n.d.

(*R*)-Walphos-CF_3<br>CAS 387868-06-6	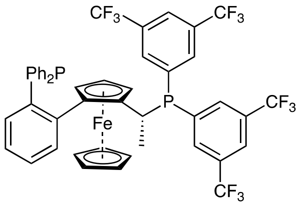	91	51:49	0[e]<br>(but 60% in 1h)	n.a.	57[e]	49:51
CAS 821009-34-1	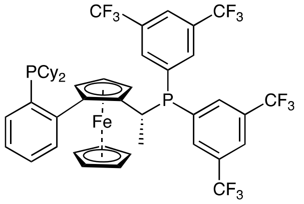	14	49:51	n.d.	n.d.	n.d.	n.d.
CAS 494227-30-4	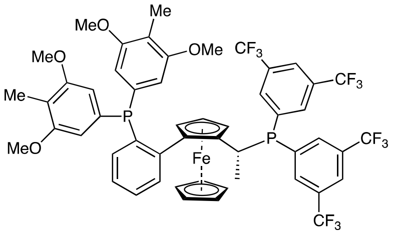	64	50:50	n.d.	n.d.	n.d.	n.d.
CAS 894771-28-9	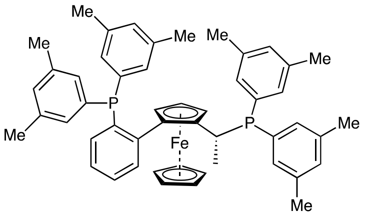	1	n.a.	n.d.	n.d.	n.d.	n.d.
(*S*)-NMDPP<br>**1 eq**<br>CAS 43077-29-8	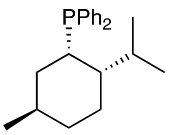	2	n.a.	n.d.	n.d.	n.d.	n.d.
(*S*)-NMDPP<br>**2 eq**[i]<br>CAS 43077-29-8		3	51:49	0[e]	n.a.	n.d.	n.d.
(*S*)-SITCP **1 eq**<br>CAS 885701-78-0	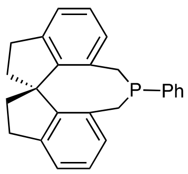	0	n.a.	n.d.	n.d.	n.d.	n.d.
(*S*)-SITCP **2 eq**[i]<br>CAS 885701-78-0		0	n.a.	0[e]	n.a.	3[e]	51:49
(*R*)-MOP **1eq**<br>CAS 145964-33-6	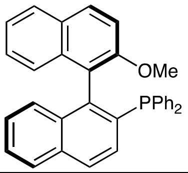	5	53:47	n.d.	n.d.	n.d.	n.d.
(*R*)-MOP **2eq**[i]<br>CAS 145964-33-6		15	48:52	n.d.	n.d.	n.d.	n.d.
(*S*)-BINEPINE-tBu<br>**1 eq**<br>CAS 475992-24-6	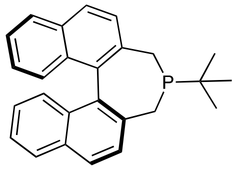	0	n.a.	n.d.	n.d.	n.d.	n.d.
(*S*)-BINEPINE-tBu<br>**2 eq**[i]<br>CAS 475992-24-6		0	n.a.	n.d.	n.d.	n.d.	n.d.
(*R*)-BINEPINE-Ph<br>**1 eq**<br>CAS 174390-35-3	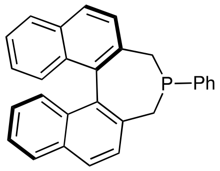	0	n.a.	n.d.	n.d.	n.d.	n.d.
(*R*)-BINEPINE-Ph<br>**2 eq**[i]<br>CAS 174390-35-3		0	n.a.	n.d.	n.d.	n.d.	n.d.

**1 eq** CAS 1835717-07-1	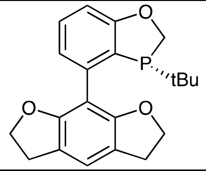	1	53:47	0[e]	n.a.	n.d.	n.d.
**2 eq**[i] CAS 1835717-07-1		4	60:40	n.d.	n.d.	98[e]	51:49

Conditions, if not indicated otherwise: 0.5% $[Pd(C_3H_5)Cl]_2$, 1.03% bidentate L (2.05 mol% for monodentate ligands), 10 mM Nu, 2.8 eq E in MeOH:water = 1:1. Both allyl methylcarbonates and amine nucleophile were added as neat materials; $[PdCl(C_3H_5)_2]$ and ligands were added as stock solutions in CH_2Cl_2. The reaction contained 1.8% v/v CH_2Cl_2.
[a] General: reactions were run with 50-100 mM amine nucleophile, 1.5 eq electrophile, 0.50-1.0 mol% $[PdCl(C_3H_5)]_2$; solvent = ethanol or acetonitrile; LG = methylcarbonate or benzoate; conditions are specified in further footnotes.
[b] The HPLC-yield was determined by reversed phase HPLC (210 nm) considering the experimentally determined response factor; *er* of **3aa** was determined by chiral phase HPLC (220 nm);
[c] n.a.=not applicable; n.d.=not determined;
[d] 100 mM Nu, LG = benzoate, 1.0 mol% $[PdCl(C_3H_5)]_2$, solvent = MeCN; the reactions contained 4 - 5% v/v CH_2Cl_2
[e] 50 mM Nu, LG = methylcarbonate, 0.50 mol% $[PdCl(C_3H_5)]_2$; solvent = ethanol;
[f] 100 mM Nu, LG = methylcarbonate, 0.50 mol% $[PdCl(C_3H_5)]_2$; solvent = ethanol; the reactions were carried out on a 0.5 mL scale;
[g] 100 mM Nu, LG = methylcarbonate, 1.0 mol% $[PdCl(C_3H_5)]_2$, solvent = MeCN; the reactions contained 7% v/v CH_2Cl_2.
[h] optimized conditions;
[i] 2.05 mol% of mono-P ligands were used.

Substrate screening

Electrophiles

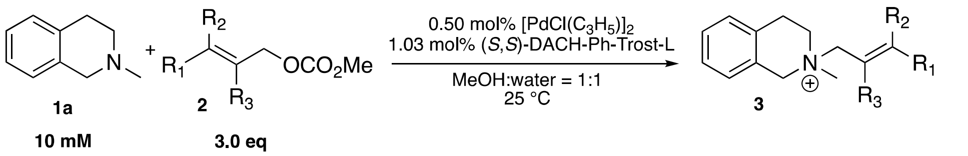

Table S9. Screening of electrophiles

	electrophile	HPLC-yield [a]<br>*er*[b]<br>reaction time 1h	HPLC-yield<br>*er*<br>reaction time 21-23h
1	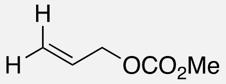	100%<br>*er* 50:50	100%<br>*er* 50:50
2[c]	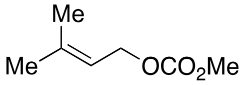	43%<br>*er* 77:23	96%<br>*er* 77:23
3	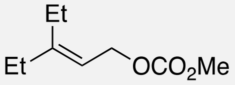	0%<br>*er* n.a.[d]	13%<br>*er* 70:30
4	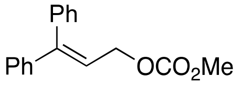	0%<br>*er* n.a.	2%<br>*er* n.a.
5[e]	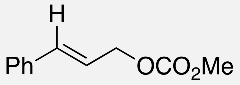	53%[f]<br>*er* 48:52	88%<br>*er* 48:52
6	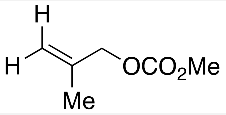	0%<br>*er* n.a.	16%<br>*er* 53:47
7	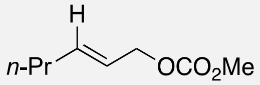	86%[g]<br>*er* 48:52	100%<br>*er* 47:53

[a] The HPLC-yield was determined by reversed phase HPLC, integrated at 210 nm, considering the experimentally determined response factor;
[b] *er* of **3** was determined by chiral phase HPLC, integrated at 220 nm;
[c] 2.8 eq of E used;
[d] not applicable;
[e] 3.5 eq of E used;
[f] time 1 = 4 min;
[g] time 1 = 10 min.

All reactions were run as duplicates except entry 4.

Nucleophiles

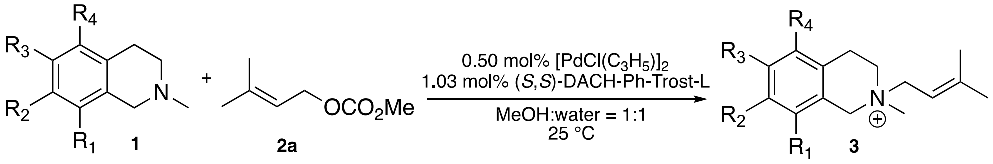

10 mM **2.8 eq**

Table S10. Screening of nucleophiles

	**nucleophile**	**HPLC-yield** [a]<br>***er*[b]**<br>**reaction time 1h**	**HPLC-yield**<br>***er***<br>**reaction time 20-22h**
1	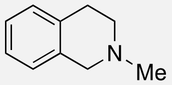	41%<br>*er* 77:23	96%; isol. yield 82%[c]<br>*er* 77:23
2	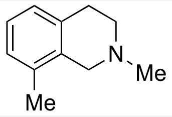	42%<br>*er* 81:19	97%<br>*er* 80:20
3	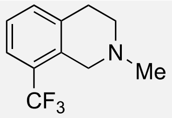	20%<br>*er* 73:27	81%<br>*er* 73:27
4	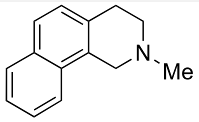	54%<br>*er* 79:21	99%<br>*er* 79:21
5	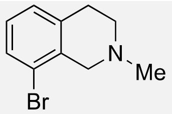	28%<br>*er* 80:20	84%; isol. yield 75%[c]<br>*er* 80:20
6	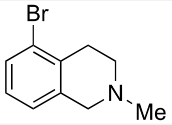	25%<br>*er* 74:26	92%<br>*er* 74:26
7	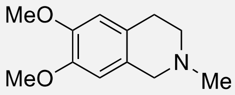	43%<br>*er* 78:22	98%<br>*er* 77:23
8	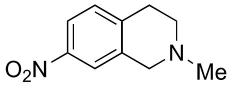	12%<br>*er* 71:29	71%<br>*er* 71:29
9	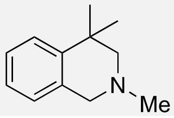	14%[d]<br>*er* 67:33	21%[d]<br>*er* 66:34
10	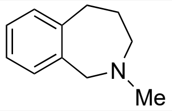	-[e]<br>er 49:51	86%[e]<br>er 49:51
11	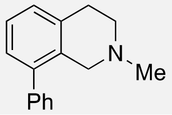	-[e]<br>*er* 69:31	87%[e]; isol. yield 80%[c]<br>*er* 69:31
12	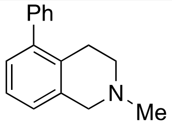	50%<br>*er* 15:85	99%<br>*er* 16:84

13	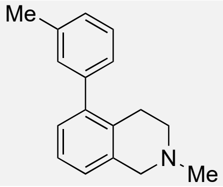	72%<br>*er* 12:88	>99%<br>*er* 12:88
14	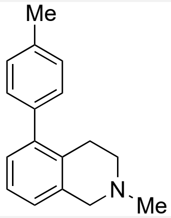	54%<br>*er* 84:16	99%<br>*er* 84:16
15	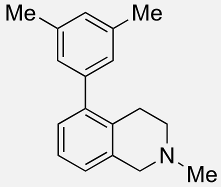	-[f]	99%[f]<br>*er* 10:90
16	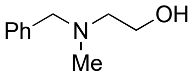	1%<br>*er* n.a.	11%<br>*er* 51:49
17	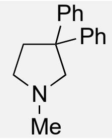	38%<br>*er* 49:51	83%<br>*er* 49:51
18	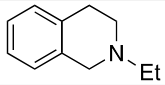	-[e]<br>*er* 34:66	29%[e]; isol. yield 27%[c]<br>*er* 34:66
19	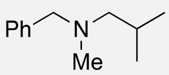	0%<br>*er* n.a.	0%<br>*er* n.a.
20	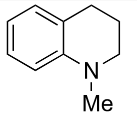	0%<br>*er* n.a.[g]	0%<br>*er* n.a.

For the entries 11 – 14 electrophile was added as a stock solution (300 mM, 3.0 eq) in degassed methanol, freshly prepared in the glovebox.
All the reactions were run in duplicates; 2 negative controls (only Pd, no L; no Pd, no L) were used for each Nu.
[a] The HPLC-yield was determined by reversed phase HPLC, integrated at 210 nm, considering the experimentally determined response factor;
[b] *ee* of **3** was determined by normal phase chiral HPLC, integrated at 220 nm;
[c] isolated yield reported for the ammonium salts in the form of acetate; entry 1 – 1.0 mmol scale, entry 4 – 0.22 mmol scale, entry 5 – 0.09 mmol scale and entry 15 – 0.10 mmol scale; for details see section *Catalysis, Preparative scale*;
[d] first result is for 20h, the second for 48h reaction time;
[e] ^{1}H NMR-yield after an overnight run (24h) with 1,3,5-trimethoxybenzene as the internal standard;
[f] HPLC-yield and *ee* after an overnight run (20h), reaction run at 20 degrees;
[g] not applicable.

Racemization studies

Preparation of enantioenriched material

Both unsubstituted und *gem*-dimethyl allyl ammonium salts were resolved by recrystallization (ca. 5 rounds) from methanol as diastereomeric salts with dibenzoyl-L-tartrate as the chiral anion (see *Synthesis* section):

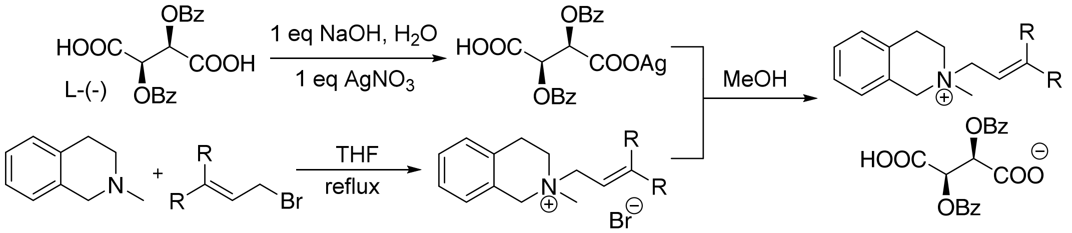

S2 (R = H)
S3 (R = CH_3)

The chiral anion was exchanged for trifluoroacetate for the racemization experiments (see Synthesis section).

Stock solutions in CH_2Cl_2 of $[Pd(C_3H_5)Cl]_2$ and (*S*,*S*)-DACH-Ph-Trost ligand were mixed for 10 min in degassed methanol. Subsequently, degassed water was added. The obtained solution was transferred to the weighed enantioenriched material placed in two vials and the mixtures were shaken until dissolution. Nucleophile **1a** (1 eq.) was quickly added to one of the vials. Total sum of volumes for each vial = 1000 µL (volume of solid not considered). First samples (50 µL each) were taken for chiral phase HPLC analysis at 'timepoint = 0h' (ca. 30 sec after the reactions started). The vials with the samples were immediately removed from the glovebox, exposed to air and volatiles were removed with a speedvac concentrator (45 °C, 50 min). The residue was then dissolved in EtOH/heptane (100 µL of 20/80 mixture) and submitted to chiral phase HPLC analysis.

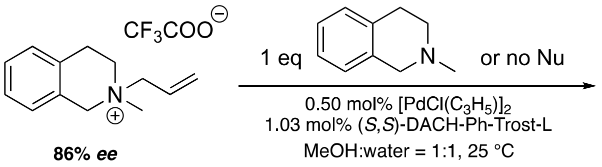

86% *ee*

10 mM

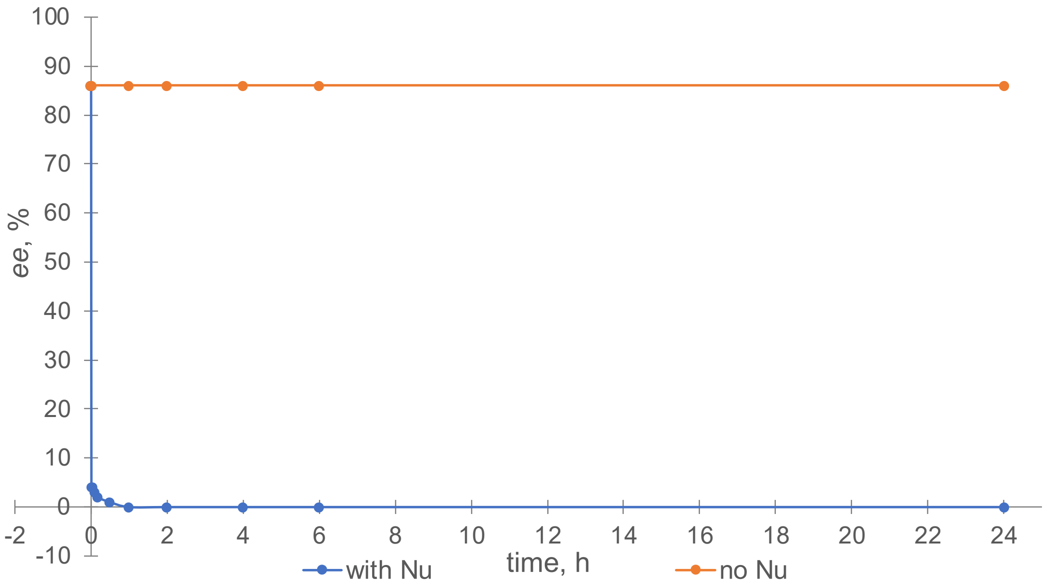

Fig.S4. Time-plot for the racemisation of allylammonium ion [**3ab**][CF_3COO] (10 mM in MeOH:H_2O = 1:1, 25°C) in the presence of $[PdCl(C_3H_5)]_2$ (0.50 mol%), (*S*,*S*)-DACH-Ph-Trost-L (1.03 mol%) and amine **1a** (10 mM), blue line; in the absence of the amine nucleophile the ammonium ion is configurationally stable, even in the presence of the Pd-precursor and chiral ligand, orange line. Individual data-points are indicated. The lines have been drawn to guide the eye.

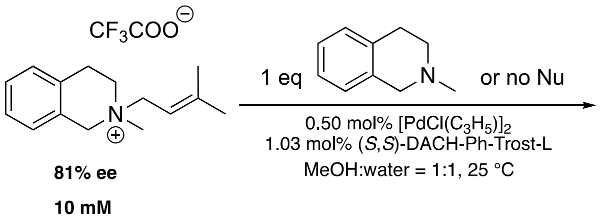

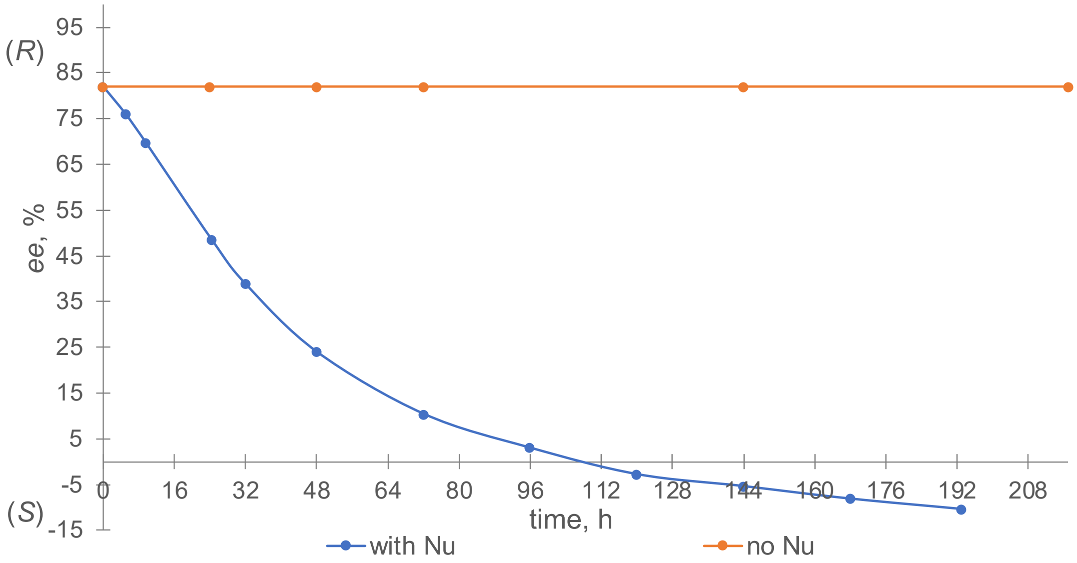

Fig. S5. Time-plot for the racemization of allylammonium ion [**3aa**][CF_3CO_2] (10 mM in MeOH:H_2O = 1:1, 25 °C) in the presence of [PdCl(C_3H_5)]$_2$ (0.50 mol%), (*S*,*S*)-DACH-Ph-Trost-L (1.03 mol%) and amine **1a** (10 mM), blue line; in the absence of the amine nucleophile the ammonium ion is configurationally stable, even in the presence of the Pd-precursor and chiral ligand, orange line. Individual data-points are indicated. The lines have been drawn to guide the eye. The apparent enrichment of the (*S*)-enantiomer after prolonged reaction times is likely due to the competing formation of isoprene via the corresponding Pd-allyl-complex which is formed in a kinetic resolution reaction from the allyl ammonium ion.

Further experiments to investigate side product formation

Isoprene formation

Stock solutions of $[Pd(C_3H_5)Cl]_2$ (37.4 mM) and ligands (76.9 mM) were freshly prepared in CD_2Cl_2; the remaining solvent was CD_3OD. CD_3OD and CD_2Cl_2 were degassed with 3 freeze-pump cycles. Reactants were mixed in HPLC vials in the glovebox on a 1.5 mL scale. Order of addition: CD_3OD, $[PdCl(C_3H_5)]_2$ and ligand stock solutions, followed by mixing for 10 min, allyl electrophile **2a** (33 µL, 0.23 mmol, 1.5 eq), followed by mixing for 5 min, and finally the amine nucleophile **1a** (22.0 µL, 0.15 mmol, 1.0 eq). Reactions were agitated in a thermoshaker (25 °C, 600 rpm) in the glovebox under a nitrogen atmosphere.
Aliquots were taken at the indicated time points and analyzed by ^1H NMR spectroscopy and HPLC. 50 µL aliquots of the reaction mixture were taken for HPLC analysis (diluted in 850 µL of MeOH for the determination of the HPLC-yield and in 650 µL of EtOH/Heptane 20/80 mixture for chiral phase analysis, respectively). 25 µL or 400 µL of the reaction mixture were taken for NMR analysis (diluted in 475 µL or 100 µL of CD_3OD for ^1H or ^{13}C and 2D NMR analysis, respectively).

The formation of isoprene was most pronounced when DACH-naphthyl Trost was employed as the ligand.

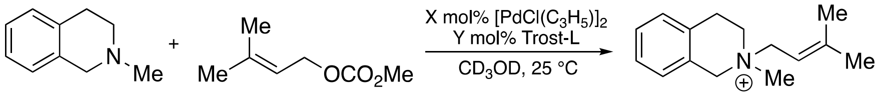

Table S11. Nucleophiles screening

	[Pd(allyl)Cl]$_2$	Ligand	HPLC-yield[a] (reaction time) *er* [b]	HPLC-yield (reaction time) *er*	HPLC-yield (reaction time) *er*	HPLC-yield (reaction time) *er*
1	0.50 mol%	(*S*,*S*)-DACH-**Ph**-Trost, 1.03 mol%	10% (45min) *er* 74:26	25% (2.5h) *er* 74:26	40% (5h) *er* 73:27	77% (23h) *er* 71:29
2	2.00 mol%	(*S*,*S*)-DACH-**Ph**-Trost, 4.10 mol%	19% (45min) *er* 72:23	44% (2.5h) *er* 71:29	65% (5h) *er* 70:30	77% (23h) *er* 61:39
3	5.00 mol%[c]	(*S*,*S*)-DACH-**Ph**-Trost, 10.3 mol%	19% (45min) *er* 66:34	44% (2.5h) *er* 64:36	66% (5h) *er* 63:37	94% (23h) *er* 59:41
4	0.50 mol%	(*S*,*S*)-DACH-**Naph**-Trost, 1.03 mol%	31% (45min) *er* 61:39	73% (2.5h) *er* 59:41	83% (5h) *er* 55:45	2% (23h) *er* n.d.[d]

[a] The HPLC-yield was determined by reversed phase HPLC (210 nm) under consideration of the experimentally determined response factor amine/ammonium salt.
[b] *er* of **3a** was determined by chiral phase HPLC (220 nm).
[c] $[Pd(C_3H_5)Cl]_2$ was added as solid material.
[d] not determined.

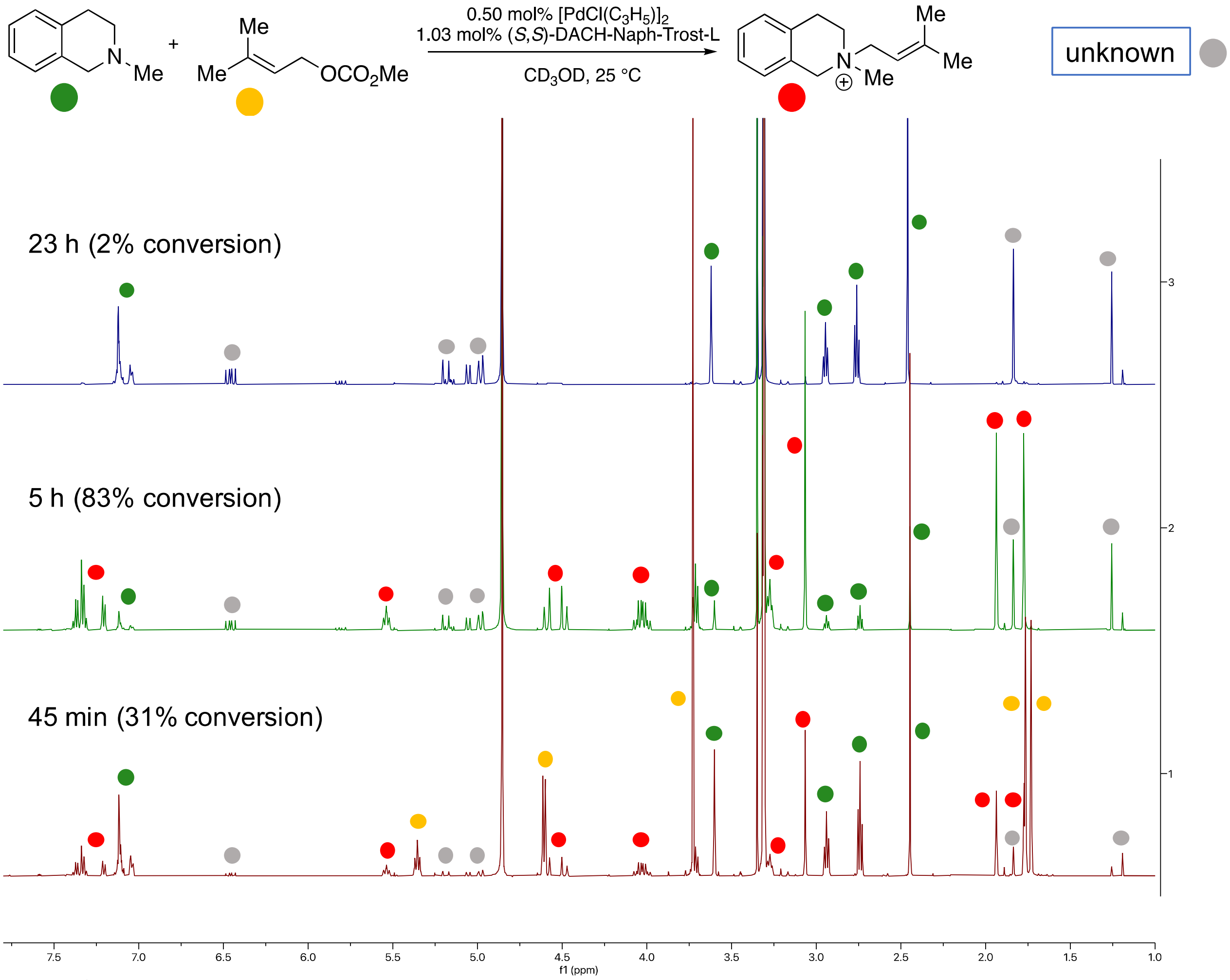

Fig. S6. ^{1}H NMR spectra (spectrometer frequency 500 MHz, solvent CD_3OD) for reaction 4 from the table above (0.50 mol% $[Pd(allyl)Cl]_2$, 1.03 mol% (*S*,*S*)-DACH-naphthyl Trost ligand).

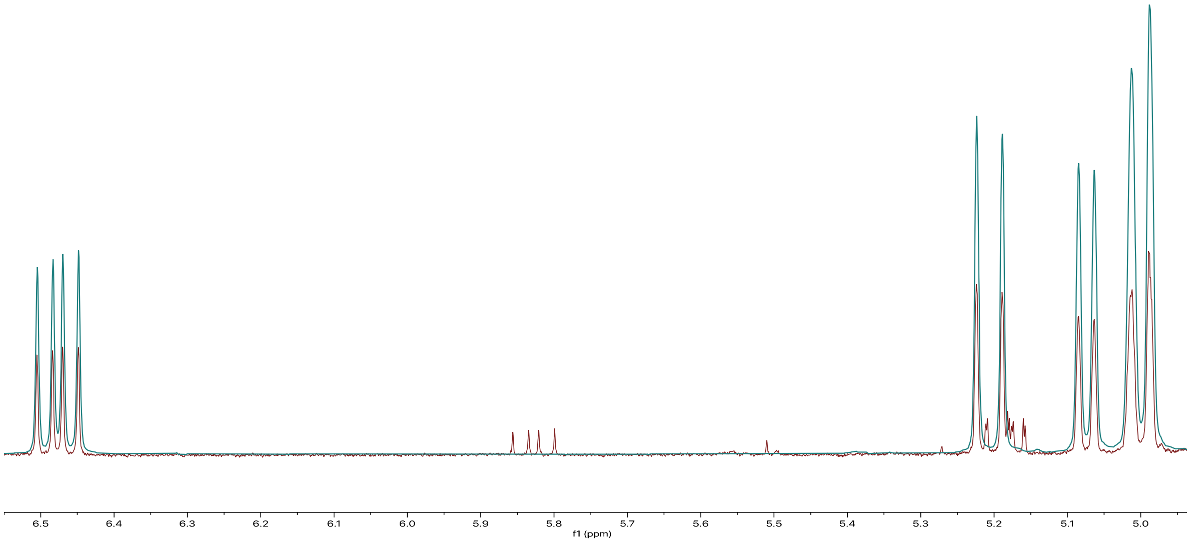

Fig. S7. Comparison of ^{1}H NMR spectra (spectrometer frequency 500 MHz, solvent CD_3OD) of isoprene (green) and reaction mixture (red). Ligand = (*S*,*S*)-DACH-Naphth-Trost-L, reaction time 23 h.

Effect of the leaving group on side product formation

Prenyl electrophiles with different leaving groups were investigated in respect to isoprene formation. Catalytic reactions were set up as described in the *Catalysis* section on a 0.6 mL scale with deuterated solvents; $[PdCl(C_3H_5)]_2$ (15 mM in CD_2Cl_2, 10 µL), (*S*,*S*)-DACH-phenyl Trost-ligand (31 mM in CD_2Cl_2,10 µL); Both nucleophile (4.5 µL, 30 µmol) and electrophiles (1.5 eq.) were used as neat materials.
Reaction mixtures were prepared in the glovebox in HPLC-vials and placed in tightly closing NMR tubes with PTFE-lined screw caps. ^1H NMR spectra were recorded at the scheduled time points ('0', 1, 2, 4, 8, 16, 24, 32 h).

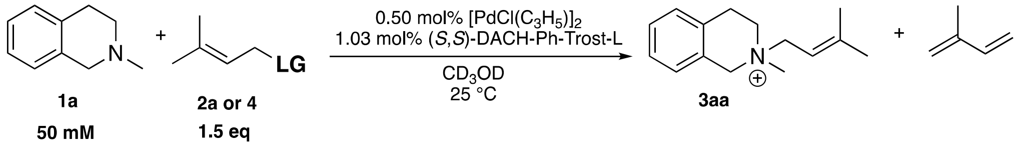

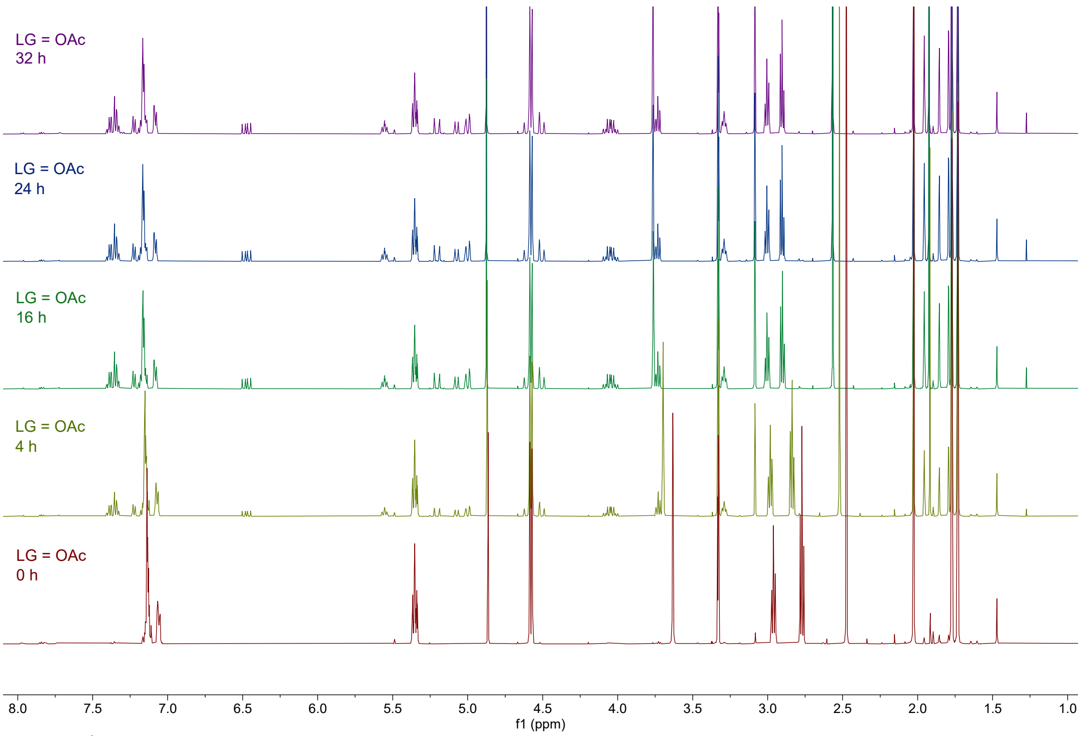

Fig. S8. ^1H NMR spectra (500 MHz) at different time points ((0, 4, 16, 24 and 32 hours) for the reaction of **1**a (50 mM) with **2** (1.5 eq., LG = OAc) in the presence of $[Pd(C_3H_5)Cl]_2$ (0.50 mol%) and (*S*,*S*)-DACH-Ph-Trost-ligand (1.03 mol%) in CD_3OD.

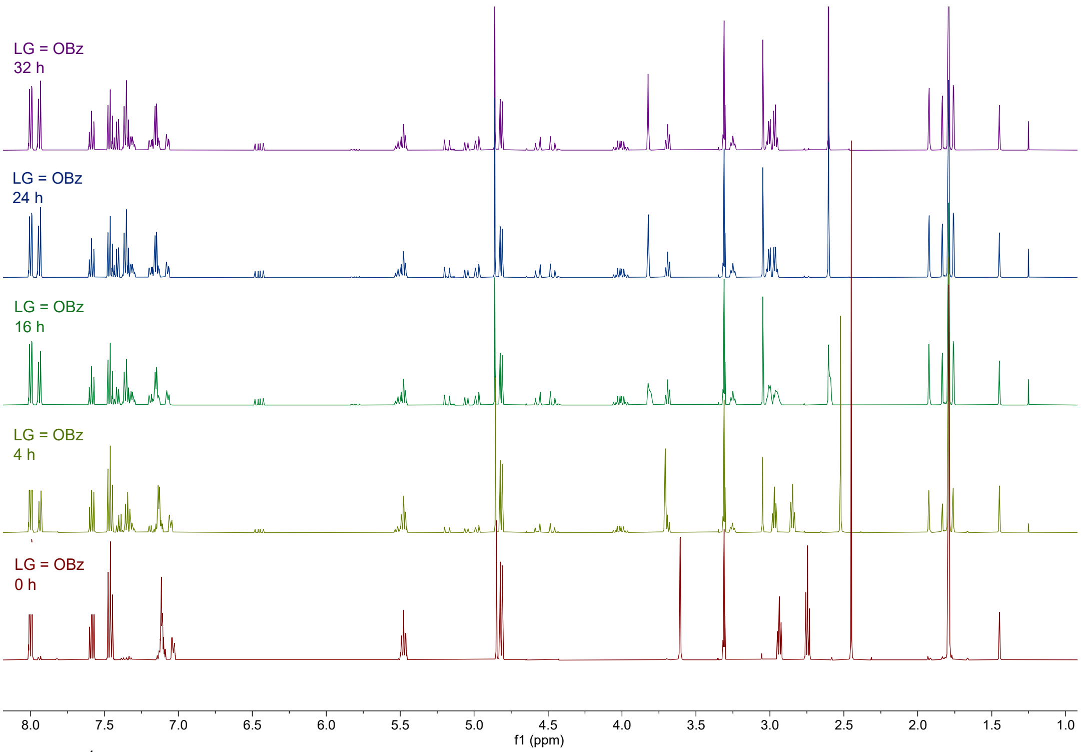

Fig. S9. ^{1}H NMR spectra (500 MHz) at different time points ((0, 4, 16, 24 and 32 hours) for the reaction of **1a** (50 mM) with **2** (1.5 eq., LG = OBz) in the presence of $[Pd(C_3H_5)Cl]_2$ (0.50 mol%) and (*S*,*S*)-DACH-Ph-Trost-ligand (1.03 mol%) in CD_3OD.

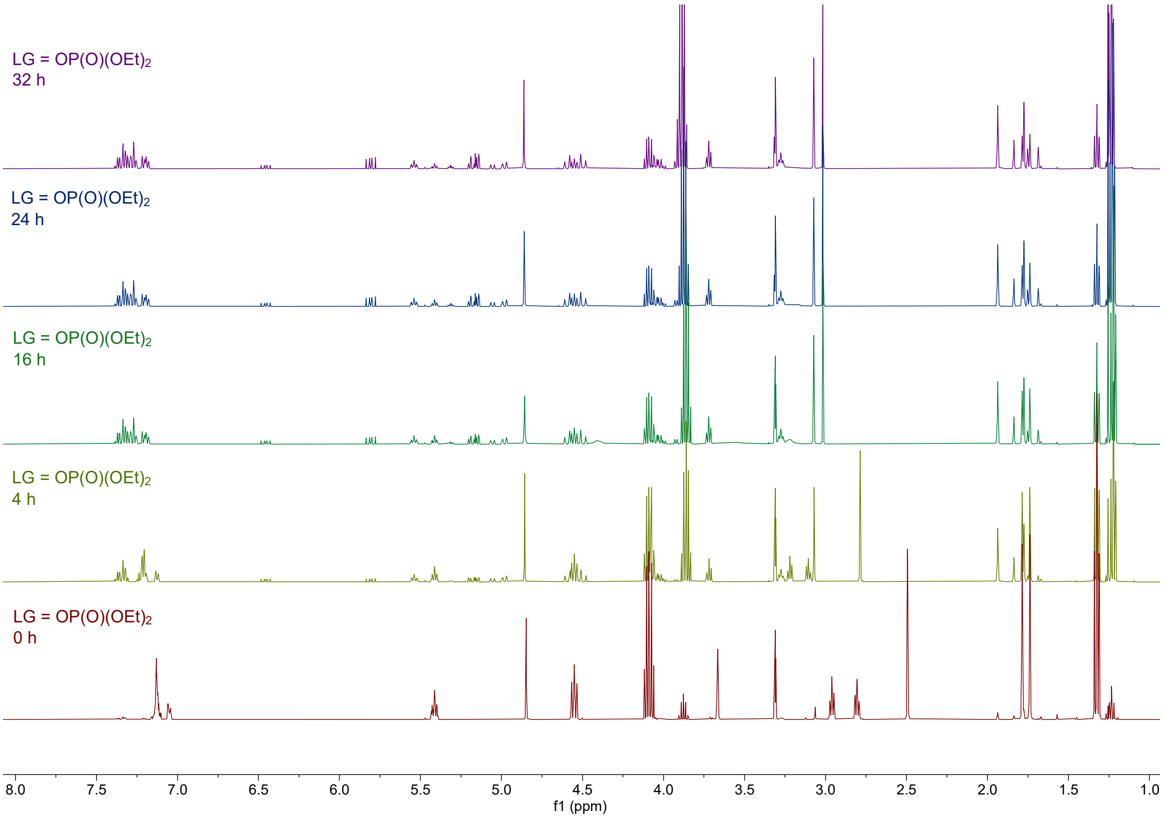

Fig. S10. ^{1}H NMR spectra (500 MHz) at different time points ((0, 4, 16, 24 and 32 hours) for the reaction of **1a** (50 mM) with **2** (1.5 eq., LG = $OP(O)(OEt)_2$) in the presence of $[Pd(C_3H_5)Cl]_2$ (0.50 mol%) and (*S*,*S*)-DACH-Ph-Trost-ligand (1.03 mol%) in CD_3OD.

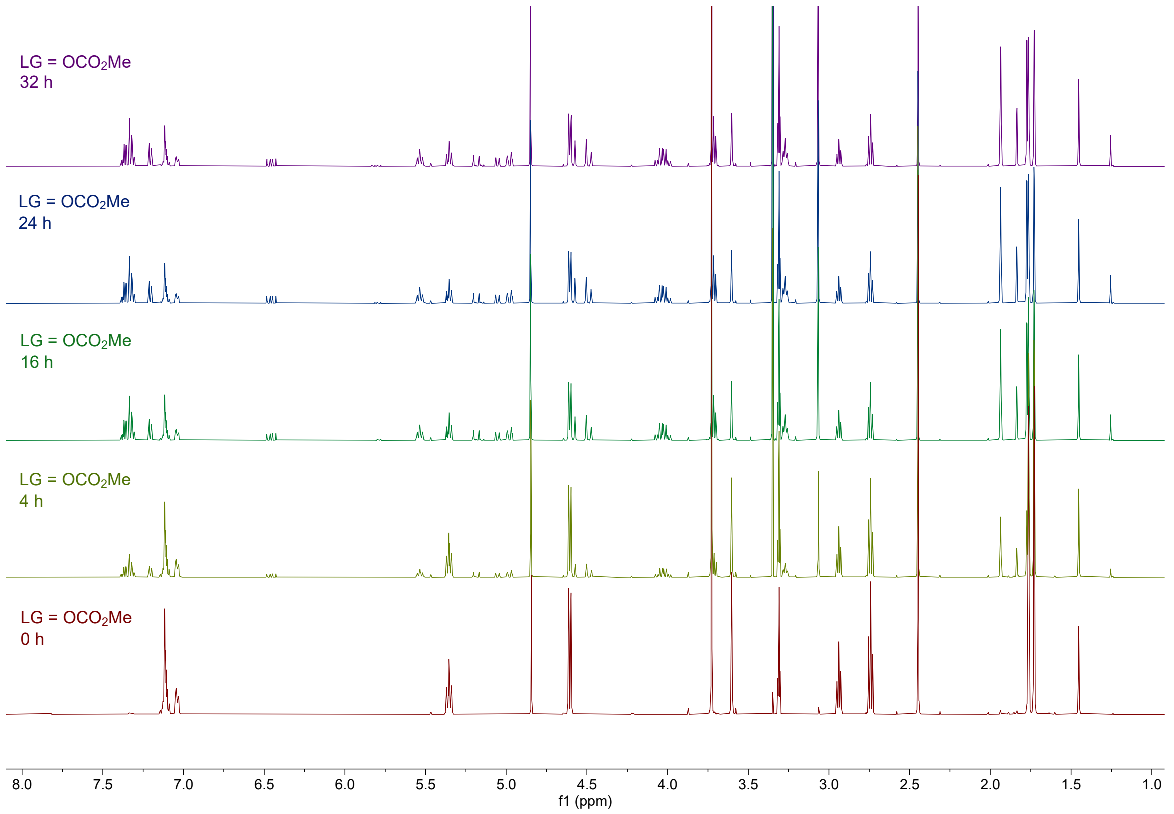

Fig. S.11. ^{1}H NMR spectra (500 MHz) at different time points ((0, 4, 16, 24 and 32 hours) for the reaction of **1**a (50 mM) with **2** (1.5 eq., LG = OCO_2Me) in the presence of $[Pd(C_3H_5)Cl]_2$ (0.50 mol%) and (*S*,*S*)-DACH-Ph-Trost-ligand (1.03 mol%) in CD_3OD.

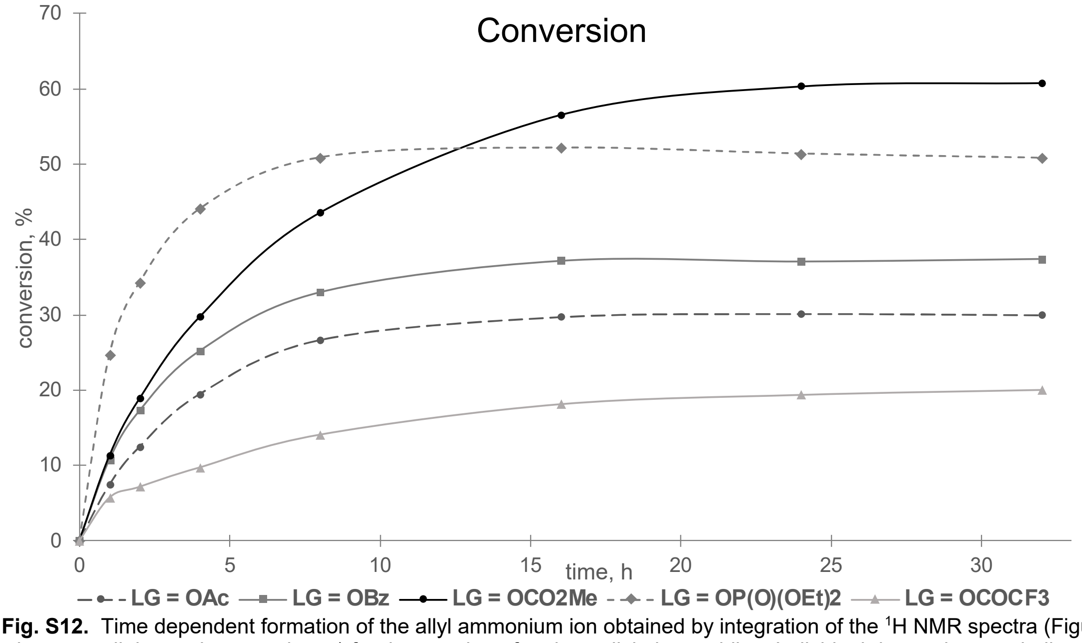

Fig. S12. Time dependent formation of the allyl ammonium ion obtained by integration of the ^{1}H NMR spectra (Figures S8-S11, where not all time points are shown) for the reaction of various allyl electrophiles. Individual data-points are indicated. The lines have been drawn to guide the eye.

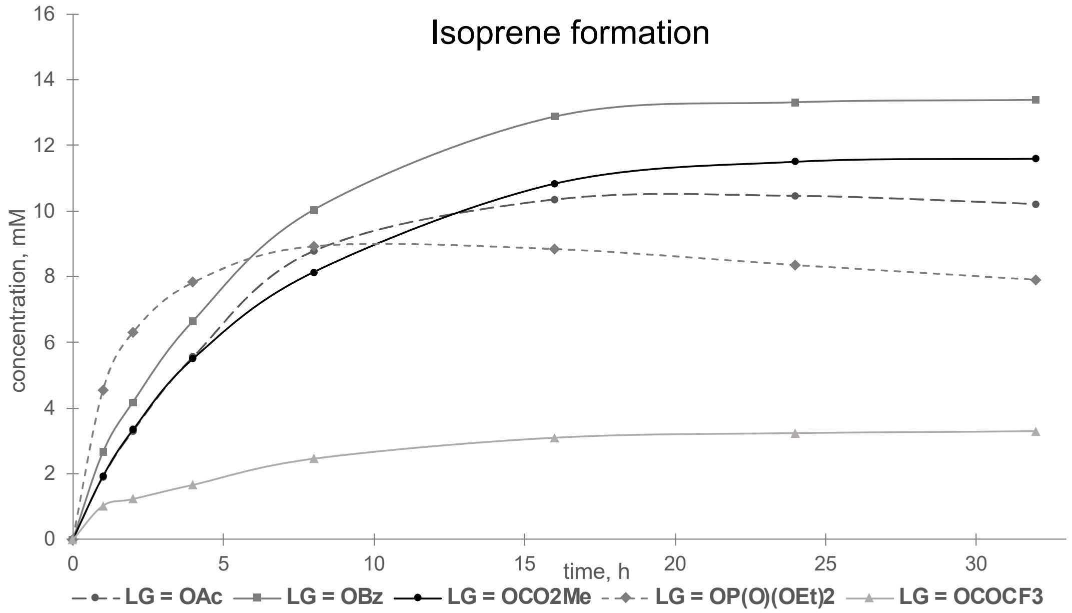

Fig. S13. Time course for isoprene formation for various allyl electrophiles obtained by integration of the ^{1}H NMR spectra (Figures S8-S11 where not all time points are shown). Individual data-points are indicated. The lines have been drawn to guide the eye.

Effect of water addition on conversion and isoprene formation

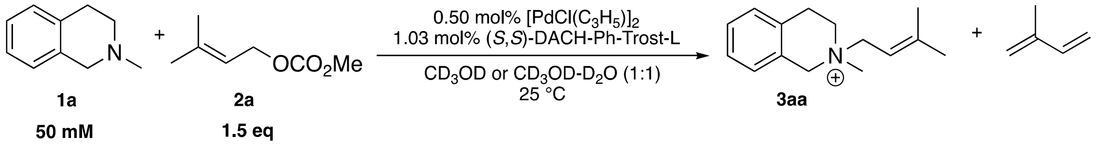

Catalytic reactions were set up as described in the *Catalysis* section on a 0.6 mL scale with deuterated solvents; $[PdCl(C_3H_5)]_2$ (15.0 mM in CD_2Cl_2), (*S*,*S*)-DACH-phenyl Trost-ligand (30.9 mM in CD_2Cl_2); Both nucleophile (4.5 µL, 0.030 mmol) and electrophile (6.4 µL, 0.043 mmol, 1.45 eq) were used as neat material.
Reaction mixtures were prepared in the glovebox in HPLC-vials and placed in tightly closing NMR tubes with PTFE-lined screw caps. ^{1}H NMR spectra were recorded at the scheduled time points ('0', 1, 2, 4, 8, 16, 24, 32 h).

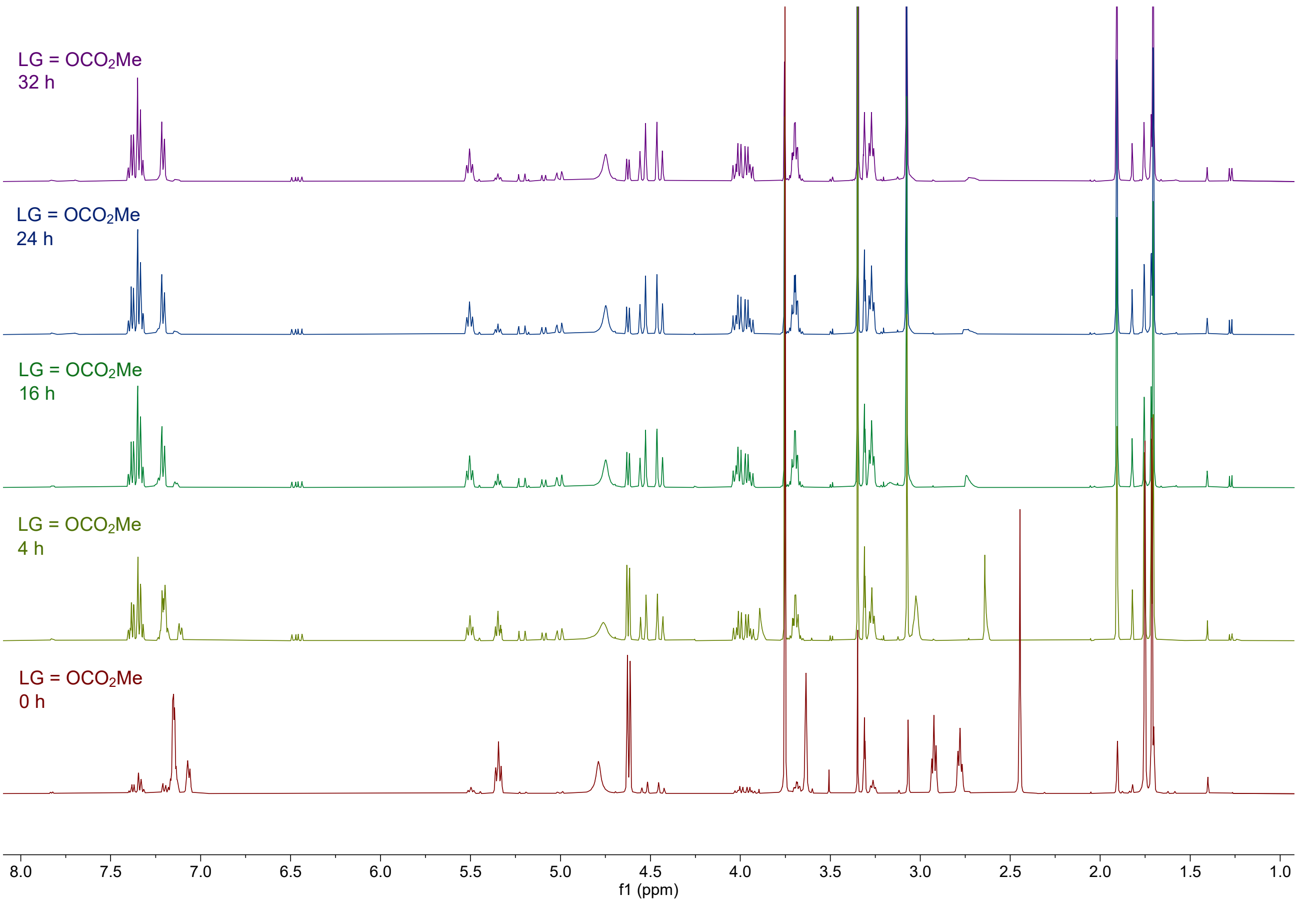

Fig. S14 ^{1}H NMR spectra (500 MHz) at different time points ((0, 4, 16, 24 and 32 hours) for the reaction of **1a** (50 mM) with **2a** (1.5 eq., LG = OCO_2Me) in the presence of $[Pd(C_3H_5)Cl]_2$ (0.50 mol%) and (*S*,*S*)-DACH-Ph-Trost-ligand (1.03 mol%) in CD_3OD-D_2O 1:1.

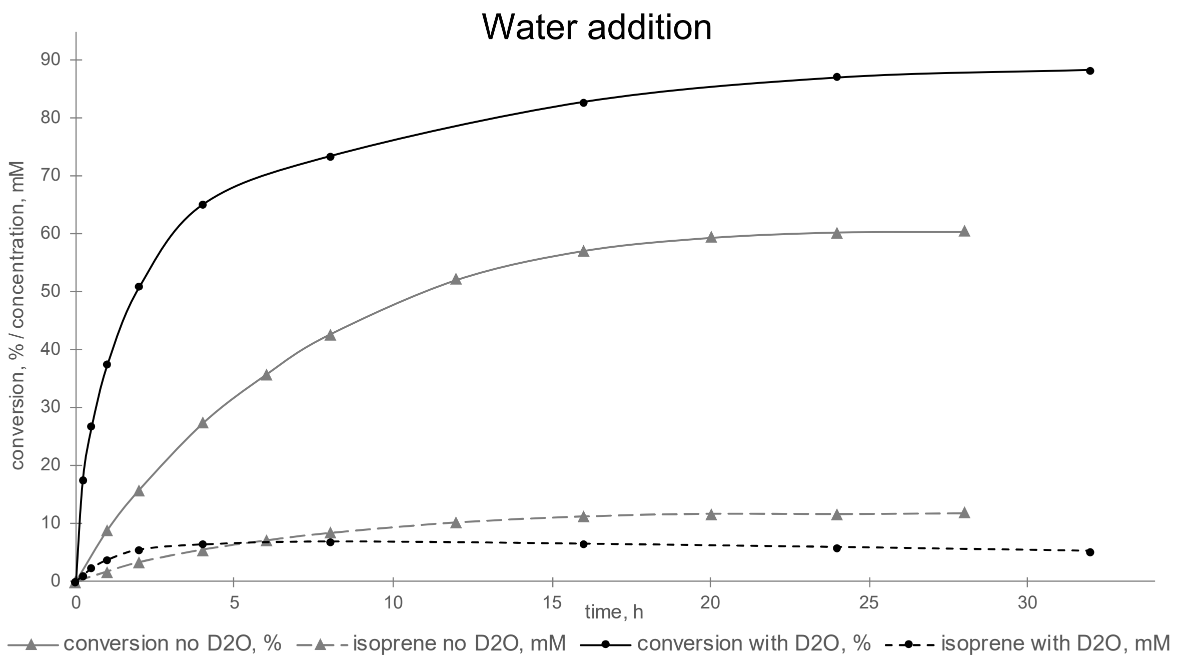

Fig. S15. The effect of water addition on isoprene formation and conversion of amine **1a** to ammonium ion **3aa** shown as time dependent plots for the reaction of allyl methyl carbonate **2a** (1.5 eq., LG = OCO_2Me) and amine **1a** obtained by integration of the ^{1}H NMR (Figure S14 where not all time points are shown). Individual data-points are indicated. The lines have been drawn to guide the eye.

Solvolysis of the electrophile

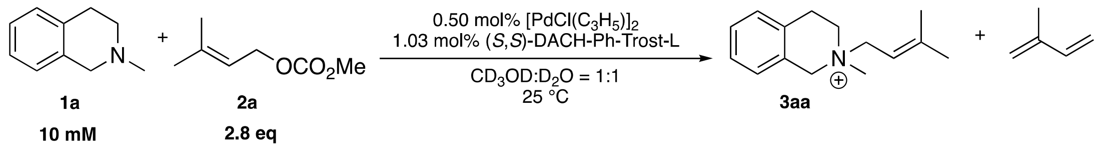

The monitoring of the reaction by ^{1}H NMR spectroscopy revealed that three main side products are formed: isoprene, the branched allyl alcohol and the branched methyl allyl ether.

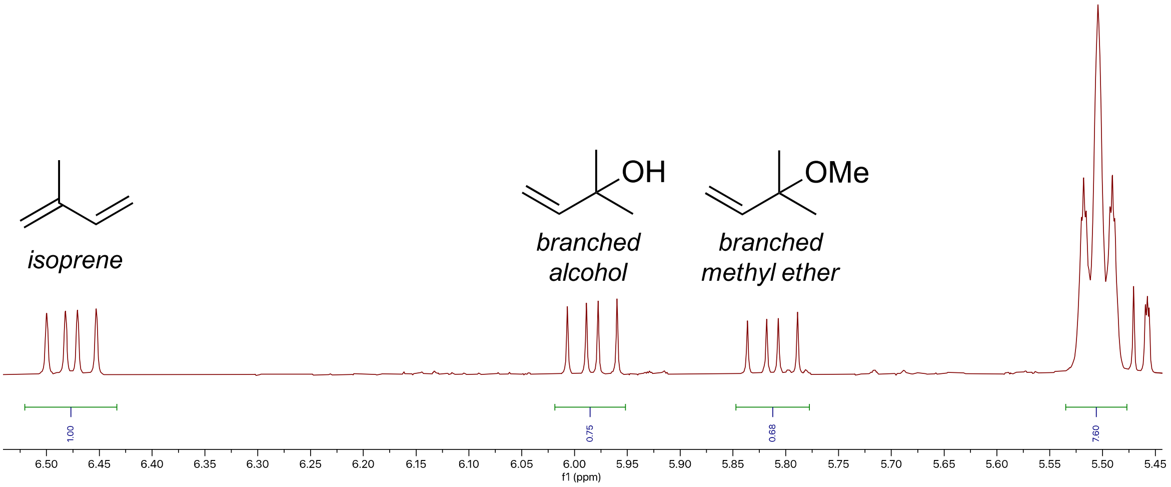

Fig. S16. Section of the ^{1}H NMR spectrum (600 MHz) of the reaction in CD_3OD:D_2O = 1:1 showing diagnostic signals for side products isoprene, 2-methylbut-3-en-2-ol and 3-methoxy-3-methylbut-1-ene after 48 h reaction time.

In order to investigate the formation of side products further, the following experiments were set up:

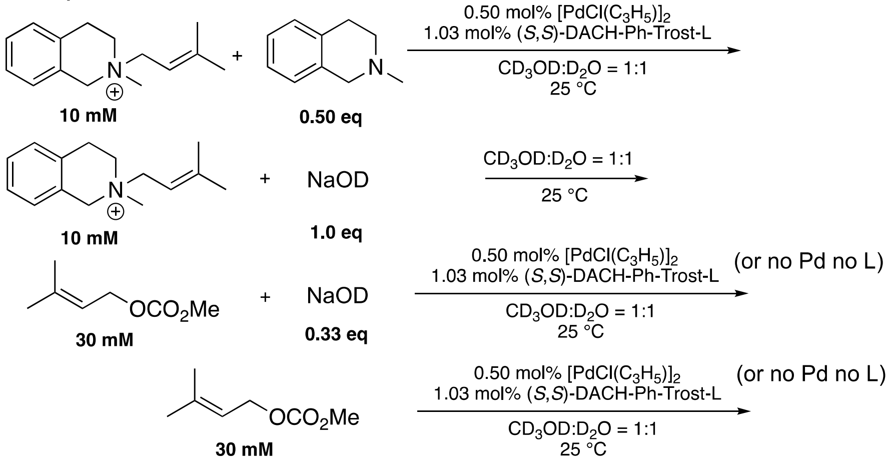

Catalytic reactions were set up as described in the *Catalysis* section on a 1.0 mL scale with deuterated solvents. Both nucleophile and electrophile were used as freshly prepared stock solutions (100 mM N-Me THIQ and 500 mM prenyl methyl carbonate in CD_3OD, stock solutions prepared in the glove box), NaOD was used as a commercial 40% solution in D_2O. Reaction mixtures were prepared in the glovebox in HPLC-vials and placed in tightly closing NMR tubes with PTFE-lined screw caps. ^{1}H NMR spectra were recorded at the scheduled time points ('0', 1, 2, 4, 8, 16, 24, 32 h).

Table S12. Study of the side products formation

Exp	Electrophile	Pd, L	Nucleophile	Isoprene[a]	Branched alcohol[a]	Branched ether[a]	Prenyl alcohol
a)	**allyl ammonium salt** ([**3aa**]Br)	yes	N-Me THIQ, 0.5 eq	5.5%	no	0.1%	–
b)	**allyl ammonium salt** ([**3aa**]Br)	no	NaOD, 1eq	no	no	no	–
c)	**allyl methyl carbonate**[b] (**2a**)	yes	NaOD, 0.33 eq	4.1%	0.8%	6.3%	36% (0h) 89% (24h)
d)	**allyl methyl carbonate**[b] (**2a**)	no	NaOD, 0.33 eq	no	0.3%	0.2%	38% (0h) 68% (24h)
e)	**allyl methyl carbonate**[c] (**2a**)	yes	no	traces	2.8%	1.5%	0% (0h) 1.0% (24h)
f)	**allyl methyl carbonate**[c] (**2a**)	no	no	traces	2.9%	1.7%	0% (0h) 1.1% (24h)

[a] Concentration of the side products was determined as the mole fraction in the reaction mixture by ^{1}H NMR (600 MHz) after 24 h of the reaction running;
[b] Already at time point '0h', 36-38% of the prenyl methyl carbonate was converted to prenyl alcohol by solvolysis;
[c] In e) and f) 0.4% of another side product was formed, also with a terminal double bond. It corresponds to the signals reported[26] for

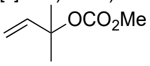

.

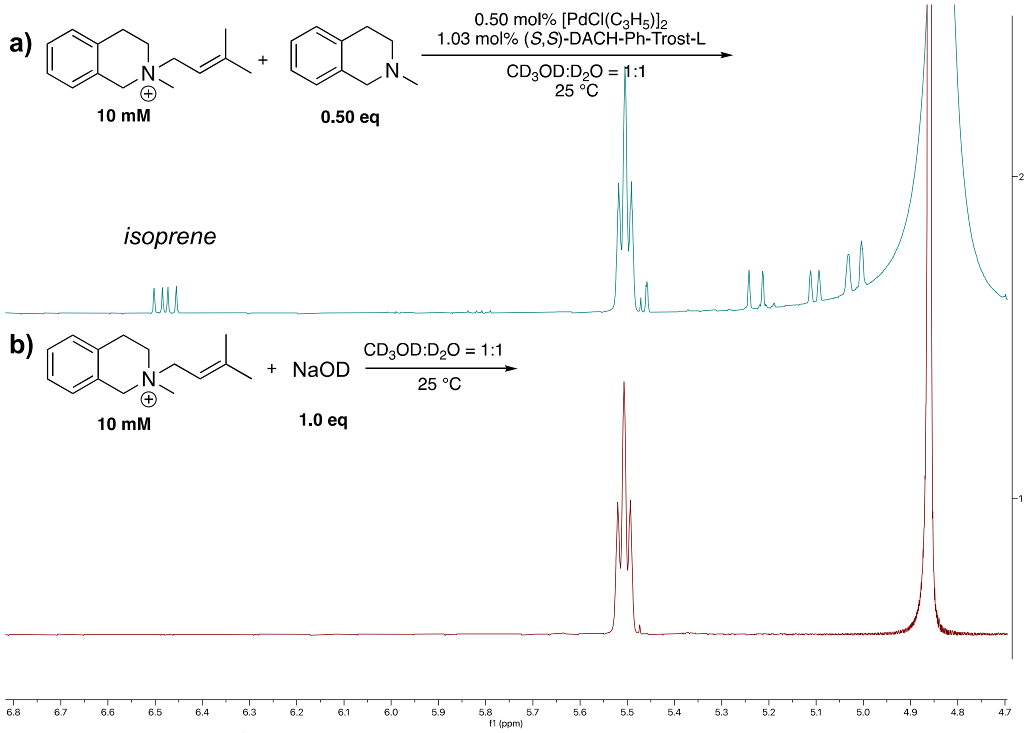

Fig. S17. Zoomed area of the ^{1}H NMR spectra (600 MHz) for reactions **a)** and **b)** after 24 hours. No side products and also no Hofmann elimination product were identified when no catalyst was present. Isoprene can be formed from the allyl ammonium salt with $[PdCl(C_3H_5)]_2$ and ligand in the presence of a base.

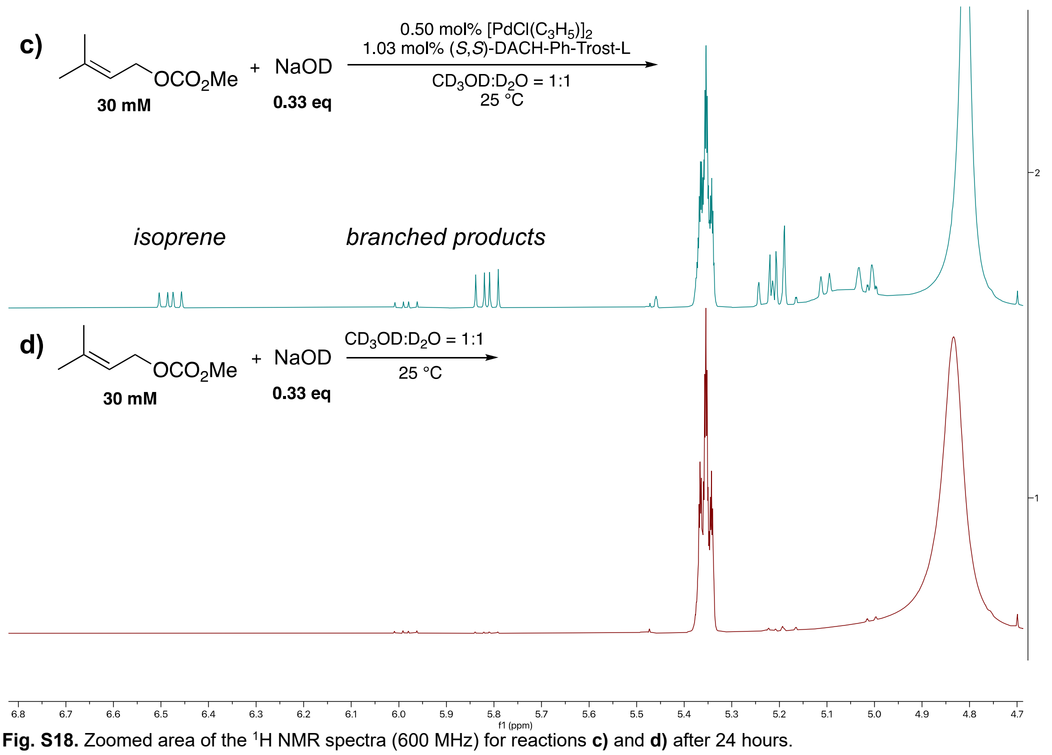

Fig. S18. Zoomed area of the ^{1}H NMR spectra (600 MHz) for reactions **c)** and **d)** after 24 hours.

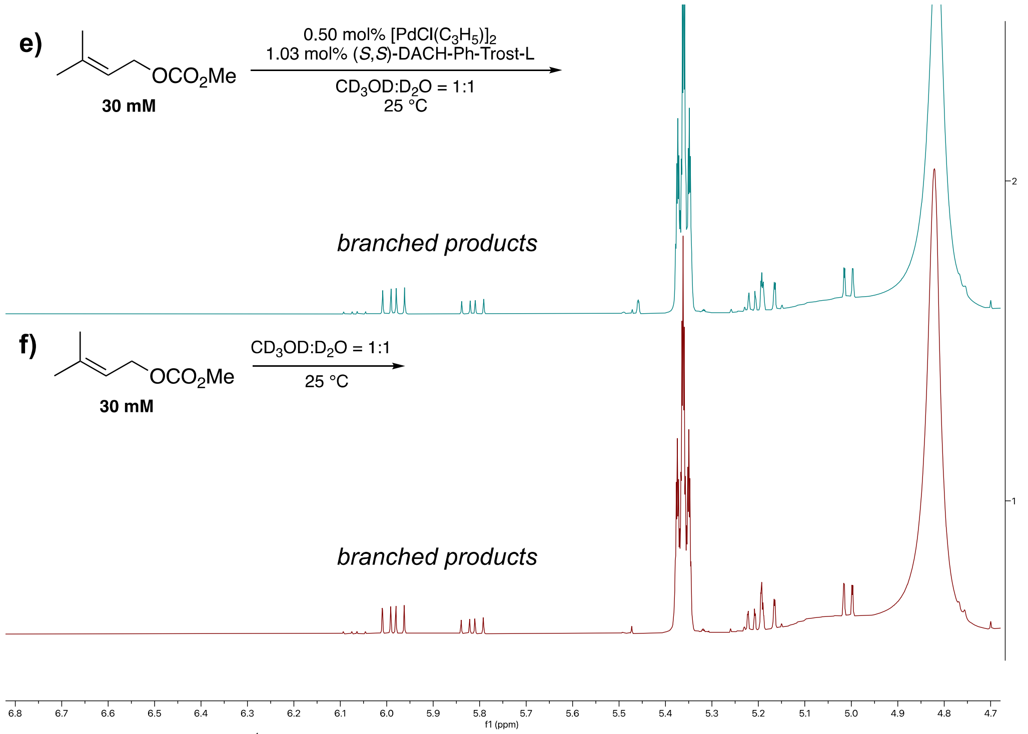

Fig. S19. Zoomed area of the ^{1}H NMR spectra (600 MHz) for reactions **e)** and **f)** after 24 hours. In the absence of a base, isoprene is not formed. Branched products were formed almost to the same extent no matter if Pd was in the system or not.

Spontaneous formation of the chloromethylated ammonium ion in CH_2Cl_2

A 1M solution of N-Me THIQ (**1a**) in CH_2Cl_2 was stored at room temperature in the glovebox for 2 weeks under a nitrogen atmosphere, whereupon crystals were found to have formed in the vial. The crystals were separated from the liquid, washed with CH_2Cl_2 and Et_2O and dried under air. NMR and LCMS indicated that the chloromethylation of the tertiary amine had occurred.

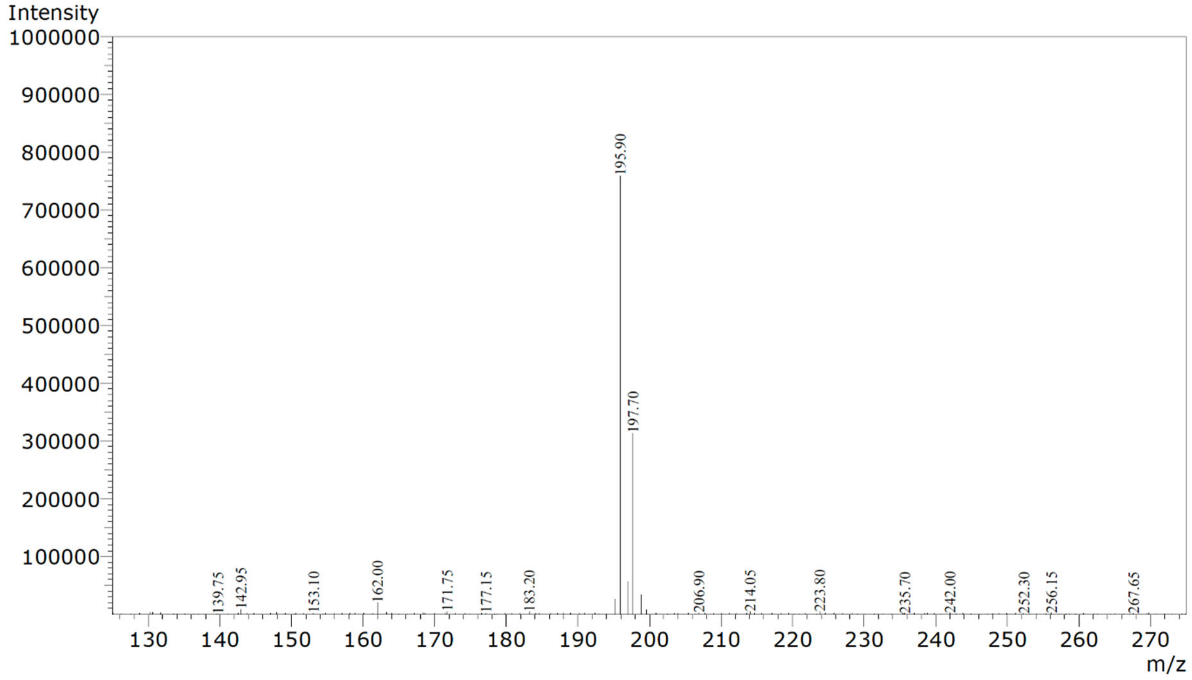

Fig. S20. MS of the chloromethylated product **S5**.

2-(chloromethyl)-2-methyl-1,2,3,4-tetrahydroisoquinolin-2-ium chloride (S5)

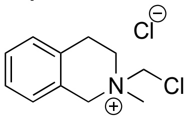

^{1}H NMR (500 MHz, $CDCl_3$) δ 7.29 (td, *J* = 7.5, 1.5 Hz, 1H), 7.26 – 7.22 (m, 1H), 7.21 (d, *J* = 7.6 Hz, 1H), 7.13 (d, *J* = 7.5 Hz, 1H), 6.11 (AB spin system, δ_A = 6.20, δ_B = 6.01, J_{AB} = 9.5 Hz, 2H), 5.12 (AB spin system, δ_A = 5.23, δ_B = 5.00, J_{AB} = 15.1 Hz, 2H), 4.41 – 4.32 (m, 1H), 4.25 – 4.16 (m, 1H), 3.51 (s, 3H), 3.29 – 3.14 (m, 2H); ^{13}C NMR (126 MHz, $CDCl_3$) δ 129.2, 129.0, 128.7, 128.0, 127.8, 125.7, 68.6, 60.5, 56.44, 46.0, 23.8; HRMS (ESI) m/z: 196.0888 found (calcd. for $C_{11}H_{15}ClN^+$, [M^+] 196.0888).

Equilibrium position of tertiary amine allylation in different solvents

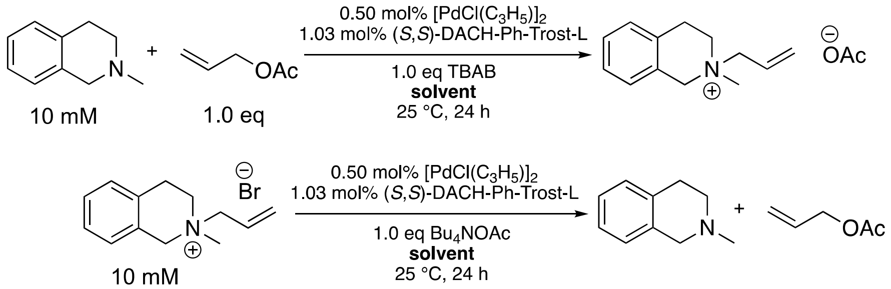

Four catalytic reactions (allylation and deallylation in MeOH/water or CH_2Cl_2) were set on a 1.4 mL scale in HPLC vials in the glovebox as described before. Nucleophiles and electrophiles were used as neat material. HPLC-yield and *er* were determined after 21 h.

The same experiment was repeated on a 1.0 mL scale in tightly closing NMR tubes with PTFE-lined screw caps with deuterated solvents.

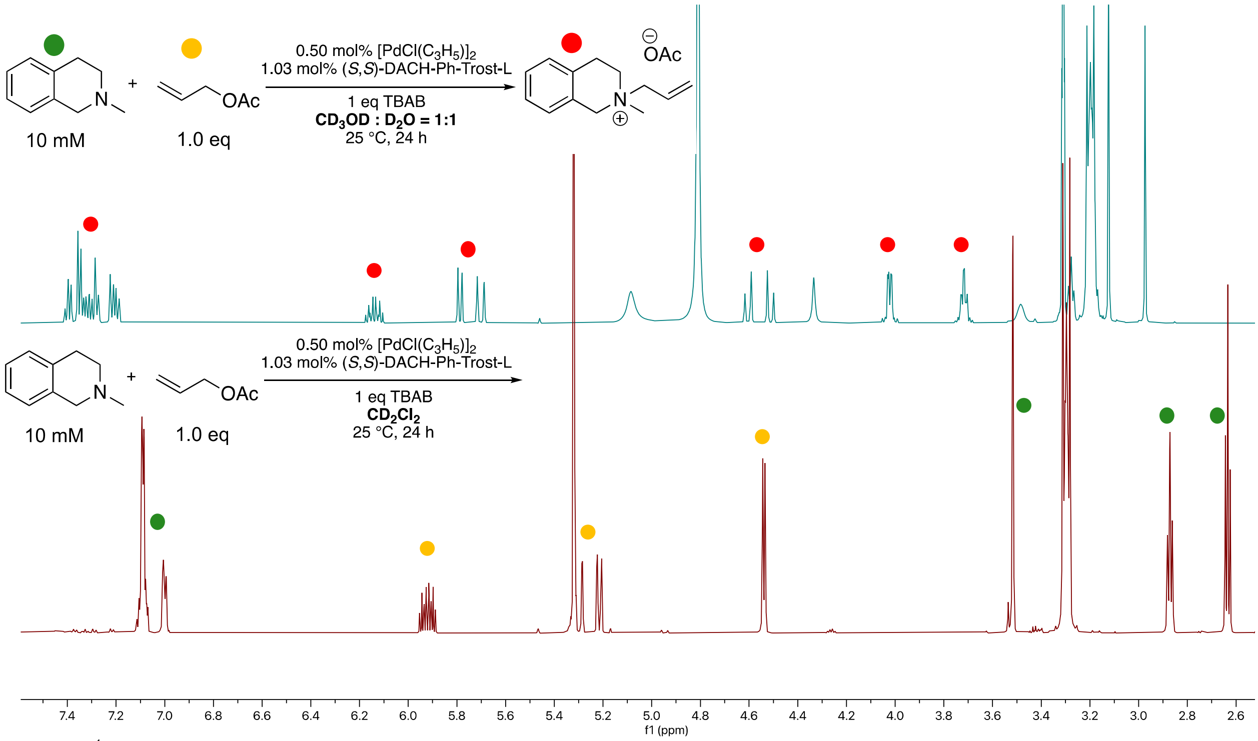

Fig. S21. ^{1}H NMR spectra (600 MHz) of the shown reaction mixtures after 24 hours. Reactions were set up on a 1.0 mL scale as described in the *Catalysis* section with deuterated solvents; Allylation of the *2-methyl-1,2,3,4-tetrahydroisoquinoline* is strongly shifted towards product in protic solvents. TBAB = $[Bu_4N]Br$.

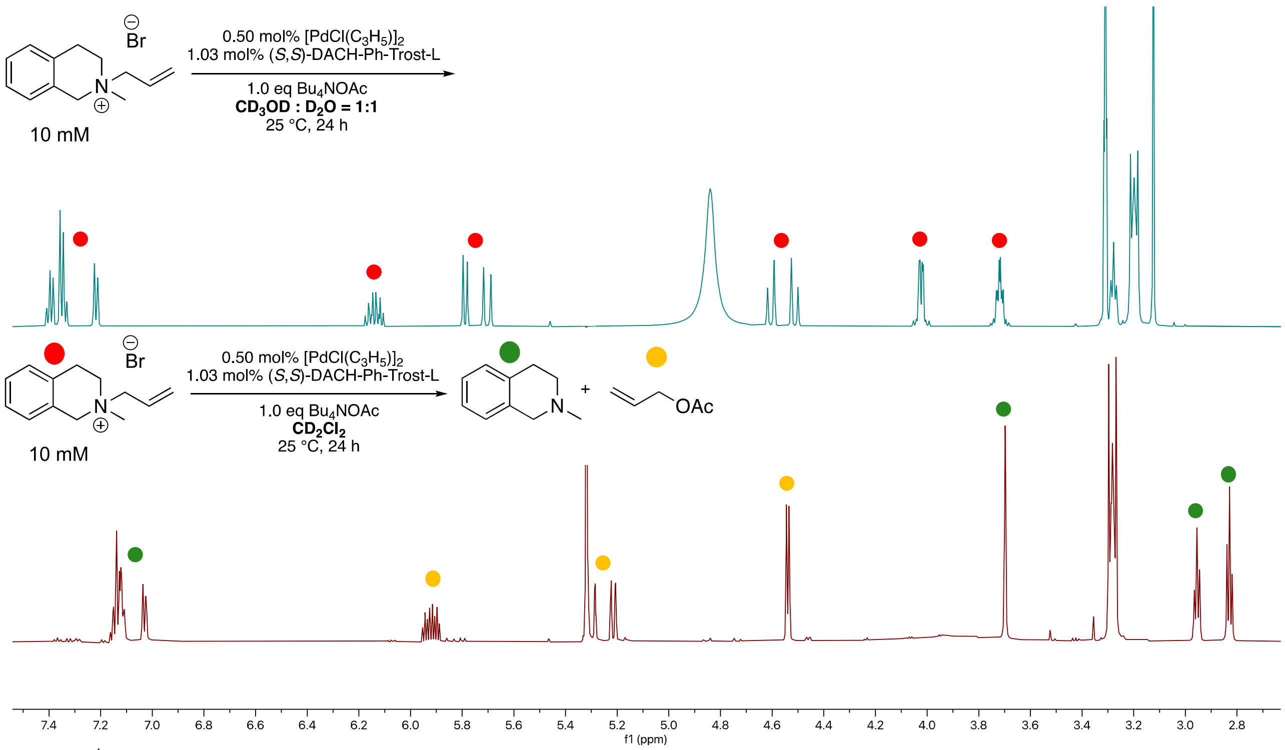

Fig. S22. ^{1}H NMR spectra (600 MHz) of the shown reaction mixtures after 24 hours. Reactions were set up on a 1.0 mL scale as described in the *Catalysis* section with deuterated solvents; Deallylation of the *2-methyl-1,2,3,4-tetrahydroisoquinolinium bromide* is strongly shifted towards the amine in CD_2Cl_2.

X-ray crystallographic data and determination of absolute configuration

To determine the absolute configuration of the major enantiomer of **3aa** formed in the catalysis reaction when (*S*,*S*)-DACH-Phenyl-Trost ligand was employed, diastereomerically enriched salt **S3** (dr 97:3) was recrystallized in an NMR tube by slow diffusion of ethyl acetate into a chloroform-methanol solution of the salt to obtain crystals suitable for X-ray crystal structure determination. The configuration of the ammonium ion **3aa** was determined in three independent crystals and could be assigned unambiguously as being (*R*)-configured in relation to the counteranion (singly protonated (*R*,*R*)-dibenzoyltartaric acid). Chiral phase HPLC-analysis of the the diastereomerically enriched salt **S3** and comparison with the ammonium ion **3aa** formed under employment of the (*S*,*S*)-Trost ligand established that (*R*)-enantiomer is the major enantiomer formed in the catalytic reaction.

Experimental. Single colourless block-shaped crystals of **SNZ358_150K** were used as supplied. A suitable crystal with dimensions 0.22 × 0.18 × 0.11 mm^3 was selected and mounted on a mylar loop in perfluoroether oil on a Basel-Cu-Stoe diffractometer. The crystal was kept at a steady T = 150 K during data collection. The structure was solved with the **ShelXT**[27] solution program using dual methods and by using **Olex2** 1.5[28] as the graphical interface. The model was refined with **ShelXL**[29] 2018/3 using full matrix least squares minimisation on $\boldsymbol{F^2}$. All non-hydrogen atoms were refined anisotropically. Hydrogen atom positions were calculated geometrically and refined using the riding model. Two more crystals were measured and were in accordance with the reported observations.

Crystal Data. $C_{33}H_{35}NO_8$, M_r = 573.62, orthorhombic, $P2_12_12_1$ (No. 19), a = 7.66040(10) Å, b = 13.7870(2) Å, c = 28.2460(5) Å, $a = b = g$ = 90˚, V = 2983.17(8) Å^3, T = 150 K, Z = 4, Z' = 1, m(Cu K_a) = 0.750, 70571 reflections measured, 5916 unique (R_{int} = 0.0626) which were used in all calculations. The final wR_2 was 0.2173 (all data) and R_1 was 0.0821 (I≥2 s(I)).

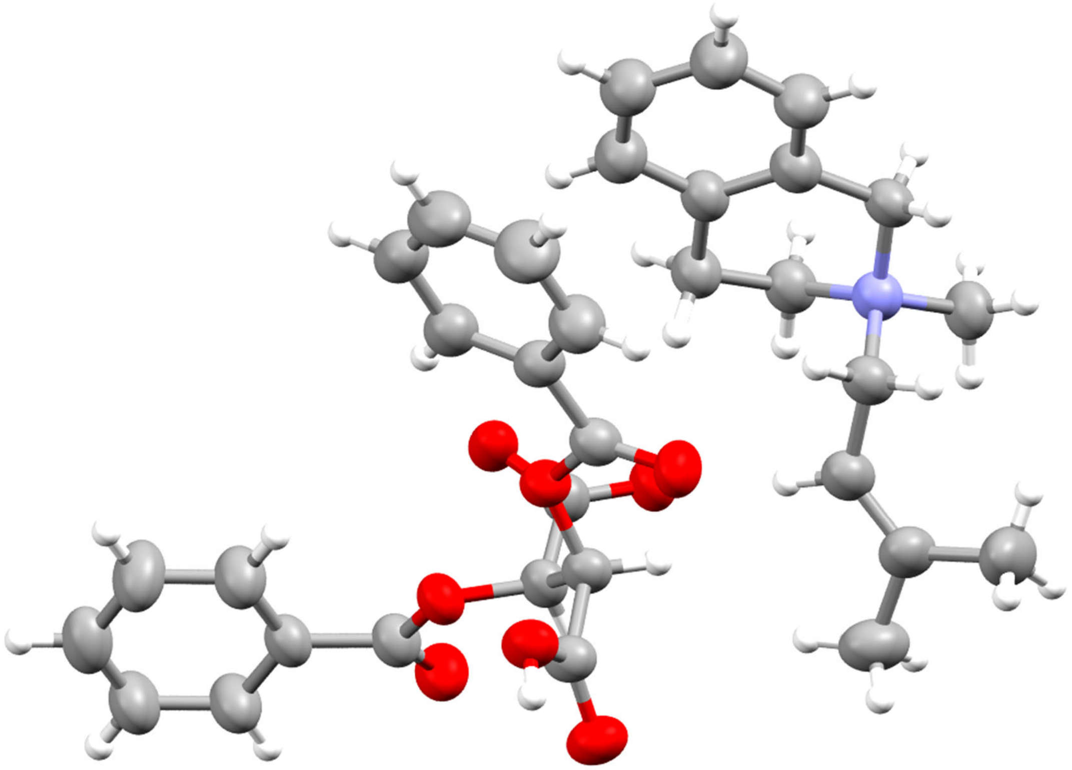

Fig. S23. Crystal structure (CCDC code 2218756) with 50% probability ellipsoids for carbon (grey), oxygen (red) and nitrogen (blue). Hydrogen atoms are displayed as fixed-size spheres with a radius of 0.2 Å. Bonds are displayed as sticks with a radius of 0.1 Å. The picture was generated with Mercury 2021.3.0 (Build 333817).

Data were measured using rotation method, *w* scans with Cu K_a radiation. The diffraction pattern was indexed and the total number of runs and images was based on the strategy calculation from the program X-Area Pilatus3_SV 1.31.175.0 (STOE, 2021). The maximum resolution that was achieved was Q = 73.140° (0.81 Å).

The unit cell was refined using X-Area Integrate 2.5.1.0 (STOE, 2021) X-Area LANA 2.6.2.0 (STOE, 2021) on 101145 reflections, 143% of the observed reflections.

Data reduction, scaling and absorption corrections were performed using X-Area Integrate 2.5.1.0 (STOE, 2021) and X-Area LANA 2.6.2.0 (STOE, 2021). The final completeness is 99.90 % out to 73.140° in Q. A multi-scan absorption correction was performed using STOE X-Red32, absorption correction by Gaussian integration, analogous to P. Coppens in: F. R. Ahmed (Editor), "Crystallographic Computing".[30] Afterwards scaling of reflection intensities was performed within STOE LANA.[31] Finally, a spherical absorption correction was done within STOE LANA. The absorption coefficient m of this material is 0.750 mm^{-1} at this wavelength (l = 1.54186Å) and the minimum and maximum transmissions are 0.518 and 0.737.

HPLC methods

Determination of HPLC-yields

Column Agilent Zorbax XDB-C18 (150 mm × 4.6 mm, 5 µm); solvent A = 97% H_2O, 3% CH_3CN; 0.1 % CF_3CO_2H, solvent B = CH_3CN, 0.1 % CF_3CO_2H; flow = 1 mL/min, T = 40°C, wavelength 210 nm, injection volume 2 µL.

Table S13. Methods used for reversed phase HPLC

ID	Description	Used for analysis of reactions with
**Method A**	isocratic 10% B until 15 min; gradient from 10% to 90% B over next 5 min; isocratic 90% B for 2 min; gradient from 90% to 10% B over next 3 min; isocratic 10% B for 5 min (total run time 30 min)	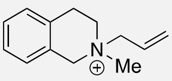 , 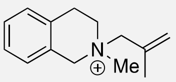
**Method B**	isocratic 18% B until 12 min; gradient from 18% to 90% B over next 3 min; isocratic 90% B for 2 min; gradient from 90% to 18% B over next 3 min; isocratic 18% B for 5 min (total run time 25 min)	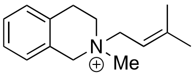 , 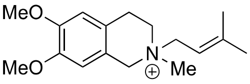 , 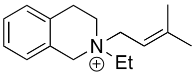
**Method C**	isocratic 25% B until 12 min; gradient from 25% to 90% B over next 3 min; isocratic 90% B for 2 min; gradient from 90% to 25% B over next 3 min; isocratic 25% B for 5 min (total run time 25 min)	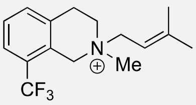
**Method D**	isocratic 18% B until 6 min; gradient from 18% to 20% B over next 3 min; isocratic 20% B for 7 min; gradient from 20% to 90% B over next 2 min; isocratic 90% B for 7 min; gradient from 90% to 18% B over next 2 min; isocratic 18% B for 5 min (total run time 32 min)	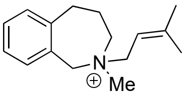
**Method E**	isocratic 18% B until 6 min; gradient from 18% to 25% B over next 3 min; isocratic 25% B for 7 min; gradient from 25% to 90% B over next 2 min; isocratic 90% B for 7 min; gradient from 90% to 18% B over next 2 min; isocratic 18% B for 5 min (total run time 32 min)	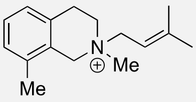 , 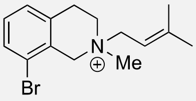
**Method F**	isocratic 18% B until 6 min; gradient from 18% to 30% B over next 3 min; isocratic 25% B for 7 min; gradient from 30% to 90% B over next 2 min; isocratic 90% B for 7 min; gradient from 90% to 18% B over next 2 min; isocratic 18% B for 5 min (total run time 32 min)	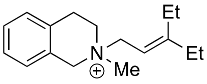 , 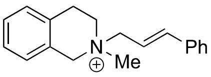 , 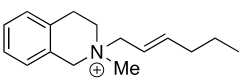 , 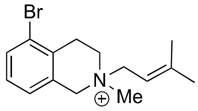
**Method G**	isocratic 18% B until 6 min; gradient from 18% to 50% B over next 3 min; isocratic 50% B for 6 min; gradient from 50% to 90% B over next 2 min; isocratic 90% B for 7 min; gradient from 90% to 18% B over next 2 min; isocratic 18% B for 5 min (total run time 31 min)	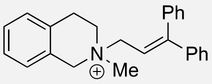

**Method H**	isocratic 20% B until 6 min; gradient from 20% to 30% B over next 3 min; isocratic 30% B for 7 min; gradient from 30% to 90% B over next 2 min; isocratic 90% B for 7 min; gradient from 90% to 20% B over next 2 min; isocratic 18% B for 5 min (total run time 32 min)	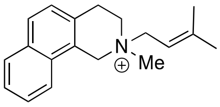
**Method I**	isocratic 30% B until 5 min; gradient from 30% to 40% B over next 3 min; isocratic 40% B for 8 min; gradient from 40% to 90% B over next 2 min; isocratic 90% B for 7 min; gradient from 90% to 30% B over next 2 min; isocratic 18% B for 5 min (total run time 32 min)	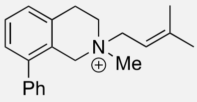
**Method J**	isocratic 32% B until 12 min; gradient from 32% to 90% B over next 3 min; isocratic 90% B for 2 min; gradient from 90% to 32% B over next 3 min; isocratic 32% B for 5 min (total run time 25 min)	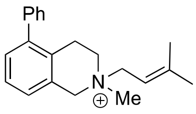
**Method N**	isocratic 40% B until 10 min; gradient from 40% to 90% B over next 3 min; isocratic 90% B for 2 min; gradient from 90% to 40% B over next 3 min; isocratic 40% B for 5 min (total run time 23 min)	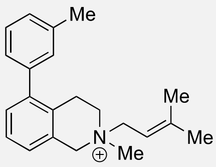, 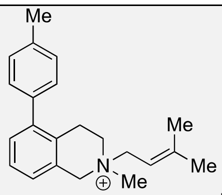, 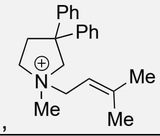
**Method O**	isocratic 15% B until 12 min; gradient from 15% to 90% B over next 3 min; isocratic 90% B for 2 min; gradient from 90% to 15% B over next 3 min; isocratic 15% B for 5 min (total run time 25 min)	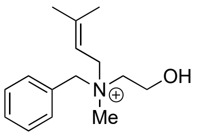
**Method P**	isocratic 50% B until 10 min; gradient from 40% to 90% B over next 3 min; isocratic 90% B for 2 min; gradient from 90% to 50% B over next 3 min; isocratic 50% B for 5 min (total run time 23 min)	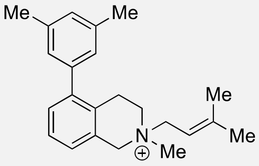

Determination of the enantiomeric ratio

Table S14. Methods used for the chiral phase HPLC

Method ID	Column	Description	used for substrates
**Method K**	Daicel OX-H, 250 mm x 4.6 mm, 5 µm	isocratic elution with 80% *n*-Heptane, 20% EtOH, 0.5% NEt_3, 0.3% CF_3CO_2H; flow 1 mL/min, T = 20 °C, wavelength 220 nm (total run time 40 min)	For all substrates except for the ones below
**Method L**	Daicel OZ-H, 250 mm x 4.6 mm, 5 µm	isocratic elution with 90% *n*-Heptane, 10% EtOH, 0.5% NEt_3, 0.3% CF_3CO_2H, flow 1 mL/min, T = 25 °C, wavelength 220 nm (total run time 60 min)	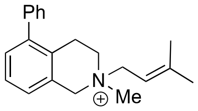, 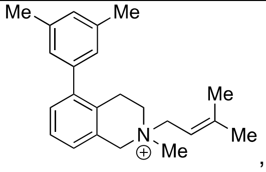, 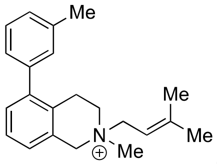, 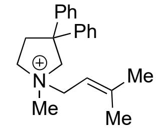, 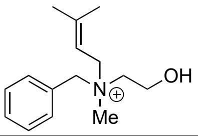
**Method M**	Daicel IB-N5, 250 mm x 4.6 mm, 5 µm	isocratic elution with 90% *n*-Heptane, 10% EtOH, 0.1% NEt_3, 0.3% CF_3CO_2H, flow 1 mL/min, T = 25 °C, wavelength 220 nm (total run time 40 min)	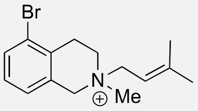

Preparative HPLC
Column: Agilent PrepHT XDB-C18 column (21.2 x 150 mm, 5 µm); solvents A (97% H_2O, 3% CH_3CN, 0.1% TFA) and B (CH_3CN, 0.1% TFA); flow = 10 mL/min; 0 min – 0% B; 2 min – 0% B; 5 min – 20% B; 30 min – 100% B; 36 min – 100% B).

LC-MS
Column: Agilent Zorbax RR Eclipse, XDB-C18 (4.6 × 75 mm, 3.5 µm); solvents A (97% H_2O, 3% CH_3CN, 0.1% TFA) and B (95% CH_3CN, 5% H_2O, 0.1% TFA); flow = 1 mL/min, T = 25 °C; 0 min – 0% B; 1 min – 0% B; 9 min – 95% B; 11 min – 95% B; 12 min – 0% B; 15 min – 0% B.

Calibration curves for HPLC-yield determination

Samples of tetraalkyl-ammonium bromides (0.1, 0.5, 1, 2, 5 and 10 mM; 15 and 20 mM) were prepared in triplicate. Stock solutions were prepared in 10 mL volumetric flasks in a 1:1 mixture of methanol-water. Internal standard (benzyl alcohol) was used as a stock solution in DMSO (296 mM, 20 µL were added to 1.0 mL of the ammonium salt solution).

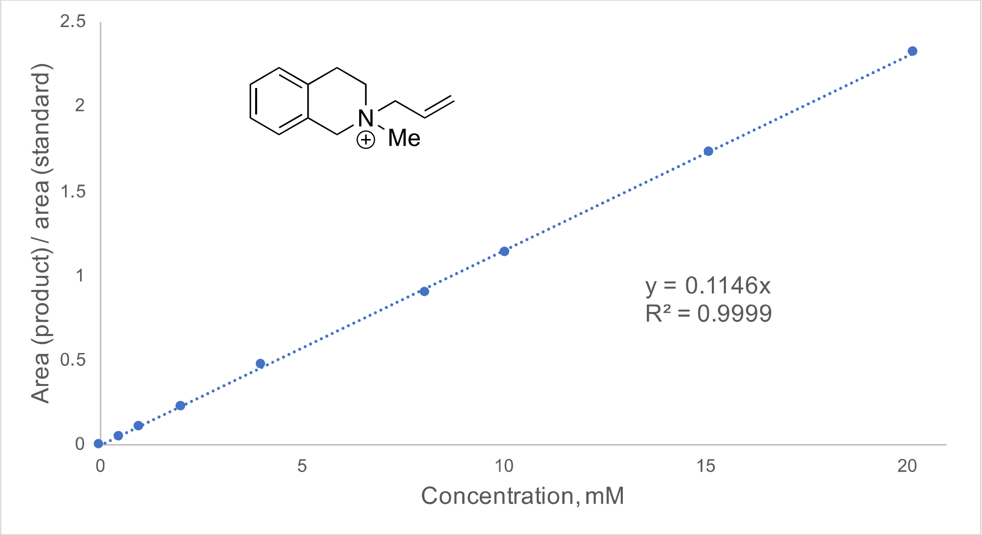

Fig. S24. Calibration curve of [**3ab**][Br] (0.1 – 20 mM) with benzyl alcohol as internal standard, determined by HPLC at 210 nm.

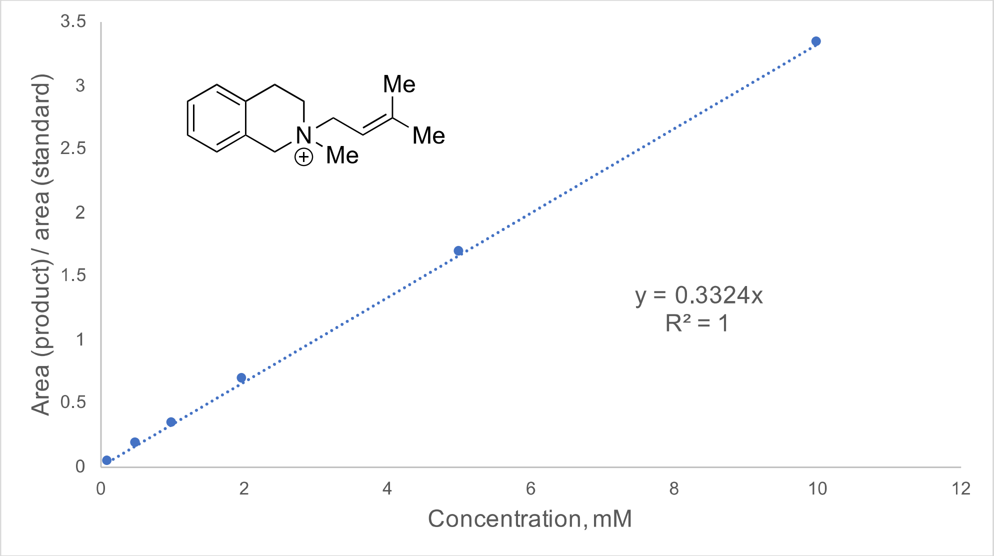

Fig. S25. Calibration curve of [**3aa**][Br] (0.1 – 20 mM) with benzyl alcohol as internal standard, determined by HPLC at 210 nm.

Response factors

Solutions of the same concentrations (3.0 – 7.0 mM) of the corresponding amine and ammonium salt were prepared in methanol in volumetric flasks. These solutions were mixed in 1:1, 1:2 and 2:1 ratios (each mixture prepared 2 times on a 0.75 or 1.0 mL scale) and analyzed by reversed phase HPLC.

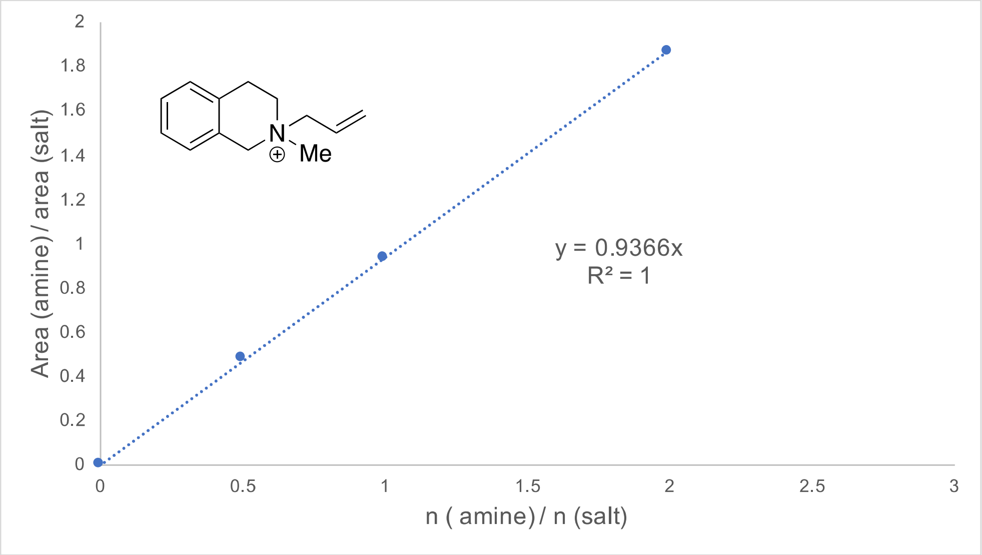

Fig. S26. Response factor curve of **3ab**[Br] to **1a**, determined by HPLC at 210 nm.

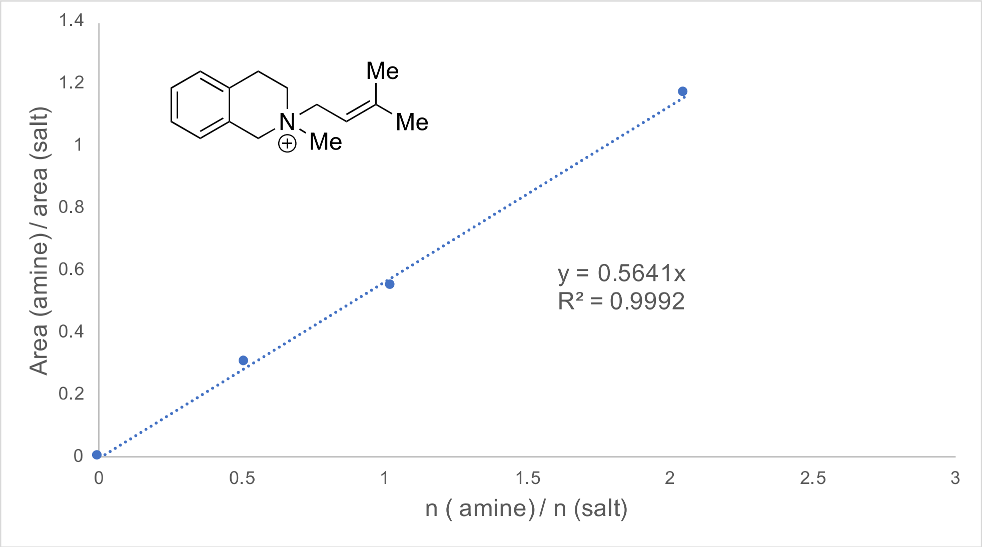

Fig. S27. Response factor curve of **3aa**[Br] to **1a**, determined by HPLC at 210 nm.

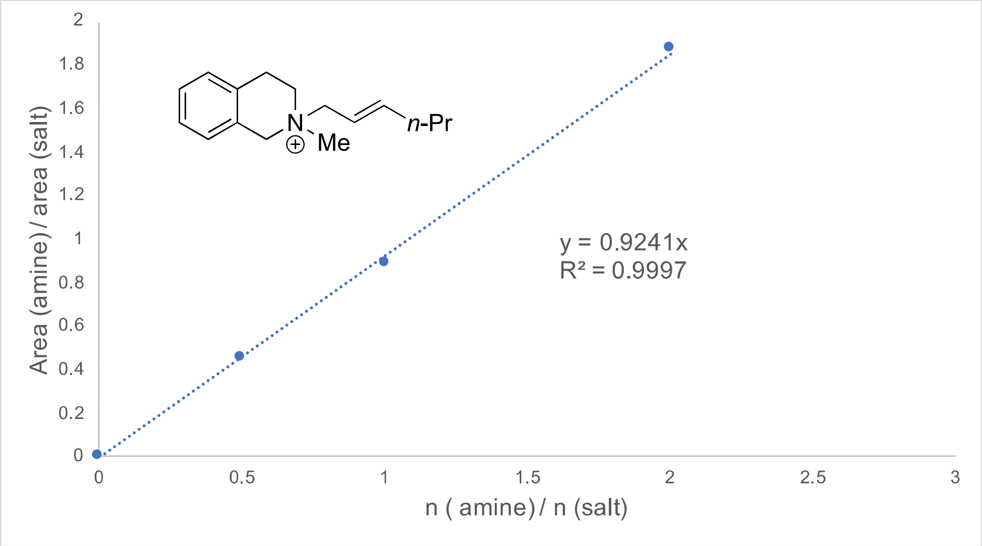

Fig. S28. Response factor curve of **3ag**[Br] to **1a**, determined by HPLC at 210 nm.

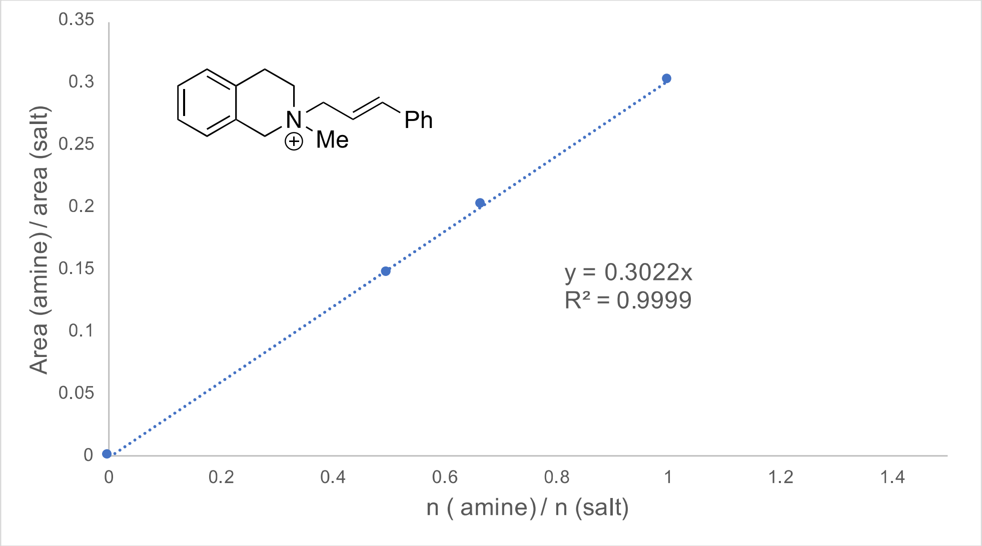

Fig. S29. Response factor curve of **3ae**[Br] to **1a**, determined by HPLC at 210 nm.

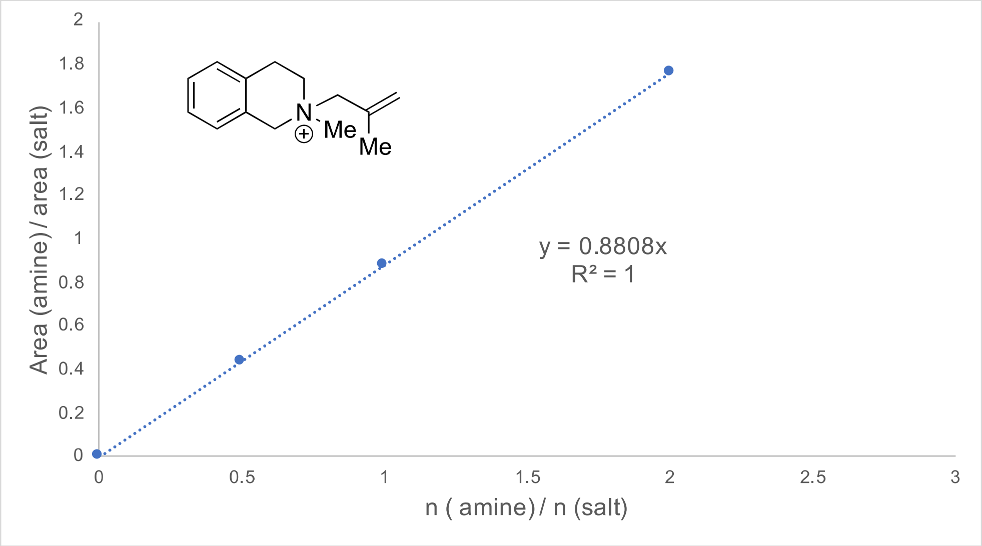

Fig. S30. Response factor curve of **3af**[Br] to **1a**, determined by HPLC at 210 nm.

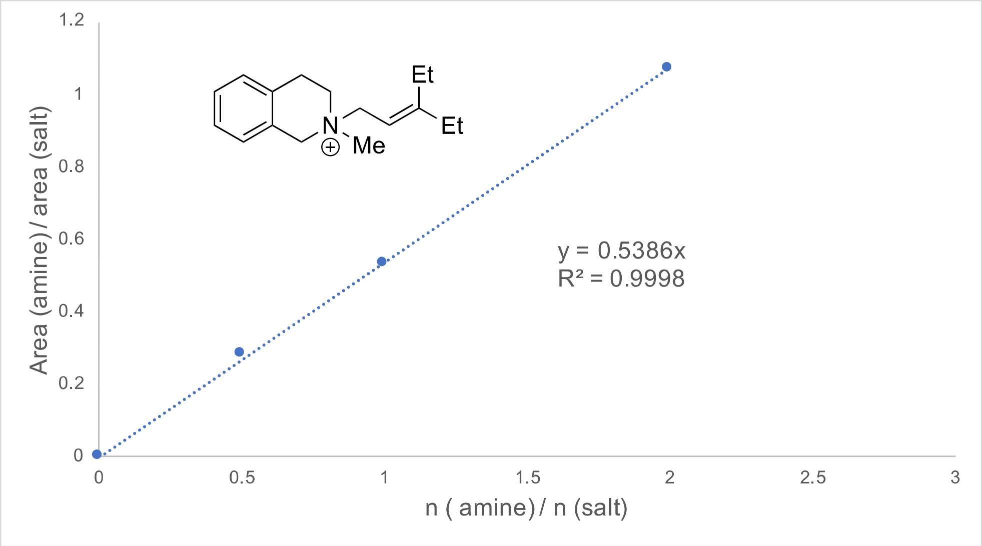

Fig. S31. Response factor curve of **3ac**[Br] to **1a**, determined by HPLC at 210 nm.

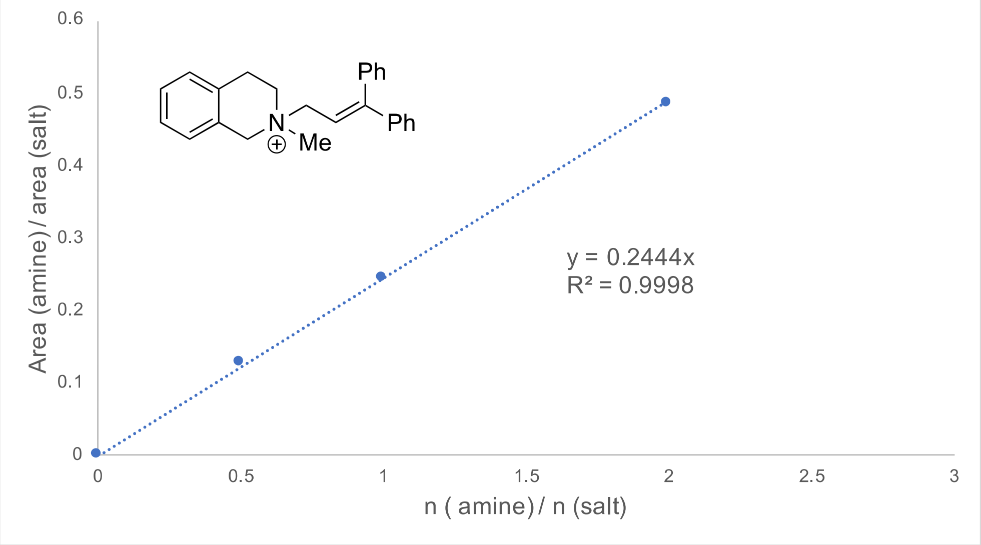

Fig. S32. Response factor curve of **3ad**[Br] to **1a**, determined by HPLC at 210 nm.

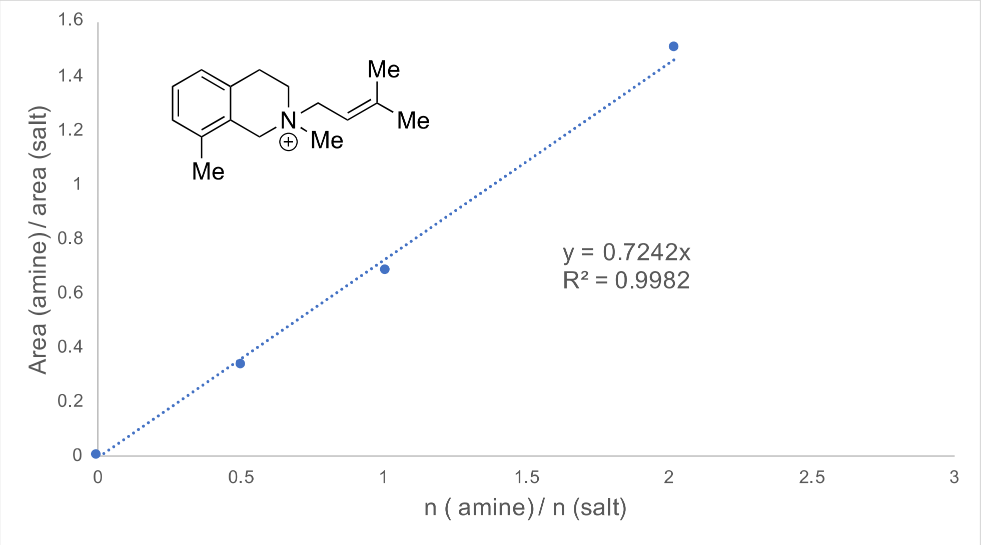

Fig. S33. Response factor curve of **3ba**[Br] to **1b**, determined by HPLC at 210 nm.

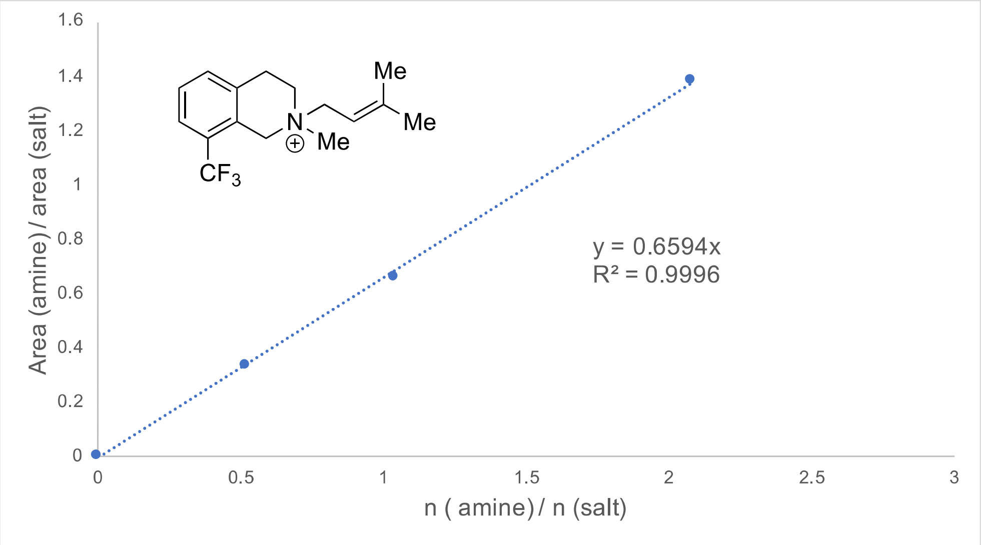

Fig. S34. Response factor curve of **3ca**[Br] to **1c**, determined by HPLC at 210 nm.

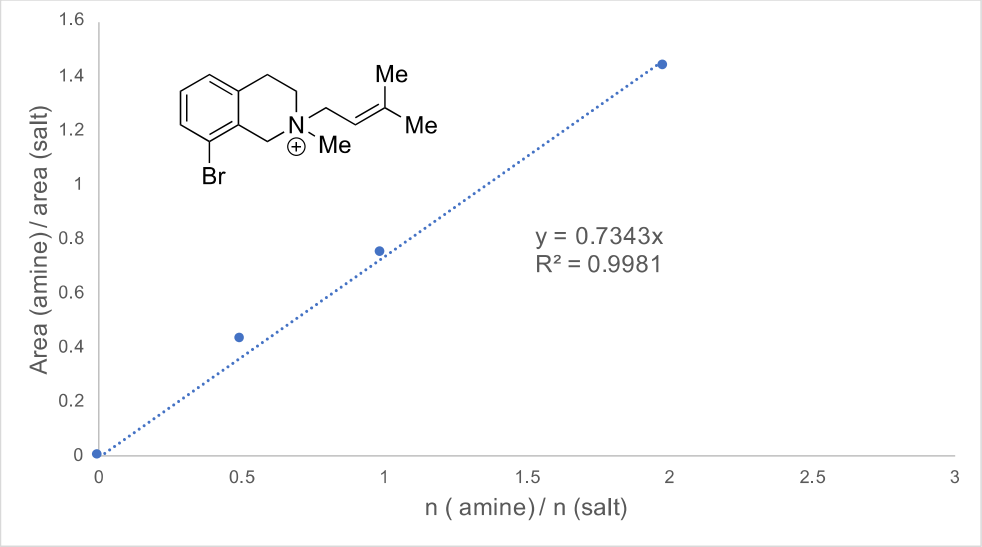

Fig. S35. Response factor curve of **3ea**[Br] to **1e**, determined by HPLC at 210 nm.

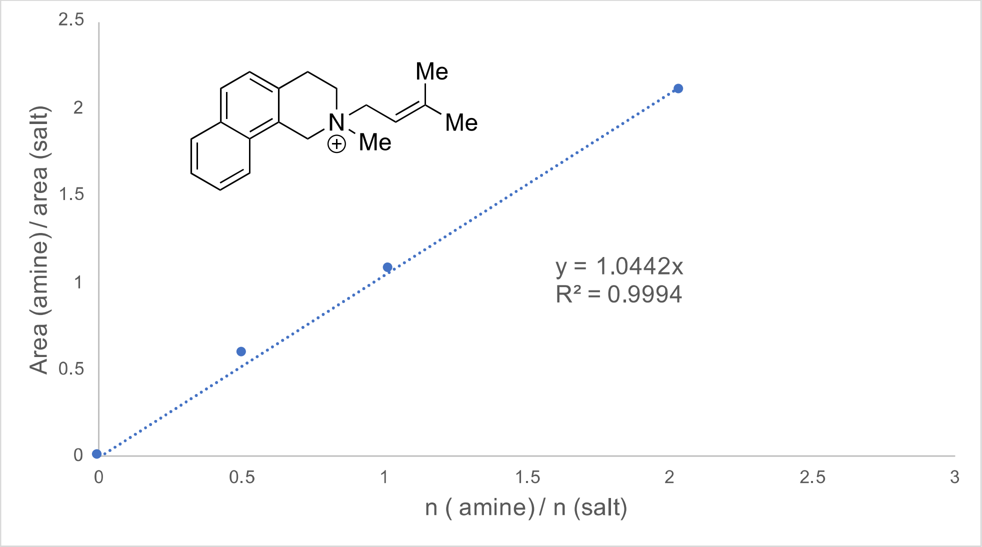

Fig. S36. Response factor curve of **3da**[Br] to **1d**, determined by HPLC at 210 nm.

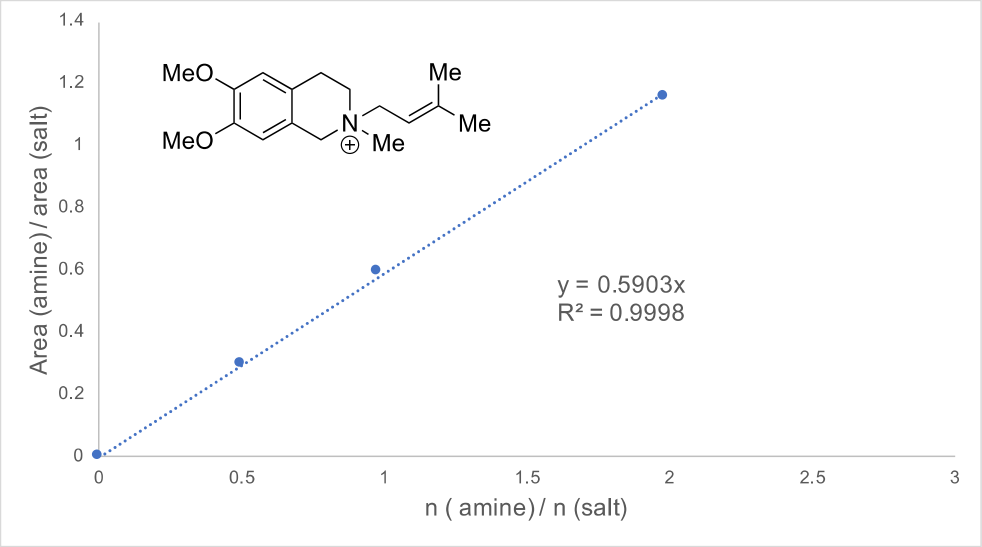

Fig. S37. Response factor curve of **3ga**[Br] to **1g**, determined by HPLC at 210 nm.

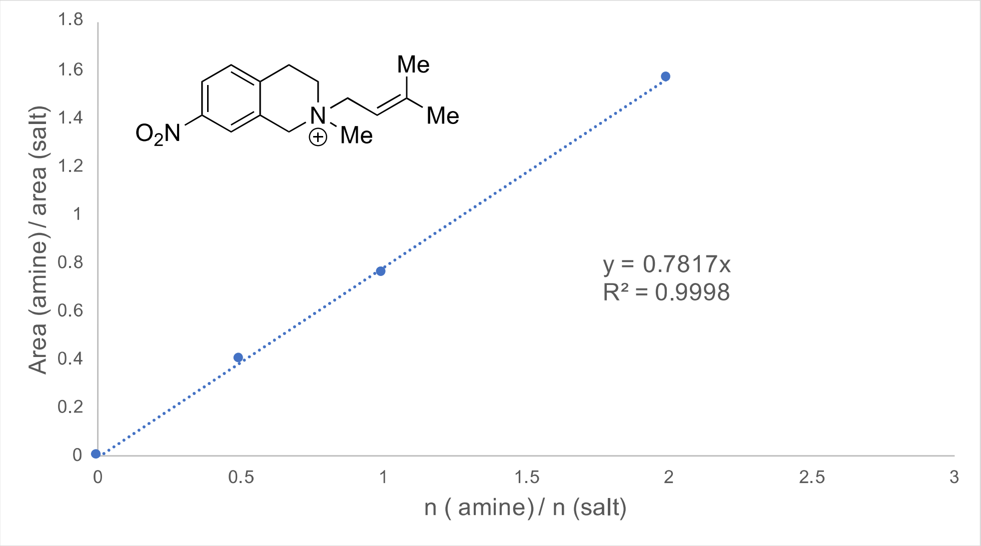

Fig. S38. Response factor curve of **3ha**[Br] to **1h**, determined by HPLC at 210 nm.

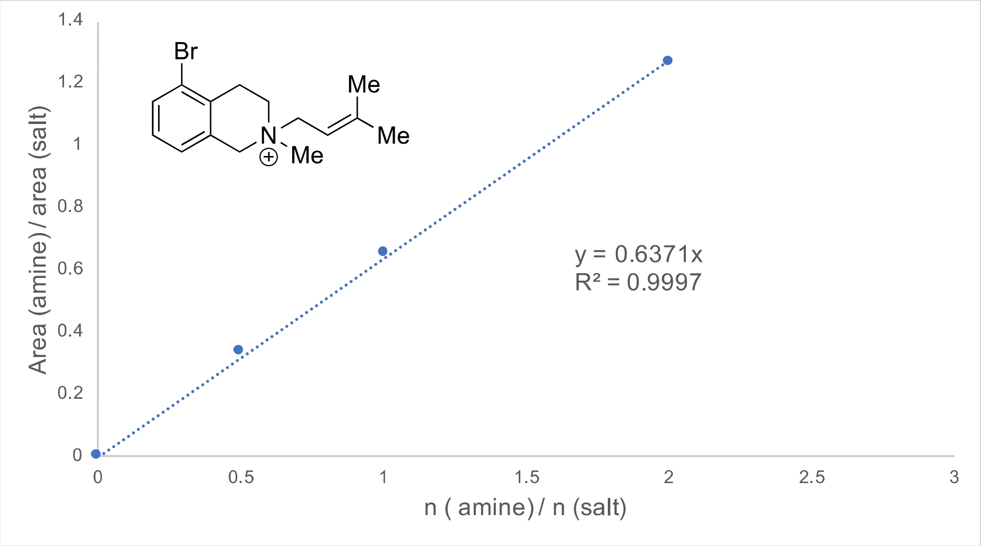

Fig. S39. Response factor curve of **3fa**[Br] to **1f**, determined by HPLC at 210 nm.

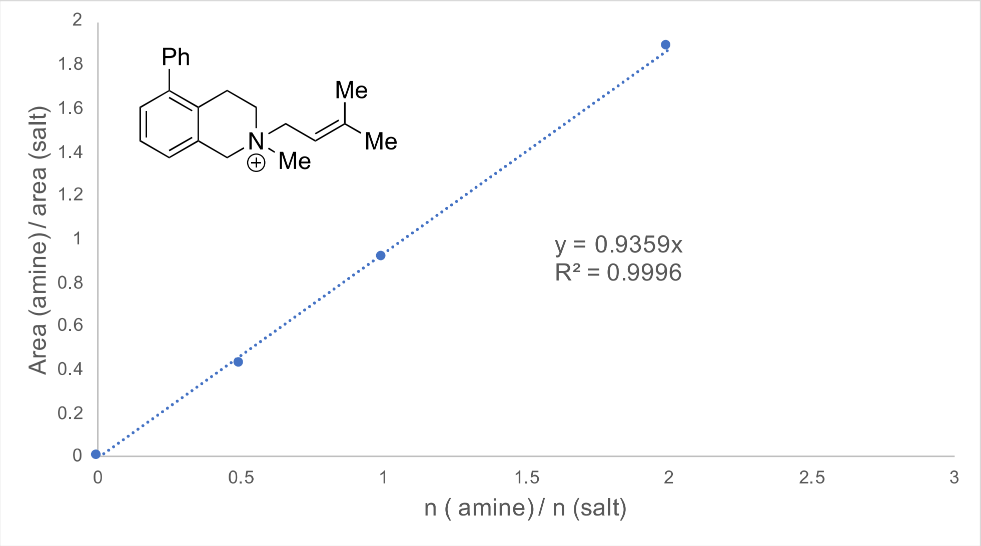

Fig. S40. Response factor curve of **3la**[Br] to **1l**, determined by HPLC at 210 nm.

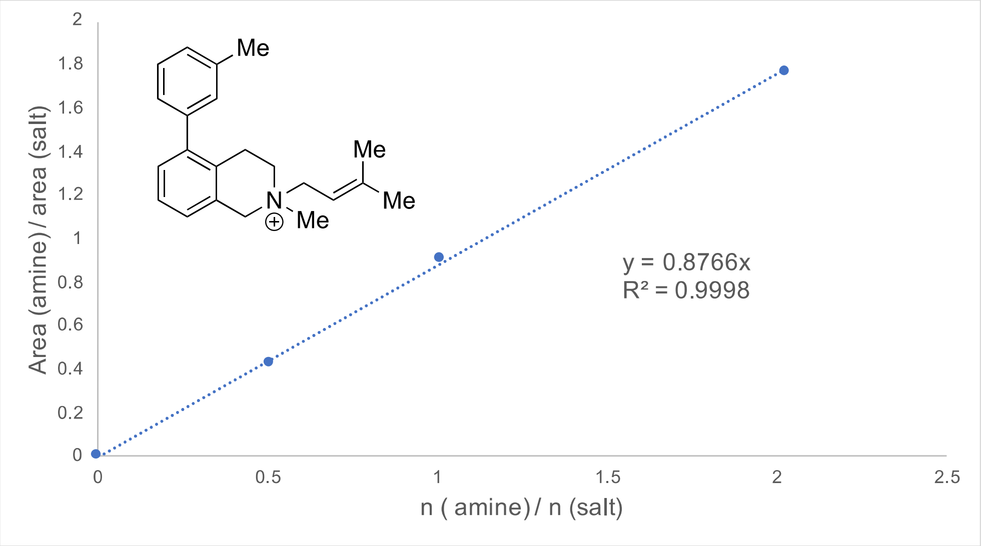

Fig. S41. Response factor curve of **3ma**[Br] to **1m**, determined by HPLC at 210 nm.

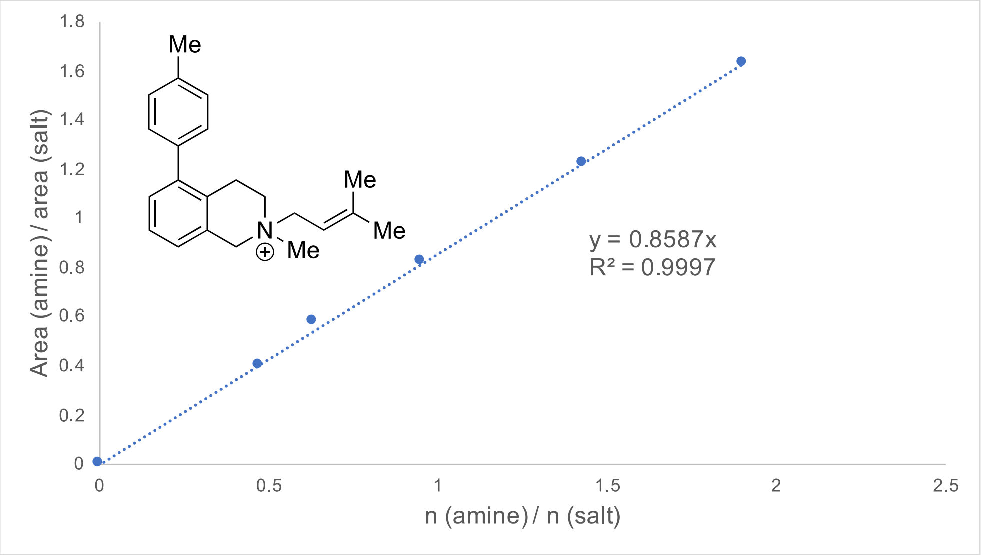

Fig. S42. Response factor curve of **3na**[Br] to **1n**, determined by HPLC at 210 nm.

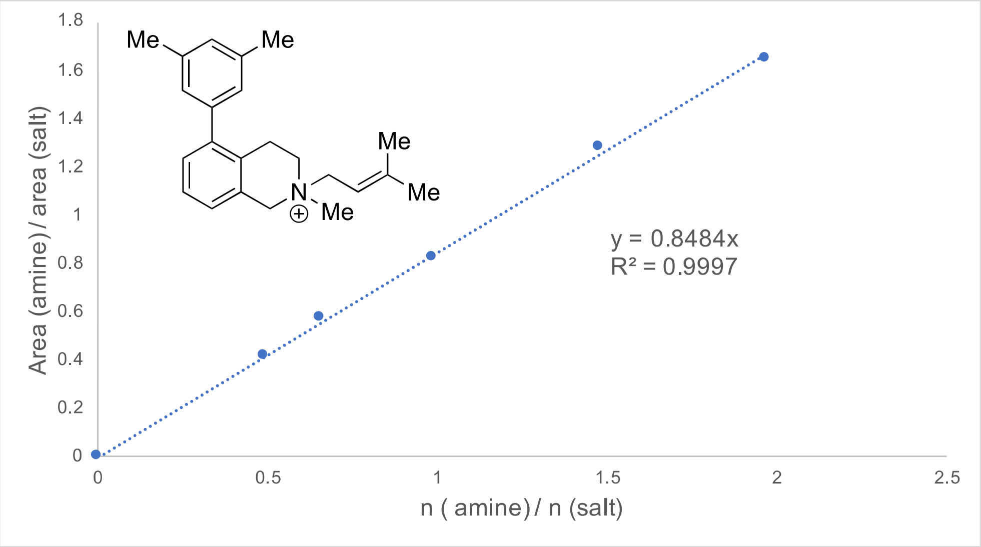

Fig. S43. Response factor curve of **3oa**[Br] to **1o**, determined by HPLC at 210 nm.

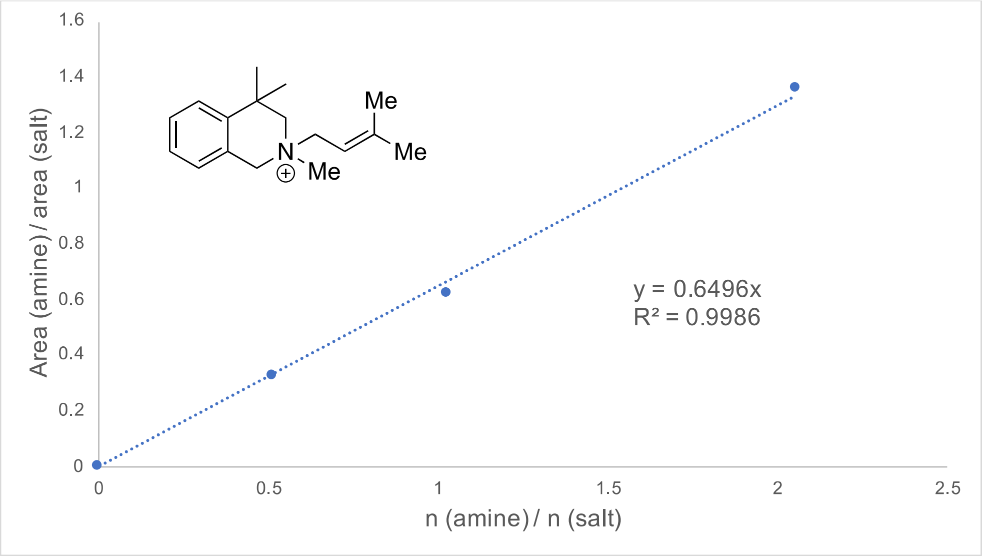

Fig. S44. Response factor curve of **3ia**[Br] to **1i**, determined by HPLC at 210 nm.

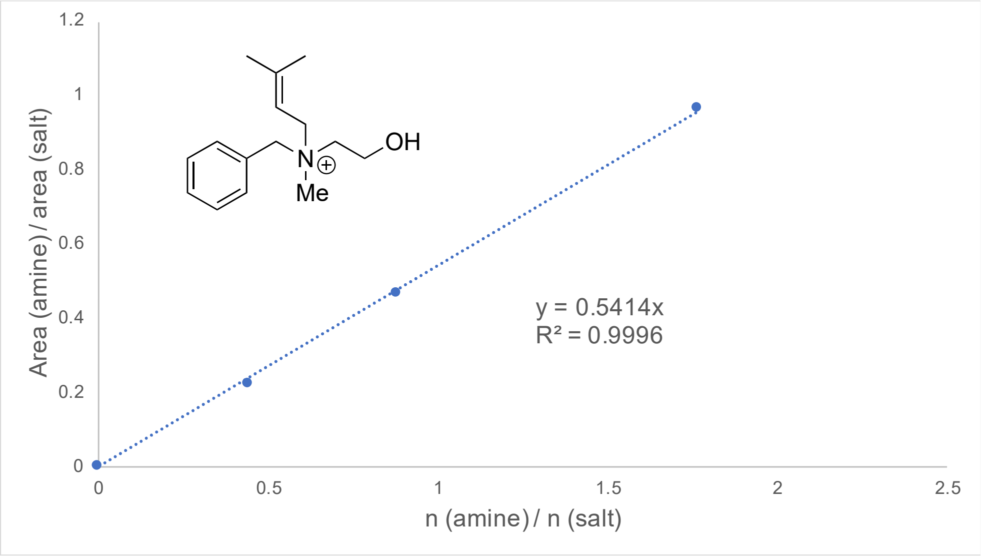

Fig. S45. Response factor curve of **3pa**[Br] to **1p**, determined by HPLC at 210 nm.

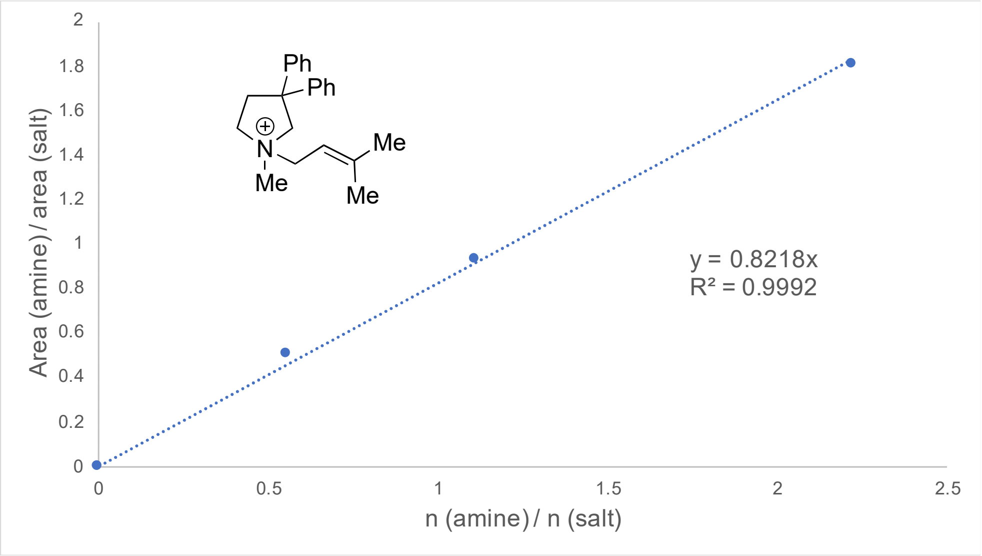

Fig. S46. Response factor curve of **3qa**[Br] to **1q**, determined by HPLC at 210 nm.

For analytical scale reactions where salts **3ea**, **3ka** and **3ra** (prepared by prep. catalysis see preparative scale reactions) were formed, conversions were determined by NMR:

Catalysis was run overnight (24 h) at 25 °C in the glovebox on the indicated scale. Subsequently, the solvent was removed under reduced pressure and an NMR sample was prepared in $CDCl_3$ with 1,3,5-trimethoxybenzene as an internal standard to determine the product concentration.

HPLC chromatograms

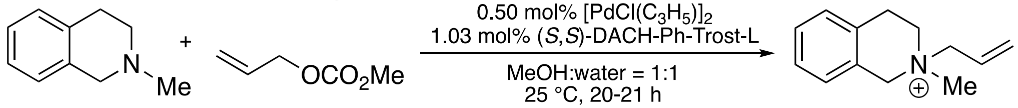

10 mM **3.0 eq**

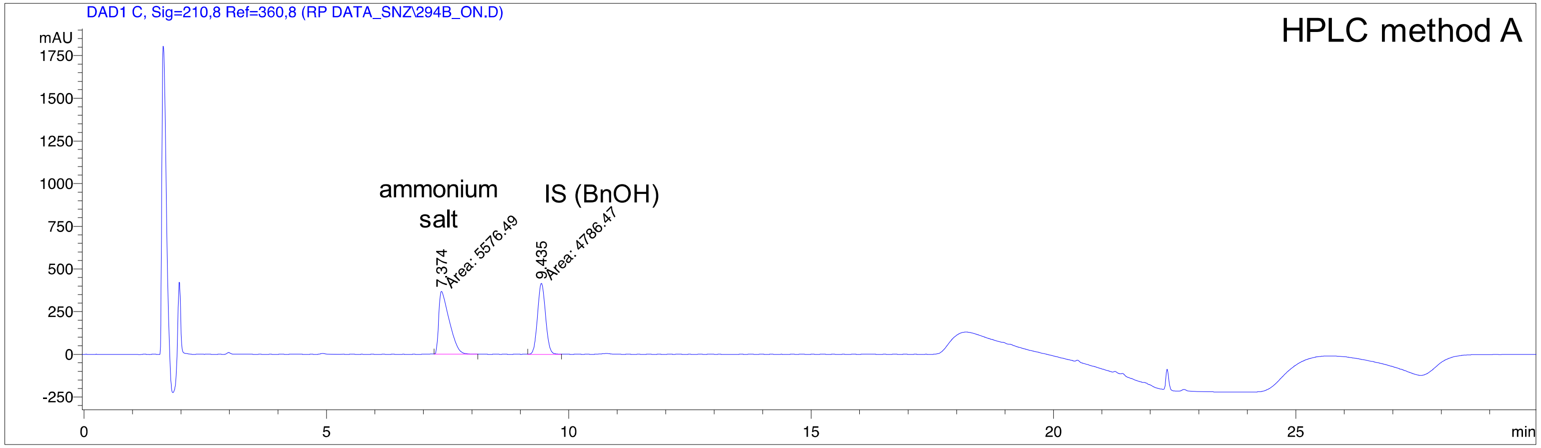

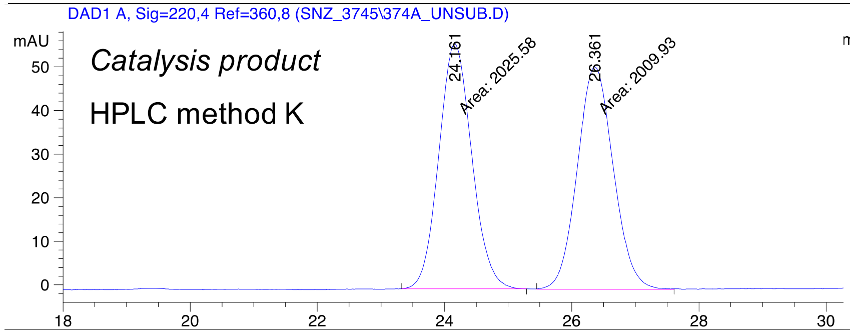

Signal 1: DAD1 A, Sig=220,4 Ref=360,8

Peak #	RetTime [min]	Type	Width [min]	Area [mAU*s]	Height [mAU]	Area %
1	24.161	MM	0.6009	2025.58289	56.18352	50.1940
2	26.361	MM	0.6569	2009.92822	50.99501	49.8060

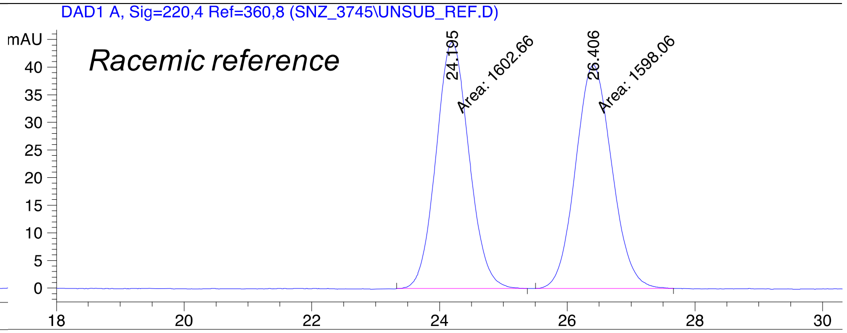

Signal 1: DAD1 A, Sig=220,4 Ref=360,8

Peak #	RetTime [min]	Type	Width [min]	Area [mAU*s]	Height [mAU]	Area %
1	24.195	MM	0.5989	1602.65784	44.60342	50.0718
2	26.406	MM	0.6587	1598.05920	40.43291	49.9282

Fig. S47. Reversed phase HPLC trace for the allylation of **1a** with **2b** (top); Chiral phase HPLC trace for the catalysis product **3ab** (bottom left) and for the racemic reference (bottom right). IS = internal standard.

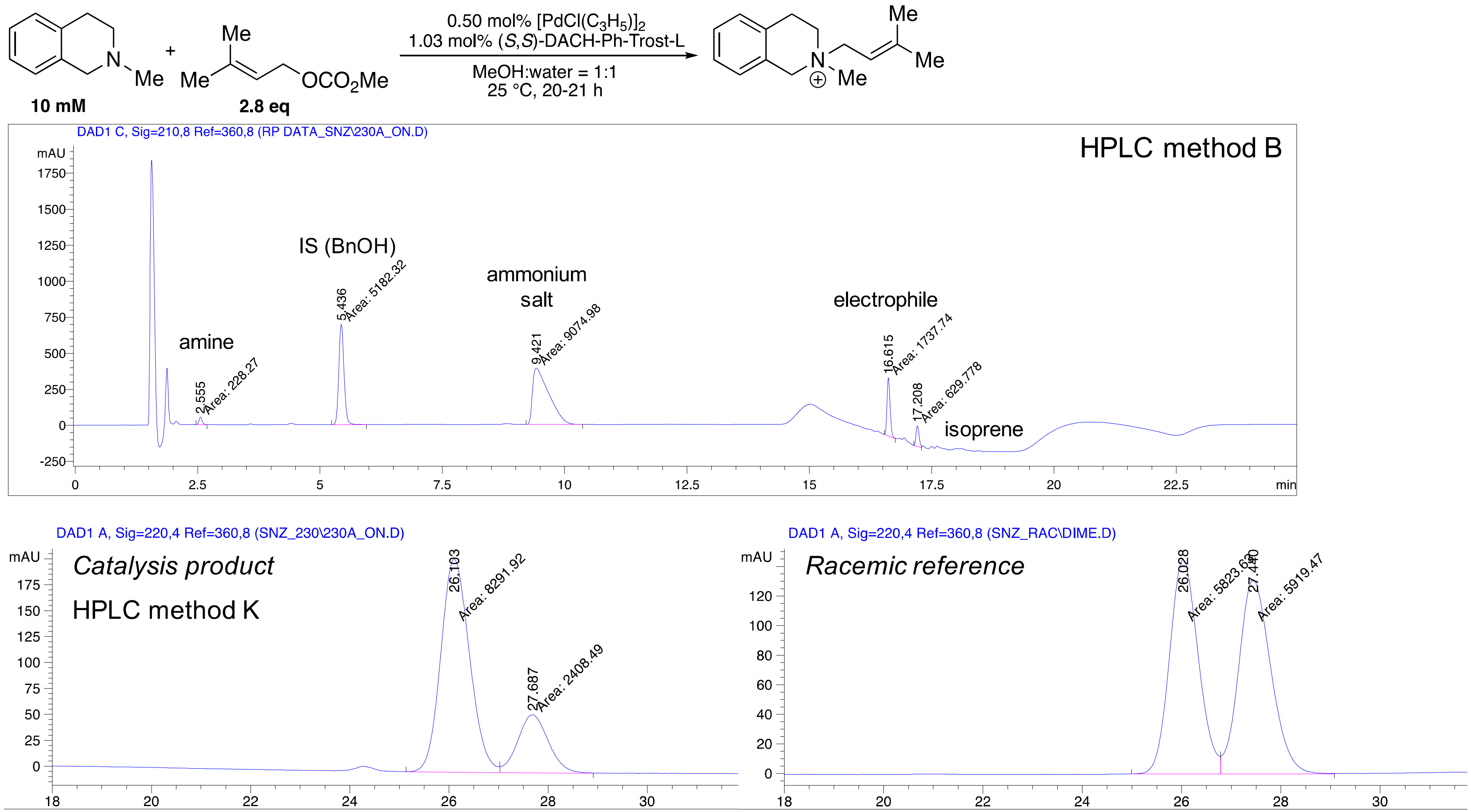

Signal 1: DAD1 A, Sig=220,4 Ref=360,8

Peak #	RetTime [min]	Type	Width [min]	Area [mAU*s]	Height [mAU]	Area %
1	26.103	MF	0.6731	8291.91699	205.31824	77.4916
2	27.687	FM	0.7173	2408.49414	55.96457	22.5084

Signal 1: DAD1 A, Sig=220,4 Ref=360,8

Peak #	RetTime [min]	Type	Width [min]	Area [mAU*s]	Height [mAU]	Area %
1	26.028	MF	0.6690	5823.62891	145.07658	49.5919
2	27.440	FM	0.7502	5919.46582	131.50609	50.4081

Fig. S48. Reversed phase HPLC trace for the allylation of **1a** with **2a** (top); Chiral phase HPLC trace for the catalysis product **3aa** (bottom left) and for the racemic reference (bottom right). IS = internal standard.

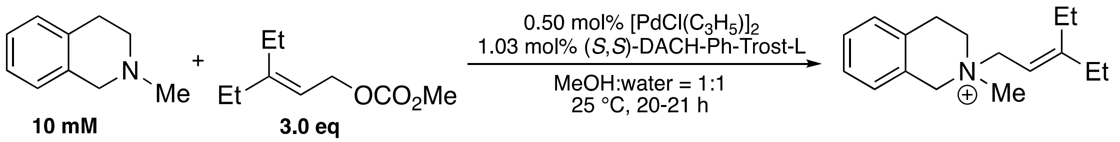

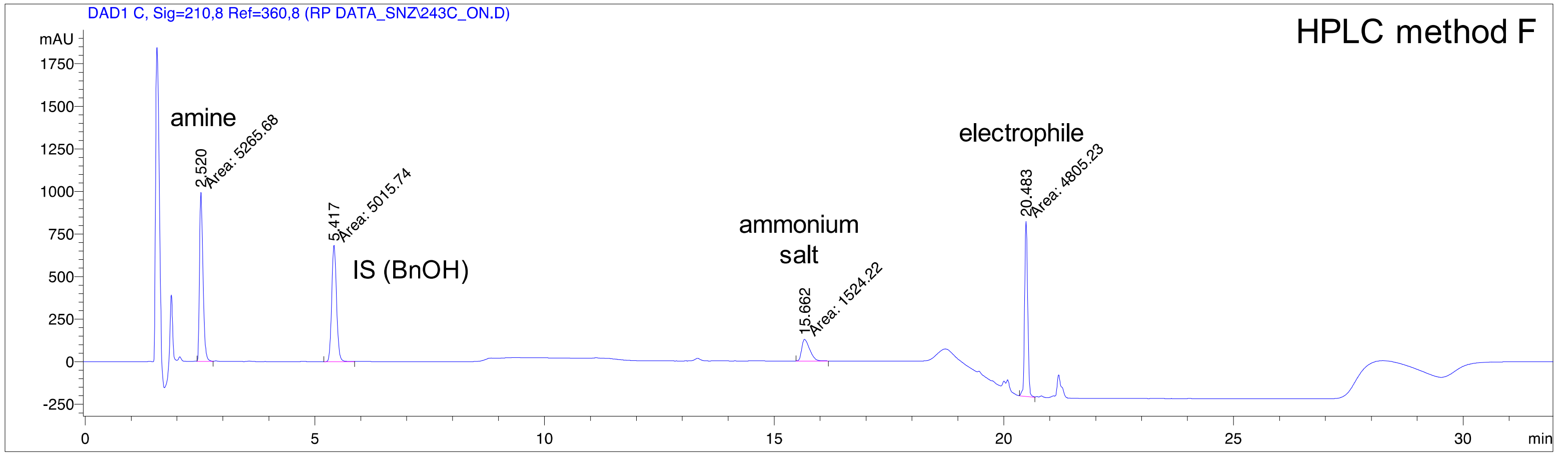

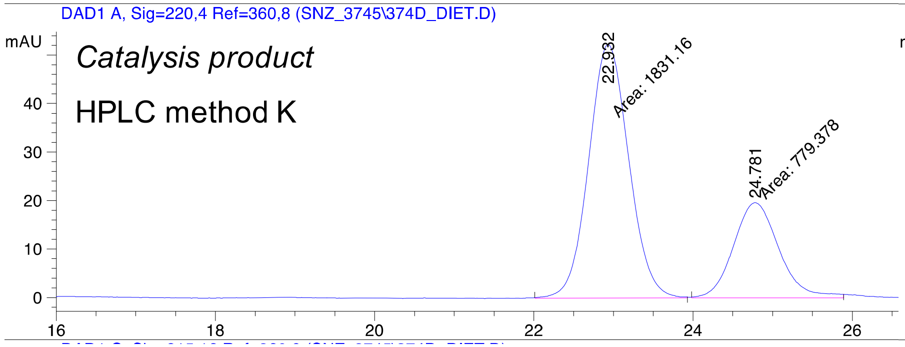

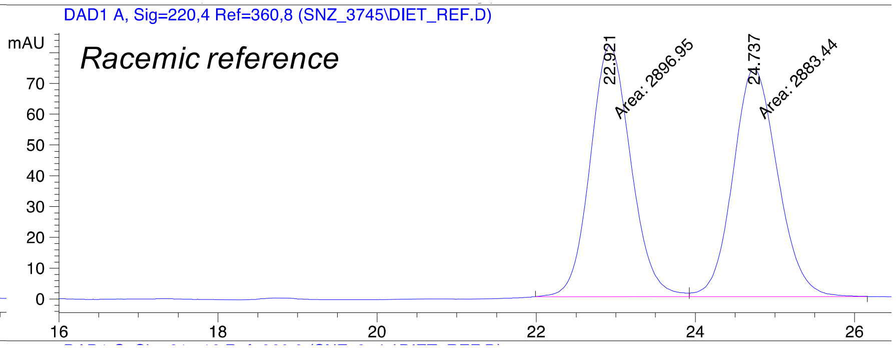

Signal 1: DAD1 A, Sig=220,4 Ref=360,8

Peak #	RetTime [min]	Type	Width [min]	Area [mAU*s]	Height [mAU]	Area %
1	22.932	MM	0.5838	1831.16235	52.27705	70.1449
2	24.781	MM	0.6616	779.37830	19.63442	29.8551

Signal 1: DAD1 A, Sig=220,4 Ref=360,8

Peak #	RetTime [min]	Type	Width [min]	Area [mAU*s]	Height [mAU]	Area %
1	22.921	MF	0.5925	2896.95361	81.49546	50.1169
2	24.737	FM	0.6488	2883.43726	74.06576	49.8831

Fig. S49. Reversed phase HPLC trace for the allylation of **1a** with **2c** (top); Chiral phase HPLC trace for the catalysis product **3ac** (bottom left) and for the racemic reference (bottom right). IS = internal standard.

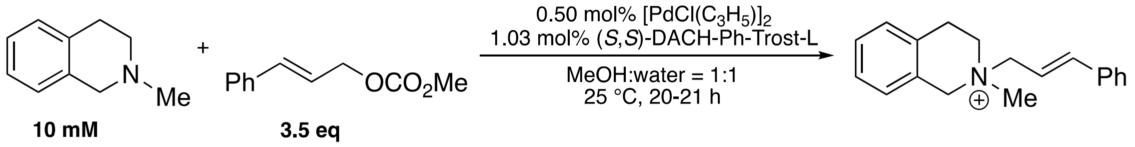

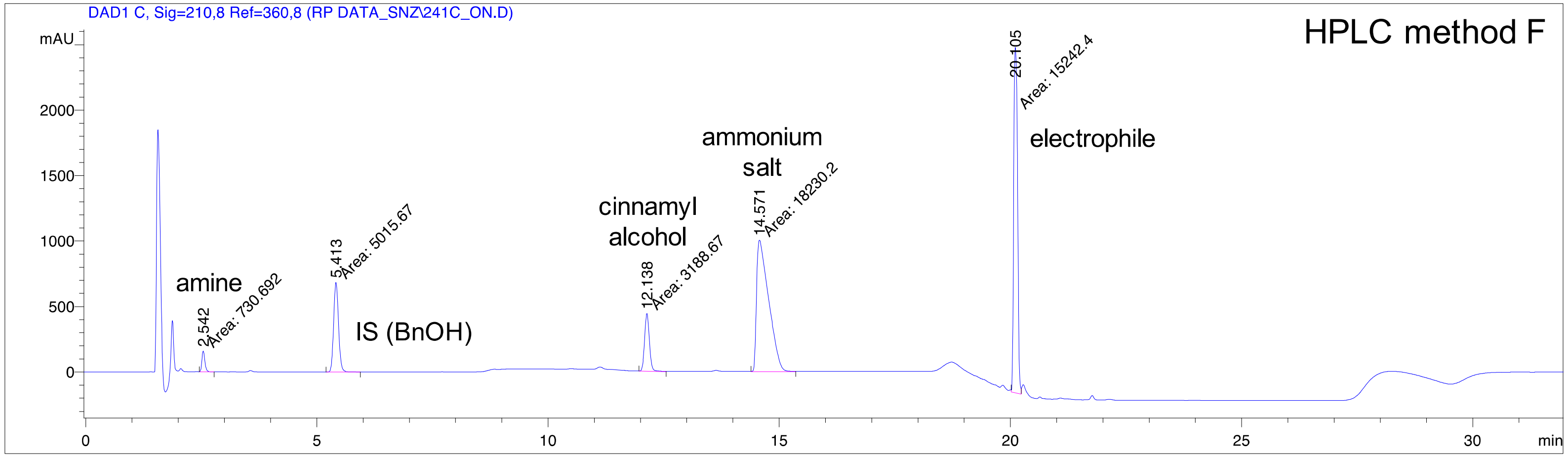

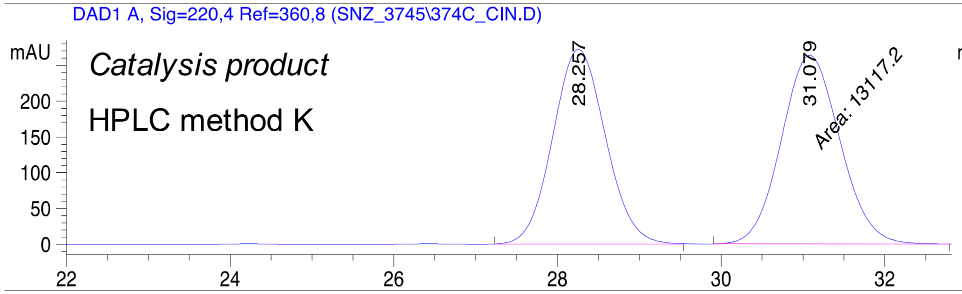

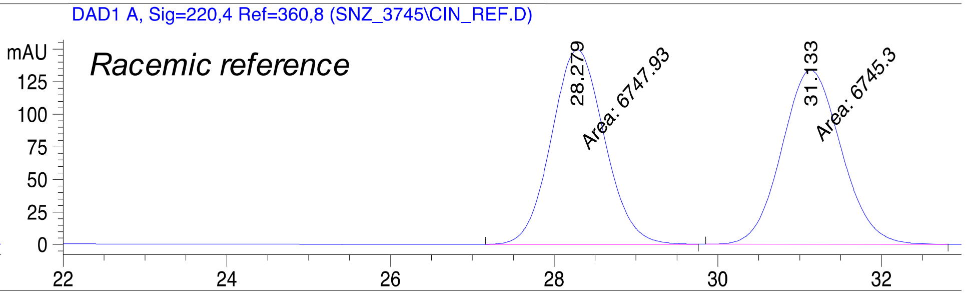

Signal 1: DAD1 A, Sig=220,4 Ref=360,8

Peak #	RetTime [min]	Type	Width [min]	Area [mAU*s]	Height [mAU]	Area %
1	28.257	BB	0.6977	1.22049e4	271.89798	48.1985
2	31.079	MM	0.8293	1.31172e4	263.60962	51.8015

Signal 1: DAD1 A, Sig=220,4 Ref=360,8

Peak #	RetTime [min]	Type	Width [min]	Area [mAU*s]	Height [mAU]	Area %
1	28.279	MM	0.7523	6747.93115	149.49838	50.0097
2	31.133	MM	0.8412	6745.30420	133.65192	49.9903

Fig. S50. Reversed phase HPLC trace for the allylation of **1a** with **2e** (top); Chiral phase HPLC trace for the catalysis product **3ae** (bottom left) and for the racemic reference (bottom right). IS = internal standard.

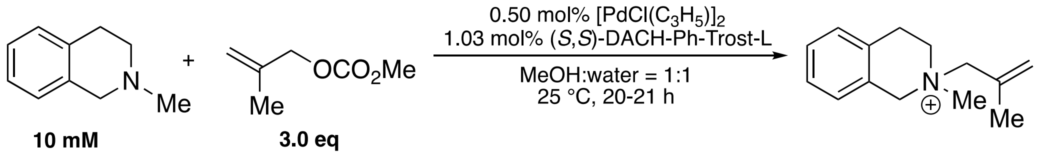

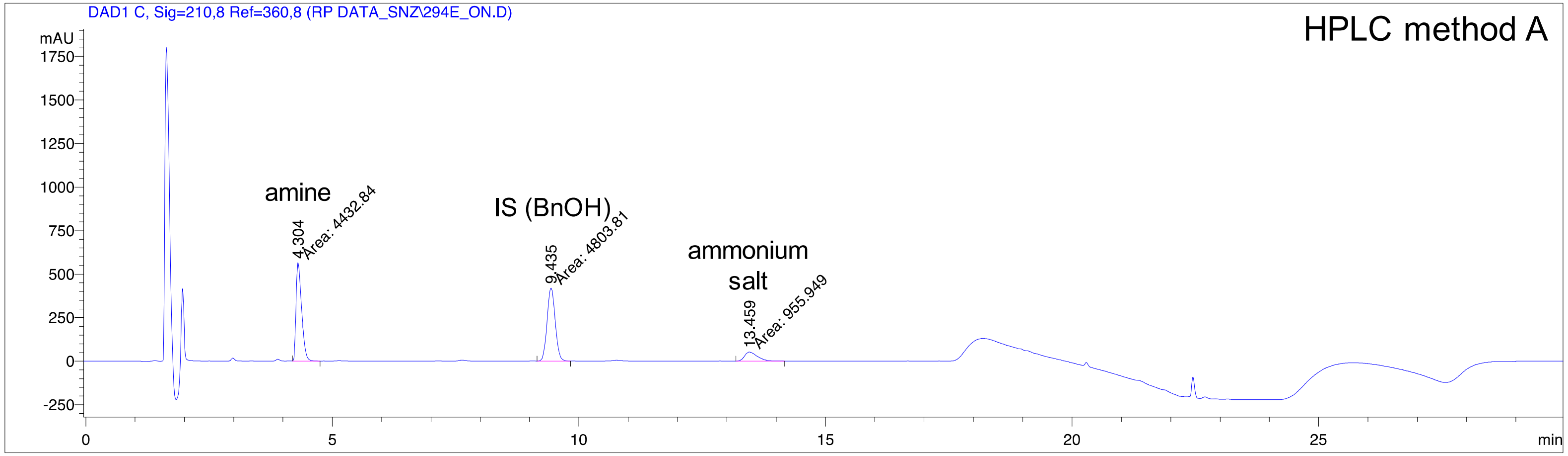

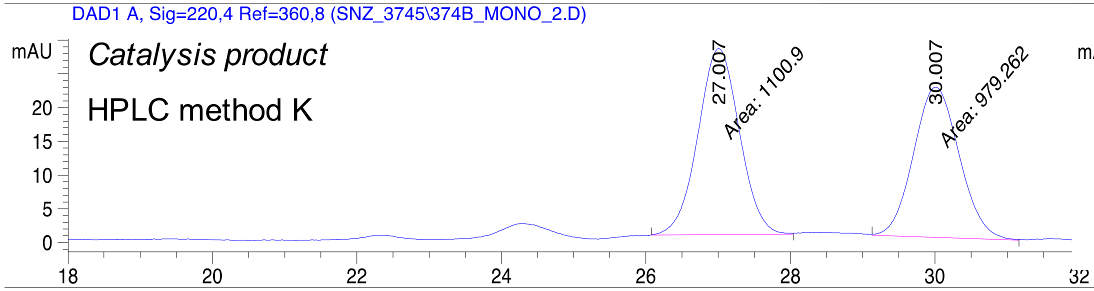

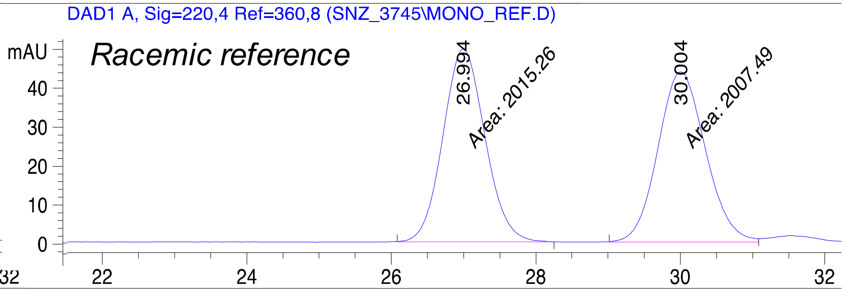

Signal 1: DAD1 A, Sig=220,4 Ref=360,8

Peak #	RetTime [min]	Type	Width [min]	Area [mAU*s]	Height [mAU]	Area %
1	27.007	MM	0.6656	1100.89612	27.56786	52.9237
2	30.007	MM	0.7335	979.26178	22.24988	47.0763

Signal 1: DAD1 A, Sig=220,4 Ref=360,8

Peak #	RetTime [min]	Type	Width [min]	Area [mAU*s]	Height [mAU]	Area %
1	26.994	MM	0.6742	2015.25574	49.81576	50.0965
2	30.004	MM	0.7620	2007.49146	43.90829	49.9035

Fig. S51. Reversed phase HPLC trace for the allylation of **1a** with **2f** (top); Chiral phase HPLC trace for the catalysis product **3af** (bottom left) and for the racemic reference (bottom right). IS = internal standard.

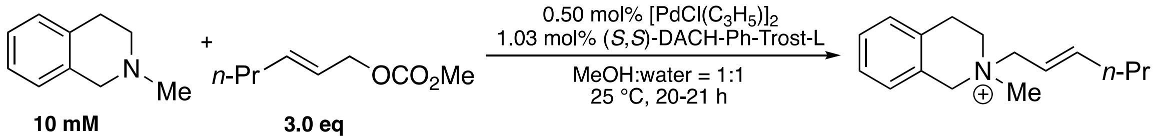

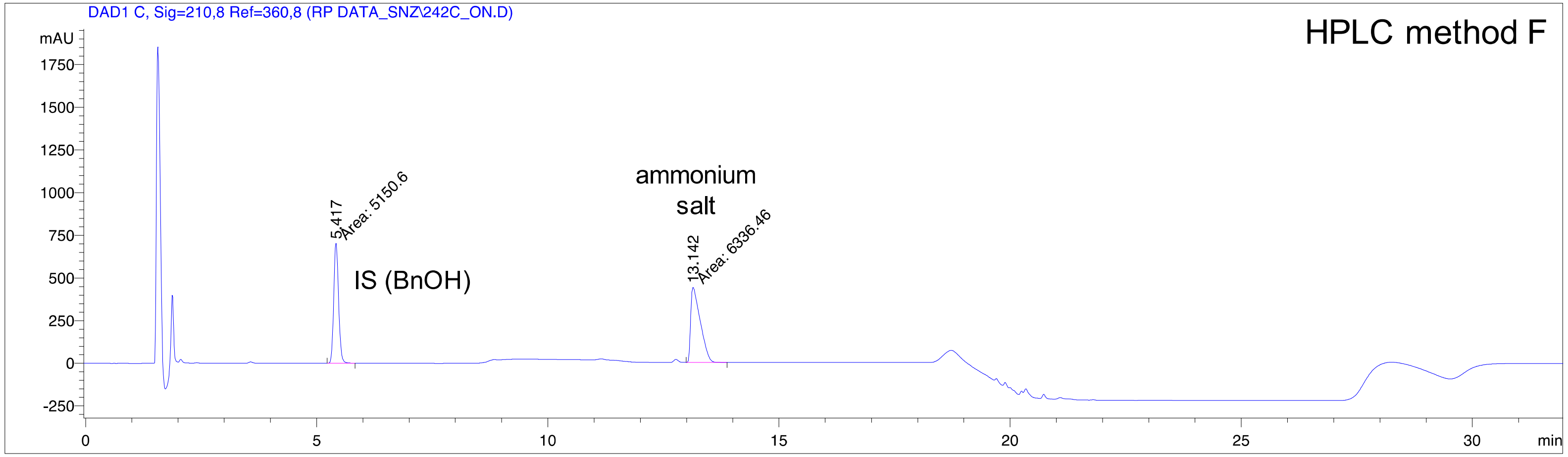

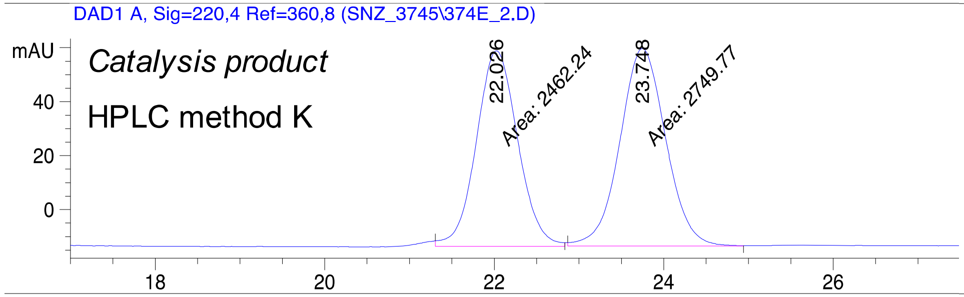

Signal 1: DAD1 A, Sig=220,4 Ref=360,8

Peak #	RetTime [min]	Type	Width [min]	Area [mAU*s]	Height [mAU]	Area %
1	22.026	MM	0.5656	2462.24072	72.56046	47.2417
2	23.748	MM	0.6260	2749.76733	73.21357	52.7583

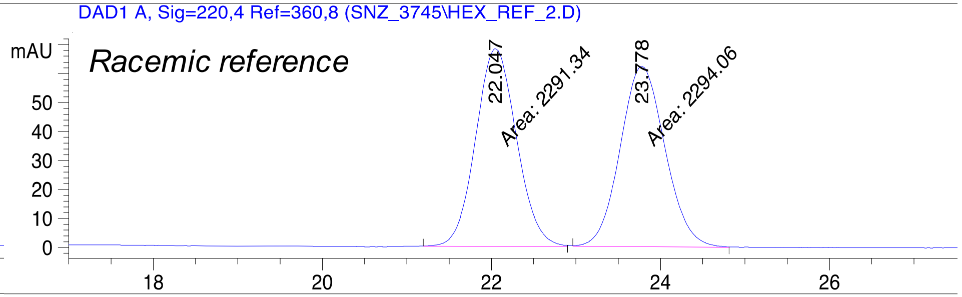

Signal 1: DAD1 A, Sig=220,4 Ref=360,8

Peak #	RetTime [min]	Type	Width [min]	Area [mAU*s]	Height [mAU]	Area %
1	22.047	MM	0.5596	2291.33789	68.24258	49.9704
2	23.778	MM	0.6139	2294.05518	62.28495	50.0296

Fig. S52. Reversed phase HPLC trace for the allylation of **1a** with **2g** (top); Chiral phase HPLC trace for the catalysis product **3ag** (bottom left) and for the racemic reference (bottom right). IS = internal standard.

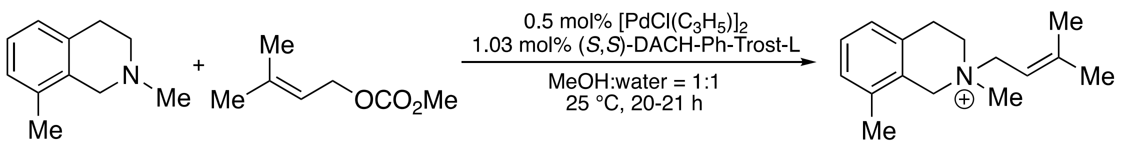

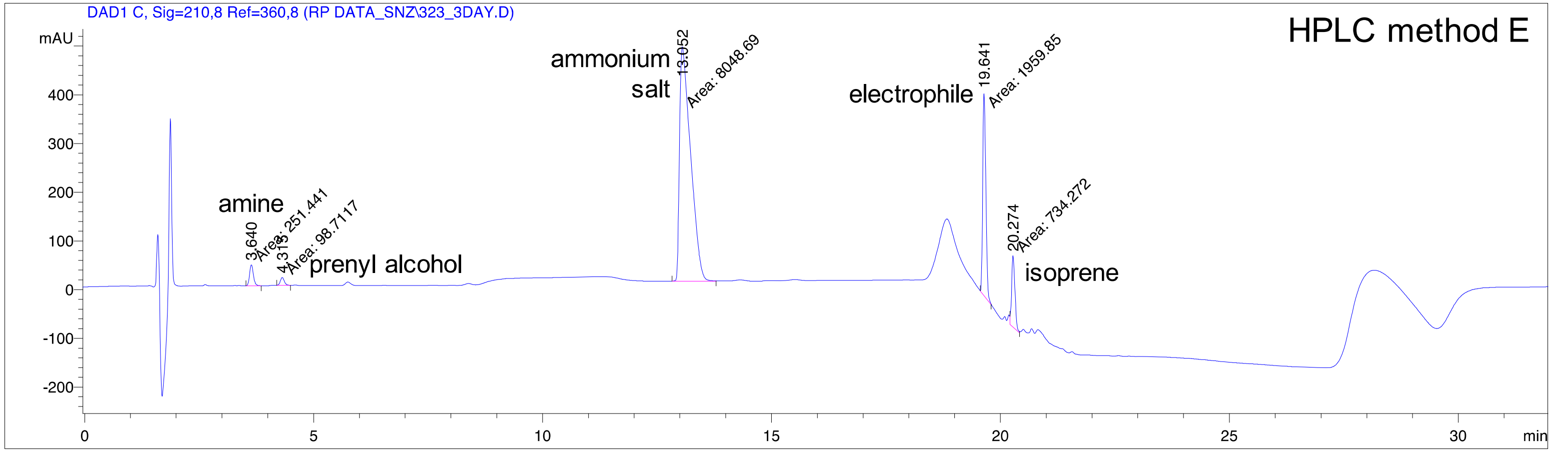

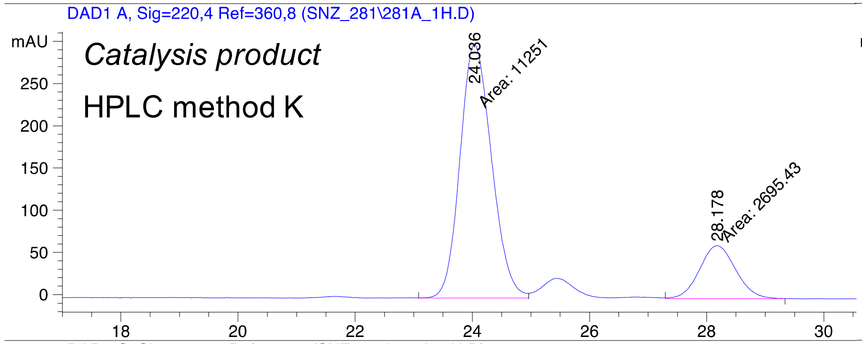

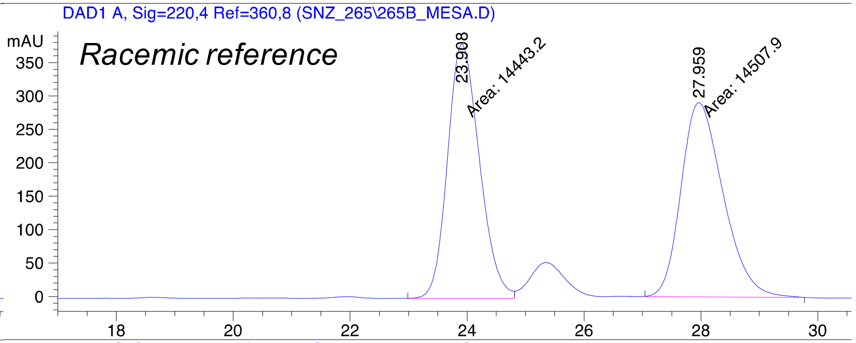

Signal 1: DAD1 A, Sig=220,4 Ref=360,8

Peak #	RetTime [min]	Type	Width [min]	Area [mAU*s]	Height [mAU]	Area %
1	24.036	MM	0.6246	1.12510e4	300.22961	80.6730
2	28.178	MM	0.7164	2695.43262	62.71111	19.3270

Signal 1: DAD1 A, Sig=220,4 Ref=360,8

Peak #	RetTime [min]	Type	Width [min]	Area [mAU*s]	Height [mAU]	Area %
1	23.908	MM	0.6327	1.44432e4	380.45798	49.8882
2	27.959	MM	0.8329	1.45079e4	290.29468	50.1118

Fig. S53. Reversed phase HPLC trace for the allylation of **1b** with **2a** (top); Chiral phase HPLC trace for the catalysis product **3ba** (bottom left) and for the racemic reference (bottom right). IS = internal standard.

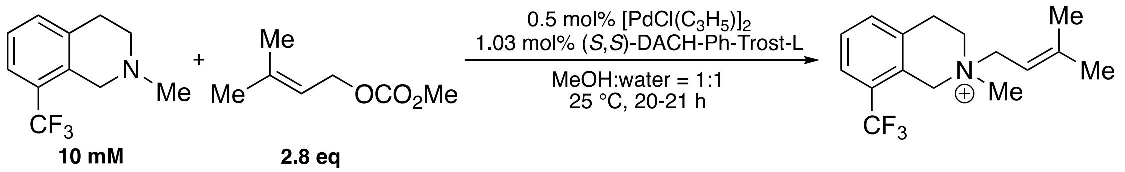

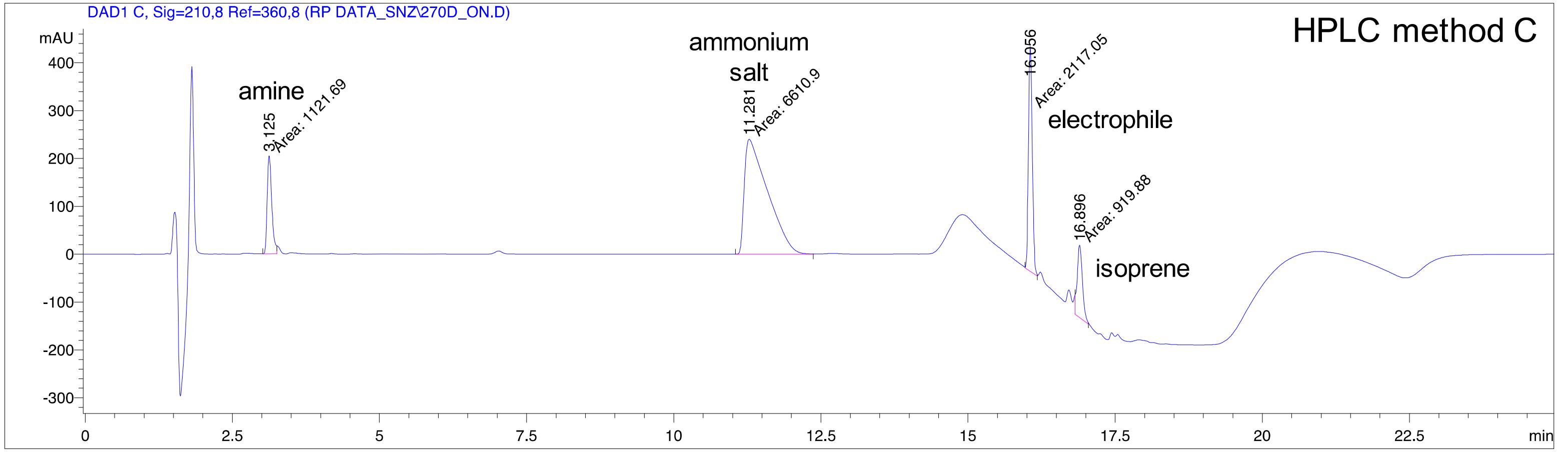

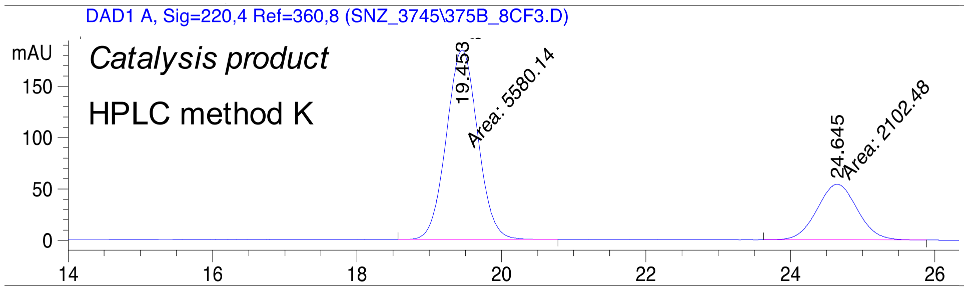

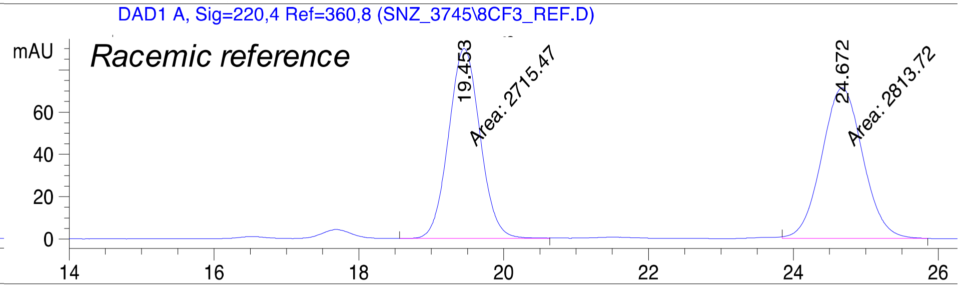

Signal 1: DAD1 A, Sig=220,4 Ref=360,8

Peak #	RetTime [min]	Type	Width [min]	Area [mAU*s]	Height [mAU]	Area %
1	19.453	MM	0.5045	5580.14111	184.34343	72.6333
2	24.645	MM	0.6465	2102.47925	54.20279	27.3667

Signal 1: DAD1 A, Sig=220,4 Ref=360,8

Peak #	RetTime [min]	Type	Width [min]	Area [mAU*s]	Height [mAU]	Area %
1	19.453	MM	0.5034	2715.46777	89.90108	49.1115
2	24.672	MM	0.6584	2813.72485	71.22749	50.8885

Fig. S54. Reversed phase HPLC trace for the allylation of **1c** with **2a** (top); Chiral phase HPLC trace for the catalysis product **3ca** (bottom left) and for the racemic reference (bottom right). IS = internal standard.

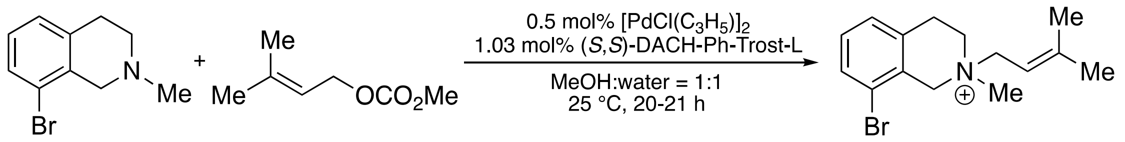

10 mM **2.8 eq**

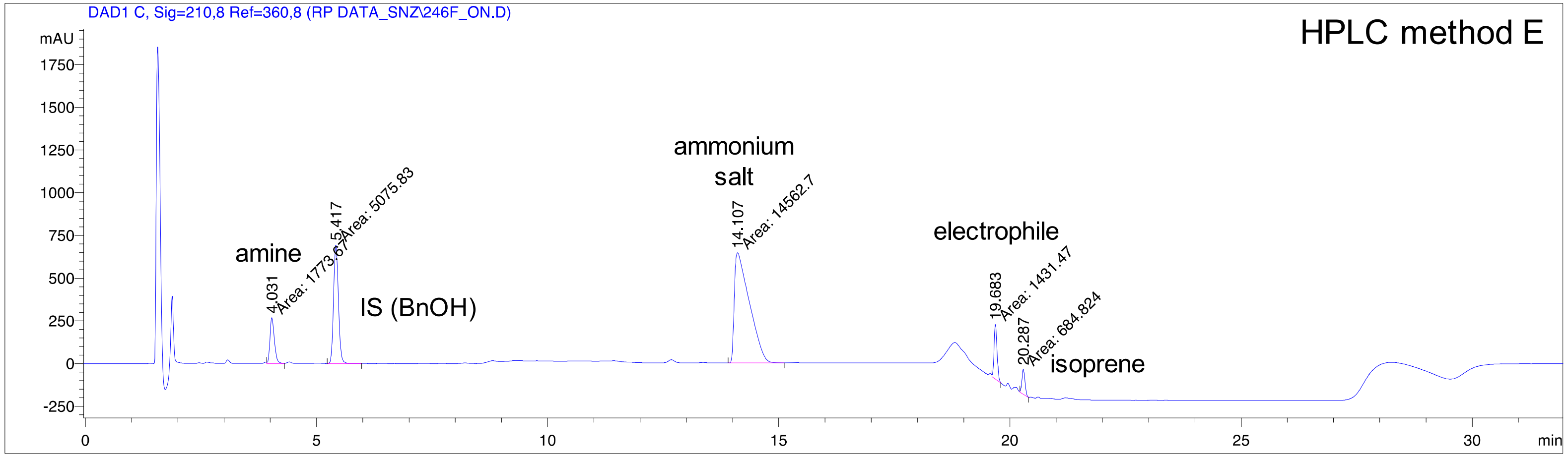

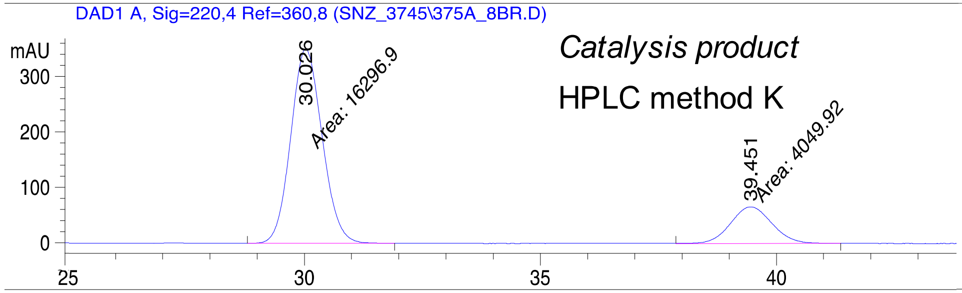

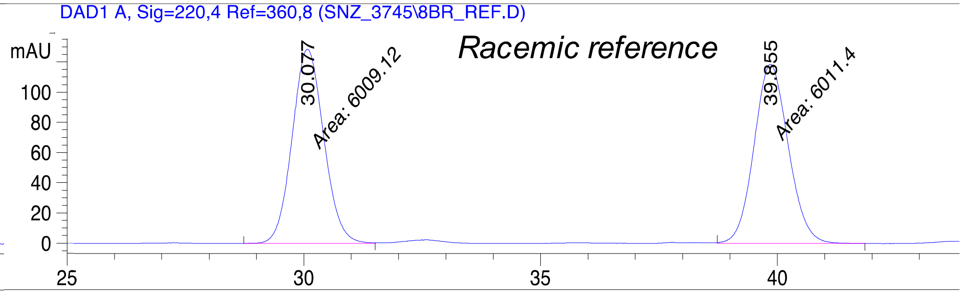

Signal 1: DAD1 A, Sig=220,4 Ref=360,8

Peak #	RetTime [min]	Type	Width [min]	Area [mAU*s]	Height [mAU]	Area %
1	30.026	MM	0.7720	1.62969e4	351.82727	80.0956
2	39.451	MM	1.0179	4049.91724	66.31435	19.9044

Signal 1: DAD1 A, Sig=220,4 Ref=360,8

Peak #	RetTime [min]	Type	Width [min]	Area [mAU*s]	Height [mAU]	Area %
1	30.077	MM	0.7758	6009.12158	129.10040	49.9905
2	39.855	MM	0.8408	6011.39893	119.15328	50.0095

Fig. S55. Reversed phase HPLC trace for the allylation of **1e** with **2a** (top); Chiral phase HPLC trace for the catalysis product **3ea** (bottom left) and for the racemic reference (bottom right). IS = internal standard.

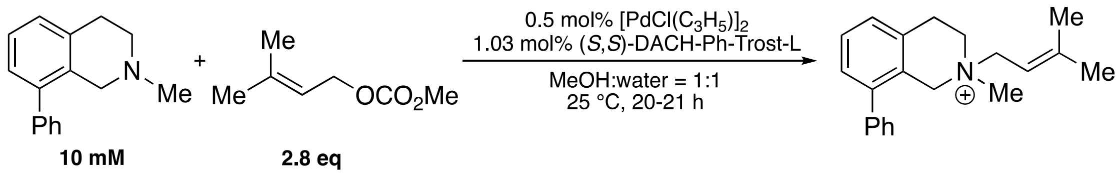

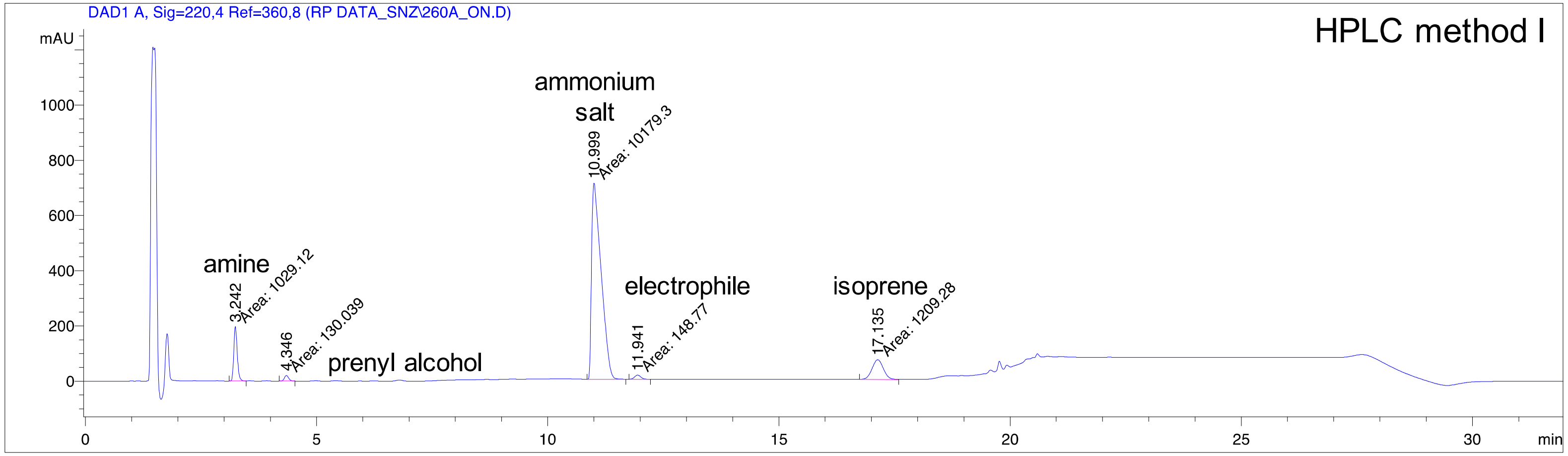

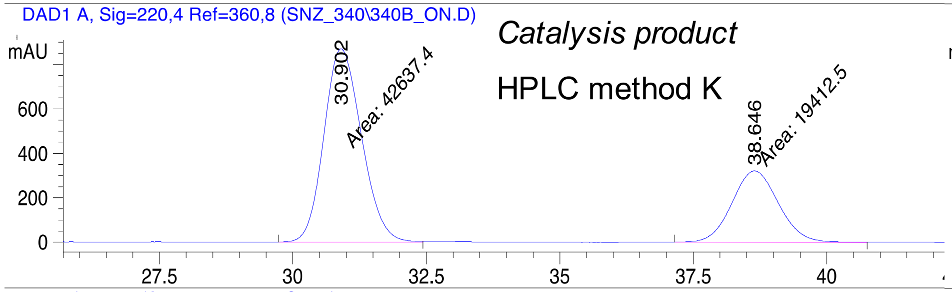

Signal 1: DAD1 A, Sig=220,4 Ref=360,8

Peak #	RetTime [min]	Type	Width [min]	Area [mAU*s]	Height [mAU]	Area %
1	30.902	MM	0.8167	4.26374e4	870.06744	68.7147
2	38.646	MM	1.0087	1.94125e4	320.76135	31.2853

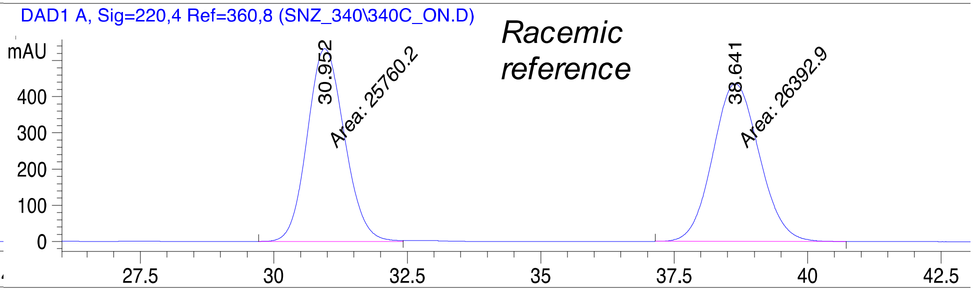

Signal 1: DAD1 A, Sig=220,4 Ref=360,8

Peak #	RetTime [min]	Type	Width [min]	Area [mAU*s]	Height [mAU]	Area %
1	30.952	MM	0.8063	2.57602e4	532.45972	49.3935
2	38.641	MM	1.0094	2.63929e4	435.77673	50.6065

Fig. S56. Reversed phase HPLC trace for the allylation of **1k** with **2a** (top); Chiral phase HPLC trace for the catalysis product **3ka** (bottom left) and for the racemic reference (bottom right). IS = internal standard.

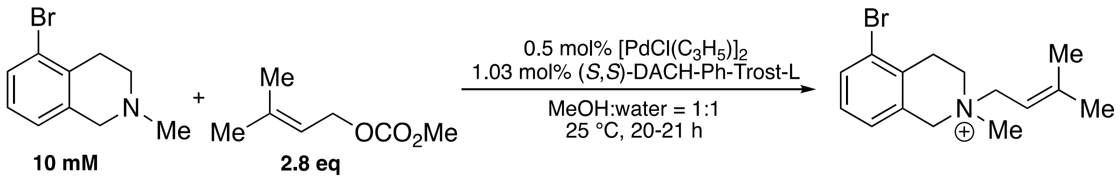

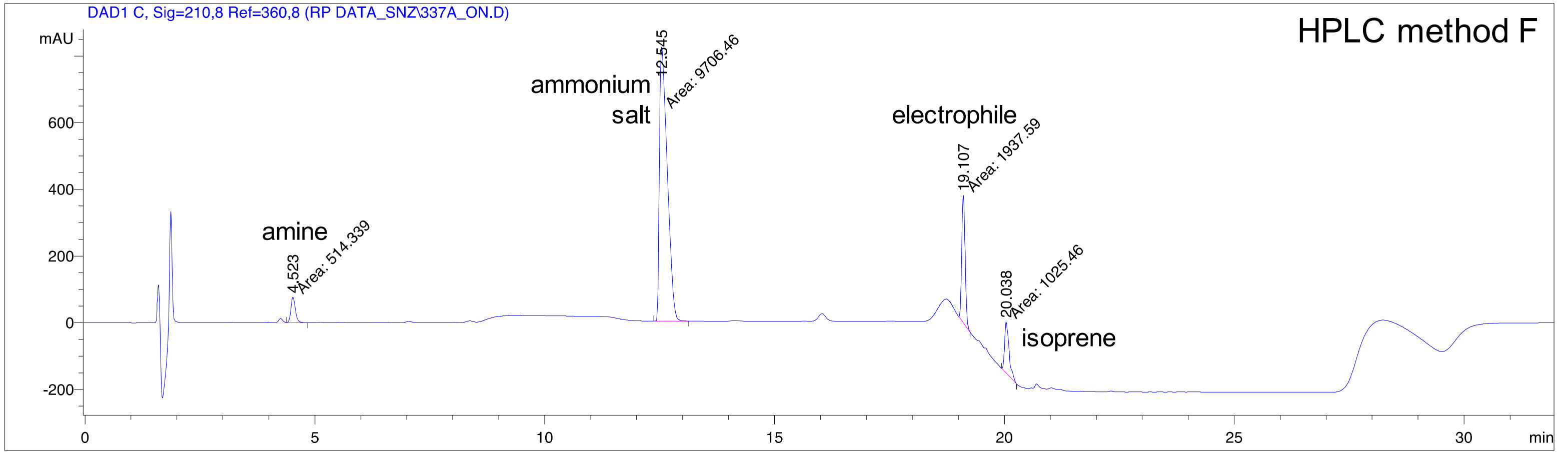

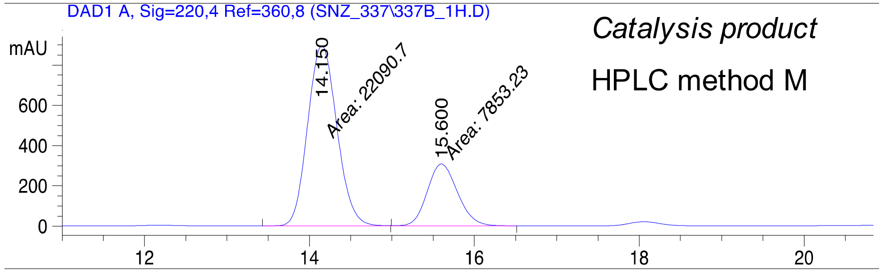

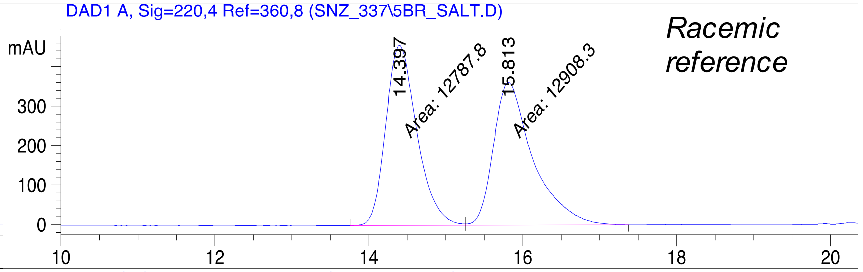

Signal 1: DAD1 A, Sig=220,4 Ref=360,8

Peak #	RetTime [min]	Type	Width [min]	Area [mAU*s]	Height [mAU]	Area %
1	14.150	MM	0.4112	2.20907e4	895.34106	73.7736
2	15.600	MM	0.4259	7853.23340	307.33795	26.2264

Signal 1: DAD1 A, Sig=220,4 Ref=360,8

Peak #	RetTime [min]	Type	Width [min]	Area [mAU*s]	Height [mAU]	Area %
1	14.397	MF	0.4674	1.27878e4	455.98605	49.7656
2	15.813	FM	0.5956	1.29083e4	361.23193	50.2344

Fig. S57. Reversed phase HPLC trace for the allylation of **1f** with **2a** (top); Chiral phase HPLC trace for the catalysis product **3fa** (bottom left) and for the racemic reference (bottom right). IS = internal standard.

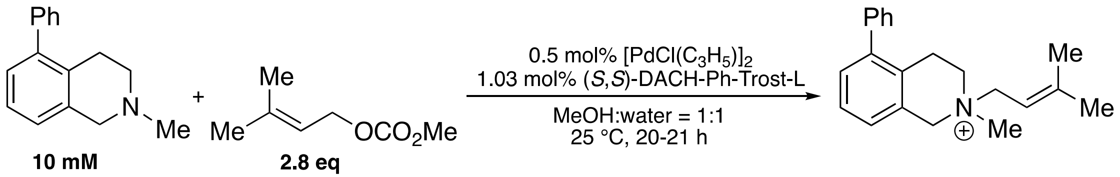

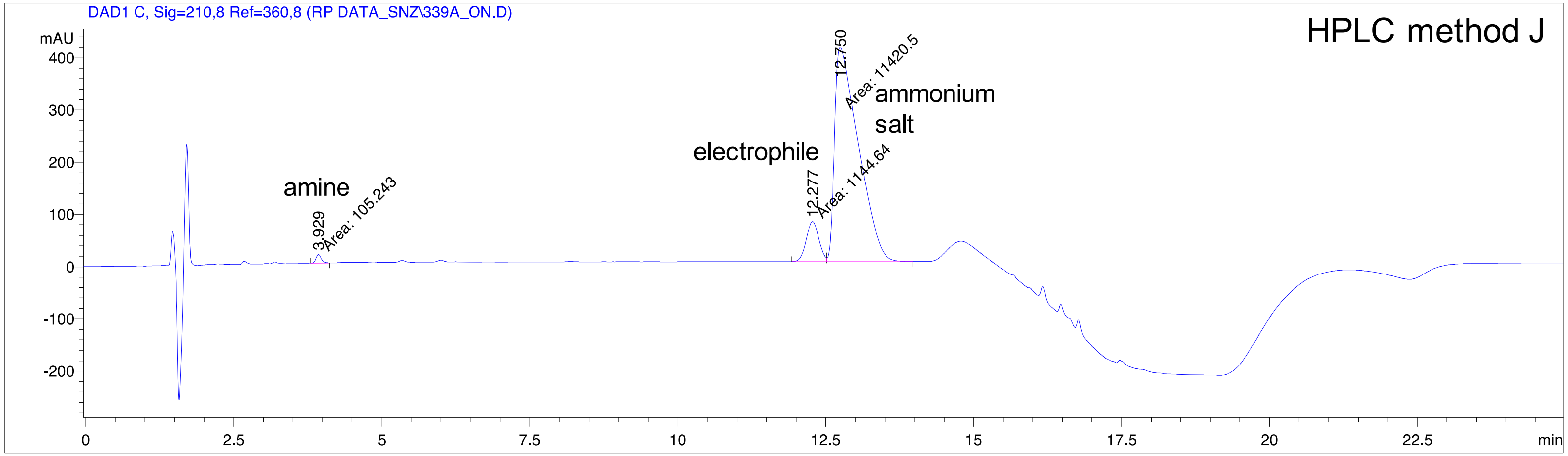

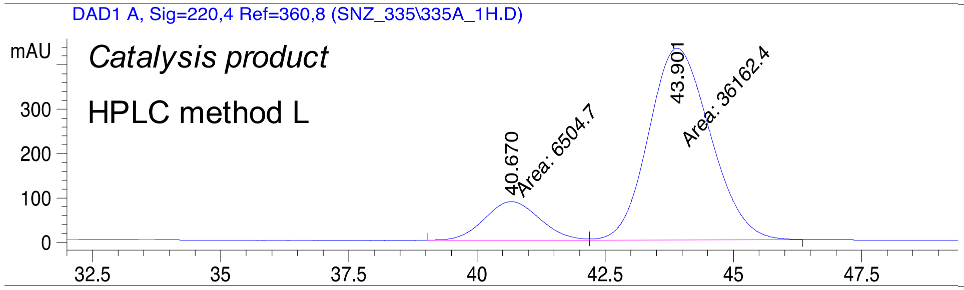

Signal 1: DAD1 A, Sig=220,4 Ref=360,8

Peak #	RetTime [min]	Type	Width [min]	Area [mAU*s]	Height [mAU]	Area %
1	40.670	MF	1.2481	6504.70068	86.85846	15.2452
2	43.901	FM	1.3947	3.61624e4	432.14673	84.7548

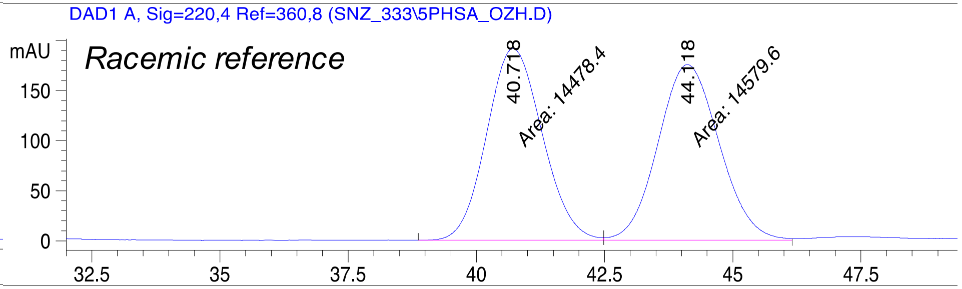

Signal 1: DAD1 A, Sig=220,4 Ref=360,8

Peak #	RetTime [min]	Type	Width [min]	Area [mAU*s]	Height [mAU]	Area %
1	40.718	MM	1.2588	1.44784e4	191.70007	49.8260
2	44.118	MM	1.3842	1.45796e4	175.54384	50.1740

Fig. S58. Reversed phase HPLC trace for the allylation of **1l** with **2a** (top); Chiral phase HPLC trace for the catalysis product **3la** (bottom left) and for the racemic reference (bottom right). IS = internal standard.

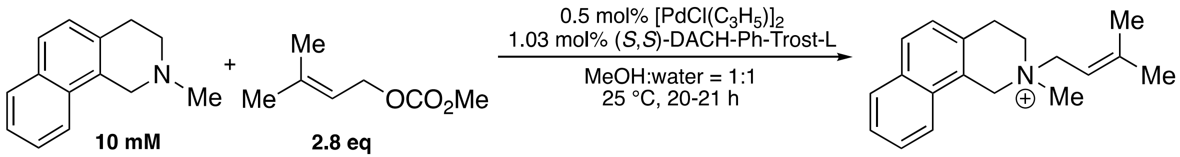

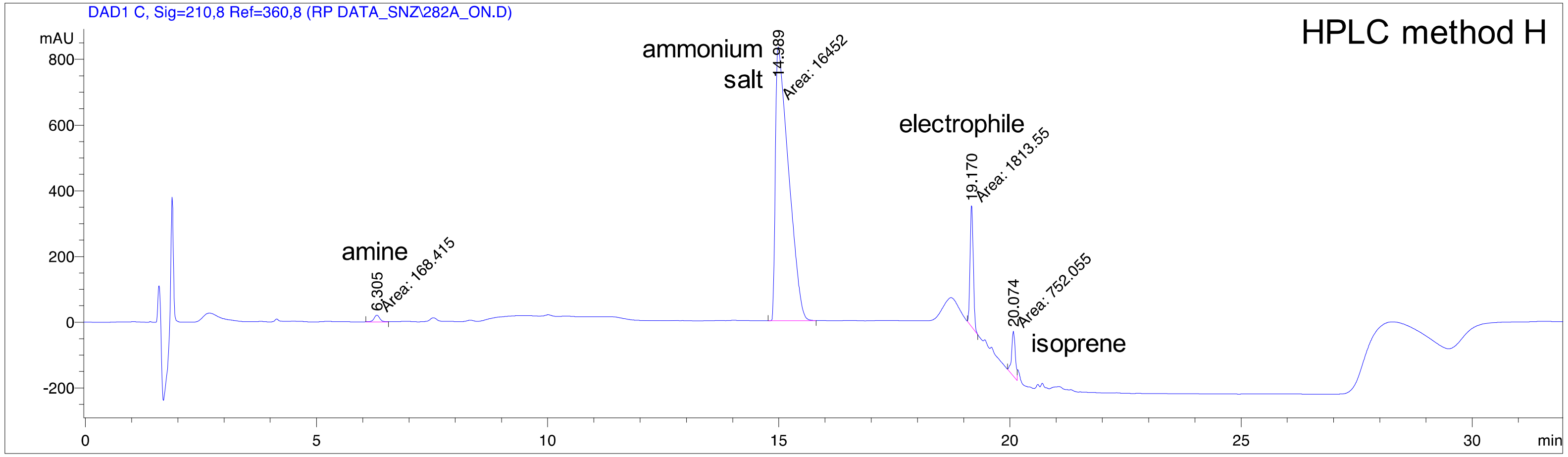

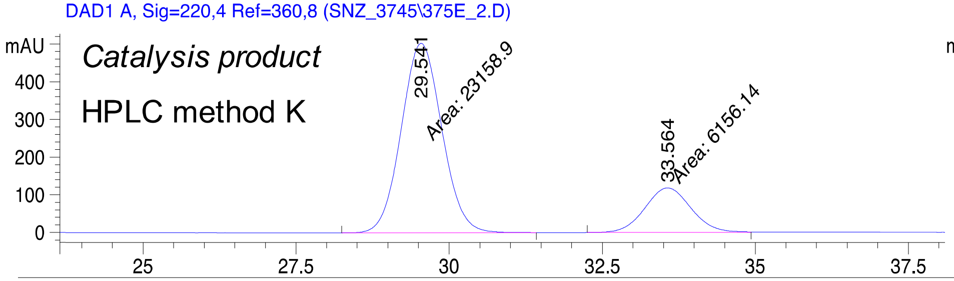

Signal 1: DAD1 A, Sig=220,4 Ref=360,8

Peak #	RetTime [min]	Type	Width [min]	Area [mAU*s]	Height [mAU]	Area %
1	29.541	MM	0.7687	2.31589e4	502.13416	79.0001
2	33.564	MM	0.8709	6156.13770	117.81519	20.9999

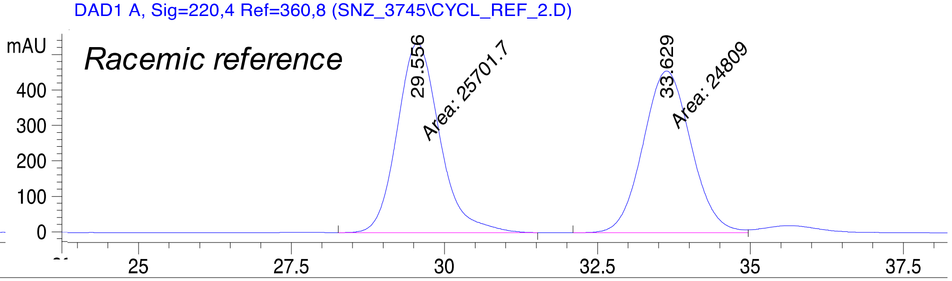

Signal 1: DAD1 A, Sig=220,4 Ref=360,8

Peak #	RetTime [min]	Type	Width [min]	Area [mAU*s]	Height [mAU]	Area %
1	29.556	MM	0.8045	2.57017e4	532.48602	50.8837
2	33.629	MM	0.9096	2.48090e4	454.57141	49.1163

Fig. S59. Reversed phase HPLC trace for the allylation of **1d** with **2a** (top); Chiral phase HPLC trace for the catalysis product **3da** (bottom left) and for the racemic reference (bottom right). IS = internal standard.

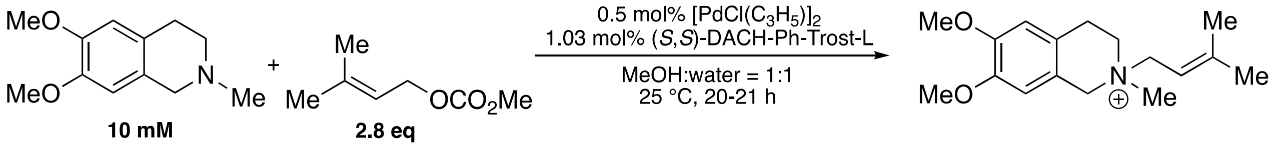

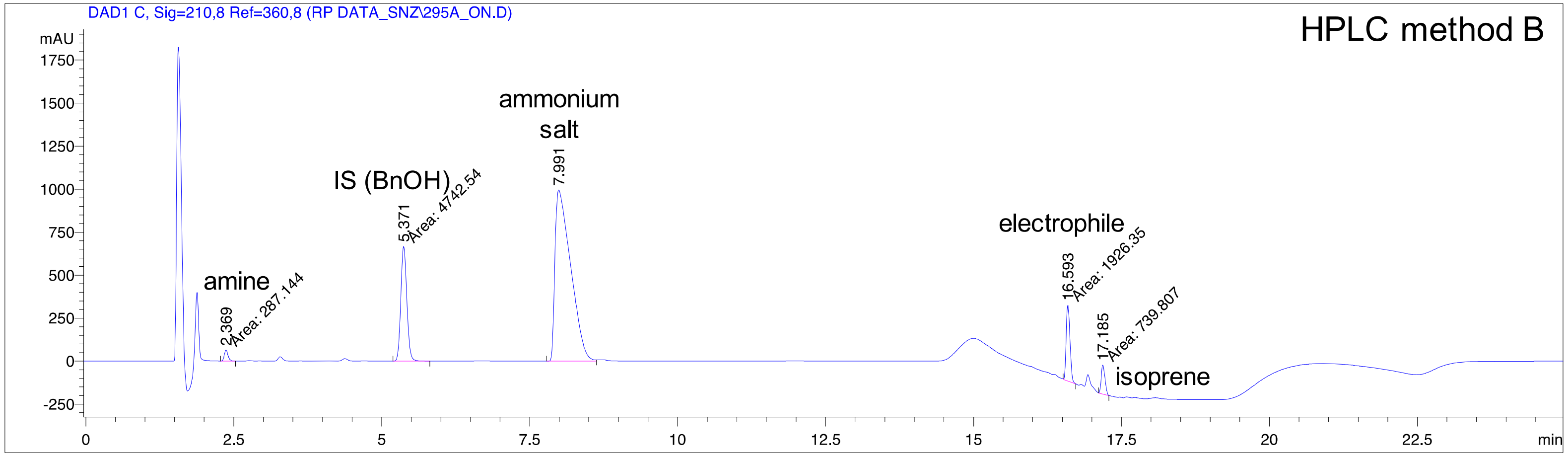

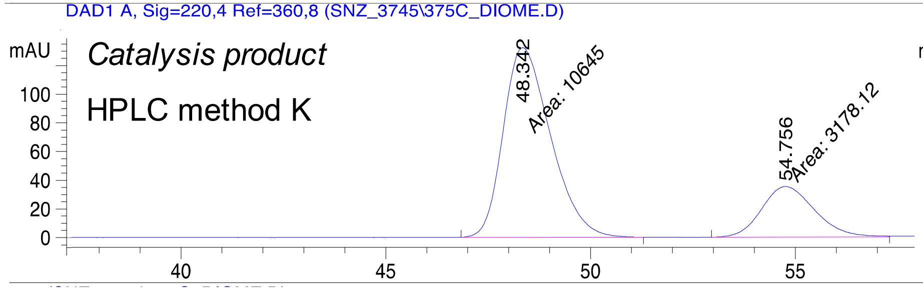

Signal 1: DAD1 A, Sig=220,4 Ref=360,8

Peak #	RetTime [min]	Type	Width [min]	Area [mAU*s]	Height [mAU]	Area %
1	48.342	MM	1.3400	1.06450e4	132.39893	77.0087
2	54.756	MM	1.5027	3178.11792	35.24971	22.9913

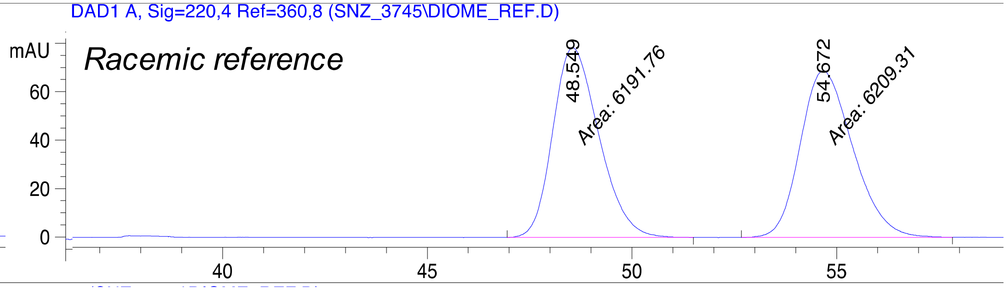

Signal 1: DAD1 A, Sig=220,4 Ref=360,8

Peak #	RetTime [min]	Type	Width [min]	Area [mAU*s]	Height [mAU]	Area %
1	48.549	MM	1.3059	6191.76123	79.02053	49.9292
2	54.672	MM	1.4944	6209.30957	69.25105	50.0708

Fig. S60. Reversed phase HPLC trace for the allylation of **1g** with **2a** (top); Chiral phase HPLC trace for the catalysis product **3ga** (bottom left) and for the racemic reference (bottom right). IS = internal standard.

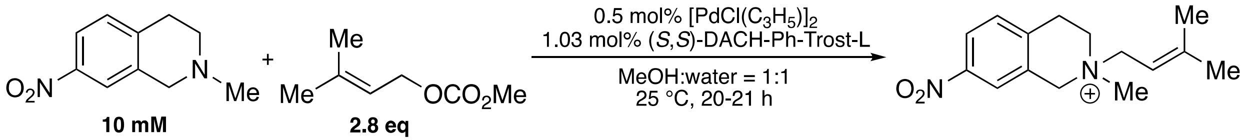

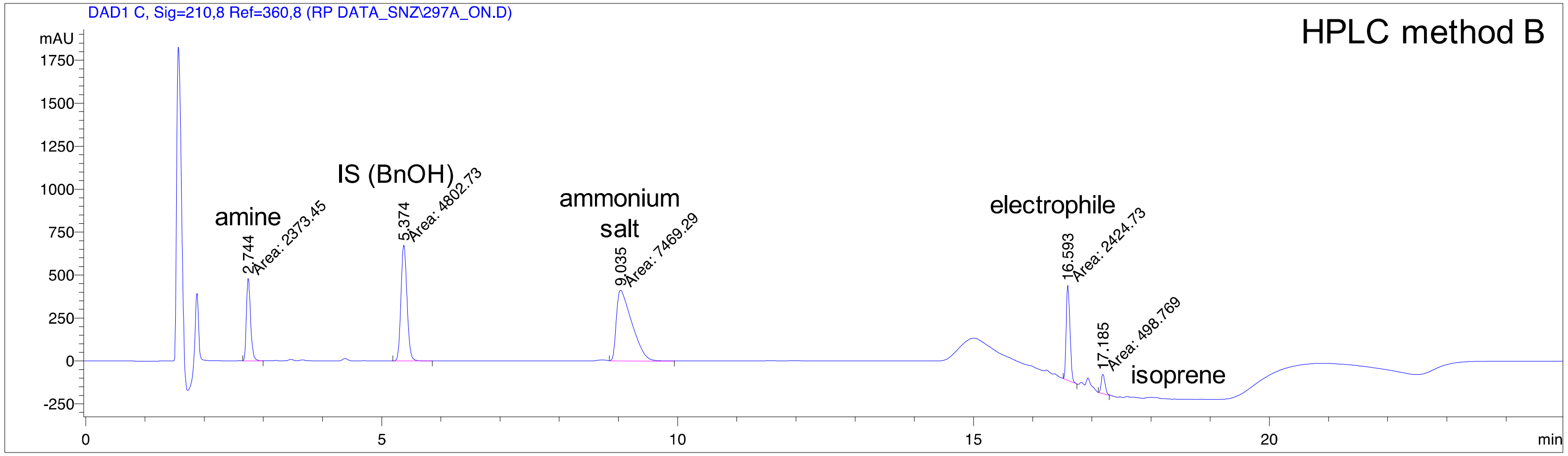

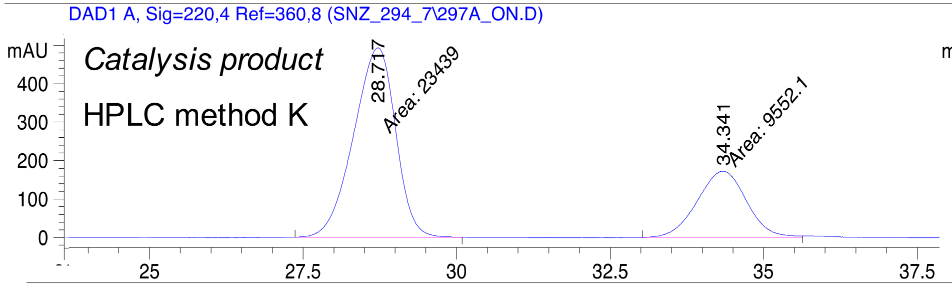

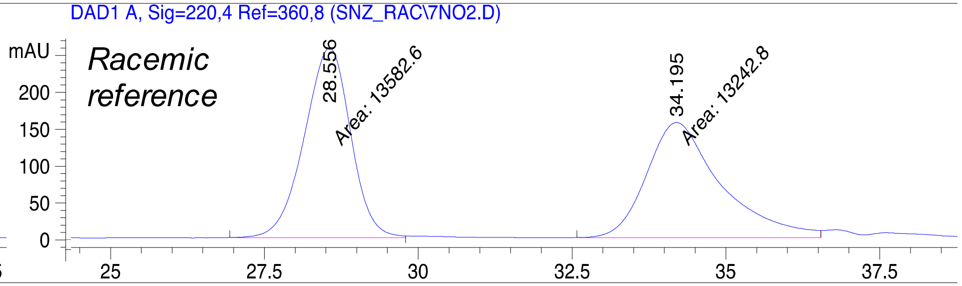

Signal 1: DAD1 A, Sig=220,4 Ref=360,8

Peak #	RetTime [min]	Type	Width [min]	Area [mAU*s]	Height [mAU]	Area %
1	28.717	MM	0.7924	2.34390e4	492.98306	71.0464
2	34.341	MM	0.9271	9552.10156	171.72131	28.9536

Signal 1: DAD1 A, Sig=220,4 Ref=360,8

Peak #	RetTime [min]	Type	Width [min]	Area [mAU*s]	Height [mAU]	Area %
1	28.556	MM	0.8720	1.35826e4	259.59985	50.6332
2	34.195	MM	1.4020	1.32428e4	157.42807	49.3668

Fig. S61. Reversed phase HPLC trace for the allylation of **1h** with **2a** (top); Chiral phase HPLC trace for the catalysis product **3ha** (bottom left) and for the racemic reference (bottom right). IS = internal standard.

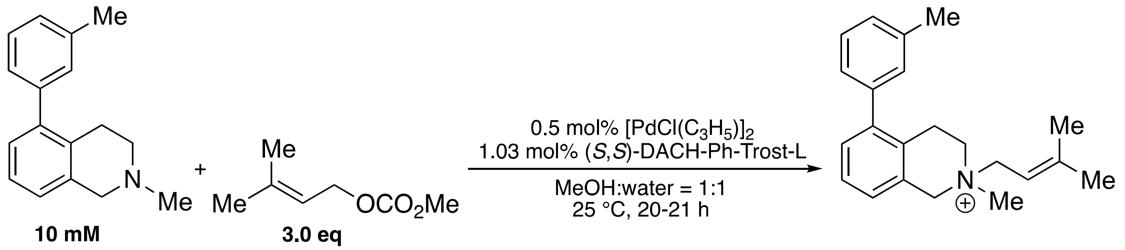

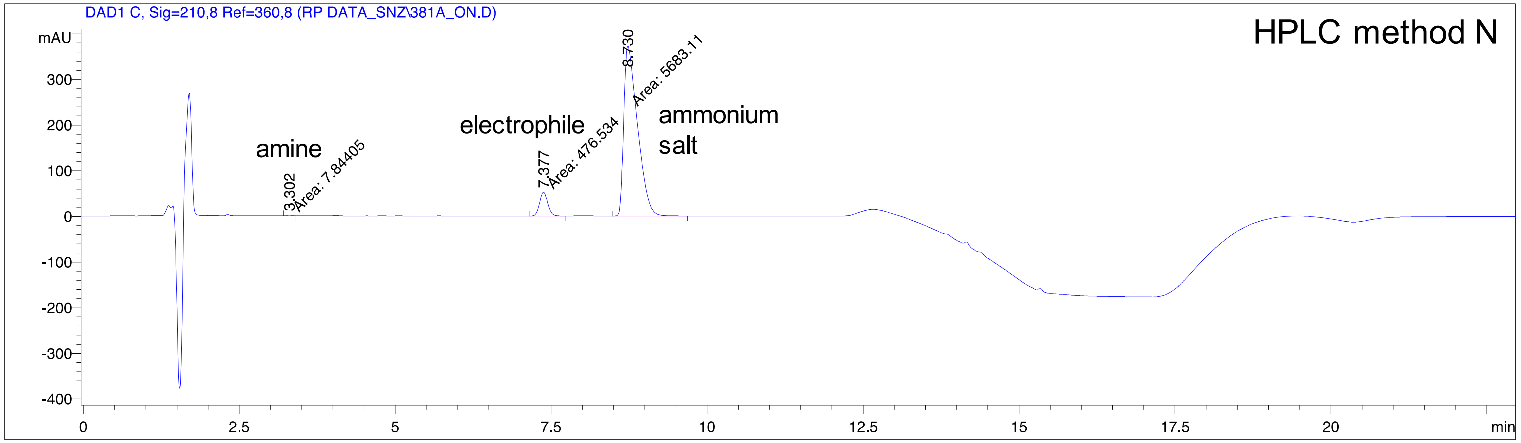

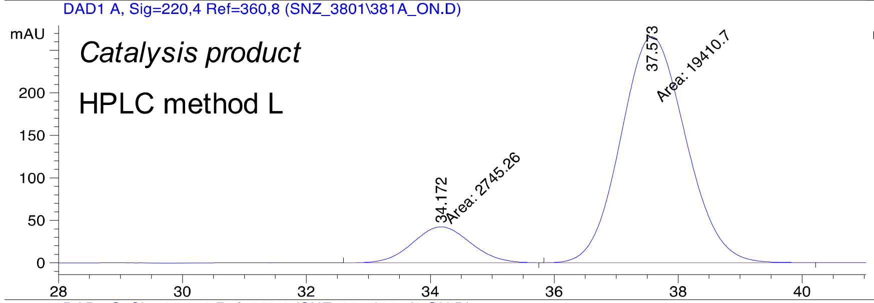

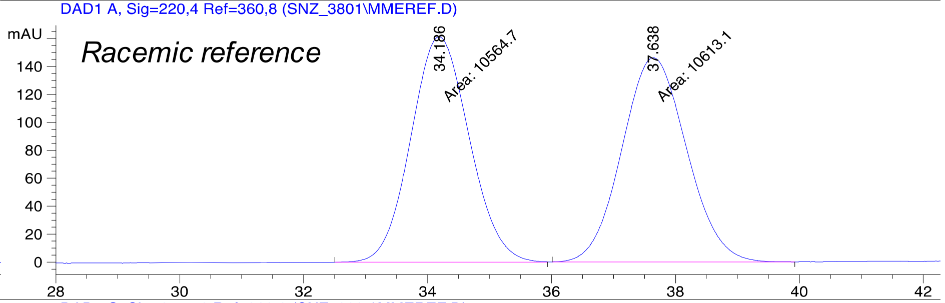

Signal 1: DAD1 A, Sig=220,4 Ref=360,8

Peak #	RetTime [min]	Type	Width [min]	Area [mAU*s]	Height [mAU]	Area %
1	34.172	MM	1.0832	2745.26172	42.24133	12.3906
2	37.573	MM	1.2164	1.94107e4	265.95978	87.6094

Signal 1: DAD1 A, Sig=220,4 Ref=360,8

Peak #	RetTime [min]	Type	Width [min]	Area [mAU*s]	Height [mAU]	Area %
1	34.186	MM	1.0914	1.05647e4	161.33258	49.8855
2	37.638	MM	1.2107	1.06131e4	146.10417	50.1145

Fig. S62. Reversed phase HPLC trace for the allylation of **1m** with **2a** (top); Chiral phase HPLC trace for the catalysis product **3ma** (bottom left) and for the racemic reference (bottom right). IS = internal standard.

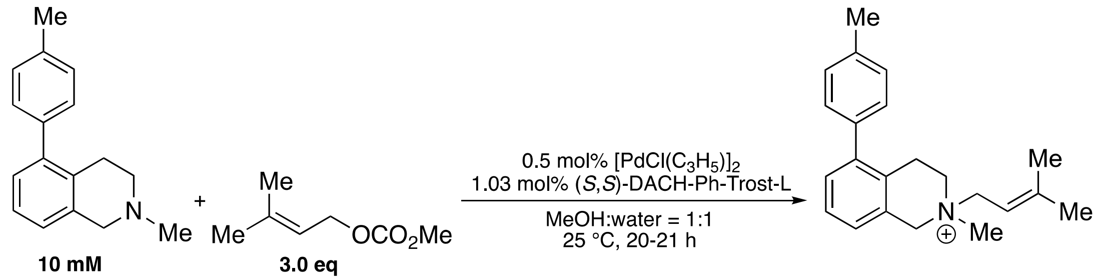

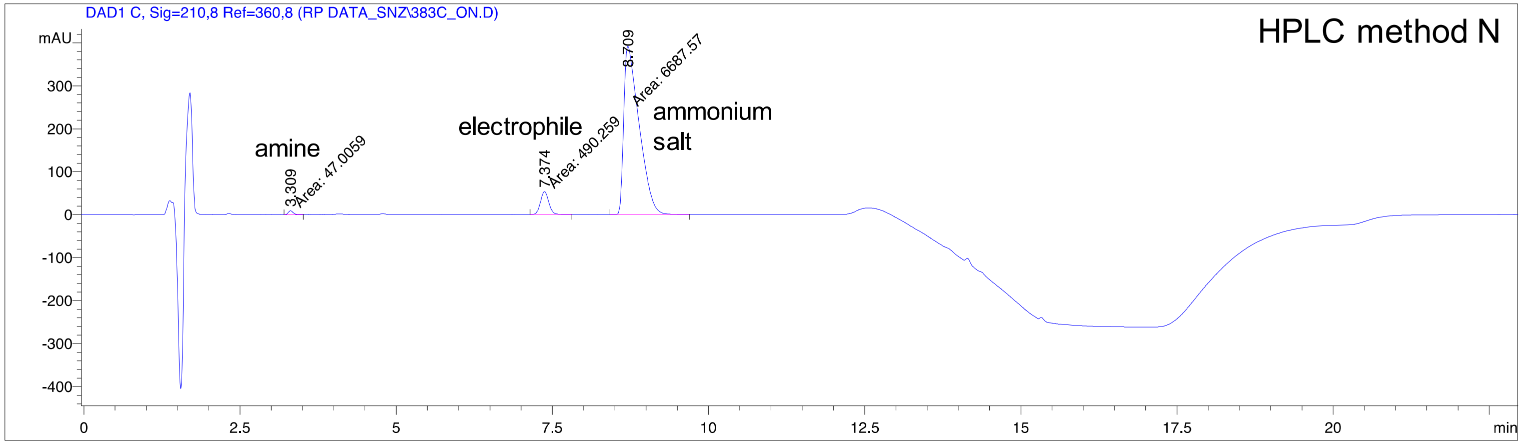

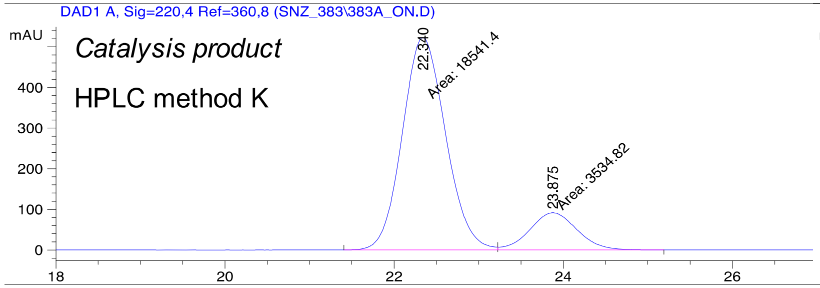

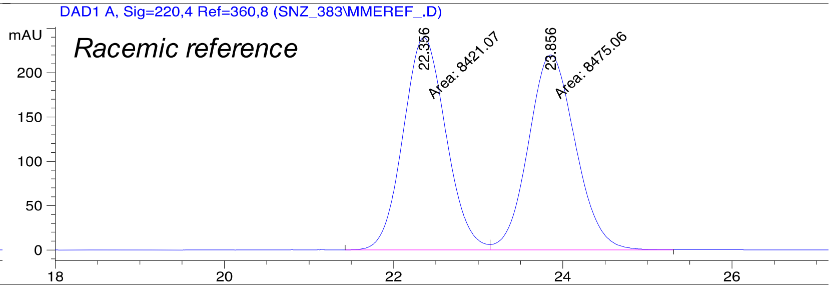

Signal 1: DAD1 A, Sig=220,4 Ref=360,8

Peak #	RetTime [min]	Type	Width [min]	Area [mAU*s]	Height [mAU]	Area %
1	22.340	MF	0.5914	1.85414e4	522.52252	83.9881
2	23.875	FM	0.6461	3534.81641	91.18279	16.0119

Signal 1: DAD1 A, Sig=220,4 Ref=360,8

Peak #	RetTime [min]	Type	Width [min]	Area [mAU*s]	Height [mAU]	Area %
1	22.356	MF	0.5865	8421.06738	239.31079	49.8402
2	23.856	FM	0.6426	8475.06250	219.81804	50.1598

Fig. S63. Reversed phase HPLC trace for the allylation of **1n** with **2a** (top); Chiral phase HPLC trace for the catalysis product **3na** (bottom left) and for the racemic reference (bottom right). IS = internal standard.

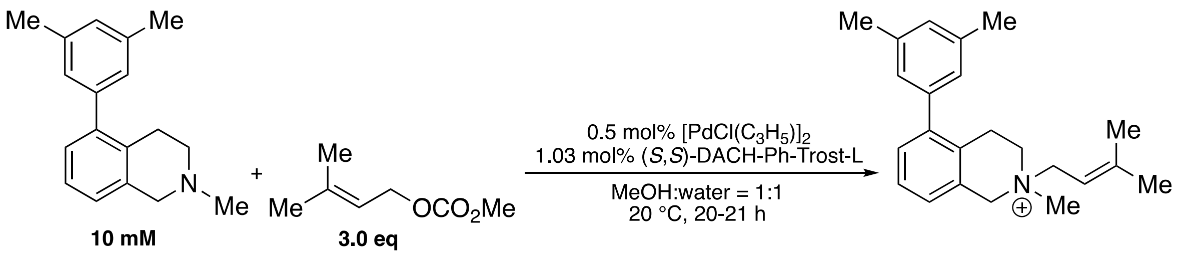

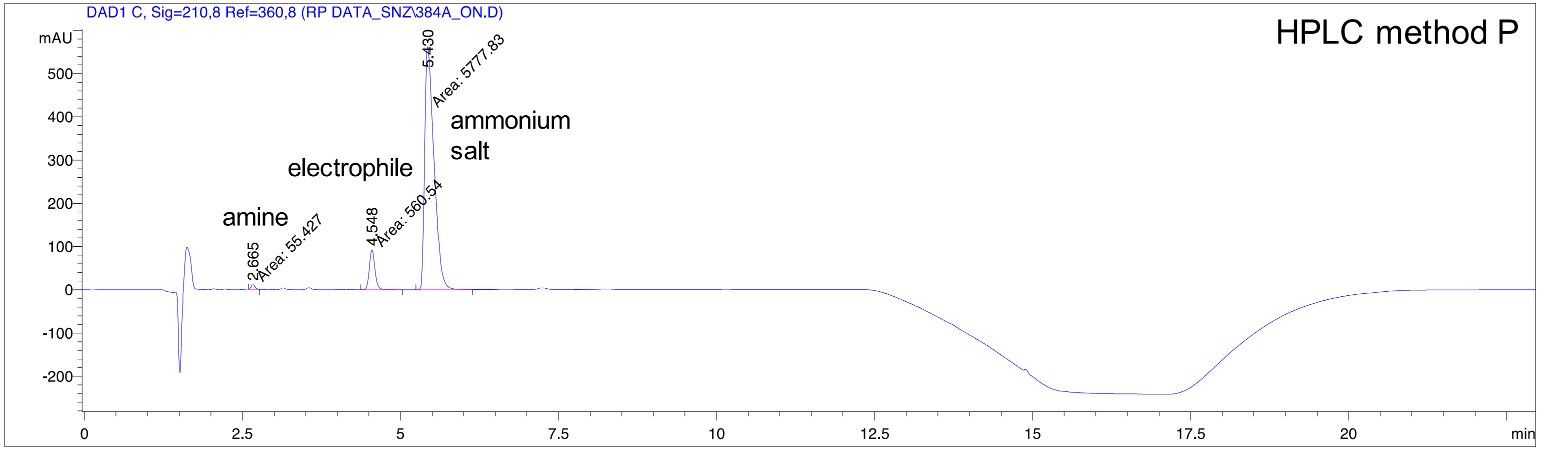

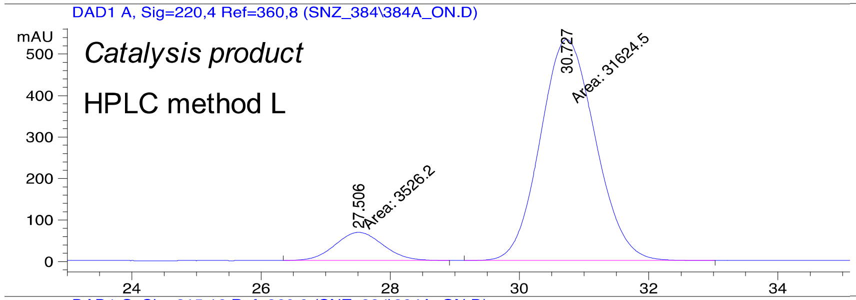

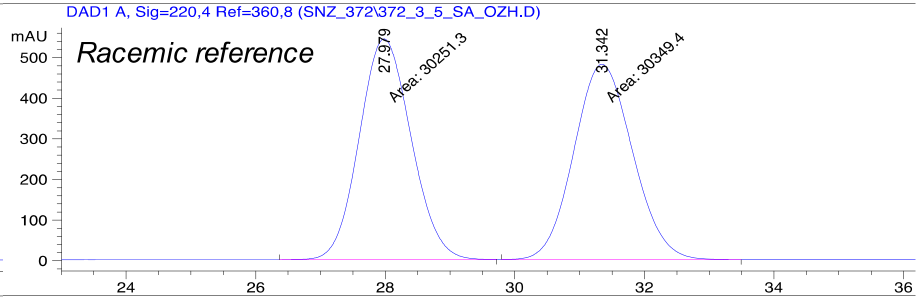

Signal 1: DAD1 A, Sig=220,4 Ref=360,8

Peak #	RetTime [min]	Type	Width [min]	Area [mAU*s]	Height [mAU]	Area %
1	27.506	MM	0.8645	3526.19922	67.98243	10.0317
2	30.727	MM	0.9890	3.16245e4	532.91583	89.9683

Signal 1: DAD1 A, Sig=220,4 Ref=360,8

Peak #	RetTime [min]	Type	Width [min]	Area [mAU*s]	Height [mAU]	Area %
1	27.979	MM	0.9279	3.02513e4	543.38654	49.9190
2	31.342	MM	1.0518	3.03494e4	480.90701	50.0810

Fig. S64. Reversed phase HPLC trace for the allylation of **1o** with **2a** (top); Chiral phase HPLC trace for the catalysis product **3oa** (bottom left) and for the racemic reference (bottom right). IS = internal standard.

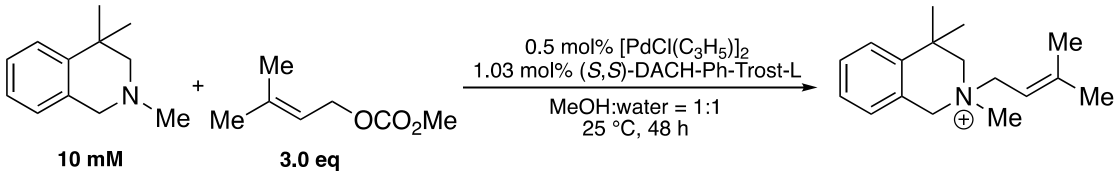

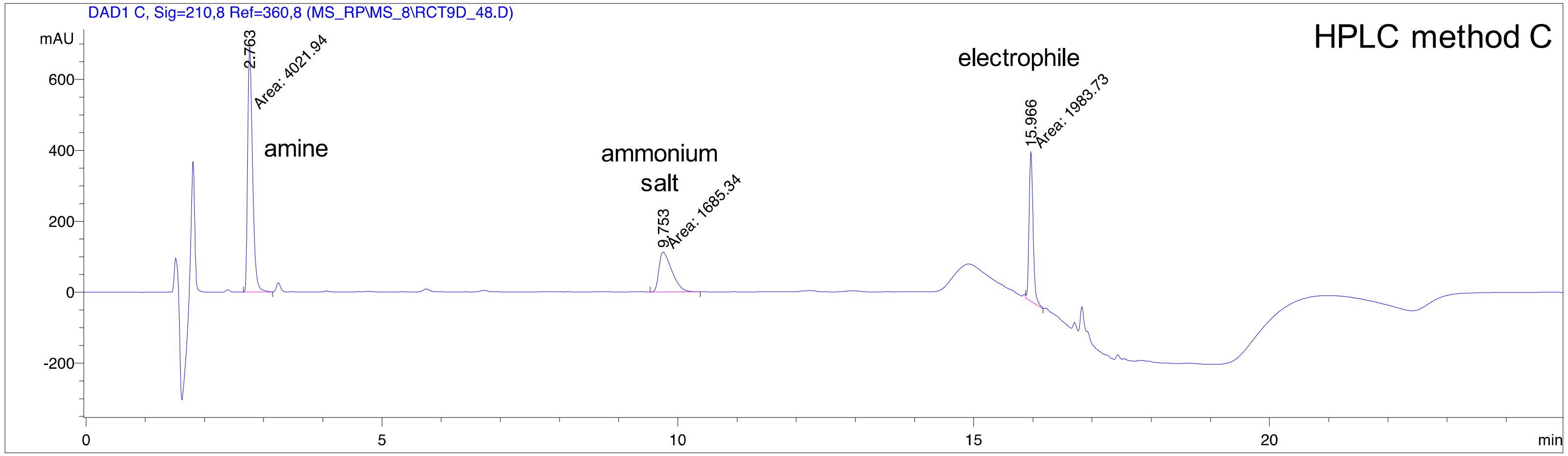

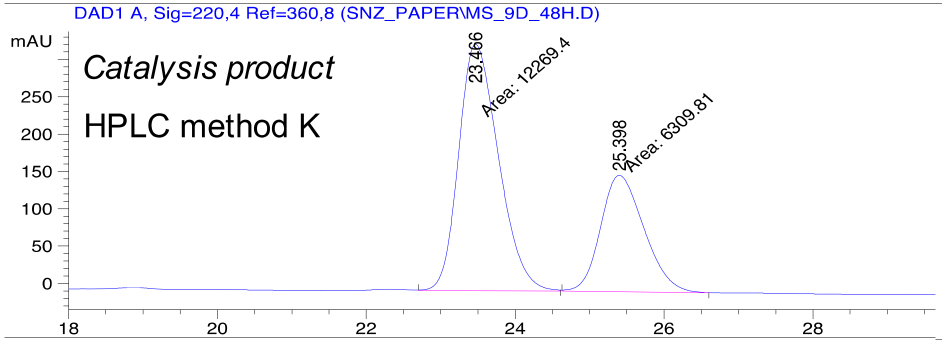

Signal 1: DAD1 A, Sig=220,4 Ref=360,8

Peak #	RetTime [min]	Type	Width [min]	Area [mAU*s]	Height [mAU]	Area %
1	23.466	MM	0.6197	1.22694e4	329.95529	66.0383
2	25.398	MM	0.6750	6309.80518	155.80167	33.9617

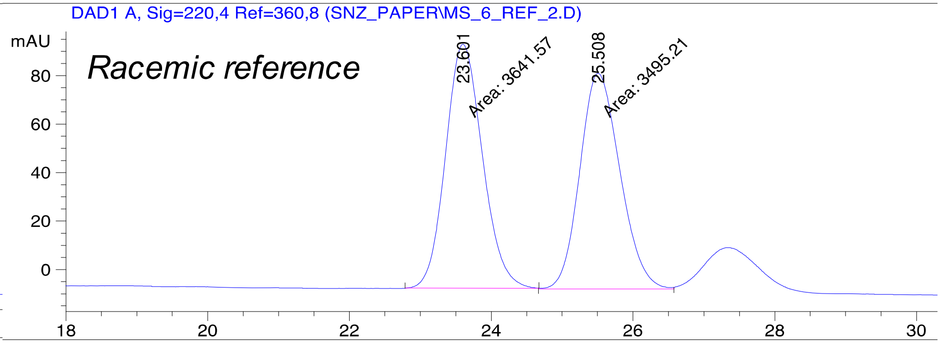

Signal 1: DAD1 A, Sig=220,4 Ref=360,8

Peak #	RetTime [min]	Type	Width [min]	Area [mAU*s]	Height [mAU]	Area %
1	23.601	MM	0.6033	3641.56665	100.59865	51.0254
2	25.508	MM	0.6551	3495.20557	88.92300	48.9746

Fig. S65. Reversed phase HPLC trace for the allylation of **1i** with **2a** (top); Chiral phase HPLC trace for the catalysis product **3ia** (bottom left) and for the racemic reference (bottom right). IS = internal standard.

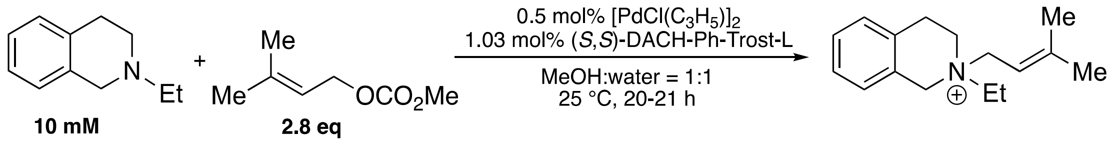

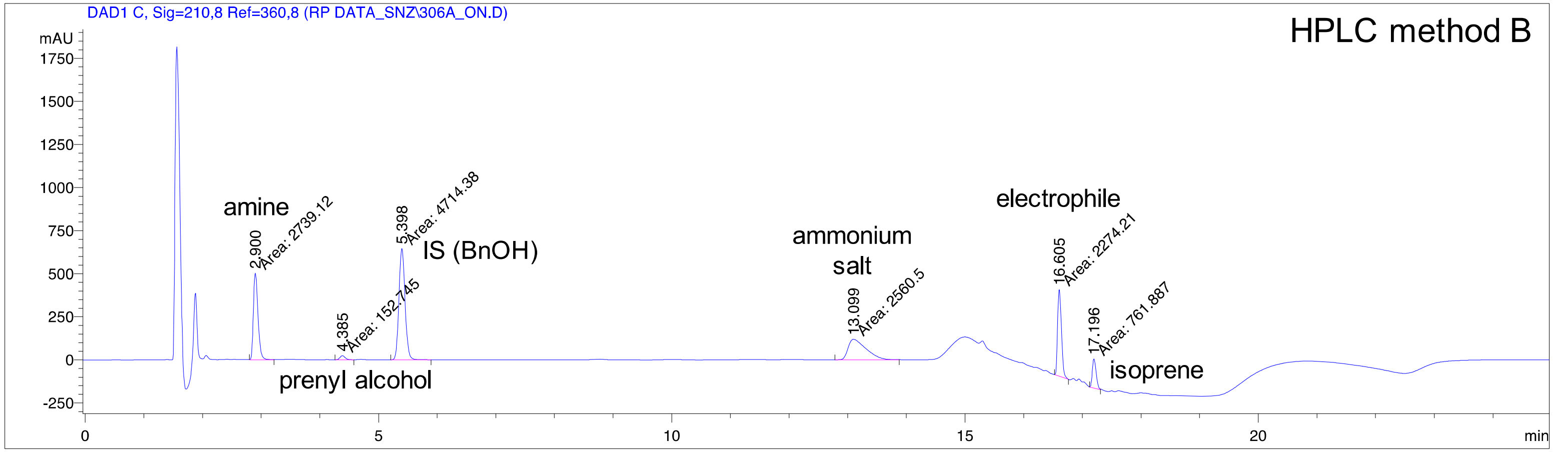

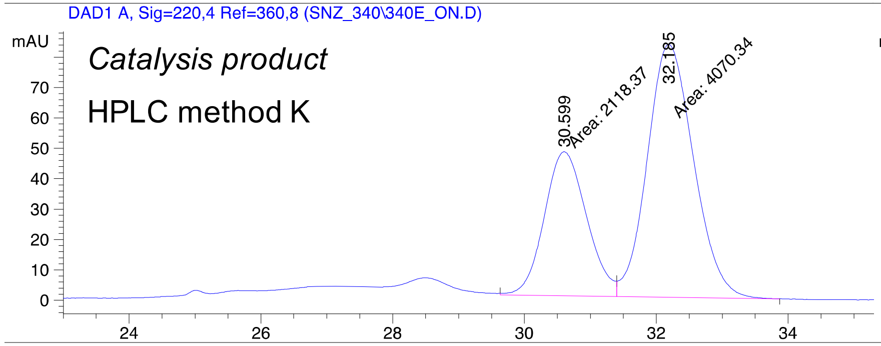

Signal 1: DAD1 A, Sig=220,4 Ref=360,8

Peak #	RetTime [min]	Type	Width [min]	Area [mAU*s]	Height [mAU]	Area %
1	30.599	MF	0.7455	2118.37280	47.36028	34.2296
2	32.185	FM	0.8152	4070.33789	83.21916	65.7704

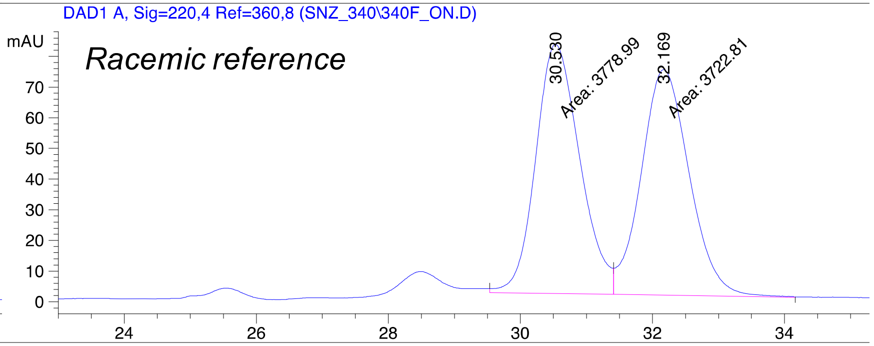

Signal 1: DAD1 A, Sig=220,4 Ref=360,8

Peak #	RetTime [min]	Type	Width [min]	Area [mAU*s]	Height [mAU]	Area %
1	30.530	MF	0.7752	3778.98706	81.24844	50.3744
2	32.169	FM	0.8437	3722.80640	73.54079	49.6256

Fig. S66. Reversed phase HPLC trace for the allylation of **1r** with **2a** (top); Chiral phase HPLC trace for the catalysis product **3ra** (bottom left) and for the racemic reference (bottom right). IS = internal standard.

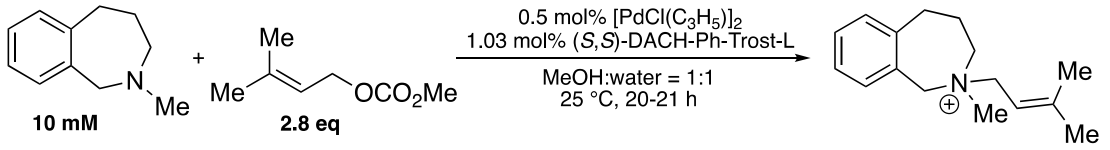

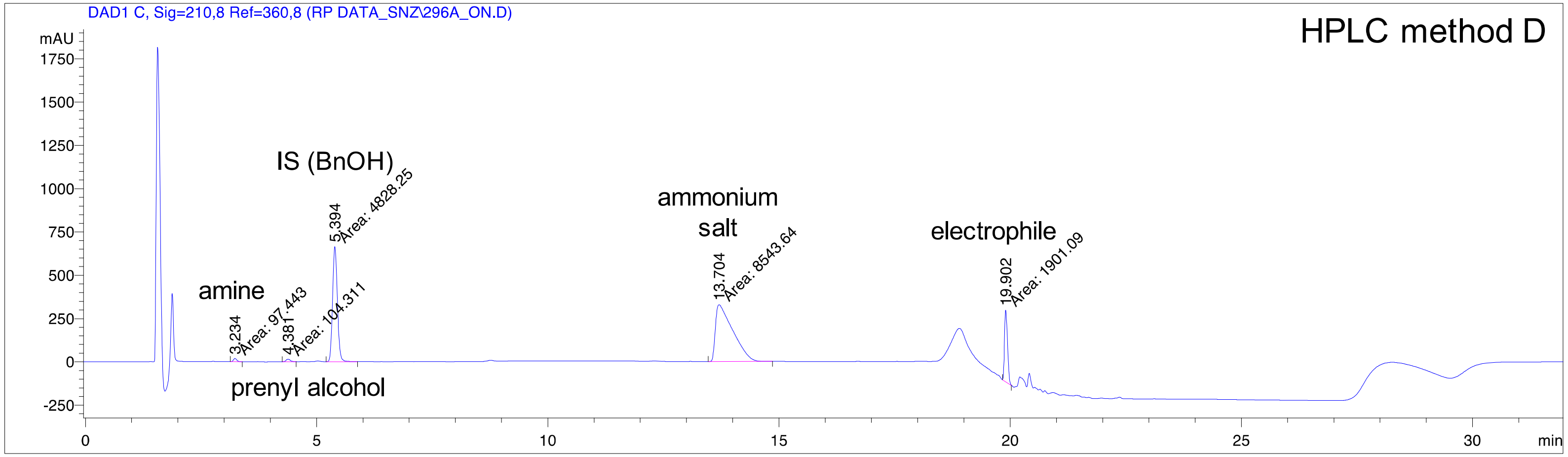

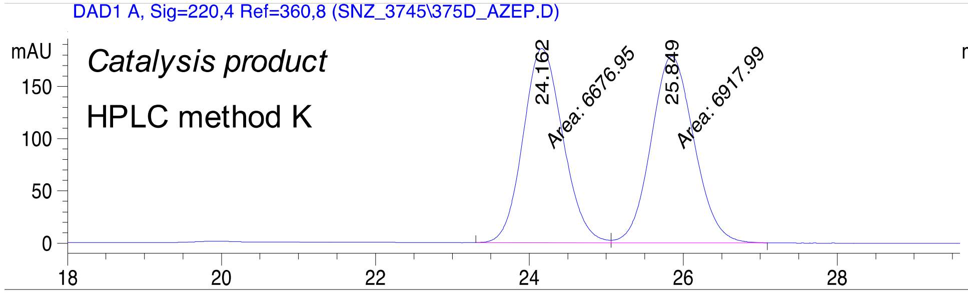

Signal 1: DAD1 A, Sig=220,4 Ref=360,8

Peak #	RetTime [min]	Type	Width [min]	Area [mAU*s]	Height [mAU]	Area %
1	24.162	MF	0.5986	6676.95068	185.89854	49.1135
2	25.849	FM	0.6458	6917.98926	178.53246	50.8865

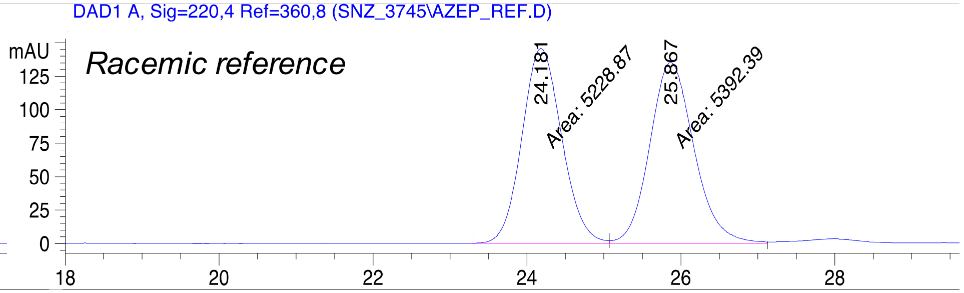

Signal 1: DAD1 A, Sig=220,4 Ref=360,8

Peak #	RetTime [min]	Type	Width [min]	Area [mAU*s]	Height [mAU]	Area %
1	24.181	MF	0.5987	5228.86572	145.56253	49.2302
2	25.867	FM	0.6654	5392.38916	135.07541	50.7698

Fig. S67. Reversed phase HPLC trace for the allylation of **1j** with **2a** (top); Chiral phase HPLC trace for the catalysis product **3ja** (bottom left) and for the racemic reference (bottom right). IS = internal standard.

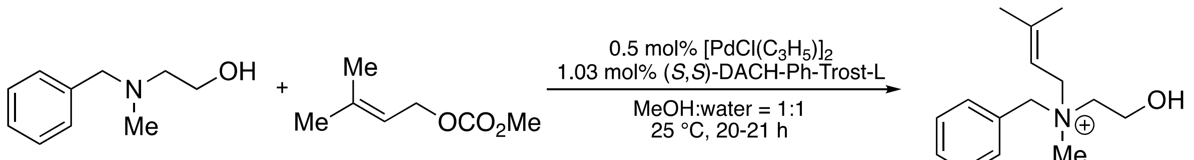

10 mM **2.8 eq**

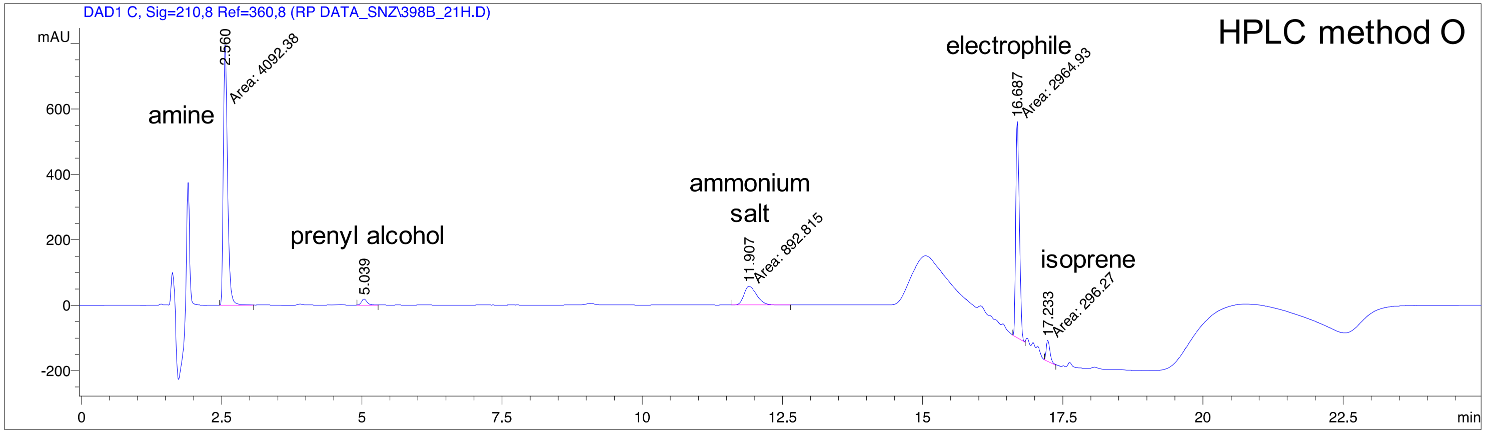

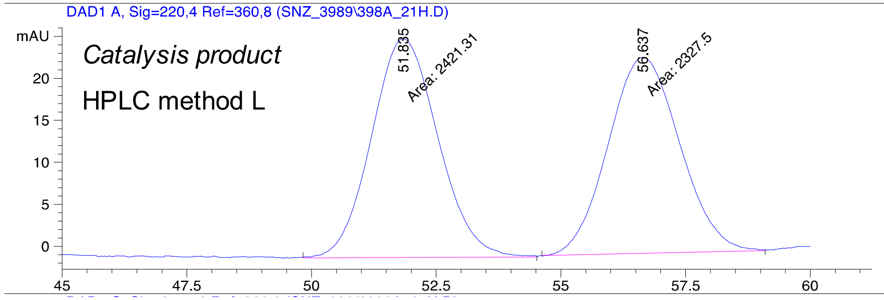

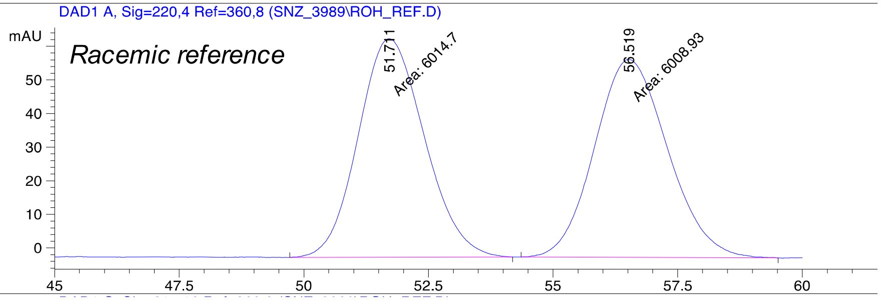

Signal 1: DAD1 A, Sig=220,4 Ref=360,8

Peak #	RetTime [min]	Type	Width [min]	Area [mAU*s]	Height [mAU]	Area %
1	51.835	MM	1.5491	2424.40308	26.08345	50.8789
2	56.637	MM	1.6659	2340.64355	23.41654	49.1211

Signal 1: DAD1 A, Sig=220,4 Ref=360,8

Peak #	RetTime [min]	Type	Width [min]	Area [mAU*s]	Height [mAU]	Area %
1	51.711	MM	1.5422	6024.87988	65.11181	50.0129
2	56.519	MM	1.7019	6021.77441	58.97070	49.9871

Fig. S68. Reversed phase HPLC trace for the allylation of **1p** with **2a** (top); Chiral phase HPLC trace for the catalysis product **3pa** (bottom left) and for the racemic reference (bottom right). IS = internal standard.

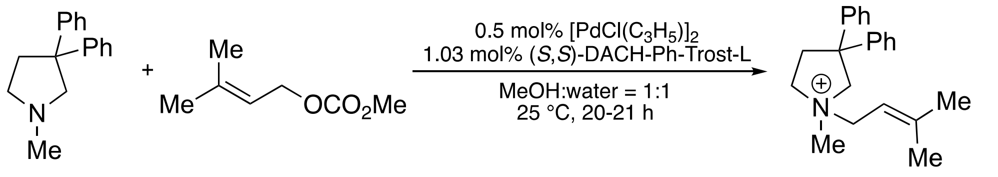

10 mM **2.8 eq**

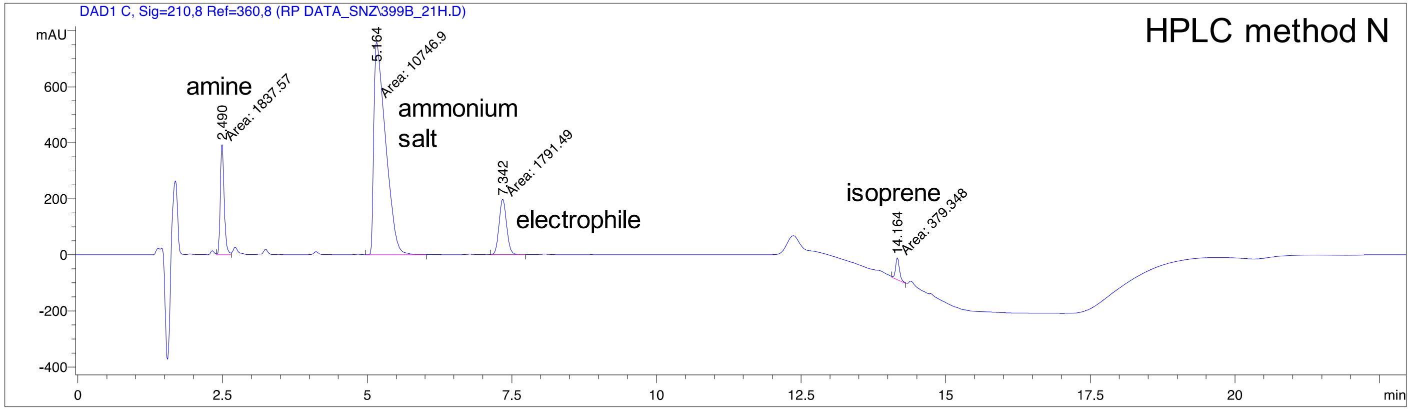

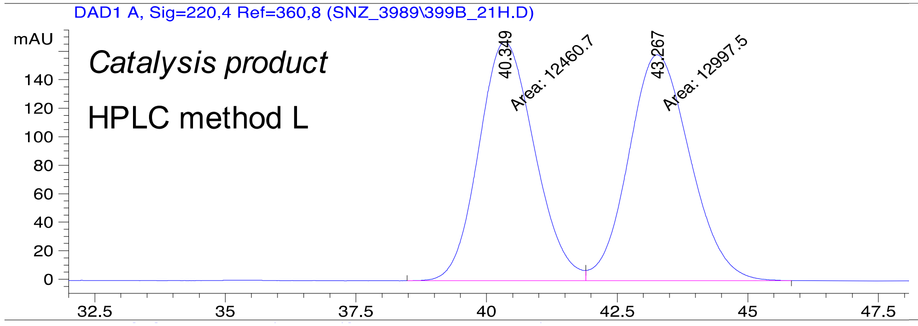

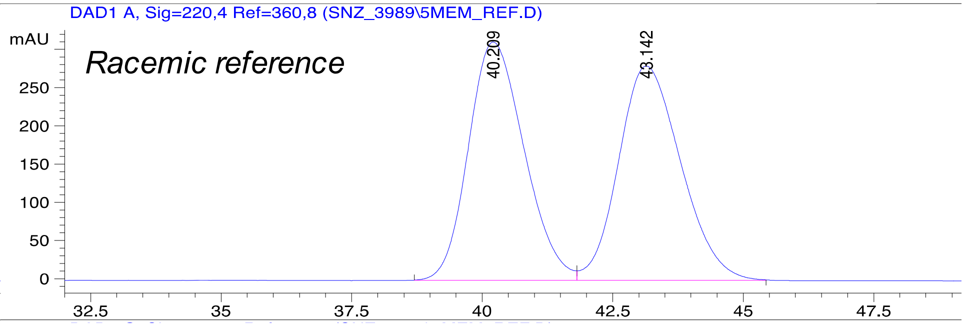

Signal 1: DAD1 A, Sig=220,4 Ref=360,8

Peak #	RetTime [min]	Type	Width [min]	Area [mAU*s]	Height [mAU]	Area %
1	40.349	MM	1.2384	1.24607e4	167.70343	48.9458
2	43.267	MM	1.3655	1.29975e4	158.63593	51.0542

Signal 1: DAD1 A, Sig=220,4 Ref=360,8

Peak #	RetTime [min]	Type	Width [min]	Area [mAU*s]	Height [mAU]	Area %
1	40.209	BV	1.1403	2.31522e4	311.78299	49.9061
2	43.142	VB	1.2548	2.32393e4	280.42413	50.0939

Fig. S69. Reversed phase HPLC trace for the allylation of **1q** with **2a** (top); Chiral phase HPLC trace for the catalysis product **3qa** (bottom left) and for the racemic reference (bottom right). IS = internal standard.

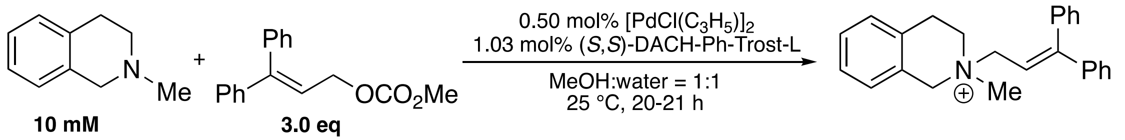

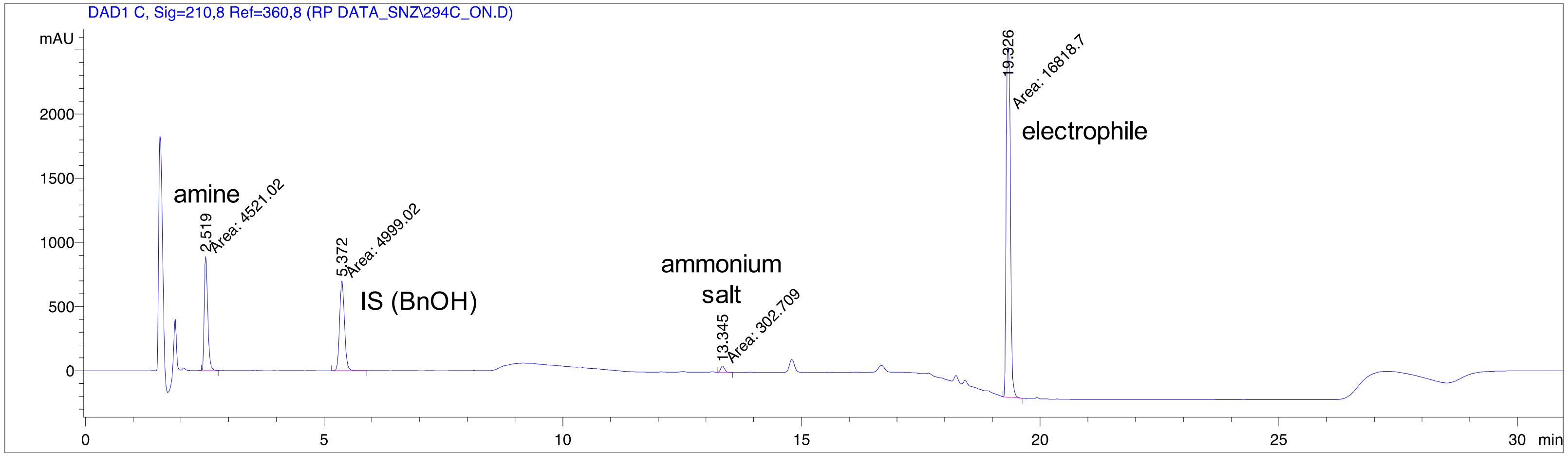

Fig. S70. RP HPLC trace for the allylation of **1a** with **2d.**

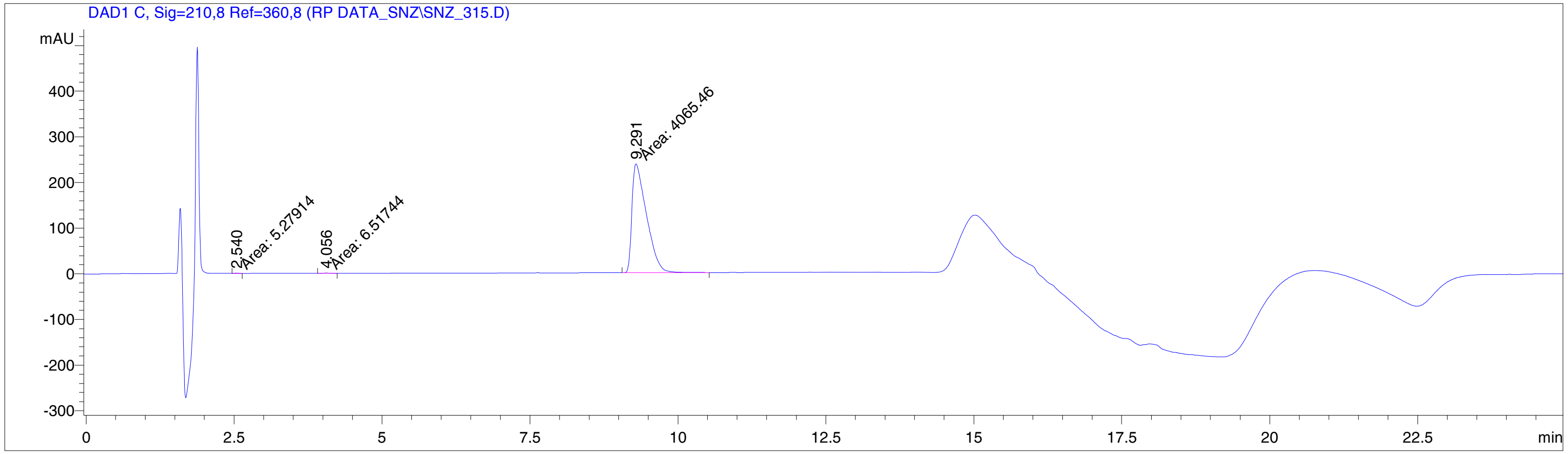

Fig. S71. RP HPLC trace for the *2-methyl-2-(3-methylbut-2-en-1-yl)-1,2,3,4-tetrahydroisoquinolin-2-ium acetate*, isolated from a preparative reaction on a 0.26 mmol scale.

allyl ammonium salt 3aa integrated at different wavelengths in chiral phase HPLC analysis

To check for underlying impurities, an HPLChromatogram of ammonium ion **3aa** (determined *er* = 77:23 at 220 nm) was integrated at various wavelengths. 220 nm (4 nm bandwidth) was chosen as the wavelength for *er* determination.

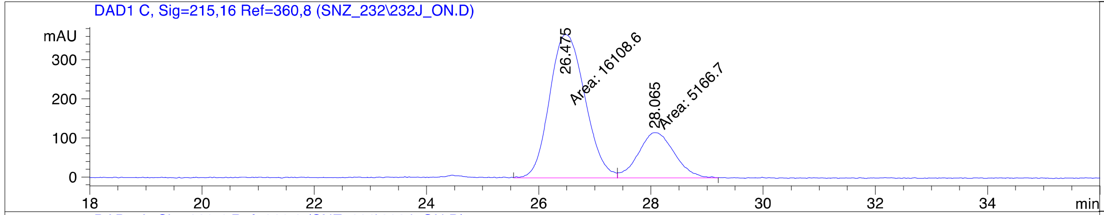

Signal 1: DAD1 C, Sig=215,16 Ref=360,8

Peak #	RetTime [min]	Type	Width [min]	Area [mAU*s]	Height [mAU]	Area %
1	26.475	MF	0.7312	1.61086e4	367.15628	75.7150
2	28.065	FM	0.7419	5166.70117	116.06332	24.2850

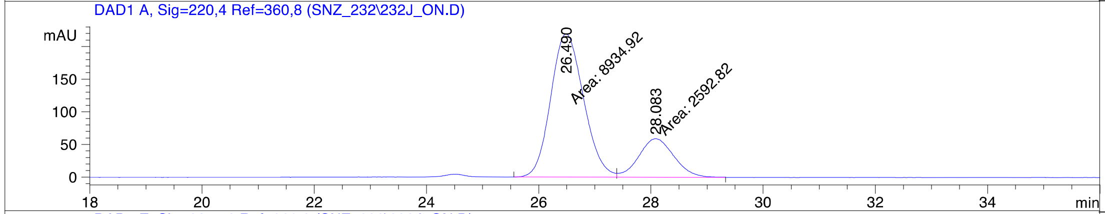

Signal 2: DAD1 A, Sig=220,4 Ref=360,8

Peak #	RetTime [min]	Type	Width [min]	Area [mAU*s]	Height [mAU]	Area %
1	26.490	MF	0.6783	8934.92090	219.54501	77.5080
2	28.083	FM	0.7292	2592.81714	59.25986	22.4920

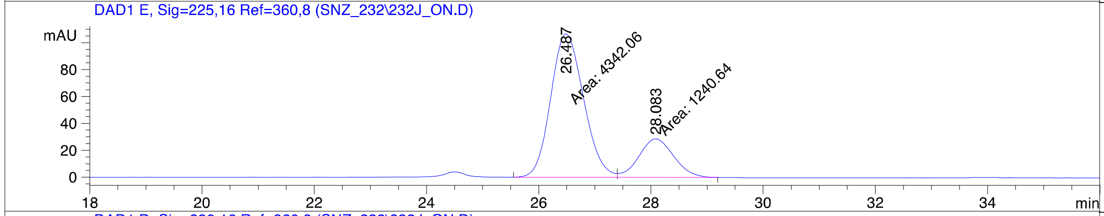

Signal 3: DAD1 E, Sig=225,16 Ref=360,8

Peak #	RetTime [min]	Type	Width [min]	Area [mAU*s]	Height [mAU]	Area %
1	26.487	MF	0.6785	4342.06348	106.65808	77.7771
2	28.083	FM	0.7213	1240.63794	28.66755	22.2229

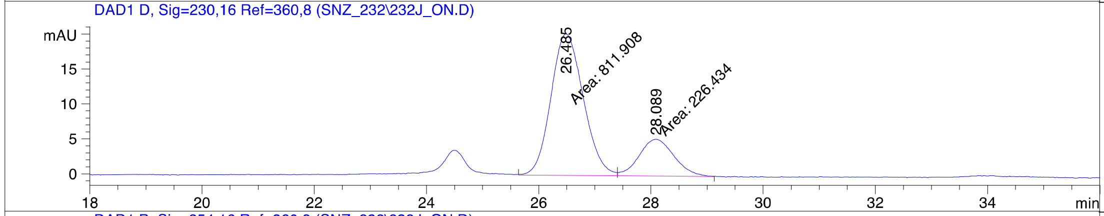

Signal 4: DAD1 D, Sig=230,16 Ref=360,8

Peak #	RetTime [min]	Type	Width [min]	Area [mAU*s]	Height [mAU]	Area %
1	26.485	MF	0.6685	811.90833	20.24269	78.1927
2	28.089	FM	0.7167	226.43408	5.26570	21.8073

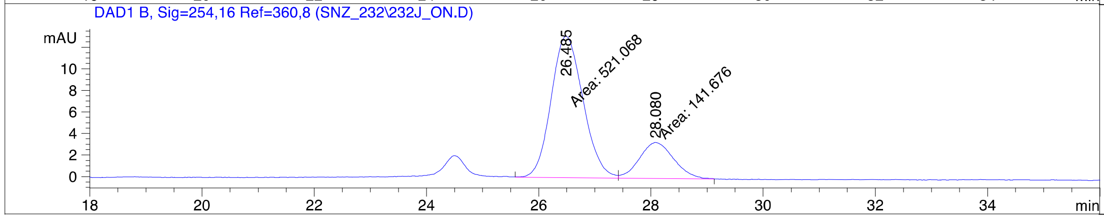

Signal 5: DAD1 B, Sig=254,16 Ref=360,8

Peak #	RetTime [min]	Type	Width [min]	Area [mAU*s]	Height [mAU]	Area %
1	26.485	MF	0.6612	521.06824	13.13412	78.6229
2	28.080	FM	0.7076	141.67561	3.33714	21.3771

Copies of ^{1}H and ^{13}C NMR spectra

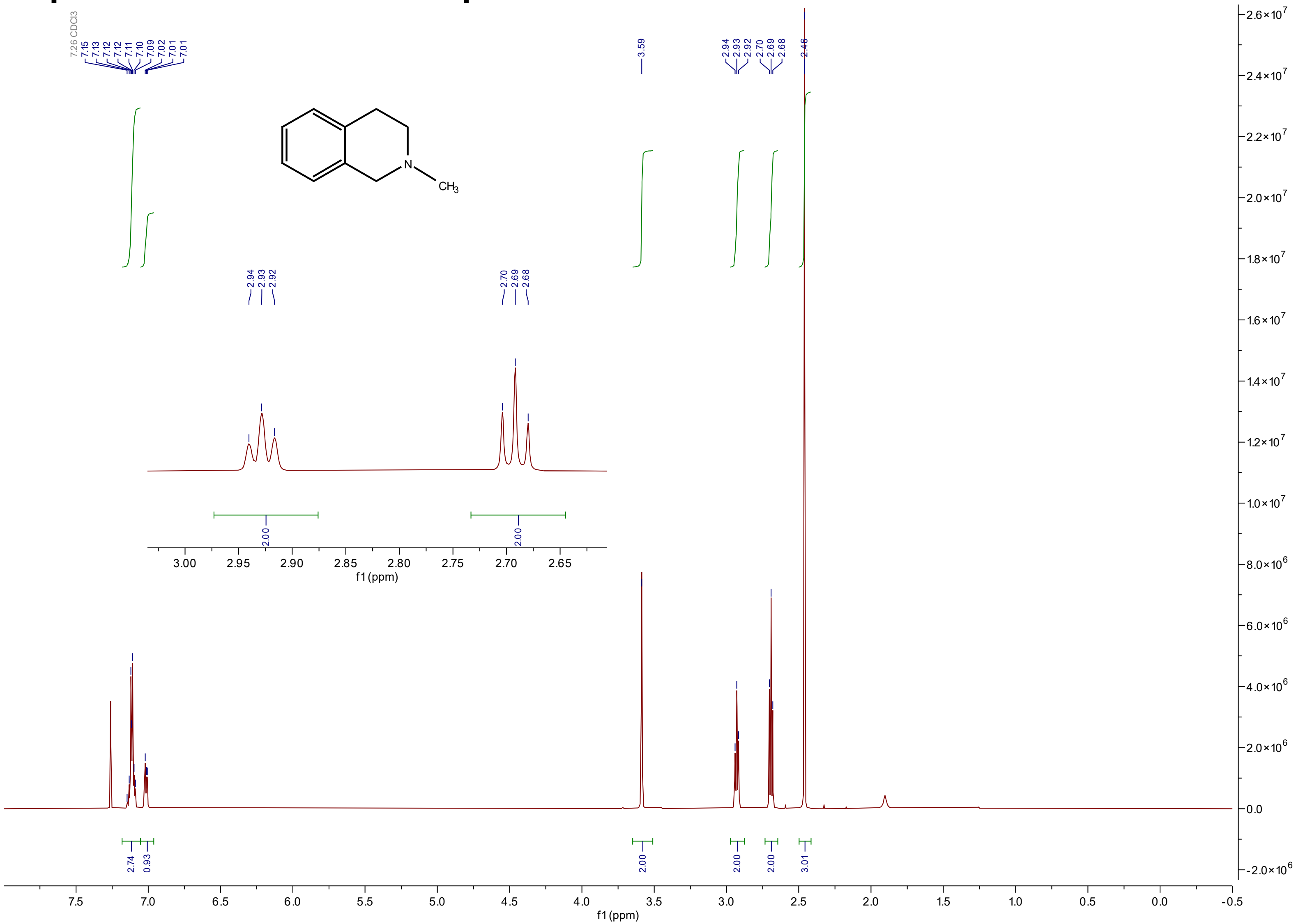

Fig. S72. ^{1}H NMR (500 MHz) of 2-methyl-1,2,3,4-tetrahydroisoquinoline (**1a**).

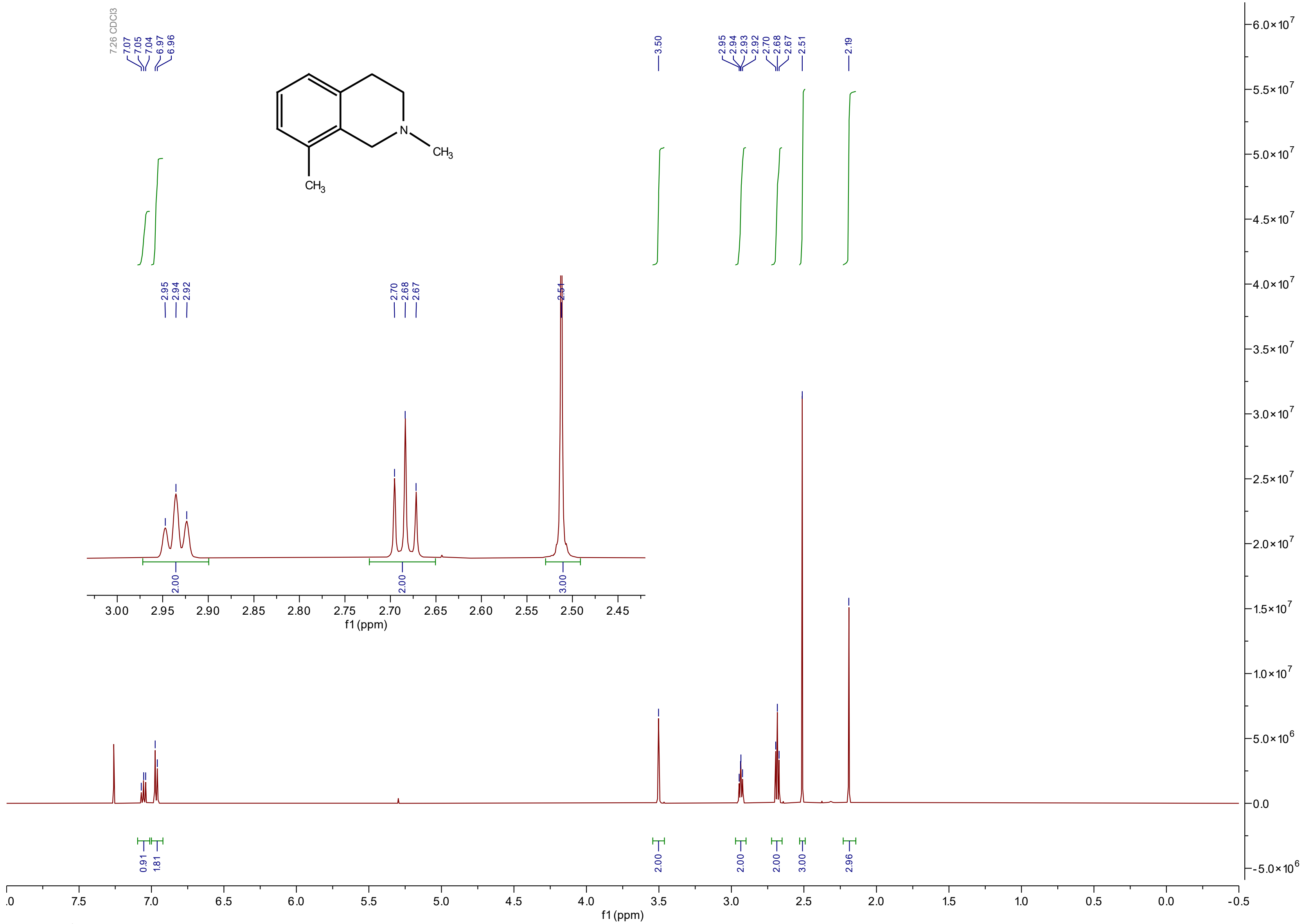

Fig. S73. ^{1}H NMR (500 MHz) of 2,8-dimethyl-1,2,3,4-tetrahydroisoquinoline (**1b**).

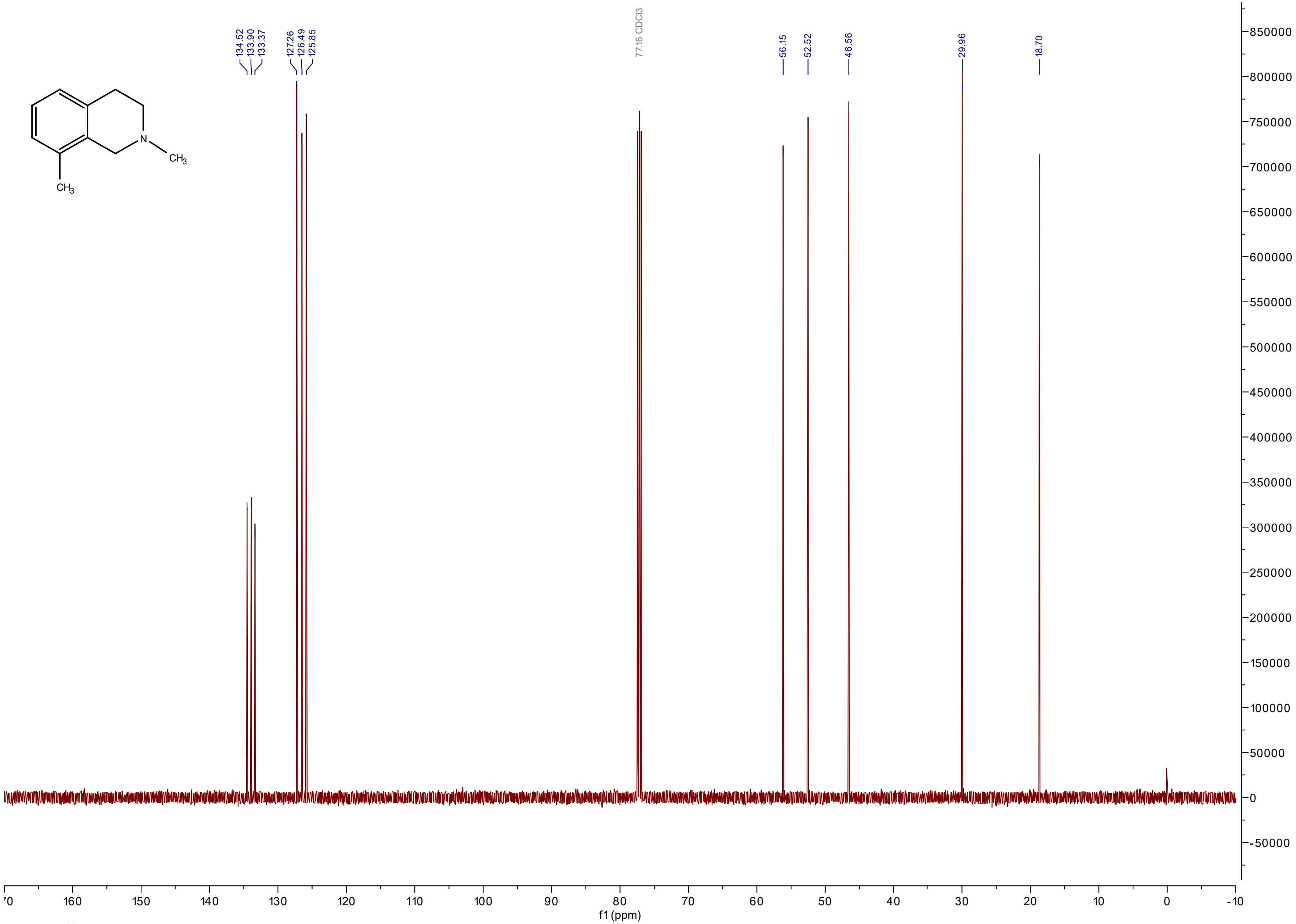

Fig. S74. ^{13}C NMR (126 MHz) of 2,8-dimethyl-1,2,3,4-tetrahydroisoquinoline (**1b**).

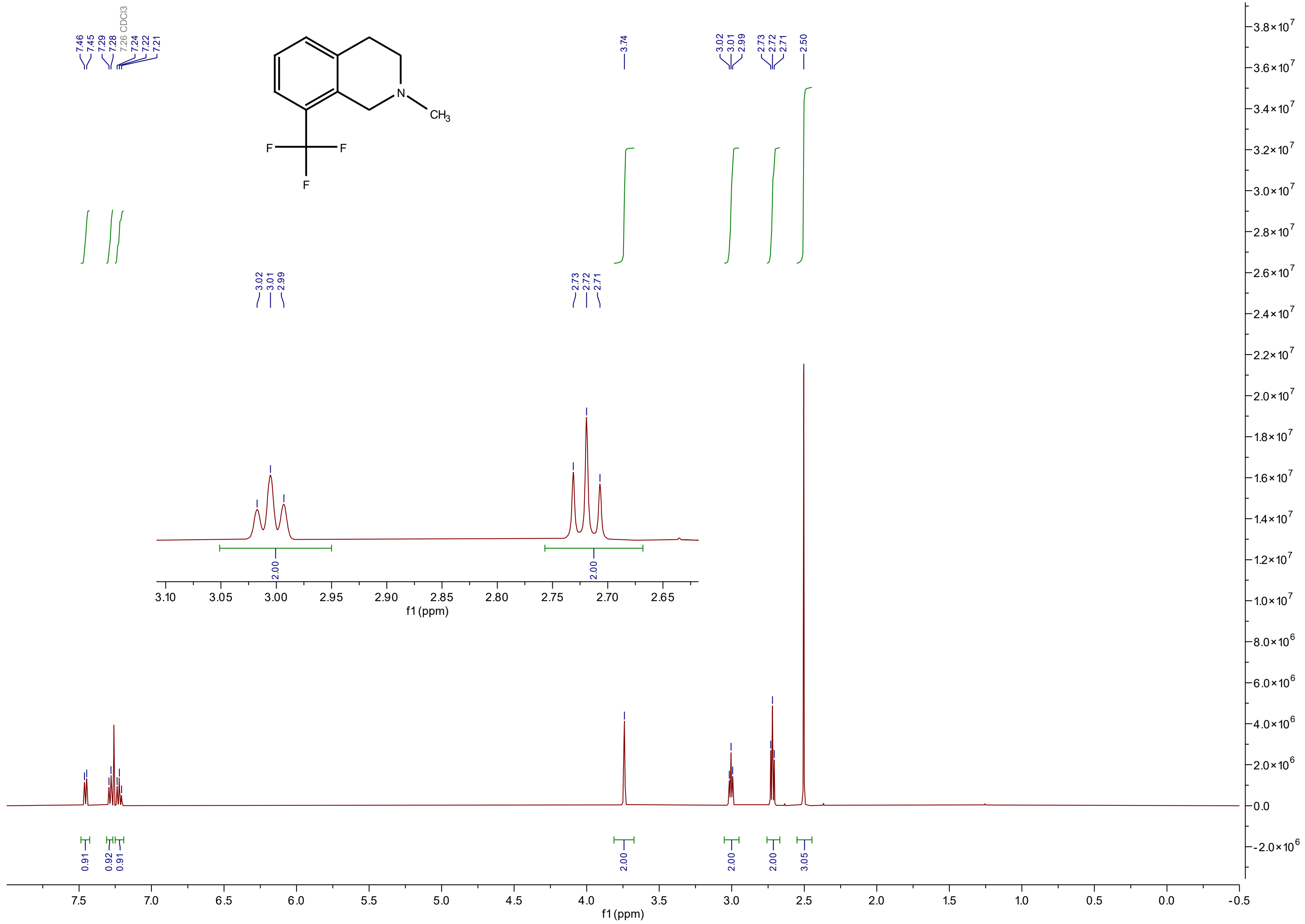

Fig. S75. ^{1}H NMR (500 MHz) of 2-methyl-8-(trifluoromethyl)-1,2,3,4-tetrahydroisoquinoline (**1c**).

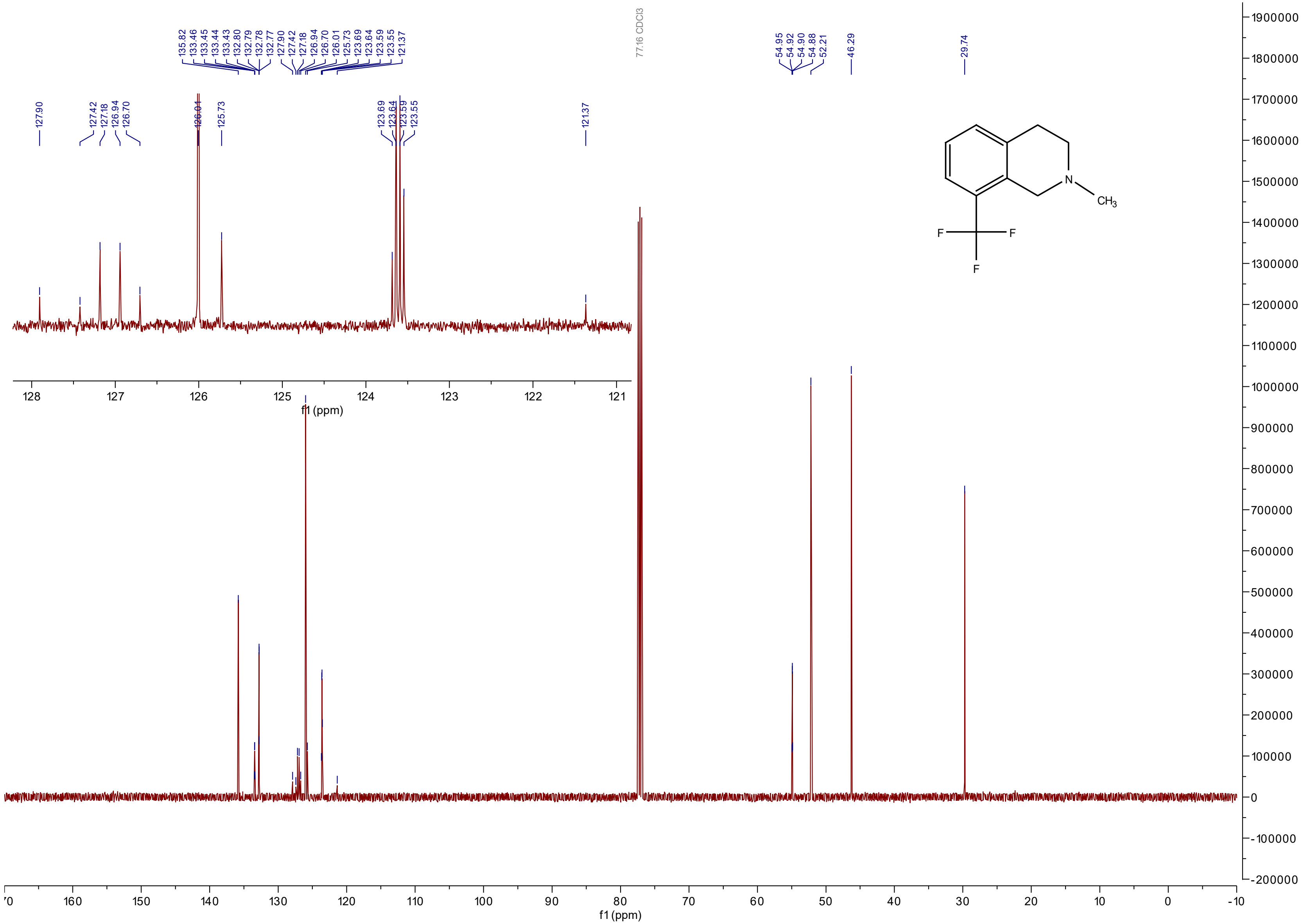

Fig. S76. ^{13}C NMR (126 MHz) of 2-methyl-8-(trifluoromethyl)-1,2,3,4-tetrahydroisoquinoline (**1c**).

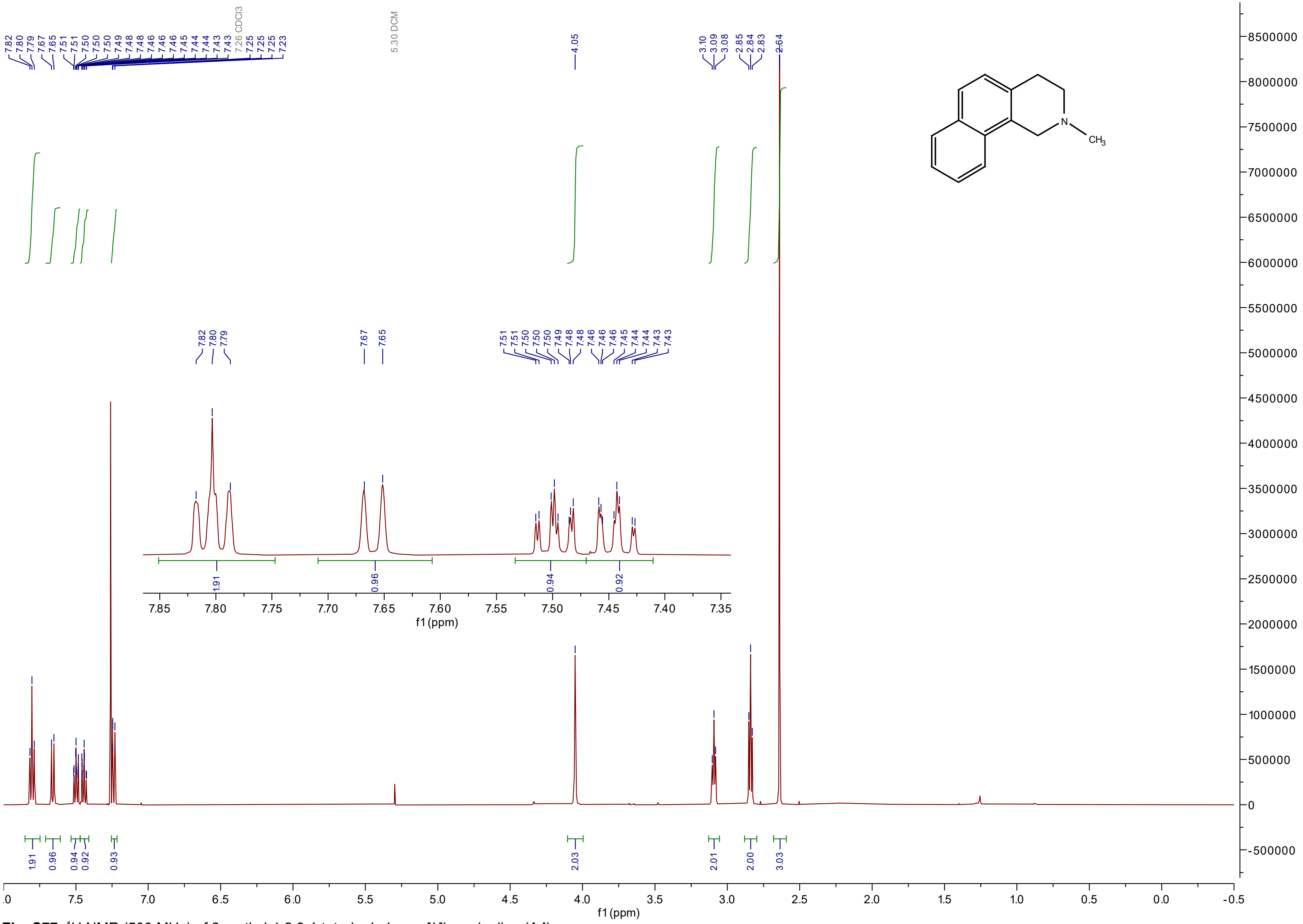

Fig. S77. ^1H NMR (500 MHz) of 2-methyl-1,2,3,4-tetrahydrobenzo[*h*]isoquinoline (**1d**).

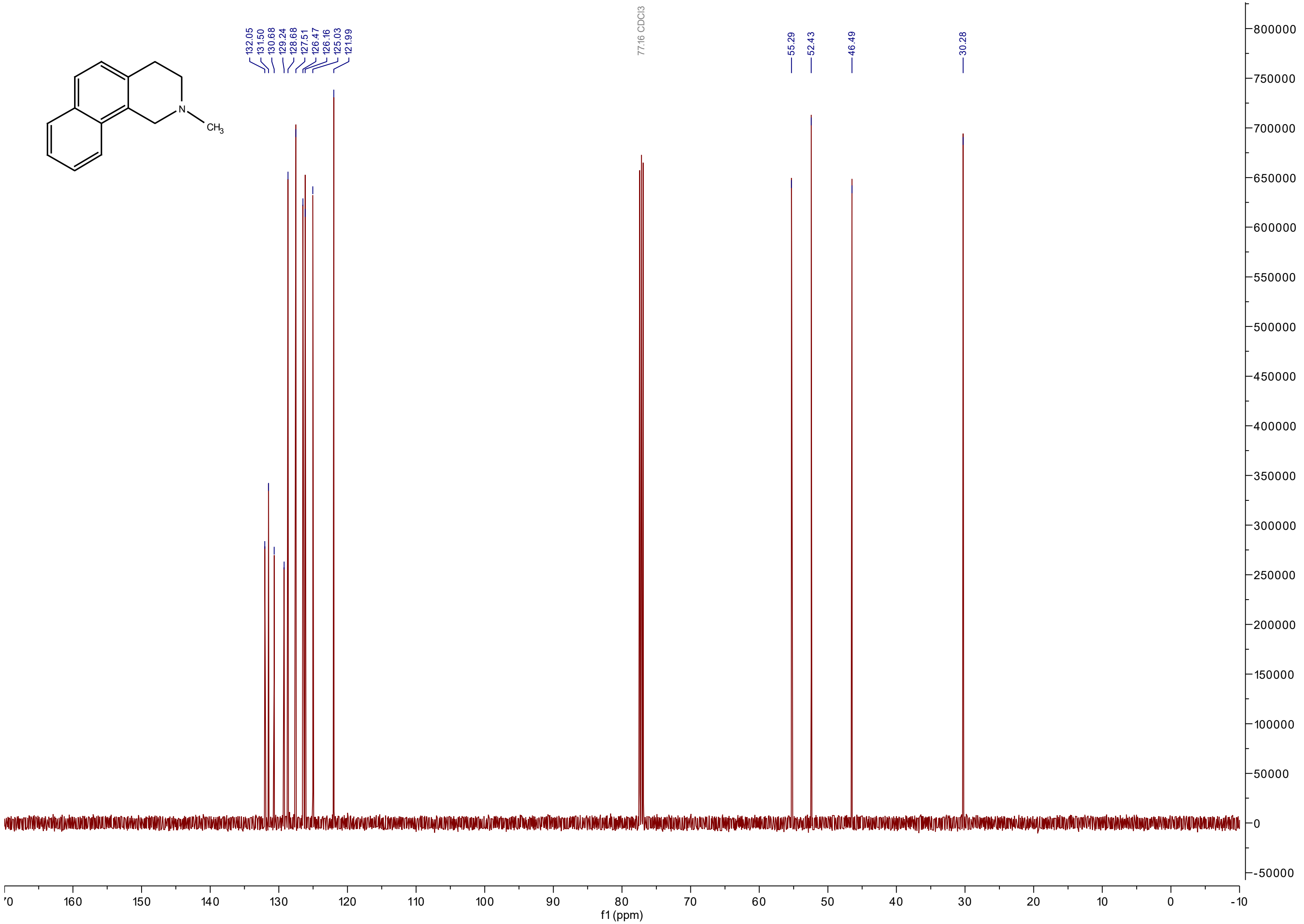

Fig. S78. ^{13}C NMR (126 MHz) of 2-methyl-1,2,3,4-tetrahydrobenzo[*h*]isoquinoline (**1d**).

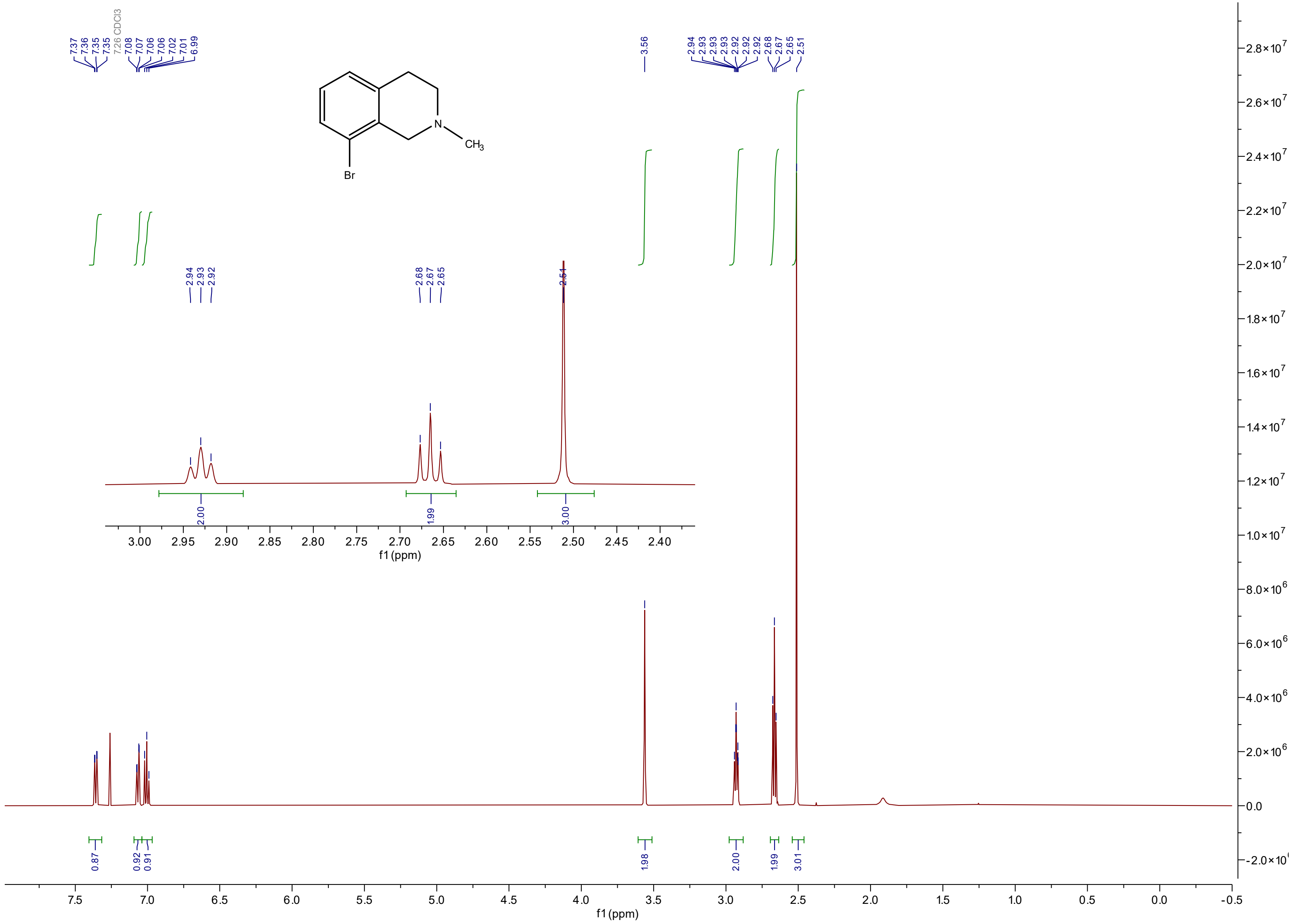

Fig. S79. ^{1}H NMR (500 MHz) of 8-bromo-2-methyl-1,2,3,4-tetrahydroisoquinoline (**1e**).

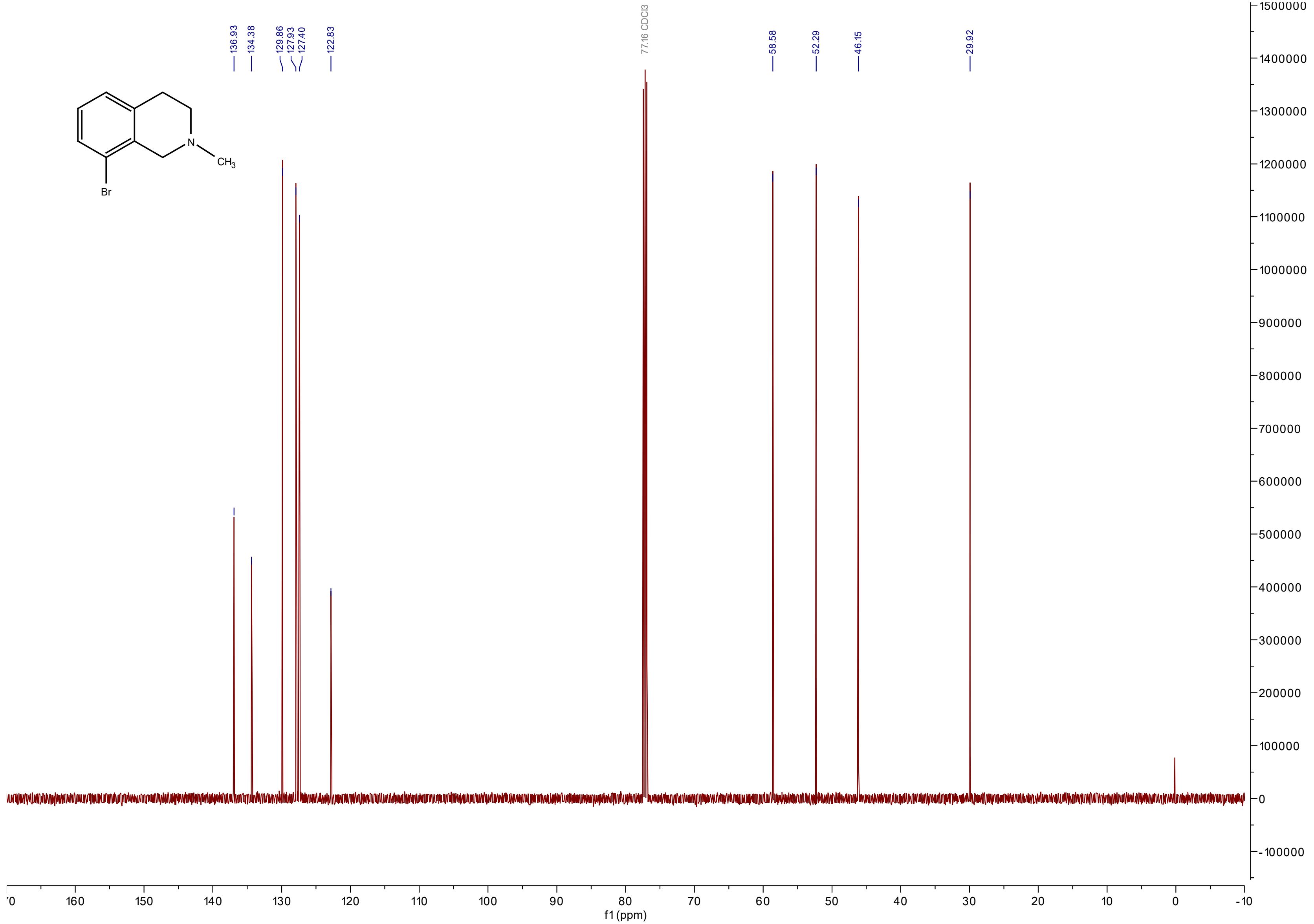

Fig. S80. ^{13}C NMR (126 MHz) of 8-bromo-2-methyl-1,2,3,4-tetrahydroisoquinoline (**1e**).

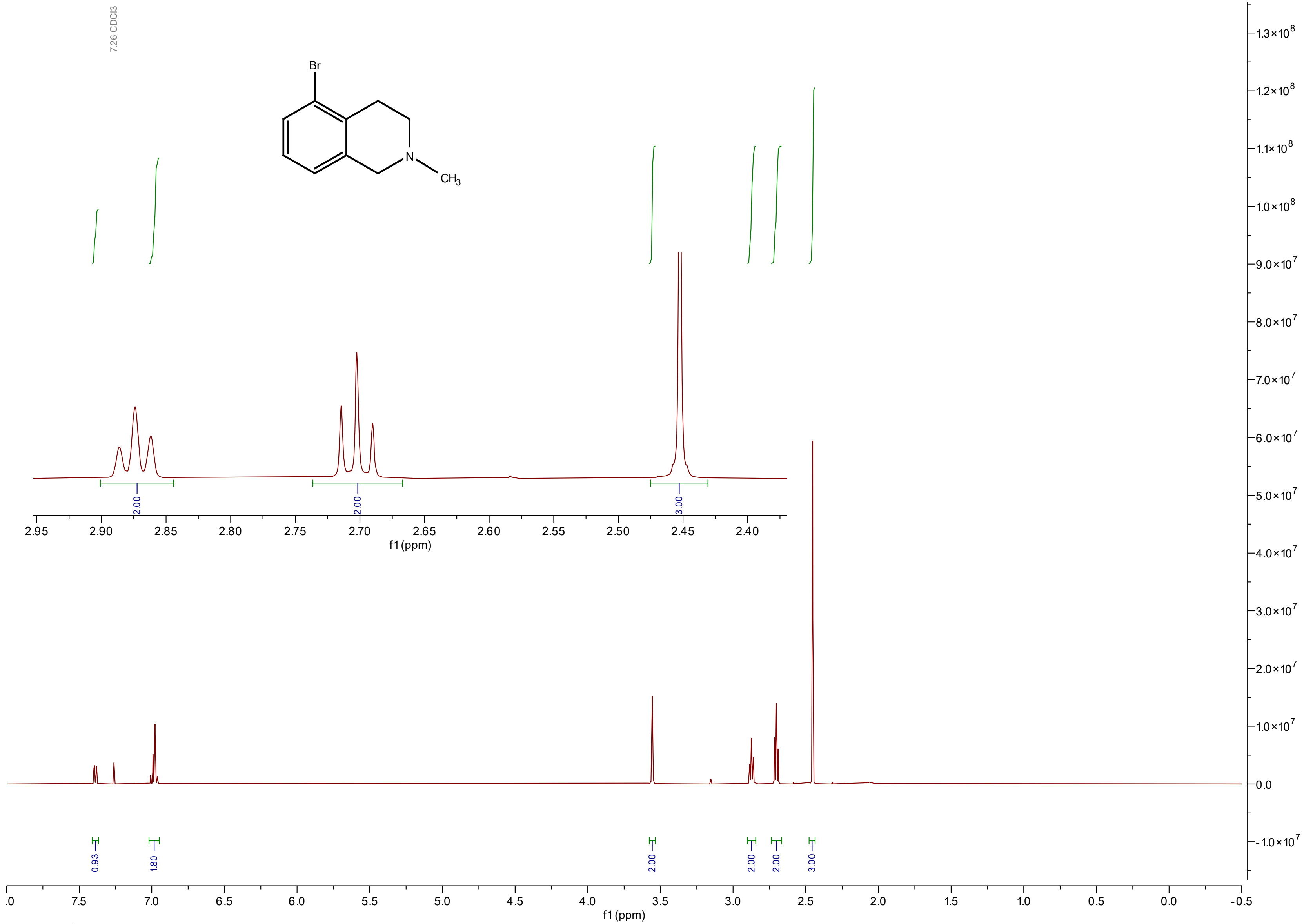

Fig. S81. ^{1}H NMR (500 MHz) of 5-bromo-2-methyl-1,2,3,4-tetrahydroisoquinoline (**1f**).

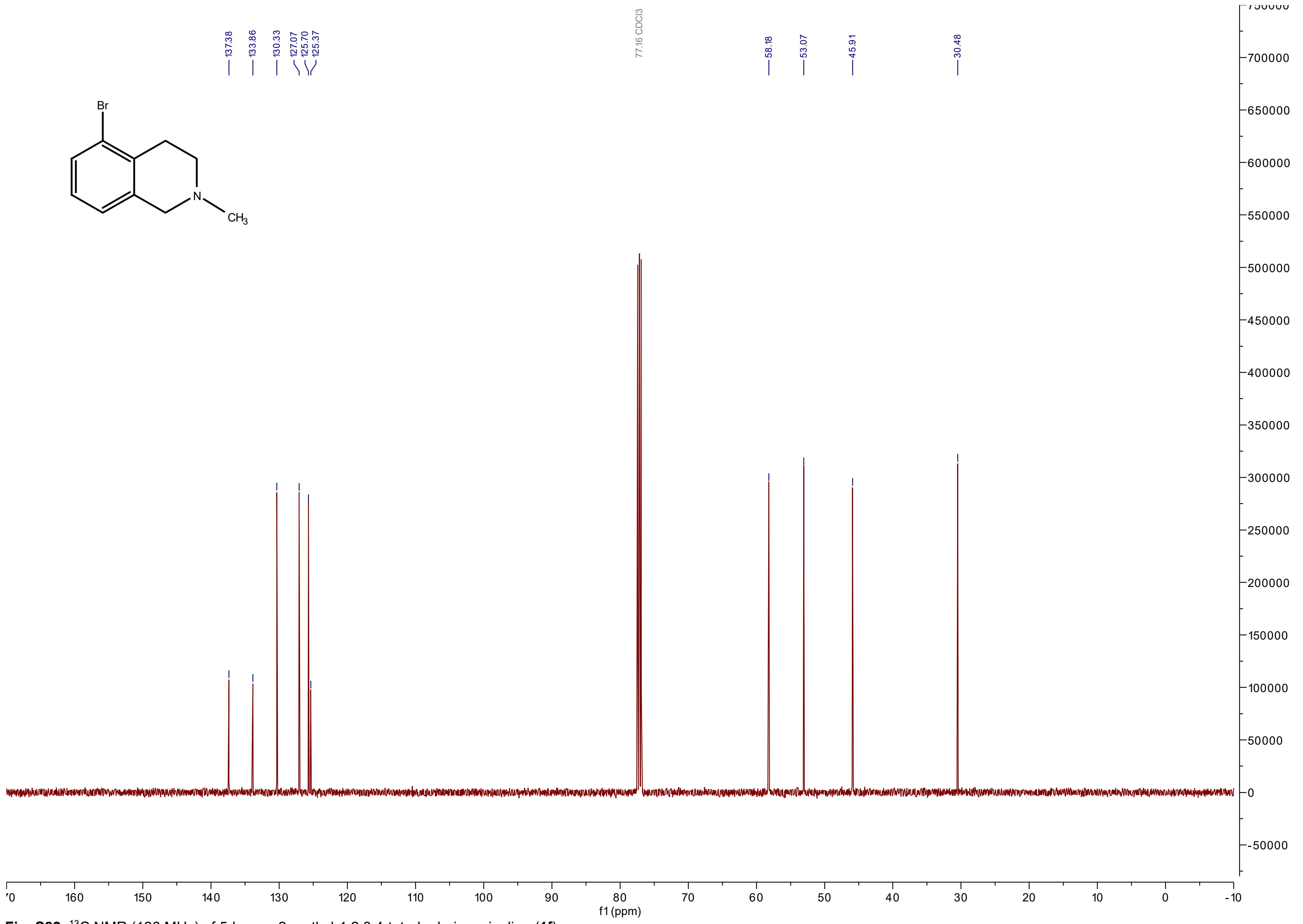

Fig. S82. ^{13}C NMR (126 MHz) of 5-bromo-2-methyl-1,2,3,4-tetrahydroisoquinoline (**1f**).

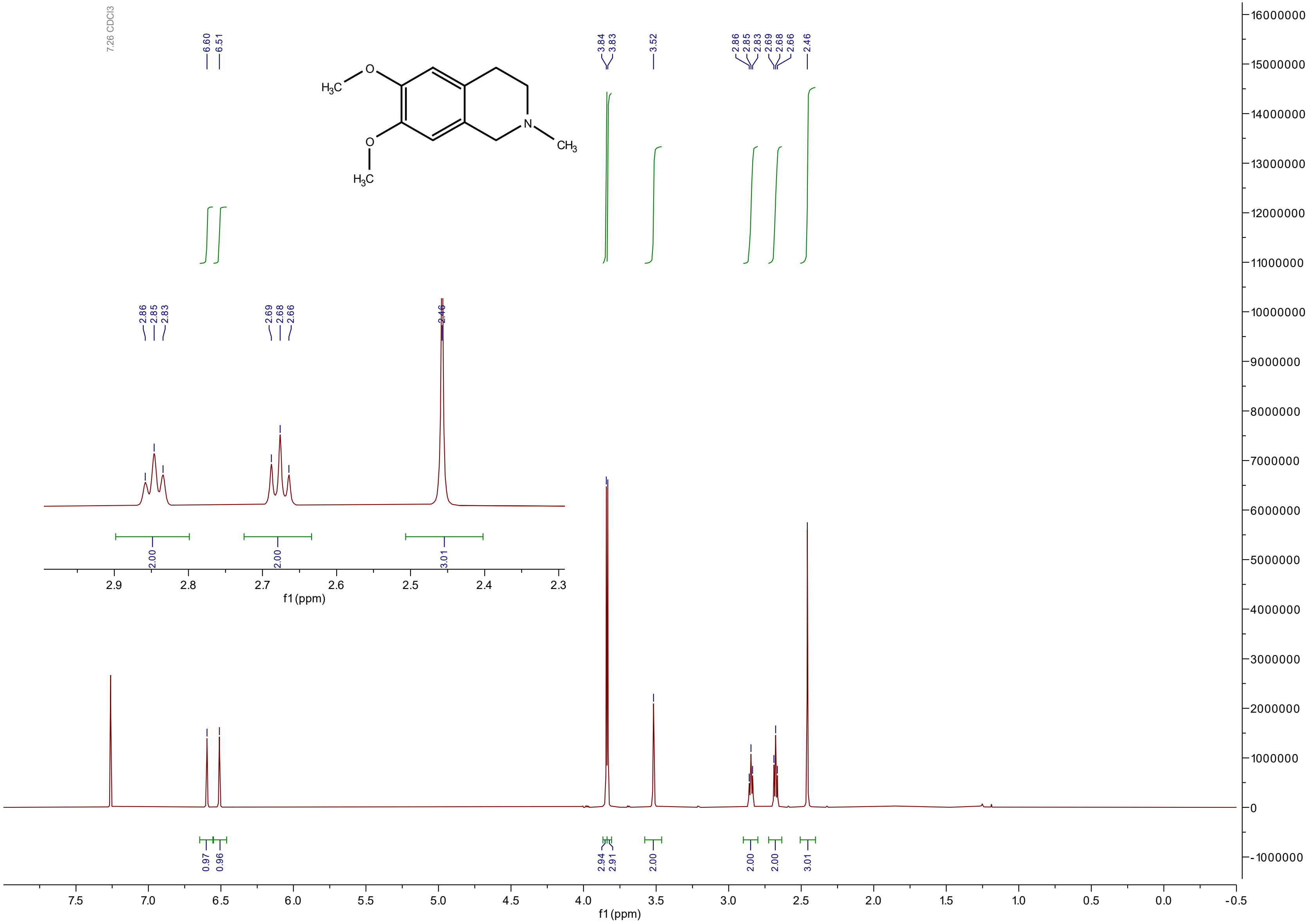

Fig. S83. ^{1}H NMR (500 MHz) of 6,7-dimethoxy-2-methyl-1,2,3,4-tetrahydroisoquinoline (**1g**).

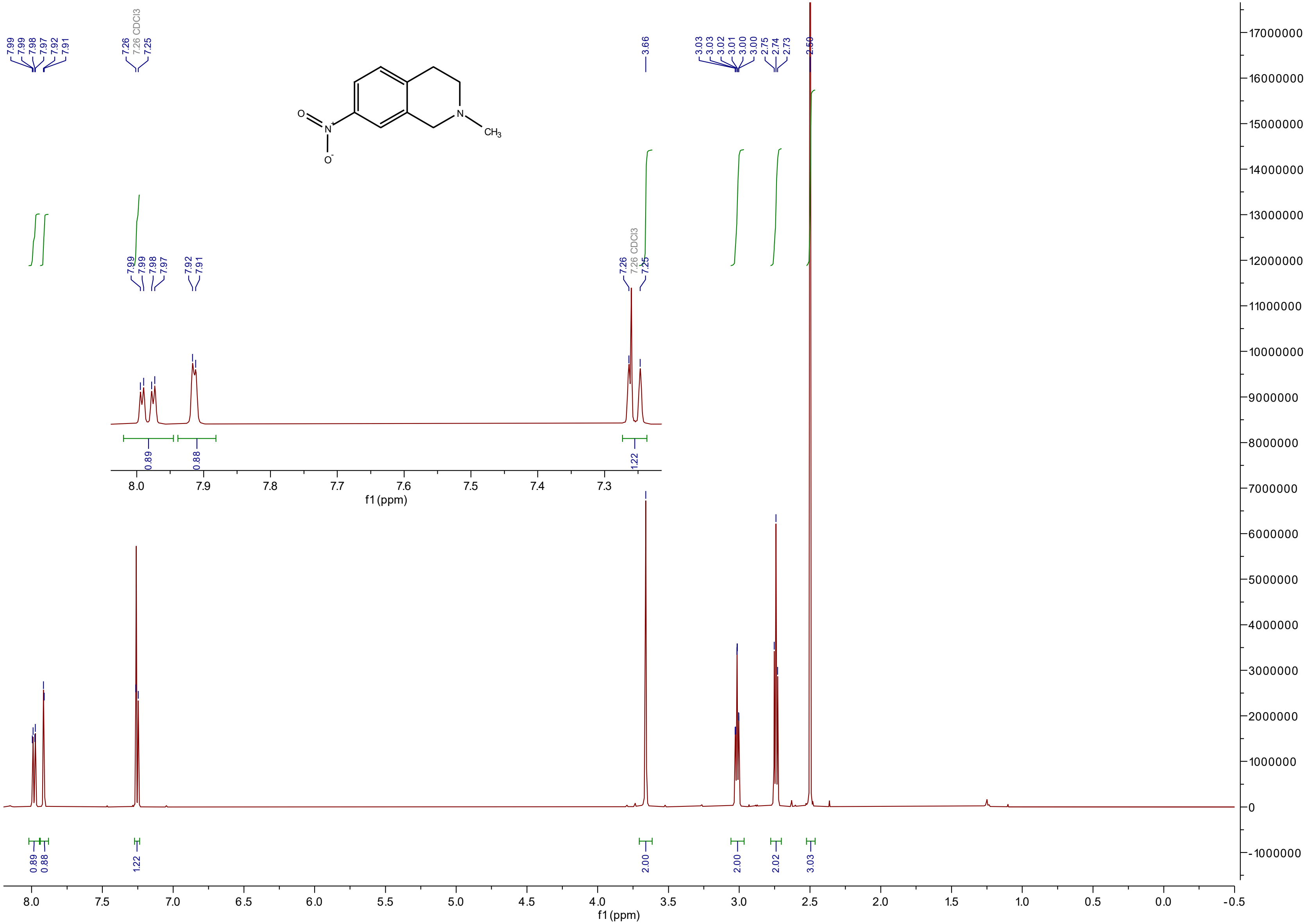

Fig. S84. ^{1}H NMR (500 MHz) of 2-methyl-7-nitro-1,2,3,4-tetrahydroisoquinoline (**1h**).

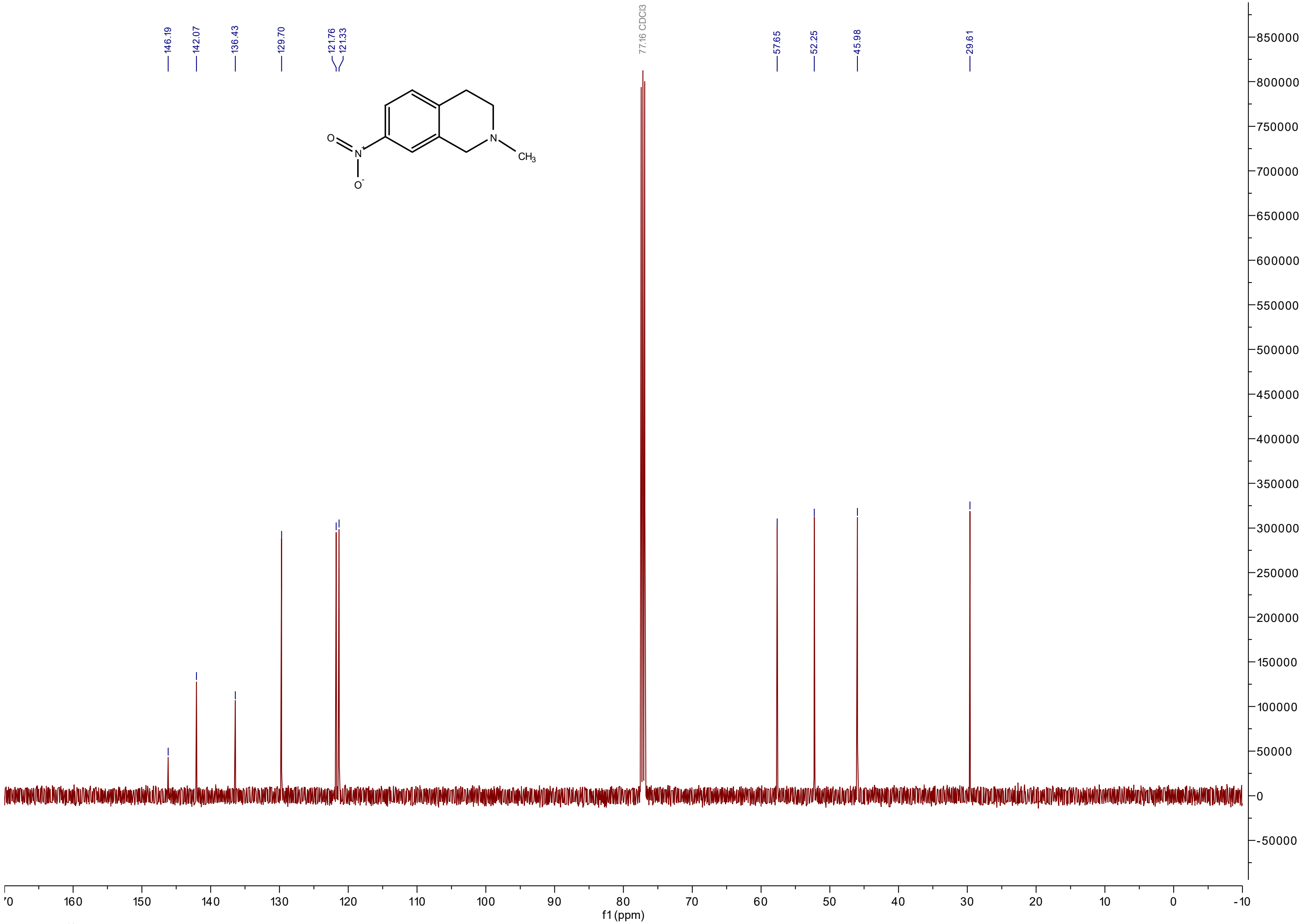

Fig. S85. ^{13}C NMR (126 MHz) of 2-methyl-7-nitro-1,2,3,4-tetrahydroisoquinoline (**1h**).

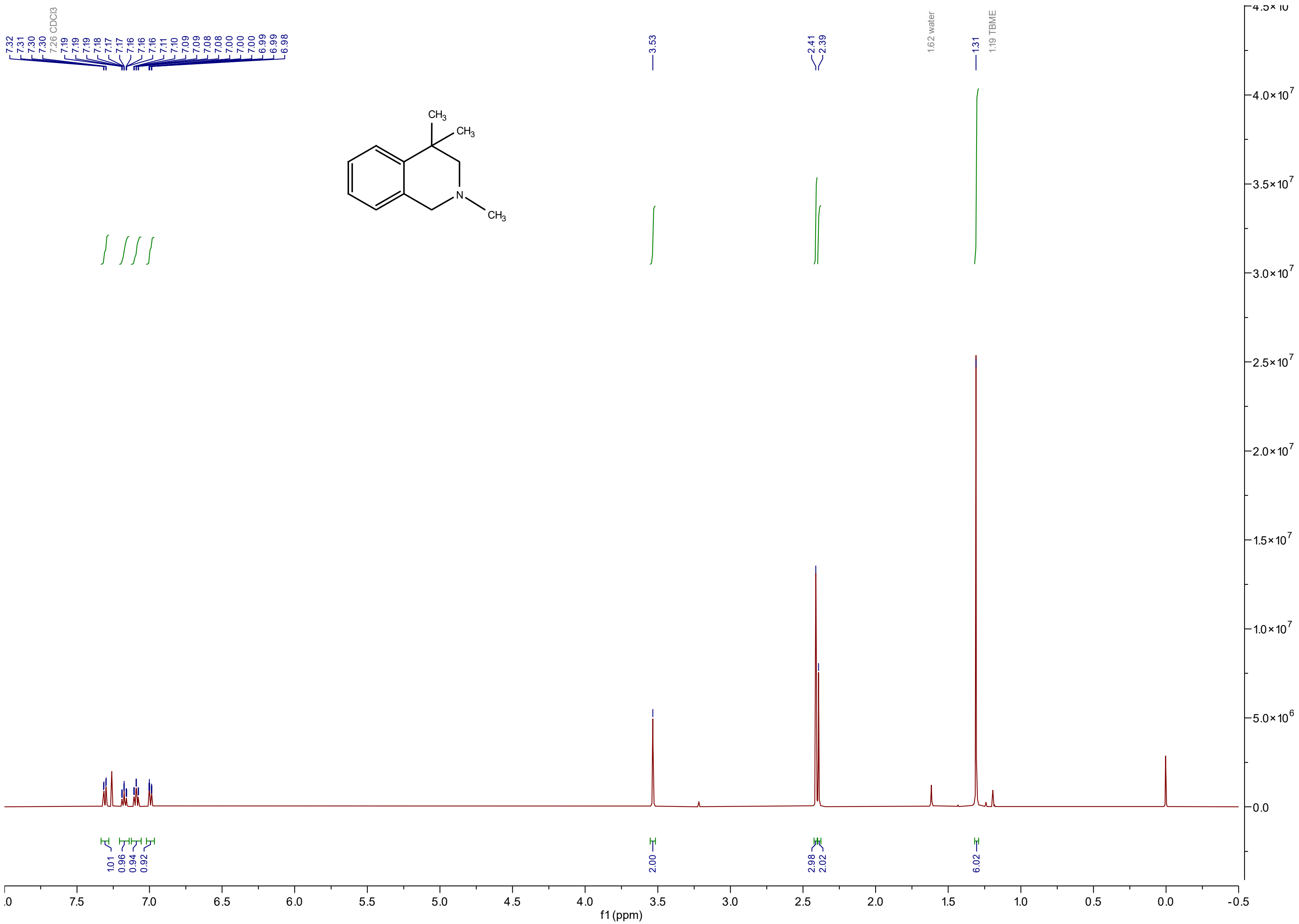

Fig. S86. ^{1}H NMR (500 MHz) of 2,4,4-trimethyl-1,2,3,4-tetrahydroisoquinoline (**1i**).

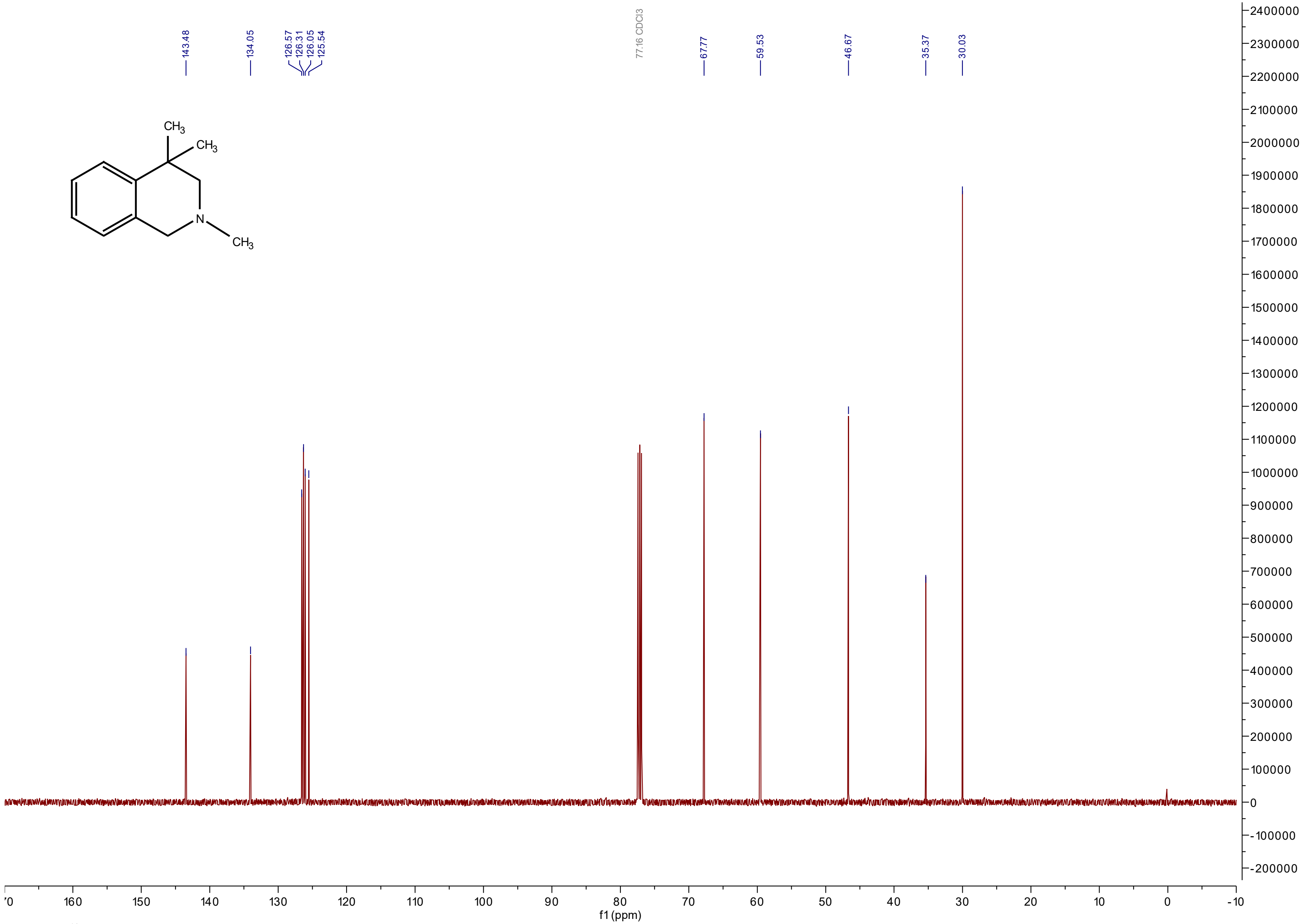

Fig. S87. ^{13}C NMR (126 MHz) of 2,4,4-trimethyl-1,2,3,4-tetrahydroisoquinoline (**1i**).

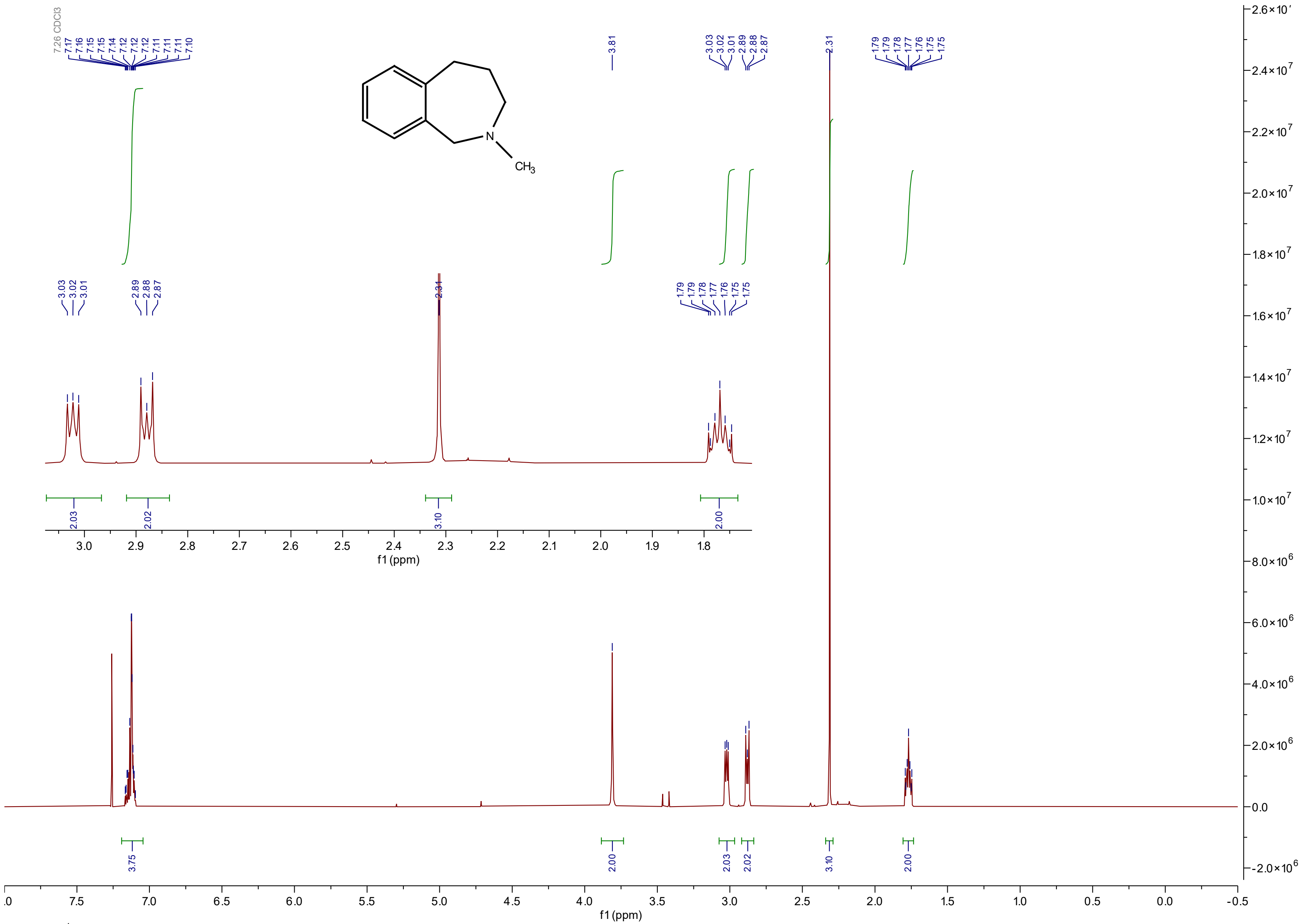

Fig. S88. ^{1}H NMR (500 MHz) of 2-methyl-2,3,4,5-tetrahydro-1*H*-benzo[*c*]azepine (**1j**).

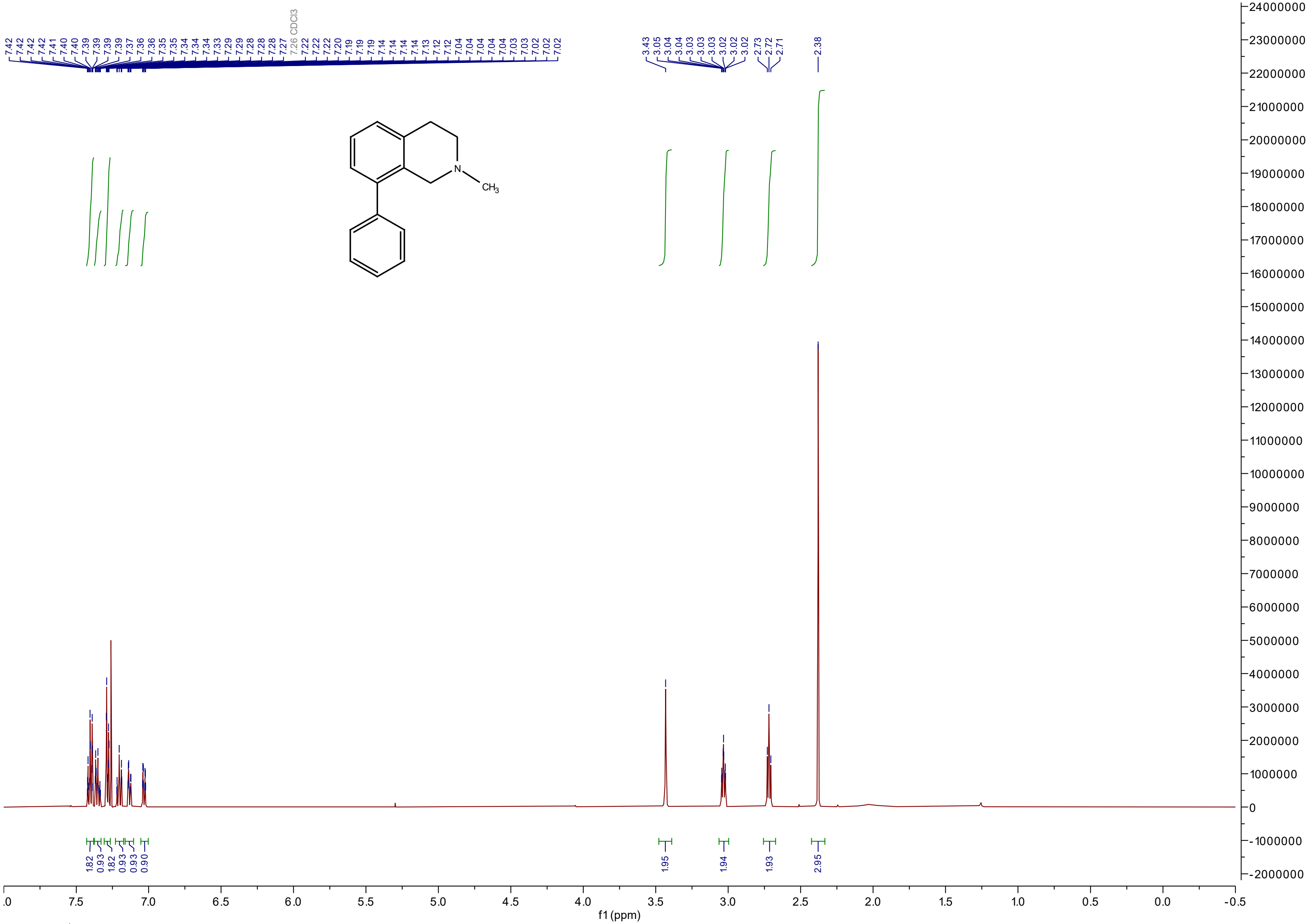

Fig. S89. ^{1}H NMR (500 MHz) of 2-methyl-8-phenyl-1,2,3,4-tetrahydroisoquinoline (**1k**).

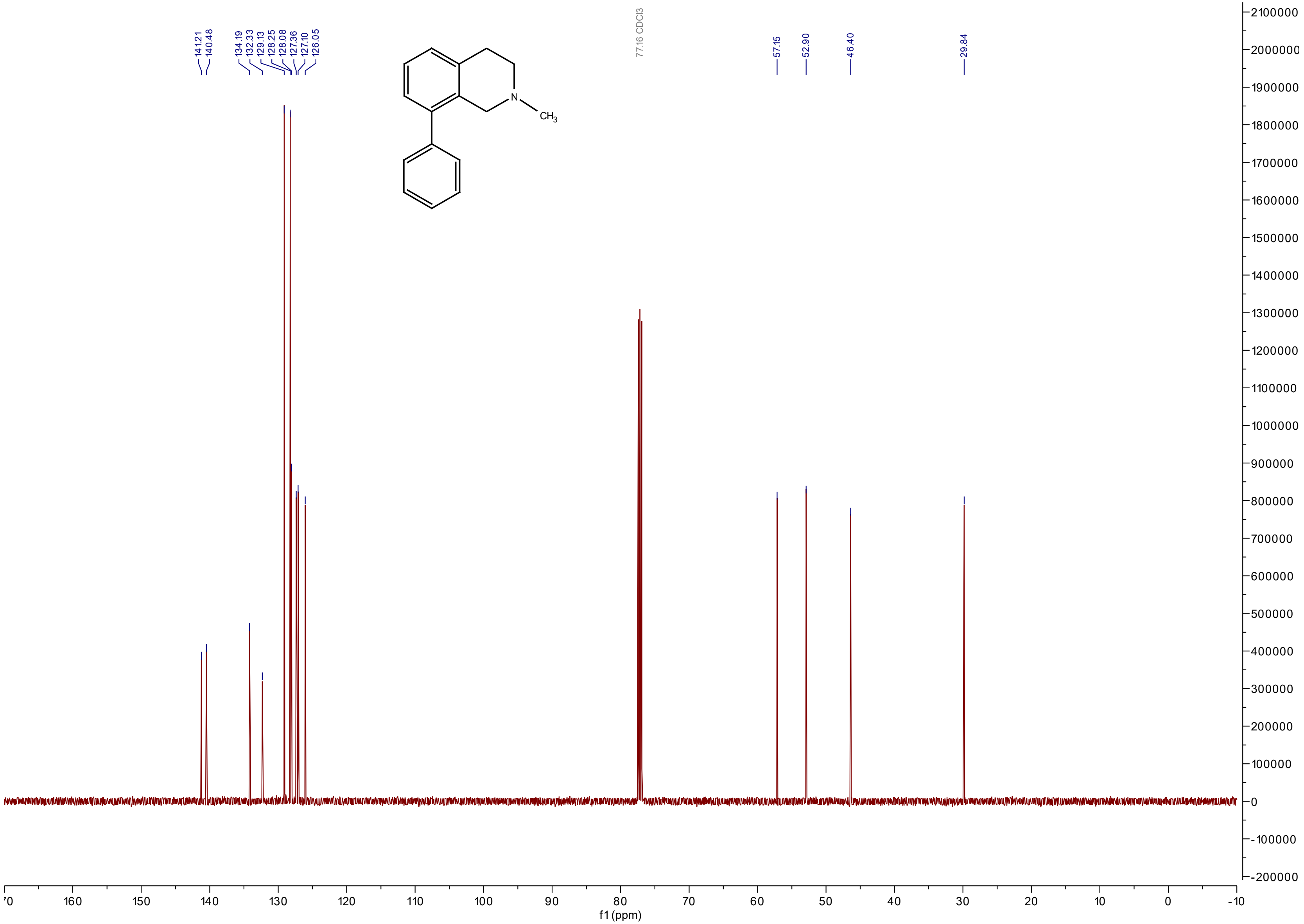

Fig. S90. ^{13}C NMR (126 MHz) of 2-methyl-8-phenyl-1,2,3,4-tetrahydroisoquinoline (**1k**).

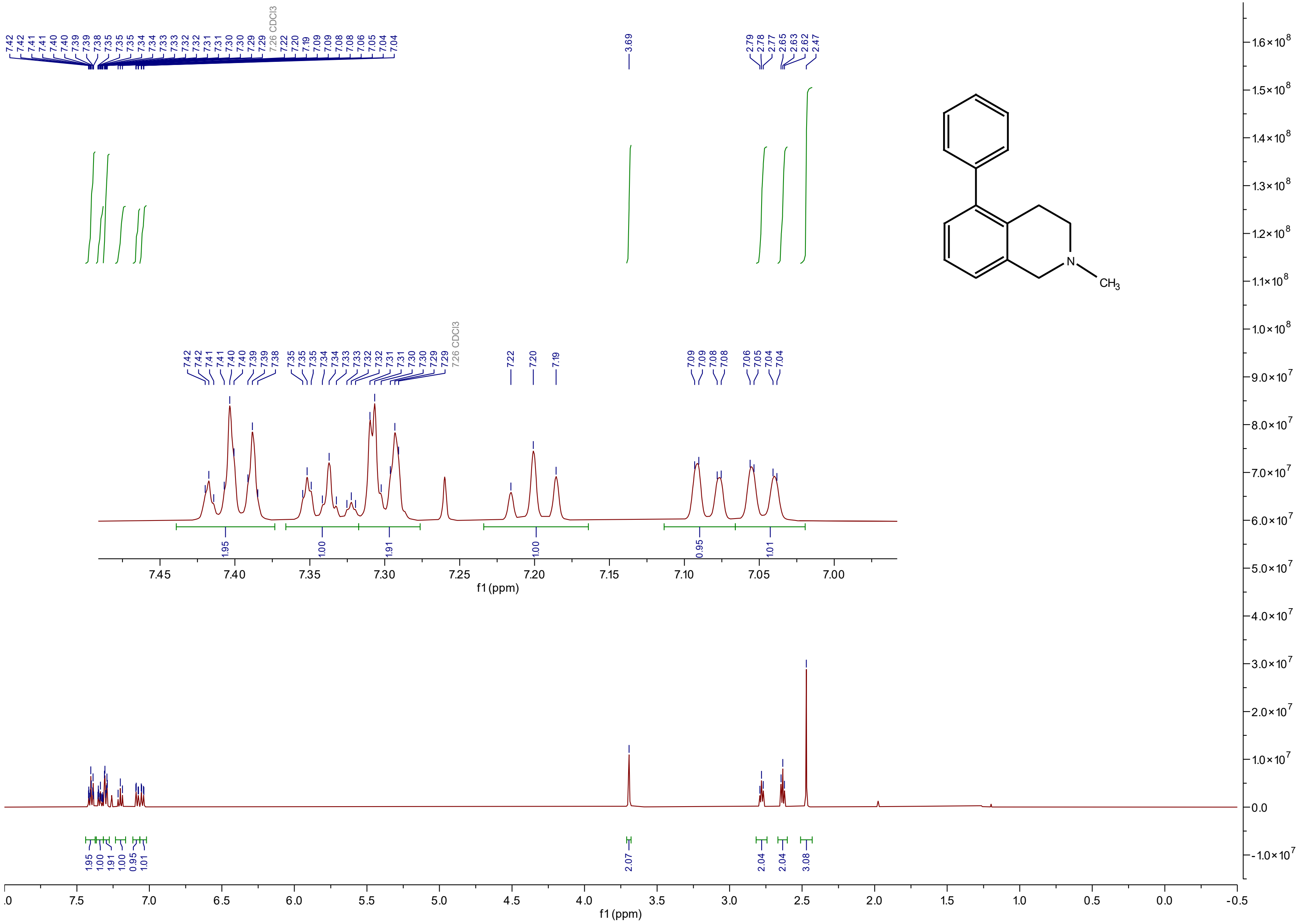

Fig. S91. ^{1}H NMR (500 MHz) of 2-methyl-5-phenyl-1,2,3,4-tetrahydroisoquinoline (**1l**).

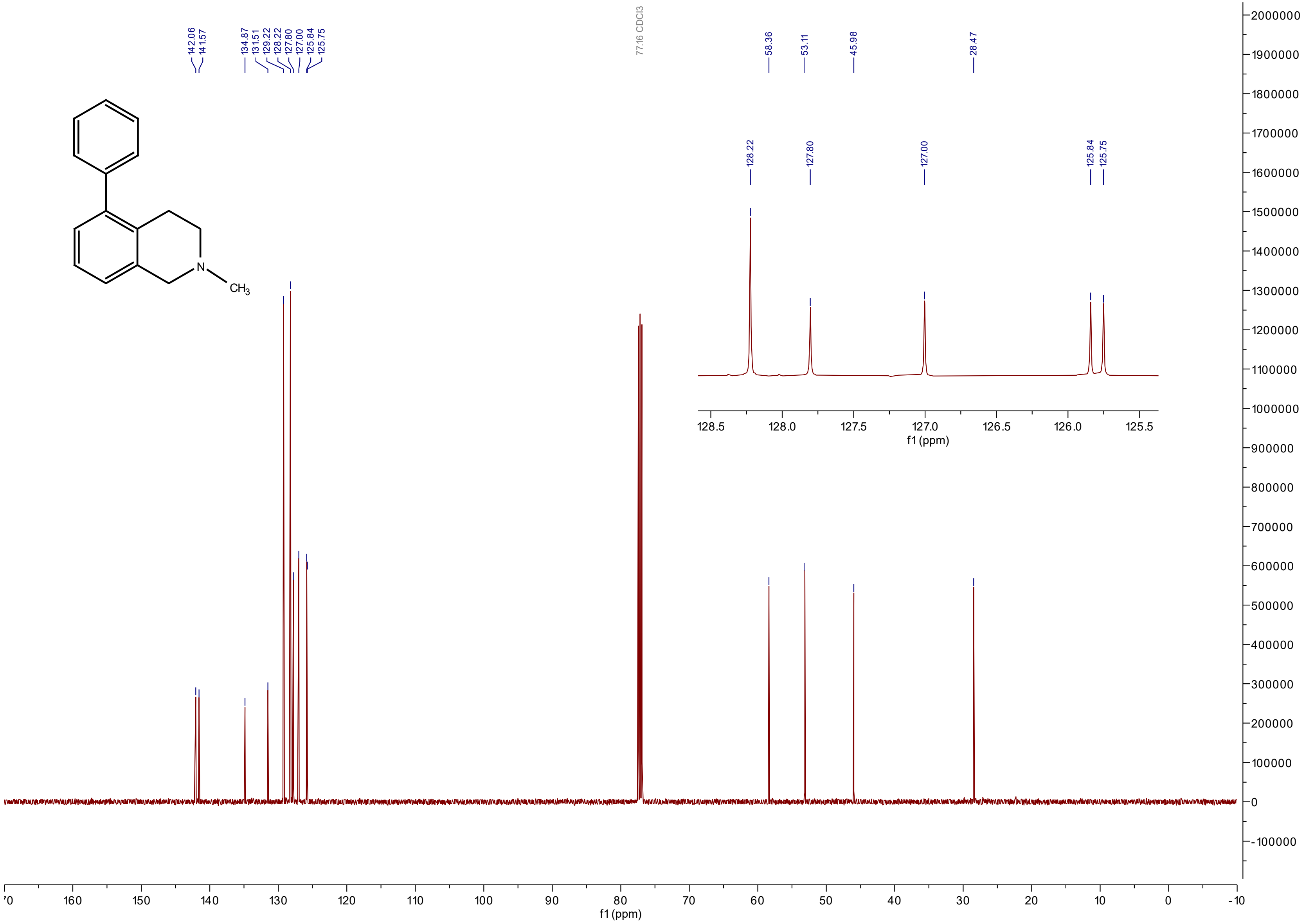

Fig. S92. ^{13}C NMR (126 MHz) of 2-methyl-5-phenyl-1,2,3,4-tetrahydroisoquinoline (**1l**).

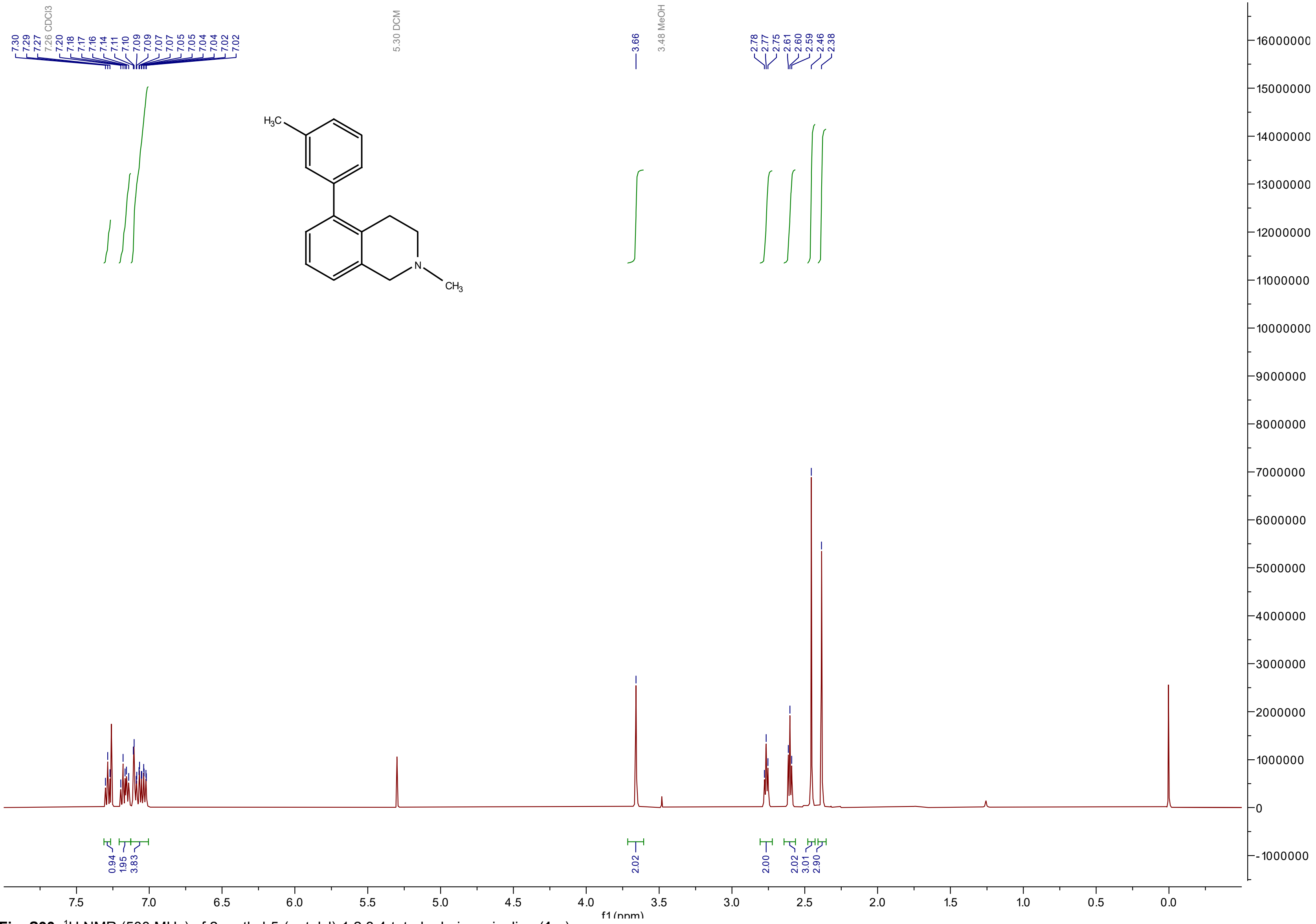

Fig. S93. ^{1}H NMR (500 MHz) of 2-methyl-5-(*m*-tolyl)-1,2,3,4-tetrahydroisoquinoline (**1m**).

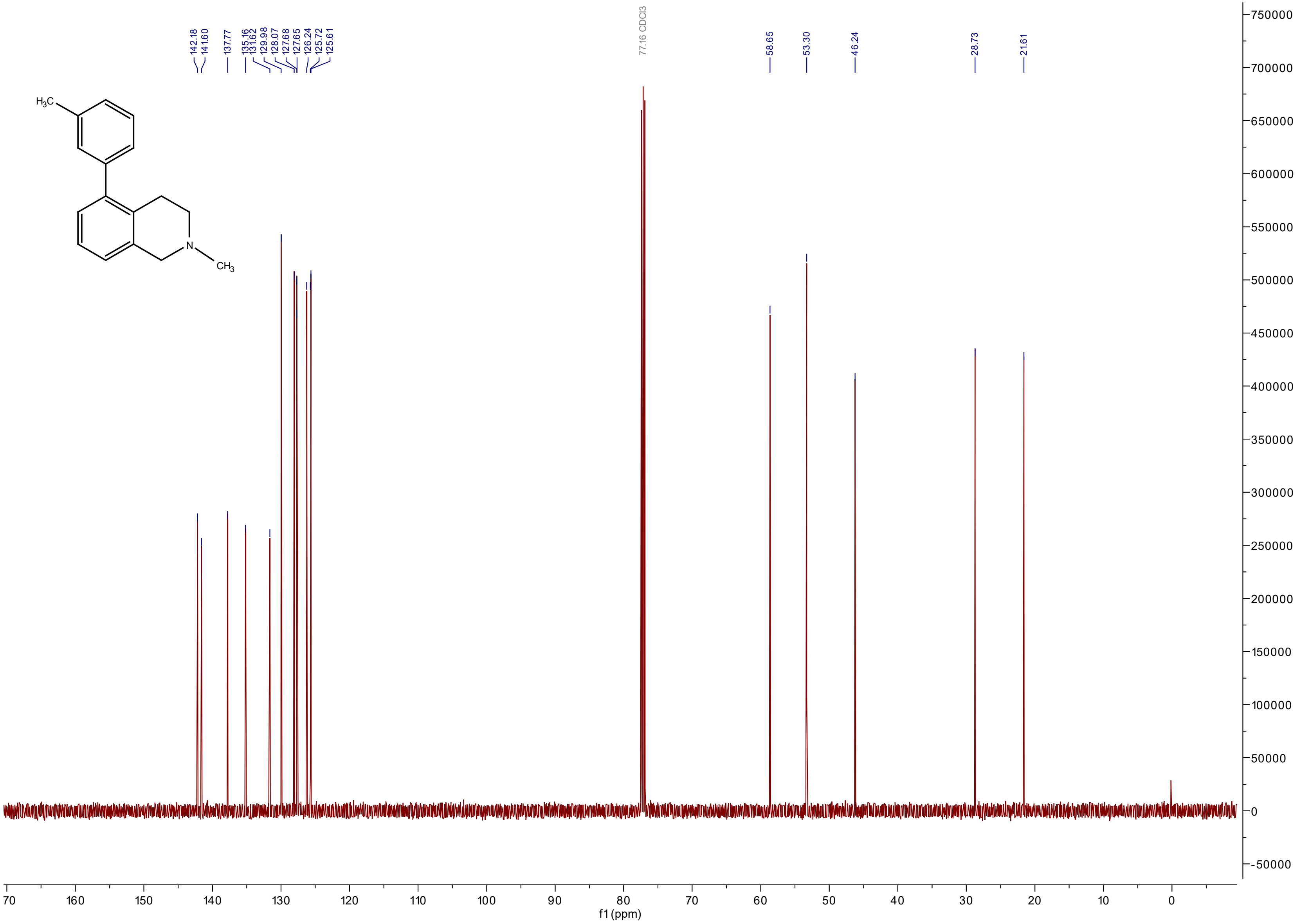

Fig. S94. ^{13}C NMR (126 MHz) of 2-methyl-5-(*m*-tolyl)-1,2,3,4-tetrahydroisoquinoline (**1m**).

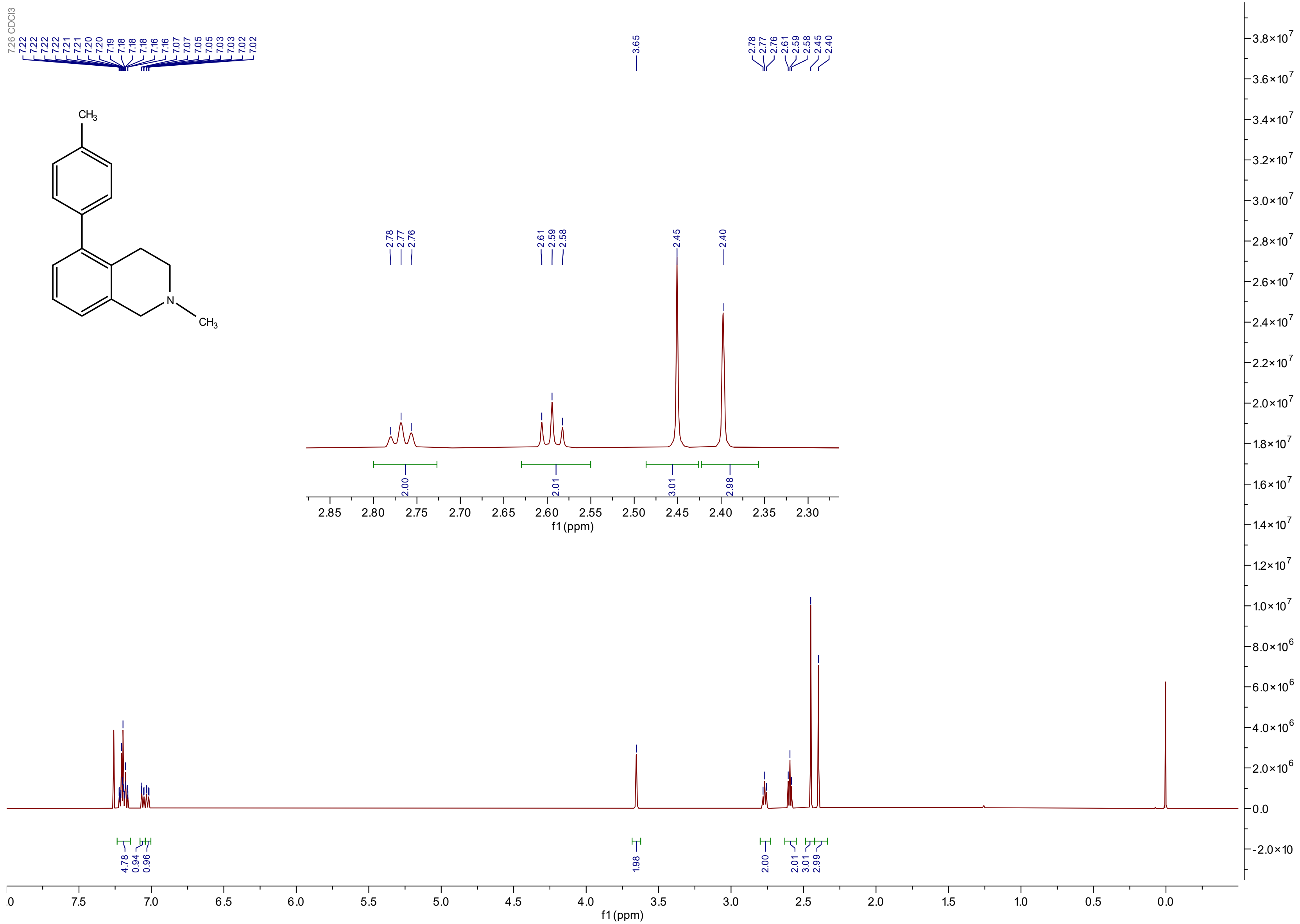

Fig. S95. ^{1}H NMR (500 MHz) of 2-methyl-5-(*p*-tolyl)-1,2,3,4-tetrahydroisoquinoline (**1n**).

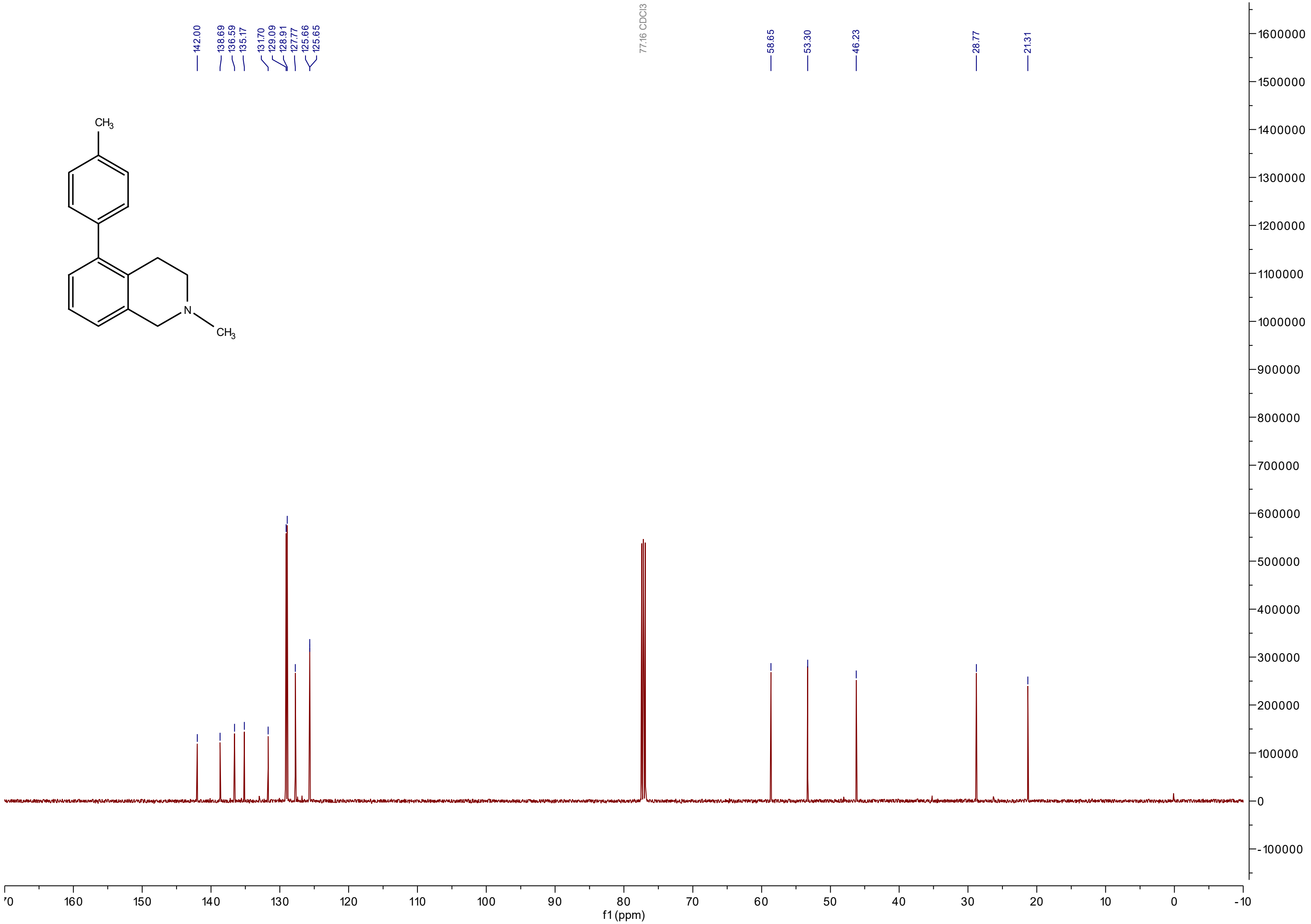

Fig. S96. ^{13}C NMR (126 MHz) of 2-methyl-5-(*p*-tolyl)-1,2,3,4-tetrahydroisoquinoline (**1n**).

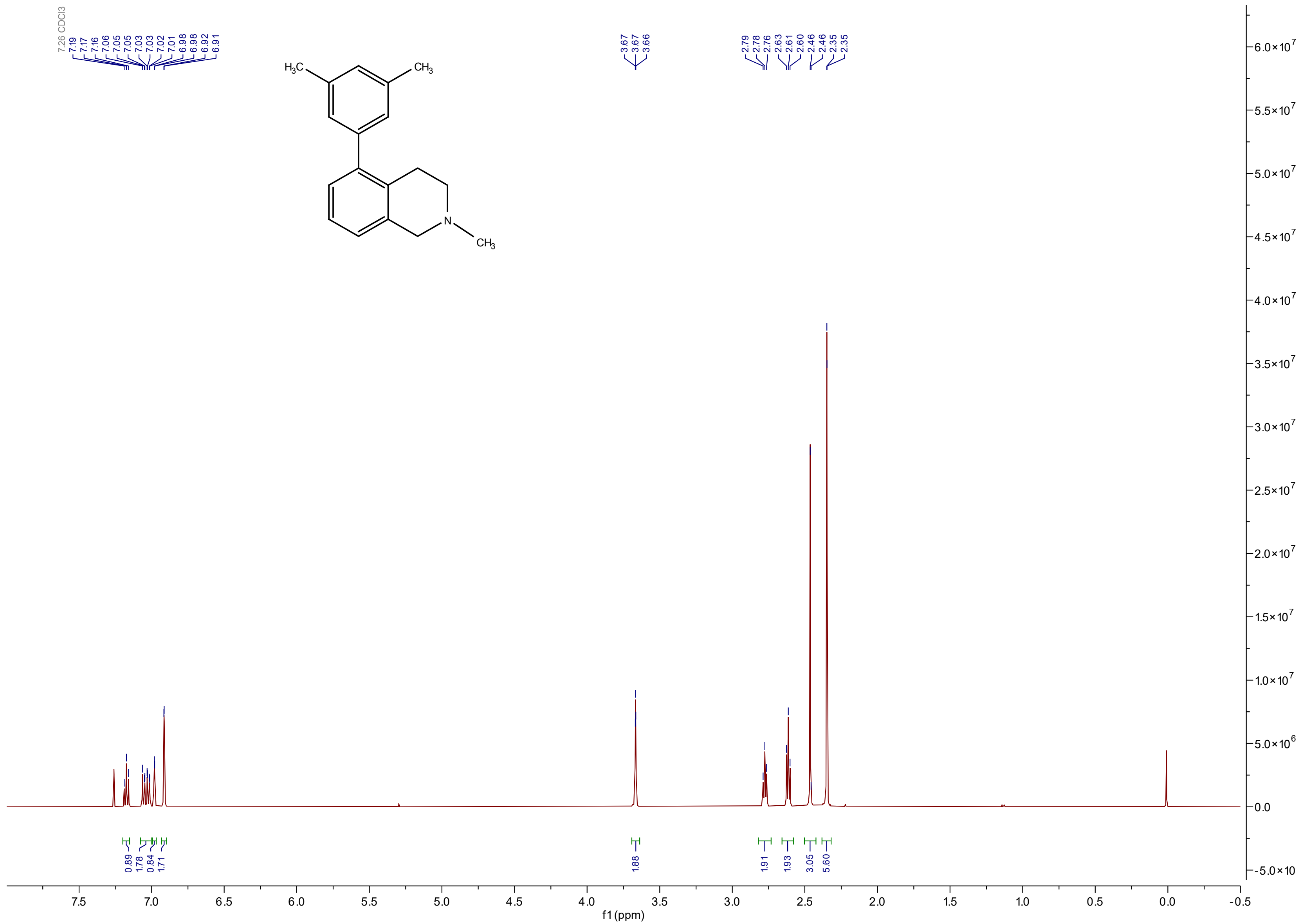

Fig. S97. ^{1}H NMR (500 MHz) of 2-methyl-5-(3,5-dimethylphenyl)-1,2,3,4-tetrahydroisoquinoline (**1o**).

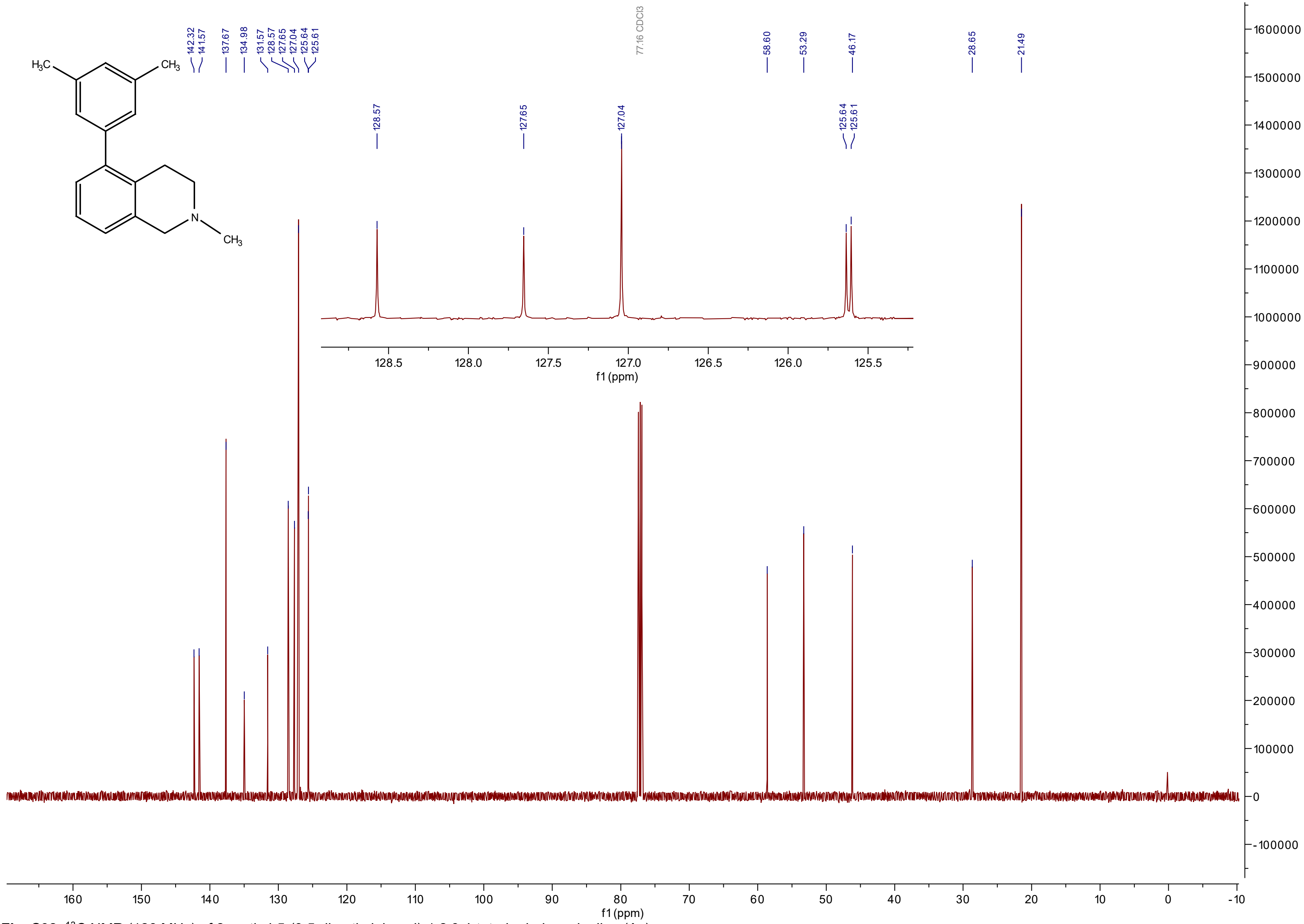

Fig. S98. ^{13}C NMR (126 MHz) of 2-methyl-5-(3,5-dimethylphenyl)-1,2,3,4-tetrahydroisoquinoline (**1o**).

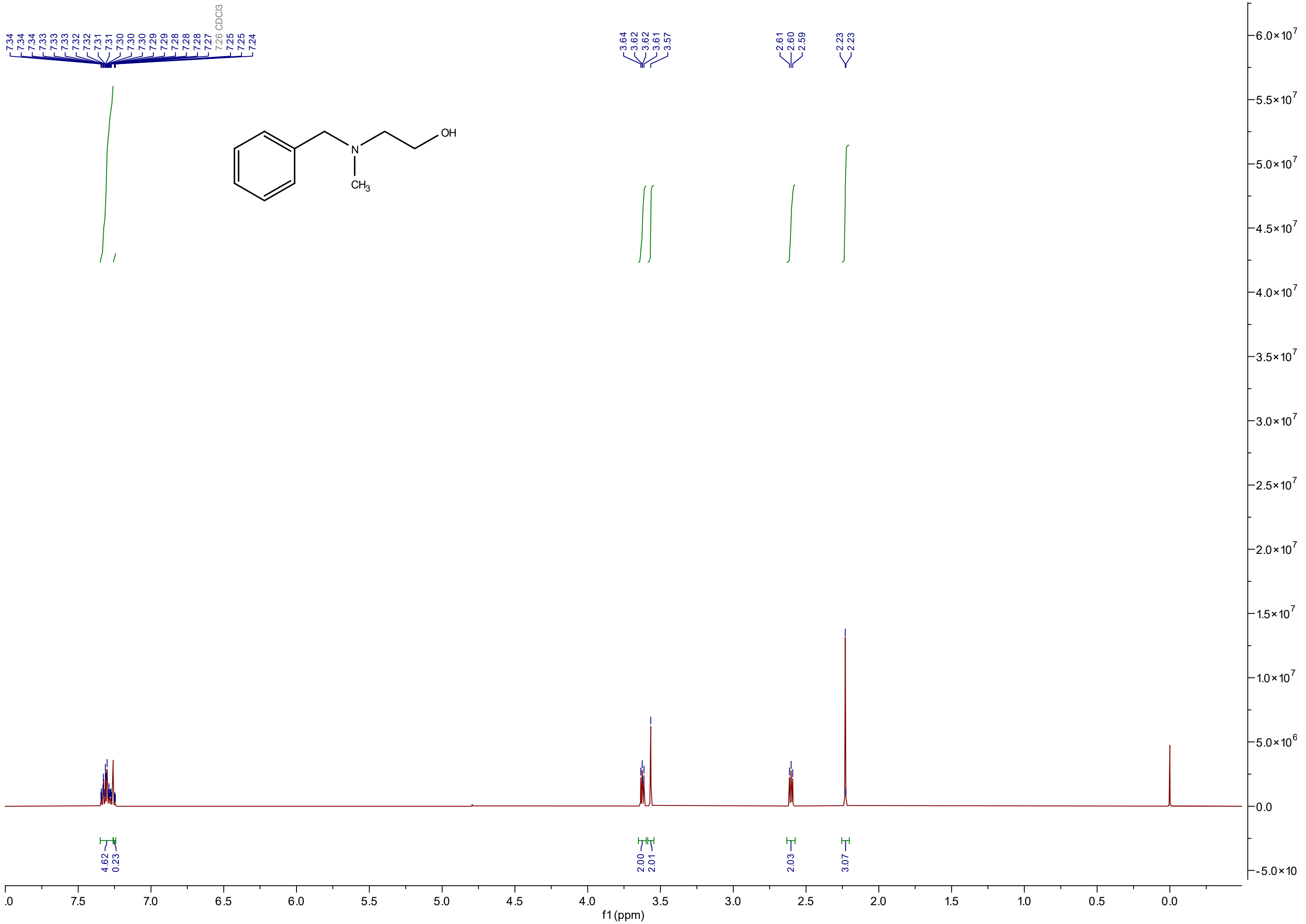

Fig. S99. ^{1}H NMR (500 MHz) of 2-(benzyl(methyl)amino)ethan-1-ol (**1p**).

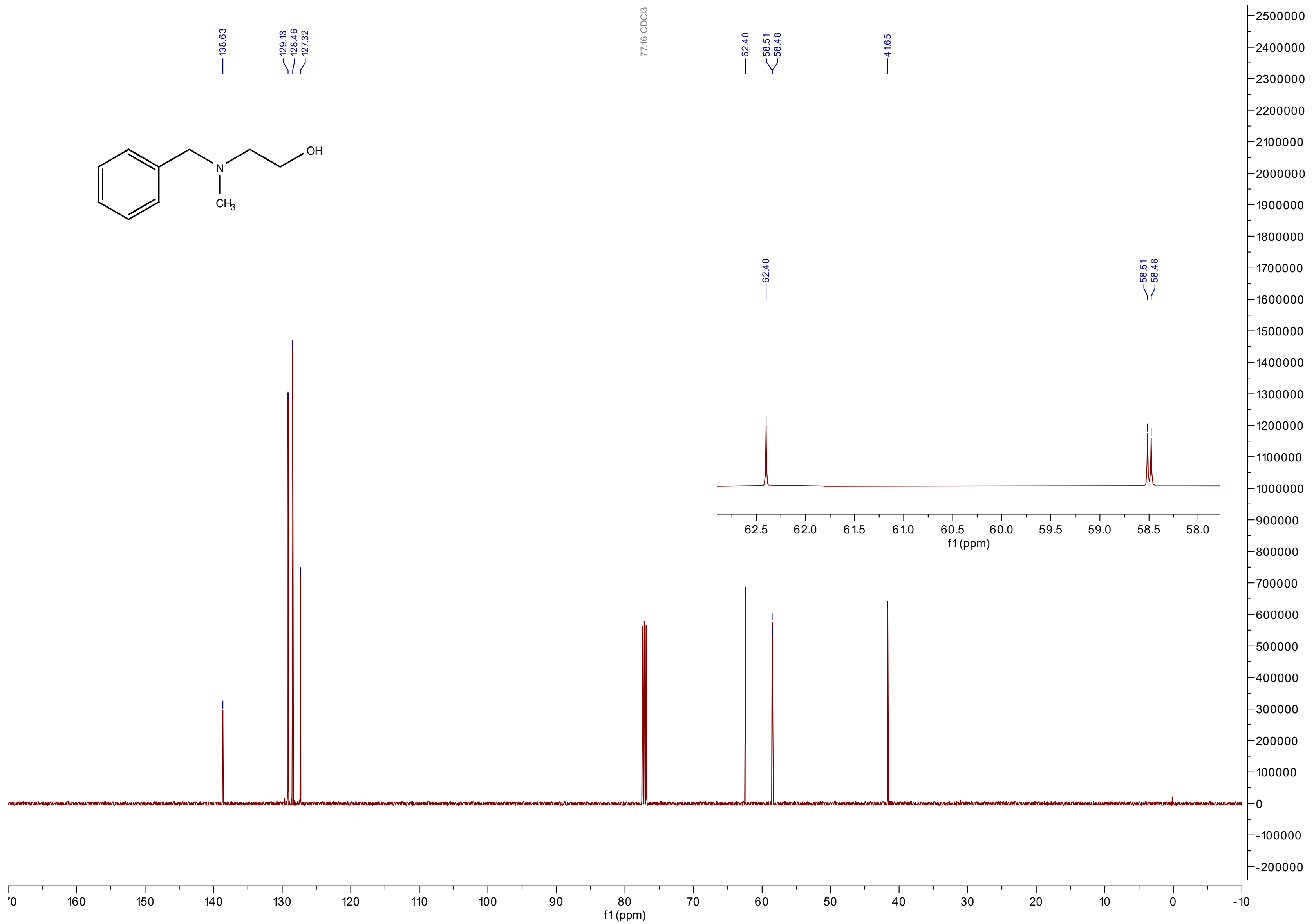

Fig. S100. ^{13}C NMR (126 MHz) of 2-(benzyl(methyl)amino)ethan-1-ol (**1p**).

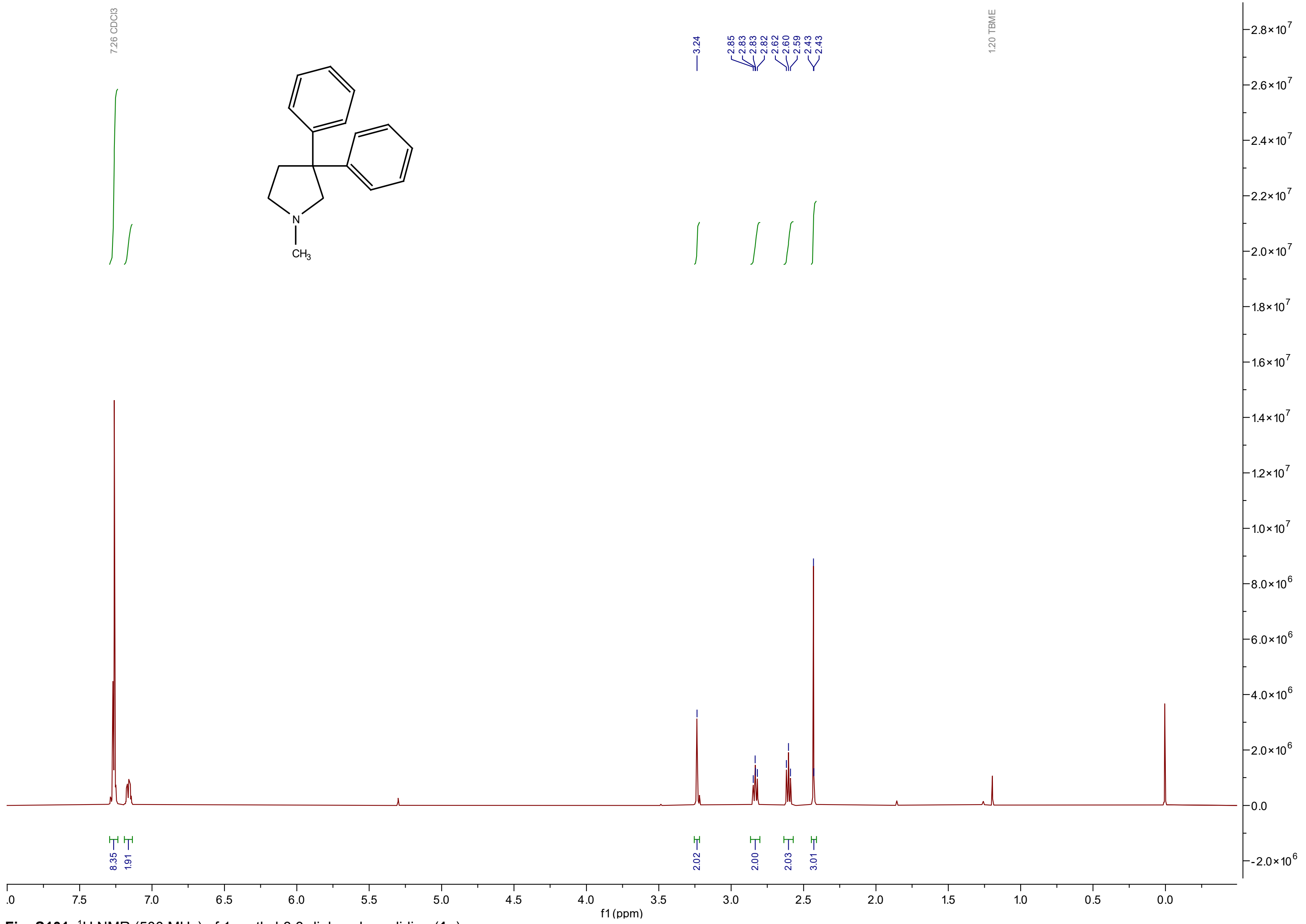

Fig. S101. ^{1}H NMR (500 MHz) of 1-methyl-3,3-diphenylpyrrolidine (**1q**).

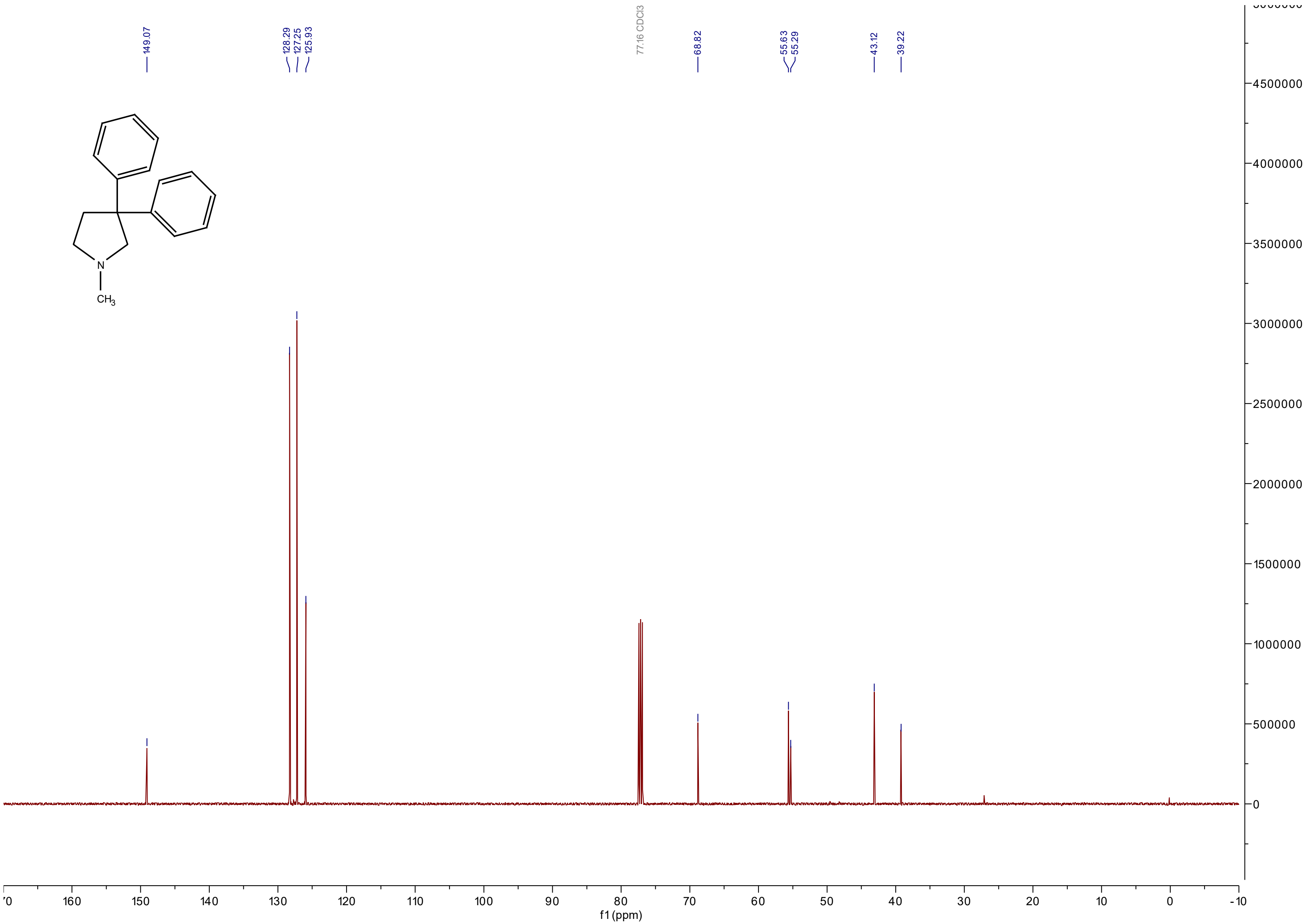

Fig. S102. ^{13}C NMR (126 MHz) of 1-methyl-3,3-diphenylpyrrolidine (**1q**).

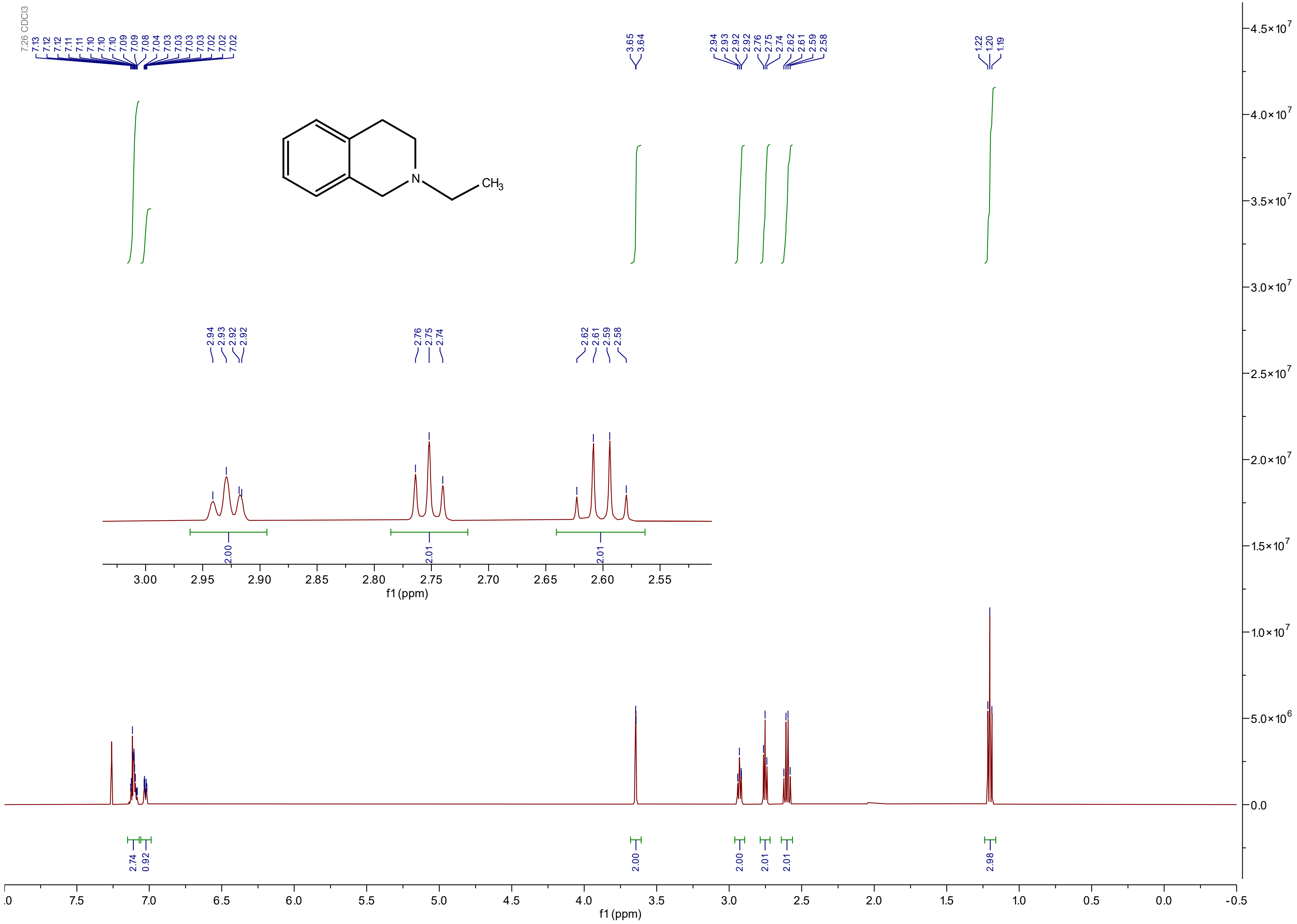

Fig. S103. ^{1}H NMR (500 MHz) of 2-ethyl-1,2,3,4-tetrahydroisoquinoline (**1r**).

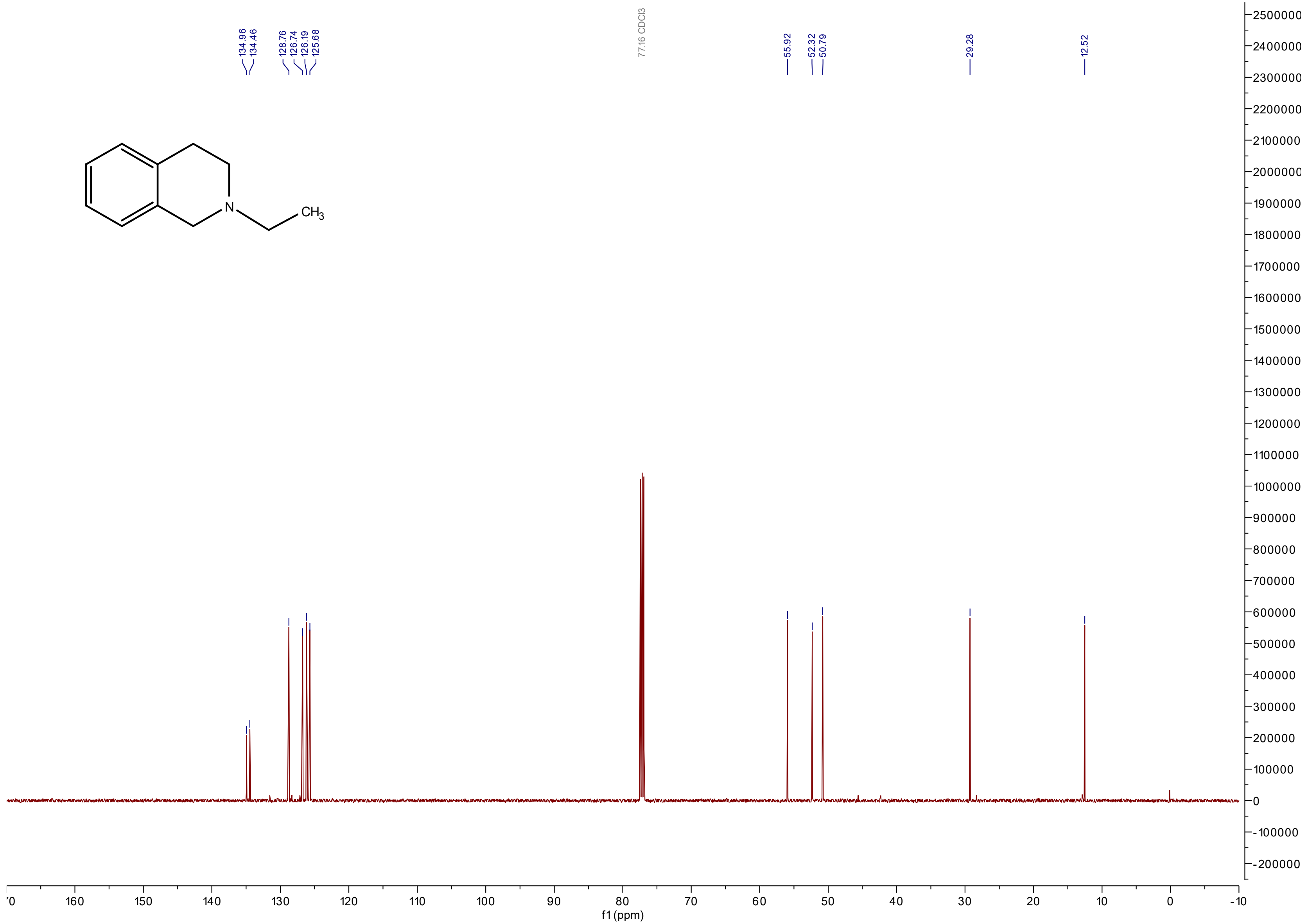

Fig. S104. ^{13}C NMR (126 MHz) of 2-ethyl-1,2,3,4-tetrahydroisoquinoline (**1r**).

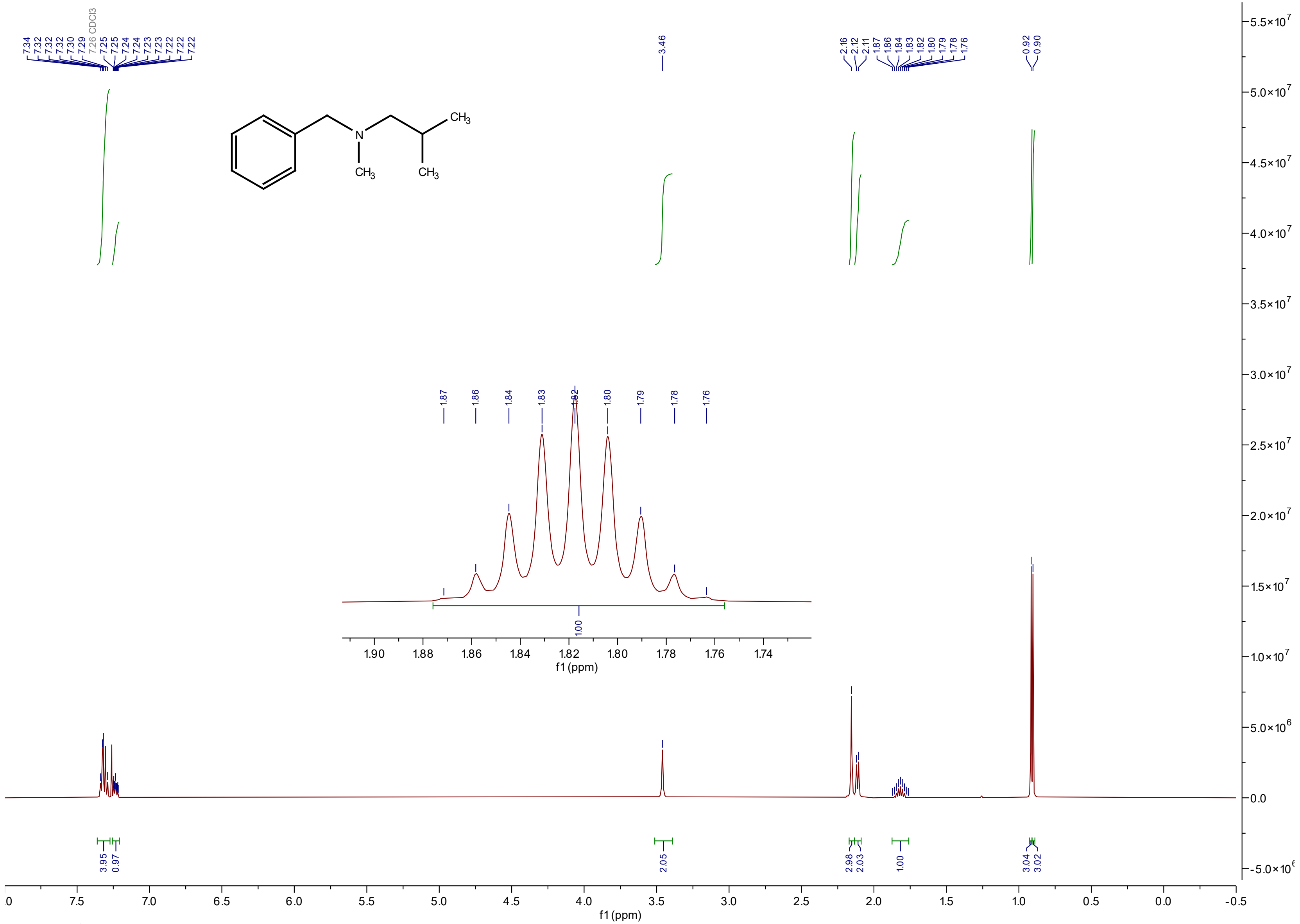

Fig. S105. ^{1}H NMR (500 MHz) of *N*-benzyl-*N*,2-dimethylpropan-1-amine (**1s**).

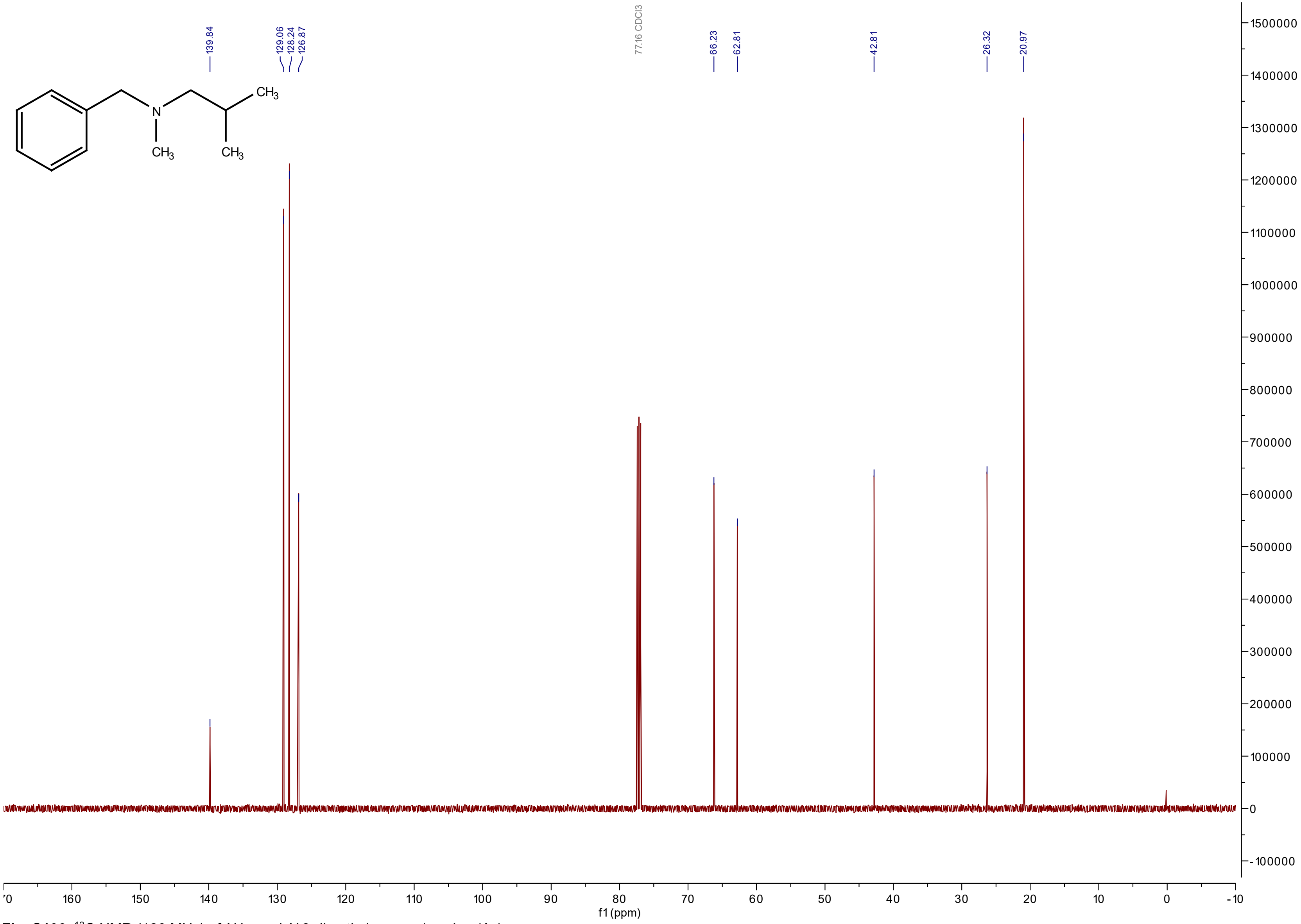

Fig. S106. ^{13}C NMR (126 MHz) of *N*-benzyl-*N*,2-dimethylpropan-1-amine (**1s**).

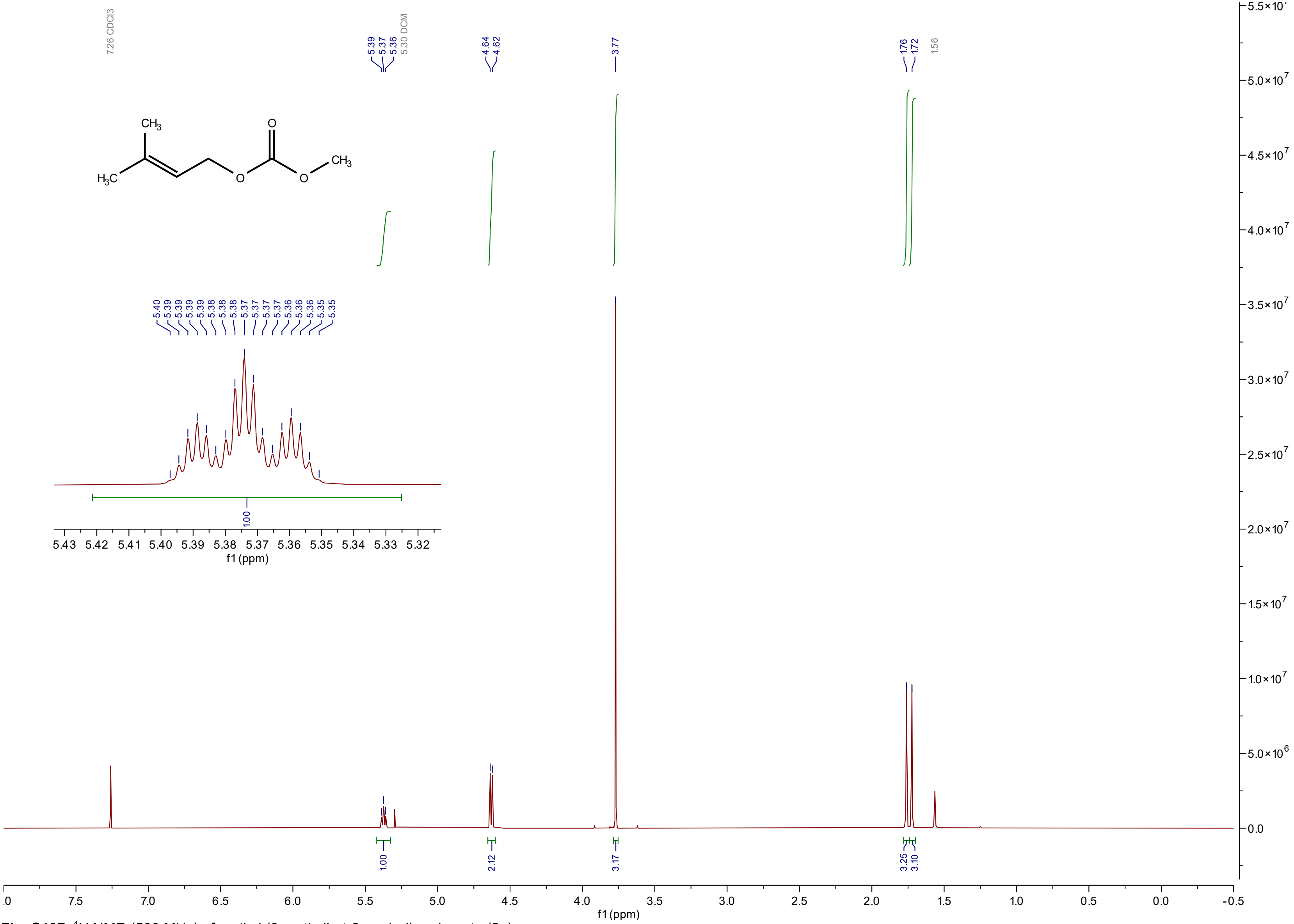

Fig. S107. ^{1}H NMR (500 MHz) of methyl (3-methylbut-2-en-1-yl) carbonate (**2a**).

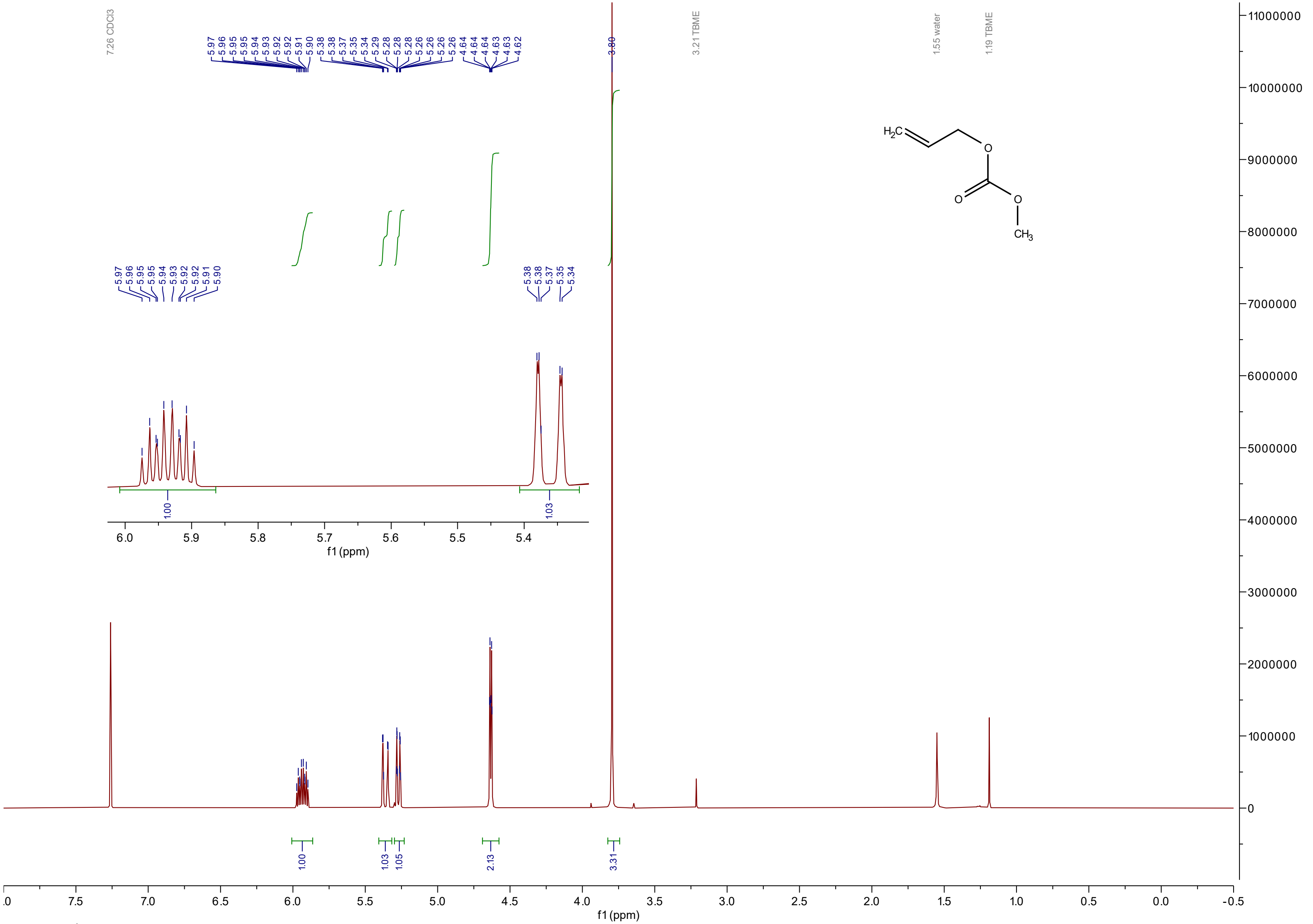

Fig. S108. ^{1}H NMR (500 MHz) of allyl methyl carbonate (**2b**).

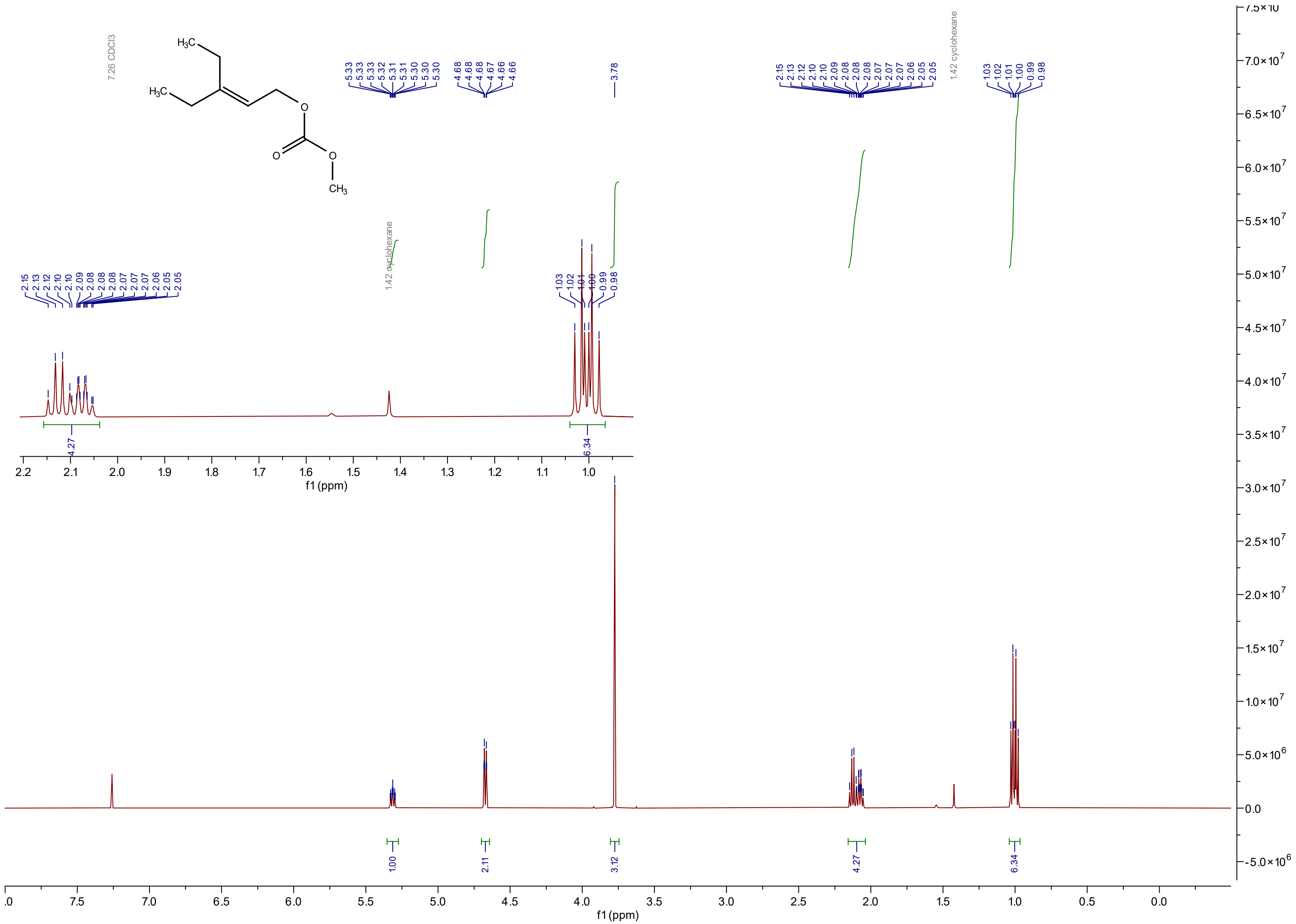

Fig. S109. ^{1}H NMR (500 MHz) of 3-ethylpent-2-en-1-yl methyl carbonate (**2c**).

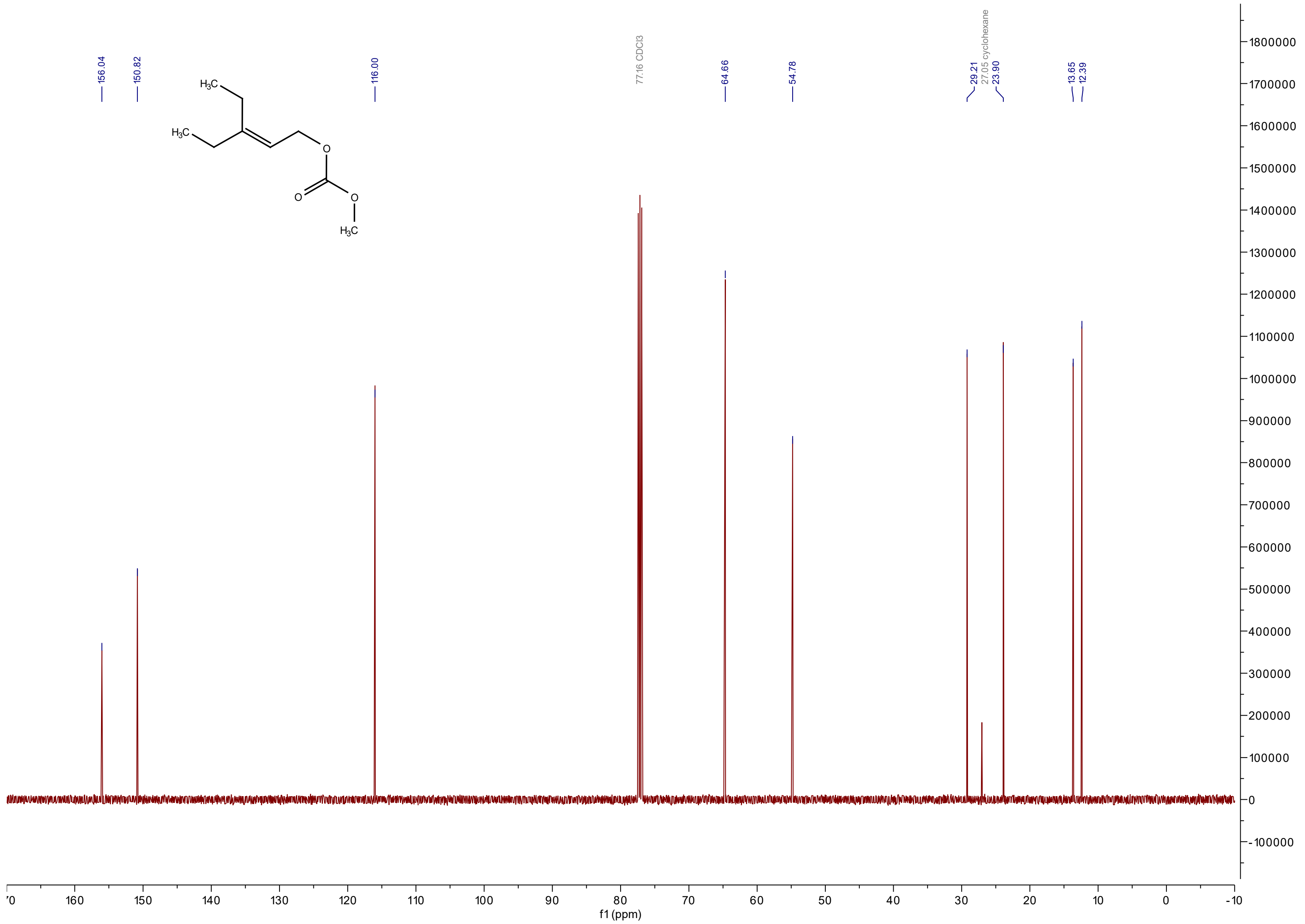

Fig. S110. ^{13}C NMR (126 MHz) of 3-ethylpent-2-en-1-yl methyl carbonate (**2c**).

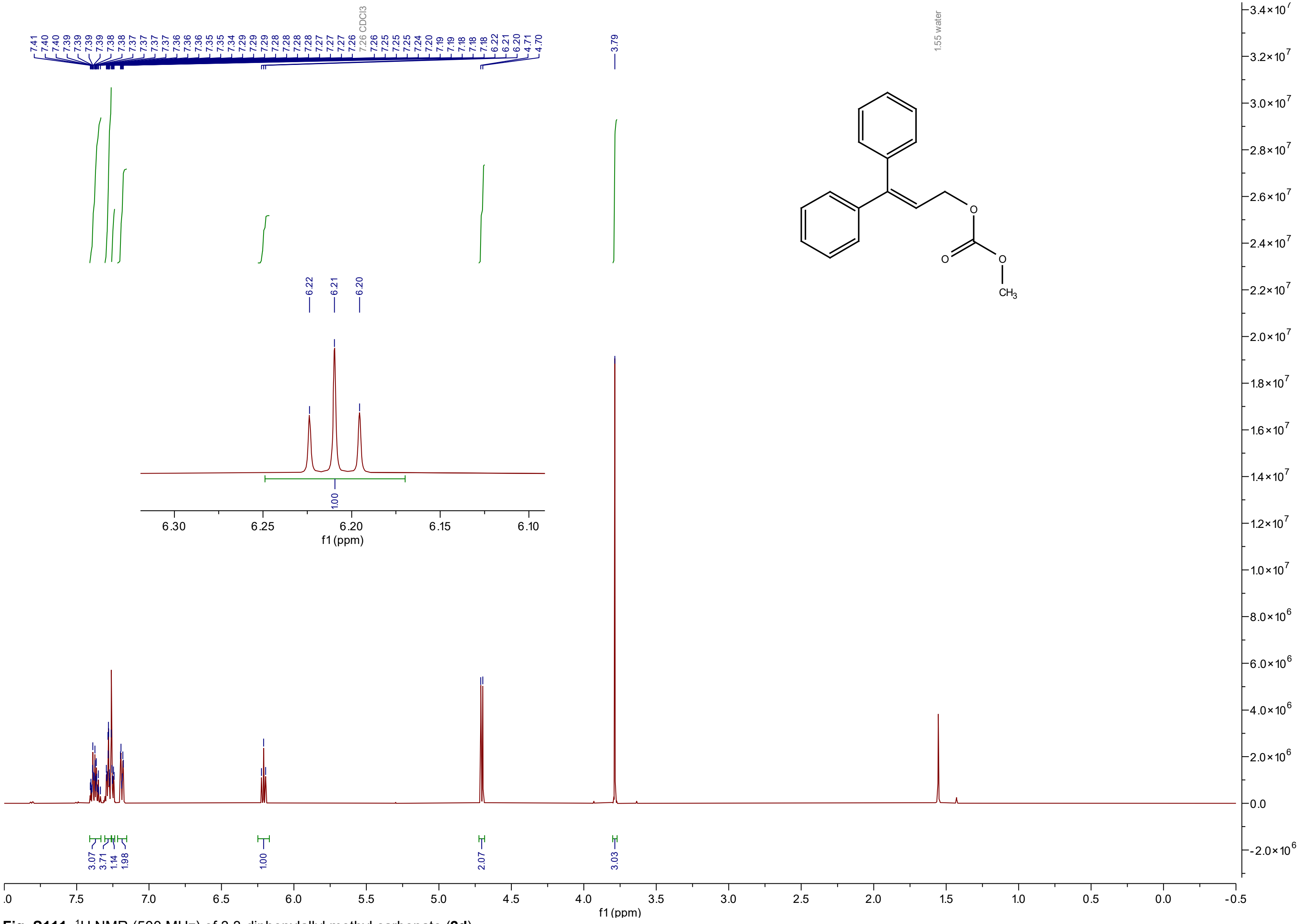

Fig. S111. ^{1}H NMR (500 MHz) of 3,3-diphenylallyl methyl carbonate (**2d**).

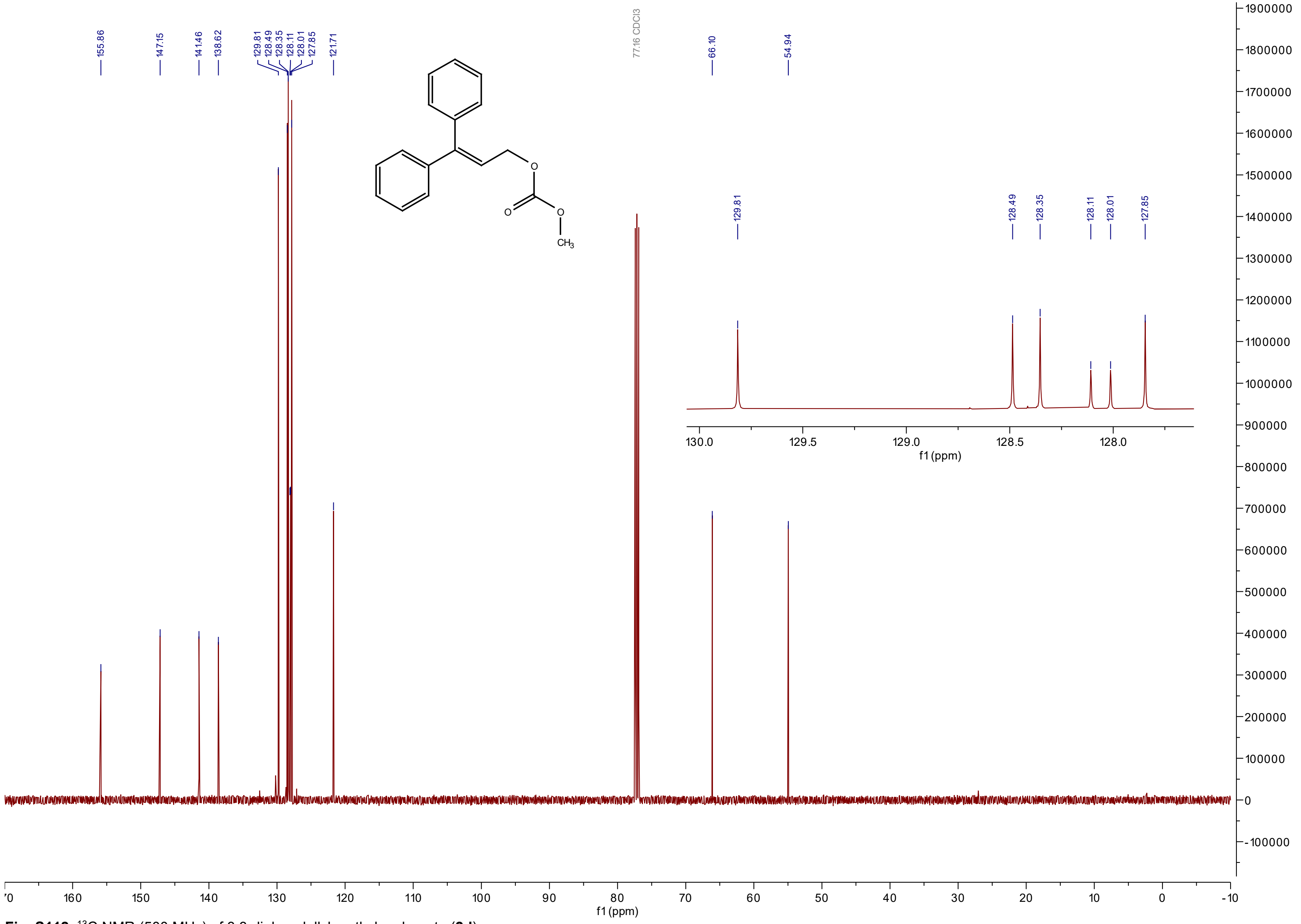

Fig. S112. ^{13}C NMR (500 MHz) of 3,3-diphenylallyl methyl carbonate (**2d**).

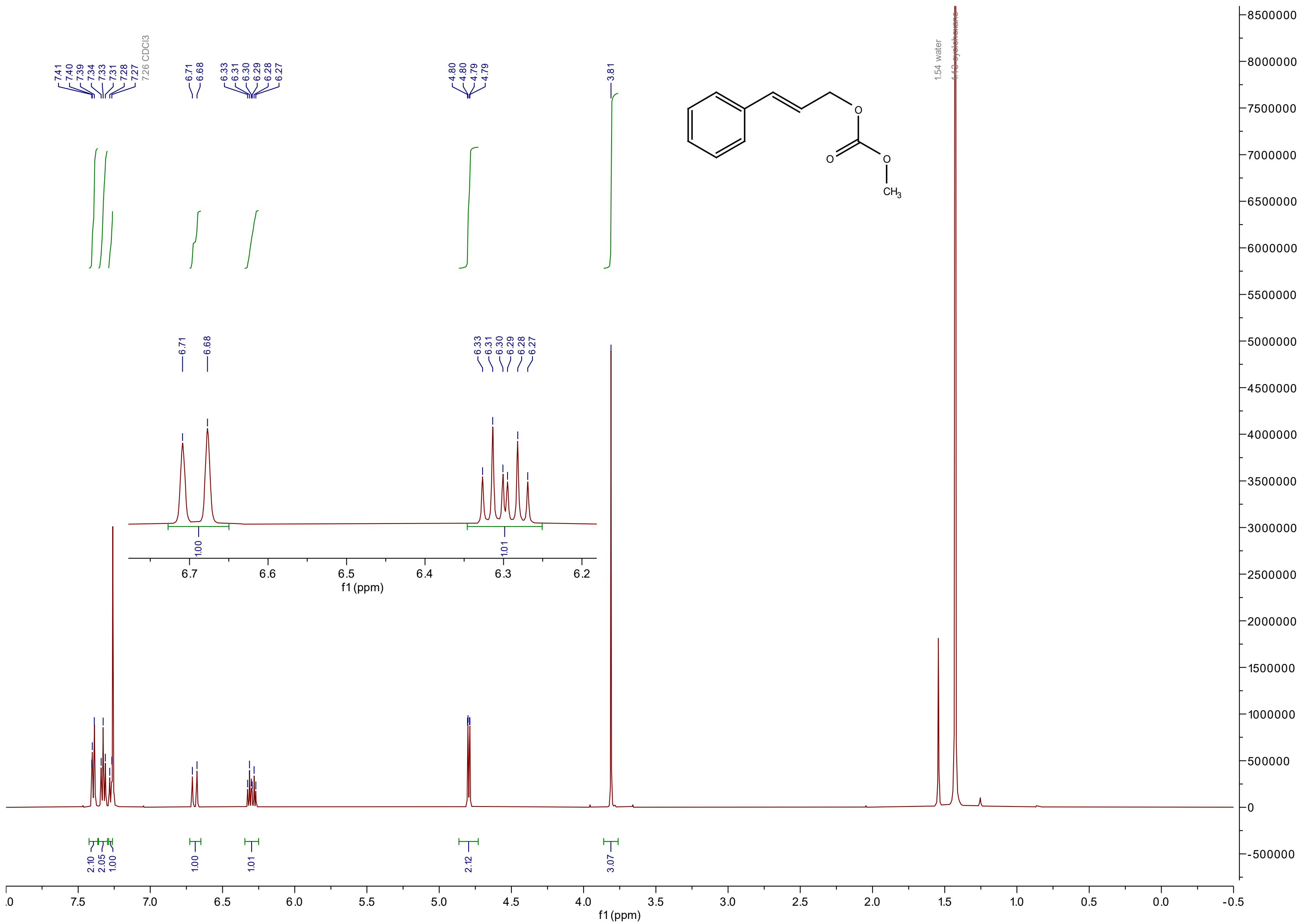

Fig. S113. ^{1}H NMR (500 MHz) of cinnamyl methyl carbonate (**2e**).

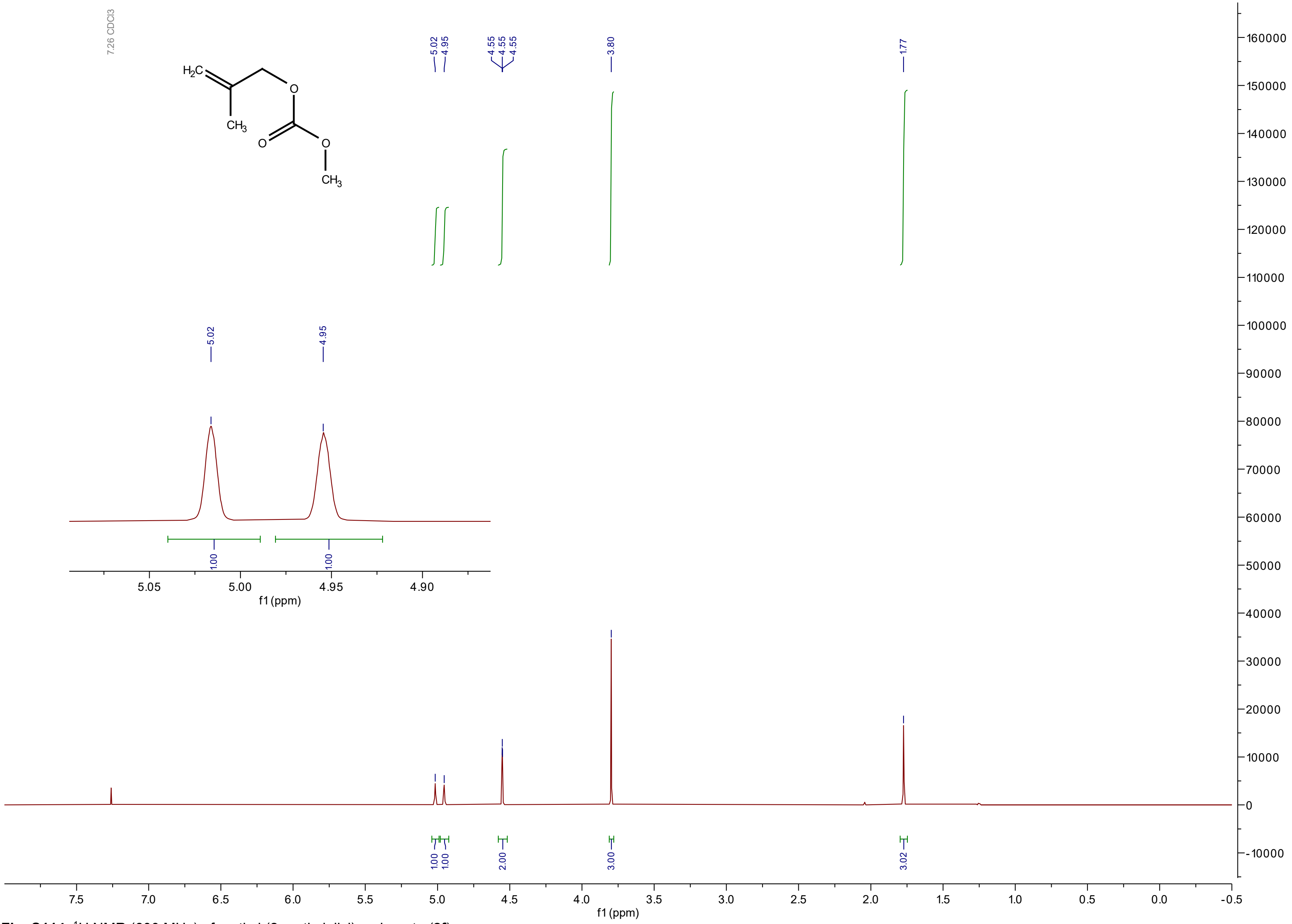

Fig. S114. ^{1}H NMR (600 MHz) of methyl (2-methylallyl) carbonate (**2f**).

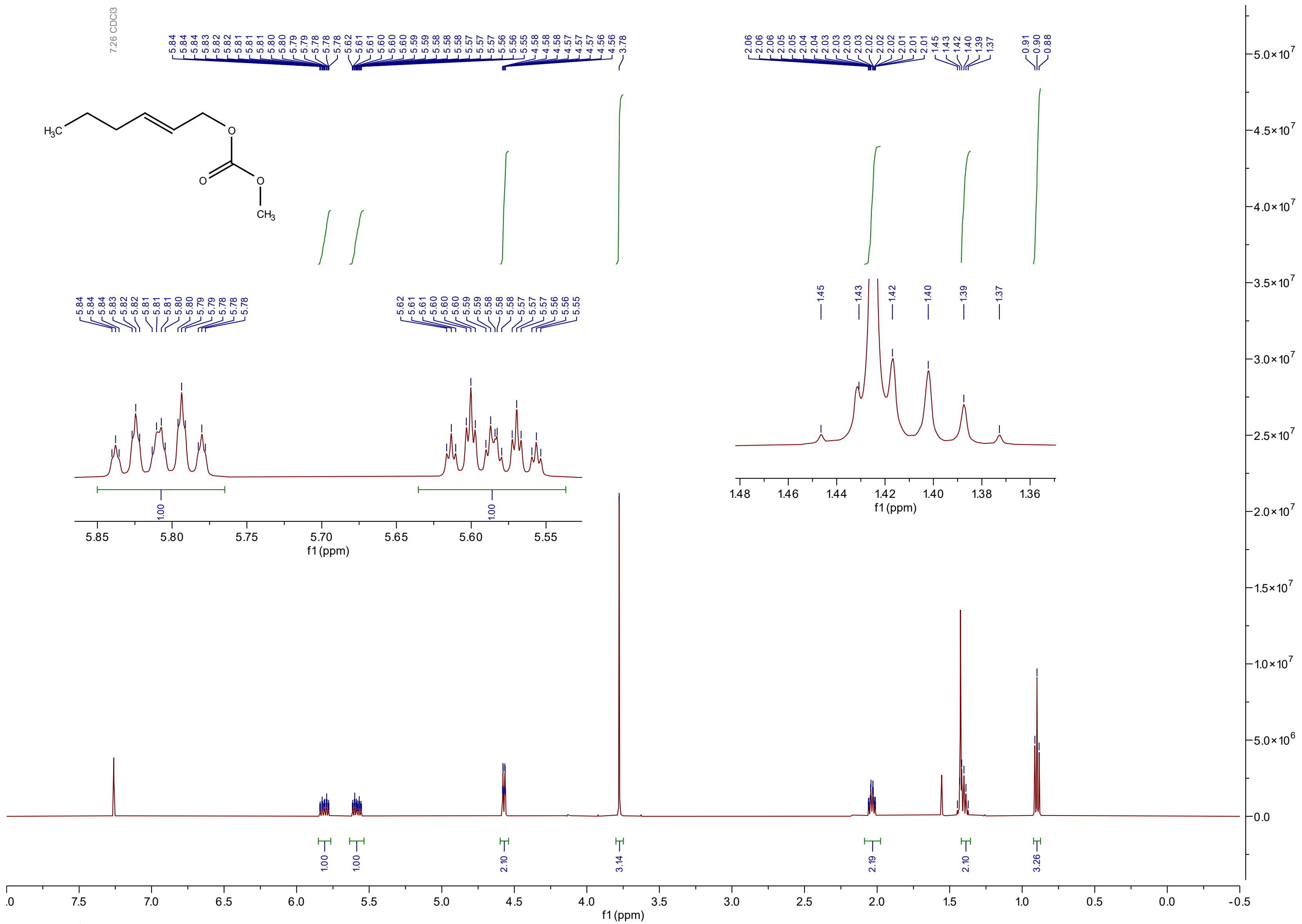

Fig. S115. ^{1}H NMR (500 MHz) of (E)-hex-2-en-1-yl methyl carbonate (**2g**).

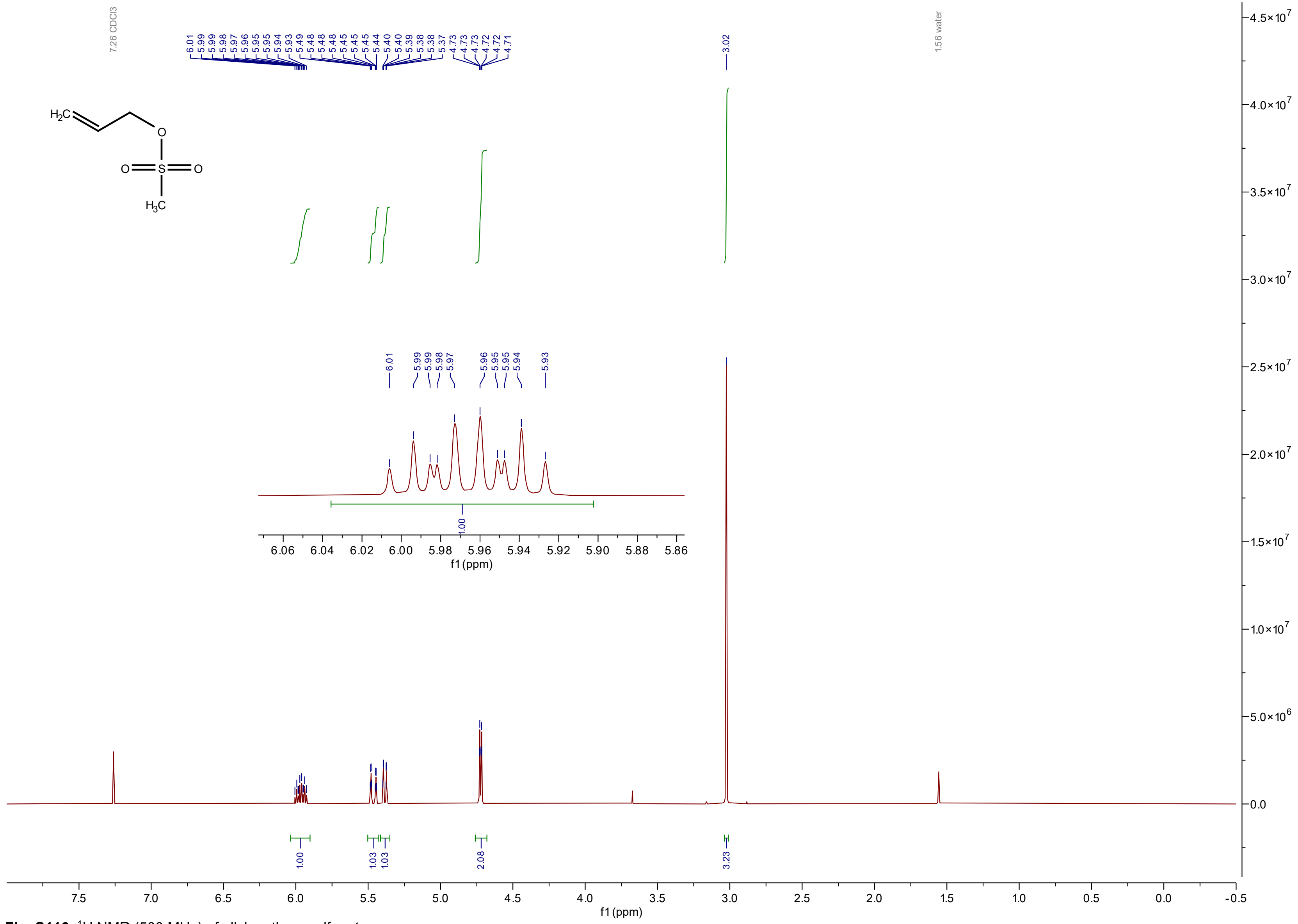

Fig. S116. ^{1}H NMR (500 MHz) of allyl methanesulfonate.

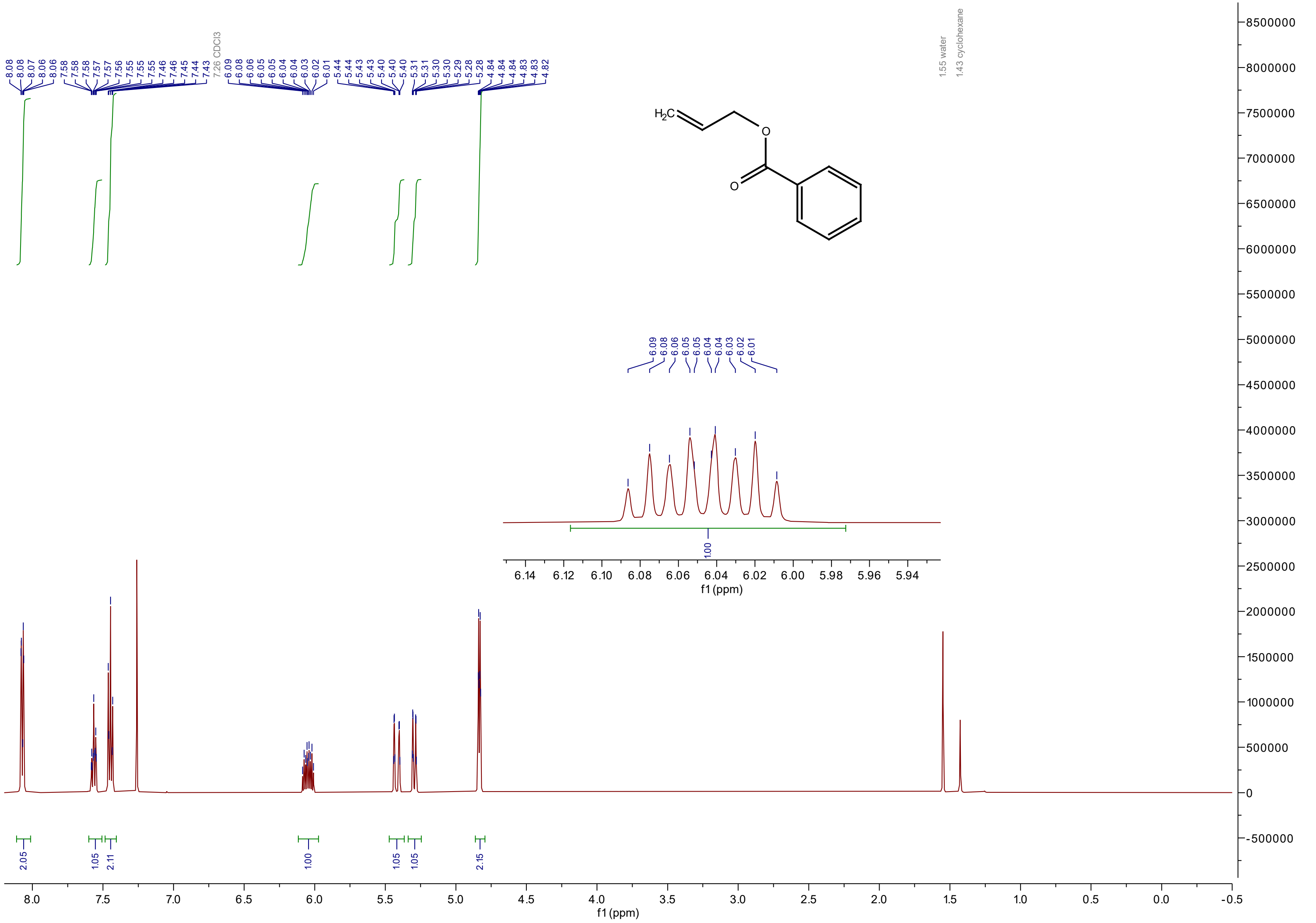

Fig. S117. ^{1}H NMR (500 MHz) of allyl benzoate.

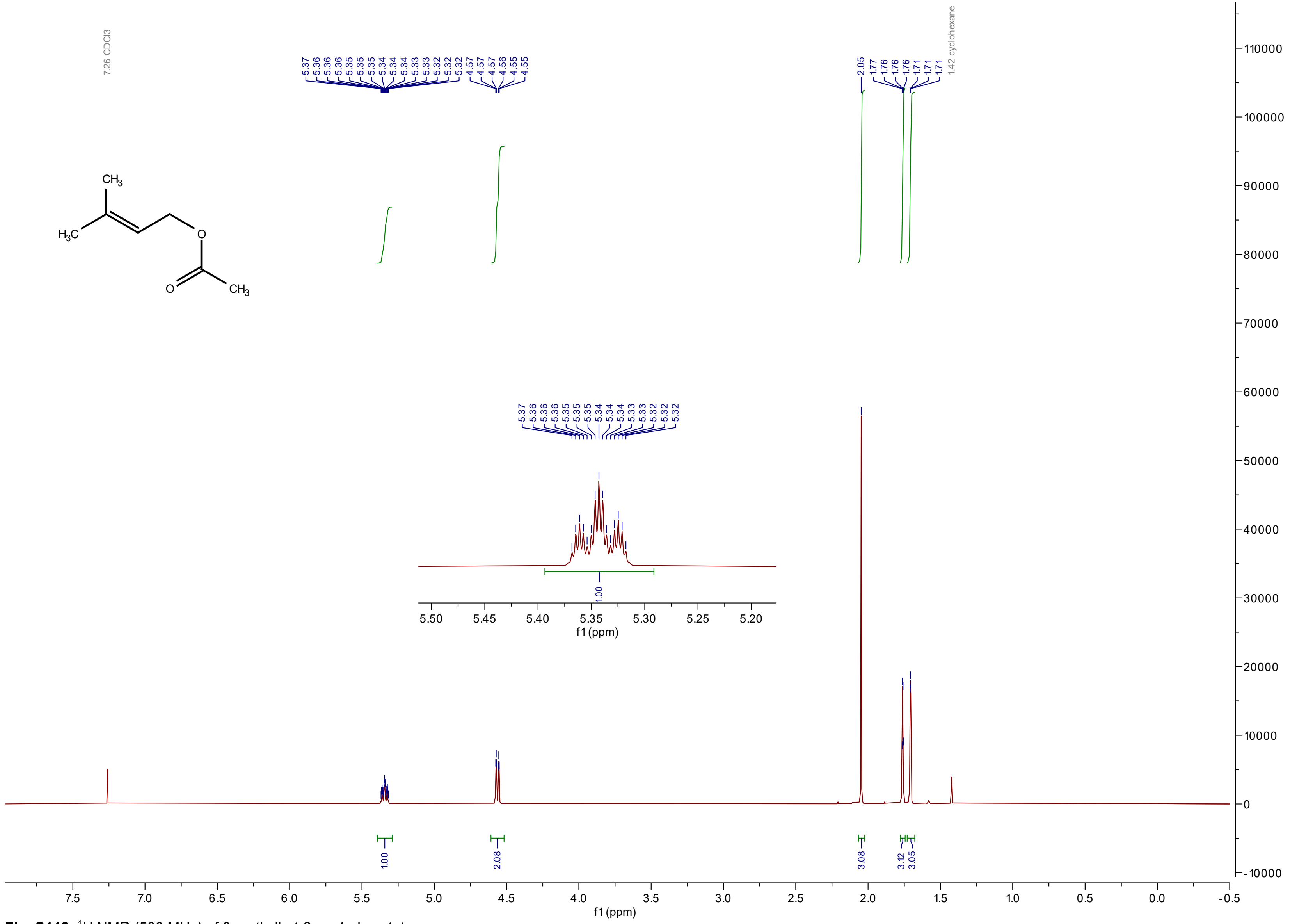

Fig. S118. ^{1}H NMR (500 MHz) of 3-methylbut-2-en-1-yl acetate.

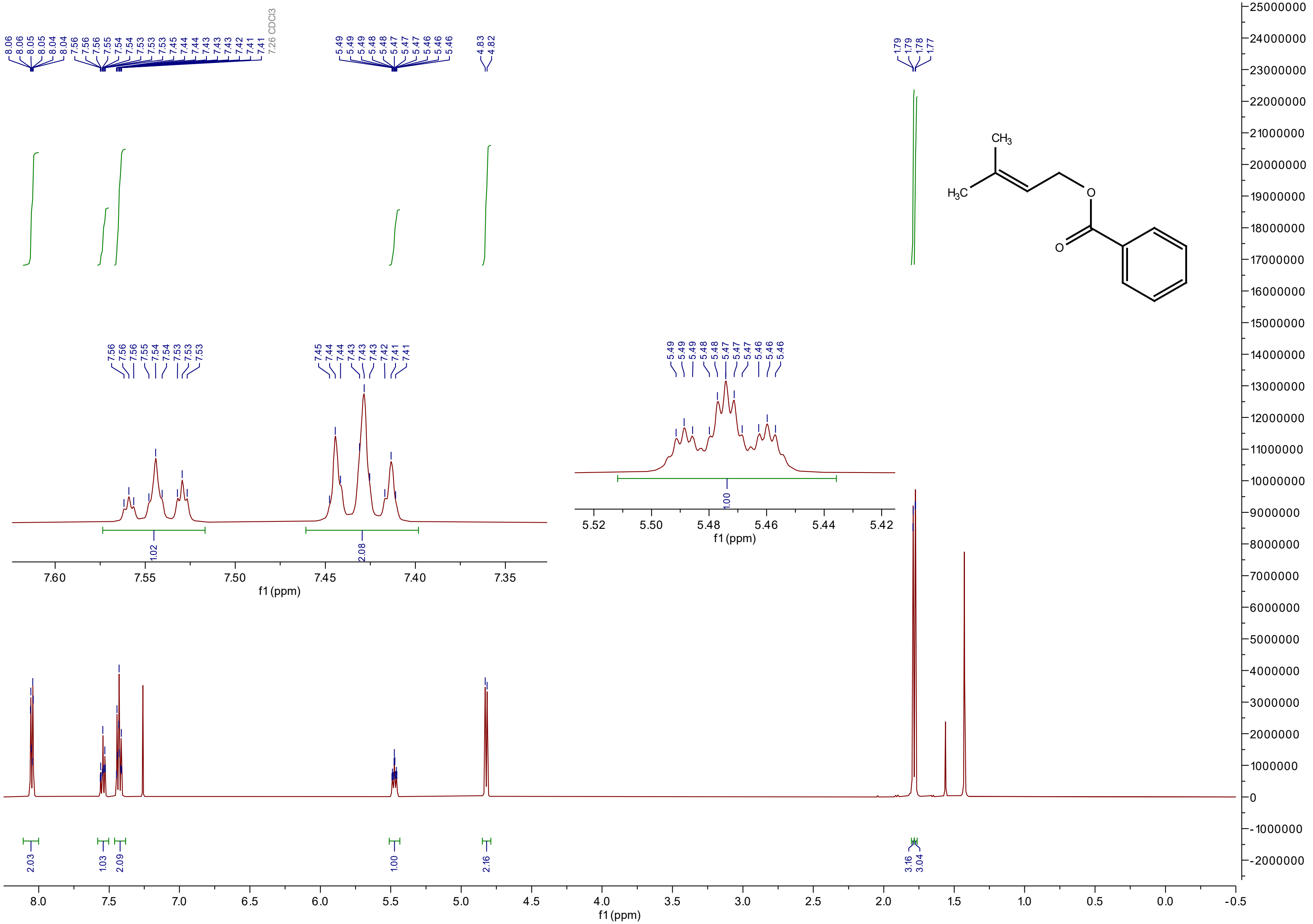

Fig. S119. ^{1}H NMR (500 MHz) of 3-methylbut-2-en-1-yl benzoate.

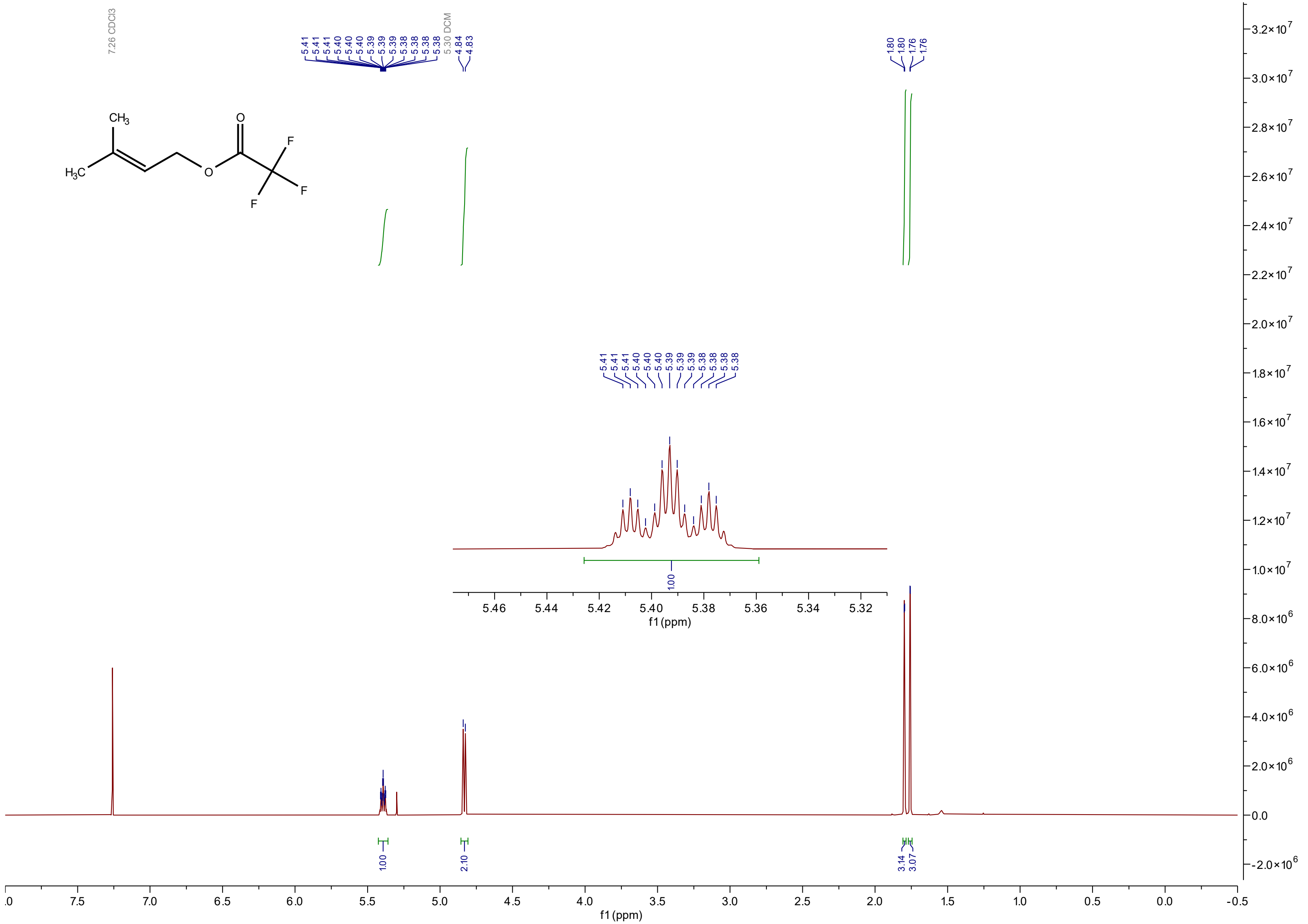

Fig. S120. ^{1}H NMR (500 MHz) of 3-methylbut-2-en-1-yl 2,2,2-trifluoroacetate.

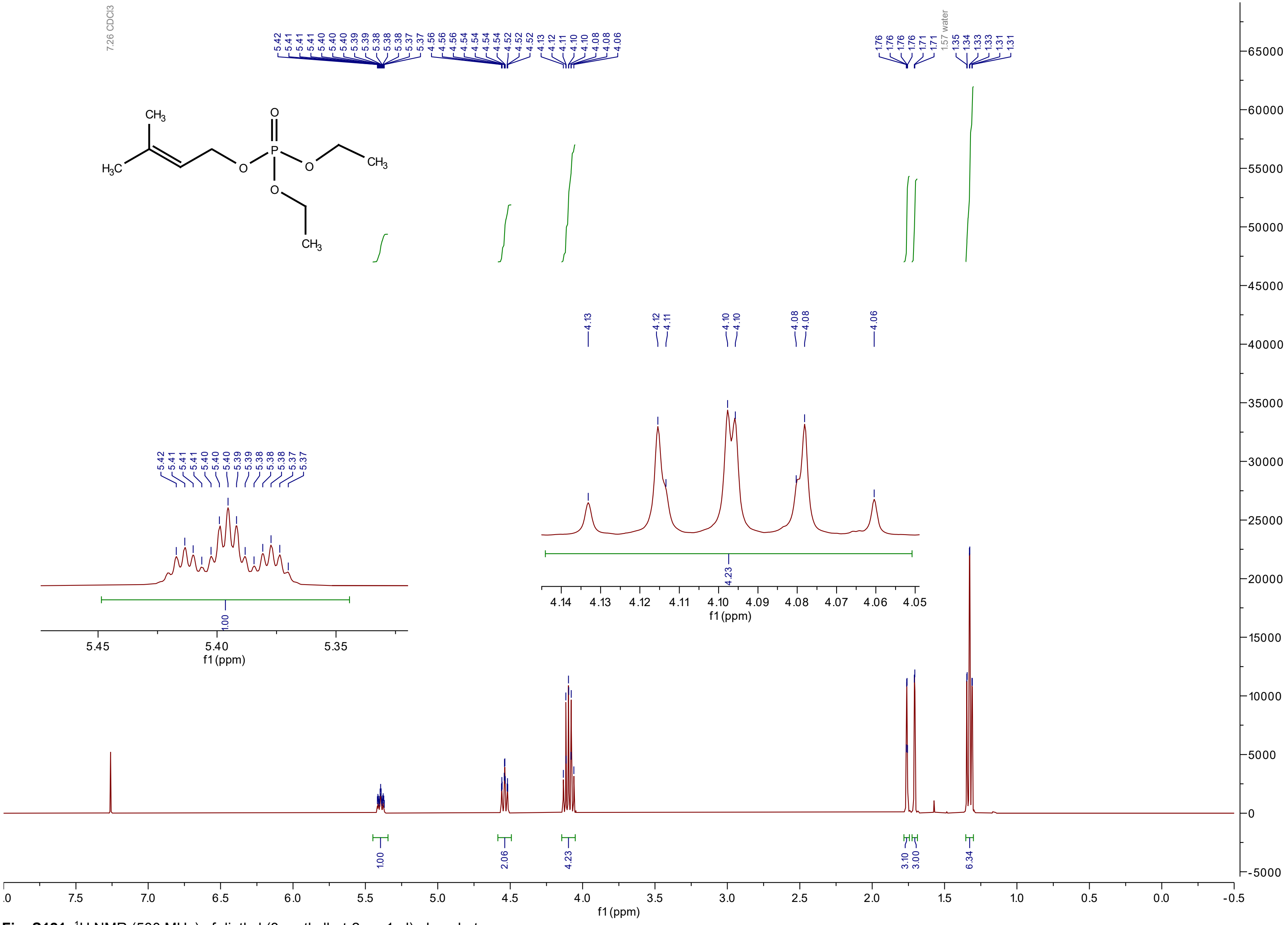

Fig. S121. ^{1}H NMR (500 MHz) of diethyl (3-methylbut-2-en-1-yl) phosphate.

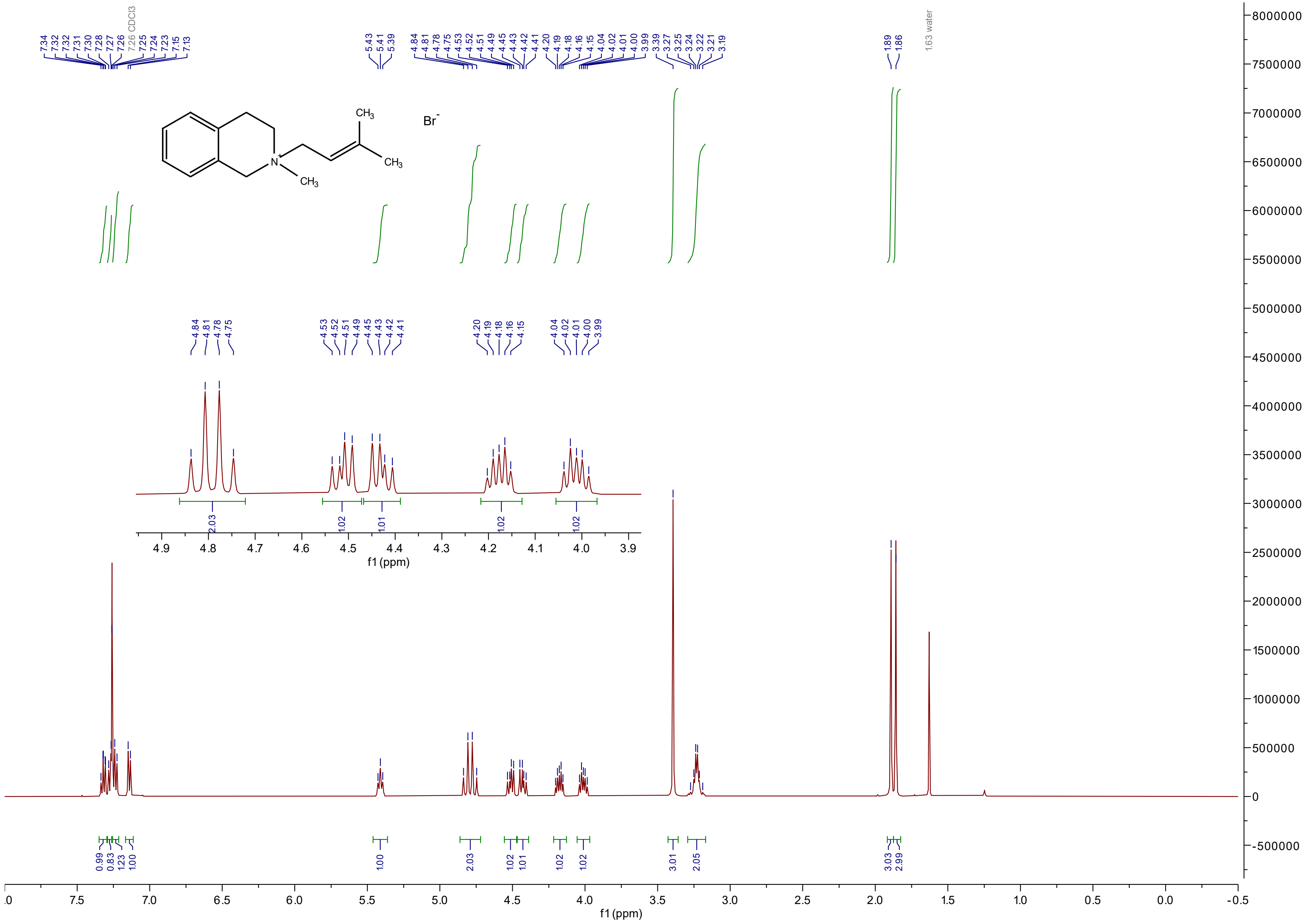

Fig. S122. ^{1}H NMR (500 MHz) of 2-methyl-2-(3-methylbut-2-en-1-yl)-1,2,3,4-tetrahydroisoquinolin-2-ium bromide ([**3aa**]Br).

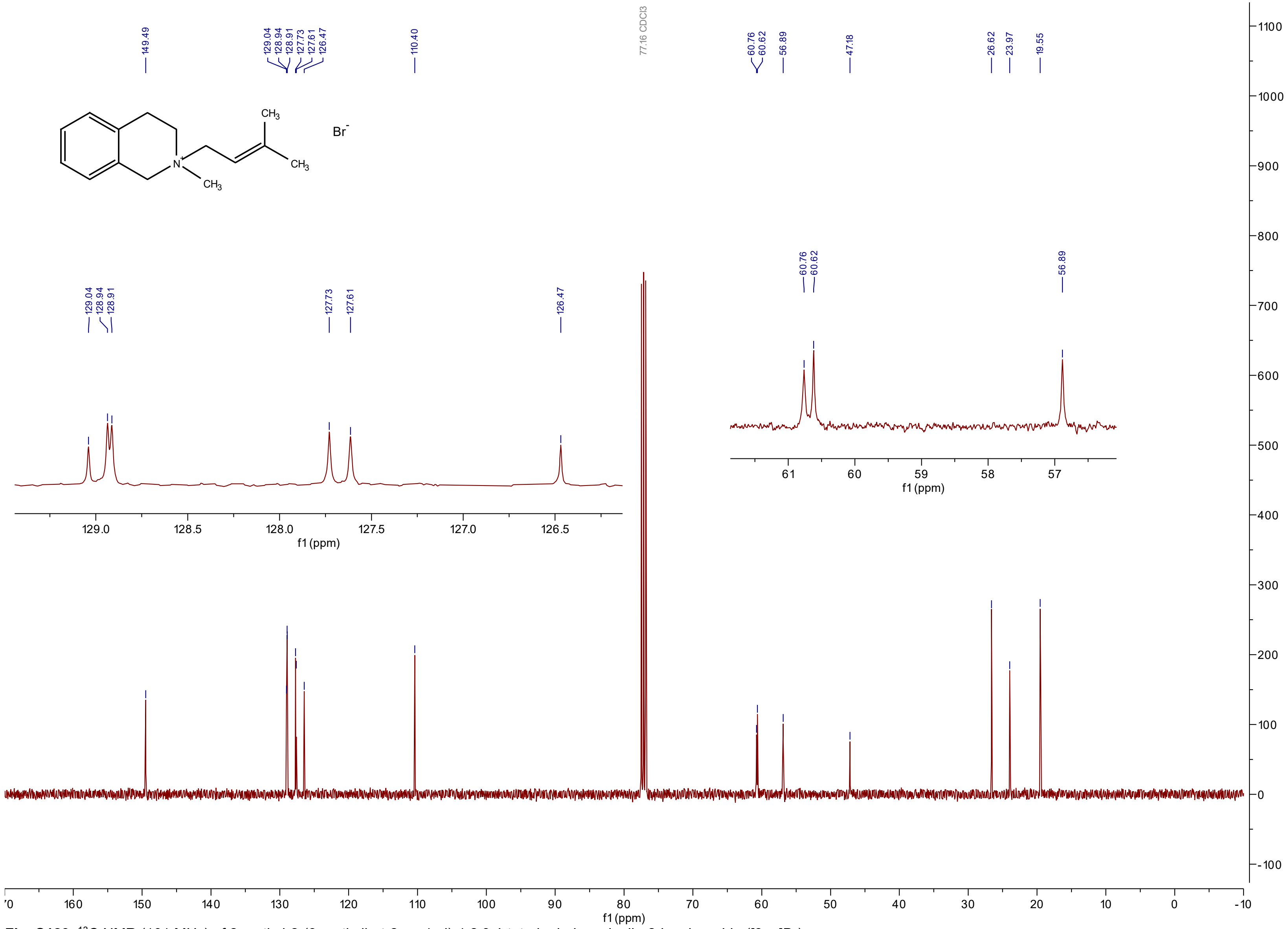

Fig. S123. ^{13}C NMR (101 MHz) of 2-methyl-2-(3-methylbut-2-en-1-yl)-1,2,3,4-tetrahydroisoquinolin-2-ium bromide ([**3aa**]Br).

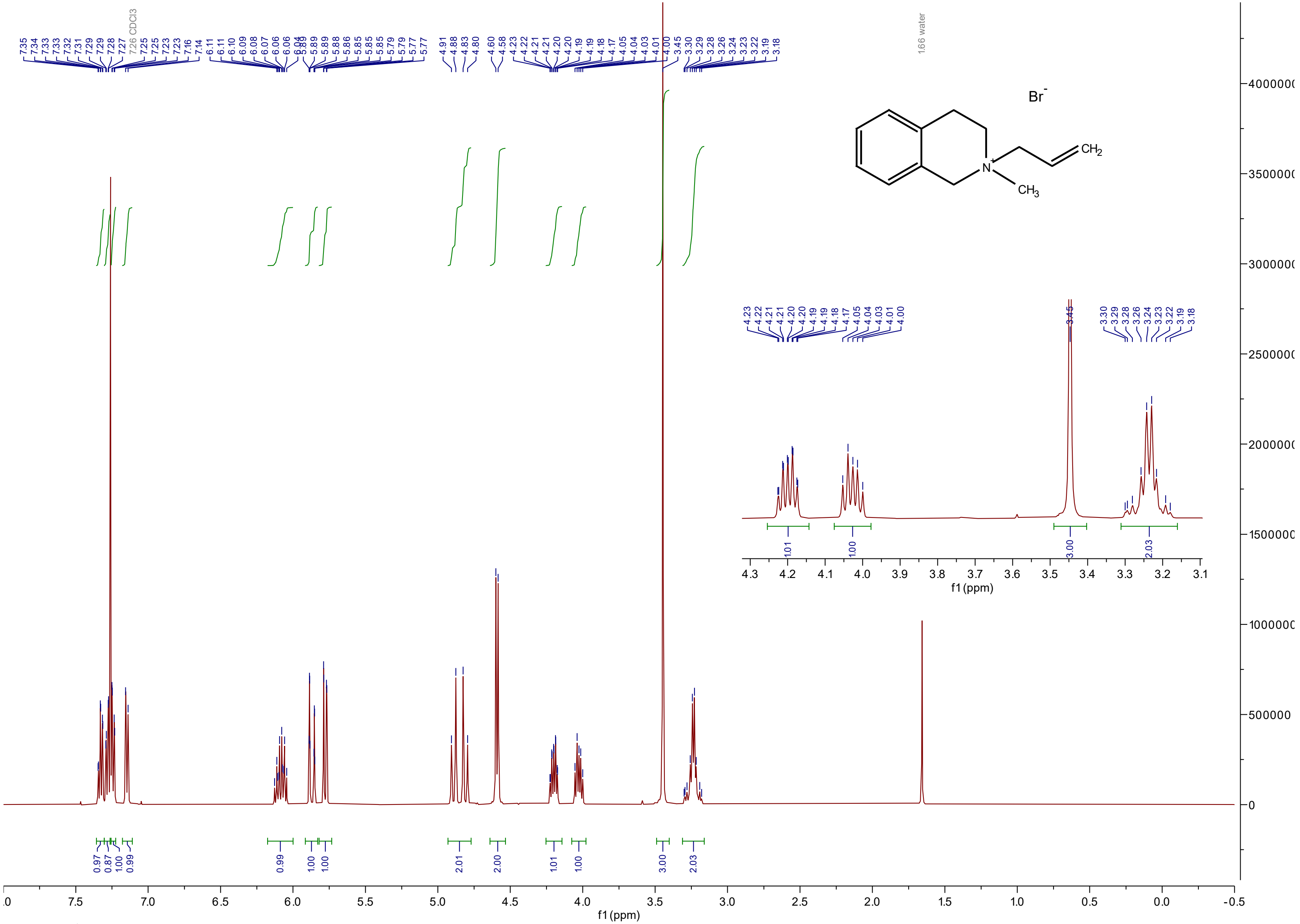

Fig. S124. ^{1}H NMR (500 MHz) of 2-allyl-2-methyl-1,2,3,4-tetrahydroisoquinolin-2-ium bromide ([**3ab**]Br).

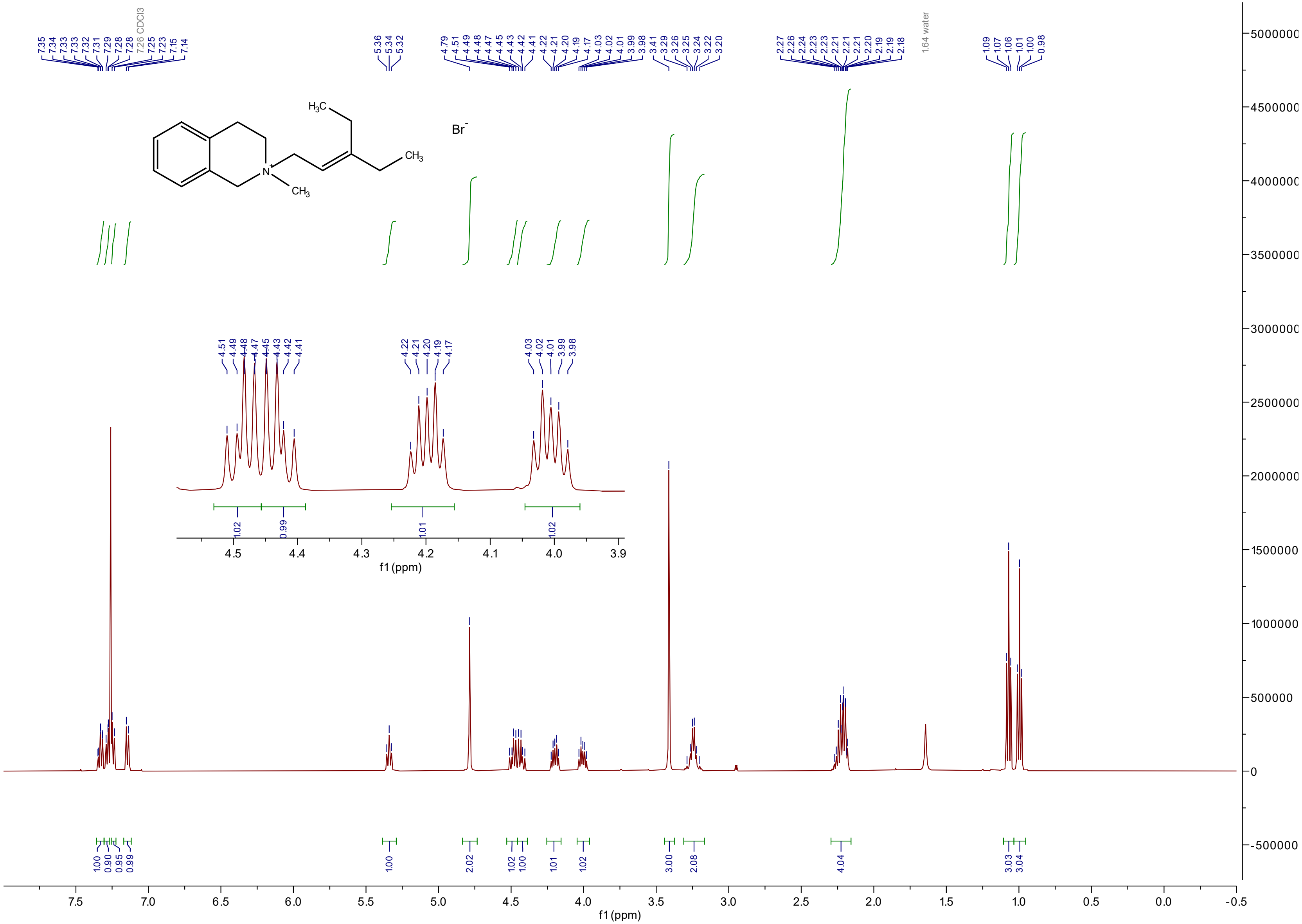

Fig. S125. ^{1}H NMR (500 MHz) of 2-(3-ethylpent-2-en-1-yl)-2-methyl-1,2,3,4-tetrahydroisoquinolin-2-ium bromide ([**3ac**]Br).

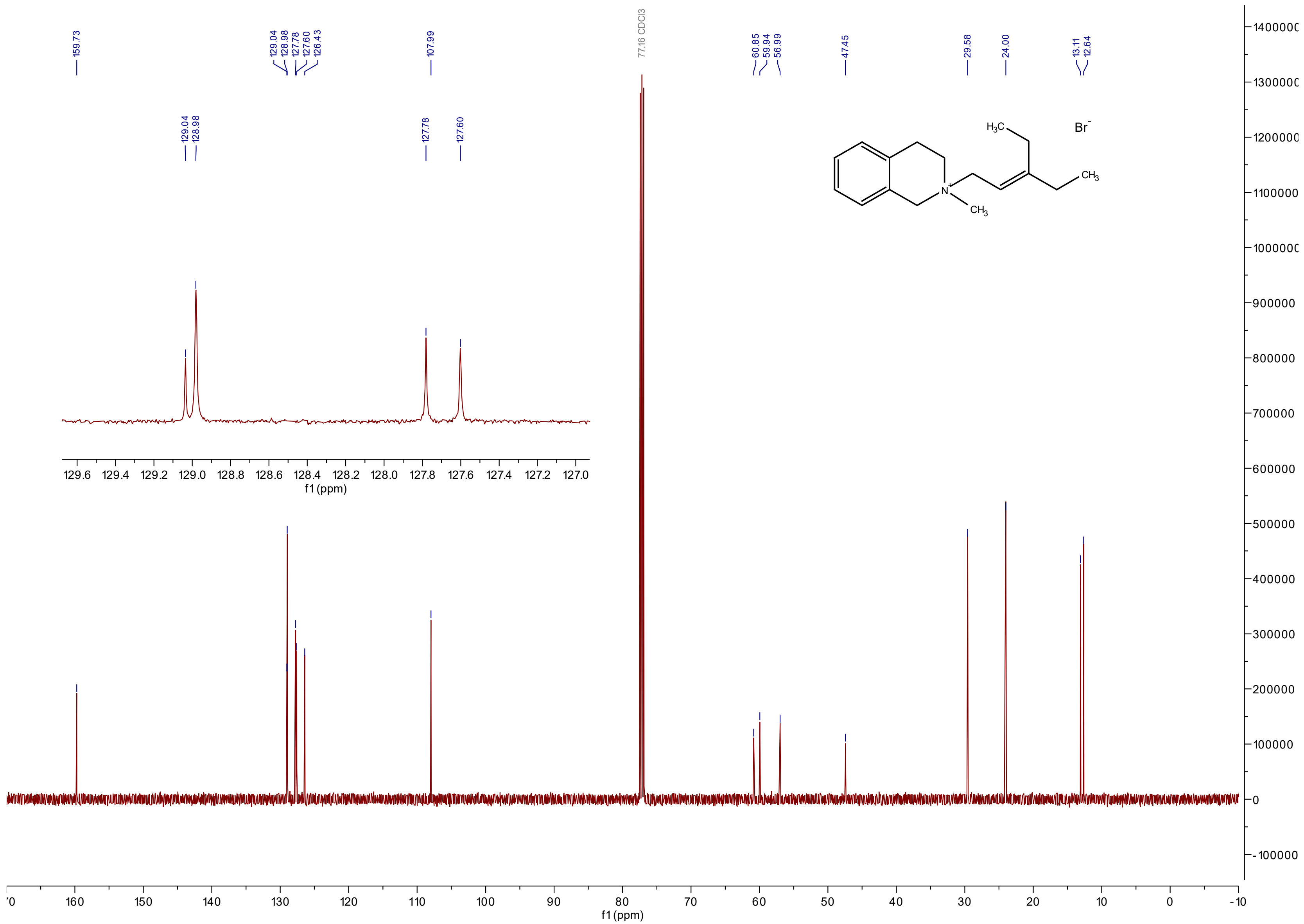

Fig. S126. ^{13}C NMR (126 MHz) of 2-(3-ethylpent-2-en-1-yl)-2-methyl-1,2,3,4-tetrahydroisoquinolin-2-ium bromide ([**3ac**]Br).

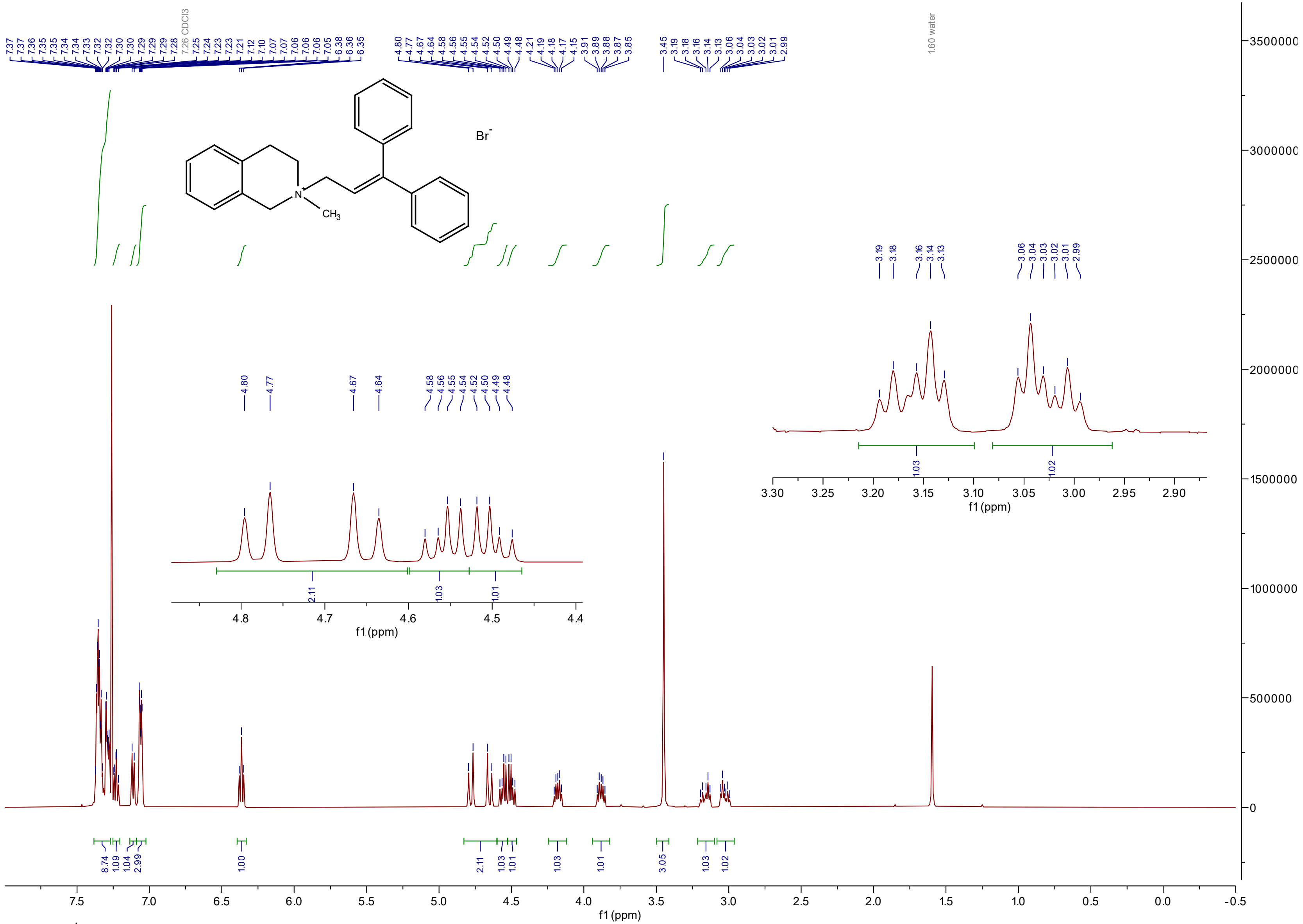

Fig. S127. ^{1}H NMR (500 MHz) of 2-(3,3-diphenylallyl)-2-methyl-1,2,3,4-tetrahydroisoquinolin-2-ium bromide ([**3ad**]Br).

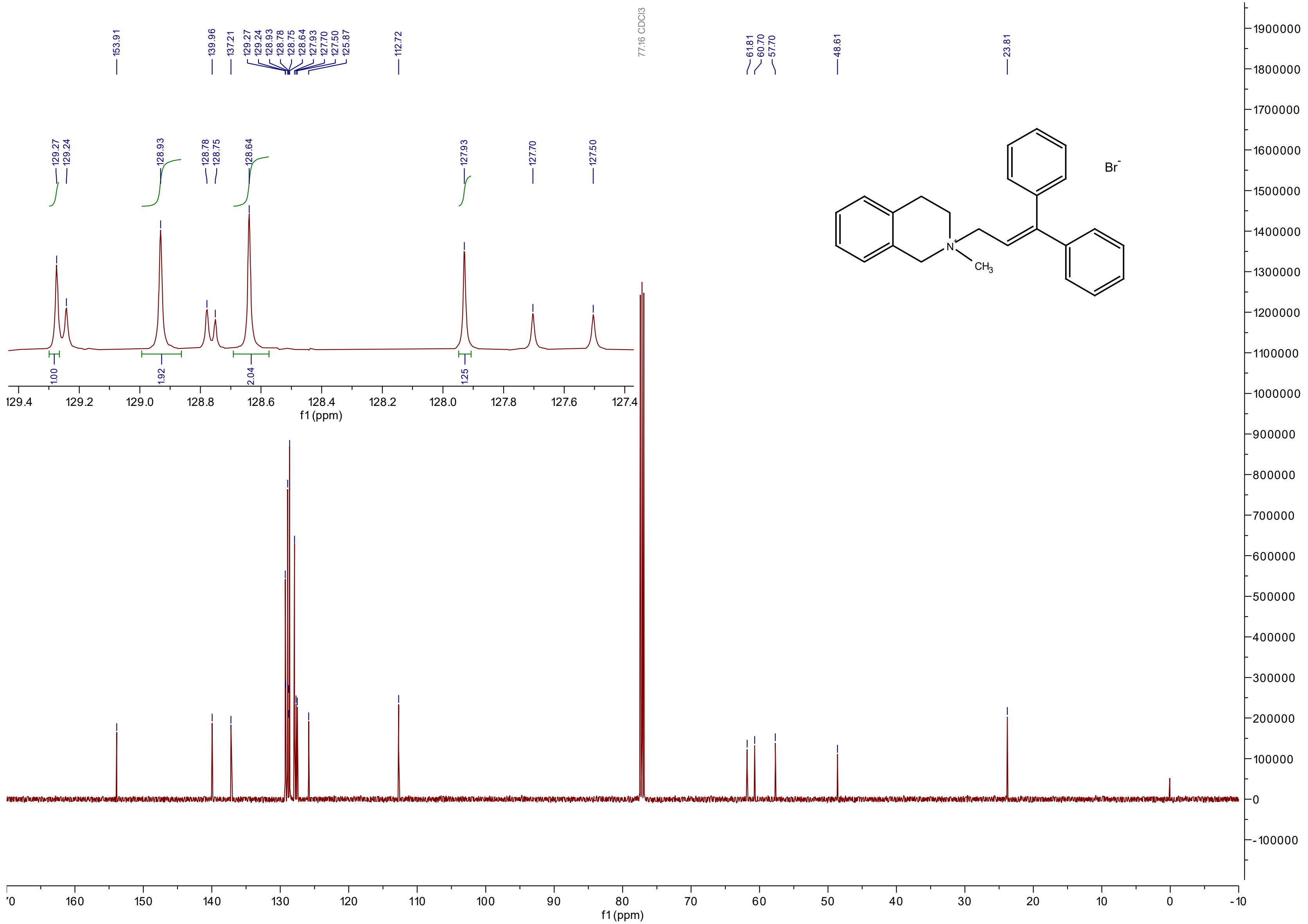

Fig. S128. ^{13}C NMR (126 MHz) of 2-(3,3-diphenylallyl)-2-methyl-1,2,3,4-tetrahydroisoquinolin-2-ium bromide ([**3ad**]Br).

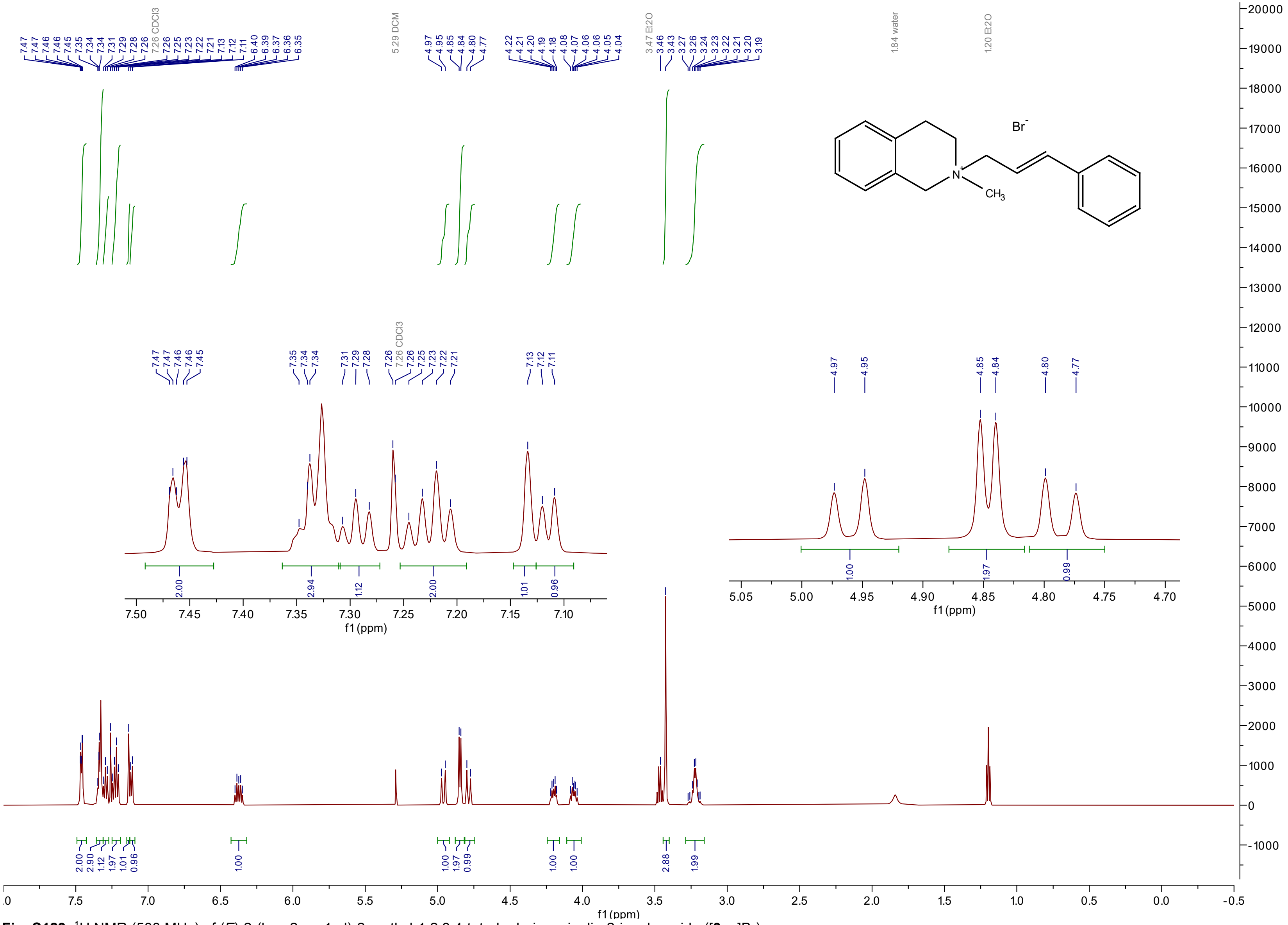

Fig. S129. ^{1}H NMR (500 MHz) of (*E*)-2-(hex-2-en-1-yl)-2-methyl-1,2,3,4-tetrahydroisoquinolin-2-ium bromide ([**3ae**]Br).

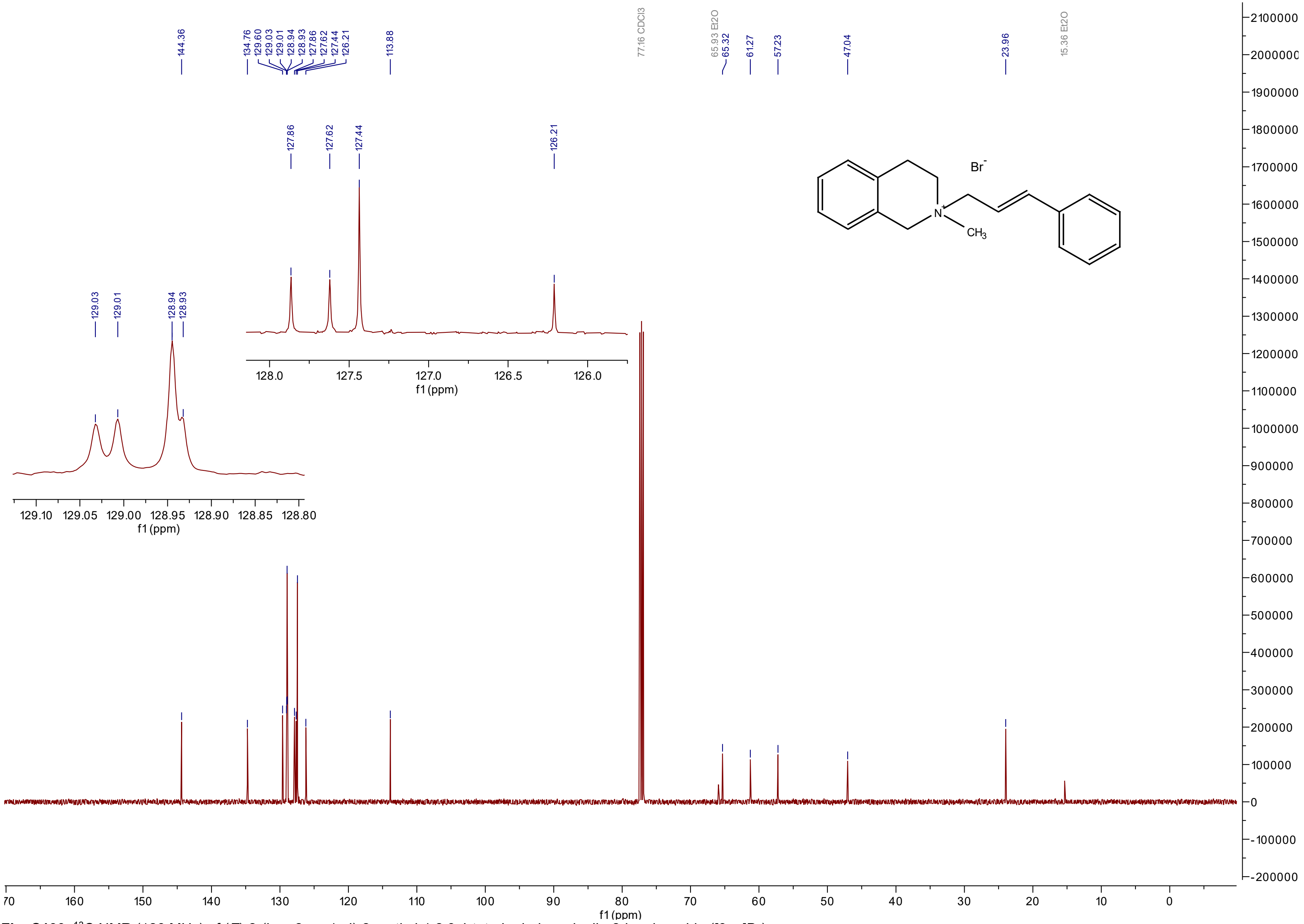

Fig. S130. ^{13}C NMR (126 MHz) of (*E*)-2-(hex-2-en-1-yl)-2-methyl-1,2,3,4-tetrahydroisoquinolin-2-ium bromide ([**3ae**]Br).

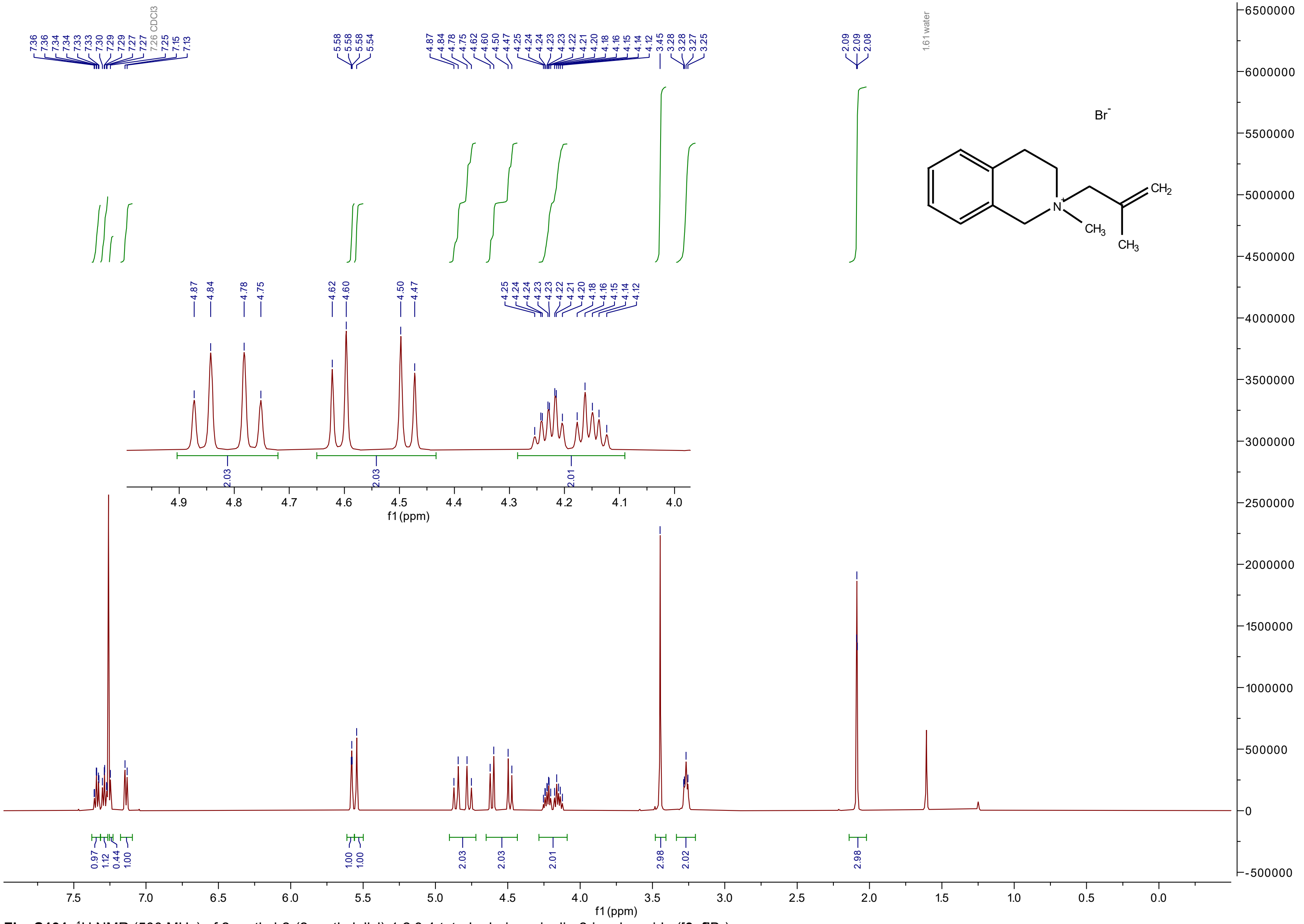

Fig. S131. ^{1}H NMR (500 MHz) of 2-methyl-2-(2-methylallyl)-1,2,3,4-tetrahydroisoquinolin-2-ium bromide ([**3af**]Br).

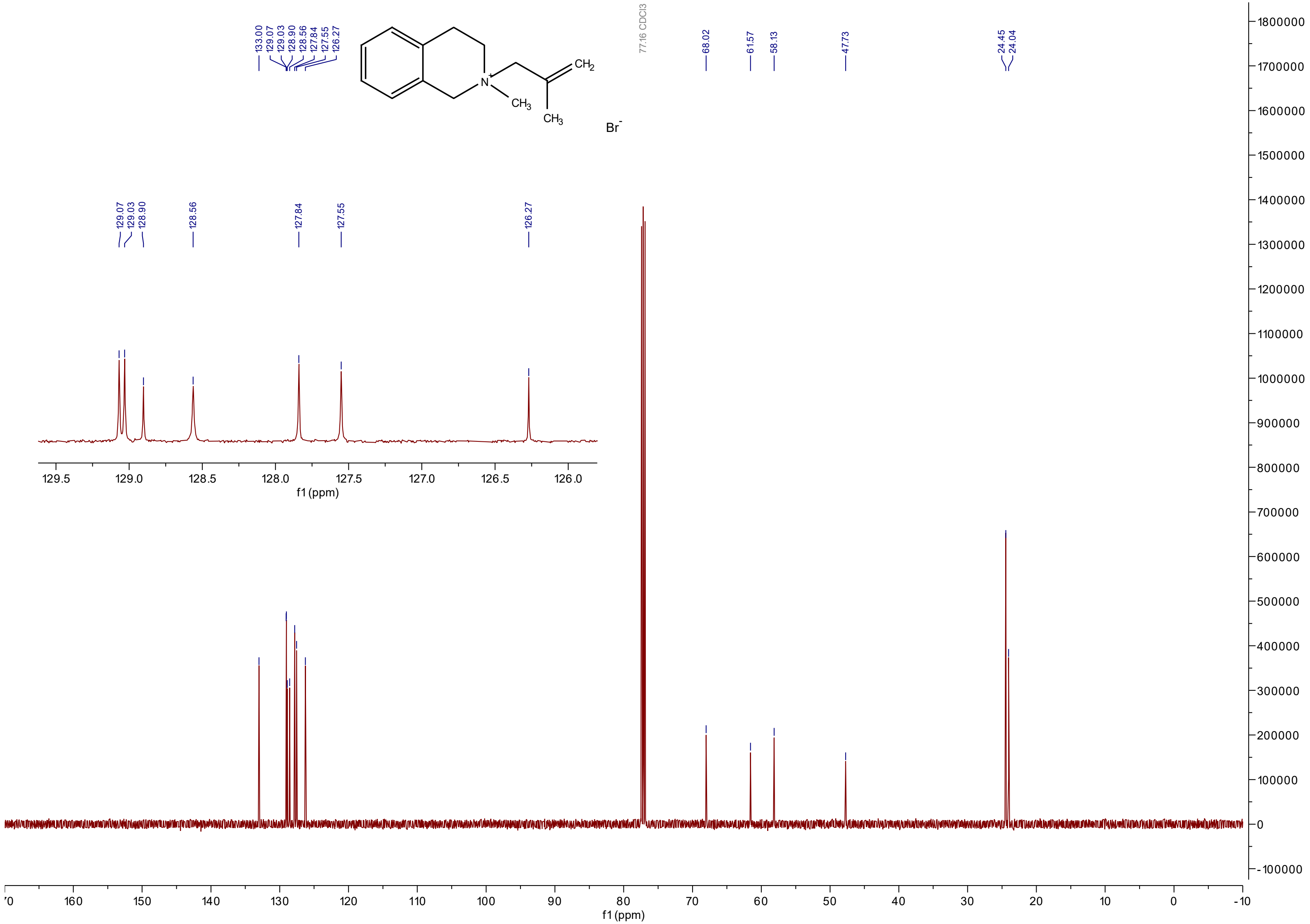

Fig. S132. ^{13}C NMR (126 MHz) of 2-methyl-2-(2-methylallyl)-1,2,3,4-tetrahydroisoquinolin-2-ium bromide ([**3af**]Br).

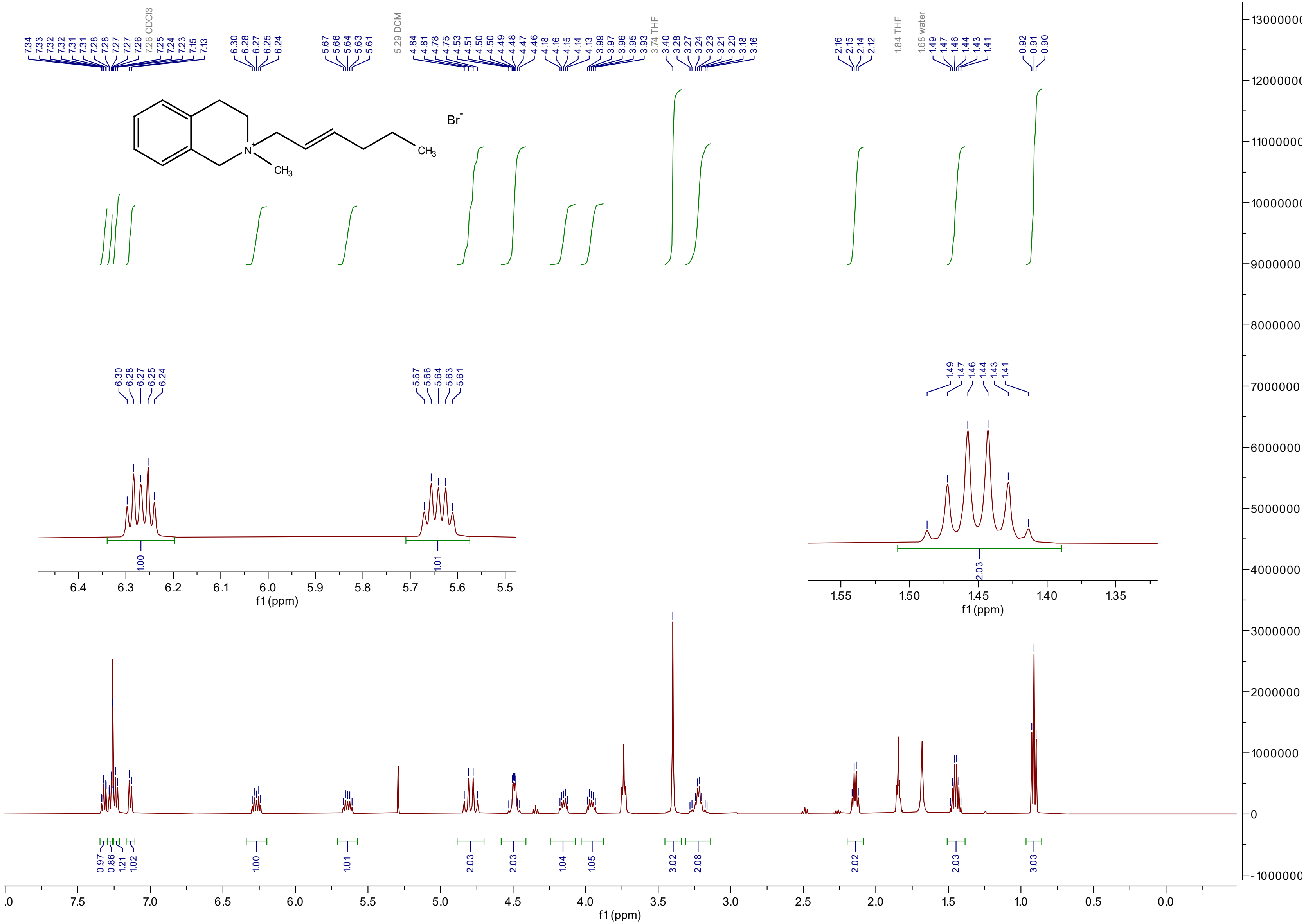

Fig. S133. ^{1}H NMR (500 MHz) of (*E*)-2-(hex-2-en-1-yl)-2-methyl-1,2,3,4-tetrahydroisoquinolin-2-ium bromide ([**3ag**]Br).

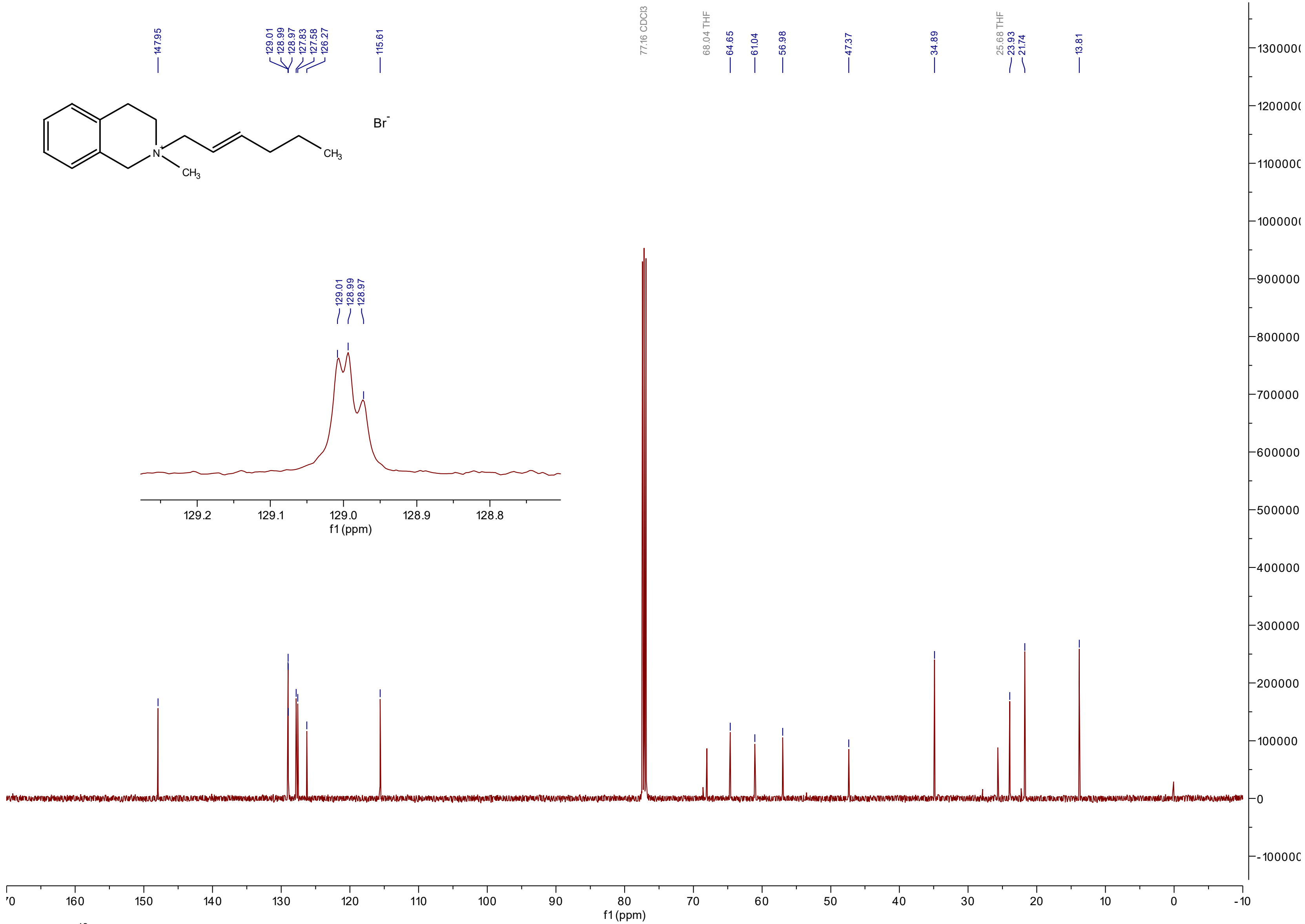

Fig. S134. ^{13}C NMR (126 MHz) of (*E*)-2-(hex-2-en-1-yl)-2-methyl-1,2,3,4-tetrahydroisoquinolin-2-ium bromide ([**3ag**]Br).

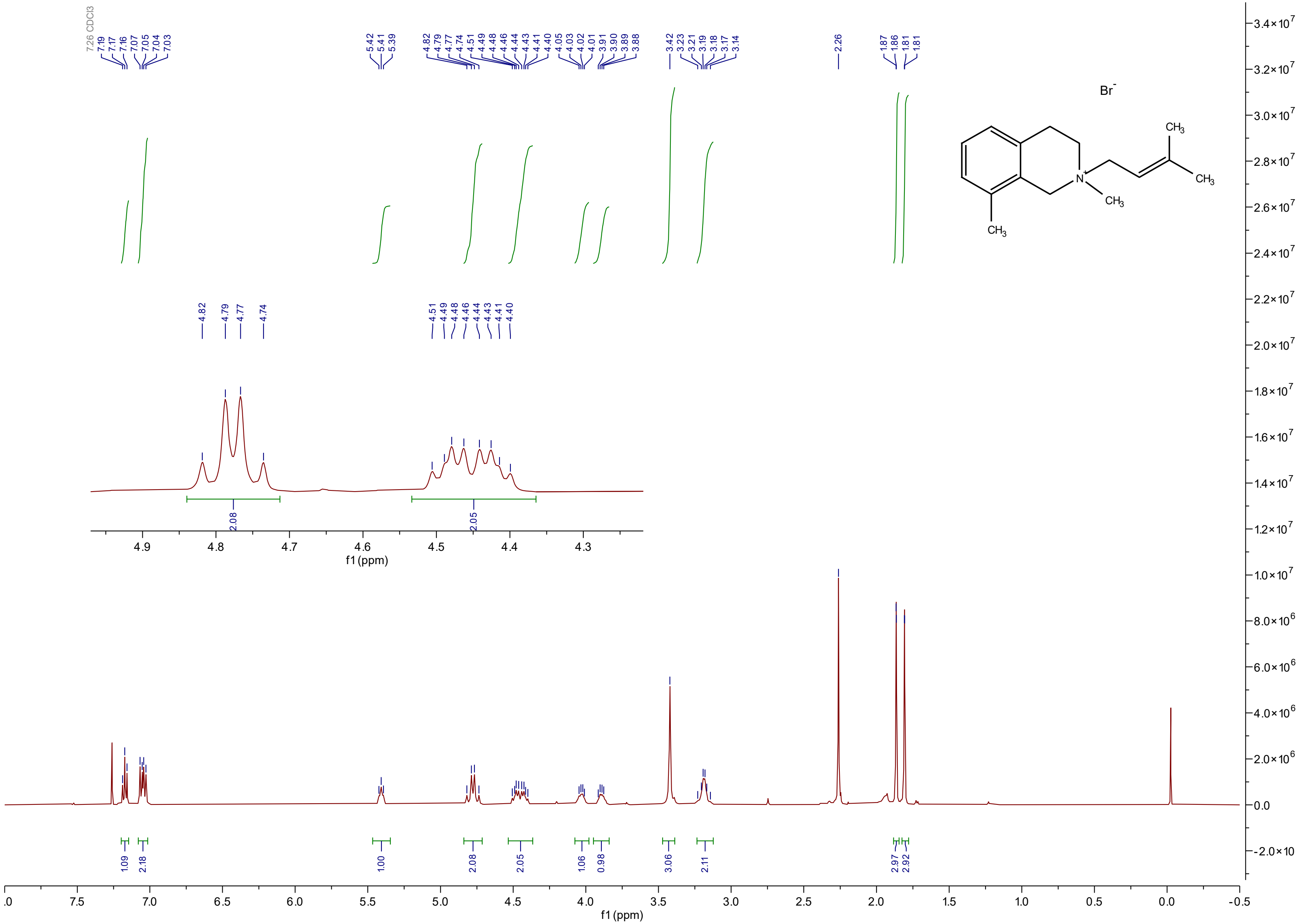

Fig. S135. ^{1}H NMR (500 MHz) of 2,8-dimethyl-2-(3-methylbut-2-en-1-yl)-1,2,3,4-tetrahydroisoquinolin-2-ium bromide ([**3ba**]Br).

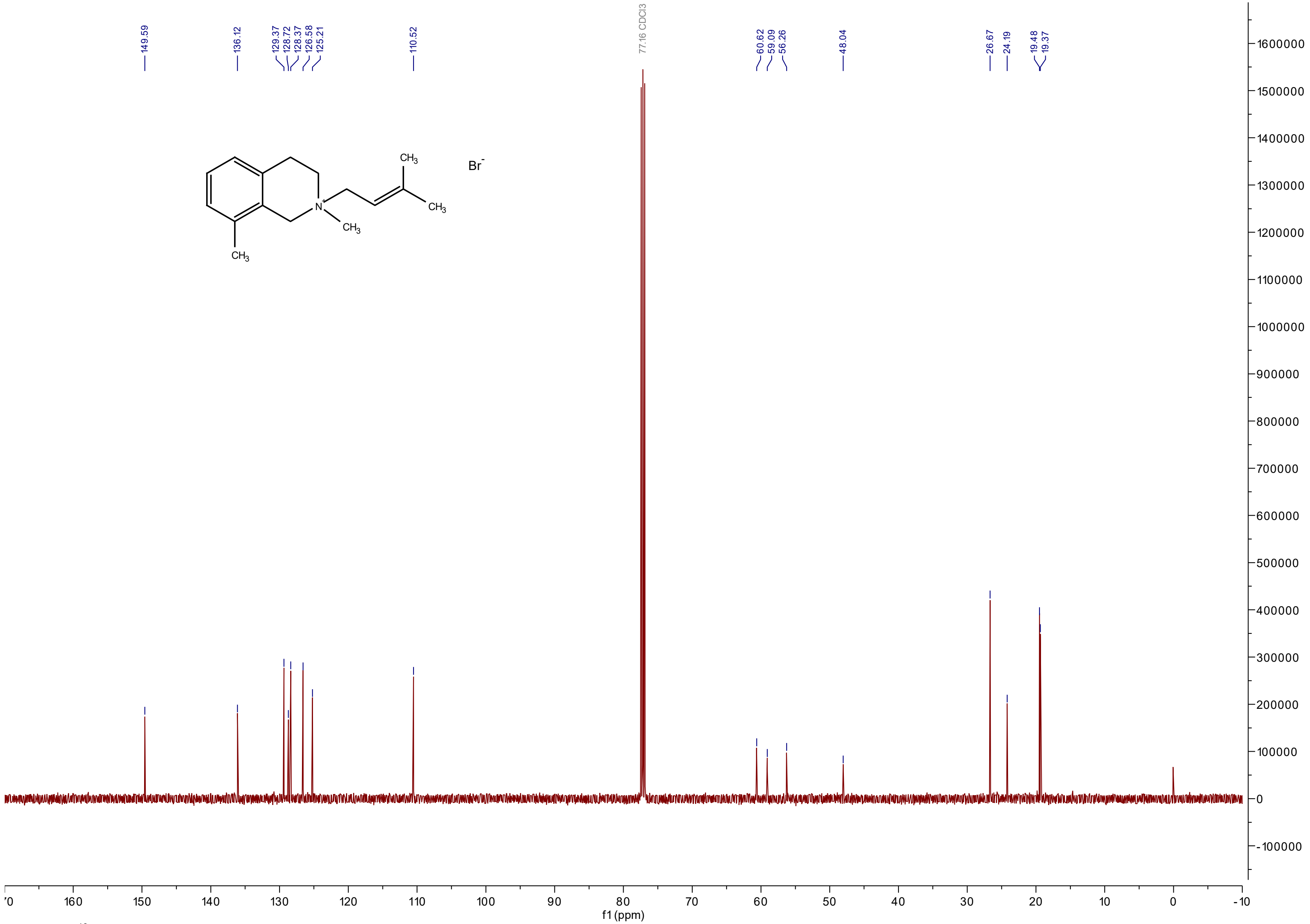

Fig. S136. ^{13}C NMR (126 MHz) of 2,8-dimethyl-2-(3-methylbut-2-en-1-yl)-1,2,3,4-tetrahydroisoquinolin-2-ium bromide ([**3ba**]Br).

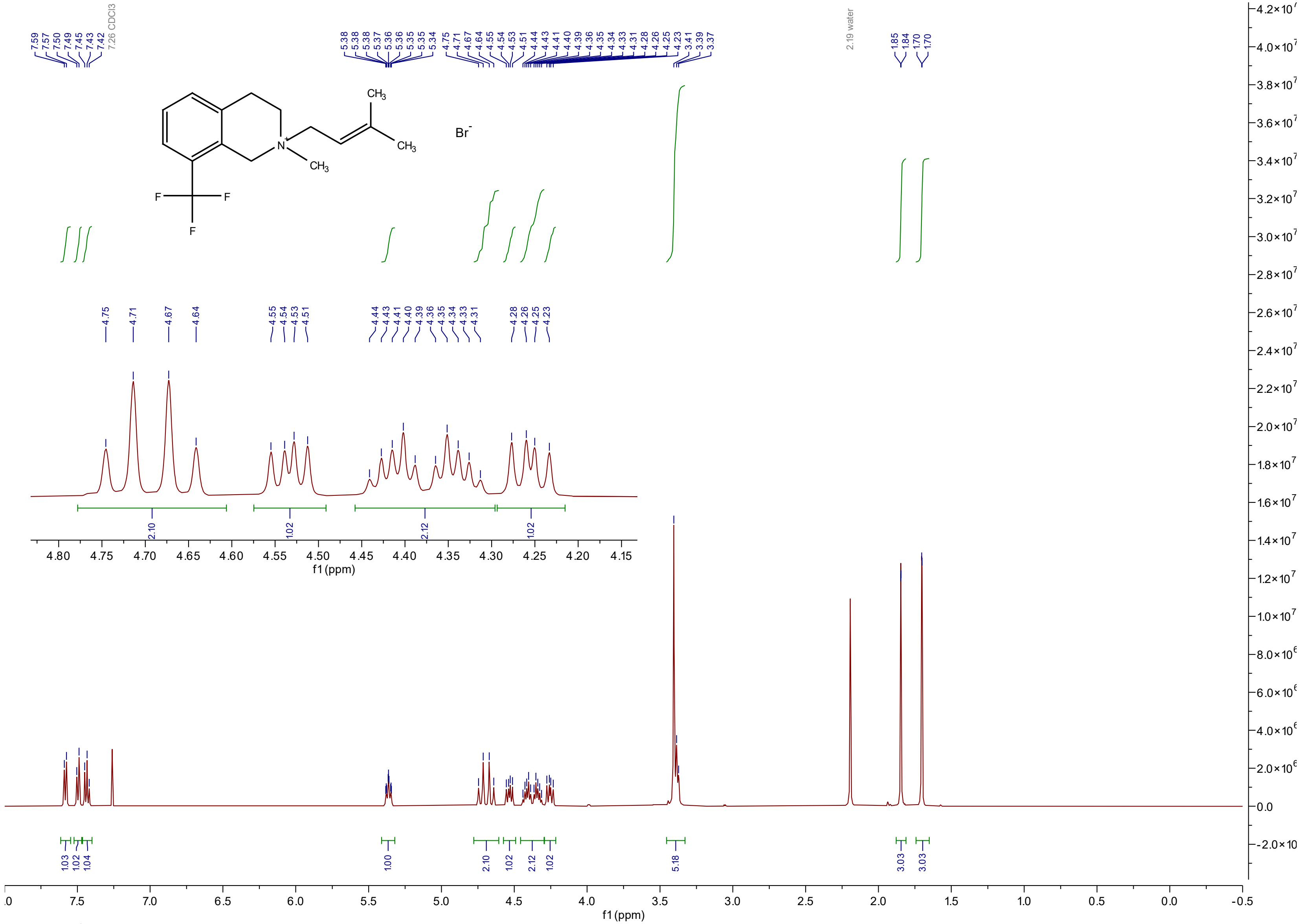

Fig. S137. ^{1}H NMR (500 MHz) of 2-methyl-2-(3-methylbut-2-en-1-yl)-8-(trifluoromethyl)-1,2,3,4-tetrahydroisoquinolin-2-ium bromide ([**3ca**]Br).

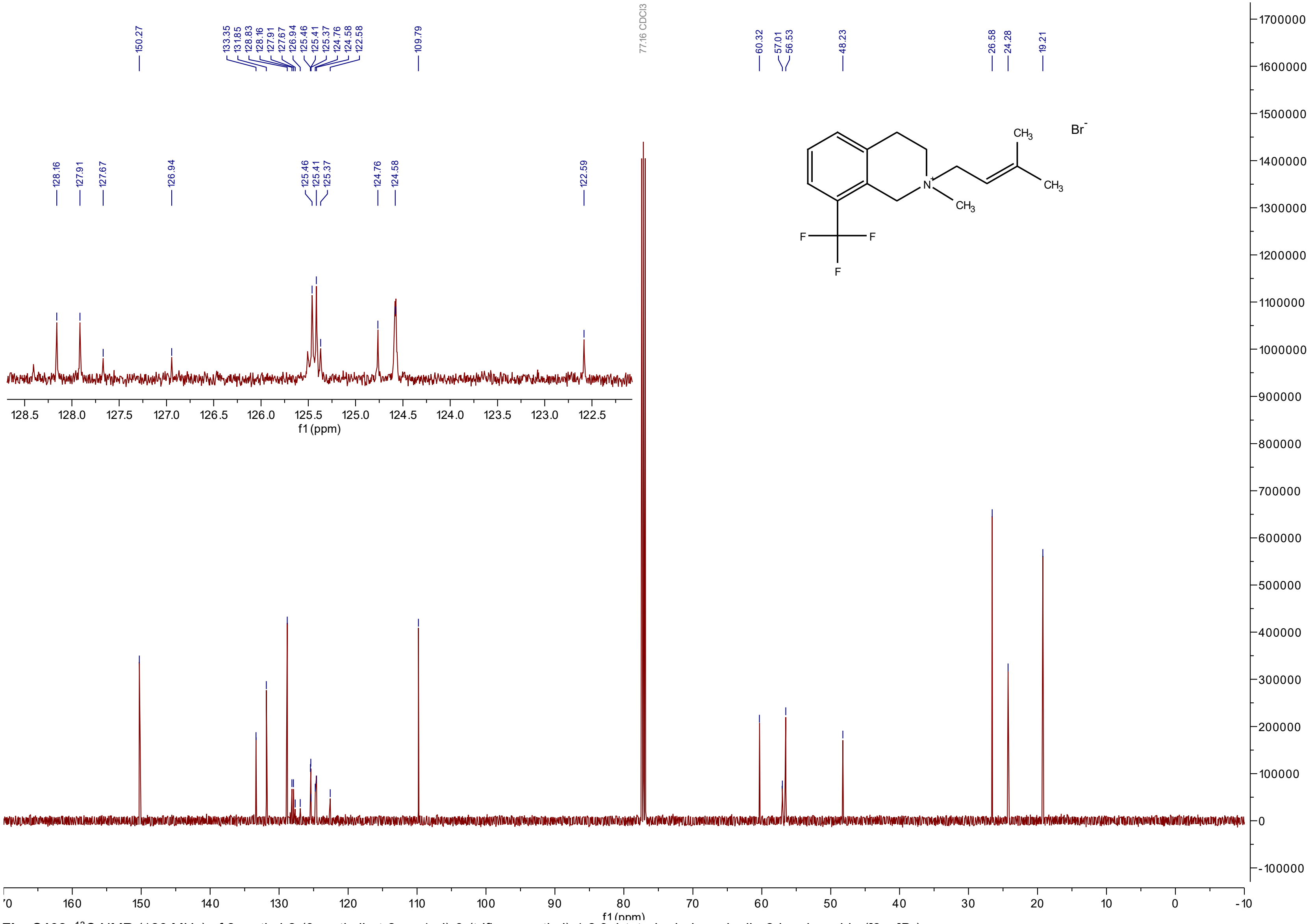

Fig. S138. ^{13}C NMR (126 MHz) of 2-methyl-2-(3-methylbut-2-en-1-yl)-8-(trifluoromethyl)-1,2,3,4-tetrahydroisoquinolin-2-ium bromide ([**3ca**]Br).

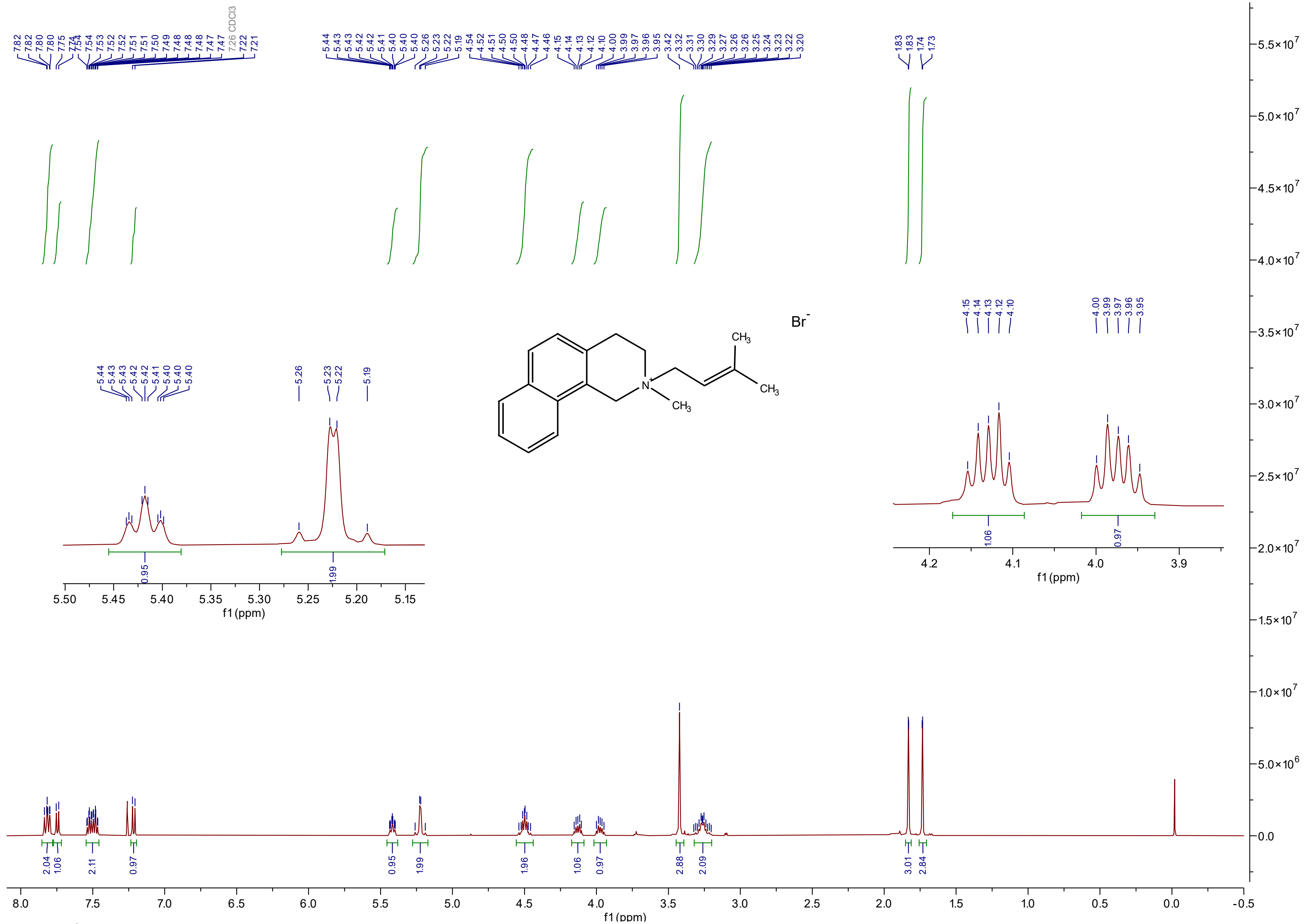

Fig. S139. ^{1}H NMR (500 MHz) of 2-methyl-2-(3-methylbut-2-en-1-yl)-1,2,3,4-tetrahydrobenzo[*h*]isoquinolin-2-ium bromide ([**3da**]Br).

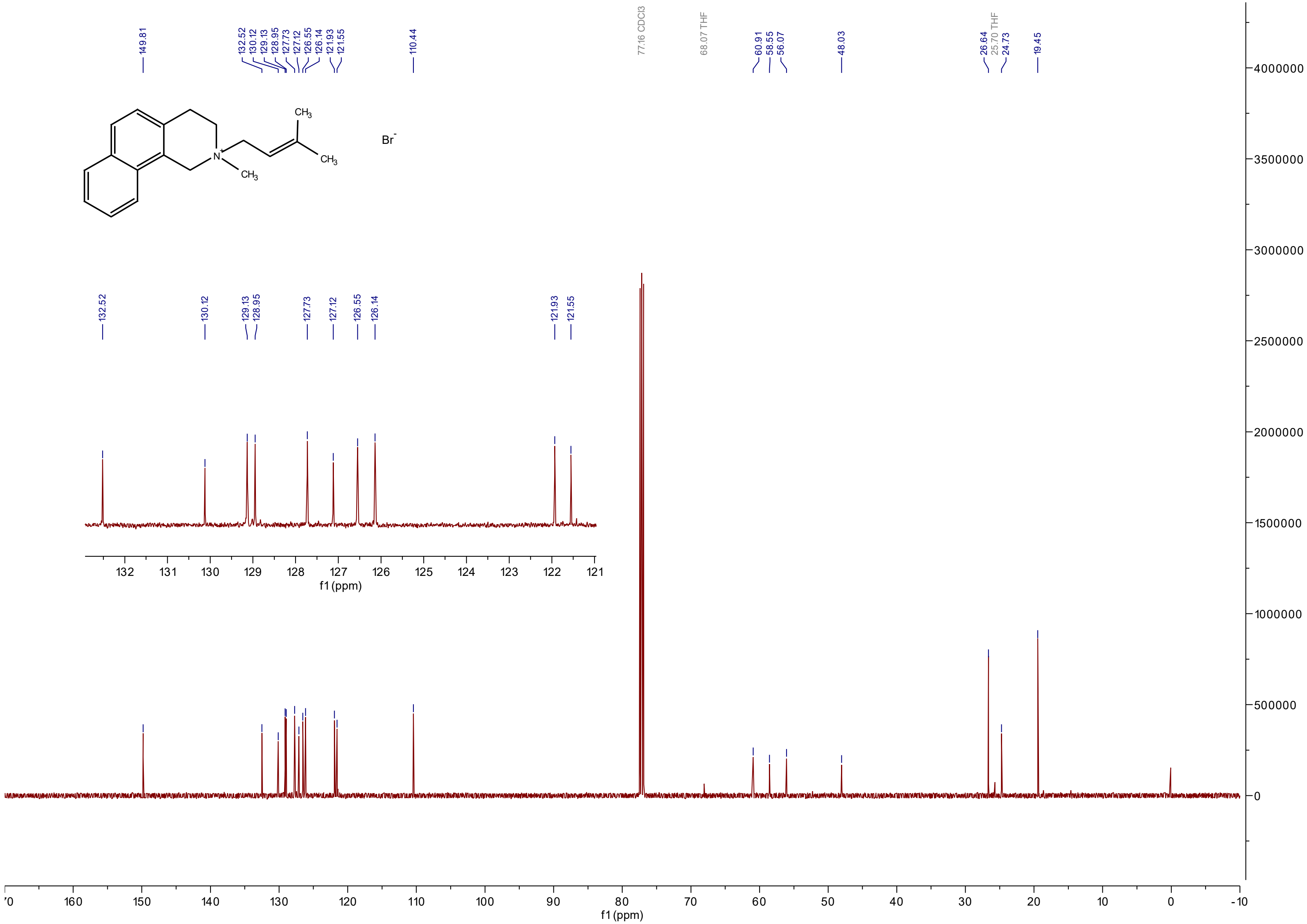

Fig. S140. ^{13}C NMR (126 MHz) of 2-methyl-2-(3-methylbut-2-en-1-yl)-1,2,3,4-tetrahydrobenzo[*h*]isoquinolin-2-ium bromide ([**3da**]Br).

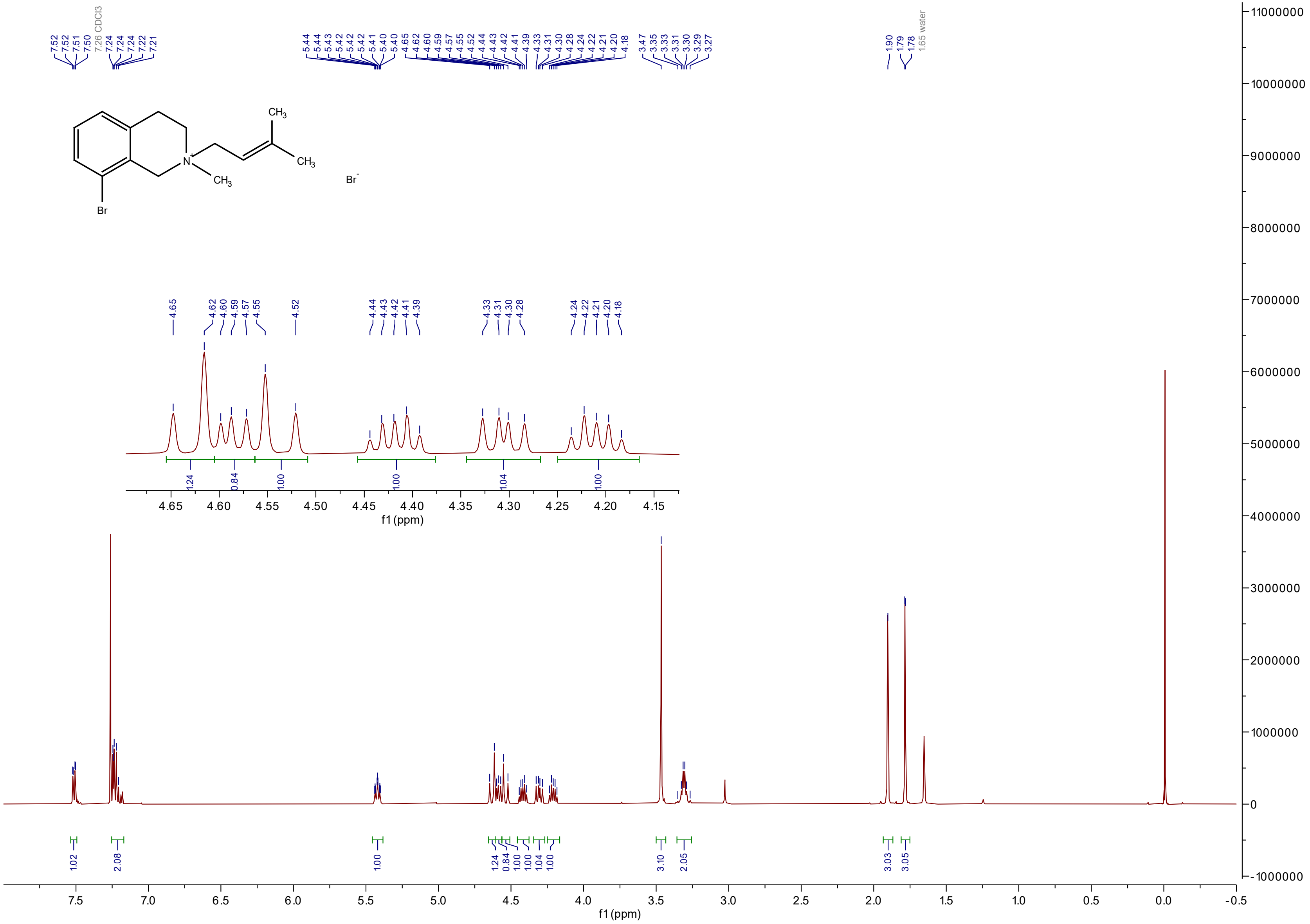

Fig. S141. ^{1}H NMR (500 MHz) of 8-bromo-2-methyl-2-(3-methylbut-2-en-1-yl)-1,2,3,4-tetrahydroisoquinolin-2-ium bromide ([**3ea**]Br).

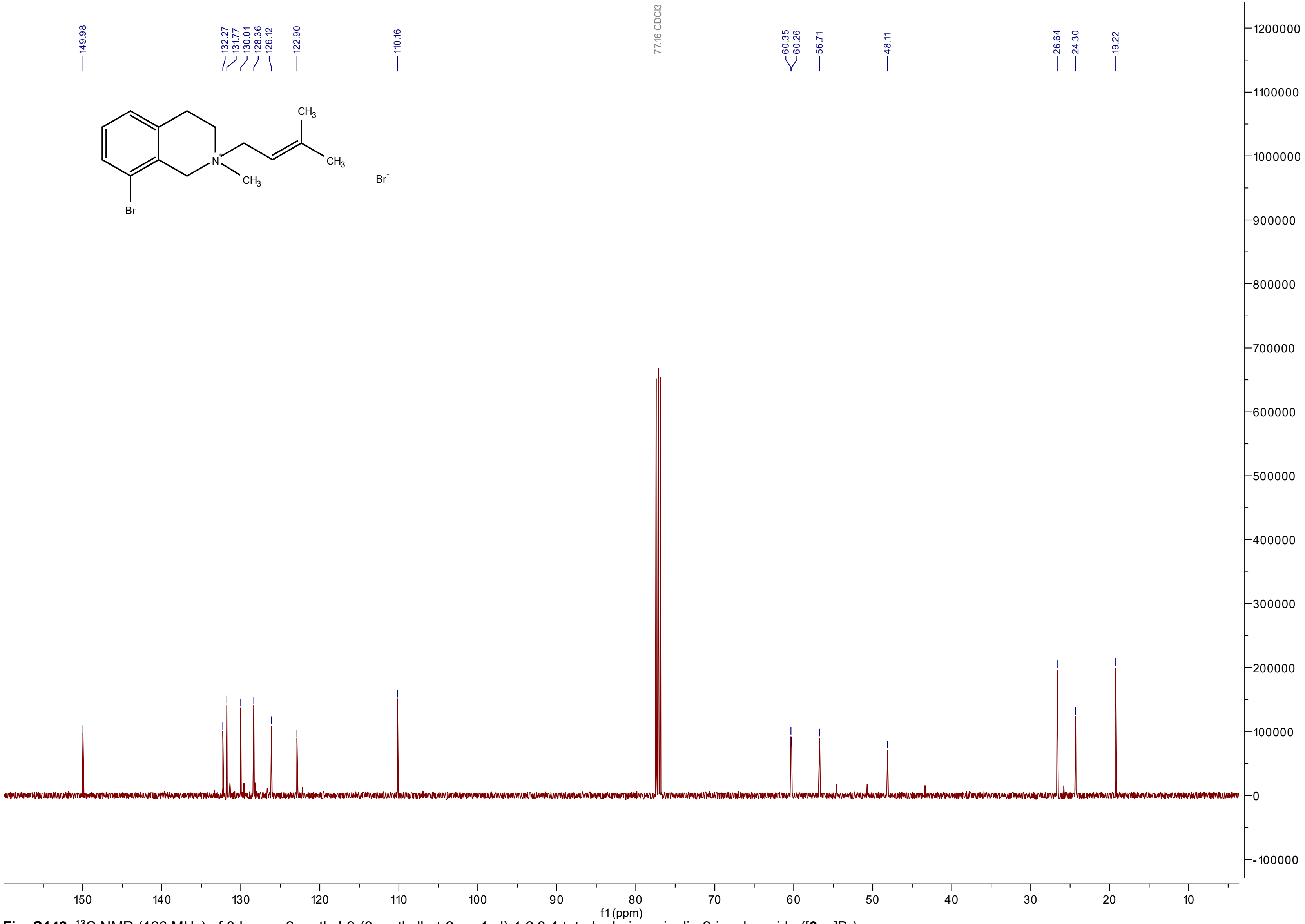

Fig. S142. ^{13}C NMR (126 MHz) of 8-bromo-2-methyl-2-(3-methylbut-2-en-1-yl)-1,2,3,4-tetrahydroisoquinolin-2-ium bromide ([**3ea**]Br).

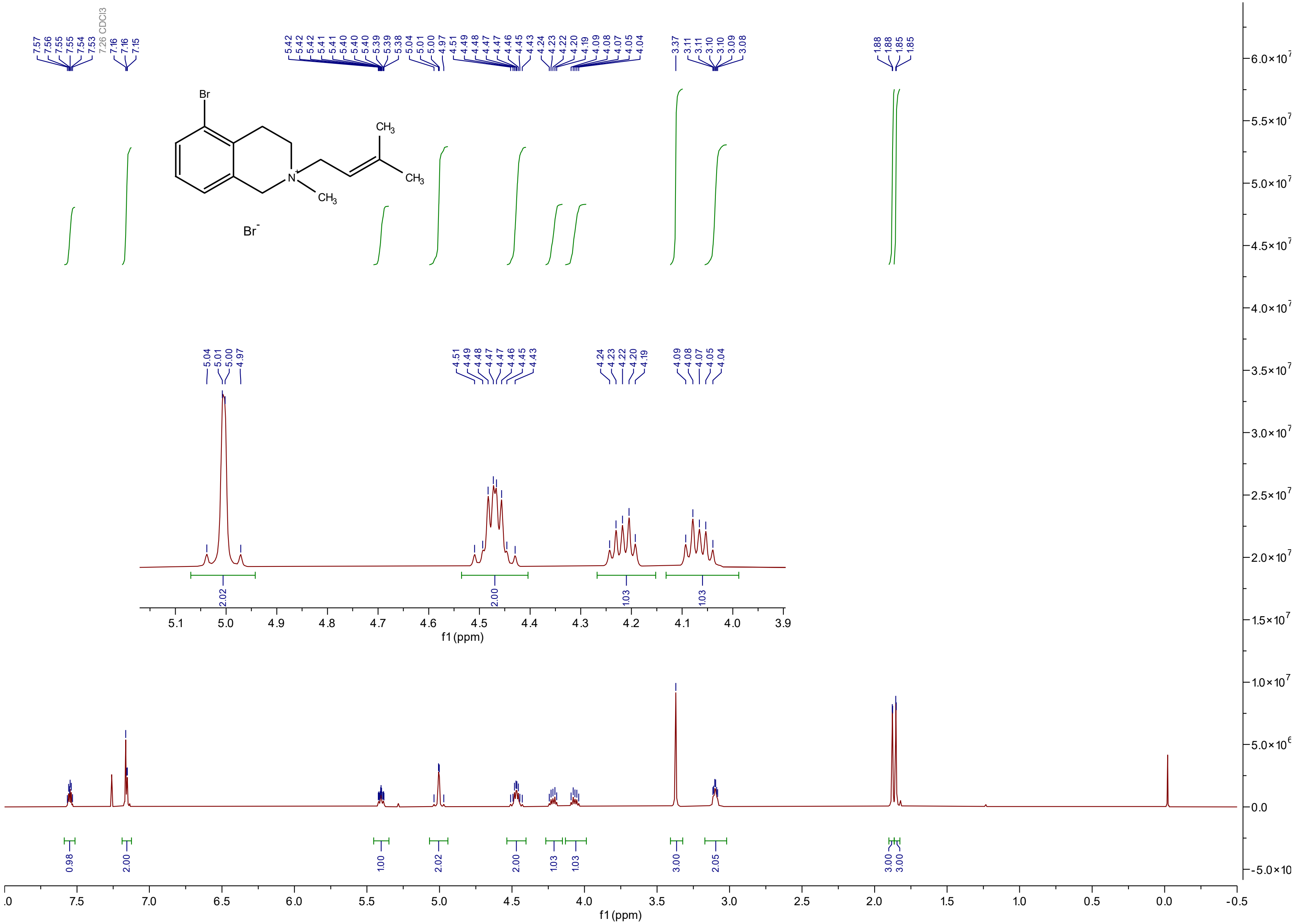

Fig. S143. ^{1}H NMR (500 MHz) of 5-bromo-2-methyl-2-(3-methylbut-2-en-1-yl)-1,2,3,4-tetrahydroisoquinolin-2-ium bromide ([**3fa**]Br).

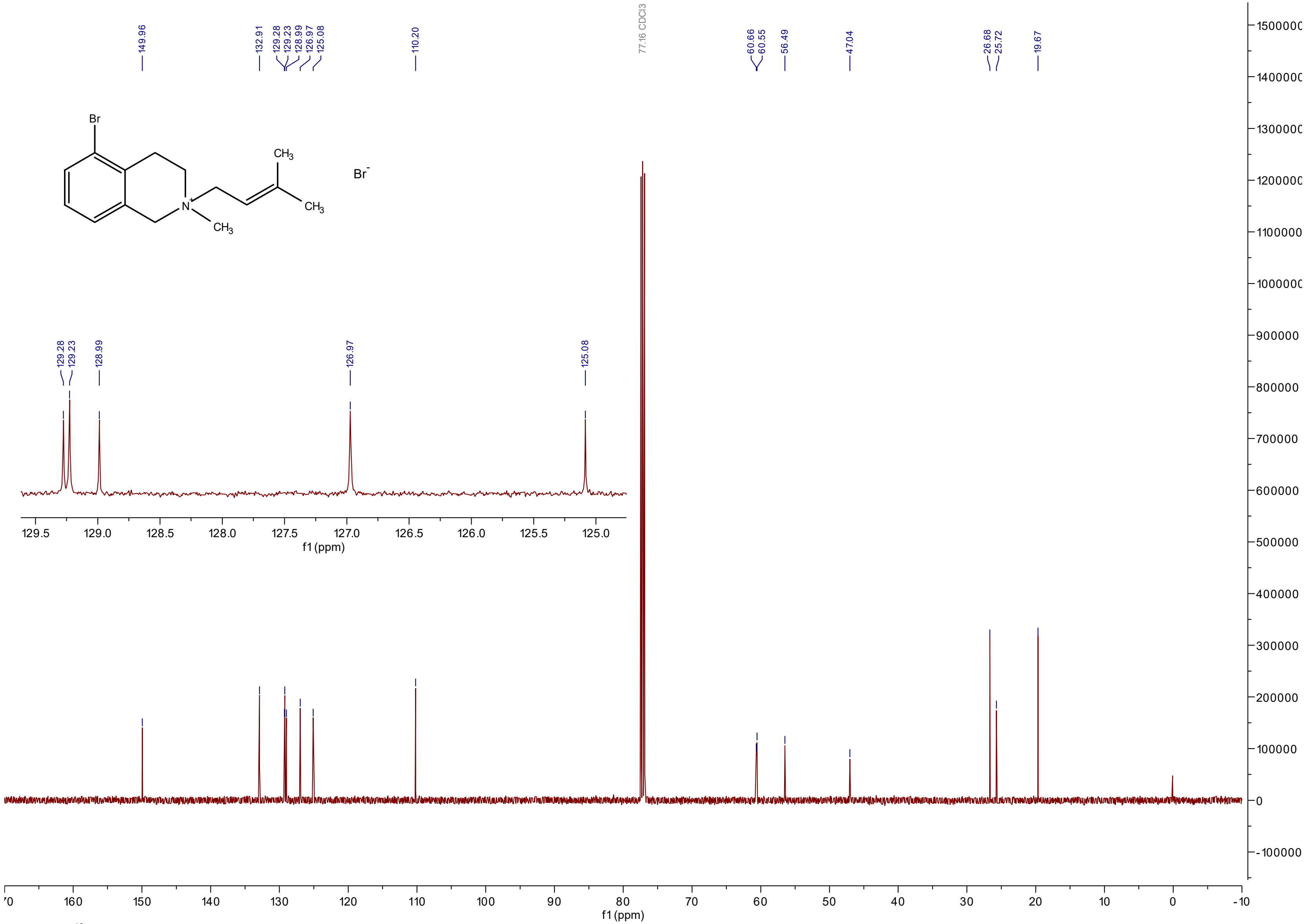

Fig. S144. ^{13}C NMR (126 MHz) of 5-bromo-2-methyl-2-(3-methylbut-2-en-1-yl)-1,2,3,4-tetrahydroisoquinolin-2-ium bromide ([**3fa**]Br).

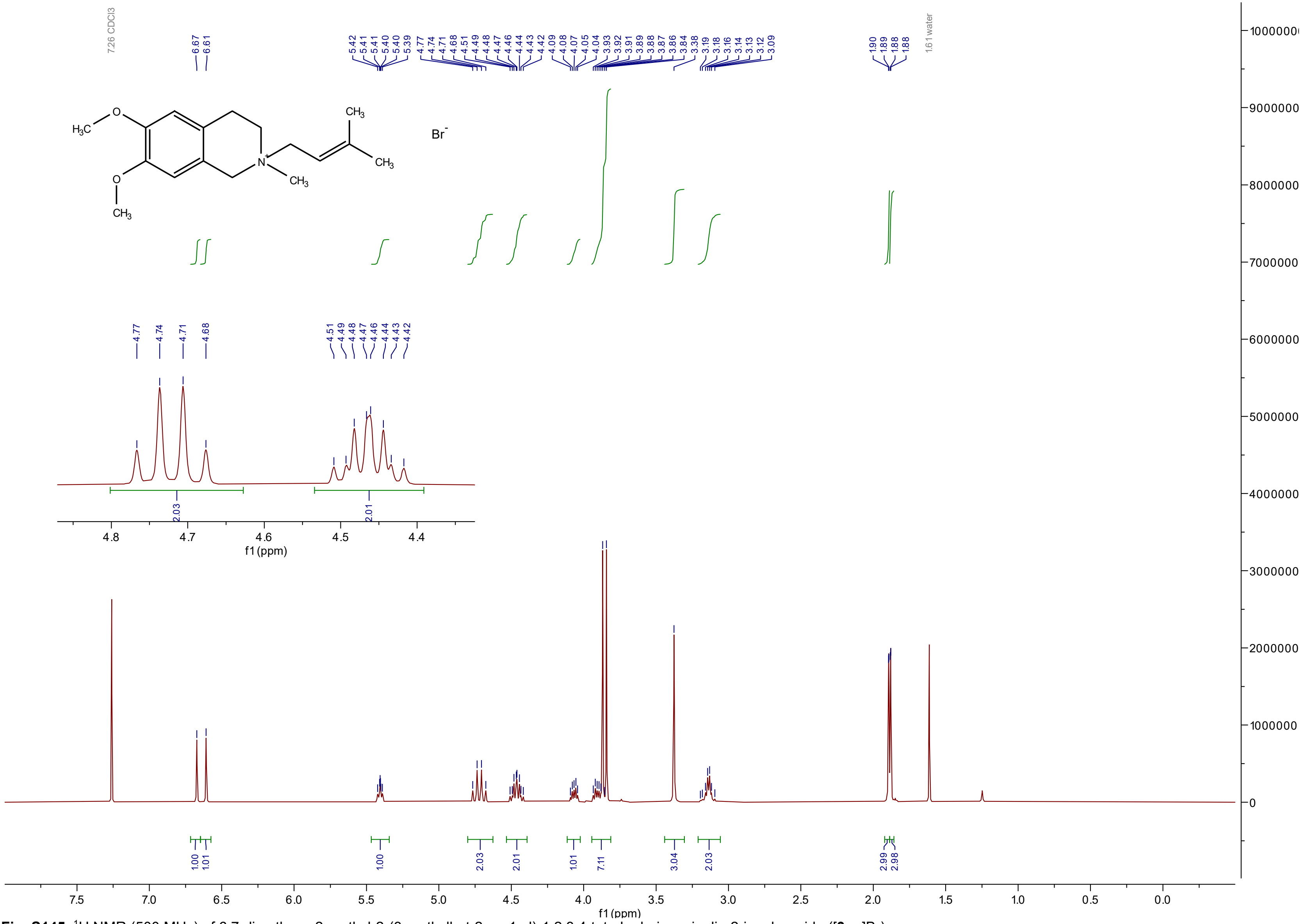

Fig. S145. ^{1}H NMR (500 MHz) of 6,7-dimethoxy-2-methyl-2-(3-methylbut-2-en-1-yl)-1,2,3,4-tetrahydroisoquinolin-2-ium bromide ([**3ga**]Br).

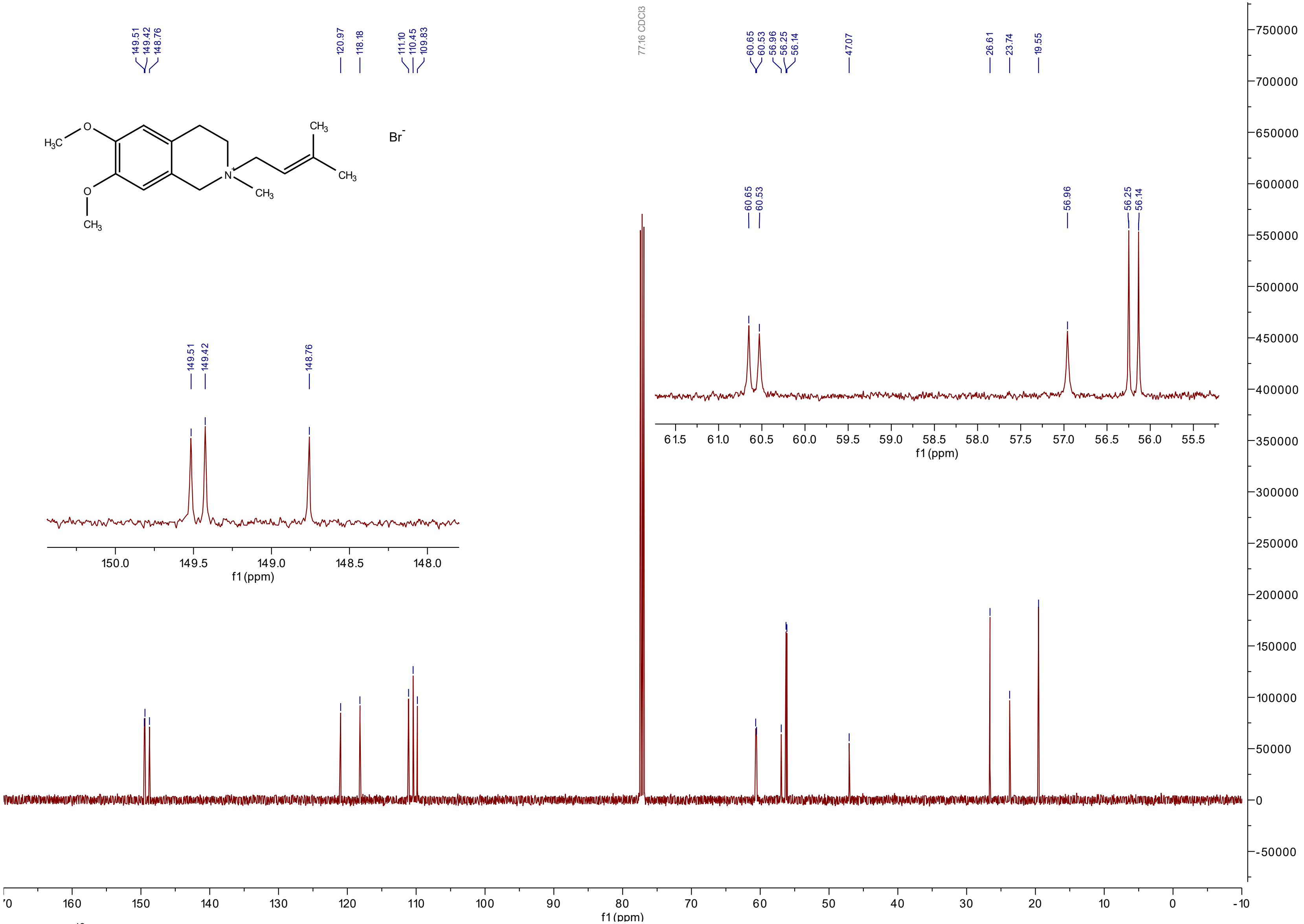

Fig. S146. ^{13}C NMR (126 MHz) of 6,7-dimethoxy-2-methyl-2-(3-methylbut-2-en-1-yl)-1,2,3,4-tetrahydroisoquinolin-2-ium bromide ([**3ga**]Br).

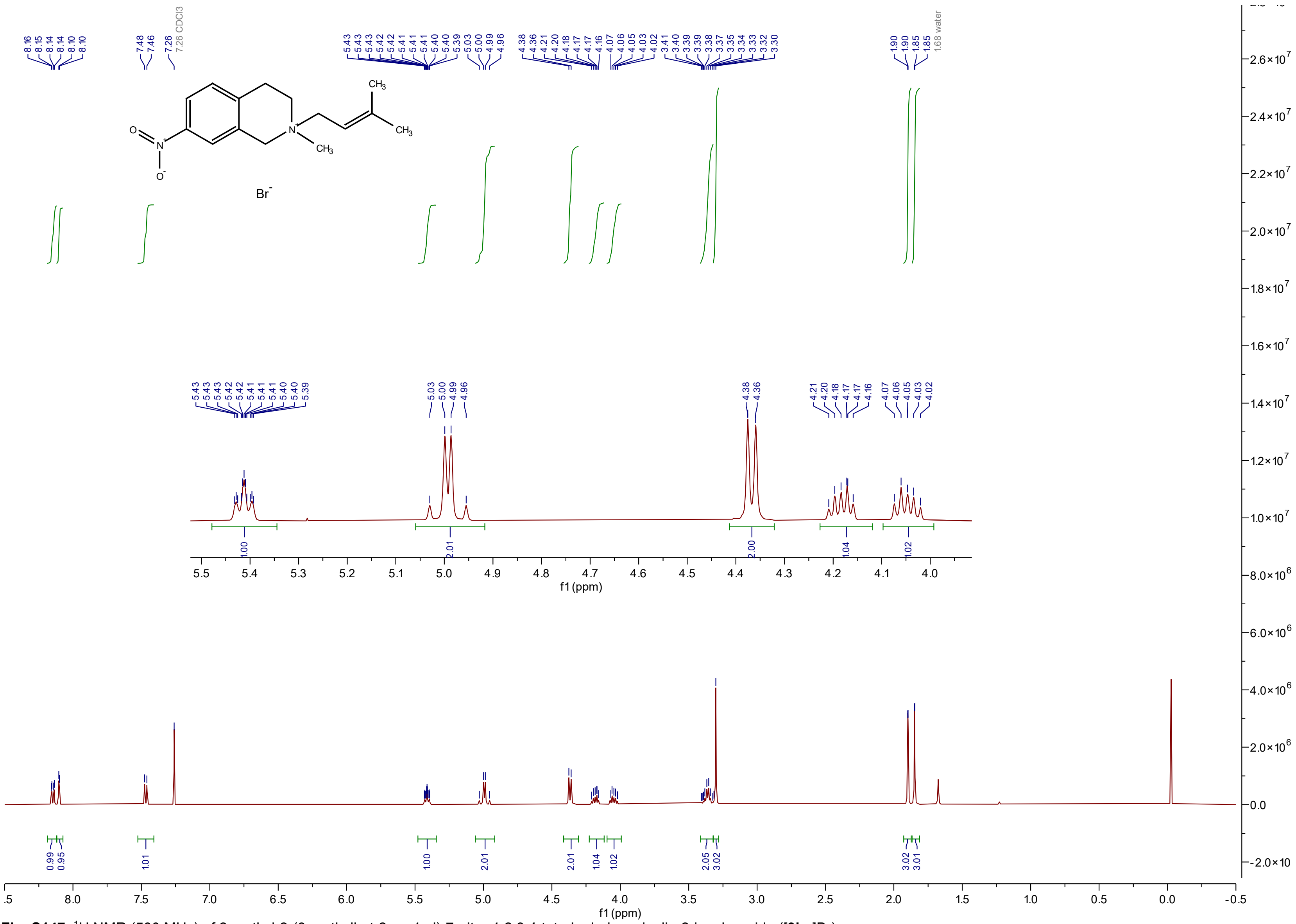

Fig. S147. ^{1}H NMR (500 MHz) of 2-methyl-2-(3-methylbut-2-en-1-yl)-7-nitro-1,2,3,4-tetrahydroisoquinolin-2-ium bromide ([**3ha**]Br).

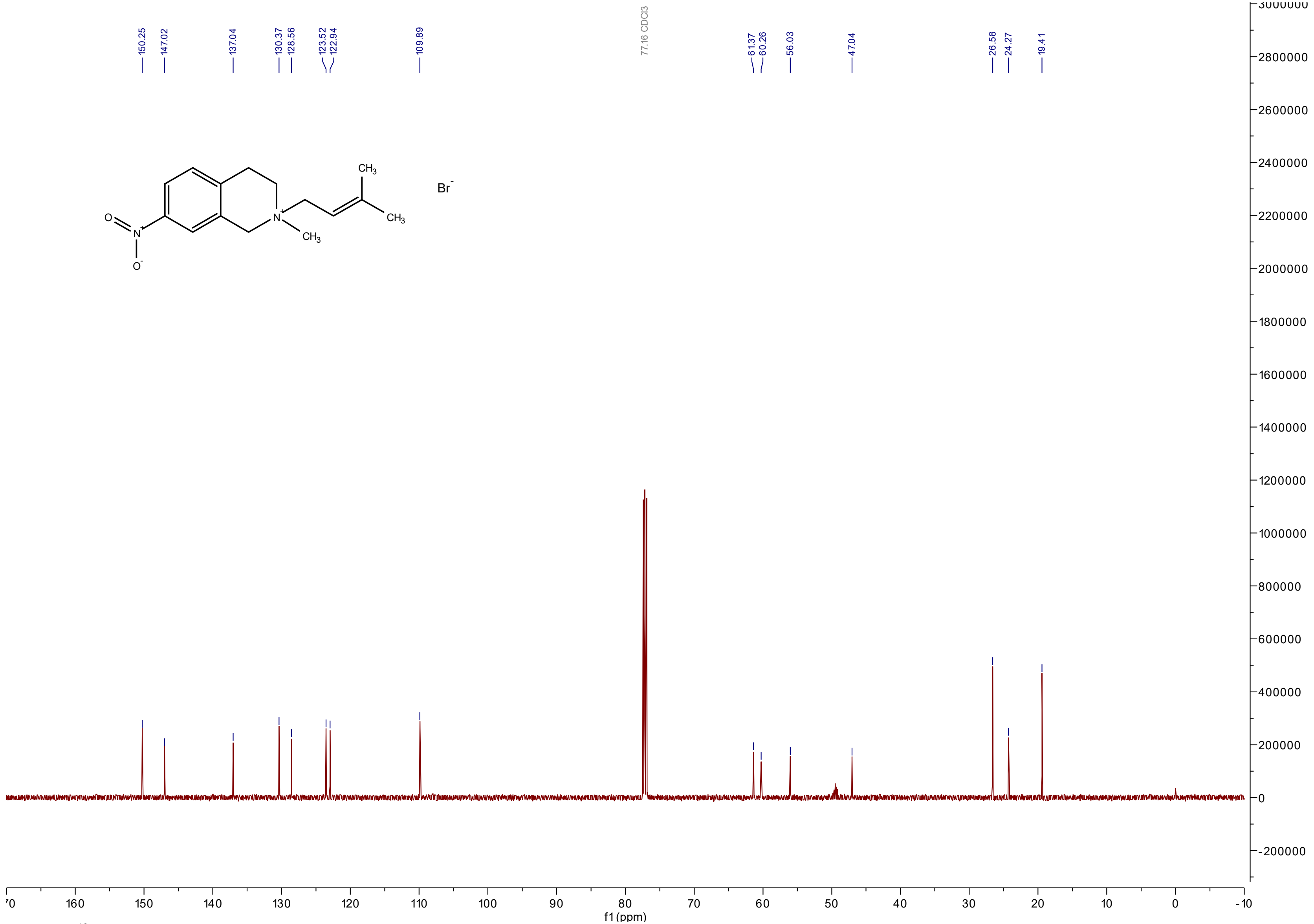

Fig. S148. ^{13}C NMR (126 MHz) of 2-methyl-2-(3-methylbut-2-en-1-yl)-7-nitro-1,2,3,4-tetrahydroisoquinolin-2-ium bromide ([**3ha**]Br).

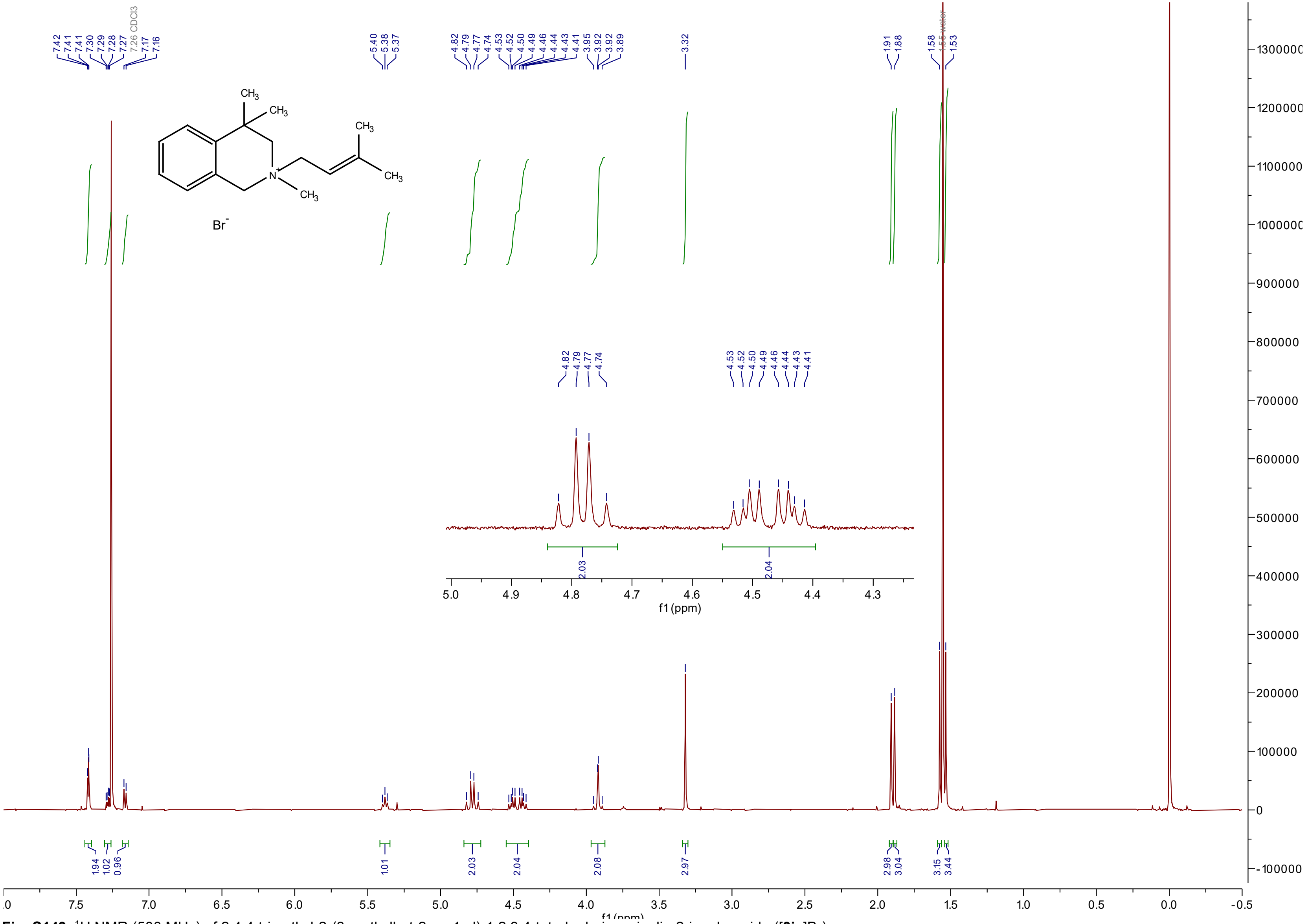

Fig. S149. ^{1}H NMR (500 MHz) of 2,4,4-trimethyl-2-(3-methylbut-2-en-1-yl)-1,2,3,4-tetrahydroisoquinolin-2-ium bromide ([**3ia**]Br).

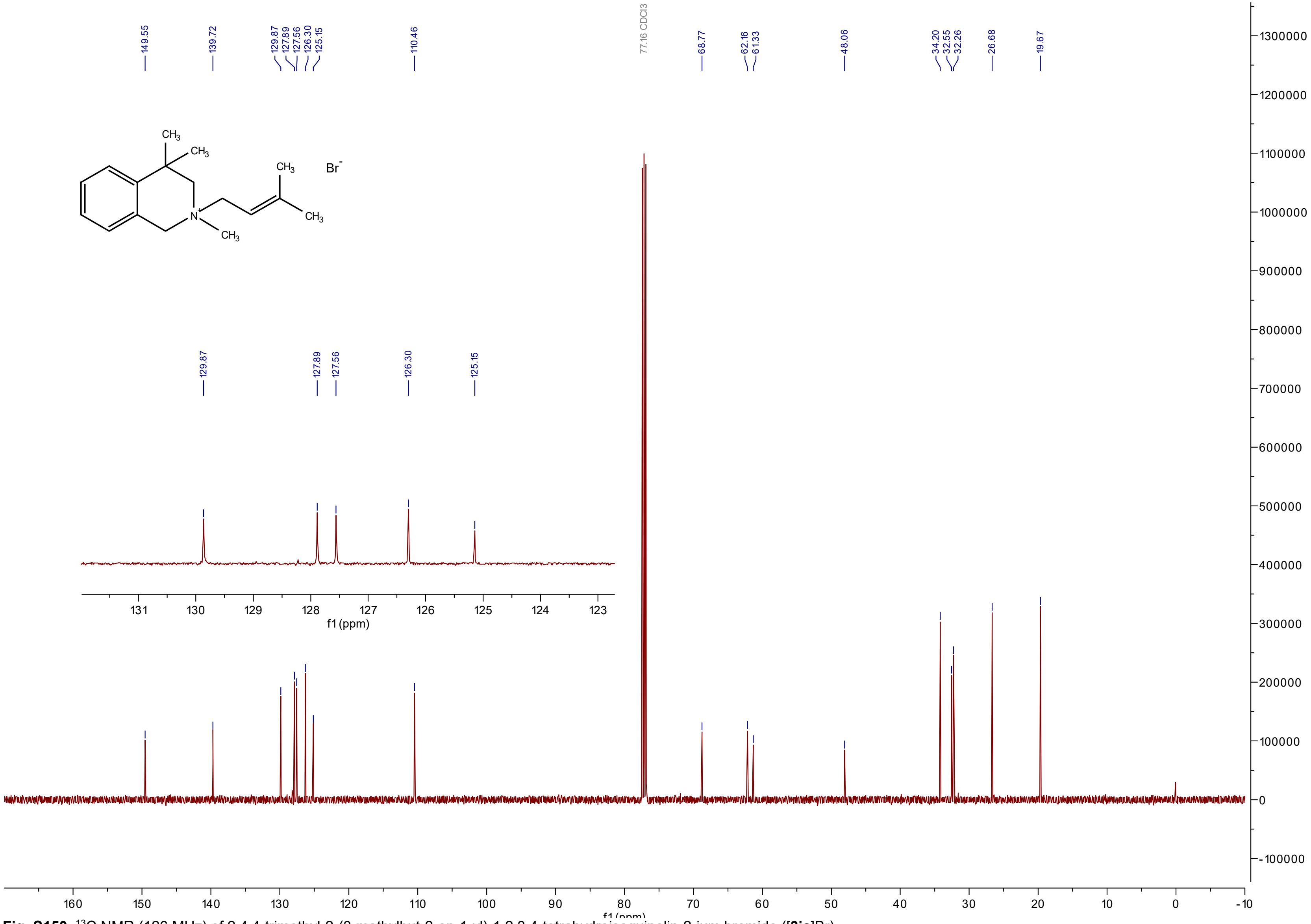

Fig. S150. ^{13}C NMR (126 MHz) of 2,4,4-trimethyl-2-(3-methylbut-2-en-1-yl)-1,2,3,4-tetrahydroisoquinolin-2-ium bromide ([**3ia**]Br).

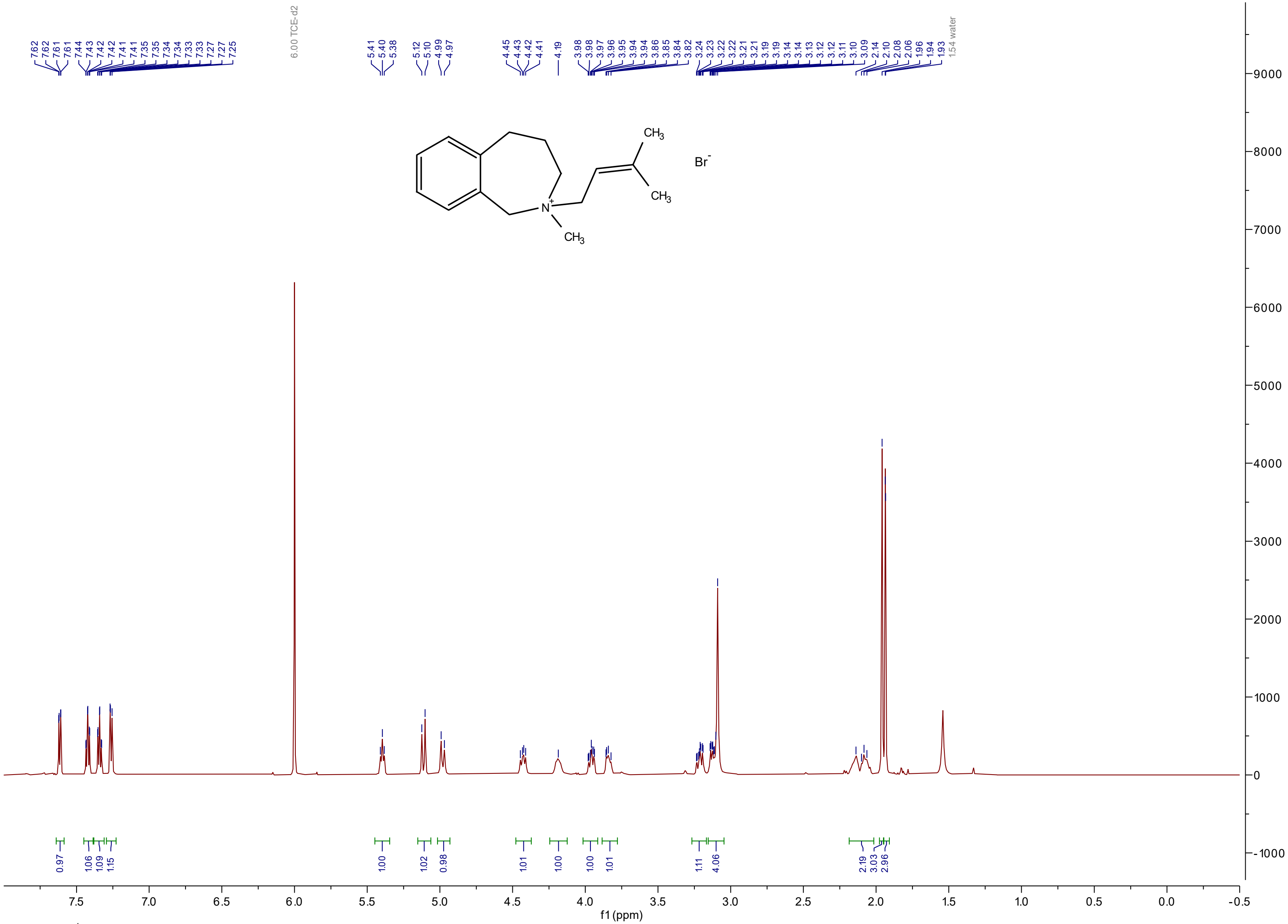

Fig. S151. ^{1}H NMR (600 MHz) of 2-methyl-2-(3-methylbut-2-en-1-yl)-2,3,4,5-tetrahydro-1H-benzo[c]azepin-2-ium bromide ([**3ja**]Br).

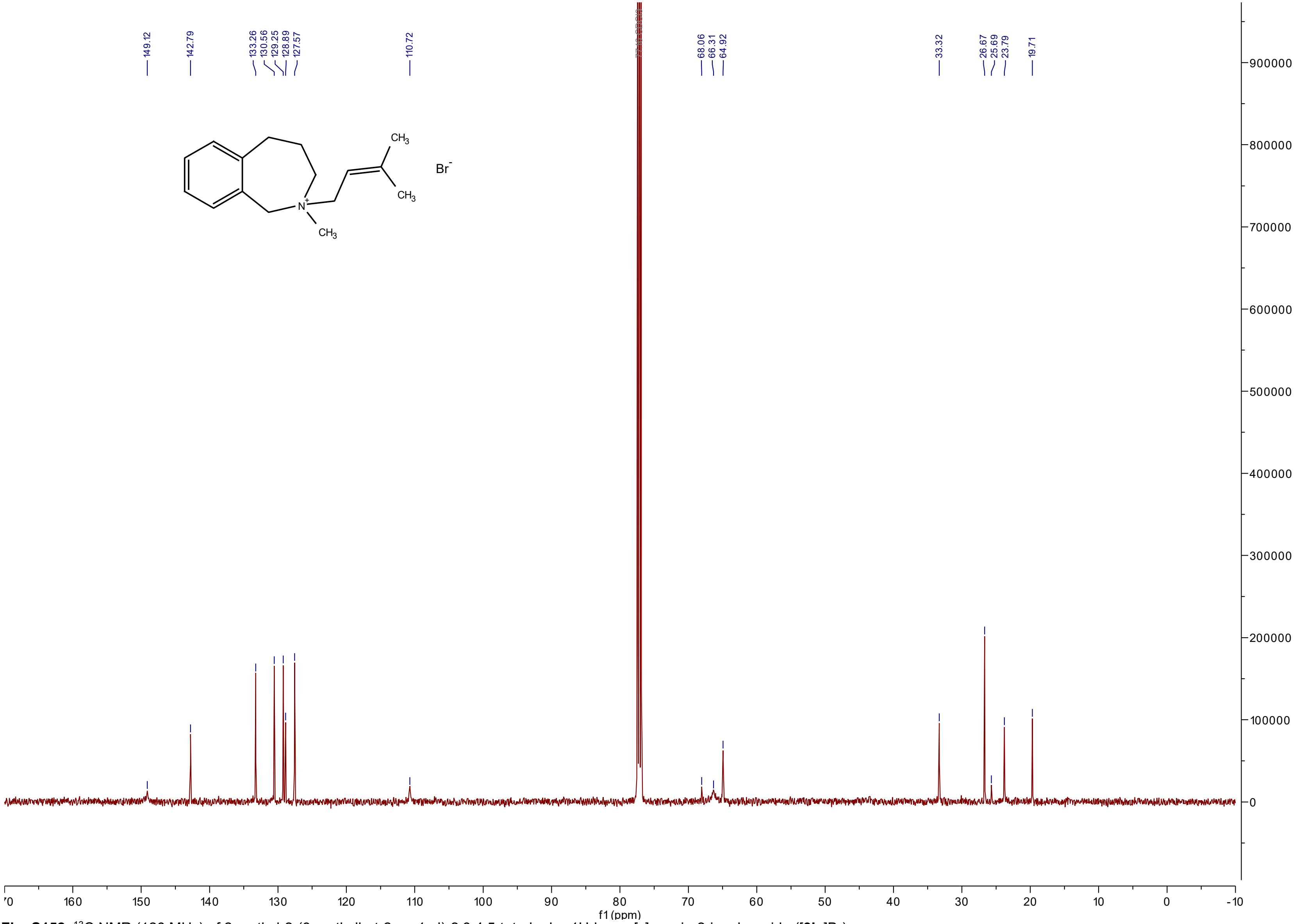

Fig. S152. ^{13}C NMR (126 MHz) of 2-methyl-2-(3-methylbut-2-en-1-yl)-2,3,4,5-tetrahydro-1H-benzo[c]azepin-2-ium bromide ([**3ja**]Br).

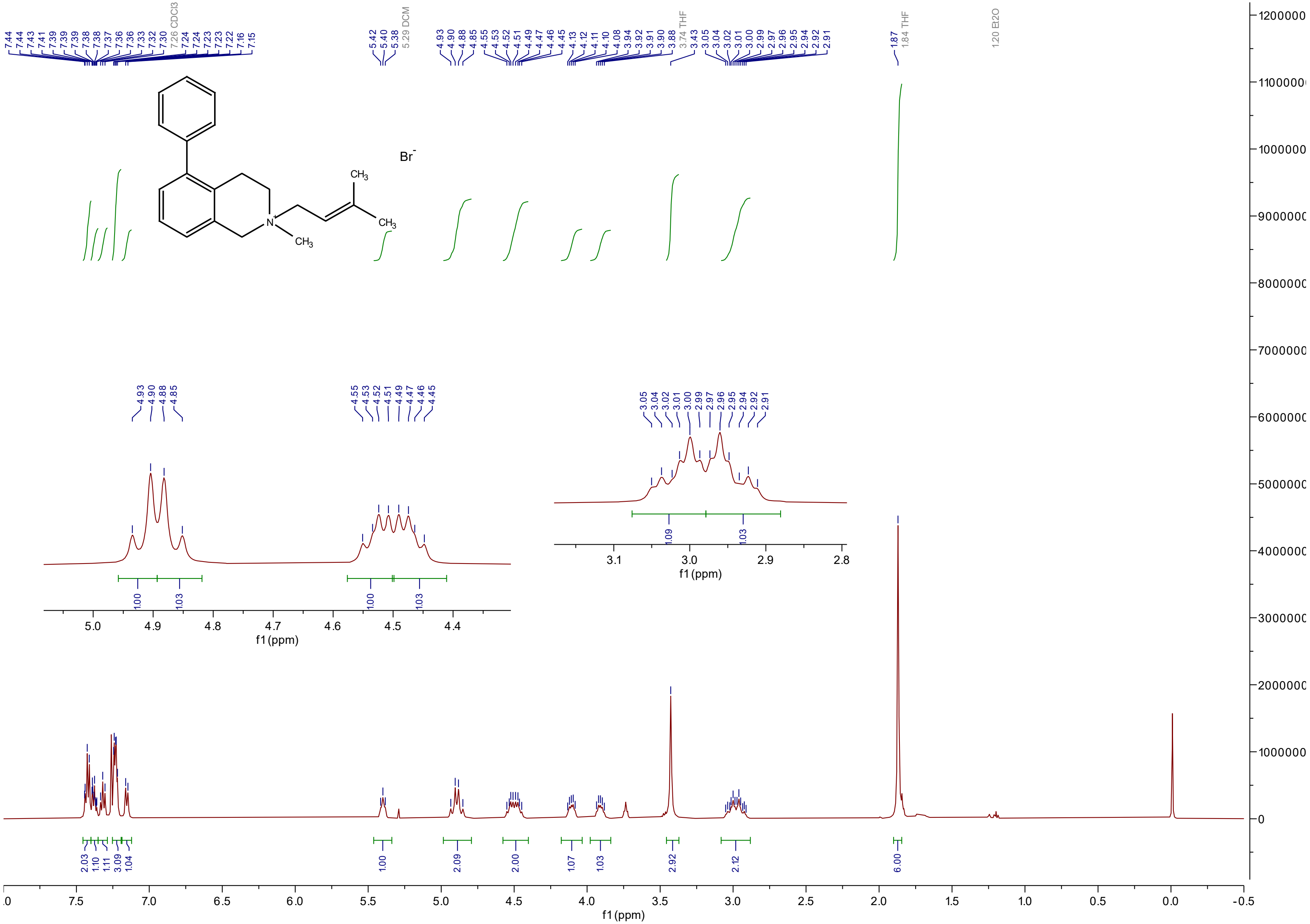

Fig. S153. ^{1}H NMR (500 MHz) of 2-methyl-2-(3-methylbut-2-en-1-yl)-5-phenyl-1,2,3,4-tetrahydroisoquinolin-2-ium bromide ([**3la**]Br).

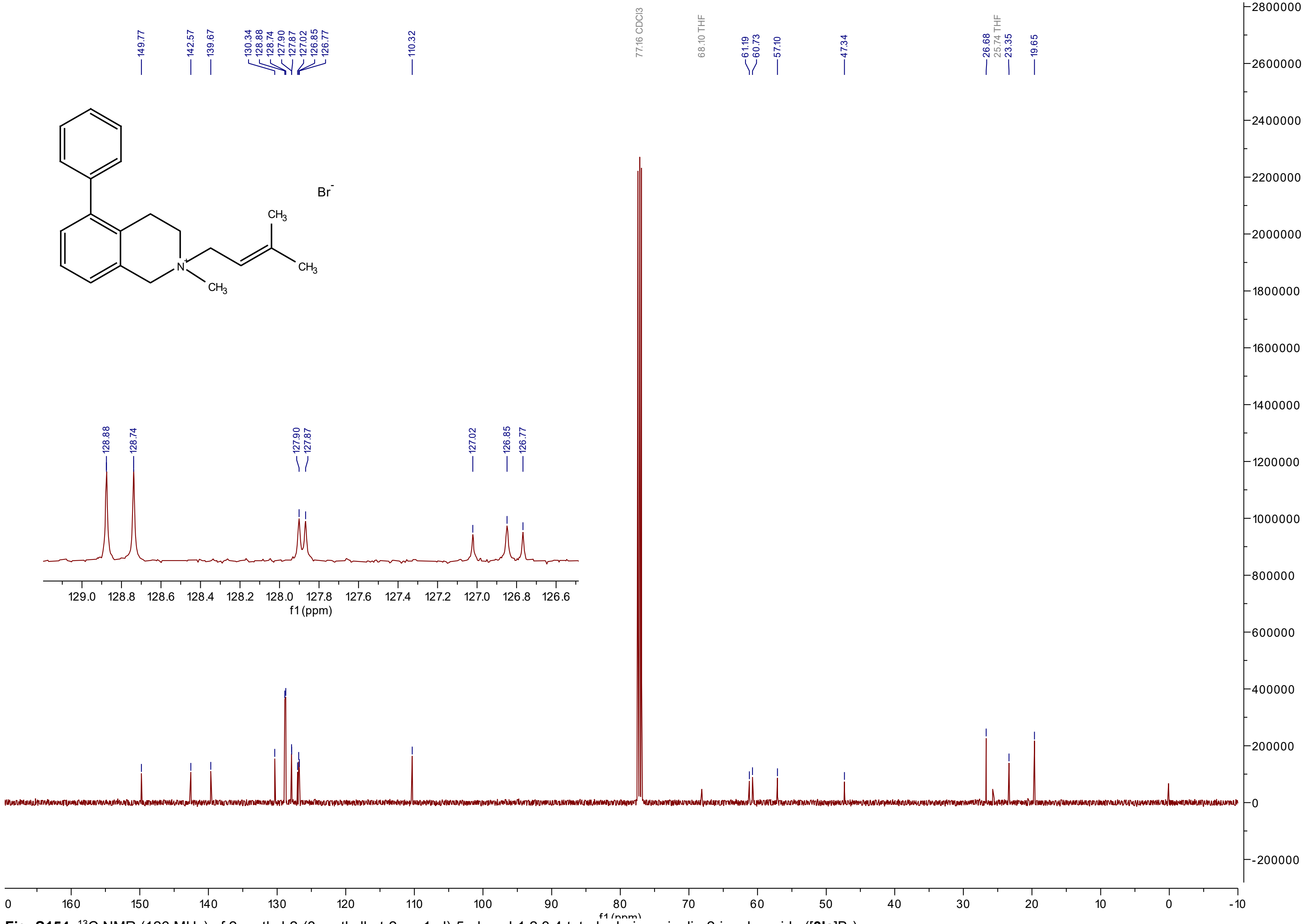

Fig. S154. ^{13}C NMR (126 MHz) of 2-methyl-2-(3-methylbut-2-en-1-yl)-5-phenyl-1,2,3,4-tetrahydroisoquinolin-2-ium bromide ([**3la**]Br).

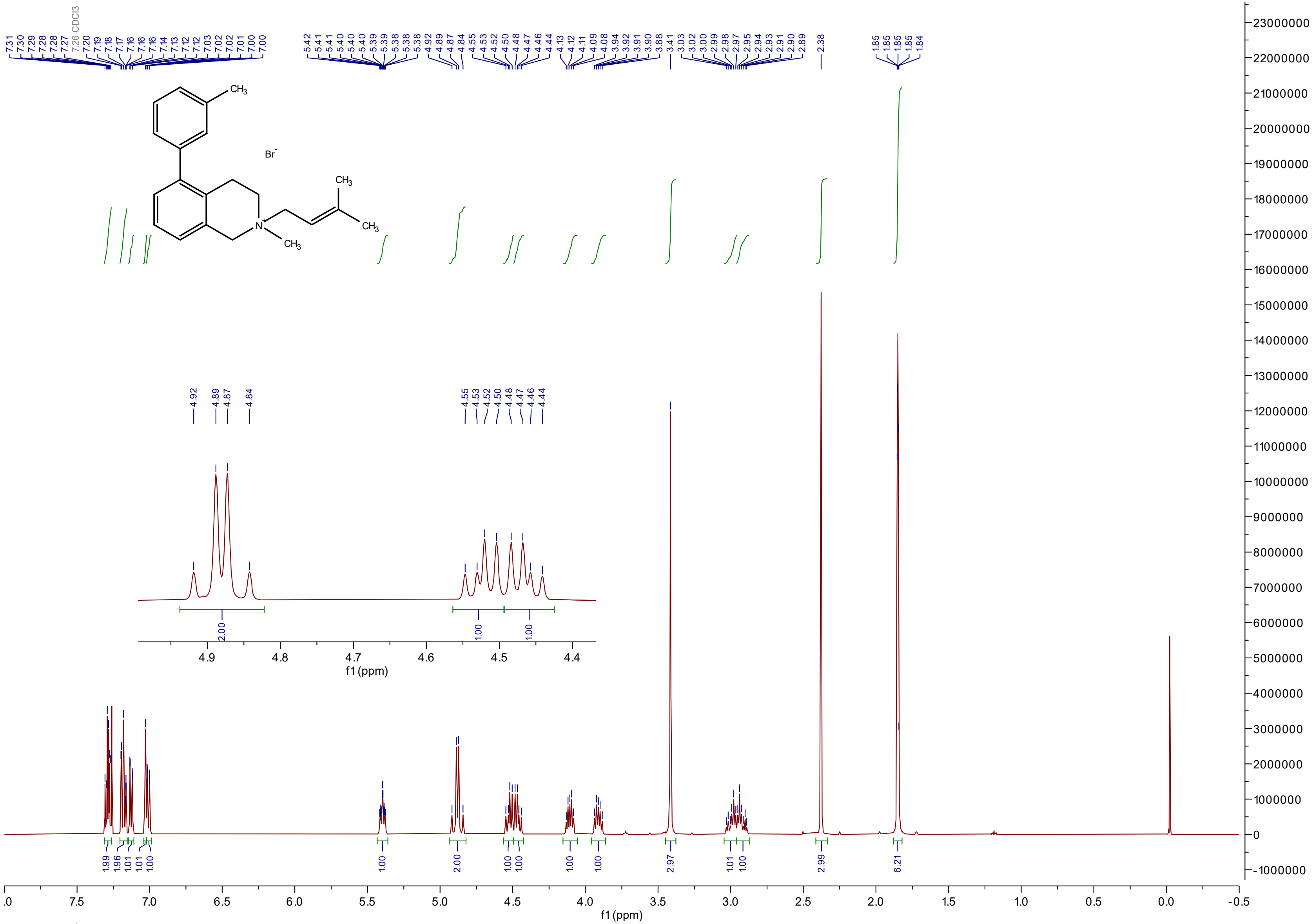

Fig. S155. ^{1}H NMR (500 MHz) of 2-methyl-2-(3-methylbut-2-en-1-yl)-5-(*m*-tolyl)-1,2,3,4-tetrahydroisoquinolin-2-ium bromide ([**3ma**]Br).

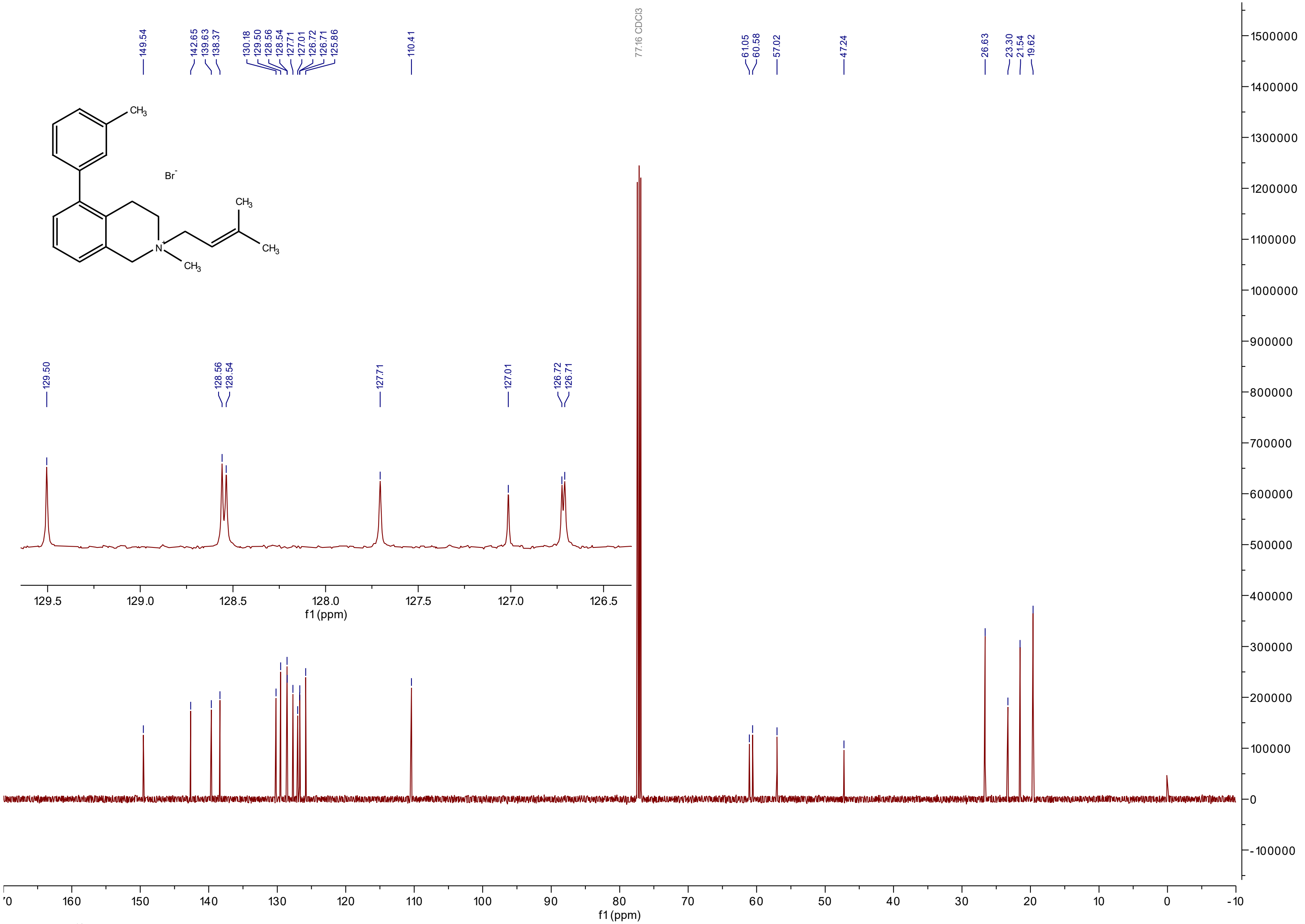

Fig. S156. ^{13}C NMR (126 MHz) of 2-methyl-2-(3-methylbut-2-en-1-yl)-5-(*m*-tolyl)-1,2,3,4-tetrahydroisoquinolin-2-ium bromide ([**3ma**]Br).

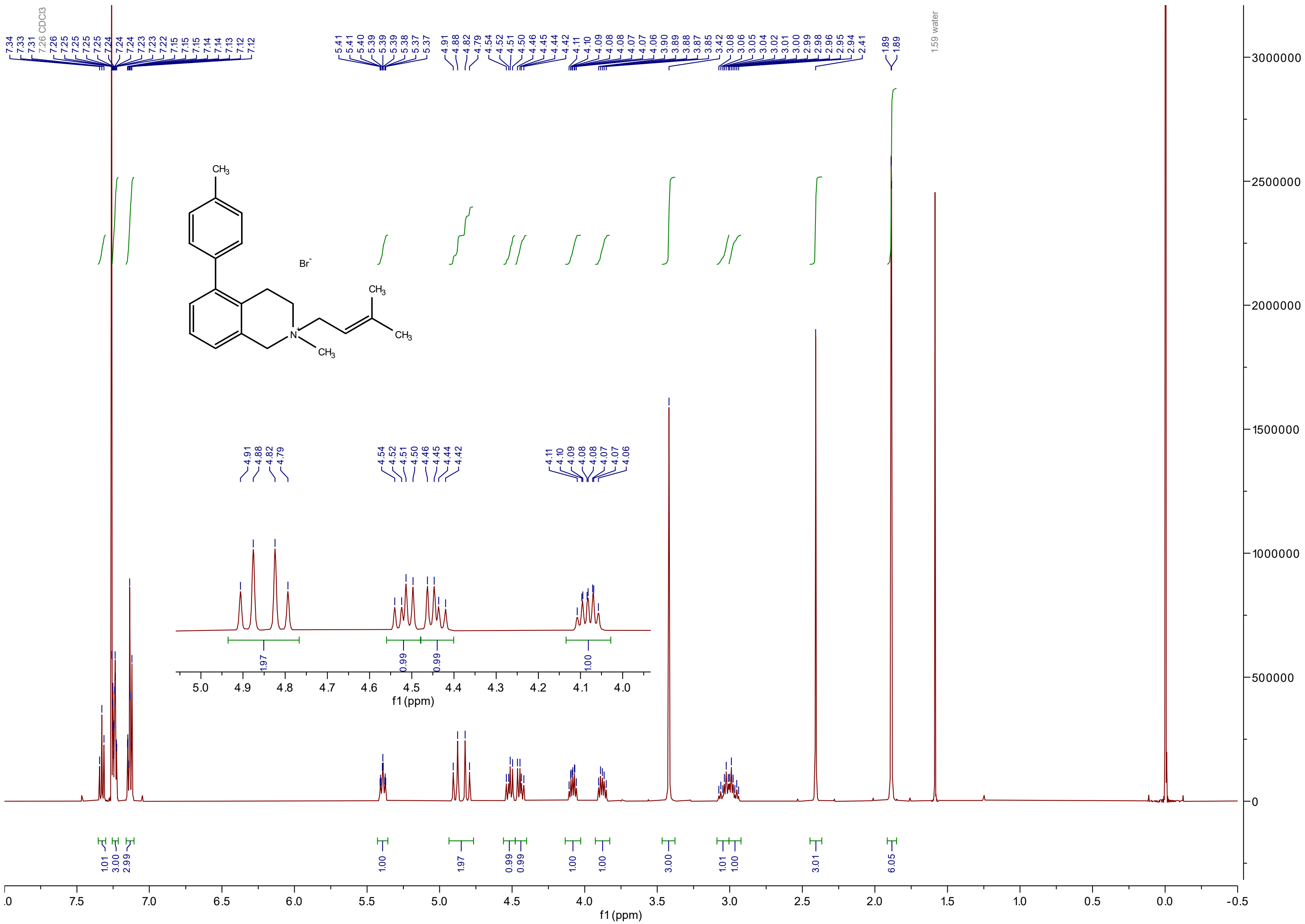

Fig. S157. ^{1}H NMR (500 MHz) of 2-methyl-2-(3-methylbut-2-en-1-yl)-5-(p-tolyl)-1,2,3,4-tetrahydroisoquinolin-2-ium bromide ([**3na**]Br).

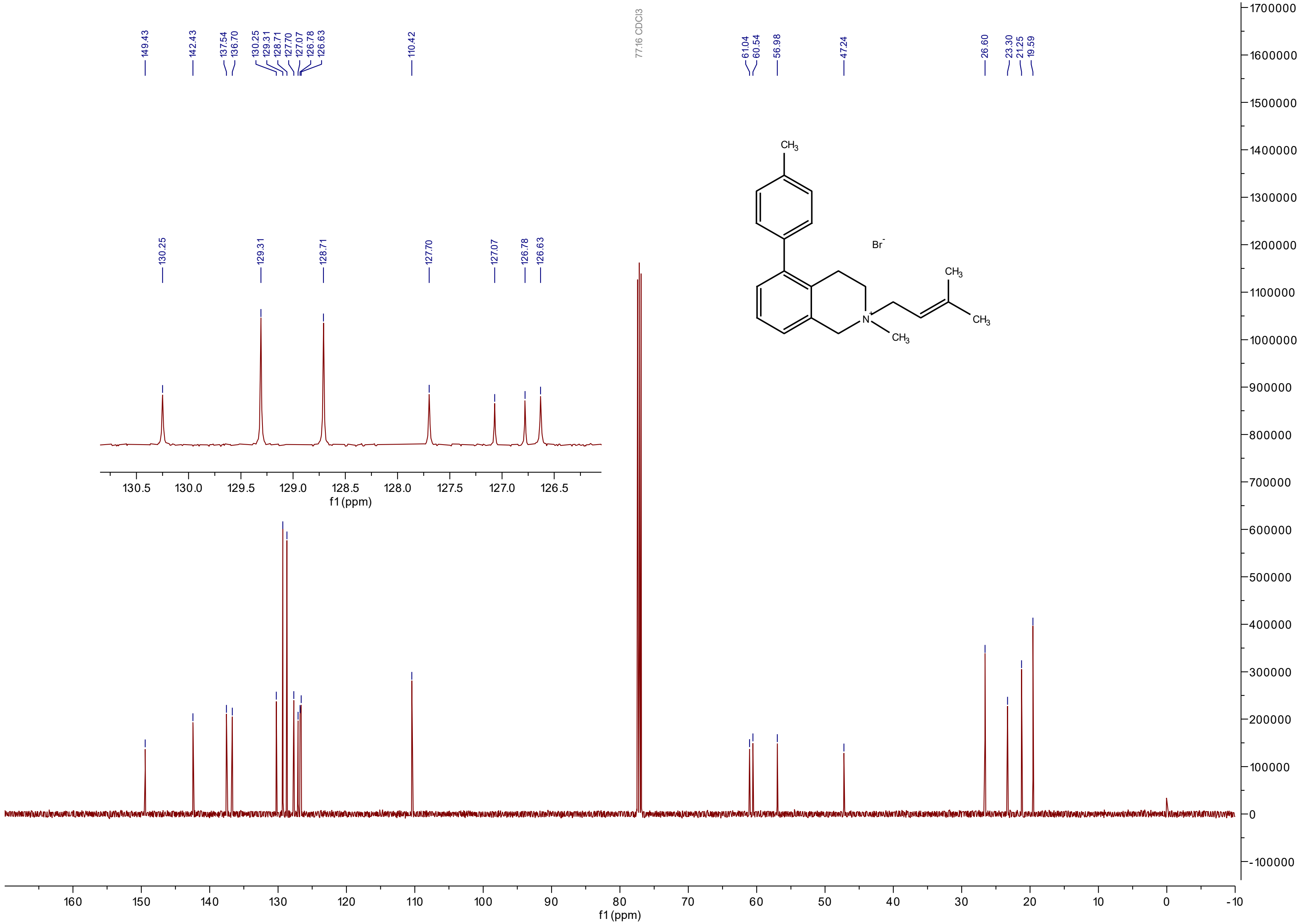

Fig. S158. ^{13}C NMR (126 MHz) of 2-methyl-2-(3-methylbut-2-en-1-yl)-5-(p-tolyl)-1,2,3,4-tetrahydroisoquinolin-2-ium bromide ([**3na**]Br).

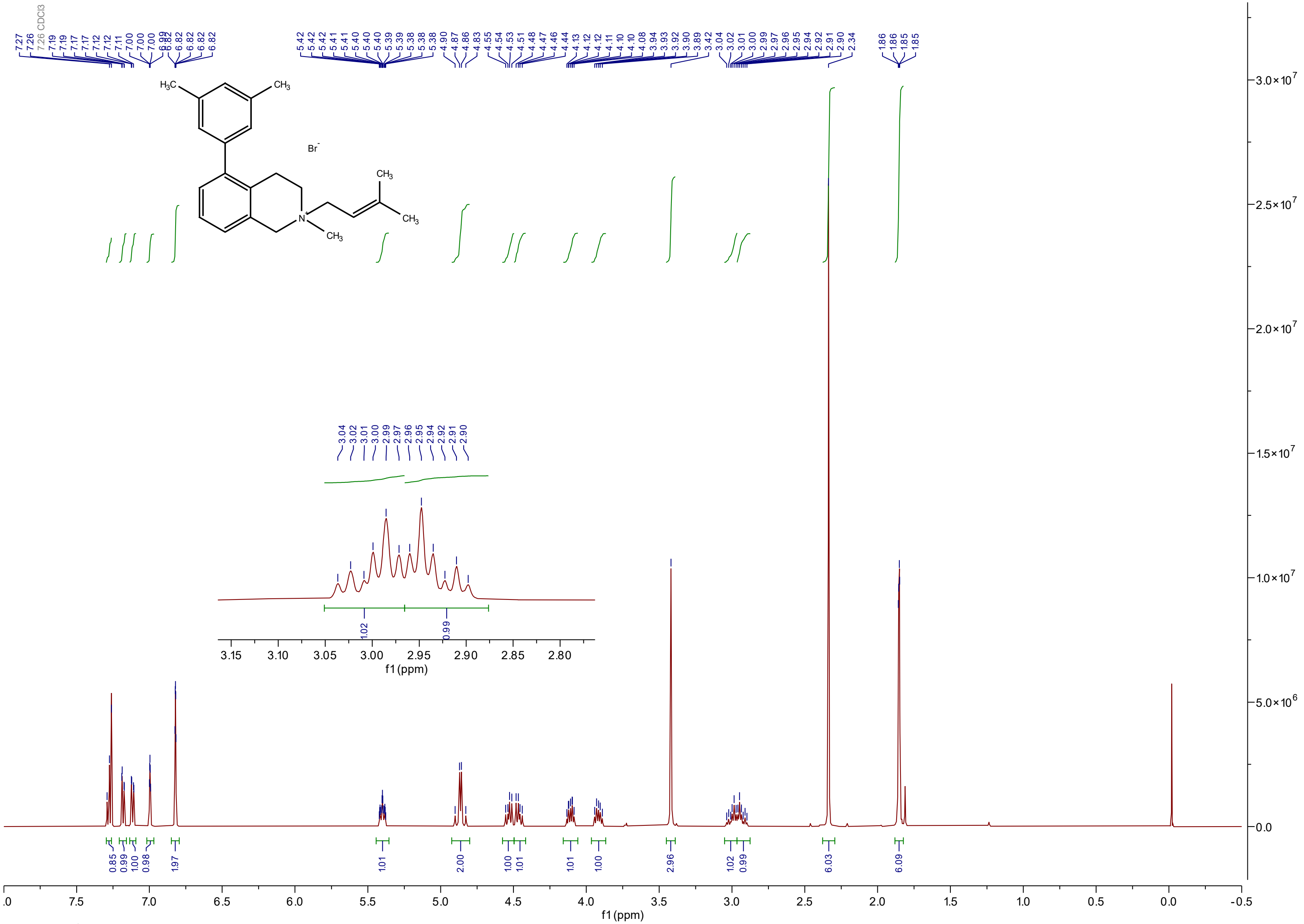

Fig. S159. ^{1}H NMR (500 MHz) of 5-(3,5-dimethyl)-2-methyl-2-(3-methylbut-2-en-1-yl)-1,2,3,4-tetrahydroisoquinolin-2-ium bromide ([**3oa**]Br).

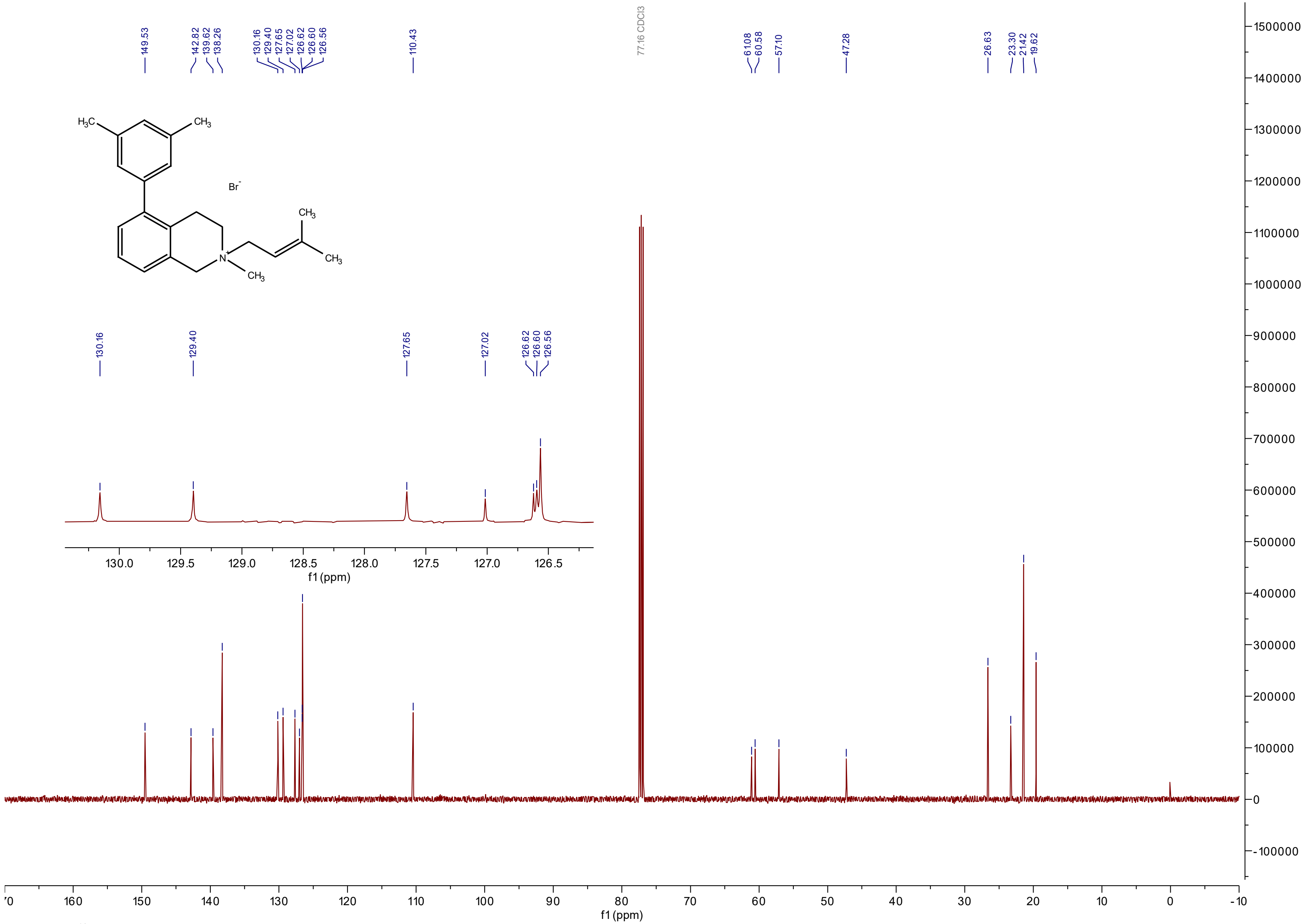

Fig. S160. ^{13}C NMR (126 MHz) of 5-(3,5-dimethyl)-2-methyl-2-(3-methylbut-2-en-1-yl)-1,2,3,4-tetrahydroisoquinolin-2-ium bromide ([**3oa**]Br).

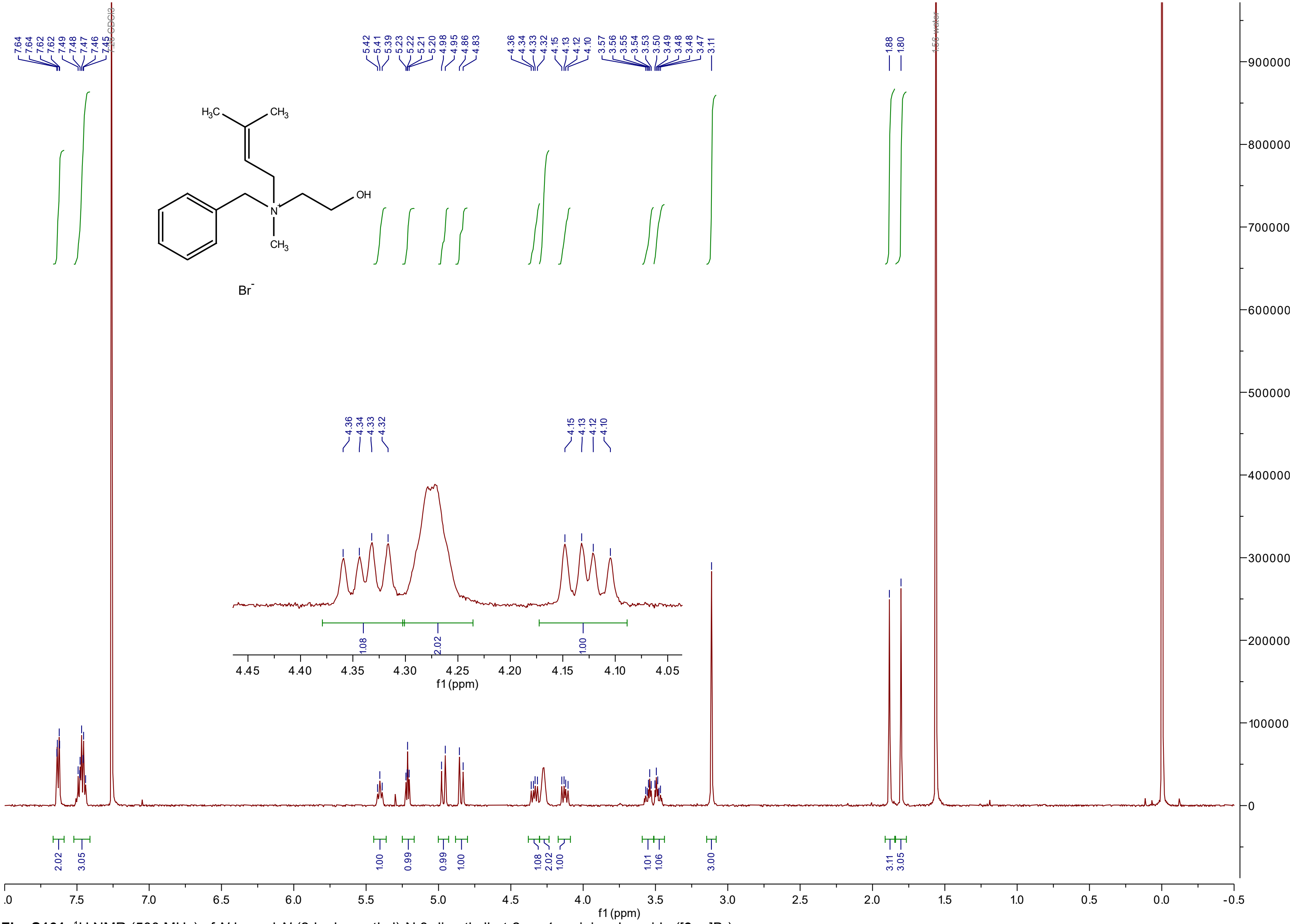

Fig. S161. ^{1}H NMR (500 MHz) of *N*-benzyl-*N*-(2-hydroxyethyl)-N,3-dimethylbut-2-en-1-aminium bromide ([**3pa**]Br).

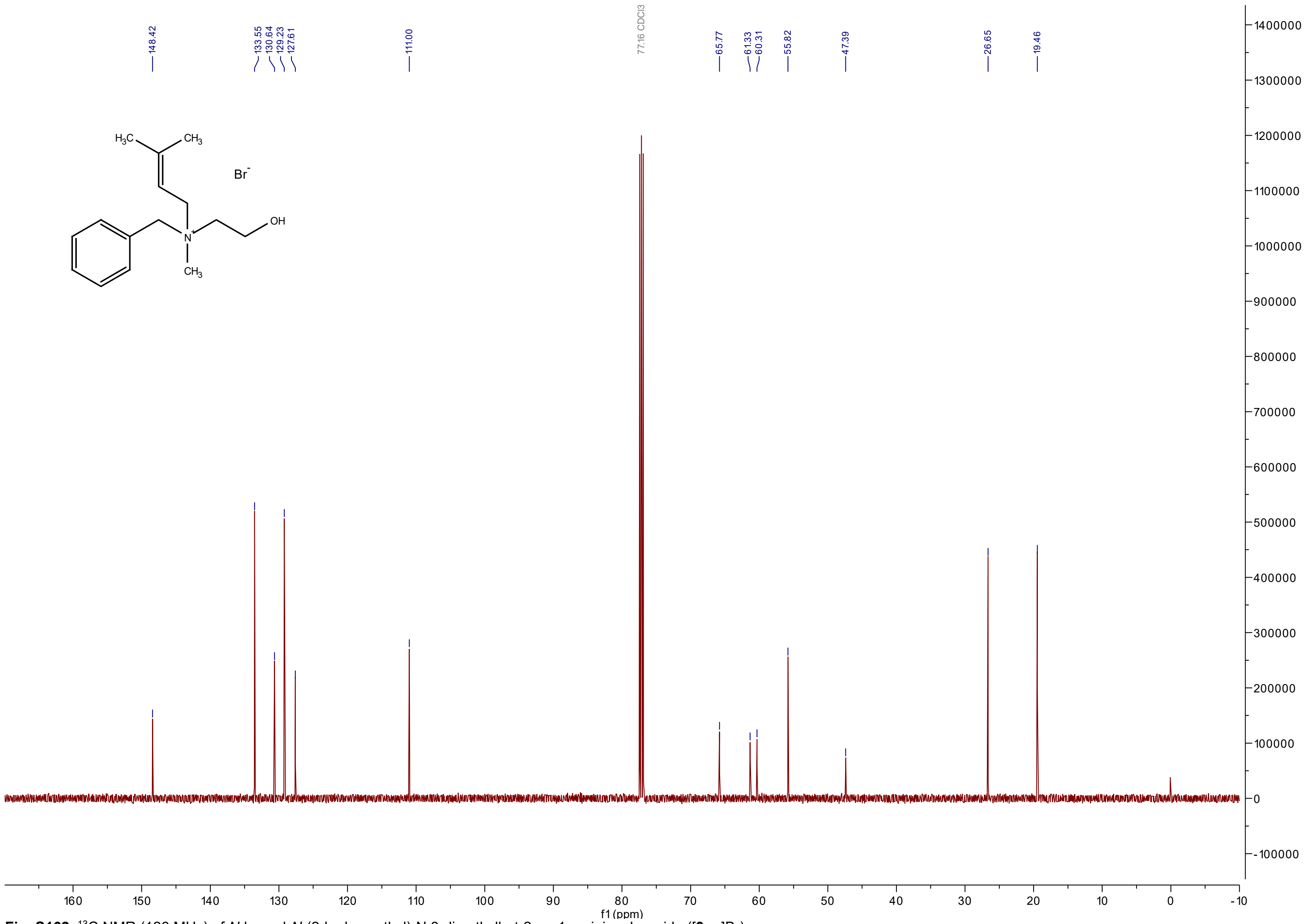

Fig. S162. ^{13}C NMR (126 MHz) of *N*-benzyl-*N*-(2-hydroxyethyl)-N,3-dimethylbut-2-en-1-aminium bromide ([**3pa**]Br).

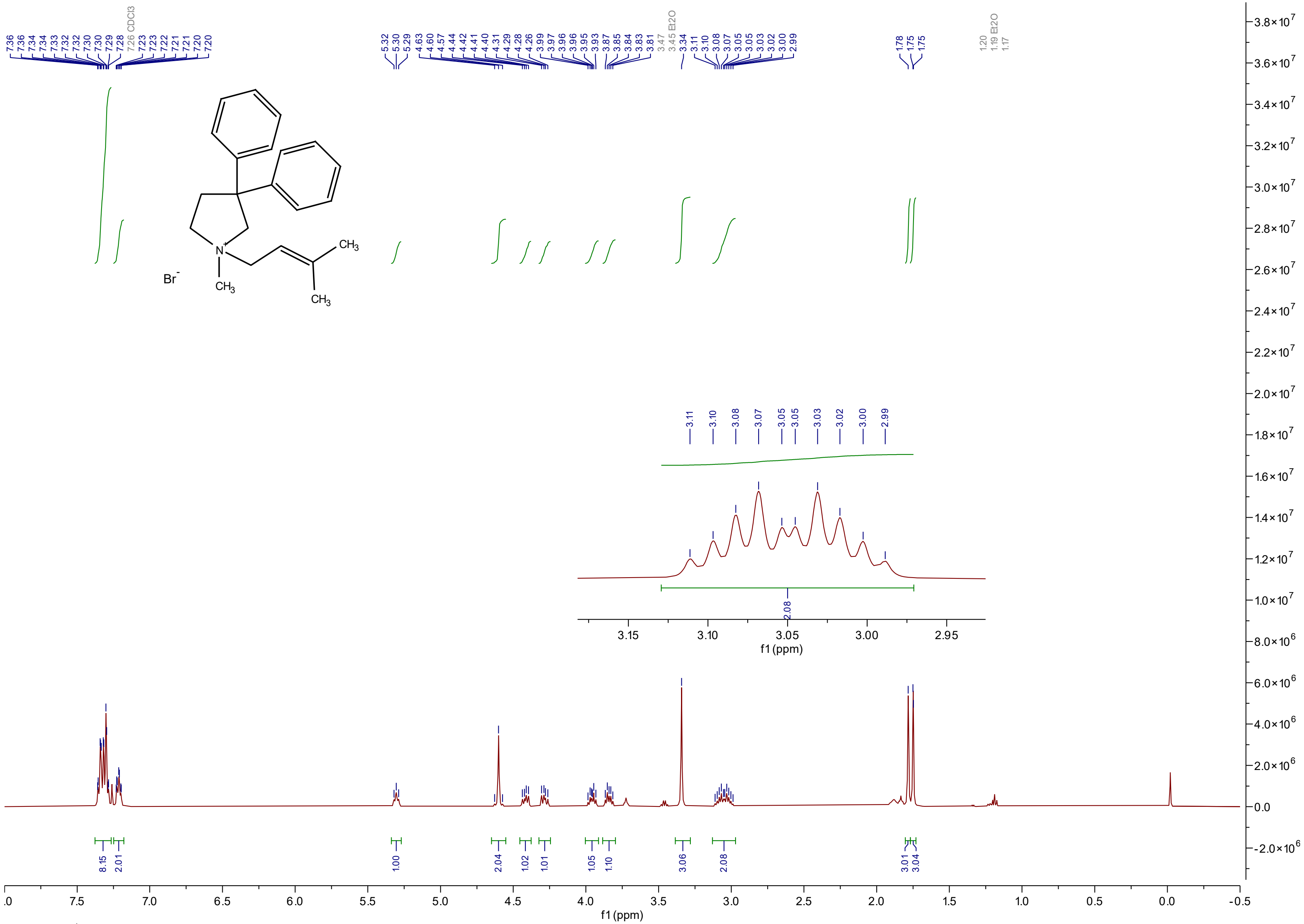

Fig. S163. ^{1}H NMR (500 MHz) of 1-methyl-1-(3-methylbut-2-en-1-yl)-3,3-diphenylpyrrolidin-1-ium bromide ([**3qa**]Br).

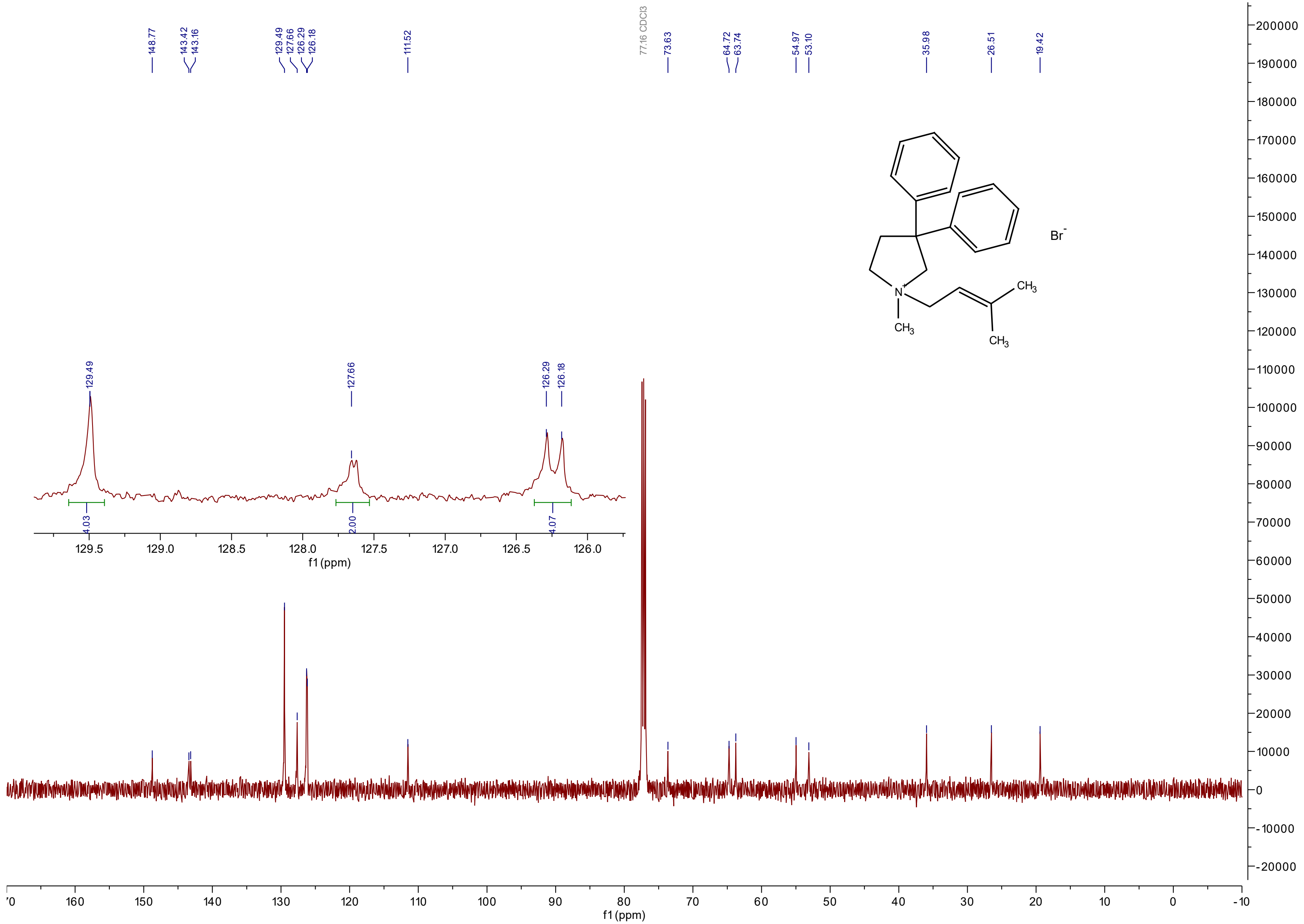

Fig. S164. ^{13}C NMR (126 MHz) of 1-methyl-1-(3-methylbut-2-en-1-yl)-3,3-diphenylpyrrolidin-1-ium bromide ([**3qa**]Br).

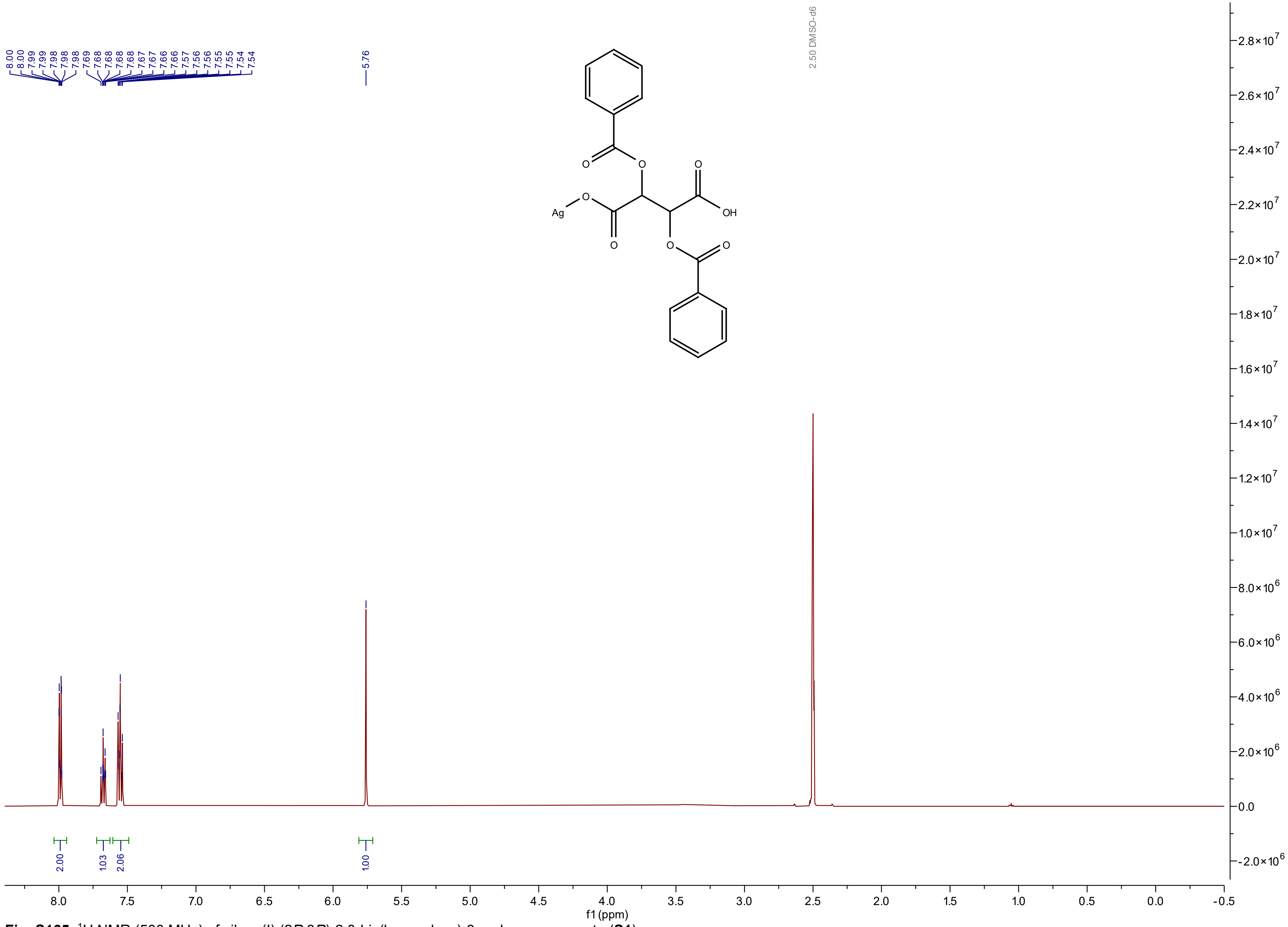

Fig. S165. ^{1}H NMR (500 MHz) of silver (I) (2*R*,3*R*)-2,3-bis(benzoyloxy)-3-carboxypropanoate (**S1**).

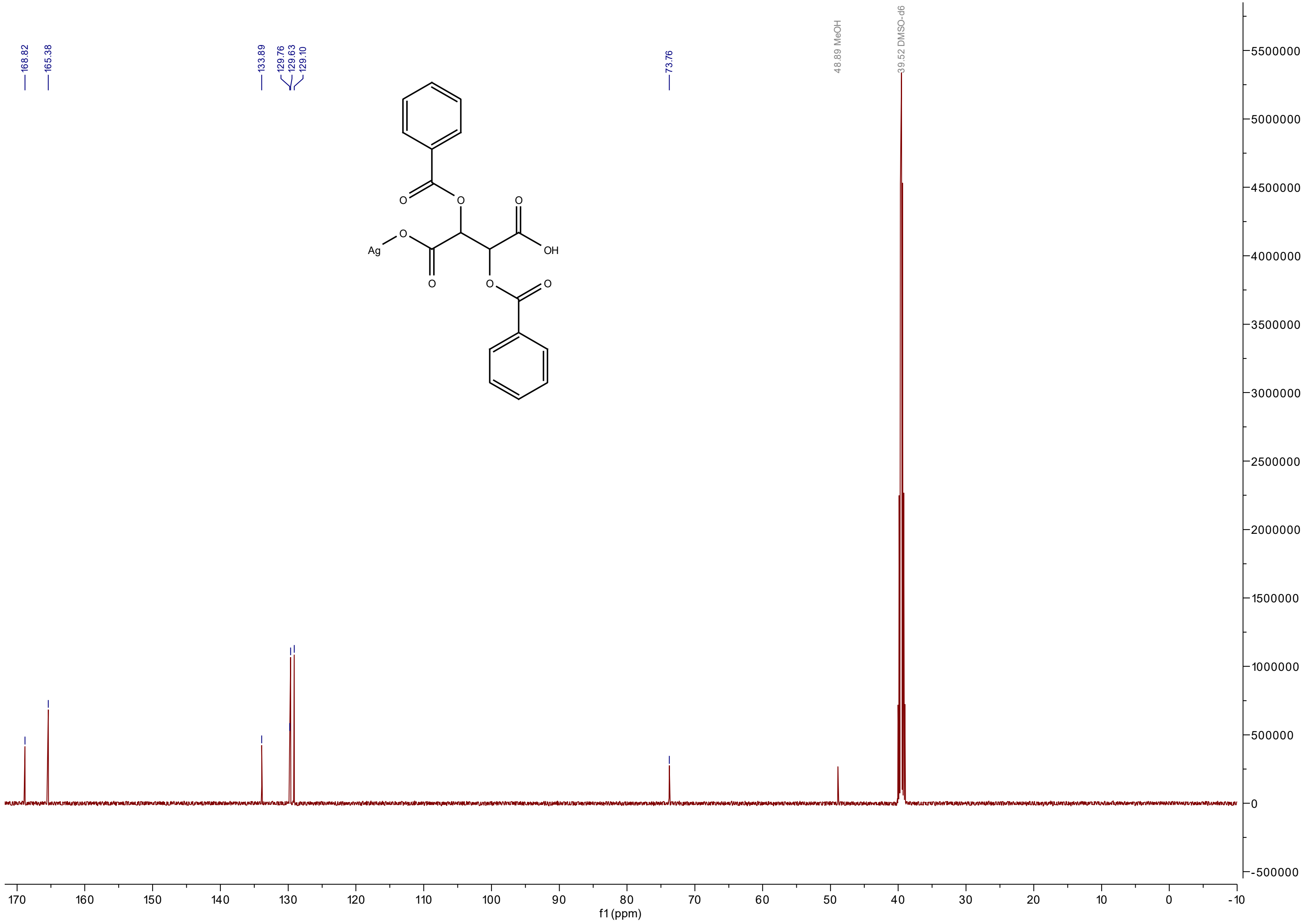

Fig. S166. ^{13}C NMR (126 MHz) of silver (I) (2*R*,3*R*)-2,3-bis(benzoyloxy)-3-carboxypropanoate (**S1**).

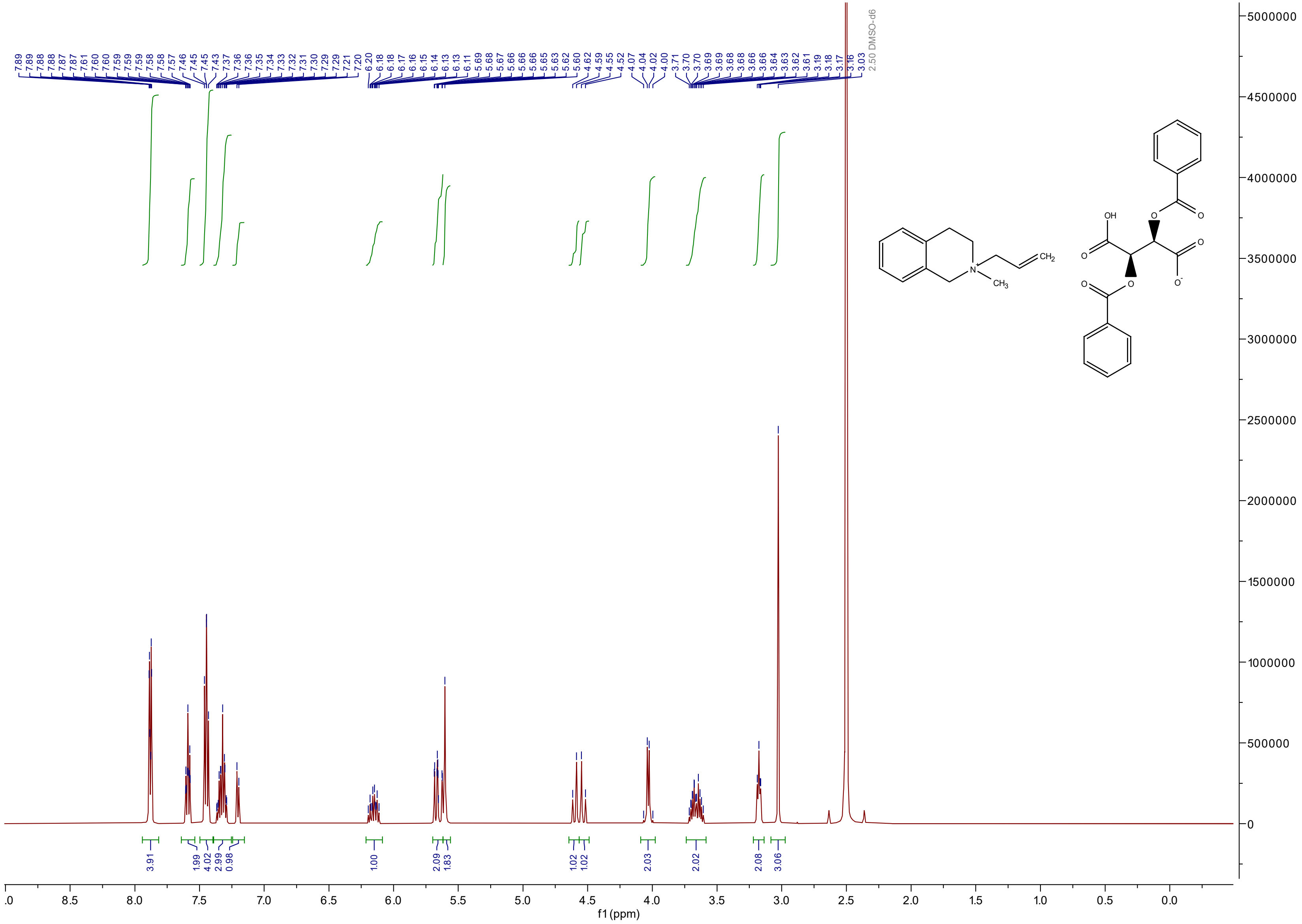

Fig. S167. ^{1}H NMR (500 MHz) of 2-allyl-2-methyl-1,2,3,4-tetrahydroisoquinolin-2-ium (2*R*,3*R*)-2,3-bis(benzoyloxy)-3-carboxypropanoate (**S2**).

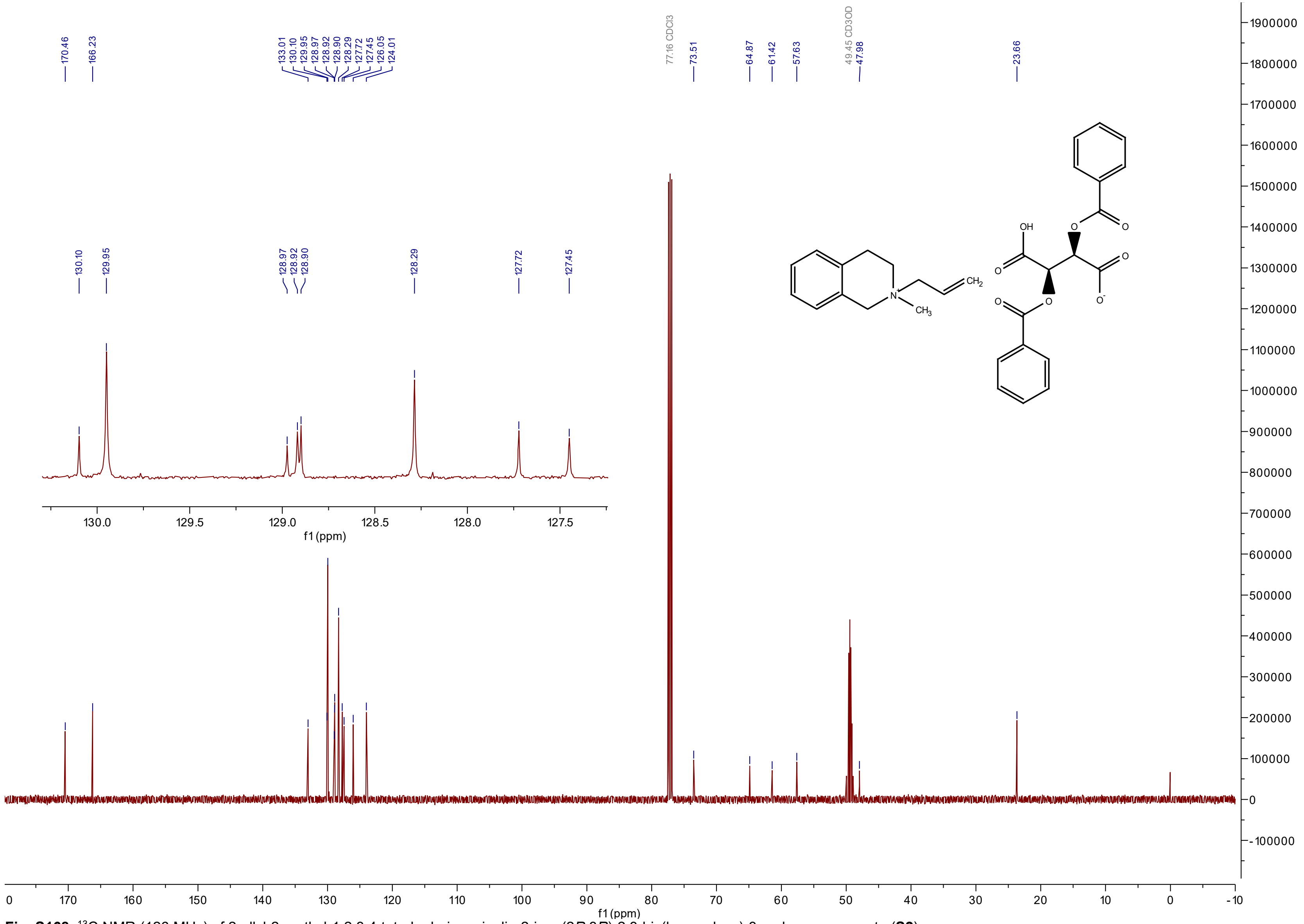

Fig. S168. ^{13}C NMR (126 MHz) of 2-allyl-2-methyl-1,2,3,4-tetrahydroisoquinolin-2-ium (2*R*,3*R*)-2,3-bis(benzoyloxy)-3-carboxypropanoate (**S2**).

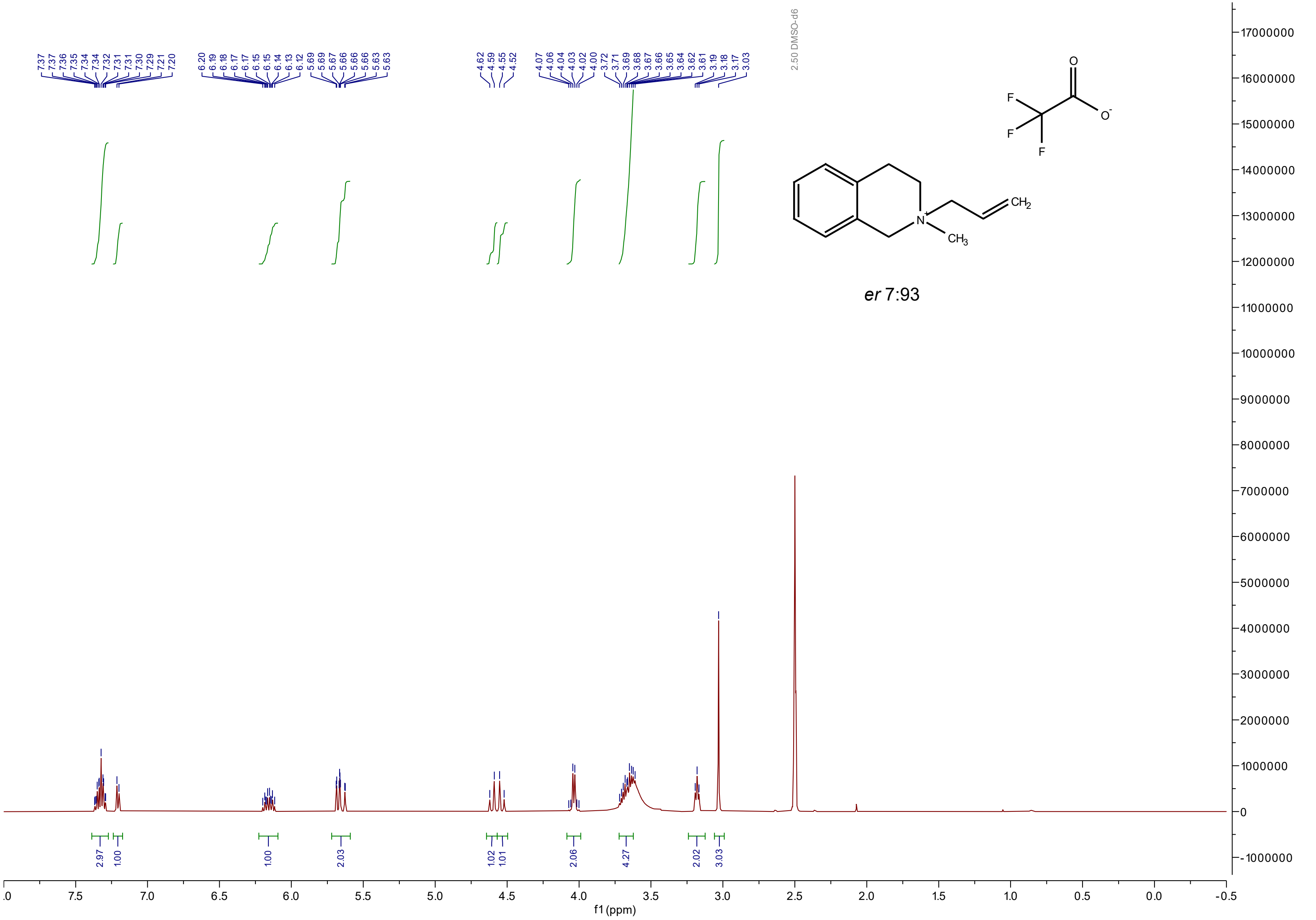

Fig. S169. ^{1}H NMR (500 MHz) of enantioenriched ammonium salt [**3ab**][CF_3CO_2], prepared by fractional crystallization of the salt **S2** and subsequent counteranion exchange.

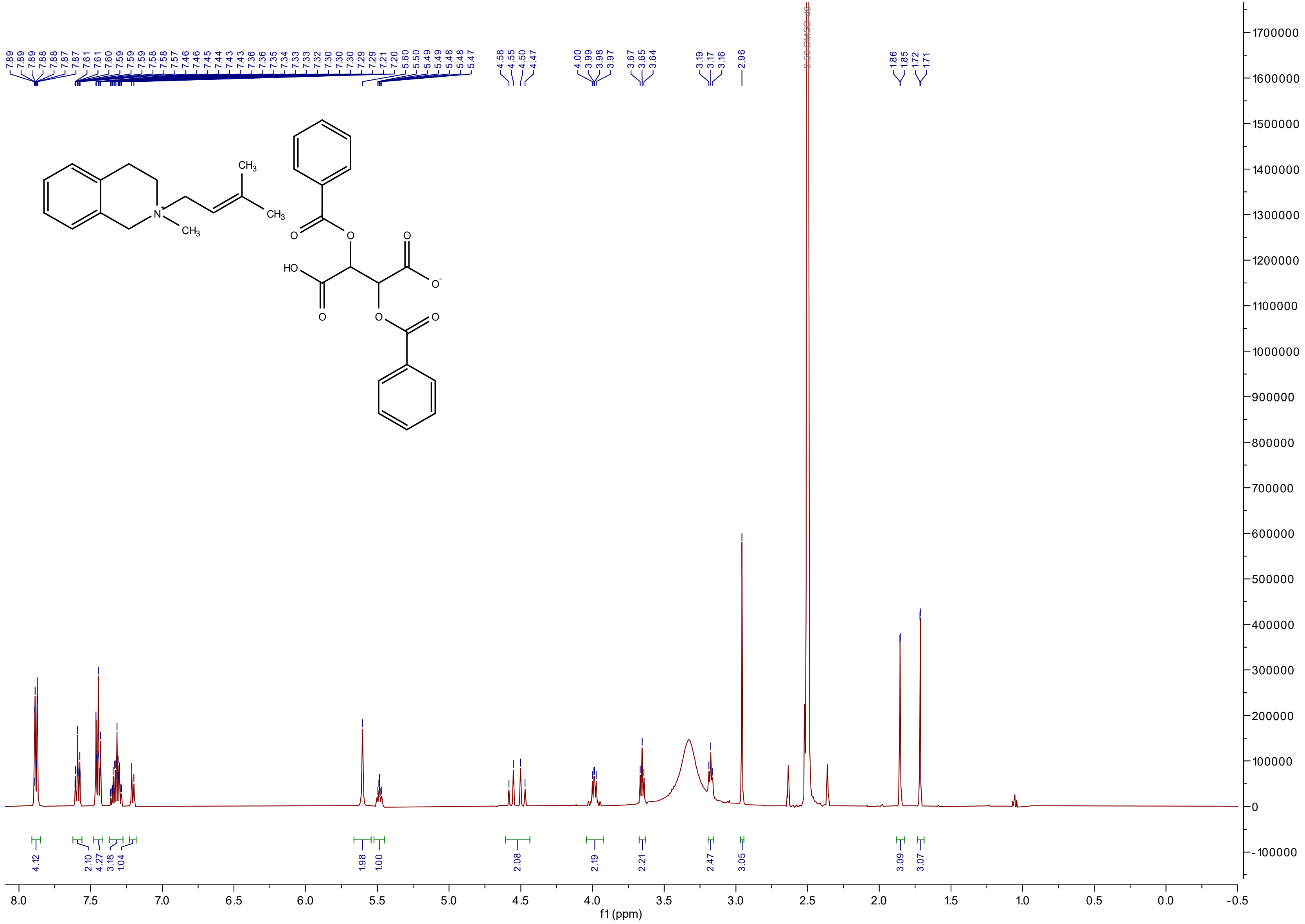

Fig. S170. ^{1}H NMR (500 MHz) of 2-methyl-2-(3-methylbut-2-en-1-yl)-1,2,3,4-tetrahydroisoquinolin-2-ium (2*R*,3*R*)-2,3-bis(benzoyloxy)-3-carboxypropanoate (**S3**).

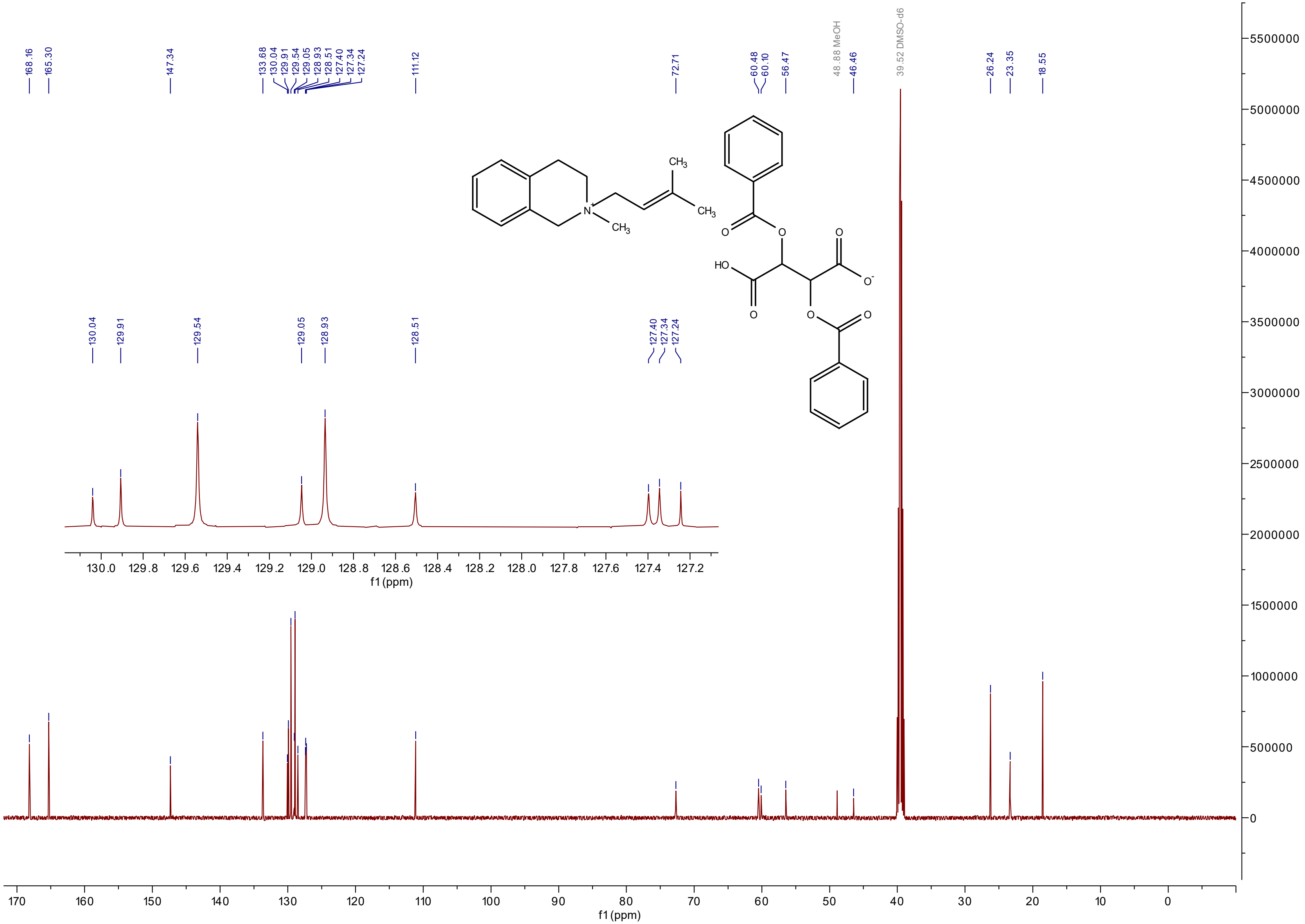

Fig. S171. ^{13}C NMR (126 MHz) of 2-methyl-2-(3-methylbut-2-en-1-yl)-1,2,3,4-tetrahydroisoquinolin-2-ium (2*R*,3*R*)-2,3-bis(benzoyloxy)-3-carboxypropanoate (**S3**).

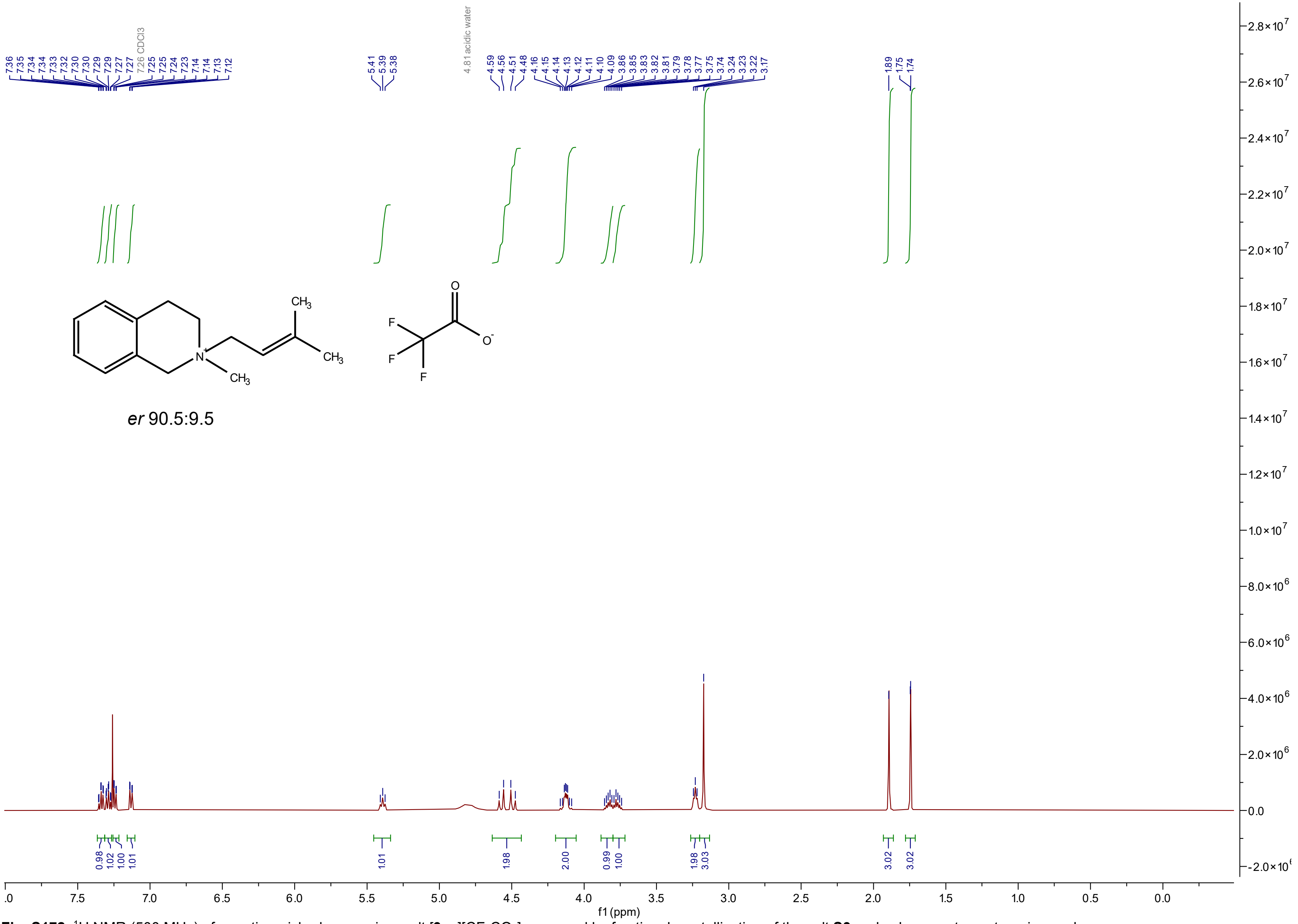

Fig. S172. ^{1}H NMR (500 MHz) of enantioenriched ammonium salt [**3aa**][CF_3CO_2], prepared by fractional crystallization of the salt **S3** and subsequent counteranion exchange.

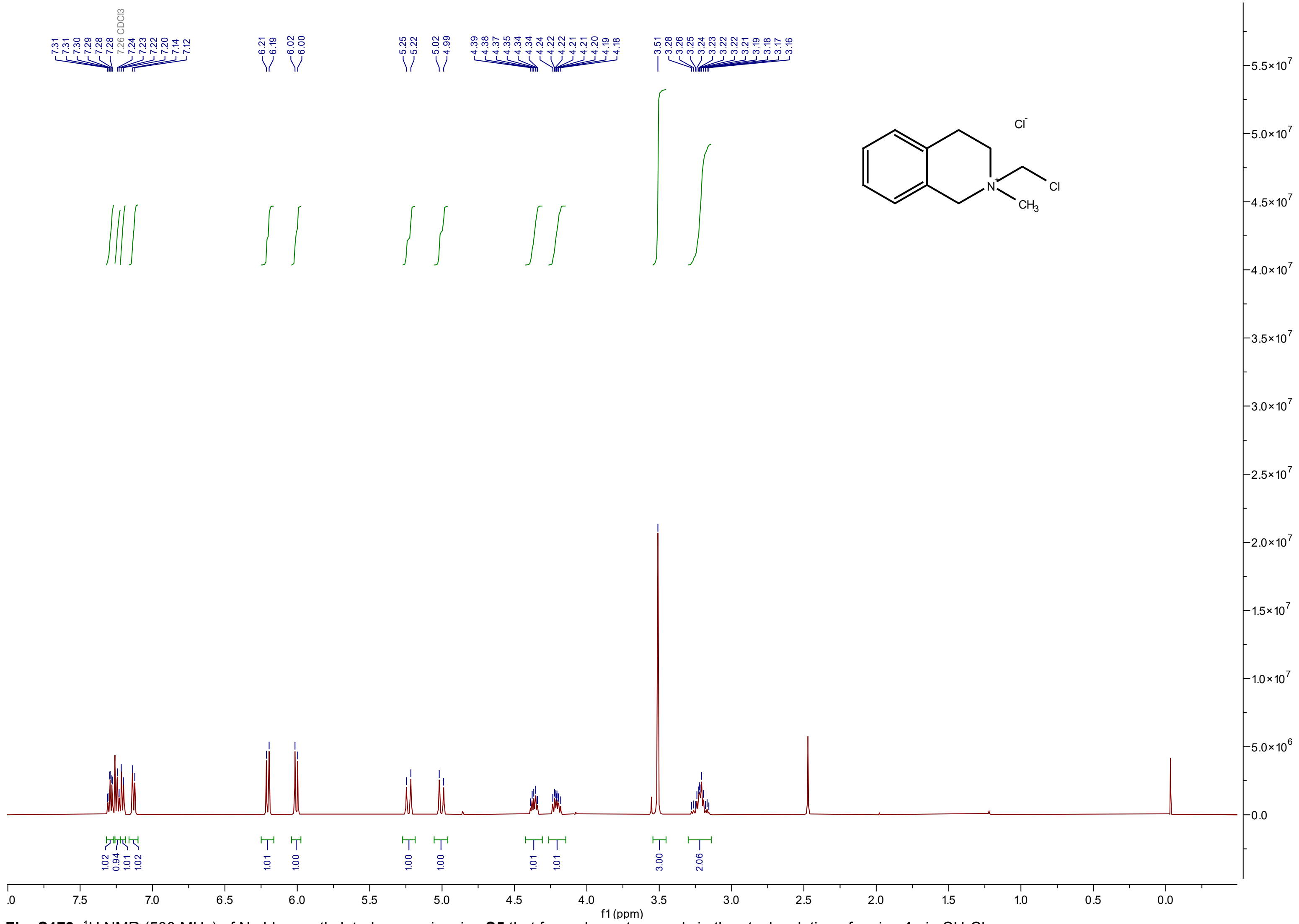

Fig. S173. ^{1}H NMR (500 MHz) of N-chloromethylated ammonium ion **S5** that formed spontaneously in the stock solution of amine **1a** in CH_2Cl_2.

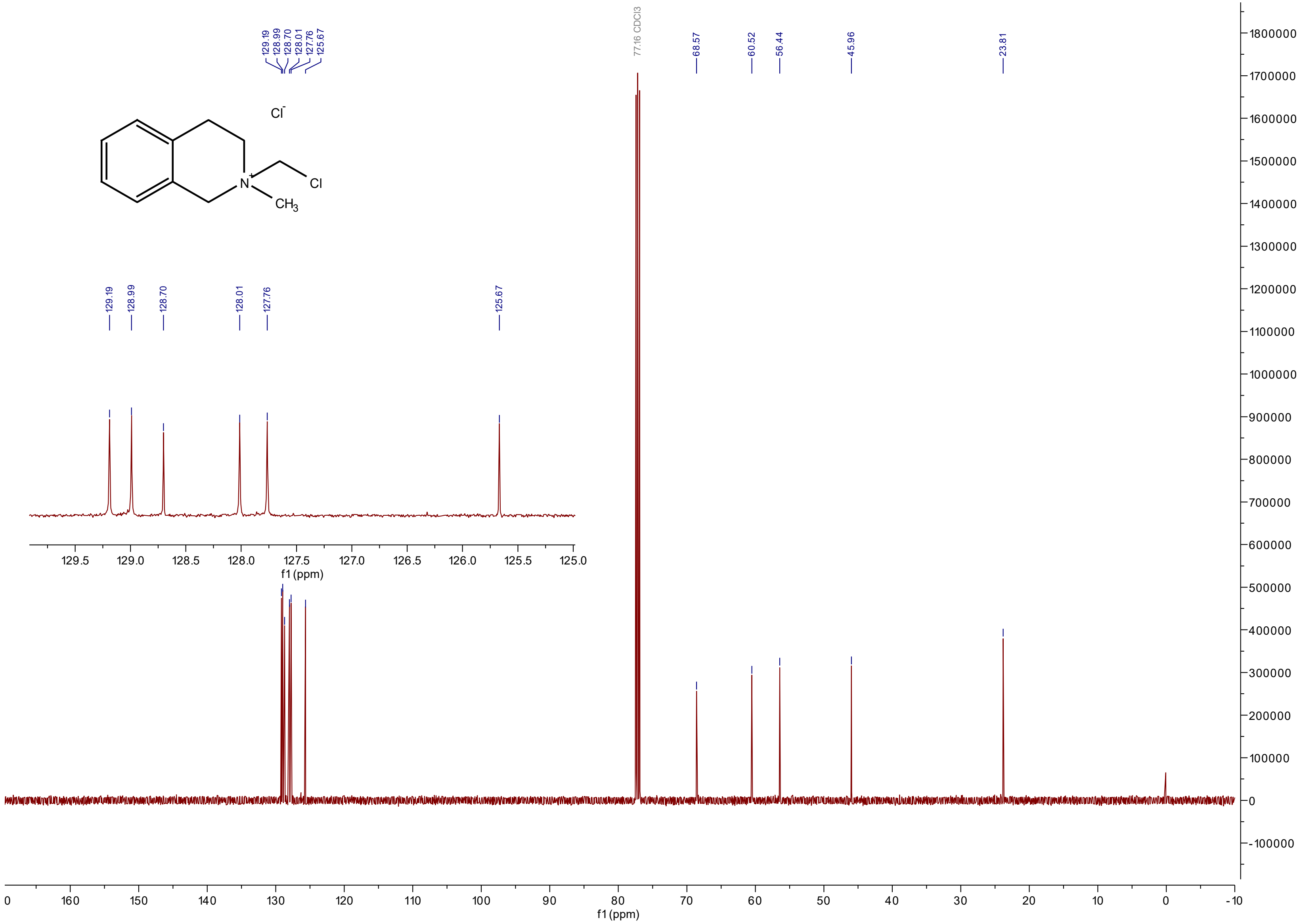

Fig. S174. ^{13}C NMR (126 MHz) of N-chloromethylated ammonium ion **S5** that formed spontaneously in the stock solution of amine **1a** in CH_2Cl_2.

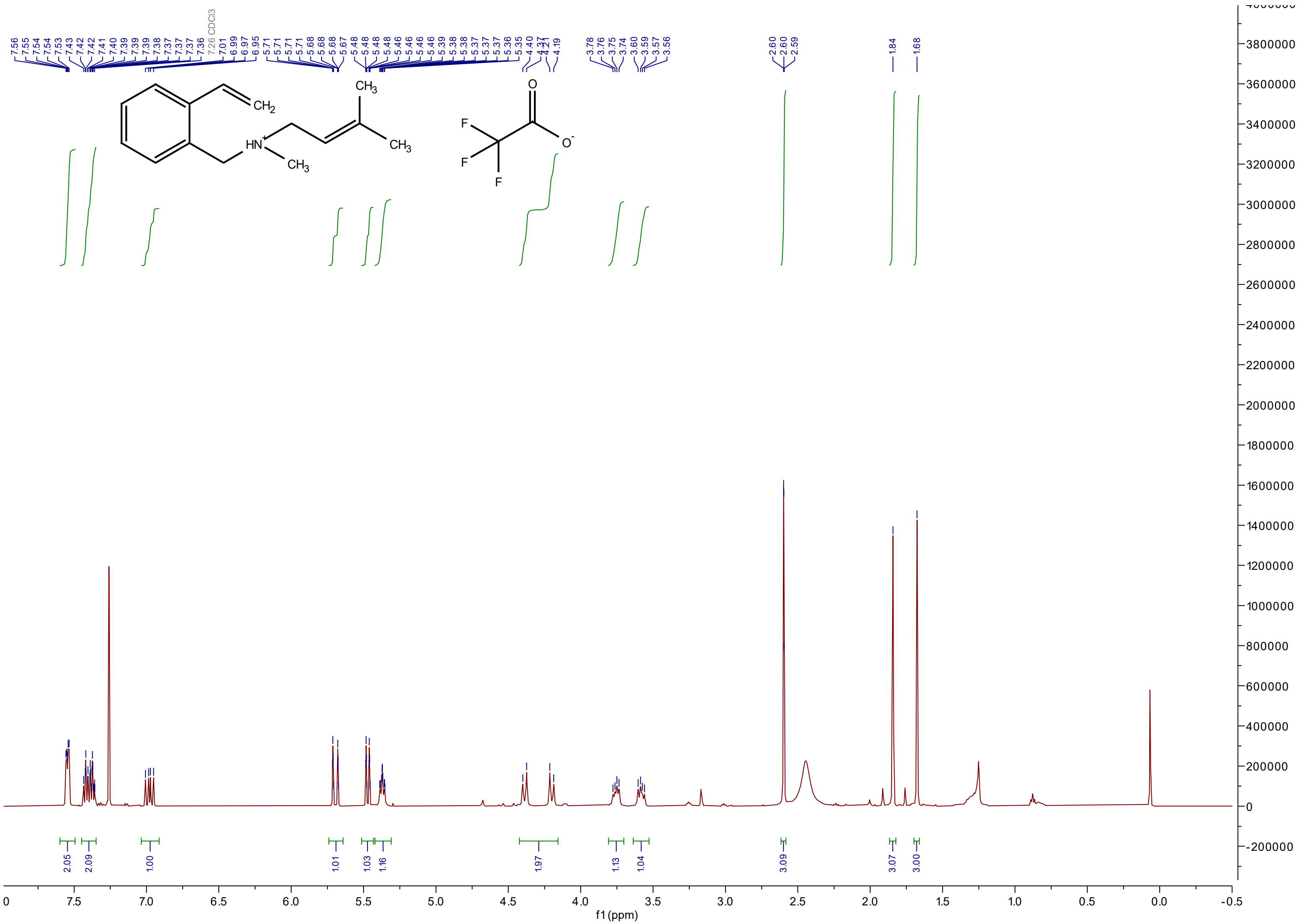

Fig. S175. ^{1}H NMR (500 MHz) of the trifluoroacetate salt of *N*,3-dimethyl-*N*-(2-vinylbenzyl)but-2-en-1-amine (**S4**) isolated from a preparative catalysis run.

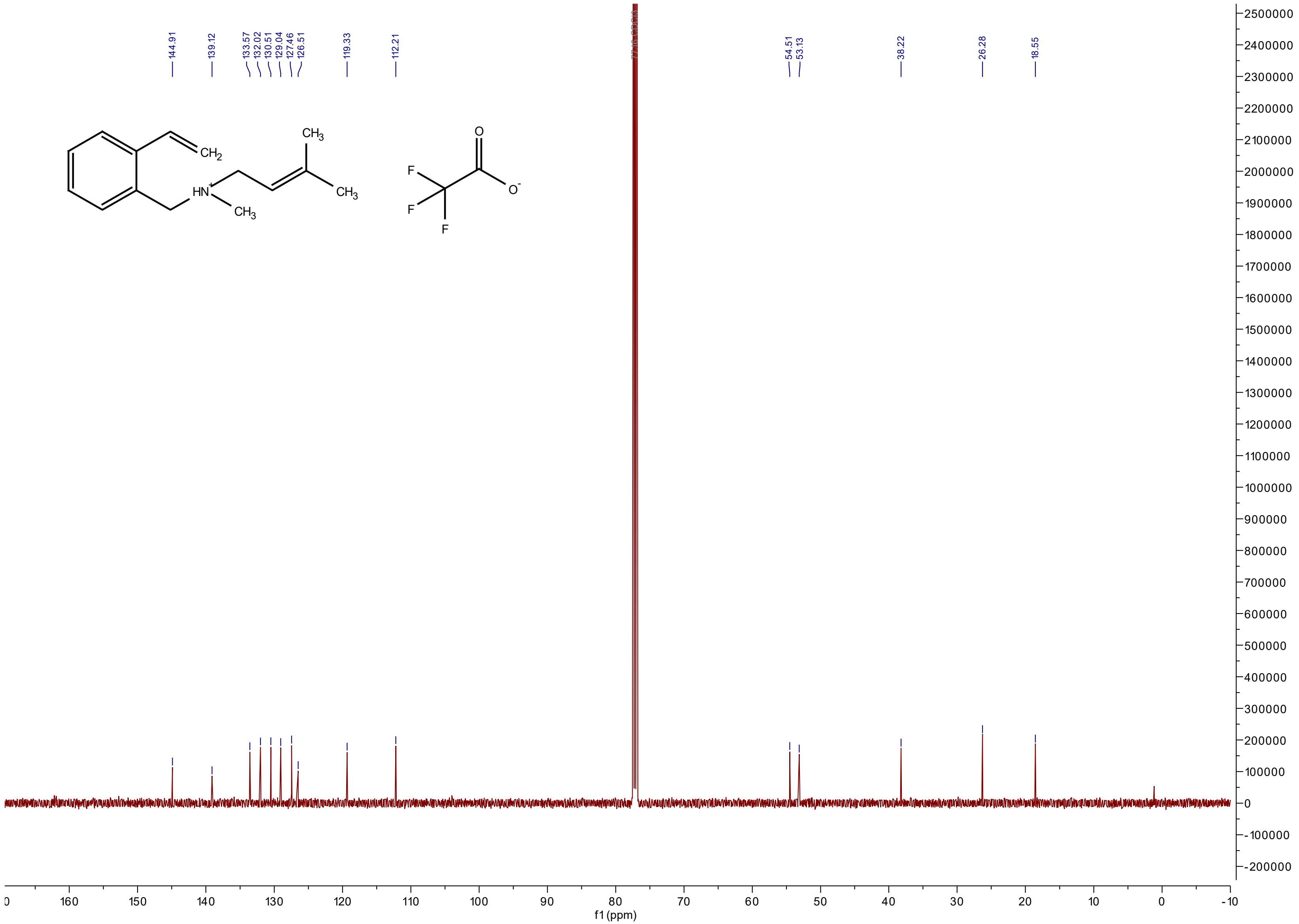

Fig. S176. ^{13}C NMR (126 MHz) of the trifluoroacetate salt of *N*,3-dimethyl-*N*-(2-vinylbenzyl)but-2-en-1-amine (**S4**) isolated from a preparative catalysis run.

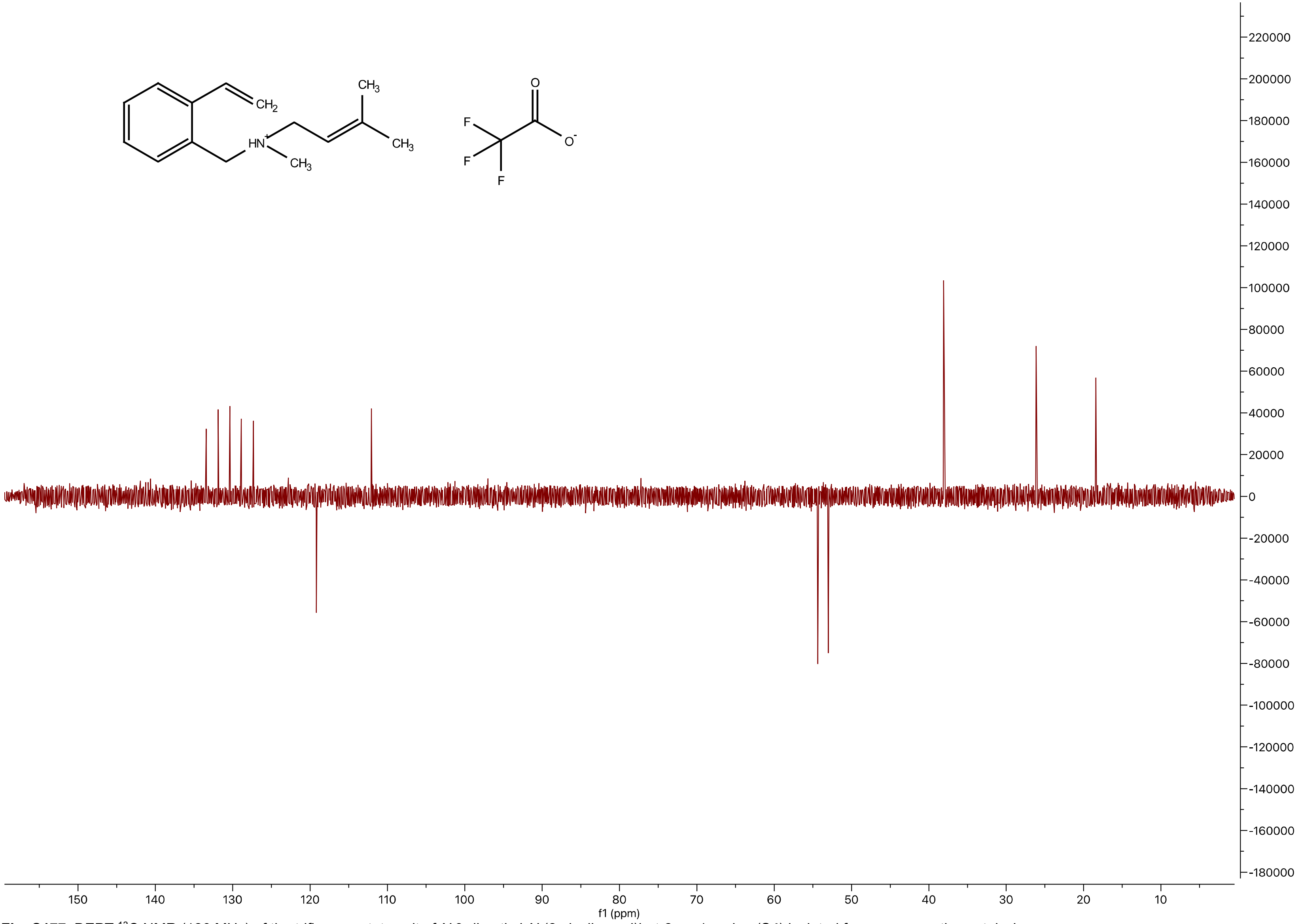

Fig. S177. DEPT ^{13}C NMR (126 MHz) of the trifluoroacetate salt of *N*,3-dimethyl-*N*-(2-vinylbenzyl)but-2-en-1-amine (**S4**) isolated from a preparative catalysis run.

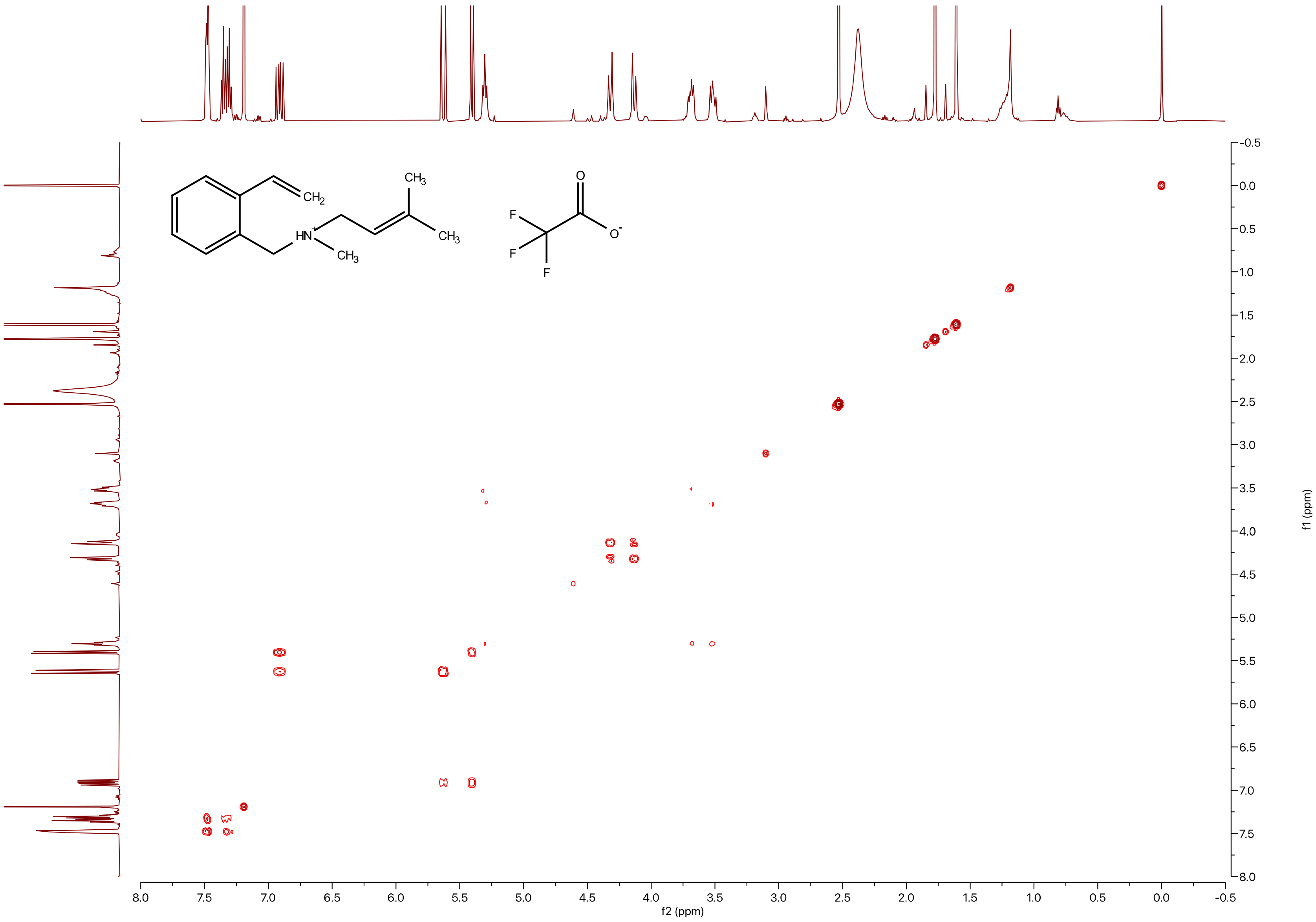

Fig. S178. ^{1}H COSY NMR of the trifluoroacetate salt of *N*,3-dimethyl-*N*-(2-vinylbenzyl)but-2-en-1-amine (**S4**) isolated from a preparative catalysis run.

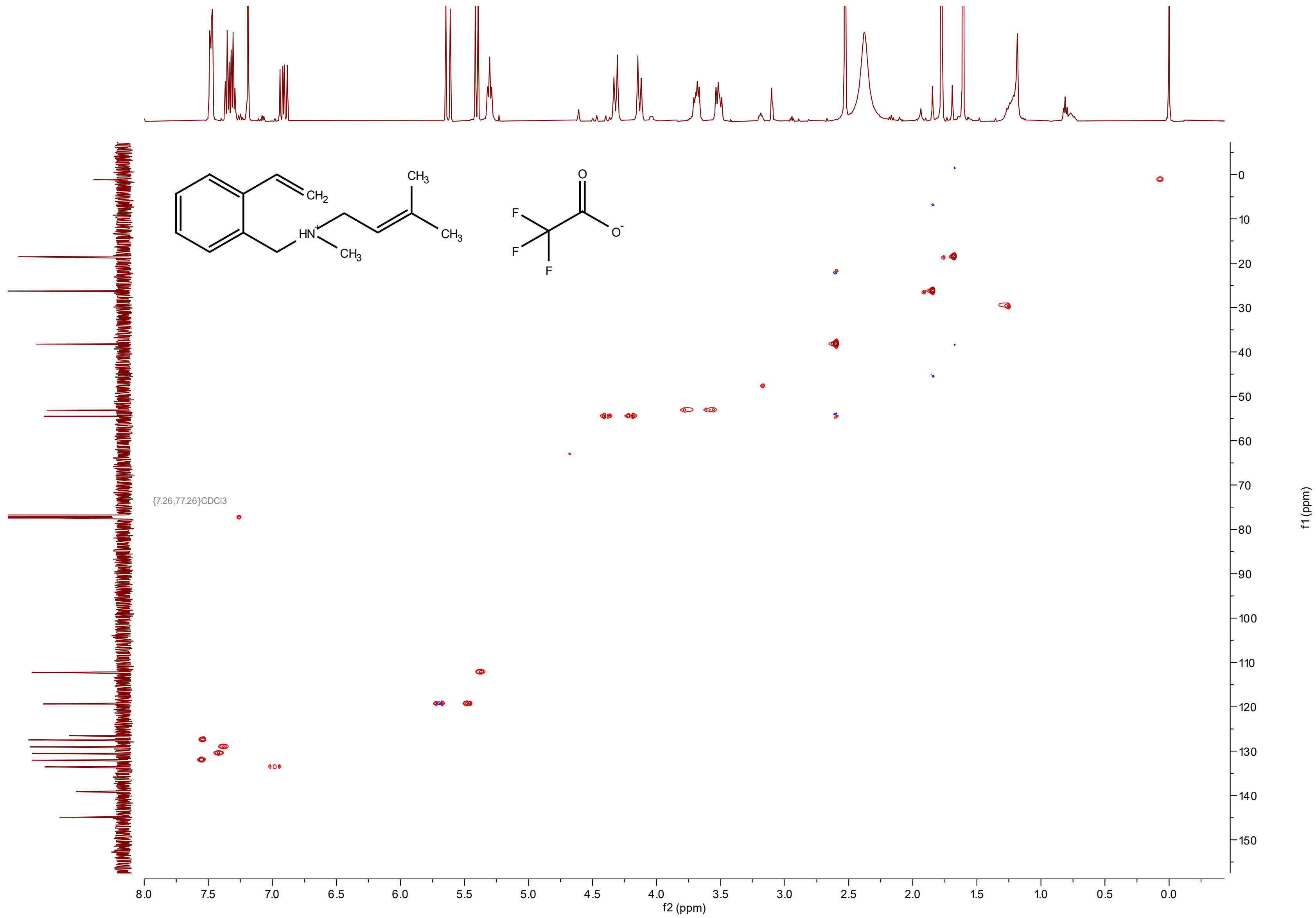

Fig. S179. ^{1}H -^{13}C HSQC NMR of the trifluoroacetate salt of *N*,3-dimethyl-*N*-(2-vinylbenzyl)but-2-en-1-amine (**S4**) isolated from a preparative catalysis run.

NMR spectra of the isolated from preparative reactions ammonium salts

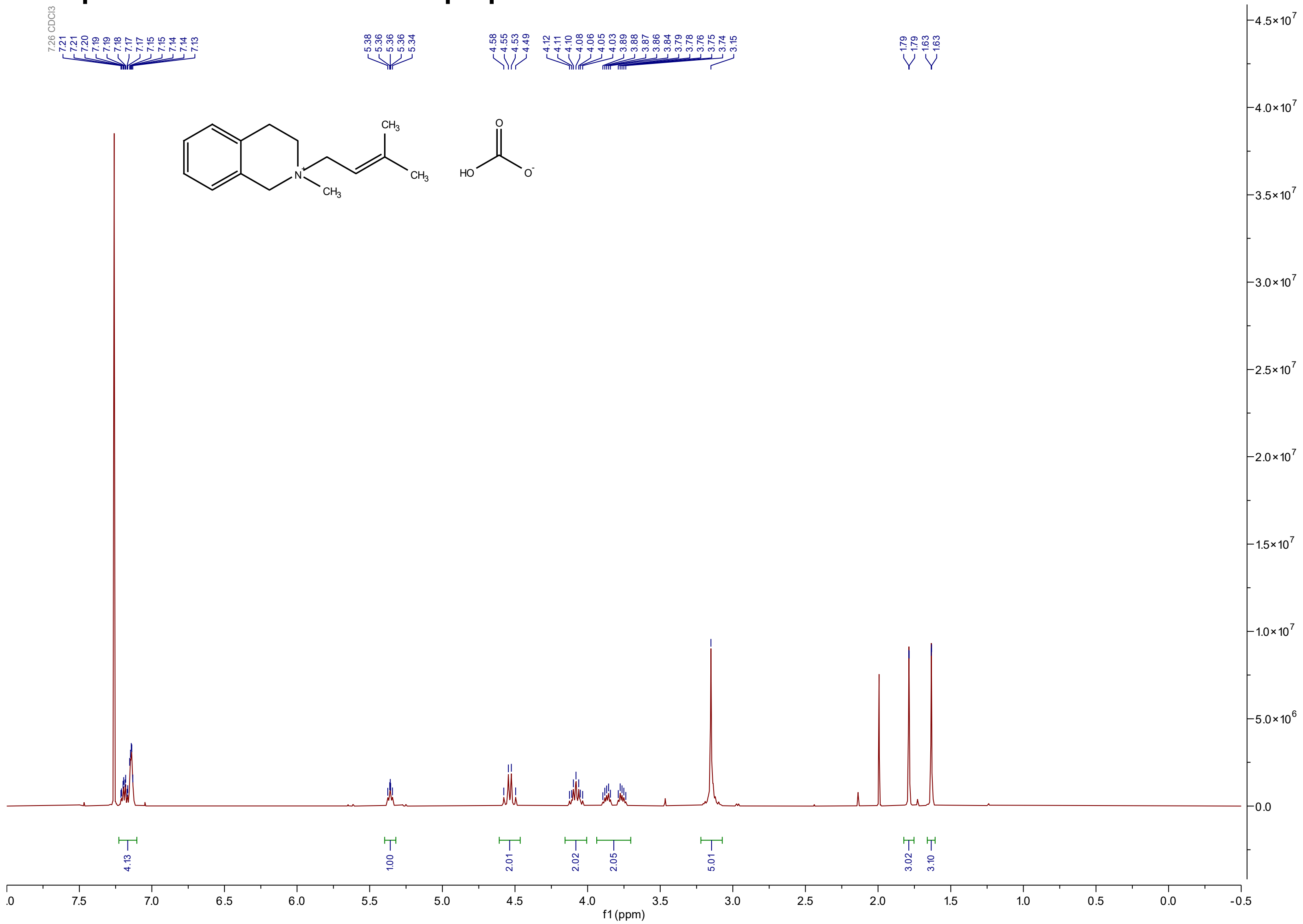

Fig. S180. ^{1}H NMR (500 MHz) of 2-methyl-2-(3-methylbut-2-en-1-yl)-1,2,3,4-tetrahydroisoquinolin-2-ium bicarbonate ([**3aa**][HCO_3]) in $CDCl_3$ isolated from a preparative catalysis run (1.0 mmol), work-up with water.

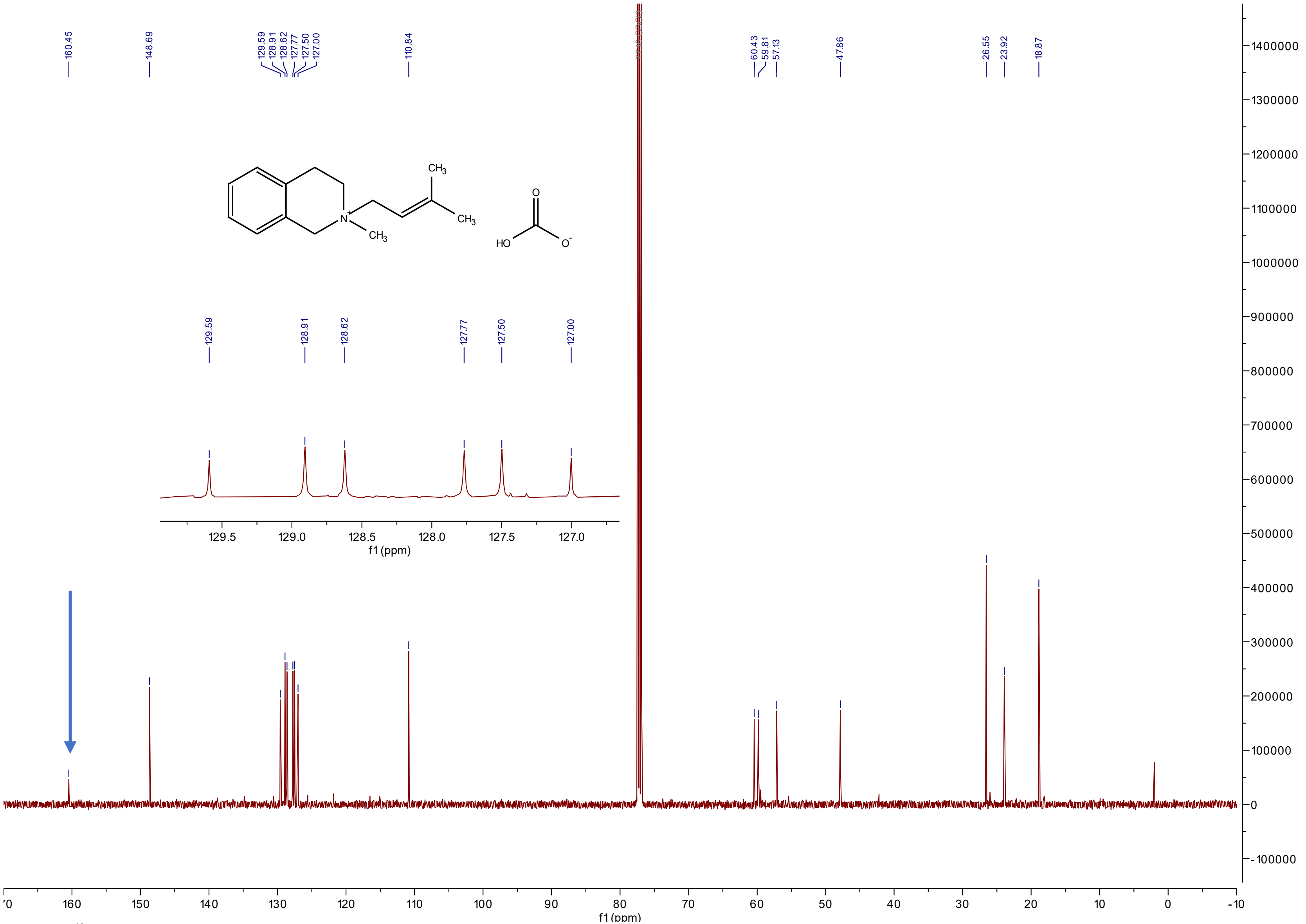

Fig. S181. ^{13}C NMR (126 MHz) of 2-methyl-2-(3-methylbut-2-en-1-yl)-1,2,3,4-tetrahydroisoquinolin-2-ium bicarbonate ([**3aa**][HCO_3]) in $CDCl_3$ isolated from a preparative catalysis run (1.0 mmol), work-up with water.

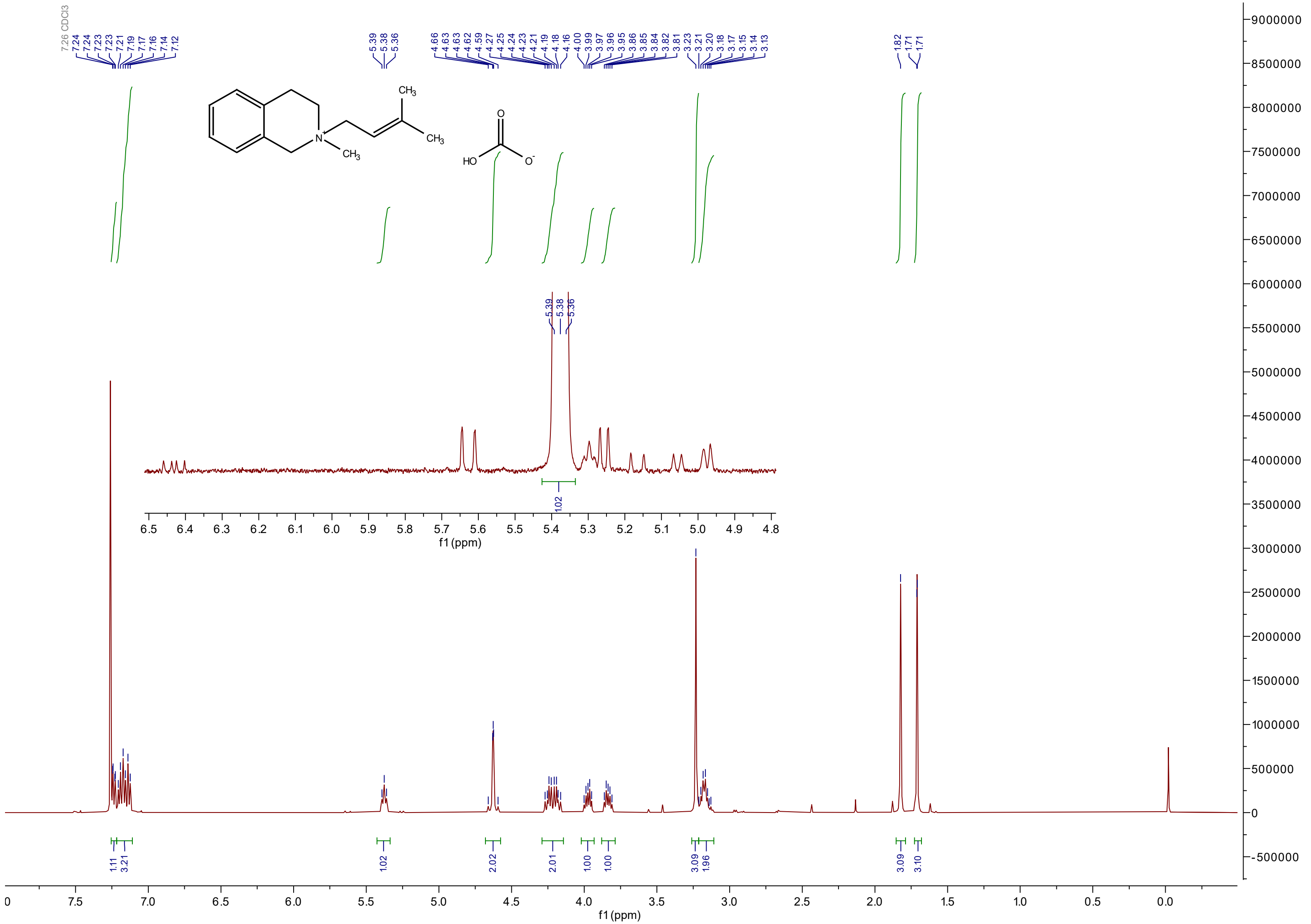

Fig. S182. ^{1}H NMR (500 MHz) of 2-methyl-2-(3-methylbut-2-en-1-yl)-1,2,3,4-tetrahydroisoquinolin-2-ium bicarbonate ([**3aa**][HCO_3]) in $CDCl_3$ isolated from a preparative catalysis run (0.26 mmol), work-up with water. Hofmann elimination product is still formed even when the evaporation is carried out at 20 °C (water bath temperature).

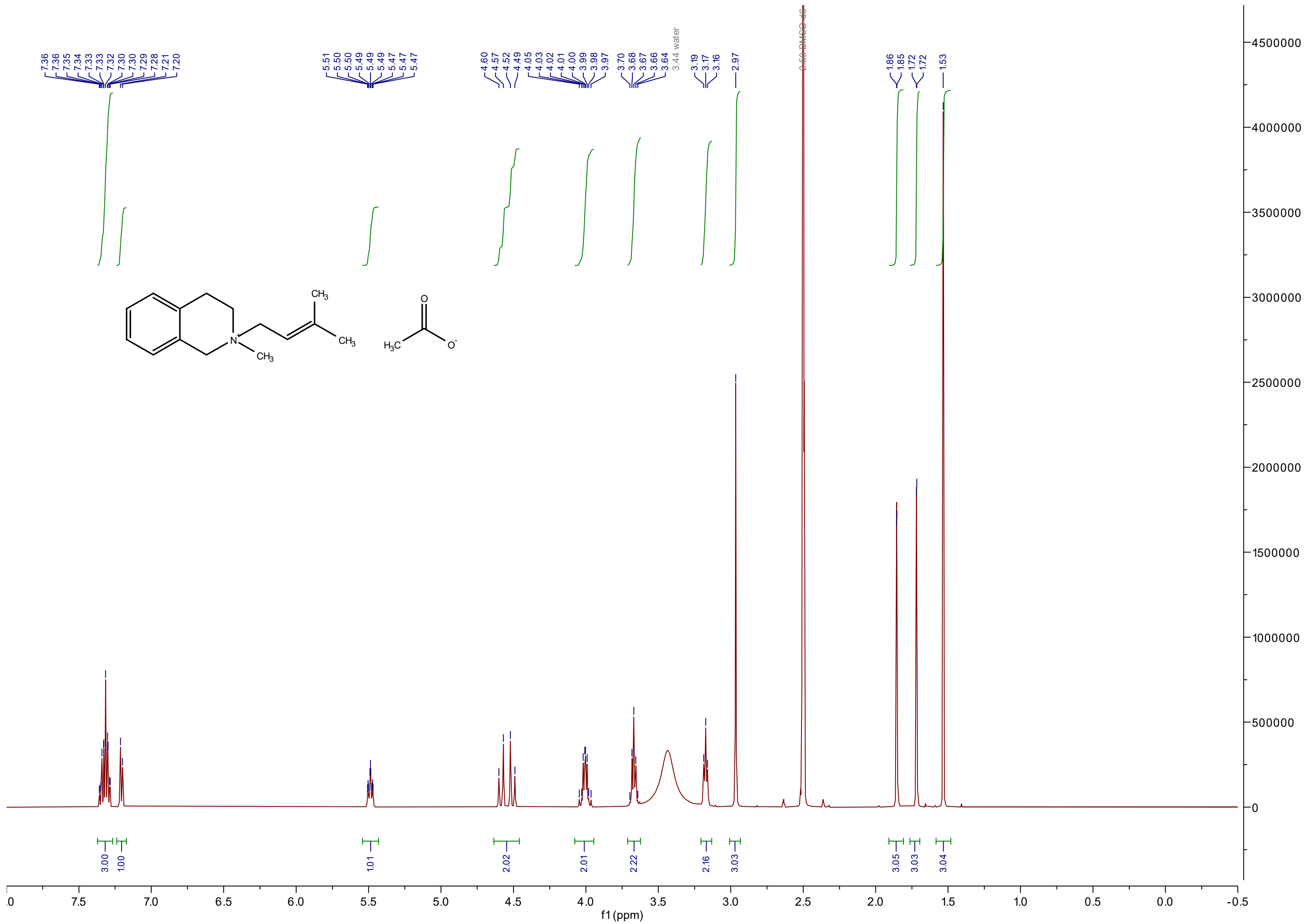

Fig. S183. ^{1}H NMR (500 MHz) of 2-methyl-2-(3-methylbut-2-en-1-yl)-1,2,3,4-tetrahydroisoquinolin-2-ium acetate ([**3aa**][OAc]) in DMSO-d_6, preparative catalysis run (0.26 mmol), work-up with basic ammonium acetate buffer.

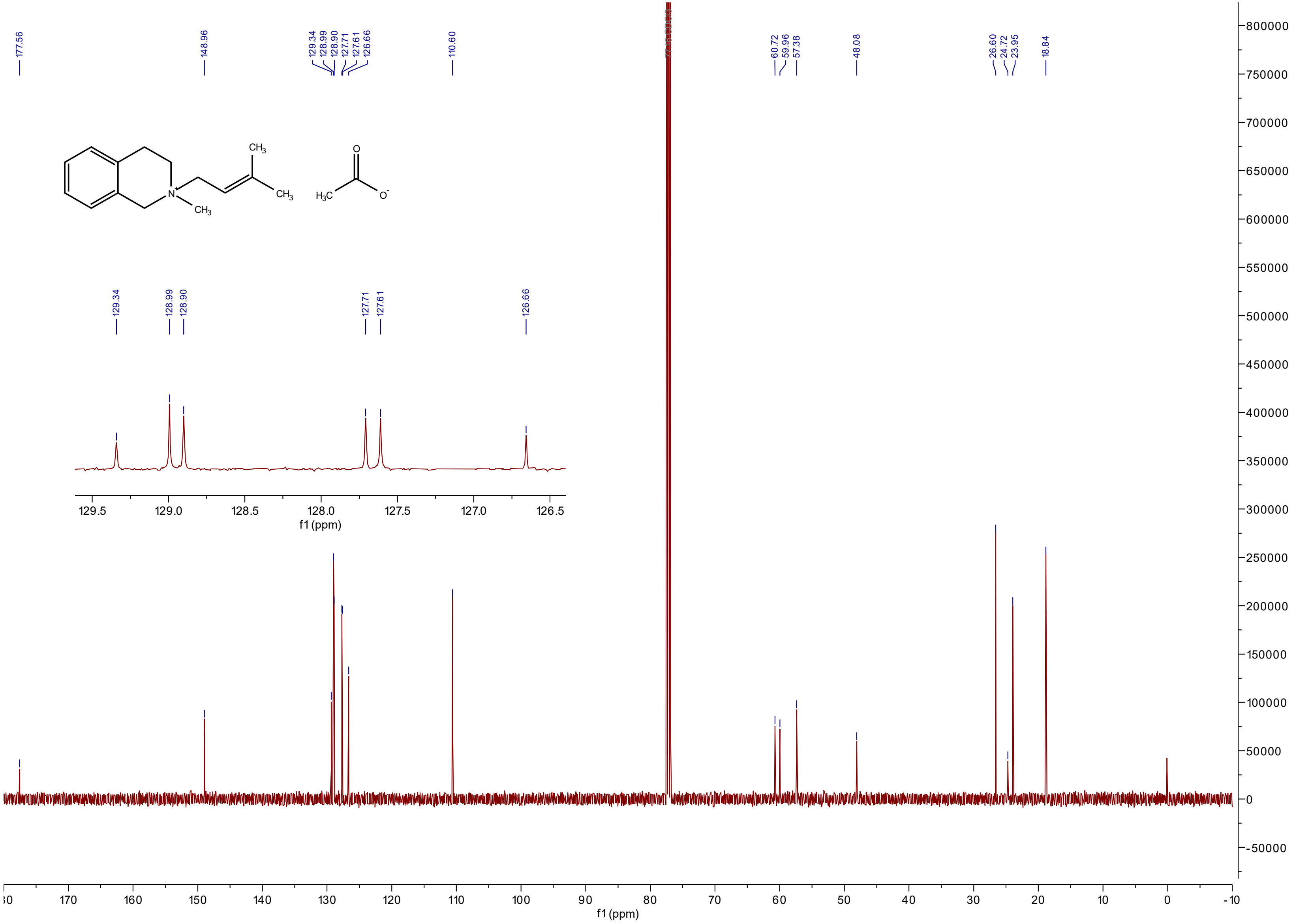

Fig. S184. ^{13}C NMR (126 MHz) of 2-methyl-2-(3-methylbut-2-en-1-yl)-1,2,3,4-tetrahydroisoquinolin-2-ium acetate ([**3aa**][OAc]) in DMSO-d_6, preparative catalysis run (0.26 mmol), work-up with basic ammonium acetate buffer.

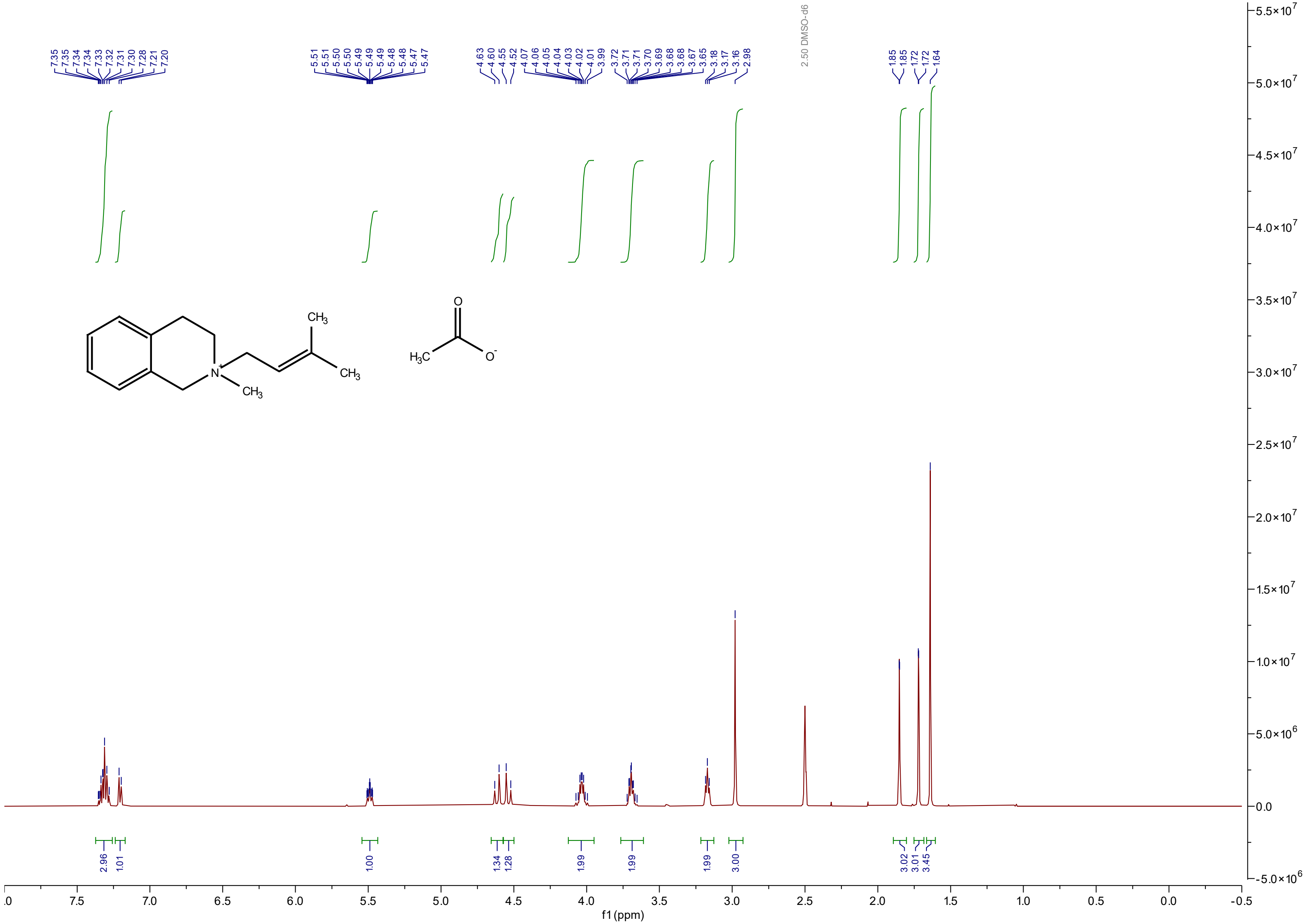

Fig. S185. ^{1}H NMR (500 MHz) of 2-methyl-2-(3-methylbut-2-en-1-yl)-1,2,3,4-tetrahydroisoquinolin-2-ium acetate ([**3aa**][OAc]) in DMSO-d_6, preparative catalysis run (1.0 mmol), work-up with basic ammonium acetate buffer.

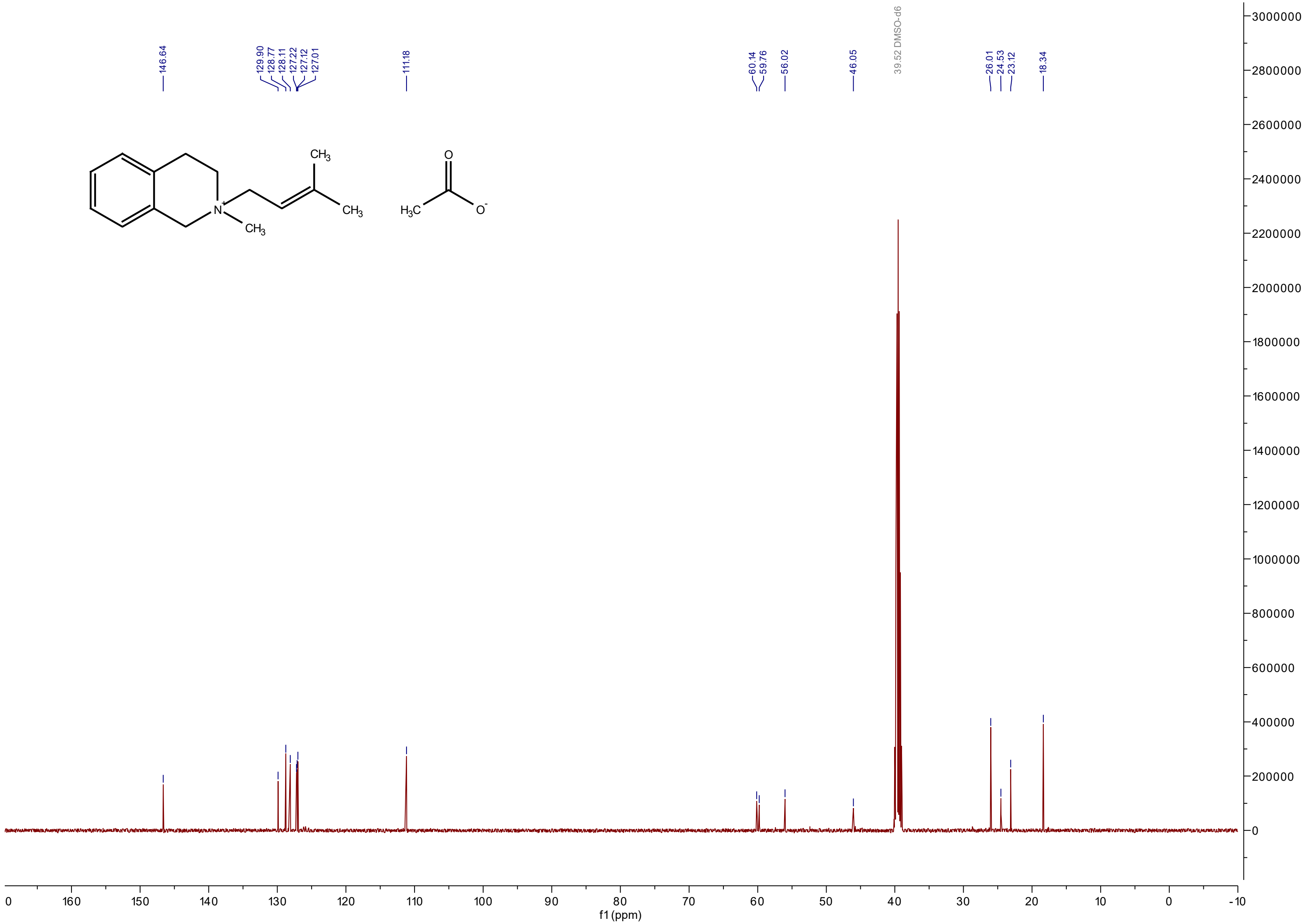

Fig. S186. ^{13}C NMR (126 MHz) of 2-methyl-2-(3-methylbut-2-en-1-yl)-1,2,3,4-tetrahydroisoquinolin-2-ium acetate ([**3aa**][OAc]) in DMSO-d$_6$, preparative catalysis run (1.0 mmol), work-up with basic ammonium acetate buffer.

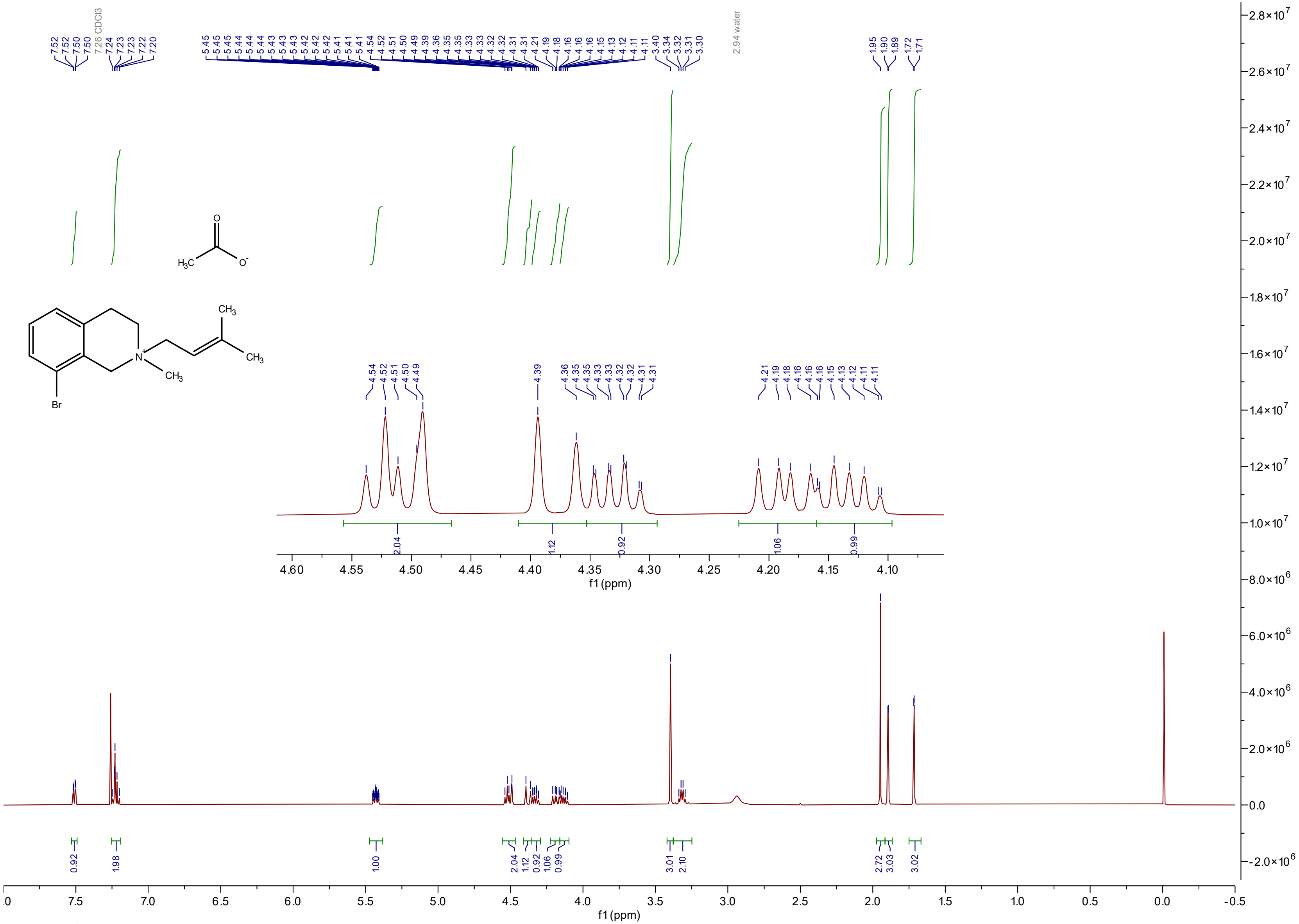

Fig. S187. ^{1}H NMR (500 MHz) of 8-bromo-2-methyl-2-(3-methylbut-2-en-1-yl)-1,2,3,4-tetrahydroisoquinolin-2-ium acetate ([**3ea**][OAc]) in $CDCl_3$, preparative catalysis run (0.22 mmol), work-up with ammonium acetate buffer.

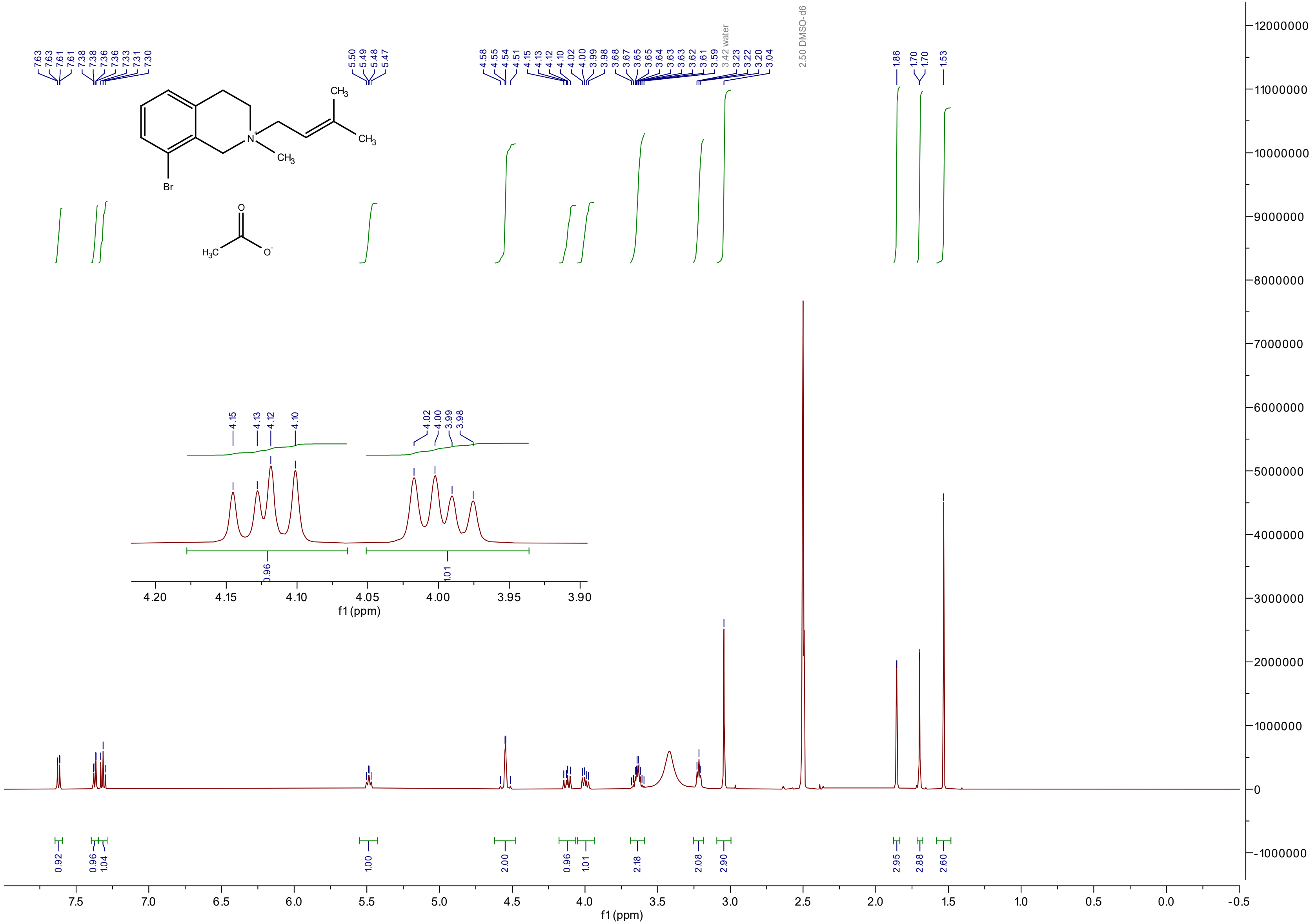

Fig. S188. ^{1}H NMR (500 MHz) of 8-bromo-2-methyl-2-(3-methylbut-2-en-1-yl)-1,2,3,4-tetrahydroisoquinolin-2-ium acetate ([**3ea**][OAc]) in DMSO-d$_6$, preparative catalysis run (0.22 mmol), work-up with ammonium acetate buffer.

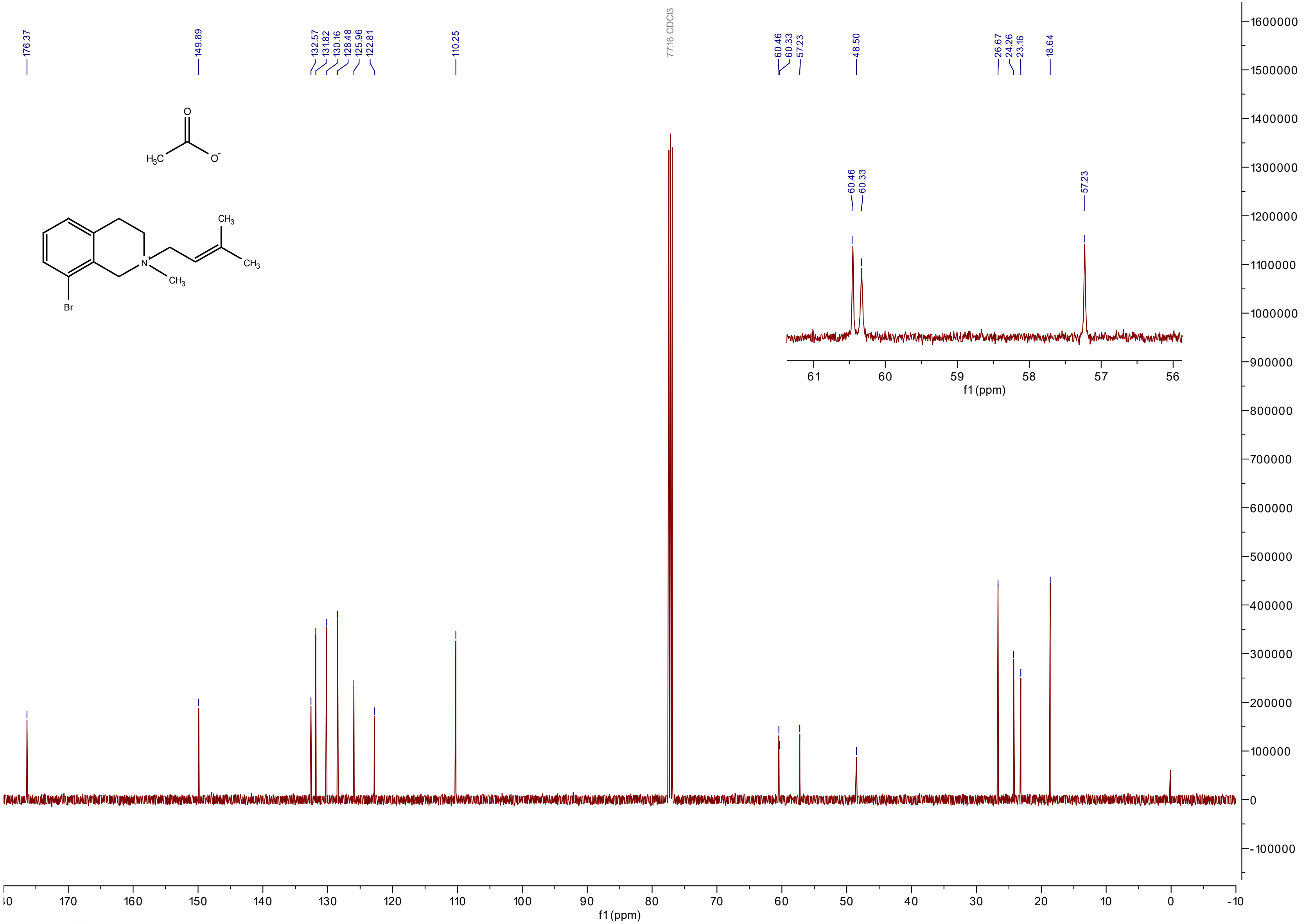

Fig. S189. ^{13}C NMR (126 MHz) of 8-bromo-2-methyl-2-(3-methylbut-2-en-1-yl)-1,2,3,4-tetrahydroisoquinolin-2-ium acetate ([**3ea**][OAc]) in $CDCl_3$, preparative catalysis run (0.22 mmol), work-up with ammonium acetate buffer.

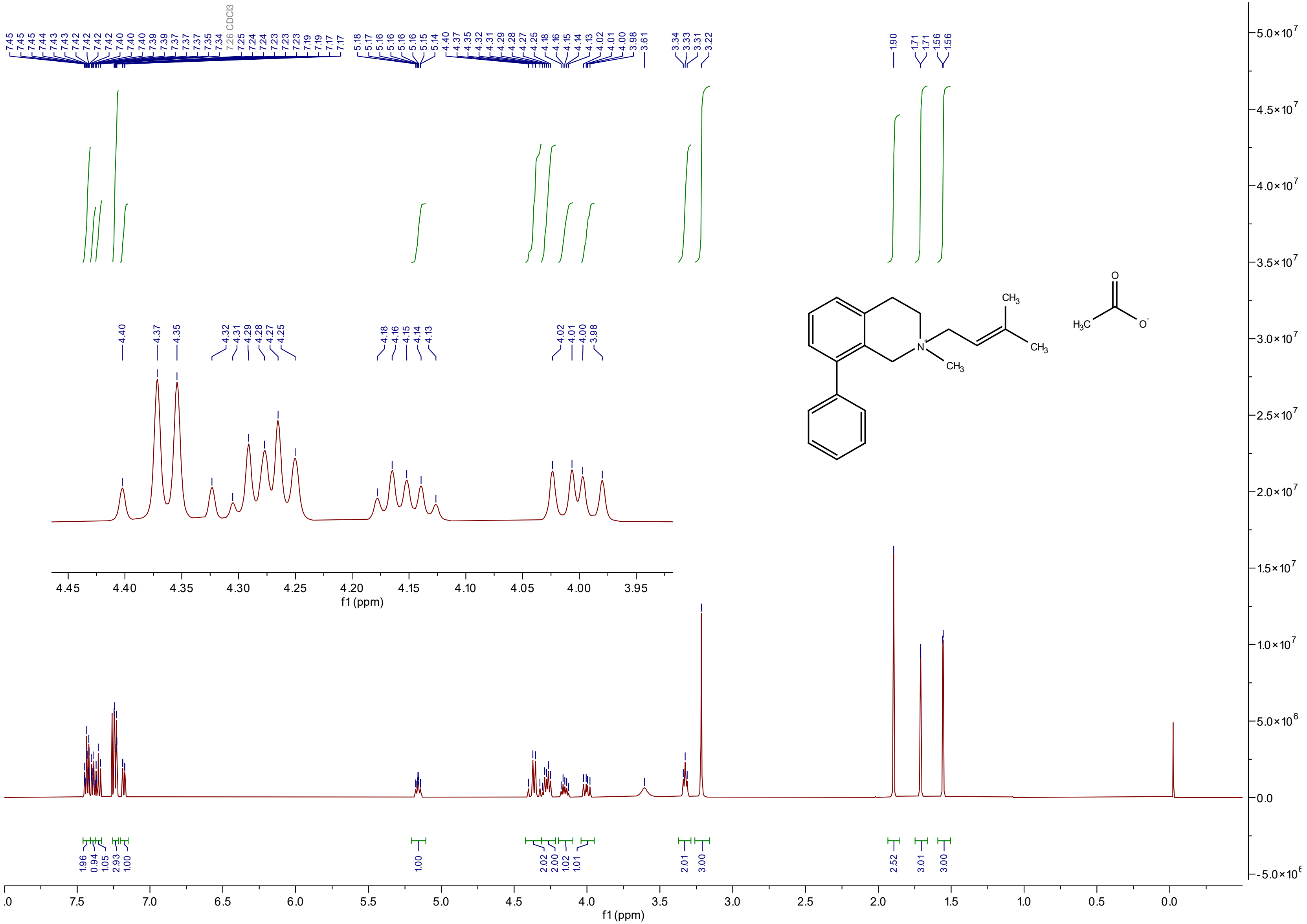

Fig. S190. ^{1}H NMR (500 MHz) of 2-methyl-2-(3-methylbut-2-en-1-yl)-8-phenyl-1,2,3,4-tetrahydroisoquinolin-2-ium acetate ([**3ka**][OAc]) in $CDCl_3$, preparative catalysis run (87 µmol), work-up with ammonium acetate buffer.

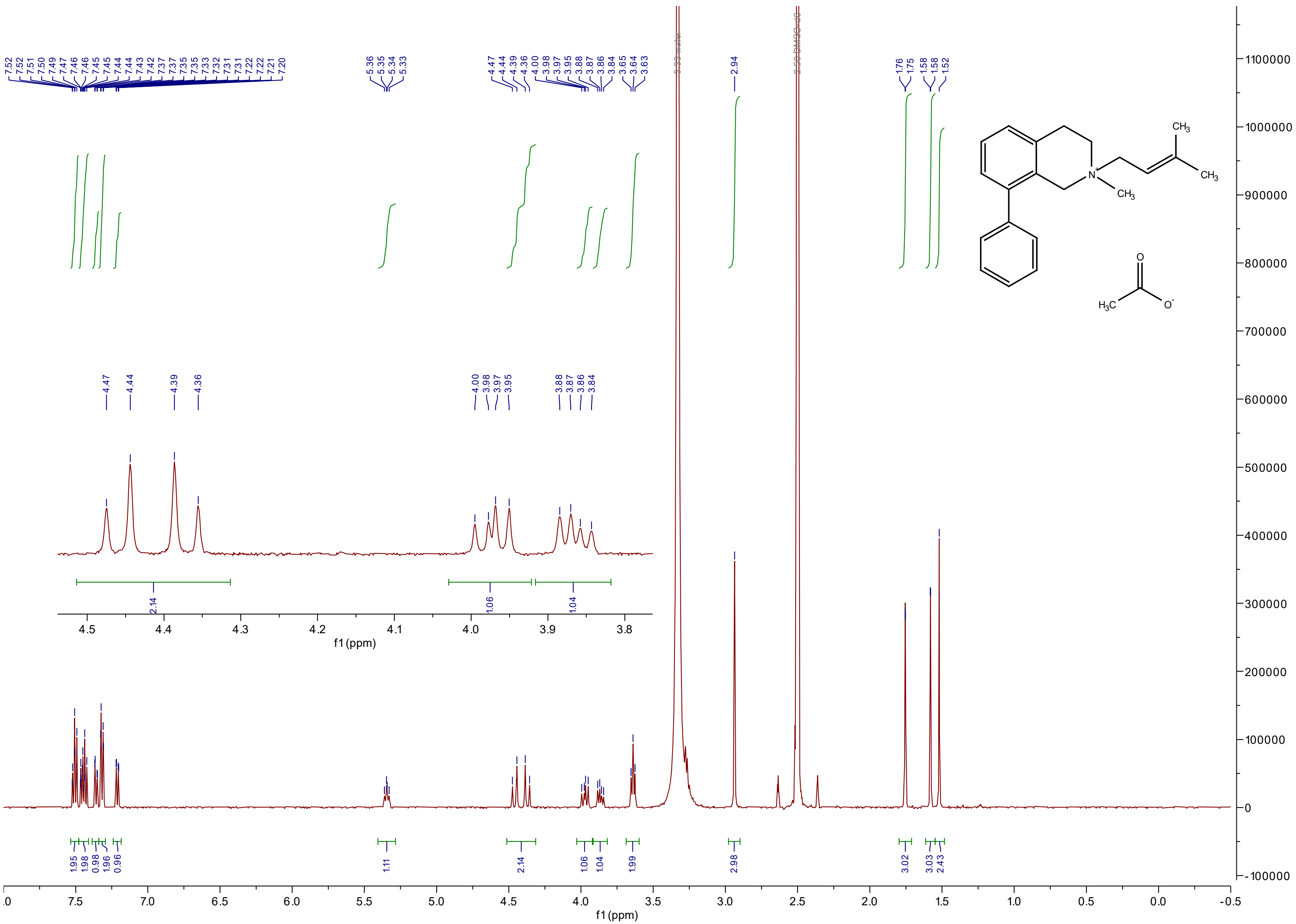

Fig. S191. ^{1}H NMR (500 MHz) of 2-methyl-2-(3-methylbut-2-en-1-yl)-8-phenyl-1,2,3,4-tetrahydroisoquinolin-2-ium acetate ([**3ka**][OAc]) in DMSO-d_6, preparative catalysis run (87 µmol), work-up with ammonium acetate buffer.

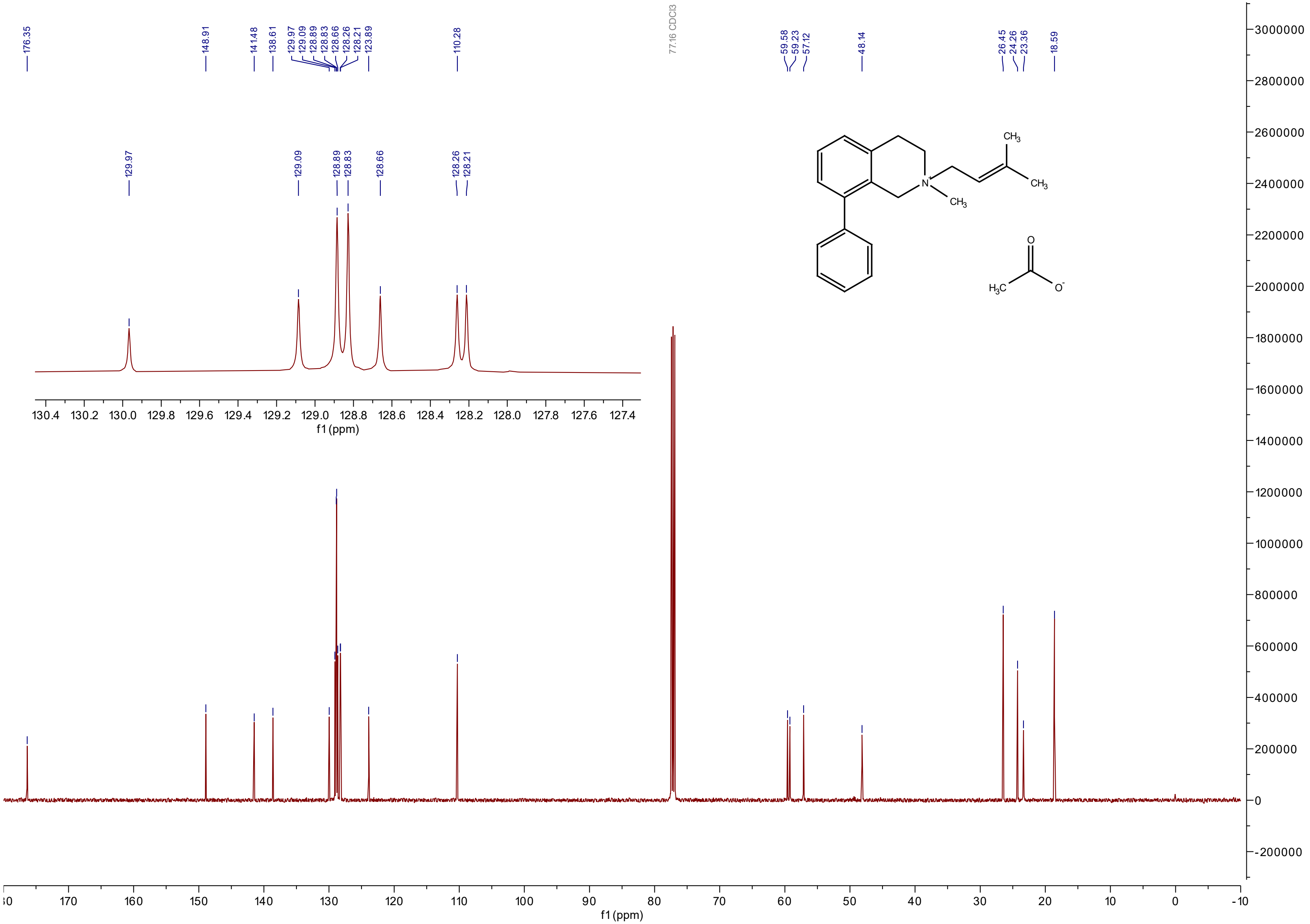

Fig. S192. ^{13}C NMR (126 MHz) of 2-methyl-2-(3-methylbut-2-en-1-yl)-8-phenyl-1,2,3,4-tetrahydroisoquinolin-2-ium acetate ([**3ka**][OAc]) in $CDCl_3$, preparative catalysis run (87 µmol), work-up with ammonium acetate buffer.

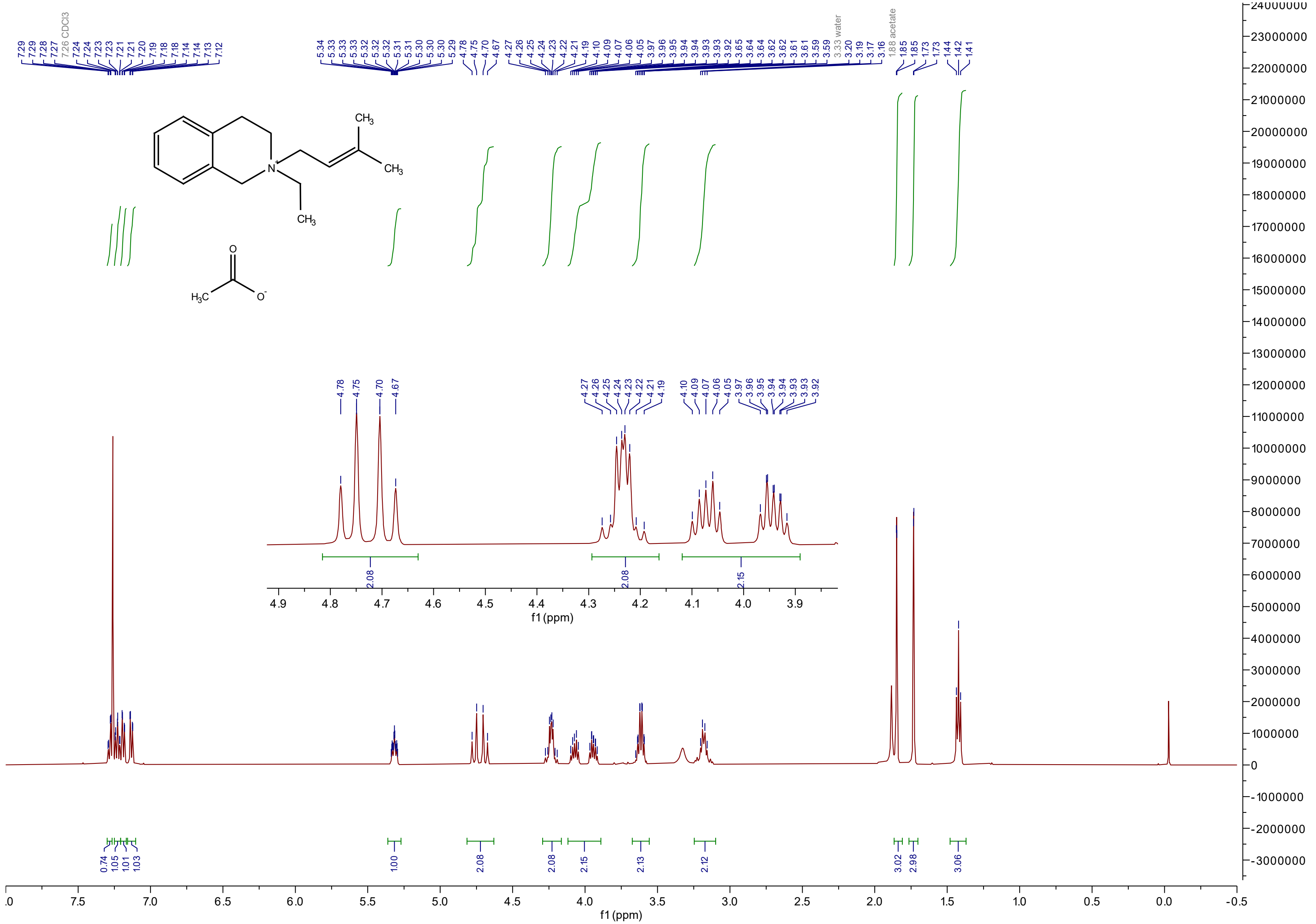

Fig. S193. ^{1}H NMR (500 MHz) of 2-ethyl-2-(3-methylbut-2-en-1-yl)-1,2,3,4-tetrahydroisoquinolin-2-ium acetate ([**3ra**][OAc]) in $CDCl_3$, preparative catalysis run (0.10 mmol), work-up with ammonium acetate buffer.

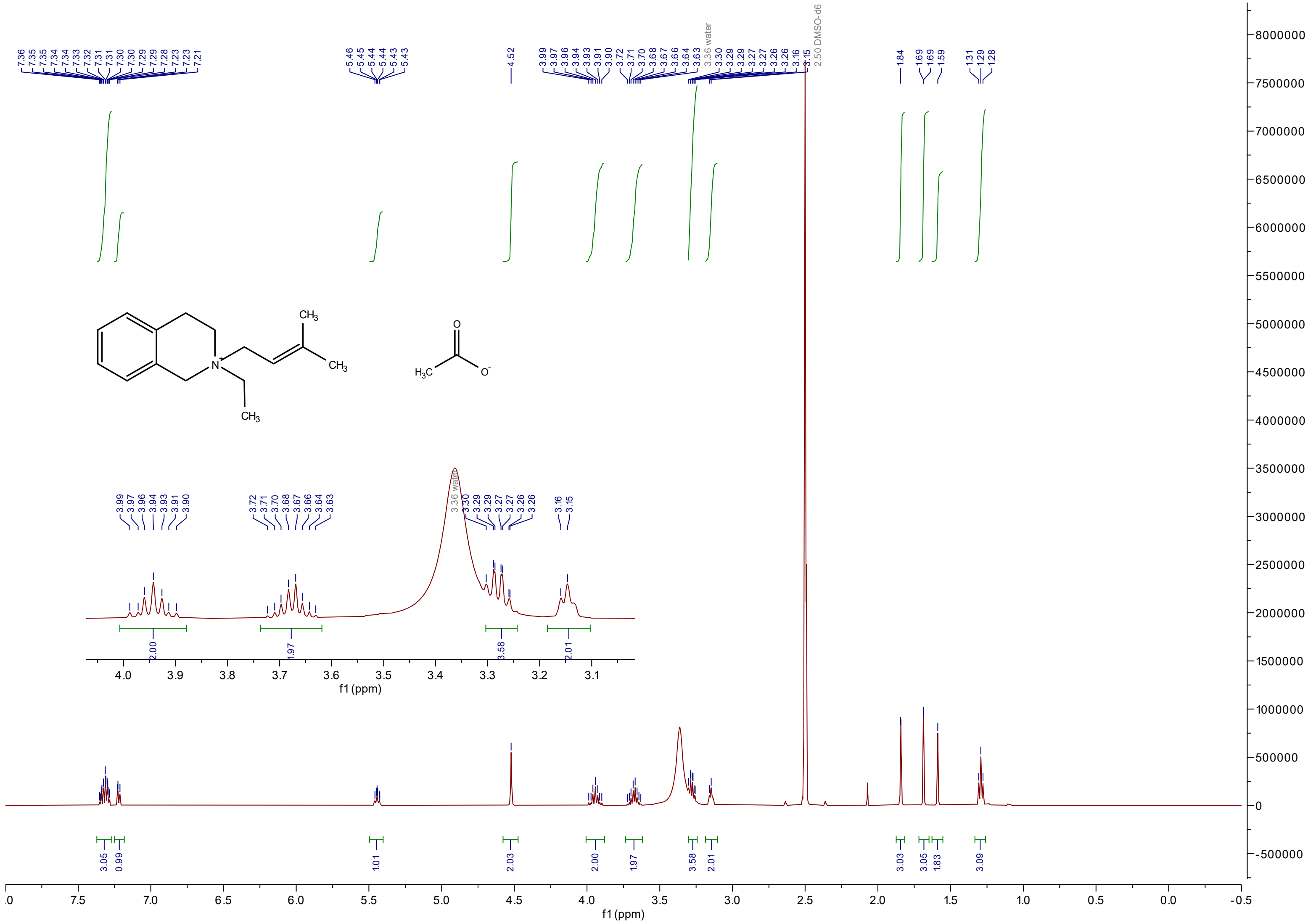

Fig. S194. ^{1}H NMR (500 MHz) of 2-ethyl-2-(3-methylbut-2-en-1-yl)-1,2,3,4-tetrahydroisoquinolin-2-ium acetate ([**3ra**][OAc]) in DMSO-d_6, preparative catalysis run (0.10 mmol), work-up with ammonium acetate buffer. The isolated product contained only 0.6 eq. of acetate as the counteranion; the remainder is likely bicarbonate.

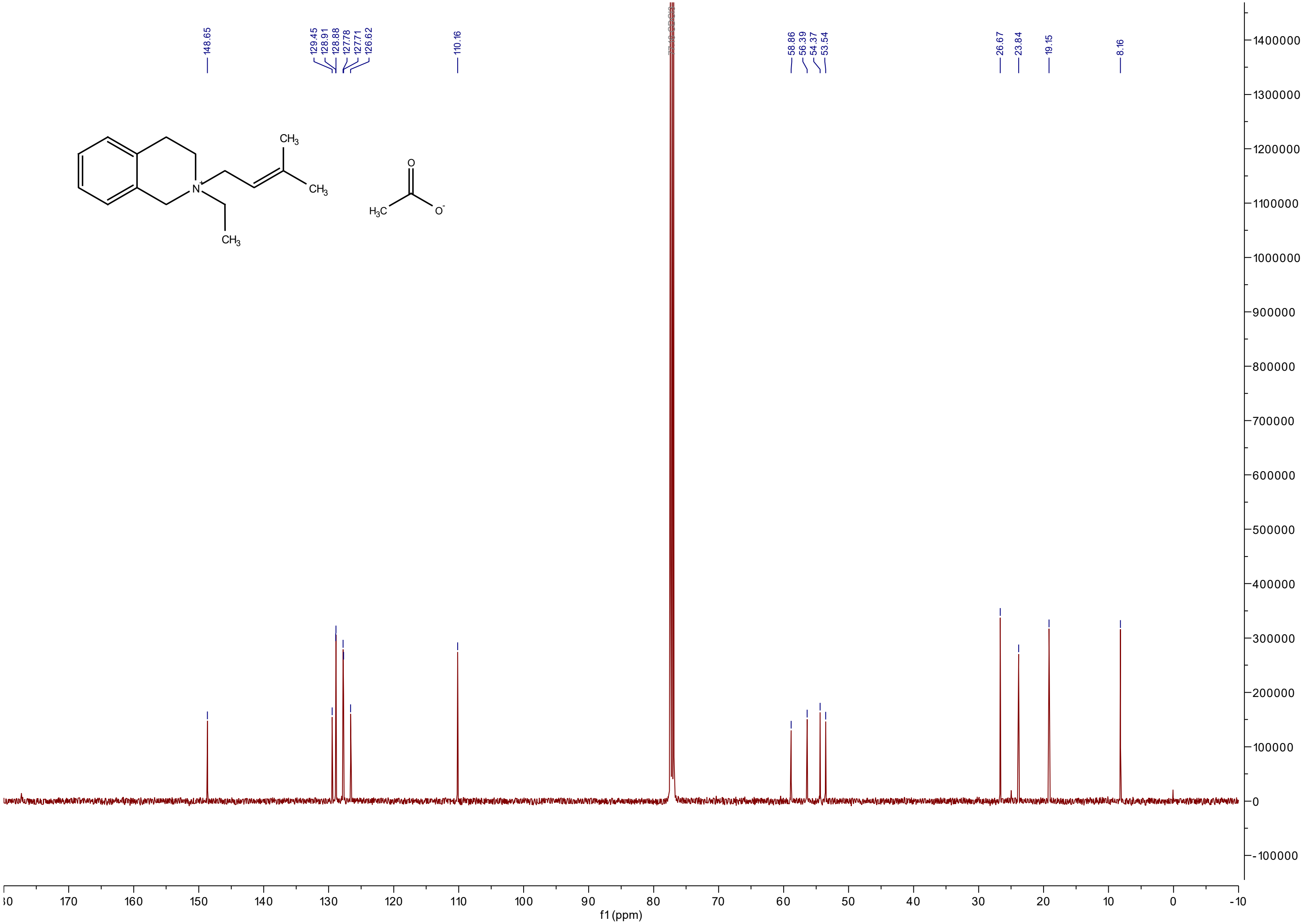

Fig. S195. ^{13}C NMR (126 MHz) of 2-ethyl-2-(3-methylbut-2-en-1-yl)-1,2,3,4-tetrahydroisoquinolin-2-ium acetate ([**3ra**][OAc]) in $CDCl_3$, preparative catalysis run (0.10 mmol), work-up with ammonium acetate buffer.

References

[1] a) W. Eschweiler, *Ber. Dtsch. Chem. Ges.* **1905**, *38*, 1, 880-882. b) H. T. Clarke, H. B. Gillespie, and S. Z. Weisshaus, *J. Am. Chem. Soc.* **1933**, *55*, 11, 4571–4587.

[2] R. Liang, S. Li, R. Wang, L. Lu, F. Li, *Org. Lett.* **2017**, *19*, 21, 5790-5793.

[3] P. Thapa, E. Corral, S. Sardar, B. S. Pierce, F. W. Foss Jr., *J. Org. Chem.* **2018**, *84*, 2, 1025-1034.

[4] A. R. Katritzky, N. G. Akhmedov, I. Ghiviriga, R. Maimait, *J. Chem. Soc., Perkin Trans. 2* **2002**, 1986-1993.

[5] A. Bonifazi, F. O. Battiti, J. Sanchez, S. A. Zaidi, E. Bow, M. Makarova, J. Cao, A. Basha Shaik, A. Sulima, K. C. Rice, V. Katritch, M. Canals, J. R. Lane, and A. H. Newman, *J. Med. Chem.* **2021**, *64*, 11, 7778–7808.

[6] a) C. Valente, and M.G. Organ, (**2011**). The Contemporary Suzuki–Miyaura Reaction. In Boronic Acids, D.G. Hall (Ed.). b) T. E. Barder, S. D. Walker, J. R. Martinelli and S. L. Buchwald, *J. Am. Chem. Soc*. **2005**, *127*, 13, 4685-4696. c) J. P. Wolfe, R. A. Singer, B. H. Yang and S. L. Buchwald, *J. Am. Chem. Soc*. **1999**, *121*, 41, 9550-9561.

[7] Y. Dai, F. Wu, Z. Zang, H. You, and H. Gong, *Chem. Eur. J*., **2012**, *18*, 808-812.

[8] T. Schlatzer, J. Kriegesmann, H. Schröder, M. Trobe, C. Lembacher-Fadum, S. Santner, A. V. Kravchuk, C. F. W. Becker, R. Breinbauer, *J. Am. Chem. Soc.* **2019**, *141*, 37, 14931-14937.

[9] N. R. Lee, F. A. Moghadam, F. C. Braga, D. J. Lippincott, B. Zhu, F. Gallou, B. H. Lipshutz, *Org. Lett.* **2020**, *22*, 13, 4949-4954.

[10] A. Horn, U. Kazmaier, *Org. Lett.* **2019**, *21*, 12, 4595-4599.

[11] Y. Yu, H. Chen, Q. Qian, K. Yao, H. Gong, *Tetrahedron* **2018**, *74*, 39, 5651-5658.

[12] T. Schlatzer, H. Schröder, M. Trobe, C. Lembacher-Fadum, S. Stangl, C. Schlögl, H. Weber, R. Breinbauer, *Adv. Synth. Catal.* **2020**, *362*, 331-336.

[13] a) F. Olivito, N. Amodio, M. L. Di Gioia, M. Nardi, M. Oliverio, G. Juli, P. Tassone, A. Procopio, *Med. Chem. Commun.* **2019**, *10*, 116-119. b) J. L. Ward, M. H. Beale, *J. Chem. Soc., Perkin Trans. 1* **2002**, 710-712. c) K. Yasui, K. Fugami, S. Tanaka, Y. Tamaru, *J. Org. Chem.* **1995**, *60*, 5, 1365-1380. d) M. Berger, D. Carboni, P. Melchiorre, *Angew. Chem. Int. Ed.* **2021**, *60*, 26373-26377.

[14] L. Dai, Z.-H. Xia, Y.-Y. Gao, Z.-H. Gao, S. Ye, *Angew. Chem. Int. Ed.* **2019**, *58*, 18124-18130.

[15] L. Xu, Z. Liu, W. Dong, J. Song, M. Miao, J. Xu, H. Ren, *Org. Biomol. Chem.* **2015**,*13*, 6333-6337.

[16] K. D. Reichl, N. L. Dunn, N. J. Fastuca, A. T. Radosevich, *J. Am. Chem. Soc.* **2015**, *137*, 16, 5292–5295.

[17] a) 3-ethylpent-1-en-3-ol as in H. T. Dang, V. T. Nguyen, V. D. Nguyen, H. D. Arman, O. V. Larionov, *Org. Biomol. Chem.* **2018**,*16*, 3605-3609. b) 1-bromo-3-ethylpent-2-ene as in G. V. Rao, B. N. Swamy, V. Chandregowda, G. C. Reddy, *Eur. J. Med. Chem*. **2009**, *44*, 5, 2239-2245.

[18] N. J. Race, J. F. Bower, *Org. Lett.* **2013**, *15*, 17, 4616–4619.

[19] A. T. Soldatenkov, S. A. Soldatova, J. A. Mamyrbekova-Bekro. et al. *Chem Heterocycl Comp,* **2012**, *48*, 1332–1339.

[20] L. G. Judin, A. N. Kost, Yu. A. Berlin, A. E. Shipov, *Zhurnal Obshchei Khimii*, **1957**, *27*, 3021-3026.

[21] Predicted ACD/Labs V11.02.

[22] a) I. L. Rakhmankulov, E. A. Kantor, D. L. Rakhmankulov, *J. Appl. Chem. USSR*, **1980**, *53*(6), 1093-1097. b) Y.-R. Naves, Bull. Soc. Chim. Fr., **1971**, 886 – 888.

[23] D. E. Adelson, H. Dannenberg. *U.S. Patent 2595214*, **1952.**

[24] A. N. Kost, A. M. Yurkevich, *Zhurnal Obshchei Khimii*, **1953**, *23*, 1738-1743.

[25] T. G. Kyazimova, E. G. Mamedov, R. S. Babaev, I. M. Mamedova, *Russ. J. Appl. Chem.* **2008**, *81*(3), 438-442.

[26] P. A. Evans, S. Oliver, J. Chae, *J. Am. Chem. Soc.* **2012**, *134*(47), 19314-19317.

[27] G. M. Sheldrick, *Acta Cryst.* **2015**, *A71*, 3-8.

[28] O. V. Dolomanov, L. J. Bourhis, R. J. Gildea, J. A. K. Howard, H. Puschmann, *J. Appl. Cryst.* **2009**, *42*, 339-341.

[29] G. M. Sheldrick, *Acta Cryst.* **2015**, *C71*, 3-8.

[30] P. Coppens in *Crystallographic Computing* (Eds.: F. R. Ahmed), Munksgaard, Copenhagen, **1970**, pp. 255 - 270.

[31] J. Koziskova, F. Hahn, J. Richter, J. Kozisek, *Acta Chim. Slov.* **2016**, *9*(2), 136 - 140.
